# Synthesis of 2,6-*trans*-Tetrahydropyrans Using a Palladium-Catalyzed Oxidative Heck Redox-Relay Strategy

**DOI:** 10.1021/acs.orglett.3c03866

**Published:** 2024-01-10

**Authors:** Holly E. Bonfield, Colin M. Edge, Marc Reid, Alan R. Kennedy, David D. Pascoe, David M. Lindsay, Damien Valette

**Affiliations:** † Department of Pure and Applied Chemistry, University of Strathclyde, Glasgow G1 1XL, U.K.; ‡ Drug Substance Development, GSK, Gunnels Wood Road, Stevenage, Hertfordshire SG1 2NY, U.K.

## Abstract

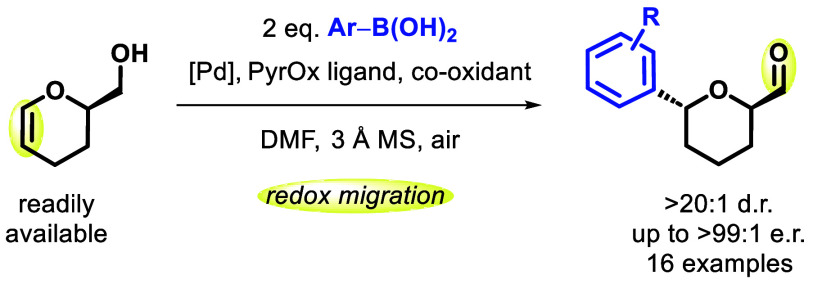

The C-aryl-tetrahydropyran motif is prevalent in nature in a number of natural products with biological activity; however, this challenging architecture still requires novel synthetic approaches. We demonstrate the application of a stereoselective Heck redox-relay strategy for the synthesis of functionalized 2,6-*trans*-tetrahydropyrans in excellent selectivity in a single step from an enantiopure dihydropyranyl alcohol, proceeding through a novel *exo*-cyclic migration. The strategy has also been applied to the total synthesis of a *trans*-epimer of the natural product centrolobine in excellent yield and stereoselectivity.

Over the past decade, it has been shown that stereogenic centers can be installed in positions remote from other functionalities in acyclic alkenol systems with high stereoselectivity, via palladium-catalyzed Heck-type redox-relay processes.^1^ Following the stereoselective formation of the new C–C bond, the palladium catalyst migrates along the alkyl chain toward the alcohol via successive *syn*-β-hydride elimination/*syn*-migratory insertion steps, termed a “chain walk”, terminating with an oxidative deprotonation step that ultimately delivers the corresponding aldehyde or ketone (Figure 1a).^2^ Since the seminal publication of this strategy by Sigman and co-workers in 2012,^3^ the scope has been expanded significantly for acyclic systems.^1^ In particular, the alkenylation of acyclic *O*-aryl enol ethers via a Heck redox-relay strategy has been demonstrated by both Sigman and Correia, using alkenyl triflates and aryl diazonium salts, respectively (Figure 1b).^4−6^ Oxidative Heck redox-relay processes are also possible, employing boronic acids instead of halides or pseudohalides. Application of this approach to lactams (Figure 1c)^7^ results in arylation α to the nitrogen atom, followed by partial migration around the ring, furnishing the α,β-unsaturated lactam product.

**Figure 1:**
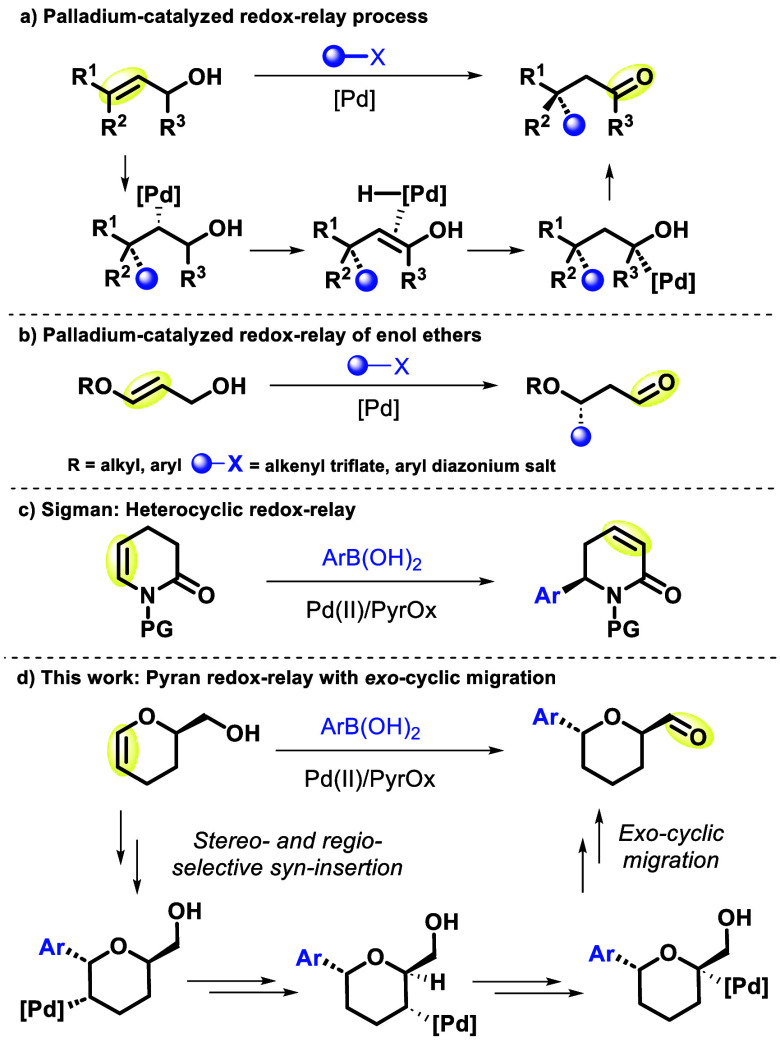
Heck redox-relay processes and a proposed strategy for accessing 2,6-disubstituted tetrahydropyrans.

Since 2009, the University of Strathclyde and GSK have engaged in a collaborative M.Phil./Ph.D. program. This new model of industry/academia partnership supports GSK employees and new graduates to embark on research in a broad range of scientific areas, from chemical biology to process development.^8^ As part of this collaborative endeavor, we were inspired to investigate whether the Heck redox-relay strategy could be applied to 6-(hydroxymethyl)-2,3-dihydropyranyl (DHP) alcohols (Figure 1d).

Requiring an ambitious and unprecedented *exo*-cyclic migration process,^9^ this approach would represent a new and complementary strategy for accessing 2,6-disubstituted tetrahydropyrans (THPs),^10−14^ which are C(sp^3^)-rich, biologically relevant,^15^ and medicinally important motifs.^16,17^ Herein, we disclose the successful realization of this novel approach.

We initiated our study with enantiomerically pure DHP-alcohol, (*R*)-**1** (>99:1 er), which is readily available from racemic DHP-alcohol *rac*-**1** via enzymatic resolution on a multigram scale (Scheme 1).^18^ Pleasingly, reaction of (*R*)-**1** with *p*-fluorophenylboronic acid, under conditions similar to those previously reported for oxidative Heck redox-relay reactions^7^ [Pd(MeCN)_2_(OTs)_2_, PyrOx ligand **L0**, Cu(OTf)_2_, open to air],^19^ validated our proposed strategy, with formation of the desired, product-derived, alcohol **3a** as a single diastereoisomer in 46% yield and 97:3 er.

**Scheme 1:**
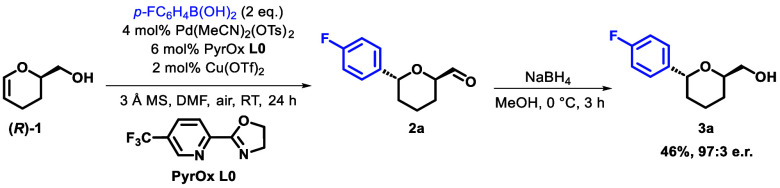
Heck Redox-Relay Reaction on a Dihydropyranyl Alcohol

Having identified preliminary conditions for the stereoselective C–C bond formation, we first chose to investigate any potential substrate/catalyst match/mismatch effects in the presence of a chiral ligand by observing the formation of the desired aldehyde in reactions of (*R*)-**1** and (*S*)-**1** with PyrOx ligands (*S*)-**L1** and (*R*)-**L1** using ^19^F NMR spectroscopy (Figure 2).^20^ High yields of the desired *trans*-THP **2a** confirmed that (*R*)-**1** and (*S*)-**L1** are a matched pair, as are (*S*)-**1** and (*R*)-**L1**. For the mismatched catalyst/ligand pairs, complete consumption of the starting material was observed, while the aldehyde product was generated only in small quantities (∼10%). It is suspected that under these conditions, a nonligand controlled addition to the opposite face of the alkene also occurs, resulting in products derived from partial migration. On this basis, a classical kinetic resolution, where one enantiomer of starting material is converted into the product and the other is left in enriched form, proved to be challenging. While the product was observed in high enantioselectivity, using *rac*-**1**, enantioenriched starting material was not recovered.^19^

**Figure 2:**
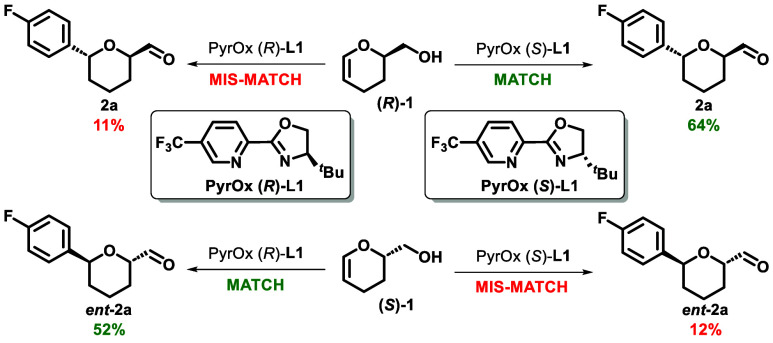
Match/mismatch data with enantiopure DHP-alcohol and PyrOx **L1**. Conditions: 2 equiv of *p*-FC_6_H_4_B(OH)_2_, 10 mol % Pd(MeCN)_2_(OTs)_2_, 15 mol % PyrOx (*S*)- or (*R*)-**L1**, 4 mol % Cu(OTf)_2_, DMF (0.1 M), 3 Å MS, air, room temperature.

We next undertook an optimization study to probe all components of the reaction process (Table 1). No improvement in yield was observed when a control reaction was performed under an oxygen atmosphere (entry 2). Two equivalents of boronic acid proved to be optimal, with 1 equiv leading to a decreased yield due to competing side reactions (homocoupling, protodeborylation, and phenol formation, entry 1 vs entry 3) and 3 equiv delivering no further increase in yield (entry 4). Control reactions in the absence of palladium and copper or in the absence of palladium only (entry 5 or 6, respectively) confirmed that the palladium(II) species is the active metal catalyst. In the absence of copper(II) triflate, only the rate of the reaction was reduced, but a comparable yield was attained after 24 h compared to standard conditions (entry 7). The exclusion of oxygen or removal of molecular sieves from the reaction led to significantly diminished solution yields, reaching only 9–11% after 24 h (entries 8 and 9).^21^ Conversely, the addition of 1 equiv of water had a positive influence on the reaction (entry 10), increasing the yield to 76%.

**Table 1:**
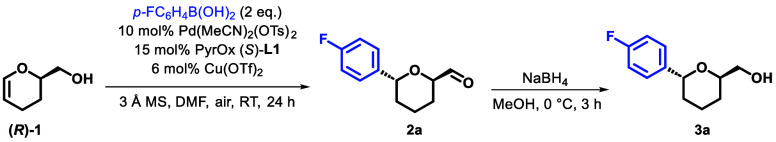
Investigation of the Reaction Parameters^a^

entry	deviation from the standard conditions	yield (%)^b^	er^c^
1	none	67	>99:1
2	oxygen atmosphere	50	–
3	1 equiv of boronic acid	26	–
4	3 equiv of boronic acid	64	–
5	no Pd, no Cu(OTf)_2_	0	–
6	no Pd, 10 mol % Cu(OTf)_2_	0	–
7	no Cu(OTf)_2_	63	–
8	nitrogen atmosphere	9	–
9	no MS	11	–
10	1 equiv of water	76	99:1
11^e^	6:10:3 Pd:PyrOx:Cu mole ratio	80 (56^d^)	99:1
12^e^	4:6:2 Pd:PyrOx:Cu mole ratio	77 (59^d^)	>99:1
13^e^	10:15:4 Pd(OAc)_2_:PyrOx:Cu mole ratio	84 (70^d^)	99:1

a Conditions: 2 equiv of boronic acid, 10 mol % Pd(MeCN)_2_(OTs)_2_, 15 mol % PyrOx **L1**, 4 mol % Cu(OTf)_2_, no water, 3 Å molecular sieves, air, unless otherwise stated.

b The 24 h solution yield of **2a** determined by ^19^F{^1^H} NMR.

c Enantiomeric ratio determined following reduction of aldehyde **2a** to the corresponding alcohol, **3a**.

d Isolated yield following reduction of aldehyde **2a** to the corresponding alcohol, **3a**.

e With 1 equiv of water.

With this water additive, the palladium:PyrOx (*S*)-**L1**:copper loading could be successfully reduced to 4:6:2 (mole percent) while maintaining the excellent yield (entries 11 and 12). Progressing with the lowest catalyst loading (entry 12), 2,6-*trans*-THP derivative **2a** was subsequently reduced with sodium borohydride, for ease of isolation, to give the corresponding alcohol, **3a**, in 59% yield, >99:1 er, and >20:1 dr. Further screening studies determined that palladium(II) acetate was another viable precatalyst for this transformation, furnishing **3a** in 70% yield and 99:1 er.^19^

While two systems that could deliver the desired product in excellent stereoselectivity and comparable yields had been identified, the substrate scope with respect to boronic acid was investigated using the lower catalyst loading of Pd(MeCN)_2_(OTs)_2_ with a practical industrial application in mind. Using this strategy, it proved to be possible to selectively generate both enantiomers of the 2,6-*trans*-THP-alcohol product, **3a** and *ent***-3a**, in comparable yield stereoselectively, by using the correct combination of DHP-alcohol **1** and PyrOx **L1** (Scheme 2).

**Scheme 2:**
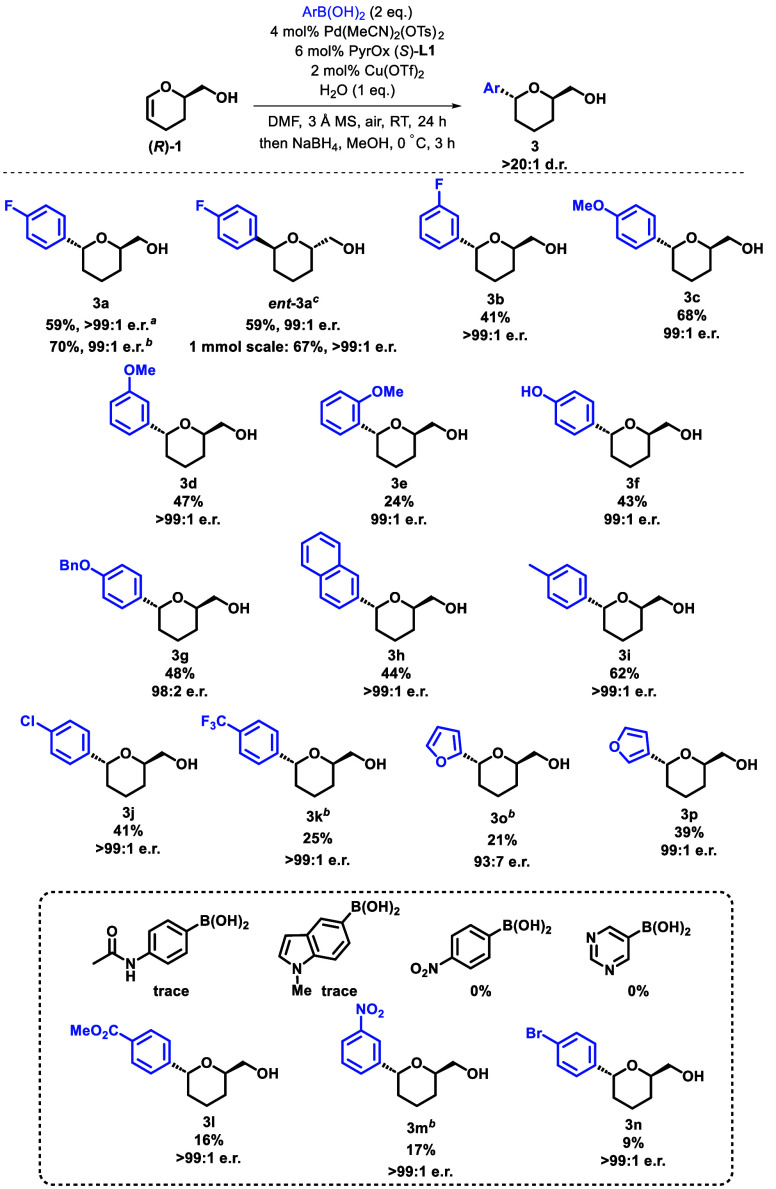
Arylboronic Acid Substrate Scope Average of two runs. Conditions: 2 equiv of *p*-FC_6_H_4_B(OH)_2_, 10 mol % Pd(OAc)_2_, 15 mol % PyrOx **L1**, 4 mol % Cu(OTf)_2_, 1 equiv of water, DMF (0.1 M), 3 Å MS, air, room temperature, 24 h; then NaBH_4_, MeOH, 0 °C, 3 h. Starting from (*S*)-DHP-**1** using PyrOx (*R*)-**L1**.

An X-ray crystal structure of ferrocenoyl-functionalized **3a** confirmed the absolute stereochemistry.^19^ The use of other fluorophenylboronic acid isomers proved to be less successful under the optimized reaction conditions.^19^ All substrates maintained excellent stereoselectivites throughout. Methoxy-, hydroxy-, benzyloxy-, naphthyl-, and alkyl-substituted boronic acids proved to be successful (**3c**-**i**). In addition to fluorine, *p*-chlorophenylboronic acid was well tolerated (**3j**); however, yields with *p*-bromo phenylboronic acid (**3n**) were significantly reduced, likely due to the propensity of the bromine to undergo reactions with palladium.

Use of *p*-(trifluoromethyl)phenylboronic acid initially led to the formation of a trace of the aldehyde product. However, when the alternative palladium(II) acetate conditions with higher catalyst loading were employed, an increase in product formation was observed (**3k**). This strategy was also applied to improve the yield of the more electron-deficient systems (**3k**, **3m**, and **3o**); however, *p*-nitrophenyl- and pyrimidylboronic acids were unreactive under these conditions. Heteroaromatic boronic acids were tolerated (**3o** and **3p**), although the use of 2-furanylboronic acid resulted in a slight erosion of enantioselectivity (**3o**).

Finally, we sought to demonstrate our developed methodology in the synthesis of centrolobine, a natural product that has been found to exhibit antibacterial and antifungal properties.^22^ Both *cis*-enantiomers of centrolobine have been isolated, and a number of total syntheses of these naturally occurring stereoisomers have been reported.^23^ Given these efforts, interest has shifted toward the unnatural diastereomers,^24^ which could be used to develop structure–activity relationships of these cores. More specifically, given that Colobert’s synthesis of the *cis*-isomer of centrolobine utilized a *cis*-isomer of **2c** as a key intermediate,^23a,23b^ we proposed that a *trans*-isomer of centrolobine could be accessed in short order using our developed methodology to more rapidly access this key 2,6-disubstituted THP motif.

To this end, application of our Heck redox-relay conditions to (*S*)-DHP-alcohol **1** and 4-(methoxy)phenylboronic acid gave *ent*-**2c** in 60% yield (Scheme 3). Then, following the approach of Colobert,^23a,23b^ Wittig reaction of *ent*-**2c** using phosphonium salt **4** afforded alkene **5** in 72% yield. Exposure of **5** to 3.5 mol % Pd/Al_2_O_3_ in the presence of H_2_ resulted in concomitant reduction of the alkene and benzyl deprotection, to afford the (3*S*,7*S*)-*trans*-isomer of centrolobine **6** in 68% yield. Given the literature precedent for the ready epimerization of the C-aryl glycoside bond from *trans* to *cis* in intermediates of type **5**,^24d^ this approach could be used to rapidly access all four stereoisomers of centrolobine.

**Scheme 3:**
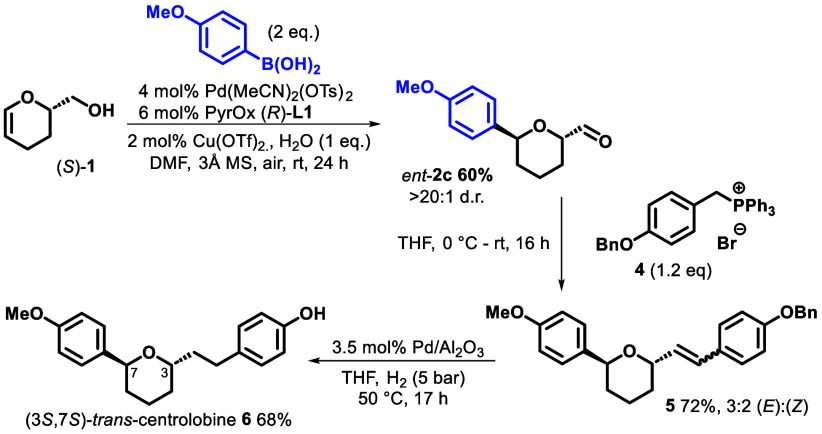
Total Synthesis of *trans*-Centrolobine

In summary, we have applied an oxidative Heck redox-relay strategy to the synthesis of C-aryl-containing 2,6-*trans*-tetrahydropyrans, from enantiopure dihydropyranyl alcohols. Using (*R*)- or (*S*)-DHP-alcohol **1**, a range of 2,6-*trans*-tetrahydropyrans, bearing diverse functionality, were generated under mild conditions in excellent stereoselectivity. This motif also provides a synthetic handle for further functionalization, enabling facile access to a diverse set of substrates from a simple building block. We also demonstrated the utility of this approach via the concise synthesis of a *trans*-isomer of the natural product centrolobine.

This approach represents a valuable addition to the redox-relay oxidative Heck toolkit, and the novel *exo*-cyclic migration underpinning this sequence opens up the potential for similar redox-relay chemistry on broader heterocyclic systems.

## Data Availability

The data underlying this study are available in the published article and its Supporting Information.

## Supporting Information Available

Experimental procedures, compound characterization data, DFT calculations and coordinates, and X-ray crystal structure data (PDF)
