# Supplementary material for: Synthesis of 2,6-trans-Tetrahydropyrans Using a Palladium-Catalyzed Oxidative Heck Redox-Relay Strategy
Source: Org Lett. 2024 Jan 10;26(14):2857–61. doi: 10.1021/acs.orglett.3c03866 (PMC11020158; doi:10.1021/acs.orglett.3c03866)
Supplement: Supplementary file 1 — ol3c03866_si_001.pdf [file ol3c03866_si_001.pdf]

Supporting Information for

The Synthesis of 2,6-*trans*-Tetrahydropyrans using a Palladium-Catalyzed Oxidative Heck Redox-Relay Strategy

Holly E. Bonfield,[a,b†] Colin M. Edge,[b] Marc Reid,[a] Alan R. Kennedy,[a] David D. Pascoe,*[b] David M. Lindsay,*[a] and Damien Valette*[b‡]

[a]Department of Pure and Applied Chemistry,
University of Strathclyde
Thomas Graham Building, 295 Cathedral Street, Glasgow G1 1XL, U.K.
E-mail: david.lindsay@strath.ac.uk

[b]Drug Substance Development, GSK
Gunnels Wood Road, Stevenage, Hertfordshire SG1 2NY, U.K.
E-mail: david.d.pascoe@gsk.com

†current address: Apex Molecular Ltd, Alderley Park, Alderley Edge SK10 4TG, U.K.

‡current address: MSD R&D Innovation Centre, 120 Moorgate, London EC2M 6UR, U.K.
E-mail: damien.x.valette@gmail.com

Supplementary Material

Table of Contents

1. General Information 6

Materials 6

Nuclear Magnetic Resonance (NMR) 6

High performance liquid chromatography (HPLC) 6

Low resolution liquid chromatography mass spectrometry (LC-MS) 6

High Resolution Mass Spectrometry (HRMS) 7

Infrared spectroscopy (IR) 8

Melting point 8

Purification by automated column chromatography 8

GC-MS 8

Polarimetry 8

Determination of enantiomeric ratio 8

Activation of Molecular Sieves 9

2. General Procedures 10

2.1 General Procedure A - Synthesis of PyrOx ligand series 10

2.2 General Procedure B - Ligand screen (see Section 3.4) 10

2.3 General Procedure C - Redox-relay oxidative Heck time courses with (*R*)-(3,4-dihydro-2*H*-pyran-2-yl)methanol 11

2.4 General Procedure D – Substrate scope redox-relay oxidative Heck and reduction with (*R*)-(3,4-dihydro-2*H*-pyran-2-yl)methanol: 12

3. Experimental Procedures 13

3.1 PyrOx Ligand Synthesis 13

Synthesis of 2-(5-(trifluoromethyl)pyridin-2-yl)-4,5-dihydrooxazole, PyrOx **L0** 13

Synthesis of (*S*)-*N*-(1-hydroxy-3,3-dimethylbutan-2-yl)-5-(trifluoromethyl)picolinamide, *int*-PyrOx (*S*)-**L1** 14

Synthesis of (*S*)-4-(*tert*-butyl)-2-(5-(trifluoromethyl)pyridin-2-yl)-4,5-dihydrooxazole, PyrOx (*S*)-**L1** 15

Synthesis of (*R*)-4-(*tert*-butyl)-2-(5-(trifluoromethyl)pyridin-2-yl)-4,5-dihydrooxazole, PyrOx (*R*)-**L1** 16

Synthesis of (*S*)-4-(*tert*-butyl)-2-(5-nitropyridin-2-yl)-4,5-dihydrooxazole, PyrOx (*S*)-**L2** 17

Synthesis of (*S*)-*N*-(1-hydroxy-3,3-dimethylbutan-2-yl)-5-nitropicolinamide, *int*-PyrOx (*S*)-**L3** 17

Synthesis of (*S*)-4-(*tert*-butyl)-2-(5-methoxypyridin-2-yl)-4,5-dihydrooxazole, PyrOx **L3** 18

Synthesis of (*S*)-4-phenyl-2-(5-(trifluoromethyl)pyridin-2-yl)-4,5-dihydrooxazole, PyrOx (*S*)-**L4** .19

Synthesis of (*S*)-2-(5-nitropyridin-2-yl)-4-phenyl-4,5-dihydrooxazole, PyrOx (*S*)-**L5** 19

3.2 Separation of (3,4-dihydro-2*H*-pyran-2-yl)methanol enantiomers 21
Benzoate Derivative - Preparative Chiral Purification 21
Synthesis of (3,4-dihydro-2*H*-pyran-2-yl)methyl benzoate 21
Synthesis of (*R*)-(3,4-dihydro-2*H*-pyran-2-yl)methanol, (*R*)-**1** 25
Synthesis of (*S*)-(3,4-dihydro-2*H*-pyran-2-yl)methanol, (*S*)-**1** 25
Acetate Derivative – Enzymatic Resolution 26
Synthesis of (3,4-dihydro-2*H*-pyran-2-yl)methyl acetate 26
Synthesis of (*R*)-(3,4-dihydro-2*H*-pyran-2-yl)methyl acetate 26
Deprotection of enantiomerically pure (3,4-dihydro-2*H*-pyran-2-yl)methyl acetate..28
Synthesis of (*R*)-(3,4-dihydro-2*H*-pyran-2-yl)methanol, (*R*)-**1** 28
3.3 Proof of Concept Reaction (Manuscript Scheme 1)..29
3.4 Ligand Screen..30
3.5 Match/Mis-match Effects (Manuscript Figure 1)..31
3.6 Use of *rac*-(3,4-dihydro-2*H*-pyran-2-yl)methanol **1** in a Kinetic Resolution-Type Process 33
3.7 Reaction Optimisation for Redox Relay Oxidative Heck Reaction of (*R*)-DHP-alcohol, (*R*)-**1** with 4-(Fluorophenyl)boronic acid (Manuscript Table 1)..36
Preliminary screening of reaction conditions..36
Variables:..36
Plate design and Set-up 36
Results Summary 37
Reaction Optimisation with bis(acetonitrile)palladium(II) *p*-toluenesulfonate..39
Reaction Optimisation with palladium(II) acetate..40
Determination of enantioselectivities for reaction optimisation 41
3.8 Substrate Scope (Manuscript Scheme 2)..43
Synthesis of ((2*R*,6*R*)-6-(4-fluorophenyl)tetrahydro-2*H*-pyran-2-yl)methanol, **3a**..43
Synthesis of the ferrocenoyl-ester of **3a**, **Fc-3a**..44
Synthesis of ((2*S*,6*S*)-6-(4-fluorophenyl)tetrahydro-2*H*-pyran-2-yl)methanol, ***ent*-3a**..45
Synthesis of ((2*R*,6*R*)-6-(3-fluorophenyl)tetrahydro-2*H*-pyran-2-yl)methanol, **3b**..47
Synthesis of ((2*R*,6*R*)-6-(4-methoxyphenyl)tetrahydro-2*H*-pyran-2-yl)methanol, **3c**..48
Synthesis of ((2*R*,6*R*)-6-(3-methoxyphenyl)tetrahydro-2*H*-pyran-2-yl)methanol, **3d** 50
Synthesis of ((2*R*,6*R*)-6-(2-methoxyphenyl)tetrahydro-2*H*-pyran-2-yl)methanol, **3e**..52
Synthesis of 4-((2*R*,6*R*)-6-(hydroxymethyl)tetrahydro-2*H*-pyran-2-yl)phenol, **3f** 53
Synthesis of ((2R,6R)-6-(4-(benzyloxy)phenyl)tetrahydro-2*H*-pyran-2-yl)methanol, **3g** 55
Synthesis of ((2R,6R)-6-(naphthalen-2-yl)tetrahydro-2*H*-pyran-2-yl)methanol, **3h** 56
Synthesis of ((2*R*,6*R*)-6-(*p*-tolyl)tetrahydro-2*H*-pyran-2-yl)methanol, **3i**..58
Synthesis of ((2*R*,6*R*)-6-(4-chlorophenyl)tetrahydro-2*H*-pyran-2-yl)methanol, **3j** 59

Synthesis of ((2*R*,6*R*)-6-(4-(trifluoromethyl)phenyl)tetrahydro-2*H*-pyran-2-yl)methanol, **3k**......61
Synthesis of methyl 4-((2*R*,6*R*)-6-(hydroxymethyl)tetrahydro-2*H*-pyran-2-yl)benzoate, **3l**.........63
Synthesis of ((2*R*,6*R*)-6-(3-nitrophenyl)tetrahydro-2*H*-pyran-2-yl)methanol, **3m**........................64
Synthesis of ((2*R*,6*R*)-6-(4-bromophenyl)tetrahydro-2*H*-pyran-2-yl)methanol, **3n**......................66
Synthesis of ((2*R*,6*R*)-6-(furan-2-yl)tetrahydro-2*H*-pyran-2-yl)methanol, **3o**..............................68
Synthesis of ((2*R*,6*R*)-6-(furan-3-yl)tetrahydro-2*H*-pyran-2-yl)methanol, **3p**..............................70
3.9 Synthesis of a *trans*- Stereoisomer of Centrolobine (Manuscript Scheme 3).......................72
Synthesis of (2*S*,6*R*)-6-(4-methoxyphenyl)tetrahydro-2*H*-pyran-2-carbaldehyde, *ent*-**2c**............72
Synthesis of (4-(benzyloxy)benzyl)triphenylphosphoniumbromide **4**..73
Synthesis of (2*S*,6*S*)-2-(4-(benzyloxy)styryl)-6-(4-methoxyphenyl)tetrahydro-2*H*-pyran **5**.........74
Synthesis of (3*S*,7*S*)-Centrolobine **6**...75
4. NMR Spectra...76
(*S*)-*N*-(1-hydroxy-3,3-dimethylbutan-2-yl)-5-(trifluoromethyl)picolinamide, *int*-PyrOx **L1**..........76
(*S*)-4-(*tert*-butyl)-2-(5-(trifluoromethyl)pyridin-2-yl)-4,5-dihydrooxazole, PyrOx **L1**....................79
(*S*)-4-(*tert*-butyl)-2-(5-nitropyridin-2-yl)-4,5-dihydrooxazole, PyrOx **L2**......................................82
(S)-N-(1-hydroxy-3,3-dimethylbutan-2-yl)-5-nitropicolinamide, *int*-PyrOx **L3**............................84
(*S*)-4-(*tert*-butyl)-2-(5-methoxypyridin-2-yl)-4,5-dihydrooxazole, PyrOx **L3**................................86
(*S*)-4-phenyl-2-(5-(trifluoromethyl)pyridin-2-yl)-4,5-dihydrooxazole, PyrOx **L4**..........................88
(*S*)-2-(5-nitropyridin-2-yl)-4-phenyl-4,5-dihydrooxazole, PyrOx **L5**...91
(3,4-dihydro-2*H*-pyran-2-yl)methyl benzoate 97
(3,4-dihydro-2*H*-pyran-2-yl)methanol, (*R*)-**1** 99
(3,4-dihydro-2*H*-pyran-2-yl)methyl acetate 101
((2*R*,6*R*)-6-(4-fluorophenyl)tetrahydro-2*H*-pyran-2-yl)methanol, **3a** 103
Ferrocenoyl derivative **Fc**-**3a**: 110
((2*R*,6*R*)-6-(3-fluorophenyl)tetrahydro-2*H*-pyran-2-yl)methanol, **3b**...117
((2*R*,6*R*)-6-(4-methoxyphenyl)tetrahydro-2*H*-pyran-2-yl)methanol, **3c**....................................124
((2*R*,6*R*)-6-(3-methoxyphenyl)tetrahydro-2*H*-pyran-2-yl)methanol, **3d**...................................130
((2*R*,6*R*)-6-(2-methoxyphenyl)tetrahydro-2*H*-pyran-2-yl)methanol, **3e**...................................136
4-((2*R*,6*R*)-6-(hydroxymethyl)tetrahydro-2*H*-pyran-2-yl)phenol, **3f**..142
((2*R*,6*R*)-6-(*p*-benzyloxy)tetrahydro-2*H*-pyran-2-yl)methanol, **3g**...148
((2R,6R)-6-(naphthalen-2-yl)tetrahydro-2*H*-pyran-2-yl)methanol, **3h** 154
((2*R*,6*R*)-6-(*p*-tolyl)tetrahydro-2*H*-pyran-2-yl)methanol, **3i**...160
((2*R*,6*R*)-6-(4-chlorophenyl)tetrahydro-2*H*-pyran-2-yl)methanol, **3j**:...166
((2*R*,6*R*)-6-(4-(trifluoromethyl)phenyl)tetrahydro-2*H*-pyran-2-yl)methanol, **3k**:.......................172
methyl 4-((2*R*,6*R*)-6-(hydroxymethyl)tetrahydro-2*H*-pyran-2-yl)benzoate, **3l**:..........................179
((2*R*,6*R*)-6-(3-nitrophenyl)tetrahydro-2*H*-pyran-2-yl)methanol, **3m**:..185

((2*R*,6*R*)-6-(4-bromophenyl)tetrahydro-2*H*-pyran-2-yl)methanol, **3n** 191
((2*R*,6*R*)-6-(furan-2-yl)tetrahydro-2*H*-pyran-2-yl)methanol, **3o** 197
((2*R*,6*R*)-6-(furan-3-yl)tetrahydro-2*H*-pyran-2-yl)methanol, **3p** 203
(2*S*,6*S*)-6-(4-methoxyphenyl)tetrahydro-2*H*-pyran-2-carbaldehyde, *ent*-**2c** 209
(4-(benzyloxy)benzyl)triphenylphosphonium bromide **4** 210
(2*S*,6*S*)-2-(4-(benzyloxy)styryl)-6-(4-methoxyphenyl)tetrahydro-2*H*-pyran **5** 211
(3*S*,7*S*)-Centrolobine **6** 212
Appendix 1. Computational Studies 213
Protocol for calculations 213
Computational study of the effect of the PyrOx ligand 214
Representative geometries 215
Appendix 2. X-ray Data 242
References 256

1. General Information

Materials

Reagents were obtained from commercial sources and used as received. Solvents were used wet unless otherwise stated.

Nuclear Magnetic Resonance (NMR)

NMR spectra were recorded using a Bruker AV400 instrument, and processed using ACD/SpecManager v12.5. Chemical shifts (δ) are reported in parts per million (ppm) relative to tetramethylsilane (TMS), and coupling constants (*J*) are reported in Hz. Coupling constants (*J*) refer to a $^3J_{H\text{-}H}$ coupling, unless otherwise stated. The following abbreviations are used for multiplicities: s = singlet; br s = broad singlet; d = doublet; t = triplet; q = quartet; quin = quintet; spt = septet; m = multiplet; dd = doublet of doublets; and td = triplet of doublets. NMR experiments were run at 30 °C.

High performance liquid chromatography (HPLC)

These data were recorded on an Agilent HPLC system, equipped with a Waters CSH column (30 mm length × 2.0 mm internal diameter, 8.5 µm packing particle size) at 40 °C.

The solvents employed were:

A = water + 0.05% v/v trifluoroacetic acid.

B = MeCN + 0.05% v/v trifluoroacetic acid.

The gradient employed was as follows:

**Time / min**	**Flow rate / mL min^{-1}**	**% A**	**% B**
0	1	97	3
3.70	1	5	95
4.00	1	5	95
4.10	1	97	3

The UV response was monitored at a wavelength of 220 nm.

Low resolution liquid chromatography mass spectrometry (LC-MS)

2 minute high pH method

These data were recorded using a Waters Acquity UPLC, equipped with a CSH C18 column (50 mm × 2.1 mm internal diameter, 1.7 µm packing diameter) at 40 °C.

The solvents employed were:

A = 10 mM ammonium bicarbonate in water, adjusted to pH 10 with ammonia solution.

B = MeCN

The gradient employed was as follows:

Time / min	Flow rate / mL min^{-1}	% A	% B
0	1	97	3
0.05	1	97	3
1.5	1	5	95
1.9	1	5	95
2.0	1	97	3

UV detection was an averaged signal from wavelength of 210 nm to 350 nm and mass spectra were recorded on a Waters ZQ mass spectrometer using alternate-scan positive and negative electrospray ionization (ES).

High Resolution Mass Spectrometry (HRMS)

The UPLC analysis was conducted on an Acquity UPLC CSH C18 column (100 mm x 2.1 mm i.d. 1.7 µm packing diameter) at 50 °C.

The solvents employed were:

A = 0.1% v/v solution of formic acid in water.

B = 0.1% v/v solution of formic acid in MeCN.

The gradient employed was:

Time (min)	Flow Rate (ml/min)	% A	% B
0	0.8	95	5
8.5	0.8	7	93
9.0	0.8	7	93
9.5	0.8	95	5
10	0.8	95	5

The UV detection was a summed signal from wavelength of 210 nm to 500 nm.

Injection volume : 0.2 µl.

MS Conditions

MS : Waters XEVO G2-XS Qtof

Ionisation mode : Positive Electrospray

Scan Range : 100 to 1200 AMU

Infrared spectroscopy (IR)

IR spectra were recorded using a Perkin Elmer Spectrum One FT-IR spectrometer fitted with a Perkin Elmer Universal ATR (attenuated total reflectance) sampling accessory. Absorption frequencies are reported in wavenumbers (cm^{-1}); only selected absorbances are reported.

Melting point

Melting points were measured on a Büchi Melting Point M-565 and are reported in °C.

Purification by automated column chromatography

Normal phase silica chromatography was performed on a Biotage® SP4, using Biotage® SNAP ultra or Biotage® Sfär prepacked cartridges.

UV response was monitored as an average of wavelengths between 200 and 400 nm.

Reverse phase chromatography was performed on a CombiFlash EZ Prep system, using a HpH column (Xselect® CSH™ Prep C18 5 µm OBD™ 30 x 100 mm)

GC-MS

These data were recorded using an Agilent Technologies Intuvo 9000 GC system, equipped with a HP5 MS UltraInert (30 mm × 0.25 mm internal diameter, 0.25 µm packing diameter) and MSD detector. An injection volume of 0.5 mcL was used with a 50:1 split ratio and a 1.0 mL min^{-1} (He) flow rate. A 40°C inlet temperature was increased at a rate of 25 °C min^{-1} to a final temperature of 300 °C.

Polarimetry

Optical rotations were obtained using a Jasco P-1030 polarimeter fitted with a sodium (589 nm) lamp, a Glan-Taylor polarising prism and a photomultiplier tube detector. For all measurements a path length of 10 cm was used.

Determination of enantiomeric ratio

For determination of the enantiomeric ratio of compounds **3a** to **3r**, samples of the opposite enantiomers were also synthesised from (*S*)-DHP-**1** using PyrOx (*R*)-**L1**. Spiking of each sample with *ent*-**3** (**a**-**r**) was used to confirm the retention time of the minor enantiomer.

Activation of Molecular Sieves

The molecular sieves were activated by placing under vacuum and heating with a heat gun for a few minutes, before being allowed to cool to RT under nitrogen.

2. General Procedures

2.1 General Procedure A - Synthesis of PyrOx ligand series

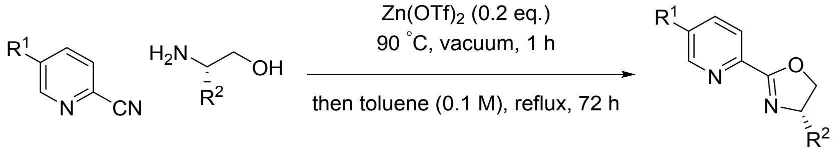

To a 50 mL 3-neck RBF was charged zinc trifluoromethanesulfonate (0.2 eq.) and a stir bar. This was heated at 90 °C under vacuum for 1 h. After this time the system was cooled to RT under nitrogen. A solution of amino alcohol (1.0 eq.) and picolinonitrile (250 mg, 1.0 eq.) in anhydrous toluene (10 mL) was then charged into the vessel and heated at reflux under nitrogen for 72 h. After this time, the reaction was allowed to cool to RT. Ethyl acetate (20 mL) and saturated aqueous sodium bicarbonate (20 mL) were added, the layers separated and the aqueous phase back-extracted with ethyl acetate (2 × 15 mL). The combined organics were then dried and the solvent removed *in vacuo*. Purification by automated column chromatography (0-100% ethyl acetate in heptane) afforded the corresponding product.

2.2 General Procedure B - Ligand screen (see Section 3.4)

Note: the redox-relay oxidative Heck reaction is thought to be mixing limited and so a high stir speed (≥800 rpm) is recommended.

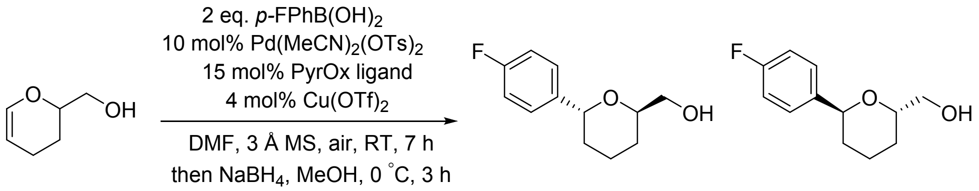

To a glass vial was added bis(acetonitrile)palladium(II) *p*-toluenesulfonate (46.5 mg, 10 mol%), PyrOx ligand (15 mol%), difluoromethylbenzene (20.0 mg), copper(II) trifluoromethanesulfonate (12.7 mg, 4 mol%), 6 × 3 Å molecular sieve beads (~250 mg) and anhydrous DMF (4.4 mL). The resulting mixture was stirred (800 rpm) open to air at RT for 15 min. A solution of (3,4-dihydro-2*H*-pyran-2-yl)methanol (100 mg, 0.88 mmol, 1.00 eq.) and (*p*-fluorophenyl)boronic acid (245 mg, 1.75 mmol, 2.0 eq.) in anhydrous DMF (4.4 mL) was then added in a single portion. The resulting mixture was then stirred

(800 rpm) open to air at RT for 7 h. After this time, ethyl acetate (8 mL) and saturated aqueous brine (4 mL) were added, the layers separated and the aqueous phase back extracted with ethyl acetate (3 × 4 mL). The combined organic phases were dried (phase separator) and concentrated *in vacuo*. The crude residue was then purified by automated column chromatography (0-100% ethyl acetate in heptane) to afford (2*R*,6*R*)-6-(4-fluorophenyl)tetrahydro-2*H*-pyran-2-carbaldehyde.

To a solution of (2*R*,6*R*)-6-(4-fluorophenyl)tetrahydro-2*H*-pyran-2-carbaldehyde in MeOH (3 mL) at 0 °C under nitrogen was added sodium borohydride (10 eq.). The resultant solution was left stirring at 0 °C under nitrogen for 3 h. After this time, the reaction was quenched by addition of 5% water in ethanol (5 mL). The resultant solution was left stirring for 10 min. Water (2 mL) was then slowly added. The resultant solution was left stirring for 20 min. Ethyl acetate (10 mL) and saturated aqueous brine (2 mL) were added, the layers separated and the aqueous phase back-extracted with ethyl acetate (3 × 2 mL). The combined organics were filtered, dried (phase separator) and the solvent removed *in vacuo*. Purification by automated column chromatography (0-100% ethyl acetate in heptane) afforded ((2*R*,6*R*)-6-(4-fluorophenyl)tetrahydro-2*H*-pyran-2-yl)methanol as a colourless oil.

2.3 General Procedure C - Redox-relay oxidative Heck time courses with (*R*)-(3,4-dihydro-2*H*-pyran-2-yl)methanol

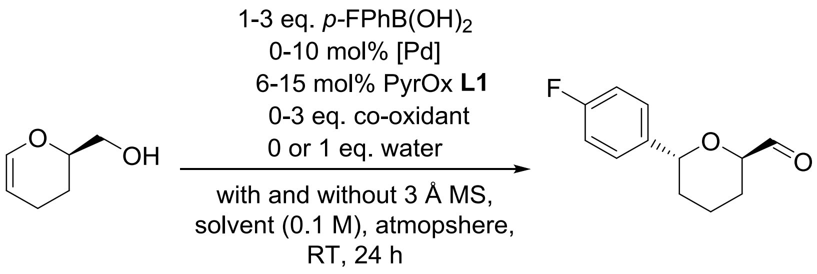

To a glass vial was added palladium source (0-10 mol%), PyrOx **L1** (6-15 mol%), co-oxidant (0-3.0 eq.), difluoromethylbenzene (4.0 mg), molecular sieve beads (0-~125 mg) and solvent (0.9 mL, 0.2 M). The resulting mixture was stirred (800 rpm) for 15 min. A solution of (3,4-dihydro-2*H*-pyran-2-yl)methanol (20.0 mg, 0.175 mmol, 1.0 eq.) and (*p*-fluorophenyl)boronic acid (1-3 eq.) in solvent (0.9 mL, 0.2 M) was then added in a single portion. Water (0-1.0 eq.) was then quickly added. The resulting mixture was then stirred (800 rpm) for 24 h. Analytical samples were prepared by taking a 20 µL aliquot of the reaction mixture and diluting it in $CDCl_3$ (600 µL) at each time point. Solution yields were determined by comparing the integration of the internal standard (difluoromethylbenzene, δ -110.66 ppm) and product (δ -115.01 ppm) by $^{19}F\{^1H\}$ NMR.

2.4 General Procedure D – Substrate scope redox-relay oxidative Heck and reduction with (*R*)-(3,4-dihydro-2*H*-pyran-2-yl)methanol:

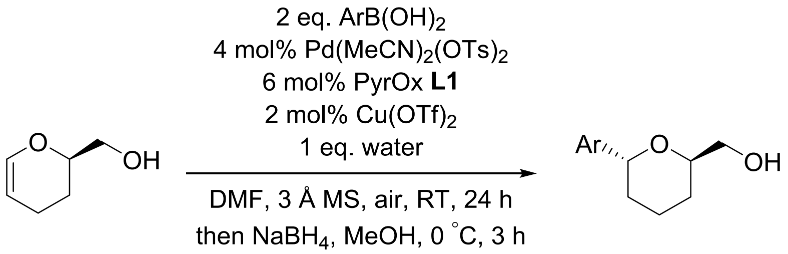

To a glass vial was added bis(acetonitrile)palladium(II) *p*-toluenesulfonate (9.3 mg, 4 mol%), PyrOx **L1** (7.2 mg, 6 mol%), copper(II) trifluoromethanesulfonate (3.2 mg, 2 mol%), 6 × 3 Å molecular sieve beads (~250 mg) and anhydrous DMF (2.2 mL, 0.2 M). The resulting mixture was stirred (800 rpm) open to air at RT for 15 min. A solution of (*R*)-(3,4-dihydro-2*H*-pyran-2-yl)methanol (50.0 mg, 0.438 mmol, 1.0 eq.) and boronic acid (2.0 eq.) in anhydrous DMF (2.2 mL, 0.2 M) was then added in a single portion. Water (1.0 eq.) was then quickly added. The resulting mixture was then stirred (800 rpm) open to air at RT for 24 h. After this time, the reaction mixture was diluted with ethyl acetate (10 mL), quenched with 5% aqueous lithium chloride (5 mL), the layers separated and the organic phase washed with 5% aqueous lithium chloride (4 × 5 mL). The combined aqueous phases were back-extracted with ethyl acetate (15 mL), the combined organics dried (phase separator) and the solvent removed *in vacuo*. The crude material was dissolved in MeOH (3.5 mL), placed under an atmosphere of nitrogen and cooled to 0 °C. Sodium borohydride (166 mg, 10.0 eq.) was subsequently added and the resultant solution stirred at 0 °C for 3 h. After this time, ethyl acetate (4 mL) and saturated aqueous brine (4 mL) were added and the layers separated. The aqueous phase was extracted with ethyl acetate (3 × 4 mL), the combined organics dried (phase separator) and the solvent removed *in vacuo*. Purification by automated column chromatography afforded the desired product.

3. Experimental Procedures

3.1 PyrOx Ligand Synthesis

Synthesis of 2-(5-(trifluoromethyl)pyridin-2-yl)-4,5-dihydrooxazole, PyrOx **L0**

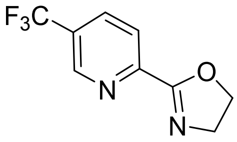

PyrOx **L0**
$C_9H_7F_3N_2O$
MW 216.16

To a solution of 5-(trifluoromethyl)picolinonitrile (1.00 g, 5.81 mmol, 1.0 eq.) in anhydrous methanol (15 mL) under nitrogen was added sodium methoxide (94.0 mg, 1.74 mmol, 0.3 eq.). The resultant solution was then stirred at 40 °C for 16 h. After this time, the reaction was allowed to cool to RT. The reaction mixture was partitioned in 1:1 EtOAc (25 mL): water (25 mL) and the layers separated. The organic phase was washed with saturated aqueous brine (30 mL), dried (phase separator) and the solvent removed in vacuo. Toluene (15 mL) was added, and the solution placed under an atmosphere of nitrogen at RT. 2-aminoethan-1-ol (0.42 mL, 6.97 mmol, 1.2 eq.) and *p*-toluenesulfonic acid (111 mg, 0.58 mmol, 0.2 eq.) were then charged into the vessel and heated at 80 °C under nitrogen for 16 h. After this time, the reaction was allowed to cool to RT. The reaction was quenched with water (20 mL) and the layers separated. The organic phase was extracted with EtOAc (2 × 20 mL). The combined organic phases were washed with saturated aqueous brine (20 mL), dried (phase separator) and the solvent removed in vacuo. Purification by automated column chromatography (0-100% ethyl acetate in heptane) afforded PyrOx **L0** as a white solid (980 mg, 78%). ^1H NMR (400 MHz, $CDCl_3$, 25 °C, TMS) δ = 8.96 (d, ^4J = 1.8 Hz, 1H), 8.18 (d, J = 8.3 Hz, 1H), 8.03 (dd, J = 8.3, ^4J = 1.8 Hz, 1H), 4.57 (t, J = 10.3 Hz, 2H), 4.17 (t, J = 10.3 Hz, 2H) ppm; ^{13}C NMR (101 MHz, $CDCl_3$, 25 °C, TMS) δ = 162.9, 149.9, 146.6 (q, $^3J_{C-F}$ = 4.6 Hz), 134.0 (q, $^3J_{C-F}$ = 3.1 Hz), 128.1 (q, $^2J_{C-F}$ = 33.6 Hz), 123.6, 123.1 (q, $^1J_{C-F}$ = 270.0 Hz), 68.6, 55.3 ppm; $^{19}F\{1H\}$ NMR (376 MHz, $CDCl_3$) δ = -62.6 (s, 3F) ppm; ν_{max} / cm^{-1} 2938, 1655, 1644, 1603, 1573, 1323, 1121, 1089, 1071, 671; mp 118-120 °C; HRMS (ESI) m/z calcd for $[M+H]^+$ $C_9H_8F_3N_2O$: 217.0589; found: 217.0596. All spectroscopic data was in agreement with the literature.[1]

Synthesis of (*S*)-*N*-(1-hydroxy-3,3-dimethylbutan-2-yl)-5-(trifluoromethyl)picolinamide, *int*-PyrOx (*S*)-**L1**

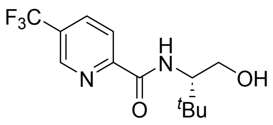

int-PyrOx (*S*)-**L1**

$C_{13}H_{17}F_3N_2O_2$
MW 290.286

N-methylmorpholine (1.04 mL, 9.42 mmol) was added to a stirred solution of 5-(trifluoromethyl)picolinic acid (1.20 g, 6.28 mmol) in anhydrous DCM (37 mL, 0.17 M) under nitrogen. The reaction mixture was cooled to 0 °C and butylchloroformate (0.86 mL, 6.59 mmol) was added dropwise over 5 min. The reaction mixture was then stirred at 0 °C for 30 min. A separate flask was charged with (*S*)-2-amino-3,3-dimethylbutan-1-ol (0.81 g, 6.91 mmol), anhydrous DCM (5 mL) and *N*-methylmorpholine (0.76 mL, 6.91 mmol) with stirring under nitrogen. The resultant solution was then added dropwise at 0 °C over 10 min and then stirred at RT for a further 6 h. The reaction was quenched by addition of saturated aqueous ammonium chloride (10 mL) and diluted with the addition of water (10 mL). The layers were separated and the aqueous phase was extracted with DCM (3 × 10 mL). The organic phase was subsequently washed with saturated aqueous sodium bicarbonate (10 mL) and saturated aqueous brine (10 mL), dried ($MgSO_4$), filtered, the solvent removed *in vacuo* and placed under high vacuum at 40 °C for 12 h. The resultant solid was purified by automated column chromatography (0-100% ethyl acetate in heptane) to afford *int*-PyrOx (*S*)-**L1** as a white amorphous solid (1.07 g, 59%). ^{1}H NMR (400 MHz, $CDCl_3$, 30 °C, TMS) δ = 8.89 - 8.86 (m, 1H), 8.37 (d, *J* = 8.2 Hz, 1H), 8.32 - 8.25 (m, 1H), 8.14 (dd, *J* = 8.2, ^4J = 2.0 Hz, 1H), 4.05 - 3.99 (m, 2H), 3.73 - 3.70 (m, 1H), 2.29 (t, *J* = 5.3 Hz, 1H), 1.08 (s, 9H); ^{13}C NMR (101 MHz, $CDCl_3$, 27 °C, TMS) δ = 164.0, 152.6, 145.3 (q, $^3J_{C-F}$ = 4.1 Hz), 134.9 (q, $^3J_{C-F}$ = 3.1 Hz), 128.9 (q, $^2J_{C-F}$ = 33.6 Hz), 123.1 (q, $^1J_{C-F}$ = 273.1 Hz), 122.3, 63.4, 60.5, 33.8, 27.0 (3 × C); ^{19}F{^{1}H} NMR (376 MHz, $CDCl_3$) δ = -62.53 (s, 3F); $[\alpha]_D^{22}$ = -60 (*c* 0.1 in $CHCl_3$) [lit. -10 (*c* 0.113 in $CHCl_3$)]; ν_{max} / cm^{-1} 3379, 3220, 2965, 1660, 1574, 1538, 1325, 1165, 1151, 1128, 1117, 1073, 1057, 1019, 702; LC-MS (ES$^+$) 1.01 min (m/z 291.3 found). All spectroscopic data was in agreement with the literature.[2]

Synthesis of (*S*)-4-(*tert*-butyl)-2-(5-(trifluoromethyl)pyridin-2-yl)-4,5-dihydrooxazole, PyrOx (*S*)-**L1**

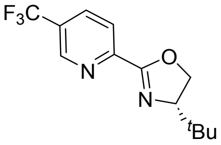

PyrOx (*S*)-**L1**
$C_{13}H_{15}F_3N_2O$
MW 272.271

To a solution of (*S*)-*N*-(1-hydroxy-3,3-dimethylbutan-2-yl)-5-(trifluoromethyl)picolinamide (1.07 g, 3.70 mmol) in anhydrous toluene (15 mL) under nitrogen at 60 °C was added dropwise a solution of thionyl chloride (0.54 mL, 7.40 mmol) in anhydrous toluene (15 mL) over 15 min and then stirred for a further 4 h. The solvent was removed *in vacuo* and the solid dissolved in MeOH (10 mL). Sodium methoxide (0.99 g, 18.49 mmol) was added in one portion. The resultant solution was heated and stirred for 12 h. Toluene (10 mL) was added and the reaction mixture was partially concentrated by rotary evaporation to remove the MeOH. The resultant slurry was washed with water (10 mL), the layers separated and the aqueous layer back-extracted with toluene (3 × 10 mL). The combined organic extracts were dried ($MgSO_4$), filtered and the solvent removed *in vacuo*. Purification by automated column chromatography (0-100% ethyl acetate in heptane) afforded PyrOx (*S*)-**L1** as white needles (670 mg, 67%). ^{1}H NMR (400 MHz, $CDCl_3$, 25 °C, TMS) δ = 8.96 (d, ^{4}J = 1.6 Hz, 1H), 8.22 (d, *J* = 8.3 Hz, 1H), 8.02 (dd, *J* = 8.3, ^{4}J = 1.6 Hz, 1H), 4.49 (dd, *J* = 10.3, ^{2}J = 8.8 Hz, 1H), 4.35 (dd, *J* = 8.8, ^{2}J = 8.8 Hz, 1H), 4.16 (dd, *J* = 8.8, 10.3 Hz, 1H), 0.98 (s, 9H); ^{13}C NMR (101 MHz, $CDCl_3$, 25 °C, TMS) δ = 161.5, 150.1, 146.6 (q, $^{3}J_{C-F}$ = 4.4 Hz), 133.9 (q, $^{3}J_{C-F}$ = 3.4 Hz), 128.0 (q, $^{2}J_{C-F}$ = 33.5 Hz), 123.7, 123.2 (q, $^{1}J_{C-F}$ = 272.9 Hz), 77.2, 69.7, 34.0, 26.0 (3 × C); ^{19}F NMR (376 MHz, $CDCl_3$) δ = -62.60 (s, 3F); $[\alpha]_D^{22}$ = -74 (*c* 0.1 in $CHCl_3$) [lit. -73 (*c* 0.1 in $CHCl_3$)]; ν_{max} / cm^{-1} 2961, 1645, 1605, 1327, 1126, 1101, 1014; LC-MS (ES^+) 1.15 min (m/z 273.3 found); mp 109-110 °C. All spectroscopic data was in agreement with the literature.[2]

Synthesis of (*R*)-4-(*tert*-butyl)-2-(5-(trifluoromethyl)pyridin-2-yl)-4,5-dihydrooxazole, PyrOx (*R*)-**L1**

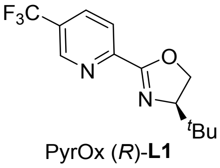

PyrOx (*R*)-**L1**

$C_{13}H_{15}F_3N_2O$

MW 272.271

Using the same two-step procedure as detailed above for PyrOx (*S*)-**L1** afforded PyrOx (*R*)-**L1** as a white solid (65% overall yield over the two steps). Characterisation is the same as for the other enantiomer, except $[\alpha]_D^{22}$ = +77 (*c* 0.1 in $CHCl_3$). All spectroscopic data was in agreement with the literature.[2]

Enantiomeric ratio by chiral HPLC (Chiralpak IF, 5 µm, 4.6 mm × 250 mm; 80% heptane/20% EtOH (+0.2% *iso*-propylamine), 1.0 mL min^{-1}, 250 nm; t_r (PyrOx (*S*)-**L1**) = 5.9 min, t_r (PyrOx (*R*)-**L1**) = 4.5 min), >99:1 e.r for both enantiomers of PyrOx **L1**.

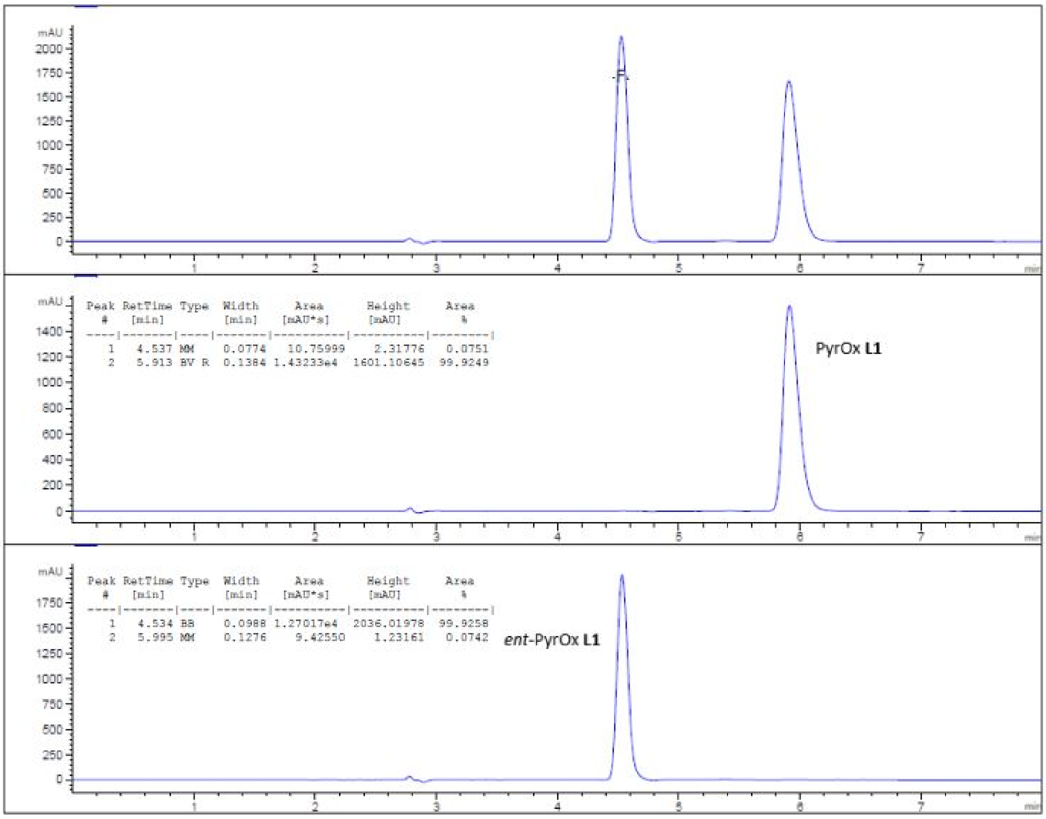

Synthesis of (*S*)-4-(*tert*-butyl)-2-(5-nitropyridin-2-yl)-4,5-dihydrooxazole, PyrOx (*S*)-L2

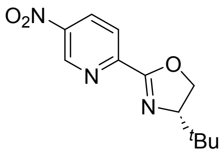

PyrOx (*S*)-**L2**
$C_{12}H_{15}N_3O_3$
MW 249.270

General procedure A was used with zinc trifluoromethanesulfonate (122 mg), 5-nitropicolinonitrile (250 mg, 1.68 mmol) and (*S*)-2-amino-3,3-dimethylbutan-1-ol (196 mg) to afford PyrOx (*S*)-**L2** as an off-white solid (141 mg, 34%). ^{1}H NMR (400 MHz, $CDCl_3$, 25 °C, TMS) δ = 9.50 (d, ^4J = 2.5 Hz, 1H), 8.56 (dd, *J* = 8.7, ^4J = 2.5 Hz, 1H), 8.31 (d, *J* = 8.7 Hz, 1H), 4.52 (dd, *J* = 10.3, ^2J = 8.7 Hz, 1H), 4.38 (dd, *J* = 8.7, ^2J = 8.7 Hz, 1H), 4.19 (dd, *J* = 10.3, 8.7 Hz, 1H), 1.00 (s, 9H); ^{13}C NMR (101 MHz, $CDCl_3$, 27 °C, TMS) δ = 161.0, 151.6, 145.0, 144.8, 131.8, 124.3, 76.9, 69.9, 34.1, 25.9 (3 × C) ppm; $[\alpha]_D^{25}$ = -98 (*c* 0.2 in $CHCl_3$) [lit. -37 (*c* 0.22 in $CHCl_3$)]; ν_{max} / cm^{-1} 3107, 3058, 2962, 2905, 2870, 1641, 1602, 1575, 1523, 1355, 1339, 1116, 1096, 956, 855, 723; mp 167–169 °C. All spectroscopic data is in agreement with the litrature.[3]

Synthesis of (*S*)-*N*-(1-hydroxy-3,3-dimethylbutan-2-yl)-5-nitropicolinamide, *int*-PyrOx (*S*)-L3

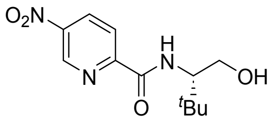

int-PyrOx (*S*)-**L3**
$C_{12}H_{17}N_3O_4$
MW 267.285

N-methylmorpholine (1.47 mL, 13.38 mmol) was added to a stirred solution of 5-(trifluoromethyl)picolinic acid (1.50 g, 8.92 mmol) in anhydrous DCM (53 mL, 0.17 M) under nitrogen. The reaction mixture was cooled to 0 °C and butylchloroformate (1.22 mL, 9.37 mmol) was added dropwise over 5 min. The reaction mixture was then stirred at 0 °C for 30 min. A separate flask was charged with (*S*)-2-amino-3,3-dimethylbutan-1-ol (1.15 g, 9.82 mmol), anhydrous DCM (7 mL) and *N*-methylmorpholine (1.08 mL, 9.82 mmol) with stirring under nitrogen. The resultant solution was then added dropwise at 0 °C over 15 min and then stirred at RT for a further 15 h. The reaction was

quenched by addition of saturated aqueous ammonium chloride (15 mL) and diluted with the addition of water (15 mL). The layers were separated and the aqueous phase was extracted with DCM (3 × 15 mL). The combined organic phases were subsequently washed with saturated aqueous sodium bicarbonate (15 mL) and saturated aqueous brine (15 mL), dried, filtered, the solvent removed *in vacuo* and placed under high vacuum at 40 °C for 12 h. The resultant solid was purified by automated column chromatography (0-100% ethyl acetate in heptane) to afford *int*-PyrOx (*S*)-**L3** as white microcrystals (697 mg, 23%). ^{1}H NMR (400 MHz, $CDCl_3$, 30 °C, TMS): δ = 9.39 (d, ^4J = 2.6 Hz, 1H), 8.65 (dd, J = 8.7, ^4J = 2.6 Hz, 1H), 8.43 (d, J = 8.7 Hz, 1H), 8.21 (br d, J = 8.3 Hz, 1H), 3.96-4.07 (m, 2H), 3.68-3.79 (m, 1H), 2.16 (t, J = 5.4 Hz, 1H), 1.06 ppm (s, 9H); ^{13}C NMR (101 MHz, $CDCl_3$, 30 °C, TMS): δ = 163.1, 154.0, 145.7, 143.8, 132.8, 123.1, 63.2, 60.5, 33.9, 27.0 (3 x C) ppm; $[\alpha]_D^{22}$ = -18 (c 0.2 in $CHCl_3$) [lit. -6 (c 0.2 in $CHCl_3$)]; ν_{max} / cm^{-1} 3392, 3364, 3106, 3091, 2957, 2947, 2869, 1671, 1606, 1580, 1524, 1462, 1356, 1345, 1063, 924, 728, 632; mp 114–119 °C. All spectroscopic data is in agreement with the literature.[3]

Synthesis of (*S*)-4-(*tert*-butyl)-2-(5-methoxypyridin-2-yl)-4,5-dihydrooxazole, PyrOx L3

The methoxy-substituted ligand PyrOx (*S*)-**L3** was prepared via nucleophilic aromatic substitution form the corresponding nitro precursor, *int*-PyrOx (*S*)-**L3**.

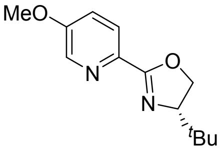

PyrOx **L3**
$C_{13}H_{18}N_2O_2$
MW 234.299

To a solution of (*S*)-*N*-(1-hydroxy-3,3-dimethylbutan-2-yl)-5-nitropicolinamide (200 mg, 0.75 mmol) in anhydrous toluene (4 mL) under nitrogen at 60 °C was added dropwise a solution of thionyl chloride (0.11 mL, 1.50 mmol) in anhydrous toluene (0.2 mL) over 5 min and then stirred for a further 5 h at 60 °C. The solvent was removed *in vacuo* and the residue dissolved in MeOH (3.5 mL). Sodium methoxide (202 mg, 3.74 mmol) was added in one portion. The resultant solution was heated to 55 °C and stirred for 12 h. Toluene (10 mL) was added and the reaction mixture was partially concentrated by rotary evaporation to remove the MeOH. The resultant slurry was washed with water (10 mL), the layers separated and the aqueous layer back-extracted with toluene (3 × 10 mL). The combined organic extracts were dried, filtered and the solvent removed *in vacuo*. Purification by automated

column chromatography (0-100% ethyl acetate in heptane) afforded PyrOx (*S*)-**L3** as an orange solid (90 mg, 51%). ^{1}H NMR (400 MHz, $CDCl_3$, 25 °C, TMS) δ = 8.37 (d, ^4J = 3.0 Hz, 1H), 8.03 (d, *J* = 8.7 Hz, 1H), 7.22 (dd, *J* = 8.7, ^4J = 3.0 Hz, 1H), 4.42 (dd, *J* = 10.1, ^2J = 8.5 Hz, 1H), 4.28 (dd, *J* = 8.5, ^2J = 8.5 Hz, 1H), 4.09 (dd, *J* = 10.1, 8.5 Hz, 1H), 3.90 (s, 3H), 0.97 (s, 9H); ^{13}C NMR (101 MHz, $CDCl_3$, 25 °C, TMS) δ = 162.2, 157.1, 139.4, 137.6, 124.9, 120.2, 76.5, 69.2, 55.7, 34.0, 26.0 (3 × C); $[\alpha]_D^{25}$ = -89 (*c* 1.0 in $CHCl_3$) [lit. +72 (*c* 1.0 in $CHCl_3$)[4]; note, this likely refers to the opposite enantiomer]; ν_{max} / cm^{-1} 3393, 3053, 3021, 2959, 2868, 2843, 1744, 1637, 1591, 1571, 1479, 1368, 1358, 1238, 1131, 1093, 1029, 954, 859, 624; mp 80–84 °C. All spectroscopic data was in agreement with the literature.[4]

Synthesis of (*S*)-4-phenyl-2-(5-(trifluoromethyl)pyridin-2-yl)-4,5-dihydrooxazole, PyrOx (*S*)-**L4**

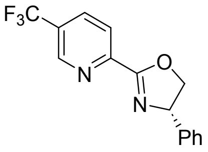

PyrOx (*S*)-**L4**
$C_{15}H_{11}F_3N_2O$
MW 292.261

General procedure A was used with zinc trifluoromethanesulfonate (106 mg), 5-(trifluoromethyl)picolinonitrile (250 mg, 1.45 mmol) and (*S*)-2-amino-2-phenylethan-1-ol (199 mg) to afford PyrOx (*S*)-**L4** as a white solid (47.2 mg, 11%). ^{1}H NMR (400 MHz, $CDCl_3$, 27 °C, TMS) δ = 9.01 - 8.99 (m, 1H), 8.31 (d, *J* = 8.1 Hz, 1H), 8.08 - 8.04 (m, 1H), 7.40 - 7.31 (m, 5H), 5.50 (dd, *J* = 10.3, 8.6 Hz, 1H), 4.94 (dd, *J* = 10.3, ^2J = 8.6 Hz, 1H), 4.44 (dd, *J* = 8.6, ^2J = 8.6 Hz, 1H); ^{13}C NMR (101 MHz, $CDCl_3$, 27 °C, TMS) δ = 162.8, 149.8, 146.7 (q, $^3J_{C-F}$ = 3.1 Hz), 141.3, 138.6, 134.9, 134.0 (q, $^3J_{C-F}$ = 3.1 Hz), 128.9 (2 × C), 127.9, 126.8 (2 × C), 124.0, 75.6, 70.5; ^{19}F NMR (376 MHz, $CDCl_3$) δ = -62.6 (s, 3F); $[\alpha]_D^{25}$ = -34 (*c* 0.1 in EtOH); ν_{max} / cm^{-1} 3251, 3066, 3034, 2983, 2923, 1634, 1602, 1400, 1318, 1168, 1134, 1121, 1100, 1084, 1073, 1013, 938, 751, 703, 680. All spectroscopic data is in agreement with the literature.[5]

Synthesis of (*S*)-2-(5-nitropyridin-2-yl)-4-phenyl-4,5-dihydrooxazole, PyrOx (*S*)-**L5**

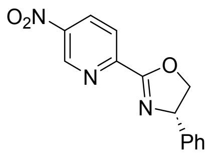

PyrOx (*S*)-**L5**
$C_{14}H_{11}N_3O_3$
MW 269.260

General procedure A was used with zinc trifluoromethanesulfonate (122 mg), 5-nitropicolinonitrile (250 mg, 1.68 mmol) and (*S*)-2-amino-2-phenylethan-1-ol (230 mg) to afford PyrOx (*S*)-**L5** as a cream gum (74 mg, 16%). ^{1}H NMR (400 MHz, $CDCl_3$, 25 °C, TMS) δ = 9.54 - 9.52 (m, 1H), 8.60 (dd, *J* = 8.6, ^4J = 2.5 Hz, 1H), 8.38 (d, *J* = 8.6 Hz, 1H), 7.40 - 7.31 (m, 5H), 5.53 (dd, *J* = 10.2, 8.7 Hz, 1H), 4.97 (dd, *J* = 10.2, ^2J = 8.7 Hz, 1H), 4.47 (dd, *J* = 8.7, ^2J = 8.7 Hz, 1H); ^{13}C NMR (101 MHz, $CDCl_3$, 25 °C, TMS) δ = 162.3, 151.3, 145.1, 145.0, 141.0, 131.9, 128.9 (2 × C), 128.0, 126.7 (2 × C), 124.6, 75.7, 70.6; $[\alpha]_D^{25}$ = -67 (*c* 1.0 in $CHCl_3$); ν_{max} / cm^{-1} 3055, 2968, 2904, 1639, 1601, 1575, 1523, 1351, 1328, 1094, 950, 855, 759, 722, 703; mp 130–133 °C; HRMS (ESI) *m*/*z* calcd for $[M+H]^+$ $C_{14}H_{12}N_3O_3$: 270.0879; found: 270.0878.

3.2 Separation of (3,4-dihydro-2*H*-pyran-2-yl)methanol enantiomers

Benzoate Derivative - Preparative Chiral Purification

Synthesis of (3,4-dihydro-2*H*-pyran-2-yl)methyl benzoate

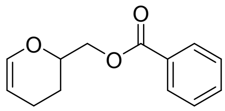

$C_{13}H_{14}O_3$
MW 218.252

To a solution of (3,4-dihydro-2*H*-pyran-2-yl)methanol (2.72 mL, 26.3 mmol) in anhydrous DCM (53 mL) was added triethylamine (11 mL, 79.0 mmol), benzoyl chloride (3.36 mL, 28.9 mmol) and DMAP (0.32 g, 2.63 mmol) at 0 °C with stirring. The resultant solution was stirred at RT for 12 h. After this time, the reaction was quenched with saturated aqueous ammonium chloride (30 mL). The layers were separated and the organic phase extracted with DCM (2 × 30 mL). The combined organic phases were dried (phase separator) and the solvent removed *in vacuo*. Purification by automated column chromatography (0-100% ethyl acetate in heptane) afforded (3,4-dihydro-2*H*-pyran-2-yl)methyl benzoate as a bronze-coloured liquid (5.84 g, 94%). ^{1}H NMR (400 MHz, $CDCl_3$, 25 °C, TMS) δ = 8.10 - 8.05 (m, 2H), 7.56 (t, *J* = 7.4 Hz, 1H), 7.44 (t, *J* = 7.4 Hz, 2H), 6.40 (br d, *J* = 6.2 Hz, 1H), 4.75 - 4.71 (m, 1H), 4.43 (d, *J* = 4.9 Hz, 2H), 4.22 - 4.15 (m, 1H), 2.20 - 2.10 (m, 1H), 2.08 - 1.91 (m, 2H), 1.85 - 1.74 (m, 1H); ^{13}C NMR (101 MHz, $CDCl_3$, 25 °C, TMS) δ = 166.5, 143.4, 133.1, 130.0, 129.7 (2 × C), 128.4 (2 × C), 100.5, 72.8, 66.6, 24.4, 19.2; ν_{max} / cm^{-1} 3062, 2926, 2851, 1718, 1649, 1602, 1584, 1270, 1239, 1112, 1067, 709. All spectroscopic data was in agreement with the literature.[6]

Conditions for chiral purification:

For small scale (<6 g (3,4-dihydro-2*H*-pyran-2-yl)methyl benzoate) preparative chiral purification, the following conditions were used:

Further chiral purification by chiral chromatography (Chiralpak® AD-H (5 µm, 250 mm × 30 mm) column, 18 °C, 2% v/v EtOH in heptane, 40 mL min^{-1}, 150 µL injection volume, UV detection (280 nm), first eluting peak t_r: 9.07 min), afforded (*S*)- (3,4-dihydro-2*H*-pyran-2-yl)methyl benzoate as a

colourless oil (2.46 g). This was followed by the second eluting peak (t_r: 10.2 min) that afforded (*R*)-(3,4-dihydro-2*H*-pyran-2-yl)methyl benzoate as a colourless oil (2.26 g).

<u>Analysis of first peak:</u>

Separation of enantiomers by Chiral HPLC, Chiralpak® AD-H (5 µm, 250 mm × 4.6 mm) column, 18 °C, 2% v/v EtOH in heptane, 1 mL min^{-1}, t_r (major): 9.07 min, t_r (minor): 10.5 min, 99.8% optical purity; $[\alpha]_D^{23}$ = +69 (*c* 1.00 in $CHCl_3$).

<u>Analysis of second peak:</u>

Separation of enantiomers by Chiral HPLC, Chiralpak® AD-H (5 µm, 250 mm × 4.6 mm) column, 18 °C, 2% v/v EtOH in heptane, 1 mL min^{-1}, t_r (major): 10.2 min, t_r (minor): 9.03 min, 97.8% optical purity; $[\alpha]_D^{23}$ = -63 (*c* 1.00 in $CHCl_3$).

Representative Preparative Chiral Chromatogram:

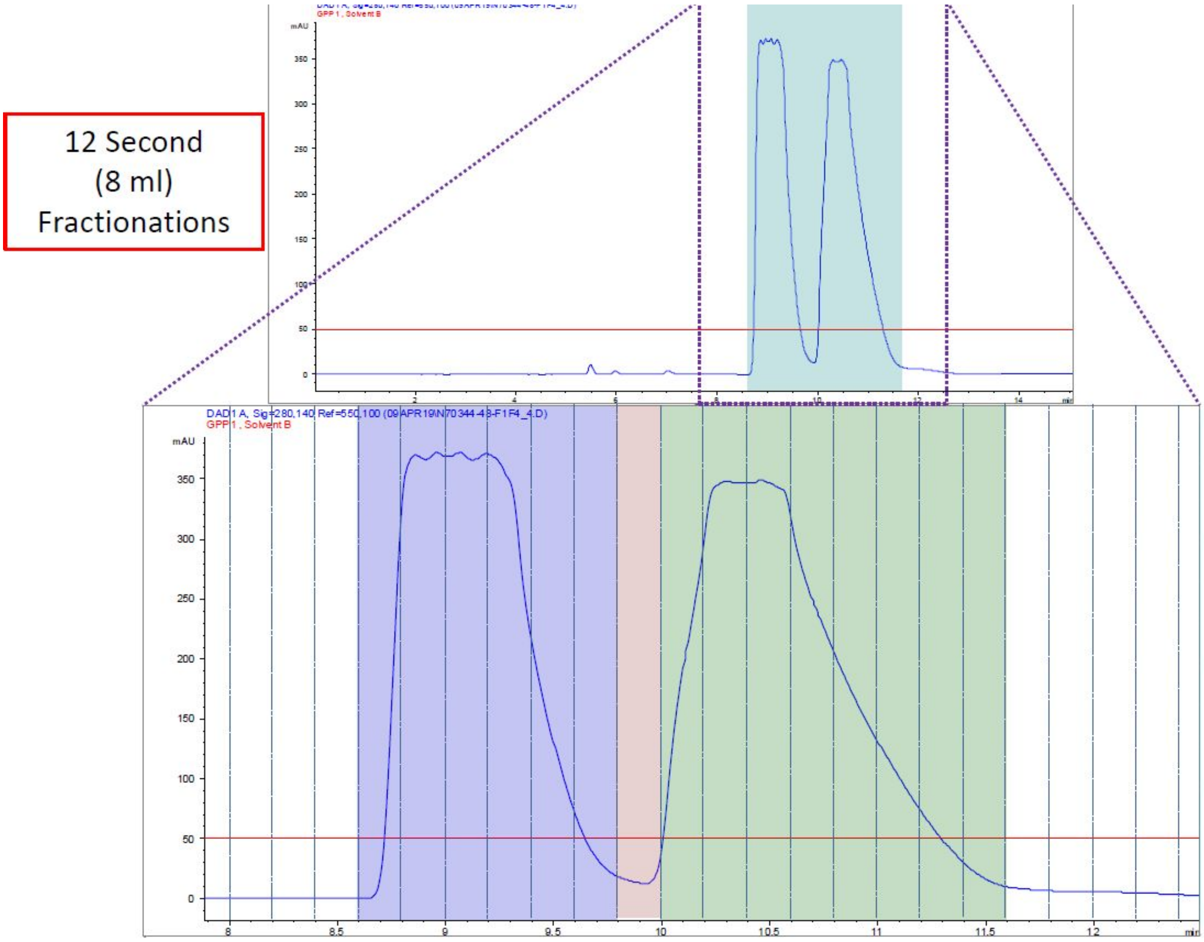

Analysis of first eluting peak:

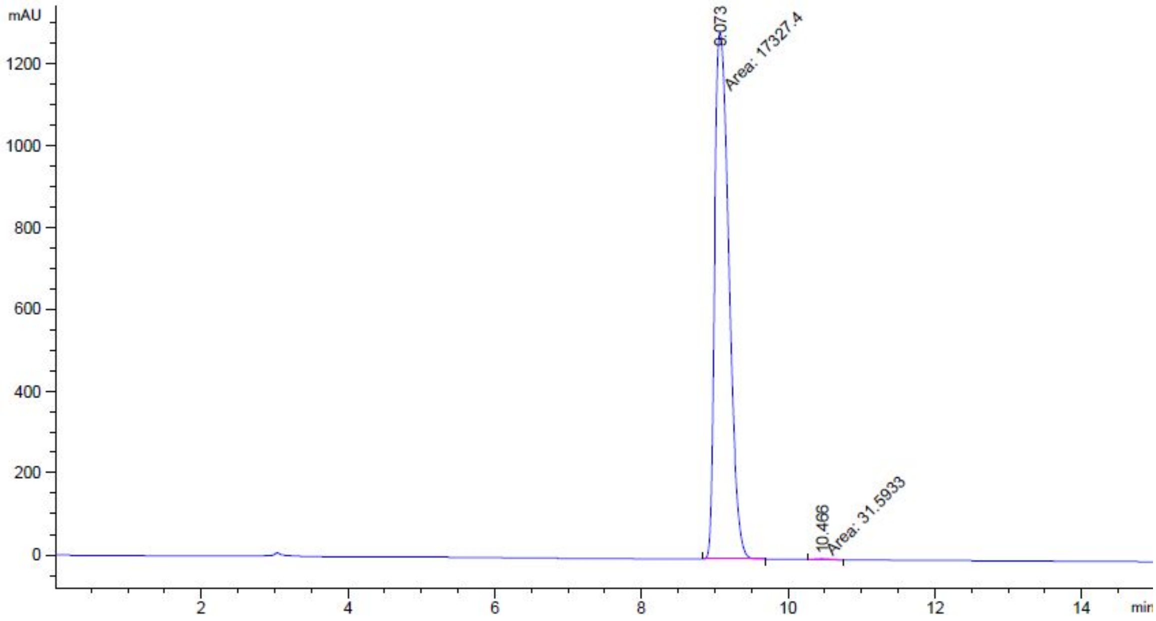

Analysis of second eluting peak:

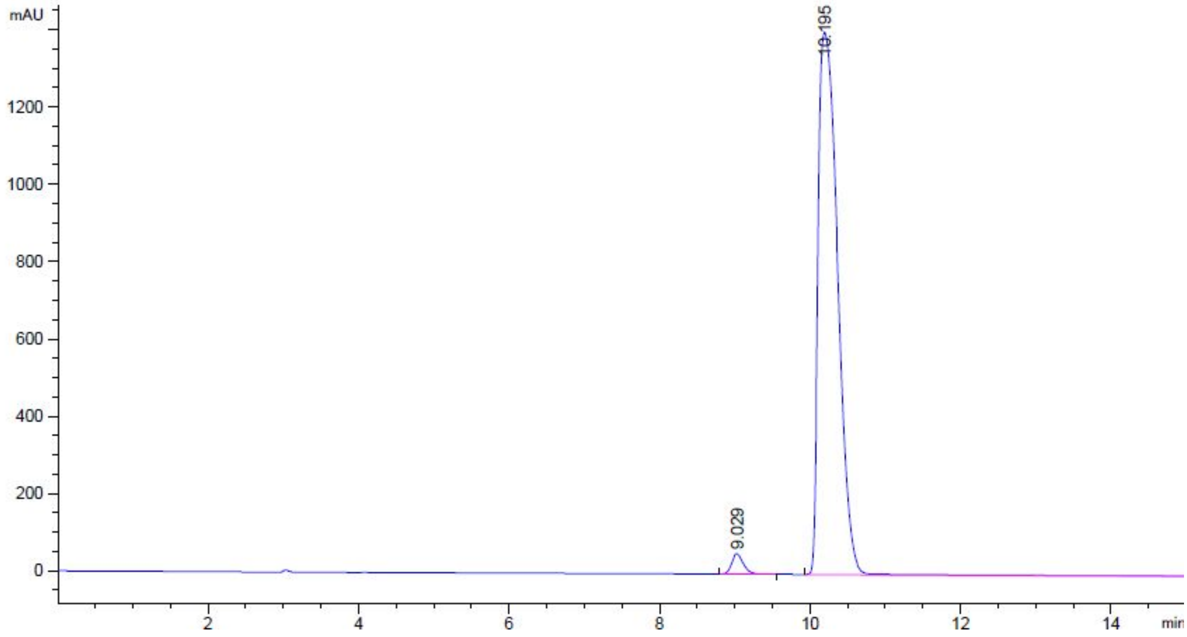

For larger scale (>8 g) 3,4-dihydro-2*H*-pyran-2-yl)methyl benzoate preparative chiral purification, the following conditions were used:

Further chiral purification by chiral chromatography (Chiralpak® IC (5 µm, 250 mm x 30 mm) column, 18 °C, 50% v/v DCM in heptane, 42 mL min^{-1}, 5000 µL injection volume of a 100 mg mL^{-1} solution in 1:1 heptane:DCM, DAD signals (290 nm, 270 nm, 230 nm), first eluting peak (peak collection: 5.9 min – 8.0 min)), afforded (*R*)- (3,4-dihydro-2*H*-pyran-2-yl)methyl benzoate as a colourless oil. This was

followed by the second eluting peak (peak collection: 8.5 min – 14.5 min) that afforded (*S*)- (3,4-dihydro-2*H*-pyran-2-yl)methyl benzoate as a colourless oil.

500 mg (3,4-dihydro-2*H*-pyran-2-yl)methyl benzoate could be processed in a single 15 minute run. For example, 8.22 g of racemate was processed in 22 runs using 15 L of mobile phase to give:
Isomer 1, (*R*)- (3,4-dihydro-2*H*-pyran-2-yl)methyl benzoate = 4.1 g as a colourless oil. Separation of enantiomers by Chiral HPLC, Chiralpak® IC (5 µm, 250 mm x 4.6 mm) column, 18 °C, 50% v/v DCM in heptane, 2 mL min^{-1}, t_r (major): 4.68 min, with a purity of 99.2% (100% *ee*).
Isomer 2, (*S*)- (3,4-dihydro-2*H*-pyran-2-yl)methyl benzoate = 3.7 g as a colourless oil. Separation of enantiomers by Chiral HPLC, Chiralpak® IC (5 µm, 250 × 4.6 mm) column, 18 °C, 50% v/v DCM in heptane, 2 mL min^{-1}, t_r (major): 10.2 min, with a purity of 98.7% (100% *ee*).

Representative Preparative Chiral Chromatogram:

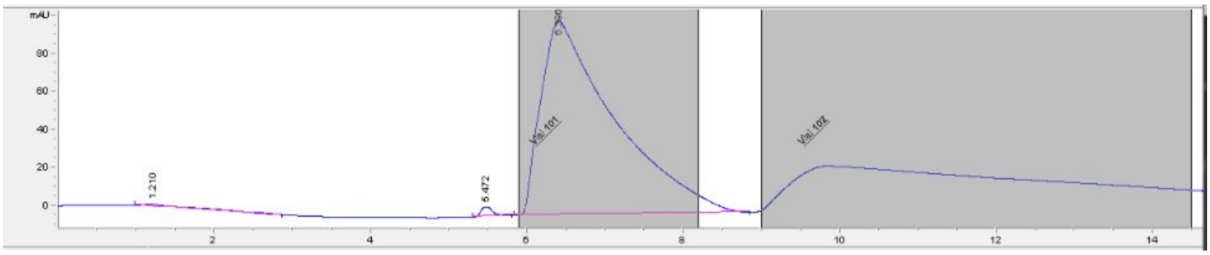

Analysis of first eluting peak:

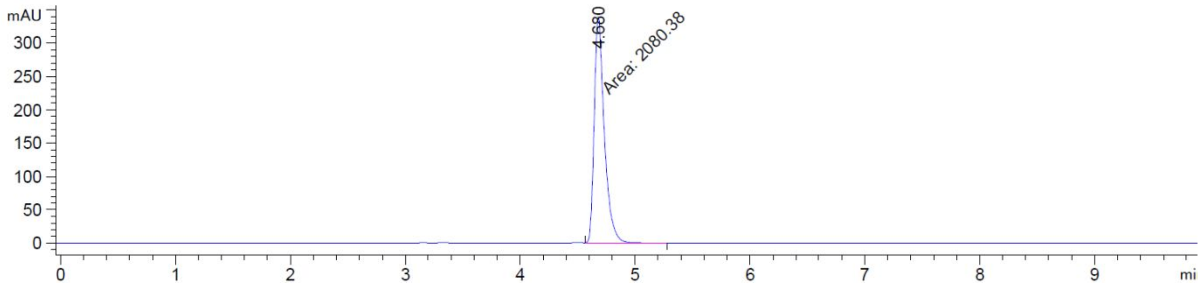

Analysis of second eluting peak:

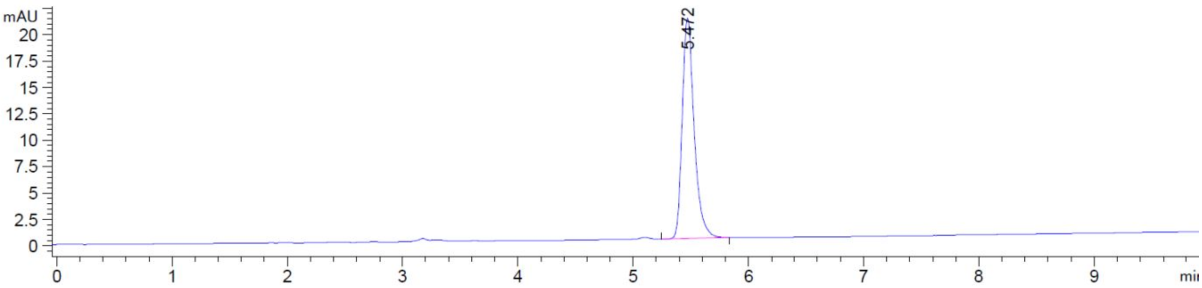

Synthesis of (*R*)-(3,4-dihydro-2*H*-pyran-2-yl)methanol, (*R*)-1

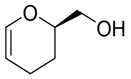

(*R*)-**1**
$C_6H_{10}O_2$
MW 114.144

To a solution of (*R*)-(3,4-dihydro-2*H*-pyran-2-yl)methyl benzoate (270 mg, 1.24 mmol) in MeOH (3.5 mL) was added sodium methoxide (180 mg, 3.33 mmol). The resultant solution was left stirring at RT under nitrogen for 15 h. After this time, the solvent was removed *in vacuo* and the crude residue was purified by automated column chromatography (0-10% MeOH in DCM) to give (*R*)-**1** as a pale yellow oil (80 mg, 57%). ^{1}H NMR (400 MHz, $CDCl_3$, 25 °C, TMS) δ = 6.39 (br d, *J* = 6.2 Hz, 1H), 4.73 - 4.69 (m, 1H), 3.96 - 3.89 (m, 1H), 3.75 - 3.62 (m, 2H), 2.05 - 1.94 (m, 2H), 1.84 - 1.63 (m, 2H); ^{13}C NMR (101 MHz, $CDCl_3$, 25 °C, TMS) δ = 143.3, 100.8, 75.5, 65.5, 23.9, 19.4; ν_{max} / cm^{-1} 3325, 2906, 1650, 1384, 1277, 1239, 1064, 1033, 712; $[\alpha]_D^{23}$ = -71 (*c* 1.07 in $CHCl_3$) [lit [7] $[\alpha]_D^{25}$= -74, *c* 1.07 in $CHCl_3$]. All spectroscopic data was in agreement with the literature.[7]

Synthesis of (*S*)-(3,4-dihydro-2*H*-pyran-2-yl)methanol, (*S*)-1

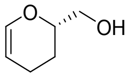

(*S*)-**1**
$C_6H_{10}O_2$
MW 114.144

To a solution of (3,4-dihydro-2*H*-pyran-2-yl)methyl benzoate (245 mg, 1.12 mmol) in MeOH (3.5 mL) was added sodium methoxide (182 mg, 3.36 mmol). The resultant solution was left stirring at RT under nitrogen for 15 h. After this time, the solvent was removed *in vacuo* and the crude residue was purified by automated column chromatography (0-10% MeOH in DCM) to give (*S*)-**1** as a pale yellow oil (74 mg, 58%). Characterisation was the same as for the opposite enantiomer, except that $[\alpha]_D^{23}$ = +91 (*c* 1.1 in $CHCl_3$). All spectroscopic data was in agreement with the literature.[7]

Acetate Derivative – Enzymatic Resolution

Synthesis of (3,4-dihydro-2*H*-pyran-2-yl)methyl acetate

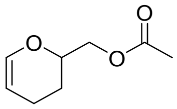

$C_8H_{12}O_3$
MW 156.181

To a solution of (3,4-dihydro-2*H*-pyran-2-yl)methanol (2.72 mL, 26.3 mmol) in dry DCM (53 mL) was added triethylamine (7.33 mL, 52.6 mmol) with stirring. The reaction mixture was cooled to 0 °C and then acetic anhydride (2.98 mL, 31.5 mmol) was added dropwise. The resultant solution was allowed to warm to RT and then left stirring at RT for a further 1 h. After 1 h, more acetic anhydride (1.49 mL, 15.77 mmol) was slowly added. The solution was then left stirring at RT for 12 h. The reaction was quenched with saturated aqueous ammonium chloride (20 mL), the layers separated and the organic phase washed with saturated aqueous ammonium chloride (20 mL). The organic phase was then dried (phase separator) and the solvent removed *in vacuo*. Purification by automated column chromatography (9:1 DCM:diethyl ether) afforded (3,4-dihydro-2*H*-pyran-2-yl)methyl acetate as a colourless oil (4.1 g, Quant.). ^{1}H NMR (400 MHz, $CDCl_3$, 25 °C, TMS) δ = 6.43 - 6.38 (m, 1H), 4.76 - 4.72 (m, 1H), 4.26 - 4.14 (m, 2H), 4.10 - 4.03 (m, 1H), 2.15 - 2.11 (m, 4H), 2.06 - 1.96 (m, 1H), 1.90 - 1.82 (m, 1H), 1.77 - 1.64 (m, 1H); ^{13}C NMR (101 MHz, $CDCl_3$, 25 °C, TMS) δ = 171.0, 143.3, 100.6, 72.7, 66.3, 24.2, 20.9, 19.2; ν_{max} / cm^{-1} 3062, 2928, 2852, 1826, 1738, 1650, 1368, 1224, 1070, 1044, 735. All spectroscopic data was in agreement with the literature.[7]

Synthesis of (*R*)-(3,4-dihydro-2*H*-pyran-2-yl)methyl acetate

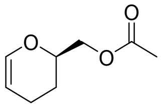

$C_8H_{12}O_3$
MW 156.181

To a stirred suspension of porcine pancreatic lipase (1.86 g, type II, 100-500 units/mg protein (using olive oil (30 min incubation)), 30-90 units/mg protein (using triacetin)) in potassium phosphate buffer (0.01 M, pH 7.6, 300 mL) in an Easymax 402 vessel, was added a solution of (3,4-dihydro-2*H*-pyran-2-yl)methyl acetate (20 g, 128 mmol) in acetone (100 mL). The resultant solution, stirred at 20 °C under nitrogen, was maintained at a pH of 6.95 by online dosing with sodium hydroxide (3 N). The reaction was monitored by GC. At 50% conversion the reaction was stopped, ethyl acetate (100 mL) was added, the layers were separated and the aqueous phase was back-extracted with ethyl acetate (100 mL). The combined organics were washed with water (100 mL), dried (phase separator) and the solvent removed *in vacuo*. Purification by automated column chromatography (100% DCM) afforded (*R*)-(3,4-dihydro-2*H*-pyran-2-yl)methyl acetate as a pale yellow oil (9.04 g, 35%). Characterisation was in agreement with that of the racemate. $[\alpha]_D^{21}$ = -132 (*c* 0.1 in $CHCl_3$).

HPLC separation of the crude reaction mixture, submitted as 50 µL in 450 µL EtOH (Chiralpak AS-H, 5 µm, 4.6 mm × 250 mm; 99% heptane/1% *iso*-propylalcohol, 1.0 mL min^{-1}, 210 nm; t_r (major) = 7.8 min, t_r (minor) = 7.6 min), >99:1 e.r.

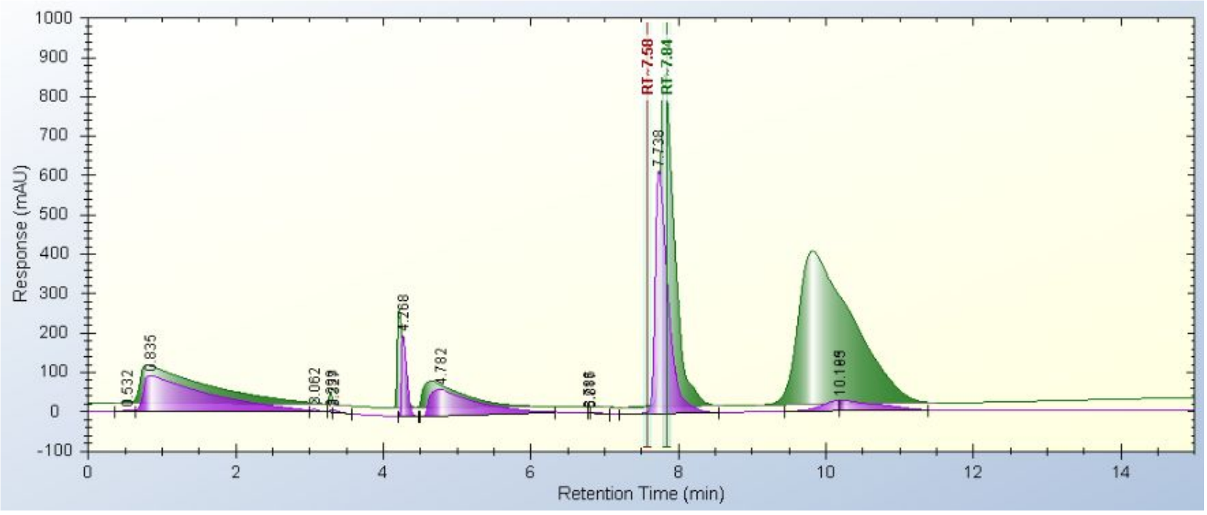

Deprotection of enantiomerically pure (3,4-dihydro-2*H*-pyran-2-yl)methyl acetate

Synthesis of (*R*)-(3,4-dihydro-2*H*-pyran-2-yl)methanol, (*R*)-**1**

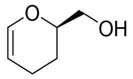

$C_6H_{10}O_2$
MW 114.144

To a solution of (3,4-dihydro-2*H*-pyran-2-yl)methyl acetate (907 mg, 5.81 mmol) in MeOH (10 mL) was added sodium methoxide (941 mg, 17.4 mmol). The resultant solution was left stirring at RT under nitrogen for 15 h. After this time, the solvent was removed *in vacuo* and the crude residue was purified by automated column chromatography (0-10% MeOH in DCM) to give (*R*)-**1** (527 mg, 80%). Characterisation was in agreement with previous syntheses of the compound (*see above*). $[\alpha]_D^{23}$ = -65 (*c* 1.07 in $CHCl_3$) [lit.[7] $[\alpha]_D^{25}$= -74, *c* 1.07 in $CHCl_3$].

3.3 Proof of Concept Reaction (Manuscript Scheme 1)

General Procedure D was used with (*p*-fluorophenyl)boronic acid (123 mg, 0.88 mmol, 2.0 eq.), except that PyrOx ligand **L0** (5.7 mg, 26.0 µmol, 6 mol%) was used in place of PyrOx ligand **L1**. Purification by automated column chromatography (0-100% ethyl acetate in heptane) afforded **2a** as a colourless oil (43 mg, 46%).

Enantiomeric ratio by chiral HPLC (Chiralpak AD-H, 5 µm, 4.6 mm × 250 mm; 90% heptane/10% EtOH (+0.2% iso-propylamine), 1.0 mL min-1, 215 nm; t_r (major) = 7.9 min, t_r (minor) = 9.6 min), 97:3 e.r.

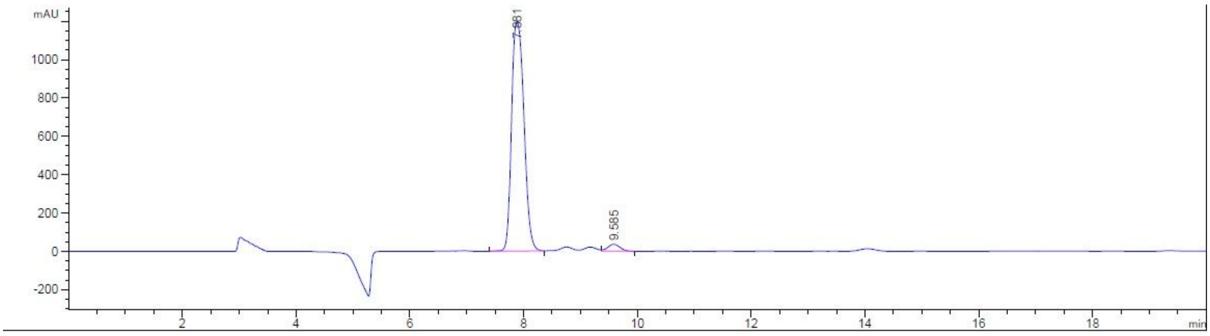

Peak #	RetTime [min]	Type	Width [min]	Area [mAU*s]	Height [mAU]	Area %
1	7.881	BB	0.2441	1.80906e4	1198.56604	97.0697
2	9.585	VB	0.2315	546.10614	36.30303	2.9303

See Section 3.7 for the method by which the retention times of the two enantiomers were established.

3.4 Ligand Screen

These results were not presented in the manuscript. The ligand screen described below demonstrates that use of PyrOx ligand **L1** led to the most effective combination of yield and enantioselectivity when employing racemic alcohol *rac*-**1** as the substrate.

General Procedure B was used to obtain the data shown below.

Table S1. Effect of PyrOx ligand structure on the yield and enantiomeric ratio of the redox relay oxidative Heck process.

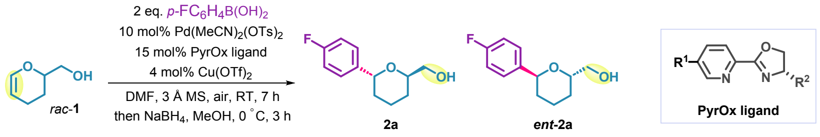

PyrOx ligand	R^1	R^2	Yield[a] / %	e.r. (**2a**:***ent*-2a**)
**L1[b]**	**CF_3**	**^{t}Bu**	**27**	**94:6**
L2	NO_2	^{t}Bu	34	93:7
L3	OMe	^{t}Bu	5	95:5
L4	CF_3	Ph	14	69:31
L5	NO_2	Ph	44	65:35

[a] $^{19}F\{^1H\}$ NMR solution yield of **2a** after 7 h; [b] Average of three runs.

For a computational rationale for these results, see Appendix 1.

Analytical samples were prepared by taking a 20 µL aliquot of the reaction mixture and diluting it in 600 µL $CDCl_3$ after 7 h. Solution yields were determined by comparing the integration of the internal standard (difluoromethylbenzene, δ -110.66 ppm) and product (δ -115.01 ppm) by $^{19}F\{^1H\}$ NMR.

3.5 Match/Mis-match Effects (Manuscript Figure 1)

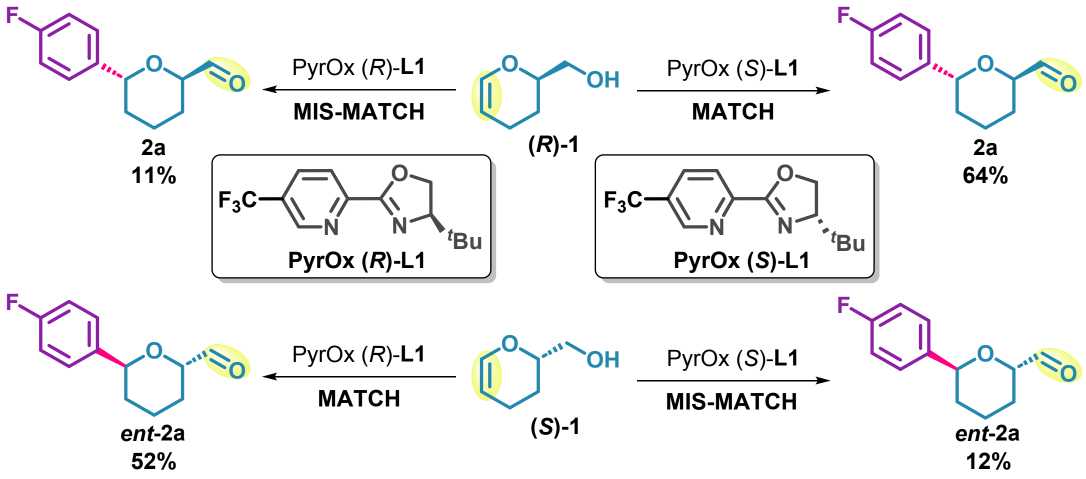

To a glass vial was added bis(acetonitrile)palladium(II) *p*-toluenesulfonate (23.3 mg, 10 mol%), PyrOx (*S*)-**L1** (17.9 mg, 15 mol%) or PyrOx (*R*)-**L1** (17.9 mg, 15 mol%), copper(II) trifluoromethanesulfonate (6.3 mg, 4 mol%), difluoromethylbenzene (10.00 mg), 6 × 3 Å molecular sieve beads (~250 mg) and anhydrous DMF (2.2 mL, 0.2 M). The resulting mixture was stirred (1000 rpm) open to air at RT for 15 min. A solution of (*R*)- or (*S*)-(3,4-dihydro-2*H*-pyran-2-yl)methanol (50.0 mg, 0.438 mmol, 1.0 eq.) and (*p*-fluorophenyl)boronic acid (123 mg, 0.88 mmol, 2.0 eq.) in anhydrous DMF (2.2 mL, 0.2 M) was then added in a single portion. The resulting mixture was then stirred (1000 rpm) open to air at RT for 24 h. Analytical samples were prepared by taking a 20 µL aliquot of the reaction mixture and diluting it in $CDCl_3$ (600 µL) at each time point. Solution yields were determined by comparing the integration of an added internal standard (difluoromethylbenzene, δ -110.66 ppm) and product (δ -115.01 ppm) by $^{19}F\{^1H\}$ NMR.

The resultant timecourse data shown in **Figure 1** of the manuscript are reproduced below in **Figure S1**. Racemic DHP-alcohol *rac*-**1** (purple data points, "DHP-alcohol, PyrOx **L1**") was also included in this study.

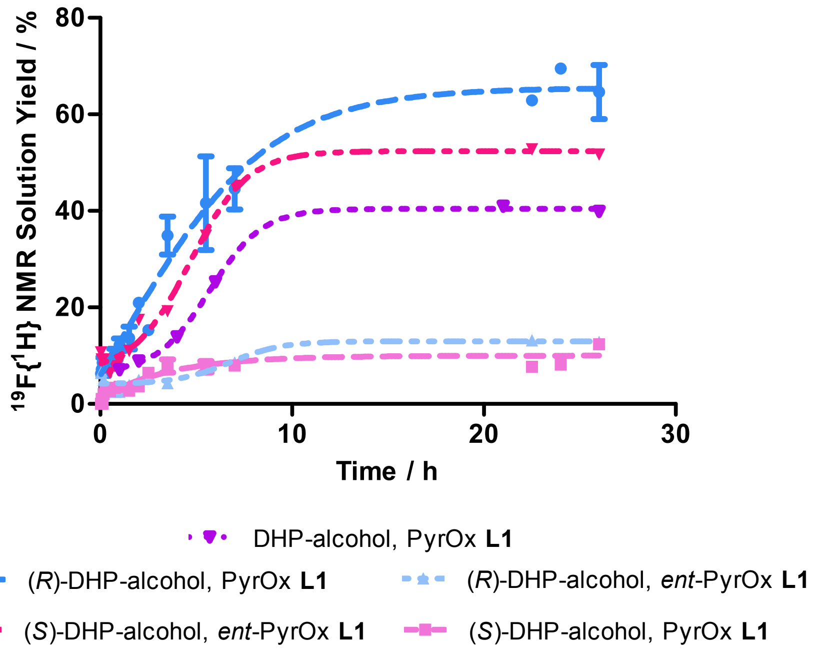

Figure S1. Match/mis-match data with enantiomerically pure DHP-alcohol and PyrOx **L1**.

3.6 Use of *rac*-(3,4-dihydro-2*H*-pyran-2-yl)methanol **1** in a Kinetic Resolution-Type Process

The reaction of racemic alcohol **1**, in a kinetic resolution-type process, was investigated, using the following general procedure:

To a glass vial was added the palladium catalyst (10 mol%; 23.4 mg, 0.044 mmol for $Pd(MeCN)_2(OTs)_2I$; 9.8 mg, 0.044 mmol for $Pd(OAc)_2$), PyrOx **L1** (17.9 mg, 15 mol%), copper(II) trifluoromethanesulfonate (4 mol% when added, 0.018 mmol, 6.4 mg), difluoromethylbenzene internal standard (10.0 mg), 6 × 3 Å molecular sieve beads (~250 mg) and anhydrous DMF (2.2 mL, 0.2 M). The resulting mixture was stirred open to air (800 rpm) for 15 min. A solution of (3,4-dihydro-2H-pyran-2-yl)methanol **1** (50.0 mg, 0.438 mmol, 1.0 eq.) and 4-fluorophenylboronic acid (123 mg, 0.88 mmol, 2.0 eq.) in anhydrous DMF (2.2 mL, 0.2 M) was then added in a single portion. Water (0-1.0 eq.) was then quickly added. The resulting mixture was stirred (800 rpm) open to air. After this time, an analytical sample was prepared by taking a 20 µL aliquot of the reaction mixture and diluting it in $CDCl_3$ (600 µL). Solution yields were determined by comparing the integration of the internal standard (difluoromethylbenzene, 10 mg, δ -110.66 ppm) and product (δ -115.01 ppm) by $^{19}F\{^1H\}$ NMR spectroscopy. The reaction mixture was diluted with ethyl acetate (10 mL), quenched with 5% aqueous lithium chloride (5 mL), the layers separated, and the organic phase washed with 5% aqueous lithium chloride (4 × 5 mL). The combined aqueous phases were back-extracted with ethyl acetate (15 mL), and the combined organic phases dried (phase separator) and the solvent removed *in vacuo*. The crude material was dissolved in MeOH (3.5 mL), placed under an atmosphere of nitrogen and cooled to 0 °C. Sodium borohydride (166 mg, 10.0 eq.) was subsequently added and the resultant solution stirred at 0 °C for 3 h. After this time, ethyl acetate (4 mL) and saturated aqueous brine (4 mL) were added and the layers separated. The aqueous phase was extracted with ethyl acetate (3 × 4 mL), the combined organics dried (phase separator) and the solvent removed *in vacuo*. Purification by automated column chromatography afforded the desired product.

The palladium source, amount of copper additive, temperature, and reaction time were investigated, and the results summarised in **Table S2**.

Table S2. Kinetic resolution-type process optimisation.

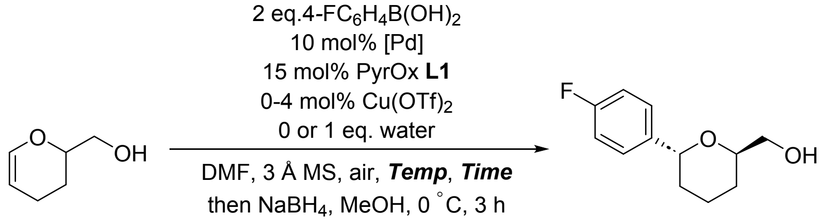

rac-**1** **3a**

Entry	[Pd]	[Cu] / mol%	T/	t/h	Yield[a]/%	e.r.
1	$Pd(MeCN)_2(OTs)_2$	4	0 °C	24	7	ND
2	$Pd(MeCN)_2(OTs)_2$	4	10 °C	46	22	98:2
3[b]	$Pd(MeCN)_2(OTs)_2$	4	RT	7	27	94:6
4[c]	$Pd(MeCN)_2(OTs)_2$	4	RT	24	39	85:15
5[d]	$Pd(MeCN)_2(OTs)_2$	4	RT	7	36	93:7
6[d]	$Pd(OAc)_2$	4	RT	7	46	85:15
7[d]	$Pd(OAc)_2$	0	RT	7	51(29[e])	95:5
8[d]	$Pd(OAc)_2$	0	RT	24	48	92:8

[a] $^{19}F\{^1H\}$ NMR solution yield of the aldehyde; [b]Average of three runs; [c]Average of two runs; [d]1 eq. water was added; [e]Isolated yield of **3a**; RT = room temperature; ND = not determined.

The full procedure using the optimal conditions from **Table S2** (Entry 7), is given below:

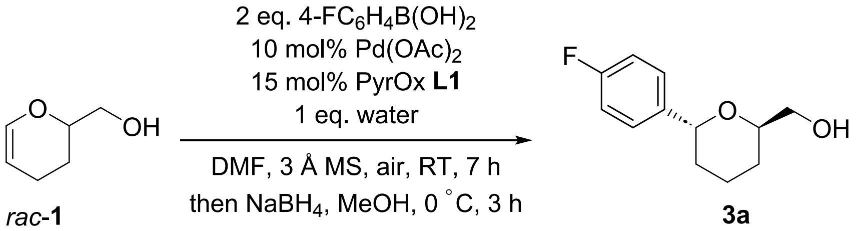

rac-**1** **3a**

To a glass vial was added palladium(II) acetate (9.8 mg, 10 mol%), PyrOx **L1** (17.9 mg, 15 mol%), 6 × 3 Å molecular sieve beads (~250 mg) and anhydrous DMF (2.2 mL, 0.2 M). The resulting mixture was stirred open to air (800 rpm) for 15 min. A solution of (3,4-dihydro-2*H*-pyran-2-yl)methanol (50.0 mg, 0.438 mmol, 1.0 eq.) *rac*-**1** and 4-fluorophenylboronic acid (123 mg, 0.88 mmol, 2.0 eq.) in anhydrous DMF (2.2 mL, 0.2 M) was then added in a single portion. Water (7.89 µL, 1.0 eq.) was then quickly added. The resulting mixture was stirred (800 rpm) open to air for 7 h. The reaction mixture was diluted with ethyl acetate (10 mL), quenched with 5% aqueous lithium chloride (5 mL), the layers separated and the organic phase washed with 5% aqueous lithium chloride (4 × 5 mL). The combined aqueous phases were back-extracted with ethyl acetate (15 mL), the combined organics dried (phase separator) and the solvent removed *in vacuo*. The crude material was dissolved in MeOH (3.5 mL), placed under an atmosphere of nitrogen and cooled to 0 °C. Sodium borohydride (166 mg, 10.0 eq.) was subsequently added and the resultant solution stirred at 0 °C for 3 h. After this time, ethyl acetate (4 mL) and saturated aqueous brine (4 mL) were added and the layers separated. The aqueous phase was extracted with ethyl acetate (3 × 4 mL), the combined organics dried (phase separator) and the

solvent removed *in vacuo*. Purification by automated column chromatography (0-100% ethyl acetate in heptane) afforded **3a** as a pale yellow oil (27 mg, 29% yield, 95:5 e.r.).

Enantiomeric ratio by chiral HPLC (Chiralpak AD-H, 5 µm, 4.6 mm × 250 mm; 90% heptane/10% EtOH (+0.2% *iso*-propylamine), 1.0 mL min^{-1}, 215 nm; t_r (major) = 8.1 min, t_r (minor) = 9.8 min), 95:5 e.r.

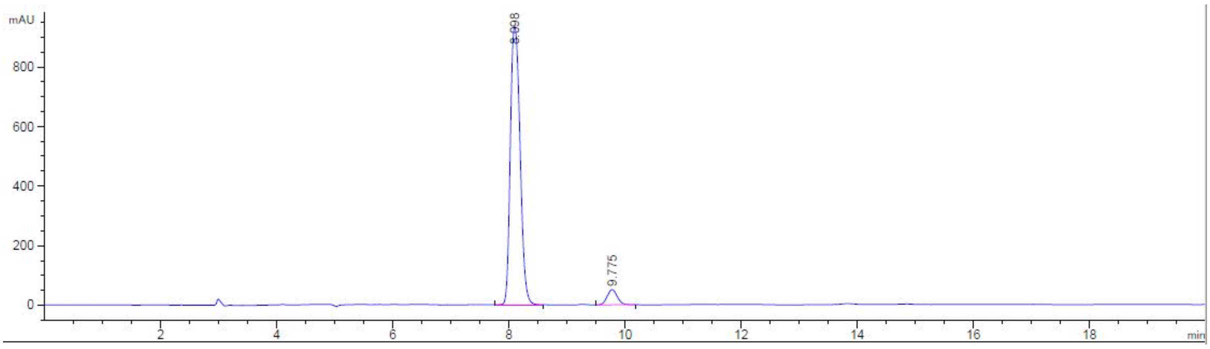

Peak #	RetTime [min]	Type	Width [min]	Area [mAU*s]	Height [mAU]	Area %
1	8.098	BBA	0.1883	1.09186e4	934.74493	94.6622
2	9.775	BB	0.1882	615.68201	51.22580	5.3378

3.7 Reaction Optimisation for Redox Relay Oxidative Heck Reaction of (*R*)-DHP-alcohol, (*R*)-**1** with 4-(Fluorophenyl)boronic acid (Manuscript Table 1)

The results of these studies are summarised in Manuscript Table 1, but the data from more extensive investigations are presented below.

Preliminary screening of reaction conditions

Variables:

- Boronic acid equivalents
    - 1.1 eq.
    - 1.5 eq.
    - 2.0 eq.
- Palladium source
    - Bis(acetonitrile)palladium(II) *p*-toluenesulfonate
    - Palladium(II) acetate
    - Palladium(II) chloride
- Solvent – selected based on solubility of reaction components at concentrations ≥0.1 M
    - DMF
    - MeOH
    - DMSO
- Co-oxidant
    - Copper(II) trifluoromethanesulfonate
    - Copper(II) chloride
    - Copper(II) acetate
    - Copper(II) acetylacetonate
    - Silver(I) nitrate
    - Silver(I) carbonate

Plate design and Set-up

		1.1 eq. BA		1.5 eq. BA		2 eq. BA	
		1	2	3	4	5	6
$Pd(MeCN)_2(OTs)_2$	A	$Cu(acac)_2$<br>DMSO	$CuCl_2$<br>MeOH	Ag_2CO_3<br>DMSO	$Cu(OTf)_2$<br>MeOH	$Cu(OAc)_2$<br>DMF	$AgNO_3$<br>DMF
$Pd(OAc)_2$	B	$AgNO_3$<br>MeOH	$Cu(OAc)_2$<br>DMSO	$Cu(acac)_2$<br>DMF	$CuCl_2$<br>DMF	$Cu(OTf)_2$<br>DMSO	Ag_2CO_3<br>MeOH
$PdCl_2$	C	Ag_2CO_3<br>DMF	$Cu(OTf)_2$<br>DMF	$Cu(OAc)_2$<br>MeOH	$AgNO_3$<br>DMSO	$CuCl_2$<br>DMSO	$Cu(acac)_2$<br>MeOH
Additional runs	D	$Pd(MeCN)_2(OTs)_2$<br>$Cu(OTf)_2$<br>DMF<br>2 eq. boronic acid	$Cu(OAc)_2$<br>DMSO<br>$Pd(OAc)_2$	$Pd(MeCN)_2(OTs)_2$<br>$Cu(OTf)_2$<br>DMF<br>2 eq. boronic acid	$Cu(OTf)_2$<br>MeOH<br>$Pd(MeCN)_2(OTs)_2$	$Pd(MeCN)_2(OTs)_2$<br>$Cu(OTf)_2$<br>DMF<br>2 eq. boronic acid	BLANK

To each 4 mL glass vial in a 24-well plate was added (*p*-fluorophenyl)boronic acid (1.1-2.0 eq.) using a QX96 weighing robot. A 924 µL aliquot from a stock solution of (*R*)-(3,4-dihydro-2*H*-pyran-2-yl)methanol (0.175 mmol per vial, 1.0 eq.) in anhydrous solvent (0.2 M) was added to each vial.

To each 4 mL glass vial in a separate 24-well plate was added palladium source (10 mol%), PyrOx **L1** (15 mol%) and co-oxidant (4 mol%) using a QX96 weighing robot. A stir bar, 3 × 3 Å molecular sieve beads (~125 mg), and a 924 µL aliquot from a stock solution of difluoromethylbenzene (equivalent to 4 mg per vial) in anhydrous solvent (0.2 M) was added to each vial. The vials were then stirred (500 rpm) open to air at RT for 15 min. After this time, the solution of boronic acid and (*R*)-(3,4-dihydro-2*H*-pyran-2-yl)methanol was added to each vial. The resultant solutions were left stirring (500 rpm) open to air at RT for 6 h.

Analytical samples were prepared at t = 3 h and t = 6 h by taking a 20 µL aliquot of the reaction mixture and diluting it in $CDCl_3$ (600 µL) at each time point. Solution yields were determined by comparing the integration of the internal standard (difluoromethylbenzene, δ -110.66 ppm) and product (δ -115.01 ppm) by ^{19}F NMR.

Results Summary

Note: the lower solution yields achieved for the control reactions in D1, D3 and D5 suggest that the reaction is mixing limited and so a high stir speed (≥800 rpm) is recommended.

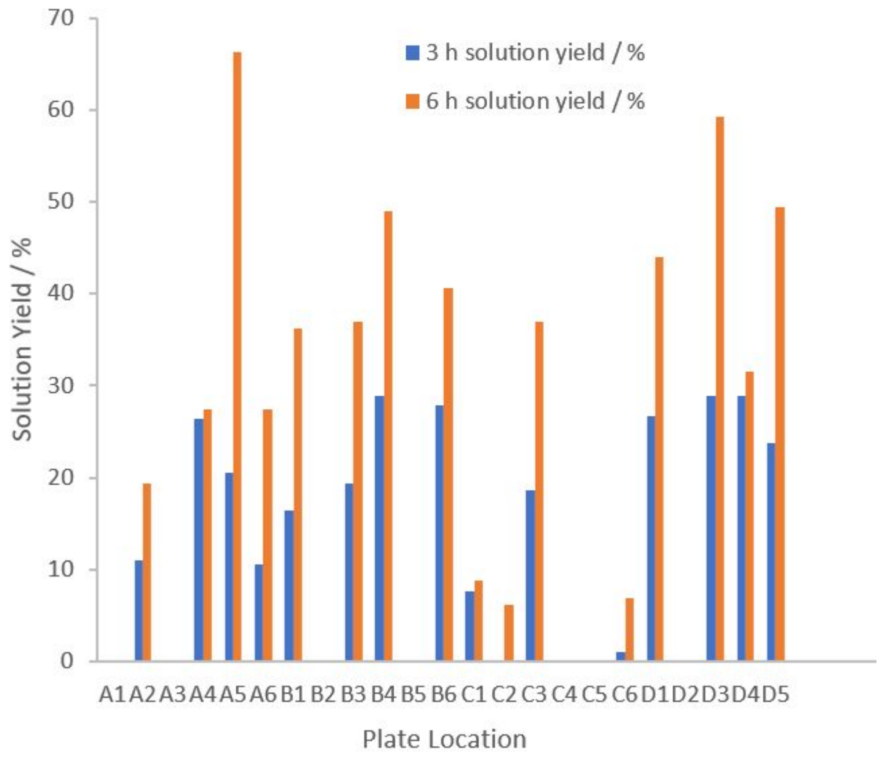

Figure S2. ^{19}F NMR Solution Yield at 3 h and 6 h.

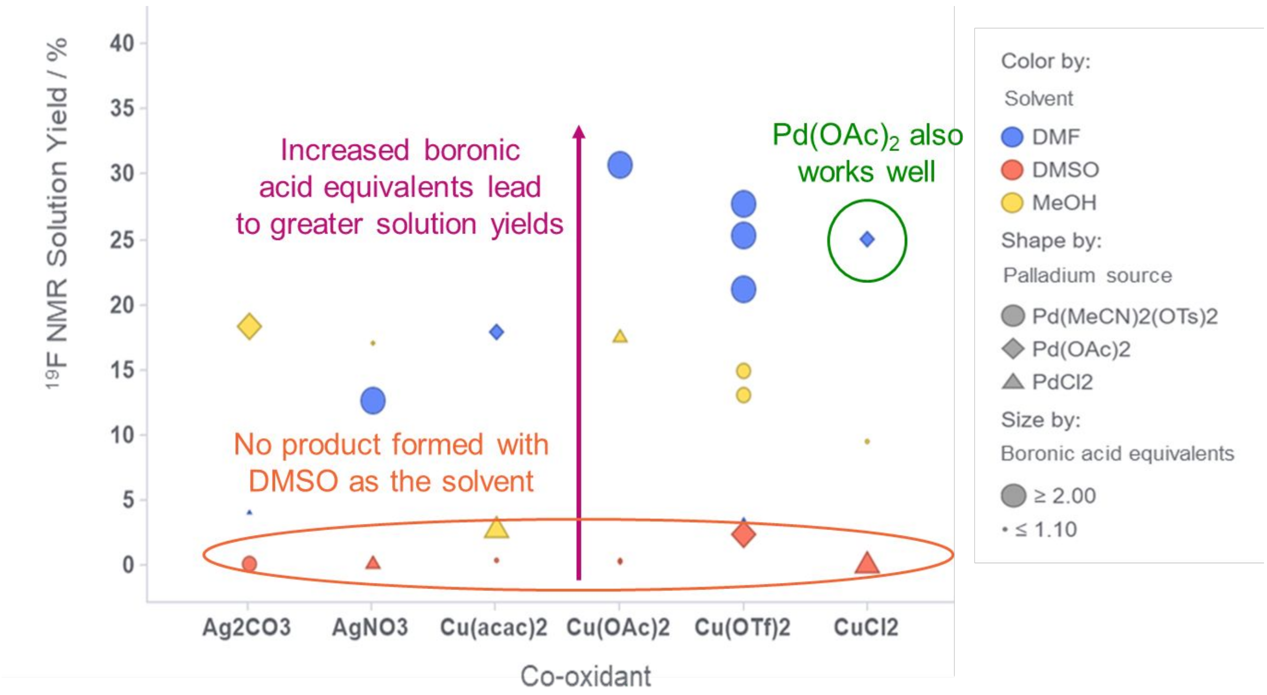

Figure S3. Trends from preliminary screening data.

Reaction Optimisation with bis(acetonitrile)palladium(II) *p*-toluenesulfonate

General Procedure C was used where palladium source refers to bis(acetonitrile)palladium(II) *p*-toluenesulfonate (10 mol% unless otherwise stated); co-oxidant refers to copper(II) trifluoromethanesulfonate (4 mol% unless otherwise stated) unless otherwise stated (other co-oxidants include benzoquinone); where molecular sieves refers 3 x 3 Å molecular sieve beads (~125 mg) unless otherwise stated (other molecular sieve sizes and methods of activation were investigated); where solvent refers to anhydrous DMF unless otherwise stated (other solvents include chloroform, cyrene, DMA, MeCN). All reactions were performed with 15 mol% PyrOx **L1**, and with 0 eq. water, open to air at RT and with 2 eq. (*p*-fluorophenyl)boronic acid unless other specific atmospheres/conditions are stated.

Table S3. Reaction optimisation for (*R*)-DHP-alcohol, (*R*)-**1**:

Entry	Conditions	Solution yield[a] / %
1	1 eq. boronic acid	26
2	2 eq. boronic acid	67
3	3 eq. boronic acid	67
4	40 °C	53
5	Cyrene as the solvent	0
6	DMA as the solvent	37
7	MeCN as the solvent	0
8	No Pd	0
9	No $Cu(OTf)_2$. No Pd	0
10	10 mol% $Ni(OAc)_2$ instead of Pd	0
11	Pd added last	60
12	3 x 3.3 mol% Pd and 5 mol% PyrOx **L1** loadings every 2.5 h	64
13[b]	Oxygen atmosphere	50
14[c]	ACE glass dispersion tube submerged in solution, attached to compressed air	-
15	needle submerged in solution, attached to compressed air	68
16	Lid on vial	42[d]
17	Nitrogen atmosphere	9
18	No $Cu(OTf)_2$	63
19	40 mol% $Cu(OTf)_2$	57
20	20 mol% $Cu(OTf)_2$, 30 mol% PyrOx **L1**	36
21	3 eq. recrystallized benzoquinone	76[e]
22	3 eq. recrystallized benzoquinone; chloroform as the solvent	75[e]
23	3 eq. non-recrystallized benzoquinone	49[e]

24	1 eq. recrystallized benzoquinone	67[e]
25	no molecular sieves	11
26	$MgSO_4$ in place of molecular sieves	32
27	4 Å molecular sieves in place of 3 Å molecular sieves	59
28	3 Å molecular sieves dried in the vacuum-oven	58
29	1 eq. water; no molecular sieves	8
30	1 eq. water and molecular sieves	76
31	3 eq. water and molecular sieves	74
32	6:10:3 mol% Pd:PyrOx **L1**:$Cu(OTf)_2$ loading	66
33	4:6:2 mol% Pd:PyrOx **L1**:$Cu(OTf)_2$ loading	45
34	6:10:3 mol% Pd:PyrOx **L1**:$Cu(OTf)_2$ loading, 1 eq. water	80
35	4:6:2 mol% Pd:PyrOx **L1**:$Cu(OTf)_2$ loading, 1 eq. water	77

[a] Solution yield reported at 24 h unless otherwise stated; [b] Oxygen atmosphere achieved by purging a 4 mL vial with a septa-containing lid with an oxygen-filled balloon until the balloon had been slowly evacuated three times. The outlet needle was then removed from the septum and a freshly-filled oxygen balloon pierced the septa for the duration of the reaction; [c] Rate of solvent evaporation made data unreliable; [d] Solution yield at 29 h *i.e.* after removing lid and sampling at 7 h, 22 h and 29 h; [e] Solution yield at 6 h. Benzoquinone systems difficult to purify.

Reaction Optimisation with palladium(II) acetate

General Procedure C was used where palladium source refers to palladium(II) acetate (10 mol% unless otherwise stated); where co-oxidant refers to 15 mol% (unless otherwise stated) copper(II) trifluoromethanesulfonate, copper(II) chloride, silver carbonate or silver nitrate; where molecular sieves refers 3 x 3 Å molecular sieve beads (~125 mg); where solvent refers to anhydrous DMF; where water refers to 0 eq. unless otherwise stated. All reactions were performed open to air at RT and with 2 eq. (*p*-fluorophenyl)boronic acid.

Table S4. Co-oxidant optimisation for (*R*)-DHP-alcohol, (*R*)-**1**:

Entry	Conditions	Solution yield[a] / %
1	$Pd(OAc)_2$, $CuCl_2$	42
2	$Pd(OAc)_2$, Ag_2CO_3	61
3	$Pd(OAc)_2$, $AgNO_3$	62
4	$Pd(OAc)_2$, $Cu(OTf)_2$	77
5	$Pd(OAc)_2$, no co-oxidant	66

[a] Solution yield reported at 24 h unless otherwise stated.

Table S5. Further reaction optimisation with palladium(II) acetate as the palladium source.

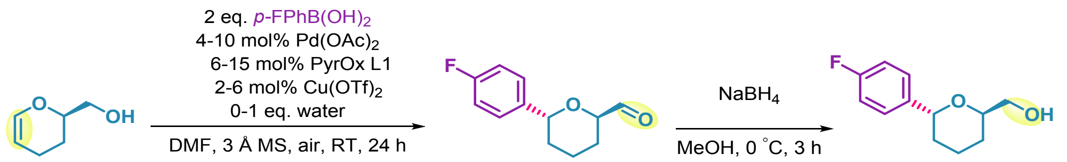

(*R*)-1 2a 3a

Entry	[Pd]:[PyrOx]:[Cu] / mol%	1 eq. water	Yield[a] / %
1	10:15:0	N	68
2	10:15:0	Y	58
3	10:15:4	N	77
**4**	**10:15:4**	**Y**	**84(70[b])**
5	6:10:3	Y	47
6	4:6:2	Y	44

[a] 24 h Solution yield of **2a** determined by $^{19}F\{^{1}H\}$ NMR; [b] isolated in 70% yield and 99:1 e.r. following reduction to alcohol **3a**; Y = yes, N = no.

Determination of enantioselectivities for reaction optimisation

To a glass vial was added palladium source, PyrOx **L1**, copper(II) trifluoromethanesulfonate, difluoromethylbenzene (10.0 mg), 6 × 3 Å molecular sieve beads (~250 mg) and anhydrous DMF (2.2 mL, 0.2 M). The resulting mixture was stirred (800 rpm) open to air at RT for 15 min. A solution of (*R*)-(3,4-dihydro-2*H*-pyran-2-yl)methanol (50 mg, 0.438 mmol, 1.0 eq.) and (*p*-fluorophenyl)boronic acid (123 mg, 0.88 mmol, 2.0 eq.) in anhydrous DMF (2.2 mL, 0.2 M) was then added in a single portion. Water (7.89 µL, 1.0 eq.) was then quickly added. The resulting mixture was stirred (800 rpm) open to air at RT for 24 h. After this time, an analytical sample was prepared by taking a 20 µL aliquot of the reaction mixture and diluting it in $CDCl_3$ (600 µL). Solution yields were determined by comparing the integration of the internal standard (difluoromethylbenzene, δ -110.66 ppm) and product (δ -115.01 ppm) by $^{19}F\{^{1}H\}$ NMR. After this time, the reaction mixture was diluted with ethyl acetate (10 mL), quenched with 5% aqueous lithium chloride (5 mL), the layers separated and the organic phase washed with 5% aqueous lithium chloride (4 × 5 mL). The combined aqueous phase was back-extracted with ethyl acetate (15 mL) and the combined organics dried (phase separator) and the solvent removed *in vacuo*. The crude material was dissolved in MeOH (3.5 mL), placed under an atmosphere of nitrogen and cooled to 0 °C. Sodium borohydride (166 mg, 10.0 eq.) was subsequently added and the resultant solution stirred at 0 °C for 3 h. After this time, ethyl acetate (4 mL) and saturated aqueous brine (4 mL) were added and the layers separated. The aqueous phase was extracted with ethyl acetate (3 × 4 mL), the combined organics dried (phase separator) and the solvent

removed *in vacuo*. Purification by automated column chromatography (0-100% ethyl acetate in heptane) afforded (((2*R*,6*R*)-6-(4-fluorophenyl)tetrahydro-2*H*-pyran-2-yl)methanol), **3a**.

3.8 Substrate Scope (Manuscript Scheme 2)

Synthesis of ((2*R*,6*R*)-6-(4-fluorophenyl)tetrahydro-2*H*-pyran-2-yl)methanol, **3a**

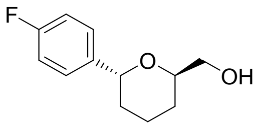

3a
$C_{12}H_{15}FO_2$
MW 210.25

General Procedure D was used with (*p*-fluorophenyl)boronic acid (123 mg, 0.88 mmol). Purification by automated column chromatography (0-100% ethyl acetate in heptane) afforded **3a** as a colourless oil (55 mg, 59%). ^{1}H NMR (400 MHz, $CDCl_3$, 30 °C, TMS): δ = 7.32 - 7.41 (m, 2H), 7.00 - 7.09 (m, 2H), 4.87 (t, *J* = 5.4 Hz, 1H), 3.75 - 3.87 (m, 2H), 3.48 - 3.59 (m, 1H), 1.89 - 2.02 (m, 3H), 1.62 - 1.76 (m, 3H), 1.46 - 1.54 ppm (m, 1H); ^{13}C NMR (101 MHz, $CDCl_3$, 30 °C, TMS): δ = 161.9 (d, $^1J_{C-F}$ = 245.0 Hz), 137.2 (d, $^4J_{C-F}$ = 2.9 Hz), 128.1 (d, $^3J_{C-F}$ = 8.1 Hz, 2 × C), 115.3 (d, $^2J_{C-F}$ = 21.3 Hz, 2 × C), 72.1, 71.9, 63.8, 29.8, 26.2, 18.8 ppm; ^{19}F{^{1}H} NMR (376 MHz, $CDCl_3$, 30 °C): δ = -115.82 ppm (s, 1F); $[\alpha]_D^{20}$ = -64 (*c* 0.1 in $CHCl_3$); ν_{max} / cm^{-1} 3396, 2937, 2868, 1640, 1603, 1508, 1221, 1037, 833. LC-MS or HRMS – no mass ion found.

Enantiomeric ratio by chiral HPLC (Chiralpak AD-H, 5 µm, 4.6 mm × 250 mm; 90% heptane/10% EtOH (+0.2% *iso*-propylamine), 1.0 mL min^{-1}, 215 nm; t_r (major) = 8.0 min, t_r (minor) = 9.7 min), >99:1 e.r.

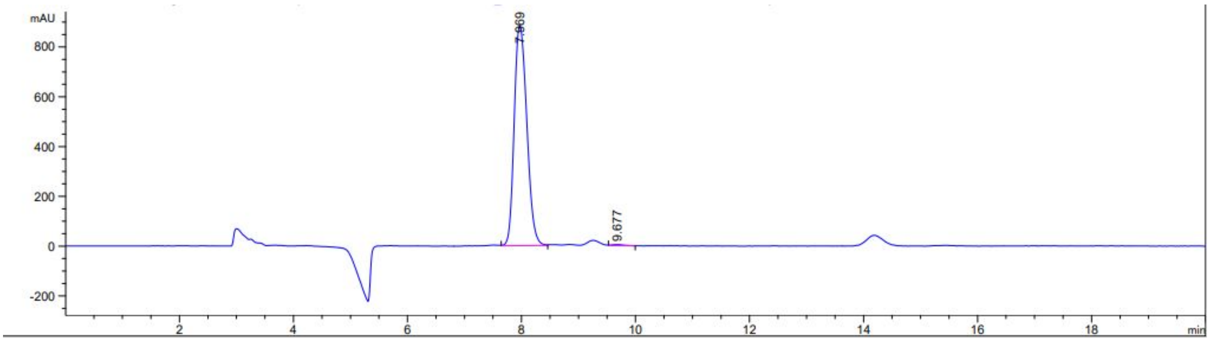

Peak #	RetTime [min]	Type	Width [min]	Area [mAU*s]	Height [mAU]	Area %
1	7.969	VV	0.2462	1.36428e4	883.39166	99.5449
2	9.677	VB	0.1859	62.37925	4.09050	0.4551

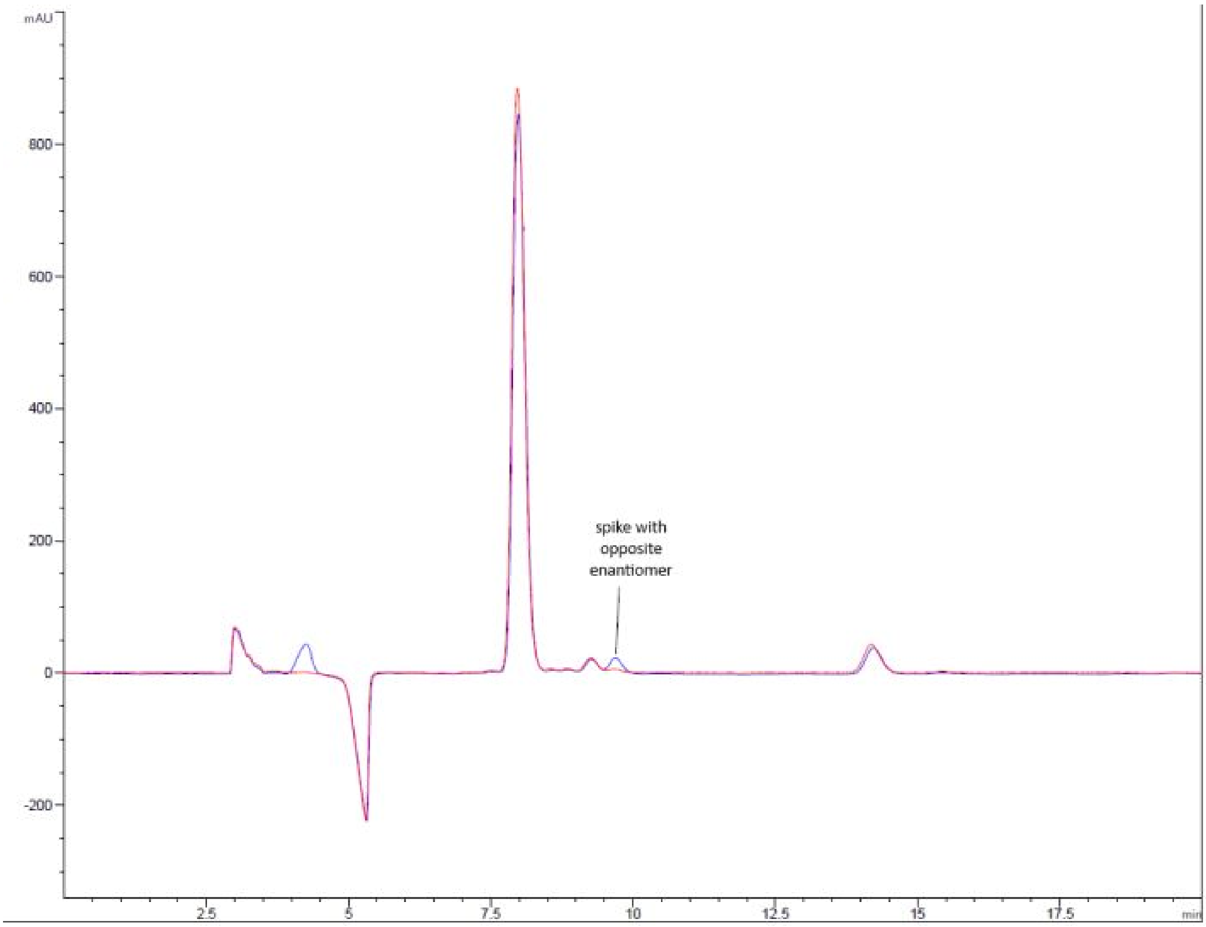

Synthesis of the ferrocenoyl-ester of **3a**, **Fc-3a**

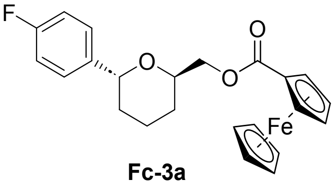

Fc-3a
$C_{23}H_{23}FFeO_3$
MW 422.10

To a solution of ((2*R*,6*R*)-6-(4-fluorophenyl)tetrahydro-2*H*-pyran-2-yl)methanol (27.0 mg, 0.128 mmol) **3a** in DCM (2.5 mL) under nitrogen was added DMAP (15.7 mg, 0.128 mmol) and ferrocenoyl chloride (31.9 mg, 0.128 mmol). The resultant solution was stirred at RT for 2 h. After this time, the reaction was quenched with water (1 mL), and the aqueous phase was back-extracted with DCM (3 x 3 mL), dried (phase separator) and the solvent removed *in vacuo*. Purification by automated column chromatography (0-30% ethyl acetate in heptane) afforded an orange oil. Recrystalisation from EtOH afforded **Fc-3a** as an orange crystalline solid (20 mg, 38%). ^{1}H NMR (400 MHz, $CDCl_3$, 30 °C, TMS): δ = 7.39 - 7.43 (m, 2H), 7.04 (tt, *J* = 8.8, 2.0 Hz, 2H), 4.94 (t, *J* = 5.4 Hz, 1H), 4.80 - 4.83 (m, 2H), 4.58 (dd, *J*

= 11.5, 8.1 Hz, 1H), 4.39 (t, *J* = 1.7 Hz, 2H), 4.37 (s, 1H), 4.18 (dd, *J* = 11.5, 4.2 Hz, 1H), 4.16 (s, 4H), 4.05 - 4.11 (m, 1H), 1.91 - 1.99 (m, 2H), 1.70 - 1.84 (m, 3H), 1.60 - 1.67 ppm (m, 1H); ^{13}C NMR (101 MHz, $CDCl_3$, 30 °C, TMS): δ = 171.6, 162.0 (d, $^1J_{C\text{-}F}$ = 244.3 Hz), 137.6 (d, $^4J_{C\text{-}F}$ = 2.9 Hz), 128.2 (d, $^3J_{C\text{-}F}$ = 7.3 Hz, 2 × C), 115.2 (d, $^2J_{C\text{-}F}$ = 21.3 Hz, 2 × C), 72.4, 71.3 (2 × C), 70.23, 70.19, 70.11, 70.09 (2 × C), 69.8 (4 × C), 64.5, 30.1, 26.5, 19.0 ppm; ^{19}F{^{1}H} NMR (376 MHz, $CDCl_3$, 30 °C): δ = -115.85 ppm (s, 1F); $[\alpha]_D^{21}$ = -42 (*c* 0.1 in $CHCl_3$); ν_{max} / cm^{-1} 2942, 1762, 1693, 1606, 1511, 1274, 1224, 1120, 1038, 822, 504; mp 76-79 °C.

From this, the following X-ray crystal structure was generated:

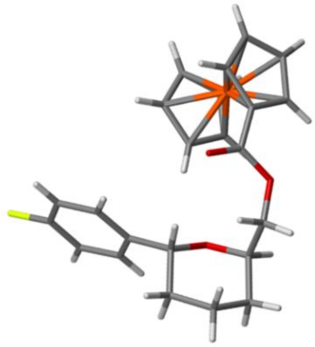

See Appendix 2 for X-ray data.

Synthesis of ((2*S*,6*S*)-6-(4-fluorophenyl)tetrahydro-2*H*-pyran-2-yl)methanol, *ent*-3a

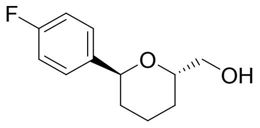

***ent*-3a**
$C_{12}H_{15}FO_2$
MW 210.25

General Procedure D was used with (*p*-fluorophenyl)boronic acid (123 mg, 0.88 mmol), except that (*S*)-(3,4-dihydro-2*H*-pyran-2-yl)methanol (50.0 mg, 0.438 mmol, 1.0 eq.) and *ent*-PyrOx **L1** (7.2 mg, 6 mol%) were used. Purification by automated column chromatography (0-100% ethyl acetate in heptane) afforded ***ent*-3a** as a colourless oil (54 mg, 59%). Characterisation was the same as for the opposite enantiomer **3a** except that $[\alpha]_D^{20}$ = +46 (*c* 0.1 in $CHCl_3$).

Enantiomeric ratio by chiral HPLC (Chiralpak AD-H, 5 µm, 4.6 mm × 250 mm; 90% heptane/10% EtOH (+0.2% *iso*-propylamine), 1.0 mL min^{-1}, 215 nm; t_r (major) = 9.5 min, t_r (minor) = 8.0 min), 99:1 e.r.

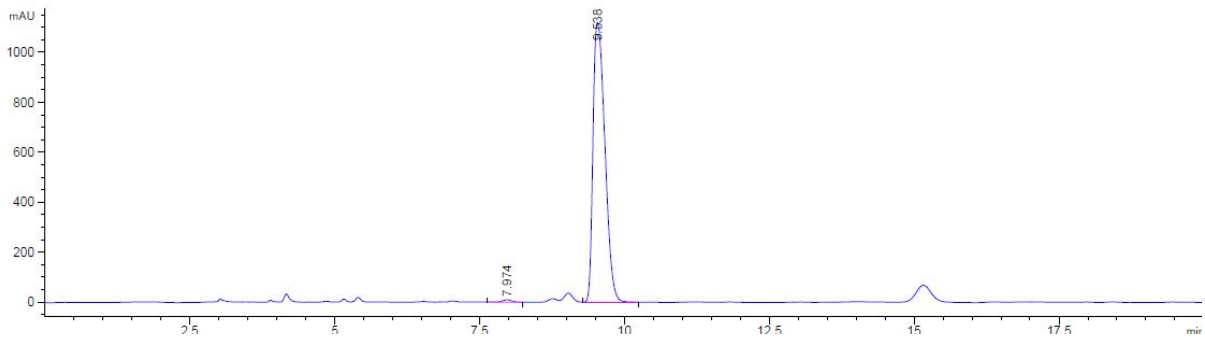

Peak #	RetTime [min]	Type	Width [min]	Area [mAU*s]	Height [mAU]	Area %
1	7.974	BB	0.1895	111.88498	9.22619	0.6986
2	9.538	VB	0.2278	1.59042e4	1118.93396	99.3014

Alternative boron derivatives:

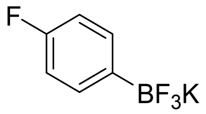

9% solution yield

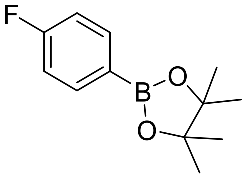

no reaction

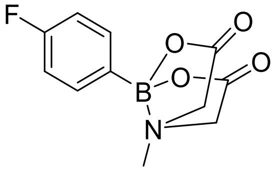

no reaction

Synthesis of ((2*R*,6*R*)-6-(3-fluorophenyl)tetrahydro-2*H*-pyran-2-yl)methanol, **3b**

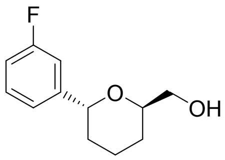

3b
$C_{12}H_{15}FO_2$
MW 210.25

General Procedure D was used with (*m*-fluorophenyl)boronic acid (123 mg, 0.88 mmol). Purification by automated column chromatography (0-100% ethyl acetate in heptane) afforded **3b** as a colourless oil (38 mg, 41%). ^{1}H NMR (400 MHz, $CDCl_3$, 30 °C, TMS): δ = 7.27 - 7.39 (m, 1H), 7.07 - 7.21 (m, 2H), 6.95 (ddd, *J* = 8.3, $^3J_{H\text{-}F}$ = 8.3, ^4J = 2.4 Hz, 1H), 4.90 (t, *J* = 5.1 Hz, 1H), 3.74 - 3.87 (m, 2H), 3.50 - 3.63 (m, 1H), 1.89 - 2.05 (m, 3H), 1.59 - 1.75 (m, 3H), 1.46 - 1.57 ppm (m, 1H); ^{13}C NMR (101 MHz, $CDCl_3$, 30 °C, TMS): δ = 163.2 (d, $^1J_{C\text{-}F}$ = 245.8 Hz), 144.3 (d, $^3J_{C\text{-}F}$ = 6.6 Hz), 130.0 (d, $^3J_{C\text{-}F}$ = 8.1 Hz), 122.0 (d, $^4J_{C\text{-}F}$ = 2.9 Hz), 113.9 (d, $^2J_{C\text{-}F}$ = 21.3 Hz), 113.5 (d, $^2J_{C\text{-}F}$ = 22.0 Hz), 72.2, 72.0, 64.0, 29.6, 26.2, 18.8 ppm; ^{19}F NMR (376 MHz, $CDCl_3$, 30 °C): δ = -113.02 ppm (s, 1F); $[\alpha]_D^{20}$ = -60 (*c* 0.1 in $CHCl_3$); ν_{max} / cm^{-1} 3396, 2937, 2867, 1613, 1589, 1487, 1441, 1242, 1100, 1038, 789; LC-MS or HRMS – no mass ion found.

Enantiomeric ratio by chiral HPLC (Chiralpak AD-H, 5 µm, 4.6 mm × 250 mm; 90% heptane/10% EtOH (+0.2% *iso*-propylamine), 1.0 mL min^{-1}, 215 nm; t_r (major) = 9.3 min, t_r (minor) = 16.1 min), >99:1 e.r.

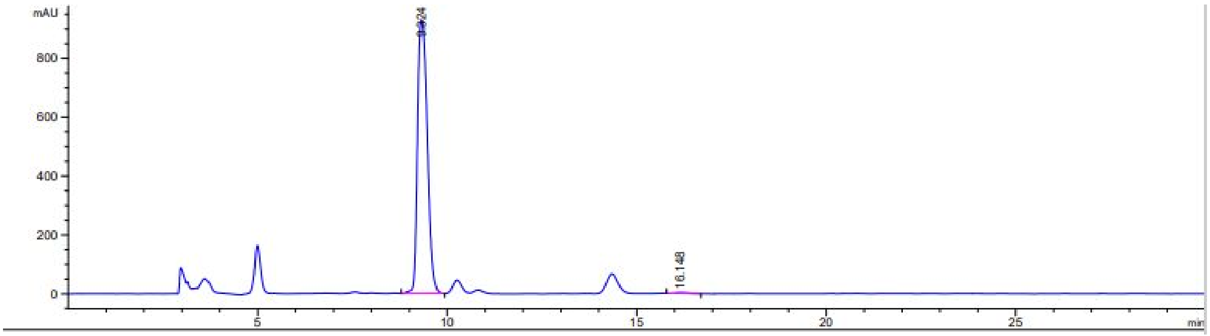

Peak #	RetTime [min]	Type	Width [min]	Area [mAU*s]	Height [mAU]	Area %
1	9.324	VB	0.2848	1.64880e4	930.11475	99.5458
2	16.148	BB	0.3258	75.23041	2.77892	0.4542

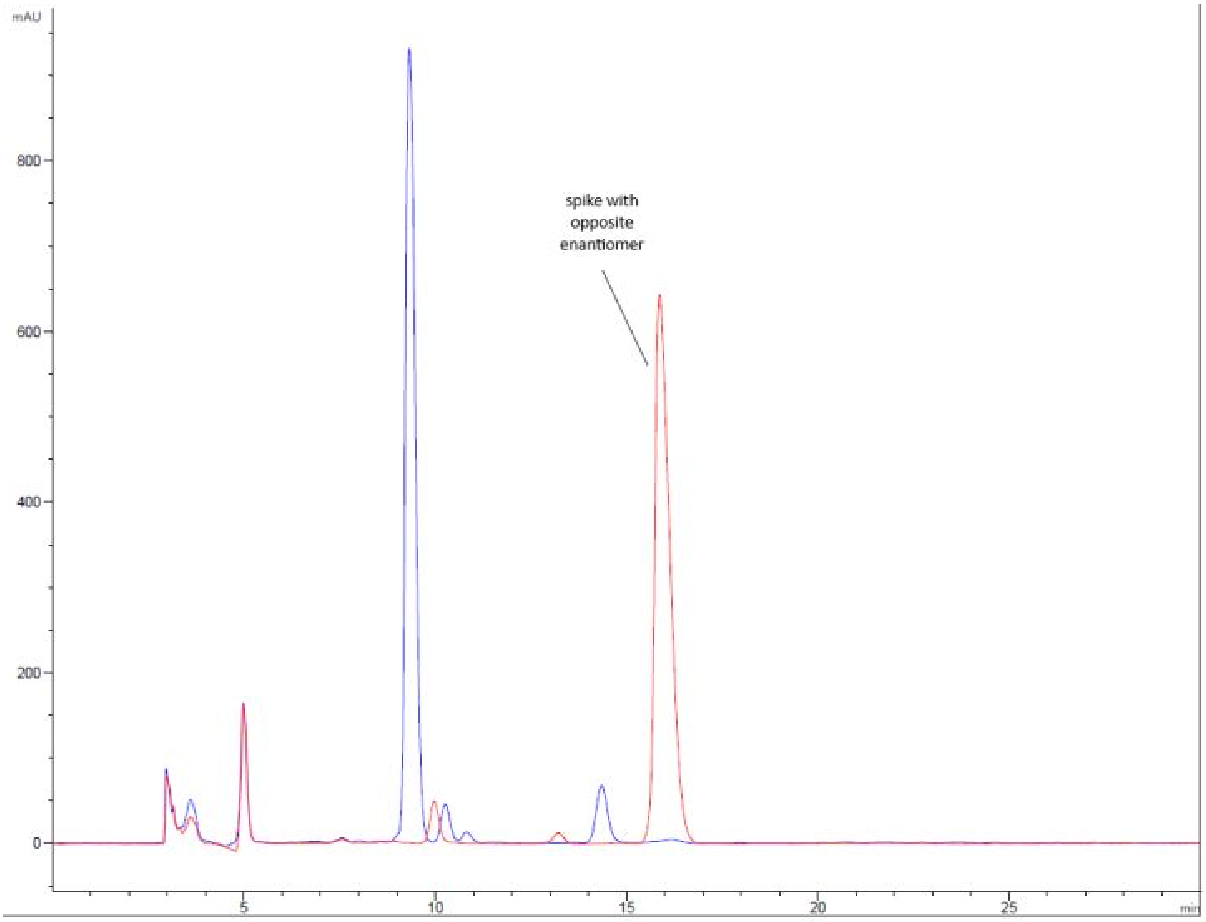

Synthesis of ((2*R*,6*R*)-6-(4-methoxyphenyl)tetrahydro-2*H*-pyran-2-yl)methanol, **3c**

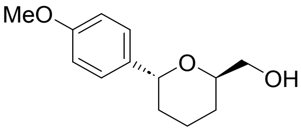

3c
$C_{13}H_{18}O_3$
MW 222.28

General Procedure D was used with (*p*-methoxyphenyl)boronic acid (133 mg, 0.88 mmol). Purification by automated column chromatography (0-100% ethyl acetate in heptane) afforded **3c** as a colourless oil (67 mg, 68%). ^{1}H NMR (400 MHz, $CDCl_3$, 30 °C, TMS): δ = 7.30 - 7.33 (m, 2H), 6.84 - 6.95 (m, 2H), 4.86 (t, *J* = 4.9 Hz, 1H), 3.74 - 3.88 (m, 5H), 3.51 (br d, *J* = 7.8 Hz, 1H), 1.86 - 2.10 (m, 2H), 1.56 - 1.76 (m, 4H), 1.42 - 1.56 ppm (m, 1H); ^{13}C NMR (101 MHz, $CDCl_3$, 30 °C, TMS): δ = 158.7, 133.5, 127.8 (2 × C), 113.9 (2 × C), 72.4, 71.7, 64.0, 55.3, 29.6, 26.3, 18.9 ppm; $[\alpha]_D^{20}$ = -62 (*c* 0.1 in $CHCl_3$); ν_{max} / cm^{-1} 3412, 2934, 2866, 1611, 1584, 1511, 1244, 1176, 1032, 819; HRMS (ESI) *m*/*z* calcd for $[M+Na]^+$ $C_{13}H_{18}O_3Na$: 245.1154; found: 245.1152.

Enantiomeric ratio by chiral HPLC (Chiralpak AD-H, 5 µm, 4.6 mm × 250 mm; 90% heptane/10% EtOH (+0.2% *iso*-propylamine), 1.0 mL min^{-1}, 215 nm; t_r (major) = 14.2 min, t_r (minor) = 18.1 min), 99:1 e.r.

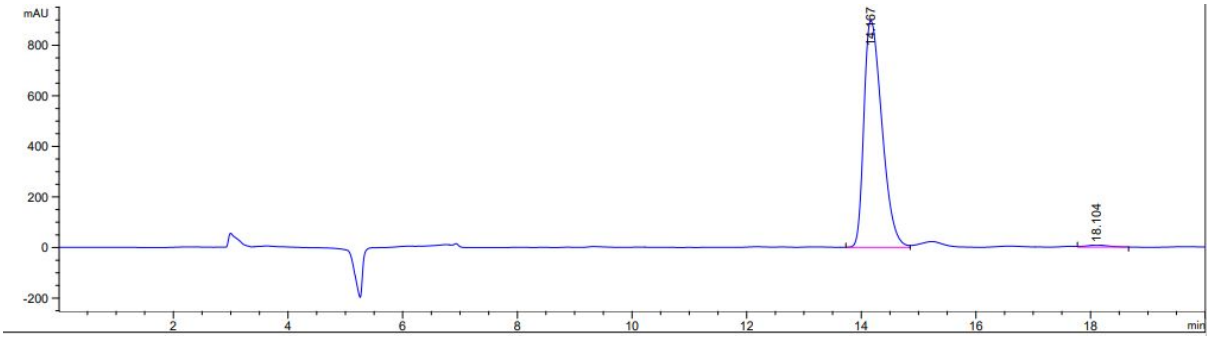

Peak #	RetTime [min]	Type	Width [min]	Area [mAU*s]	Height [mAU]	Area %
1	14.167	BV	0.3401	1.95287e4	896.47510	98.9213
2	18.104	VBA	0.3636	212.95074	8.12961	1.0787

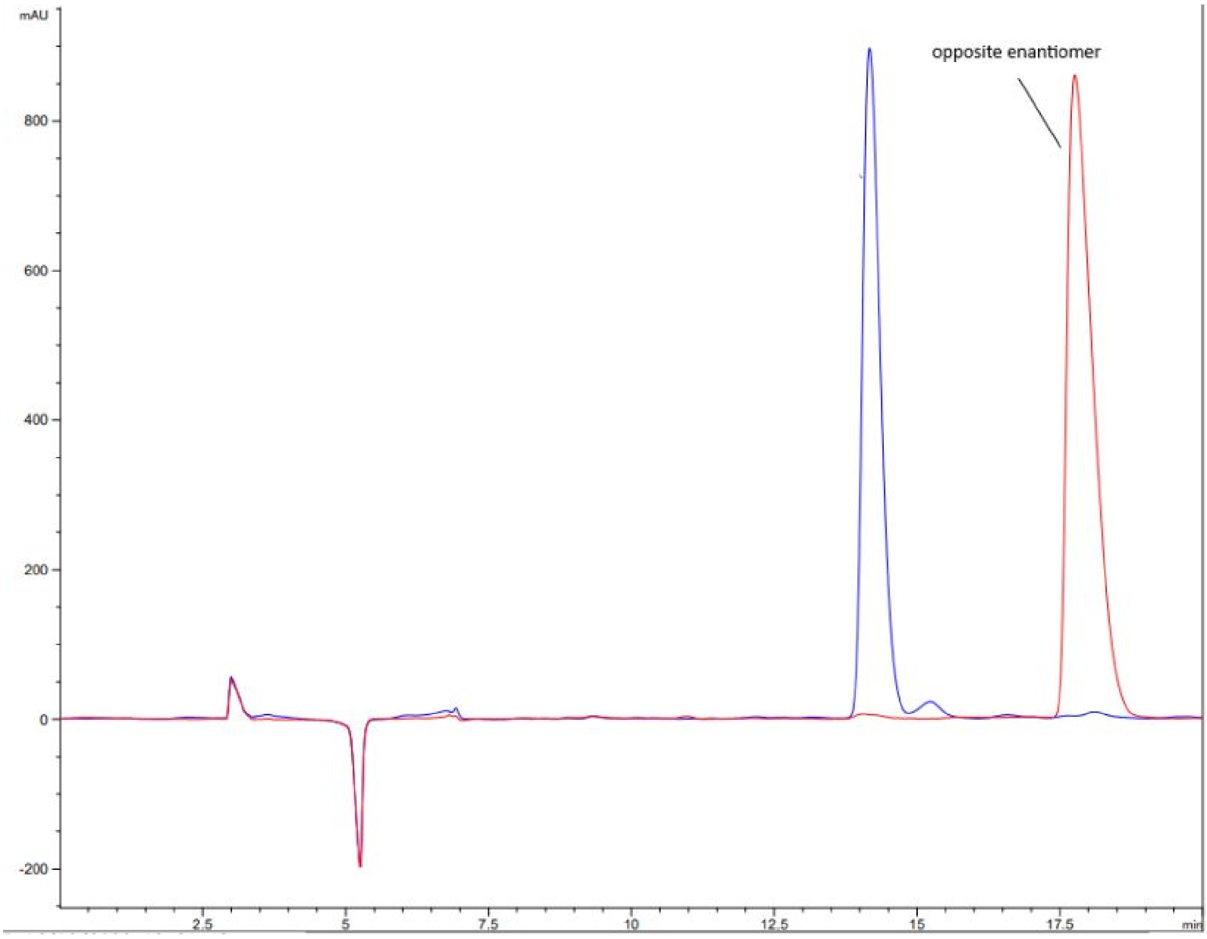

Synthesis of ((2*R*,6*R*)-6-(3-methoxyphenyl)tetrahydro-2*H*-pyran-2-yl)methanol, **3d**

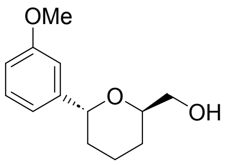

3d
$C_{13}H_{18}O_3$
MW 222.28

General Procedure D was used with (*m*-methoxyphenyl)boronic acid (133 mg, 0.88 mmol). Purification by automated column chromatography (0-100% ethyl acetate in heptane) afforded **3d** as a colourless oil (46 mg, 47%). ^{1}H NMR (400 MHz, $CDCl_3$, 30 °C, TMS): δ = 7.17-7.37 (m, 1H), 6.91 - 7.03 (m, 2H), 6.81 (d, *J* = 8.5 Hz, 1H), 4.88 (t, *J* = 5.1 Hz, 1H), 3.75 - 3.88 (m, 5H), 3.54 (br dd, ^{2}J = 10.5, *J* = 2.7 Hz, 1H), 1.89 - 2.10 (m, 3H), 1.57 - 1.77 (m, 3H), 1.45 - 1.57 ppm (m, 1H); ^{13}C NMR (101 MHz, $CDCl_3$, 30 °C, TMS): δ = 159.9, 143.2, 129.5, 118.8, 112.5, 112.3, 72.7, 71.9, 64.1, 55.2, 29.6, 26.3, 18.9 ppm; $[\alpha]_D^{20}$ = -30 (*c* 0.1 in $CHCl_3$); ν_{max} / cm^{-1} 3411, 2936, 2866, 1600, 1584, 1488, 1455, 1433, 1253, 1153, 1036, 788, 752, 694; HRMS (ESI) *m*/*z* calcd for $[M+Na]^+$ $C_{13}H_{18}O_3Na$: 245.1154; found: 245.1148.

Enantiomeric ratio by chiral HPLC (Chiralpak AD-H, 5 µm, 4.6 mm × 250 mm; 80% heptane/20% EtOH (+0.2% *iso*-propylamine), 1.0 mL min^{-1}, 215 nm; t_r (major) = 8.9 min), >99:1 e.r.

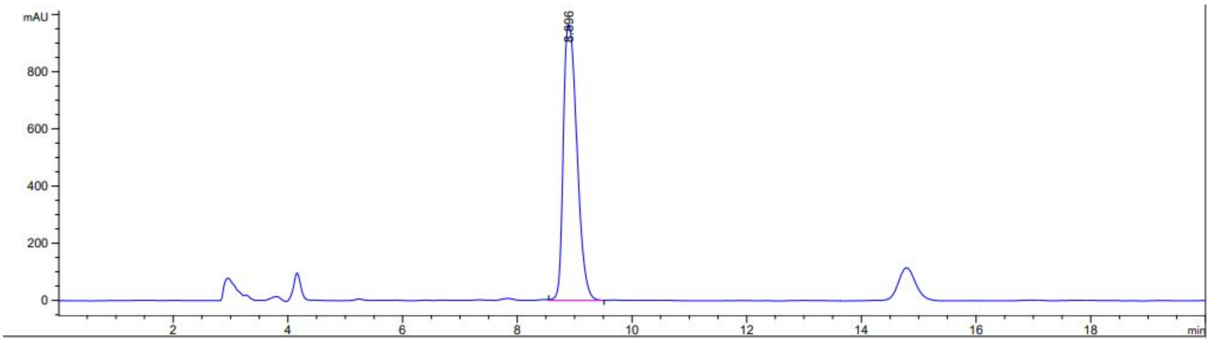

Peak #	RetTime [min]	Type	Width [min]	Area [mAU*s]	Height [mAU]	Area %
1	8.896	VV	0.2623	1.58929e4	964.25549	100.0000

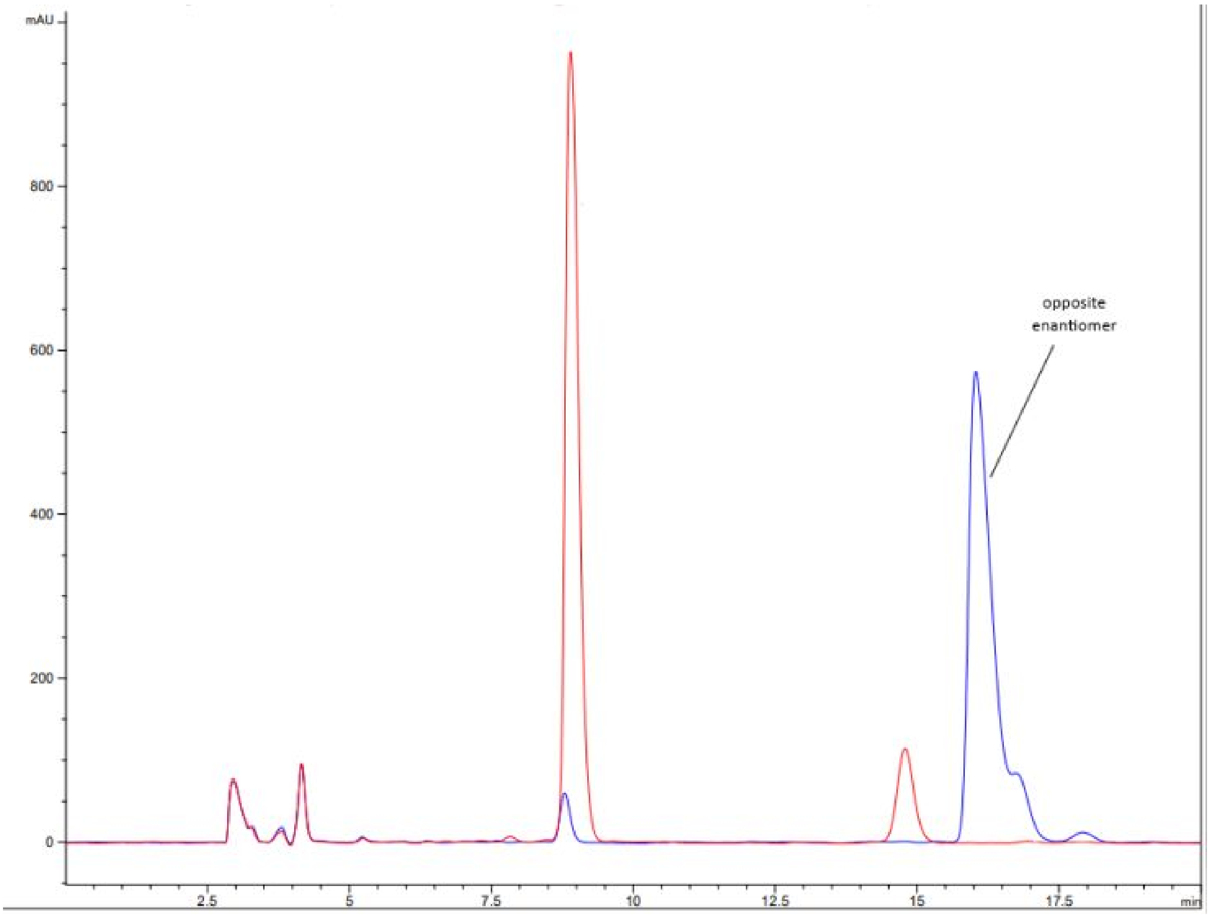

mAU
800
600
400
200
0
2.5
5
7.5
10
12.5
15
17.5
min
opposite enantiomer

Synthesis of ((2*R*,6*R*)-6-(2-methoxyphenyl)tetrahydro-2*H*-pyran-2-yl)methanol, **3e**

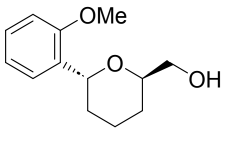

3e
$C_{13}H_{18}O_3$
MW 222.28

General Procedure D was used with (*o*-methoxyphenyl)boronic acid (133 mg, 0.88 mmol). Purification by automated column chromatography (0-100% ethyl acetate in cyclohexane) afforded **3e** as a colourless oil (24 mg, 24%). ^{1}H NMR (400 MHz, $CDCl_3$, 30 °C, TMS): δ = 7.42 (dd, *J* = 7.6, ^4J = 1.7 Hz, 1H), 7.22 - 7.31 (m, 1H), 6.98 (td, *J* = 7.6, ^4J = 1.0 Hz, 1H), 6.89 (dd, *J* = 8.3, ^4J = 1.0 Hz, 1H), 4.94 - 5.04 (m, 1H), 4.27 (dd, *J* = 10.5, ^2J = 10.5 1H), 4.04 - 4.18 (m, 1H), 3.86 (s, 3H), 3.43 (br d, ^2J = 10.5 Hz, 1H), 2.83 (br s, 1H), 2.00 (s, 1H), 1.73 - 1.93 (m, 4H), 1.41 - 1.65 ppm (m, 1H); ^{13}C NMR (101 MHz, $CDCl_3$, 30 °C, TMS): δ = 156.8, 130.0, 128.8, 126.6, 120.7, 110.5, 73.4, 65.5, 60.6, 55.4, 29.6, 24.9, 19.9 ppm; $[\alpha]_D^{19}$ = -52 (*c* 0.1 in $CHCl_3$); ν_{max} / cm^{-1} 3439, 2934, 1602, 1588, 1492, 1462, 1439, 1239, 1097, 1038, 1013, 753; HRMS (ESI) *m*/*z* calcd for $[M+Na]^+$ $C_{13}H_{18}O_3Na$: 245.1154; found: 245.1154.

Enantiomeric ratio by chiral HPLC (Chiralpak AD-H, 5 µm, 4.6 mm × 250 mm; 90% heptane/20% EtOH (+0.2% *iso*-propylamine), 1.0 mL min^{-1}, 215 nm; t_r (major) = 7.1 min, t_r (minor) = 10.8 min), 99:1 e.r.

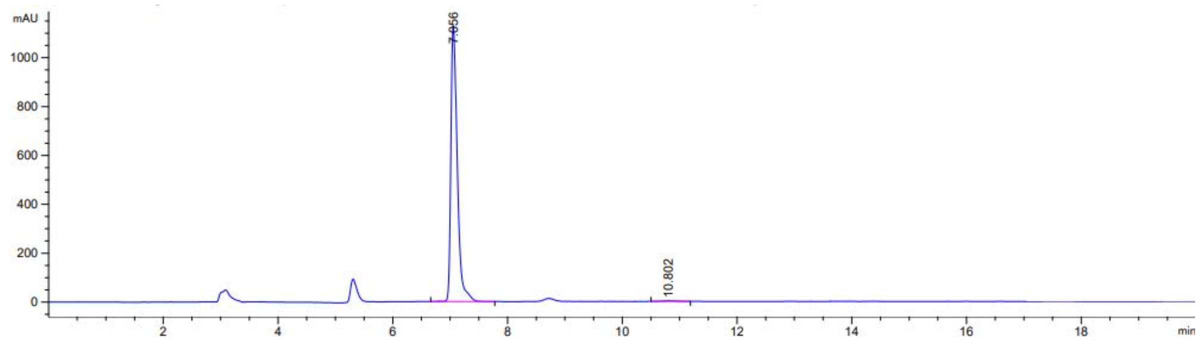

Peak #	RetTime [min]	Type	Width [min]	Area [mAU*s]	Height [mAU]	Area %
1	7.056	VB	0.1174	8655.55371	1130.15295	99.4873
2	10.802	BB	0.2309	44.60263	2.37755	0.5127

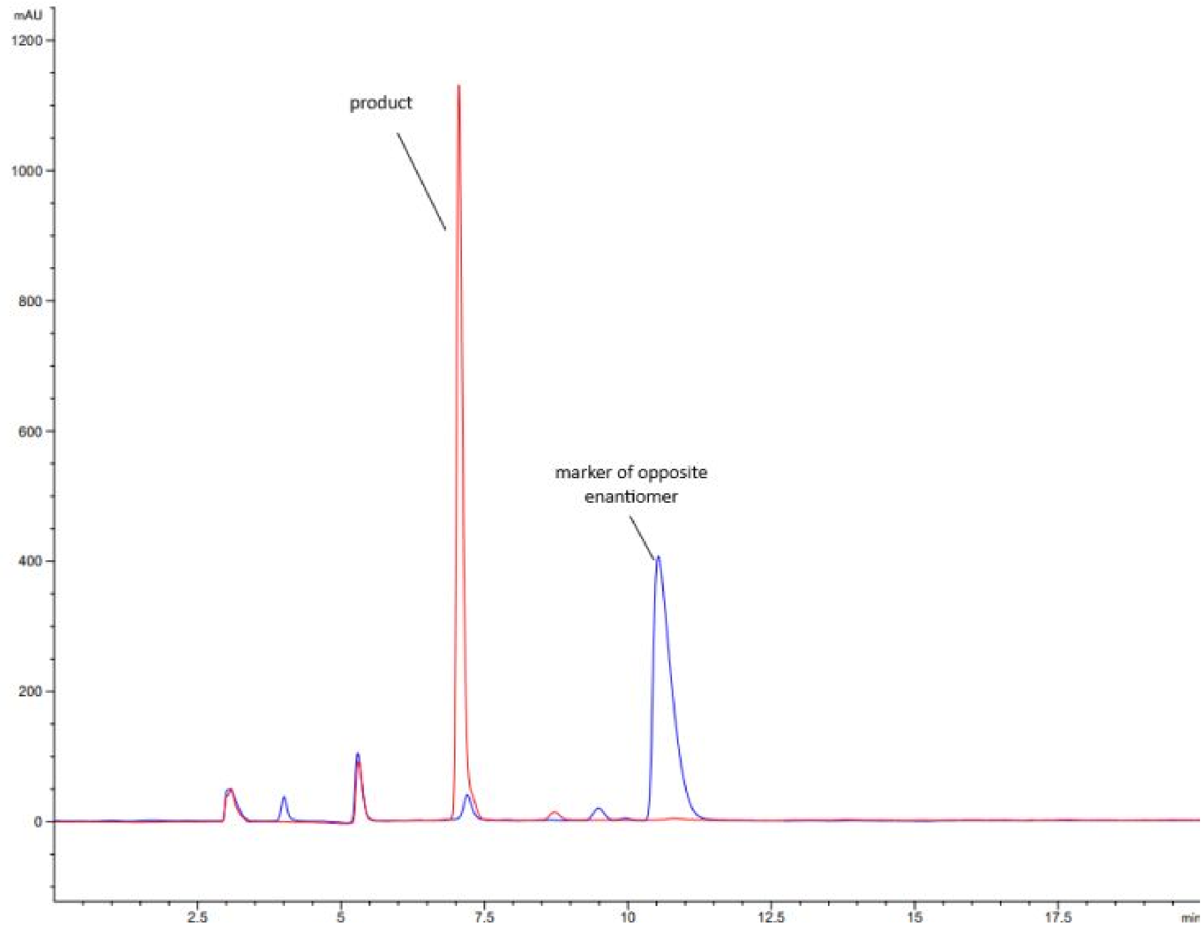

Synthesis of 4-((2*R*,6*R*)-6-(hydroxymethyl)tetrahydro-2*H*-pyran-2-yl)phenol, **3f**

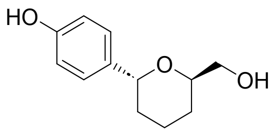

3f
$C_{12}H_{16}O_3$
MW 208.26

General Procedure D was used with (*p*-hydroxyphenyl)boronic acid (121 mg, 0.88 mmol). Purification by automated column chromatography (0-100% ethyl acetate in heptane) afforded **3f** as a berry red oil (39 mg, 43%). ^{1}H NMR (400 MHz, $CDCl_3$, 30 °C, TMS): δ = 7.19 - 7.32 (m, 2H), 6.81 (d, *J* = 7.5 Hz, 2H), 5.41 (br s, 1H), 4.86 (t, *J* = 4.9 Hz, 1H), 3.76 - 3.87 (m, 2H), 3.45 - 3.64 (m, 1H), 2.21 (br s, 1H), 1.87 - 2.08 (m, 2H), 1.57 - 1.75 (m, 3H), 1.42 - 1.56 ppm (m, 1H); ^{13}C NMR (101 MHz, $CDCl_3$, 25 °C, TMS): δ = 154.9, 133.1, 128.0 (2 × C), 115.2 (2 × C), 72.4, 71.7, 63.9, 29.4, 26.3, 18.8 ppm; $[\alpha]_D^{20}$ = -57 (*c* 0.1 in $CHCl_3$); ν_{max} / cm^{-1} 3232, 2938, 2867, 1614, 1597, 1515, 1455, 1247, 1225, 1033, 1015, 1005, 827, 815, 549, 517; HRMS (ESI) *m*/*z* calcd for $[M+Na]^+$ $C_{12}H_{16}O_3Na$: 231.0997; found: 231.0999.

Enantiomeric ratio by chiral HPLC (Chiralpak AD-H, 5 µm, 4.6 mm × 250 mm; 80% heptane/20% EtOH (+0.2% *iso*-propylamine), 1.0 mL min^{-1}, 215 nm; t_r (major) = 8.5 min, t_r (minor) = 13.9 min), 99:1 e.r.

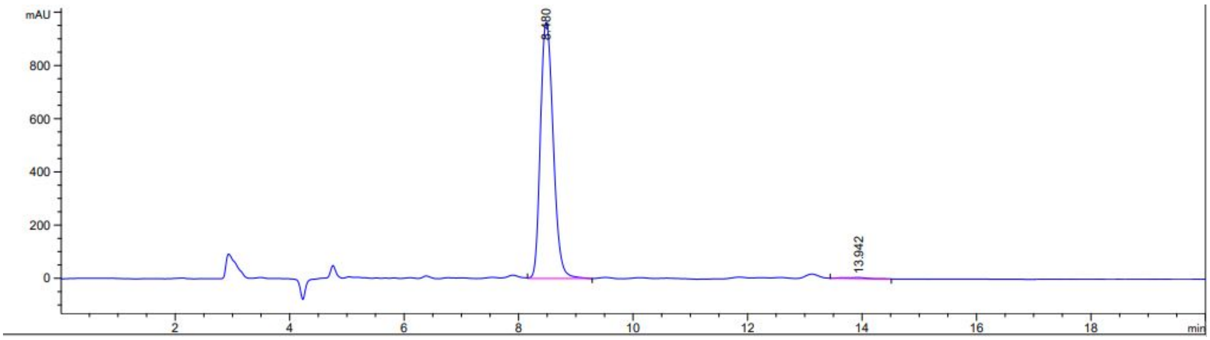

Peak #	RetTime [min]	Type	Width [min]	Area [mAU*s]	Height [mAU]	Area %
1	8.480	VB	0.2445	1.49073e4	963.29059	99.3076
2	13.942	BB	0.3265	103.93372	3.78361	0.6924

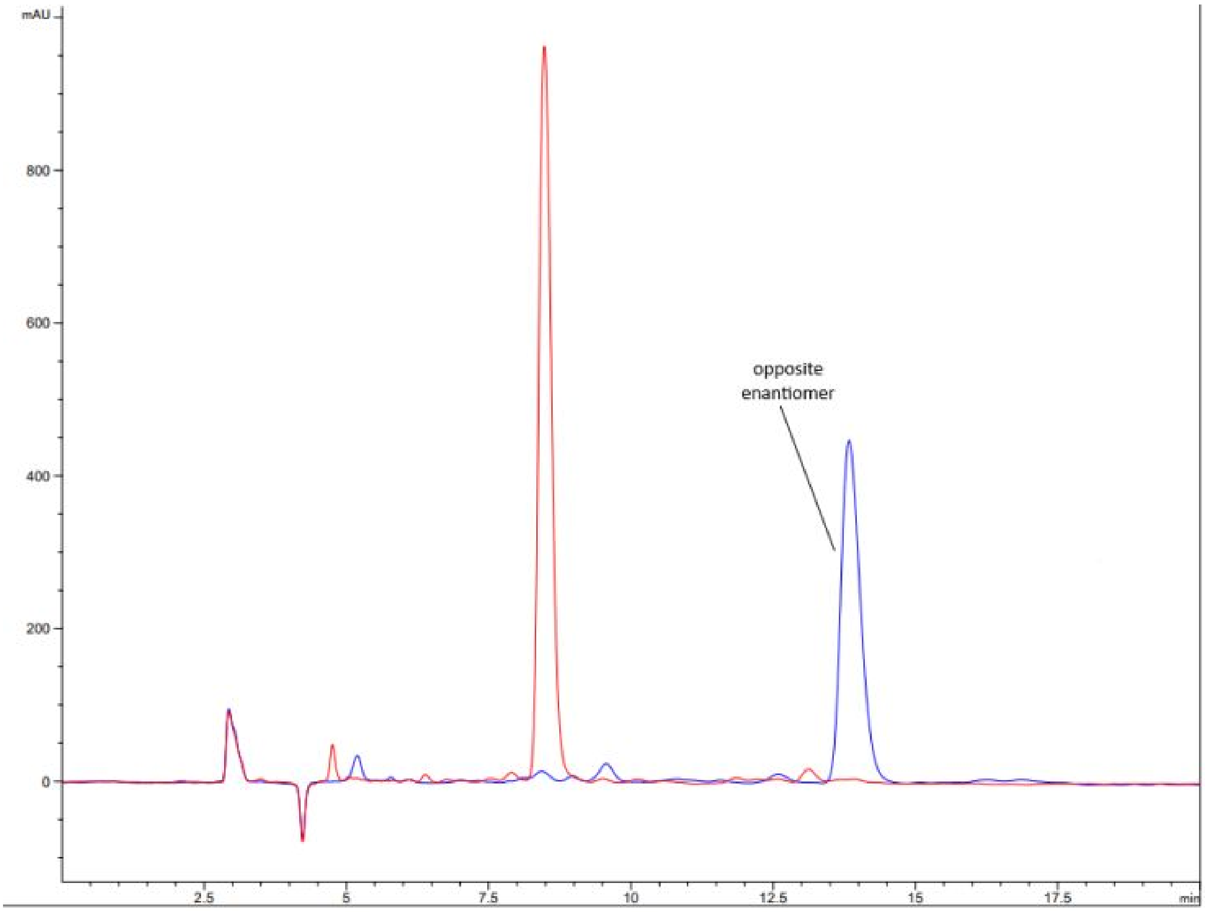

Synthesis of ((2R,6R)-6-(4-(benzyloxy)phenyl)tetrahydro-2*H*-pyran-2-yl)methanol, **3g**

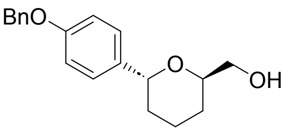

3g
$C_{19}H_{22}O_3$
MW 298.38

General Procedure D was used with (*p*-benzyloxyphenyl)boronic acid (200 mg, 0.88 mmol). Purification by automated column chromatography (0-100% ethyl acetate in heptane) afforded **3g** as a colourless oil (62 mg, 48%). ^{1}H NMR (400 MHz, $CDCl_3$, 30 °C, TMS): δ = 7.44 - 7.29 (m, 7H), 6.96 (d, *J* = 8.8 Hz, 2H), 5.05 (s, 2H), 4.85 (t, *J* = 5.1 Hz, 1H), 3.84 - 3.76 (m, 2H), 3.55 - 3.49 (m, 1H), 2.14 (br s, 1H), 2.02 - 1.86 (m, 2H), 1.73 - 1.57 (m, 3H), 1.52 - 1.43 (m, 1H); ^{13}C NMR (101 MHz, $CDCl_3$, 30 °C, TMS): δ = 157.9, 137.1, 133.8, 128.6 (2 × C), 127.9, 127.8 (2 × C), 127.4 (2 × C), 114.8 (2 × C), 72.3, 71.8, 70.1, 63.9, 29.5, 26.3, 18.9 ppm; $[\alpha]_D^{20}$ = -35 (*c* 0.1 in $CHCl_3$); ν_{max} / cm^{-1} 3417, 3034, 2935, 2866, 1610, 1583, 1509, 1239, 1035, 732, 696.

Enantiomeric ratio by chiral HPLC (Chiralpak AD-H, 5 µm, 4.6 mm × 250 mm; 60% heptane/40% EtOH (+0.2% *iso*-propylamine), 1.0 mL min^{-1}, 215 nm; t_r (major) = 8.0 min, t_r (minor) = 7.0 min), 98:2 e.r.

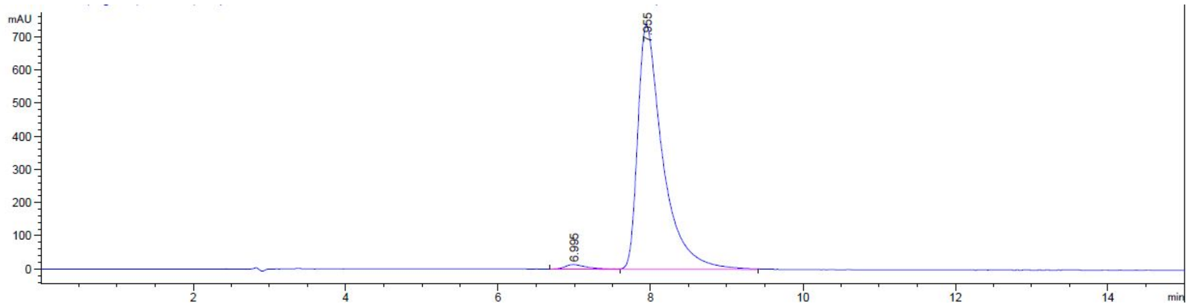

Peak #	RetTime [min]	Type	Width [min]	Area [mAU*s]	Height [mAU]	Area %
1	6.995	BV E	0.3013	268.04248	13.17581	1.5707
2	7.955	VB R	0.3362	1.67974e4	719.89423	98.4293

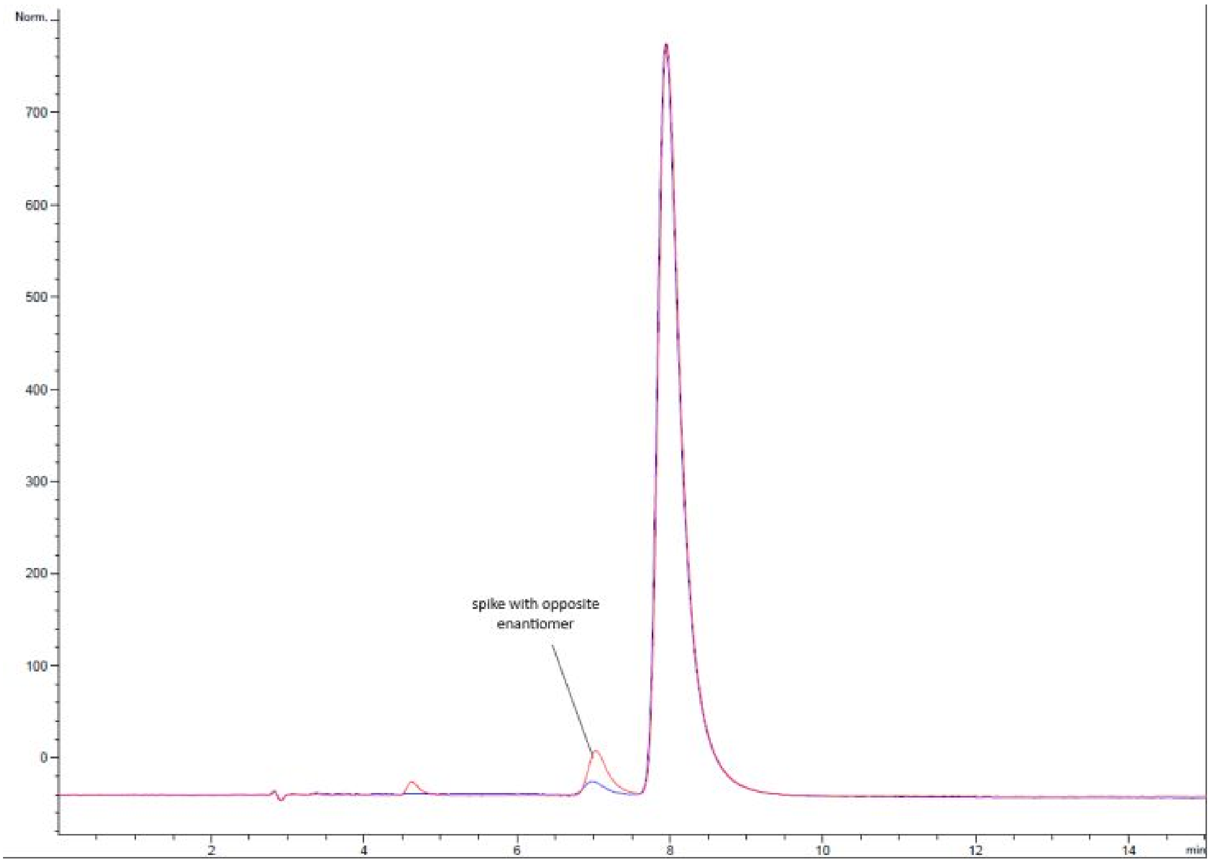

Synthesis of ((2R,6R)-6-(naphthalen-2-yl)tetrahydro-2*H*-pyran-2-yl)methanol, **3h**

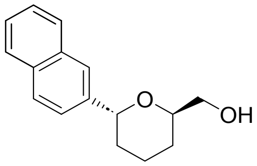

3h
$C_{16}H_{18}O_2$
MW 242.32

General Procedure D was used with naphthalen-2-ylboronic acid (151 mg, 0.88 mmol). Purification by automated column chromatography (0-100% ethyl acetate in heptane) afforded **3h** as a colourless oil (47 mg, 44%). ^{1}H NMR (400 MHz, $CDCl_3$, 30 °C, TMS): δ = 7.87 - 7.79 (m, 4H), 7.55 (d, *J* = 8.3 Hz, 1H), 7.49 - 7.43 (m, 2H), 5.07 (t, *J* = 5.1 Hz, 1H), 3.91 - 3.78 (m, 2H), 3.56 (br t, *J* = 7.8 Hz, 1H), 2.17 - 1.99 (m, 3H), 1.80 - 1.71 (m, 2H), 1.69 - 1.62 (m, 1H), 1.58 - 1.51 (m, 1H); ^{13}C NMR (101 MHz, $CDCl_3$, 30 °C, TMS): δ = 138.9, 133.4, 132.7, 128.3, 128.0, 127.6, 126.1, 125.8, 125.2, 124.8, 72.8, 72.1, 64.1, 29.6, 26.4, 19.0 ppm; $[\alpha]_D^{20}$ = -7 (*c* 0.1 in $CHCl_3$); ν_{max} / cm^{-1} 3396, 3055, 2935, 2866, 1633, 1600, 1506, 1036, 476.

Enantiomeric ratio by chiral HPLC (Chiralpak AS-H, 5 µm, 4.6 mm × 250 mm; 90% heptane/10% EtOH (+0.2% *iso*-propylamine), 1.0 mL min^{-1}, 215 nm; t_r (major) = 6.1 min, t_r (minor) = 7.2 min), >99:1 e.r.

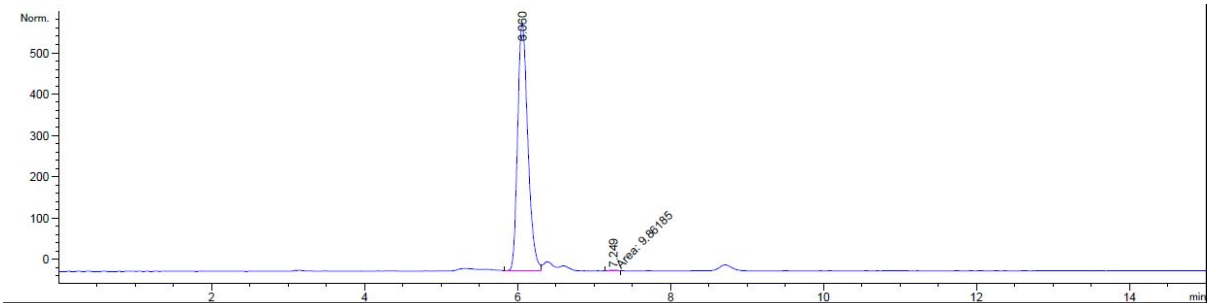

Peak #	RetTime [min]	Type	Width [min]	Area [mAU*s]	Height [mAU]	Area %
1	6.060	VV S	0.1448	5048.61914	541.23840	99.8050
2	7.249	MM	0.1332	9.86185	1.23383	0.1950

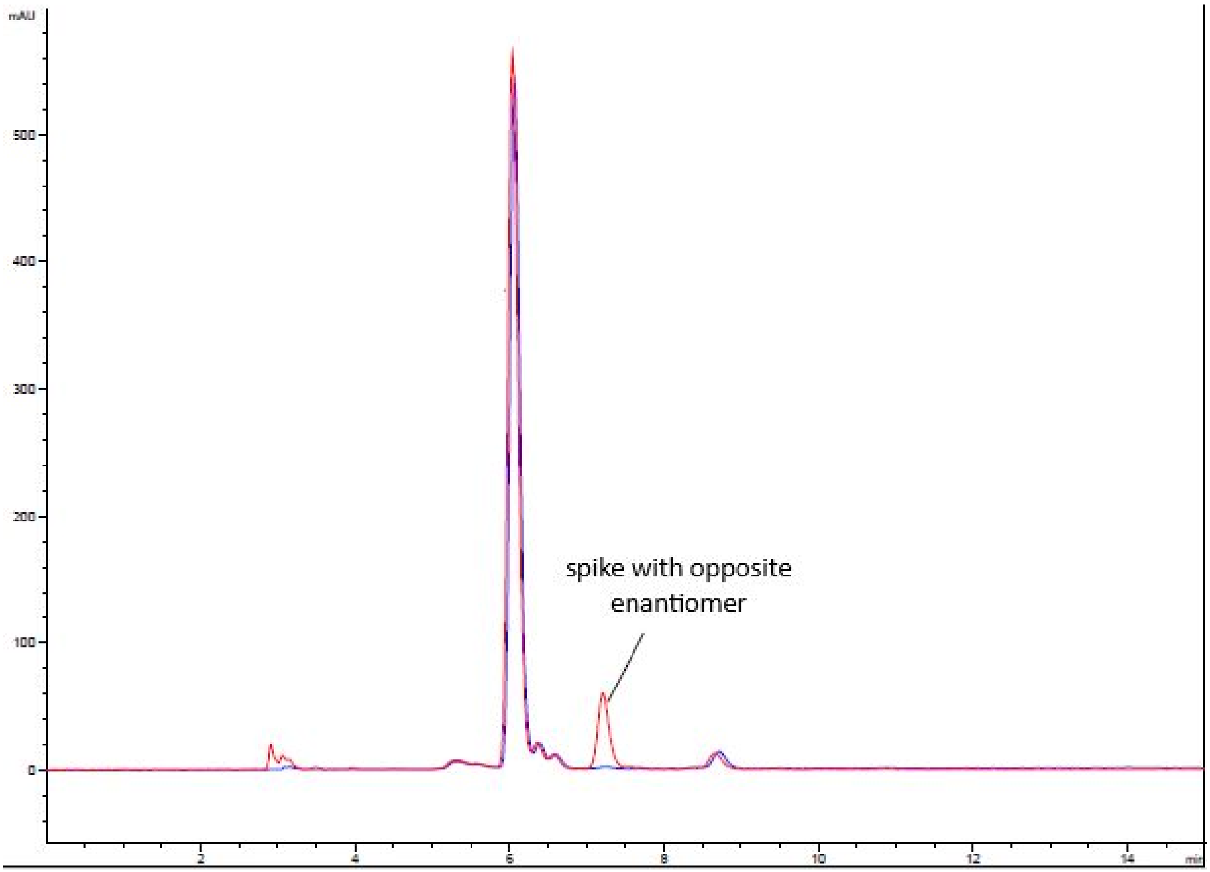

Synthesis of ((2*R*,6*R*)-6-(*p*-tolyl)tetrahydro-2*H*-pyran-2-yl)methanol, **3i**

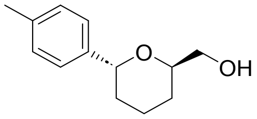

3i
$C_{13}H_{18}O_2$
MW 206.29

General Procedure D was used with (*p*-tolyl)boronic acid (119 mg, 0.88 mmol). Purification by automated column chromatography (0-100% ethyl acetate in heptane) afforded **3i** as a colourless oil (56 mg, 62%). ^{1}H NMR (400 MHz, $CDCl_3$, 30 °C, TMS): δ = 7.29 (d, *J* = 7.8 Hz, 2H), 7.17 (d, *J* = 7.8 Hz, 2H), 4.88 (t, *J* = 5.1 Hz, 1H), 3.70 - 3.92 (m, 2H), 3.44 - 3.66 (m, 1H), 2.34 (s, 3H), 1.82 - 2.08 (m, 3H), 1.56 - 1.77 (m, 3H), 1.42 - 1.52 ppm (m, 1H); ^{13}C NMR (101 MHz, $CDCl_3$, 30 °C, TMS): δ = 138.4, 136.7, 129.2 (2 × C), 126.5 (2 × C), 72.6, 71.7, 64.0, 29.5, 26.3, 21.0, 18.9 ppm; $[\alpha]_D^{20}$ = -42 (*c* 0.1 in $CHCl_3$); ν_{max} / cm^{-1} 3411, 2934, 2865, 1638, 1514, 1445, 1097, 1071, 1036, 810; HRMS (ESI) *m/z* calcd for $[M+Na]^+$ $C_{13}H_{18}O_2Na$: 229.1205; found: 229.1203.

Enantiomeric ratio by chiral HPLC (Chiralpak AD-H, 5 µm, 4.6 mm × 250 mm; 90% heptane/10% EtOH (+0.2% *iso*-propylamine), 1.0 mL min^{-1}, 215 nm; t_r (major) = 8.0 min, t_r (minor) = 9.0 min), >99:1 e.r.

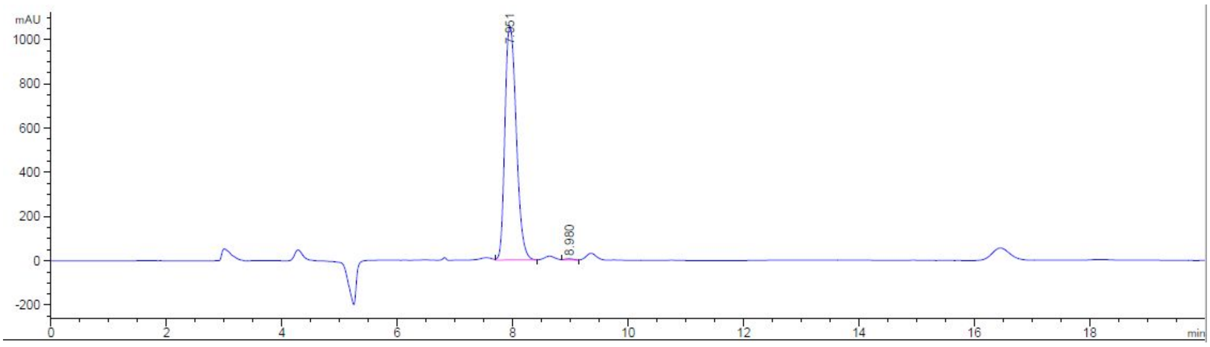

Peak #	RetTime [min]	Type	Width [min]	Area [mAU*s]	Height [mAU]	Area %
1	7.951	VB	0.2181	1.44914e4	1056.27832	99.6418
2	8.980	VV	0.1553	52.09341	5.00984	0.3582

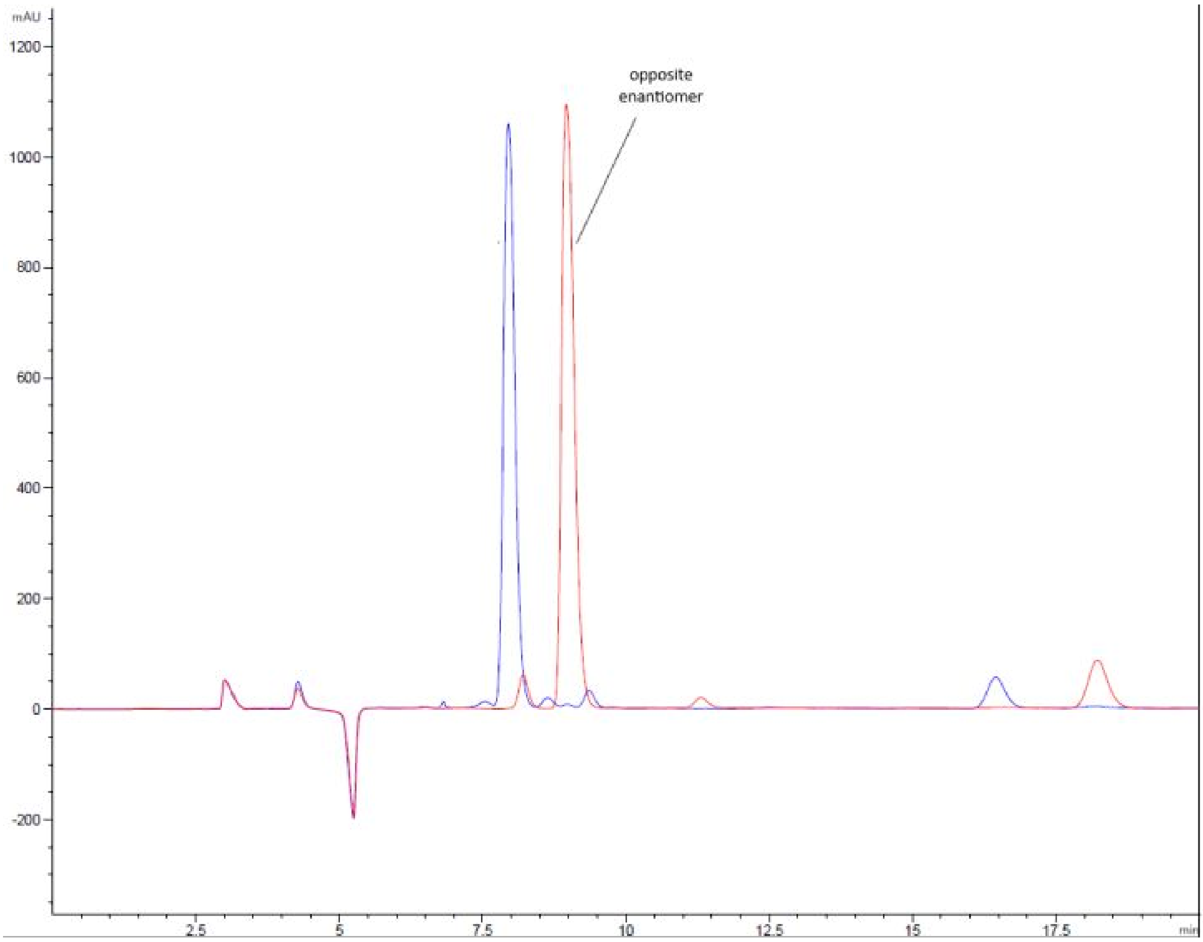

Synthesis of ((2*R*,6*R*)-6-(4-chlorophenyl)tetrahydro-2*H*-pyran-2-yl)methanol, **3j**

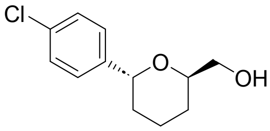

3j
$C_{12}H_{15}ClO_2$
MW 226.70

General Procedure D was used with (*p*-chlorophenyl)boronic acid (137 mg, 0.88 mmol). Purification by automated column chromatography (0-100% ethyl acetate in cyclohexane) afforded **3j** as a colourless oil (40 mg, 41%). ^{1}H NMR (400 MHz, $CDCl_3$, 30 °C, TMS): δ = 7.33 (s, 4H), 4.88 (t, J = 4.9 Hz, 1H), 3.75 - 3.85 (m, 2H), 3.50 - 3.59 (m, 1H), 1.89 - 2.01 (m, 2H), 1.58 - 1.77 (m, 3H), 1.45 - 1.56 ppm (m, 1H); ^{13}C NMR (101 MHz, $CDCl_3$, 30 °C, TMS): δ = 140.0, 132.9, 128.6 (2 × C), 127.9 (2 × C), 72.2, 72.0, 63.9, 29.6, 26.2, 18.8 ppm; $[\alpha]_D^{19}$ = -37 (c 0.1 in $CHCl_3$); ν_{max} / cm^{-1} 3401, 2937, 2866, 1903, 1597, 1490, 1445, 1401, 1091, 1037, 1012, 811; LC-MS or HRMS – no mass ion found.

HPLC separation (Chiralpak AD-H, 5 µm, 4.6 mm × 250 mm; 90% heptane/10% EtOH (+0.2% *iso*-propylamine), 1.0 mL min^{-1}, 215 nm; t_r (major) = 9.0 min, t_r (minor) = 9.8 min), >99:1 e.r.

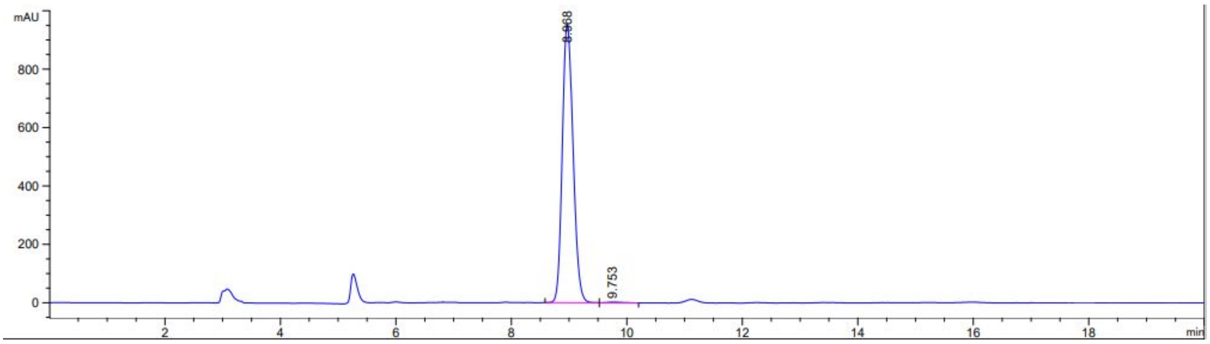

Peak #	RetTime [min]	Type	Width [min]	Area [mAU*s]	Height [mAU]	Area %
1	8.968	BV	0.2027	1.23043e4	952.72729	99.6082
2	9.753	VB	0.2302	48.40115	2.63664	0.3918

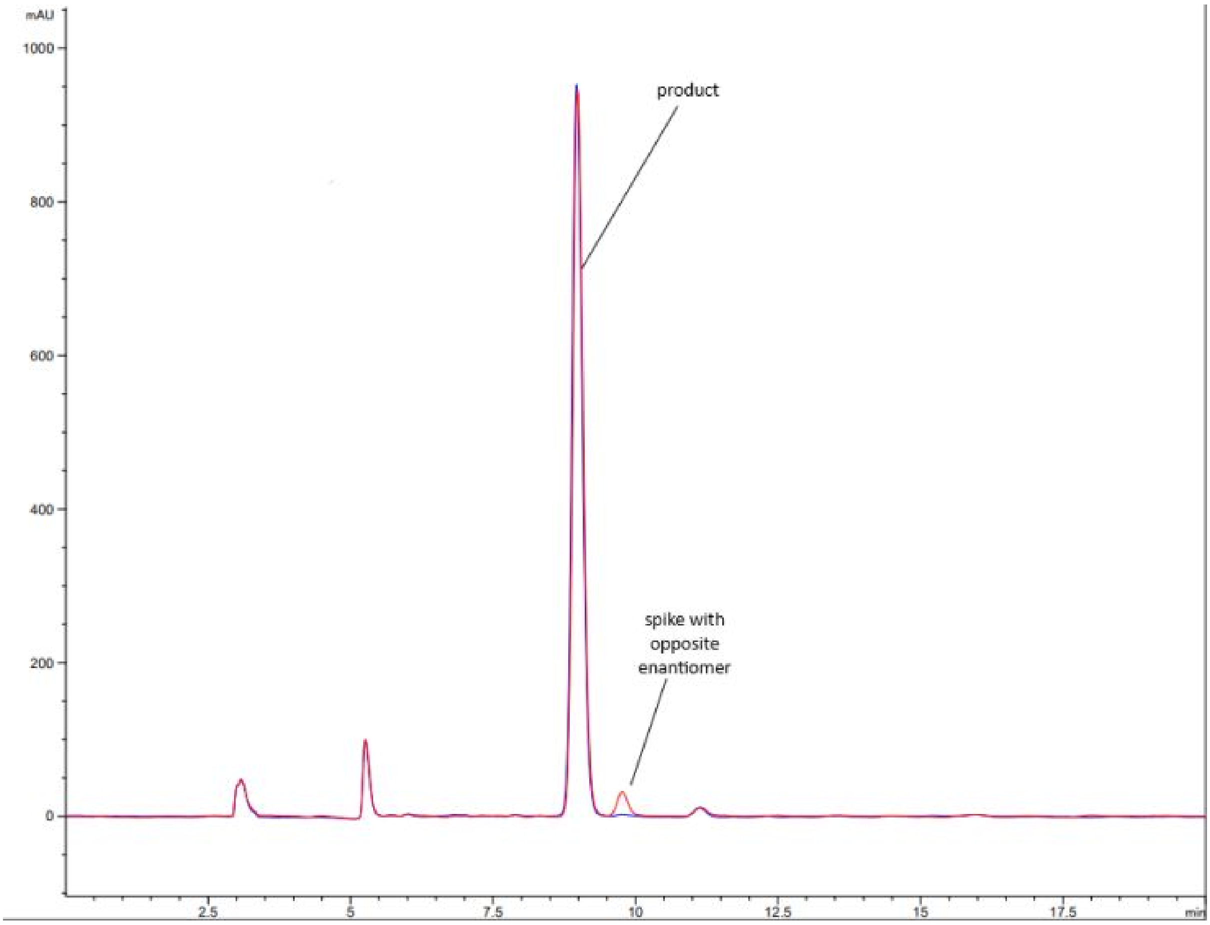

Synthesis of ((2*R*,6*R*)-6-(4-(trifluoromethyl)phenyl)tetrahydro-2*H*-pyran-2-yl)methanol, **3k**

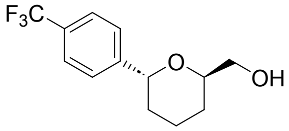

3k
$C_{13}H_{15}F_3O_2$
MW 260.26

Only trace aldehyde observed under the conditions in **General Procedure D**. Therefore the following procedure was used:

To a glass vial was added palladium(II) acetate (9.8 mg, 10 mol%), PyrOx **L1** (17.9 mg, 15 mol%), copper(II) trifluoromethanesulfonate (6.3 mg, 4 mol%), 6 × 3 Å molecular sieve beads (~250 mg) and anhydrous DMF (2.2 mL, 0.2 M). The resulting mixture was stirred (800 rpm) open to air at RT for 15 min. A solution of (*R*)-(3,4-dihydro-2*H*-pyran-2-yl)methanol (50.0 mg, 0.438 mmol, 1.0 eq.) and *p*-(trifluoromethyl)phenylboronic acid (166 mg, 0.88 mmol, 2.0 eq.) in anhydrous DMF (2.2 mL, 0.2 M) was then added in a single portion. Water (7.89 µL, 1.0 eq.) was then quickly added. The resulting mixture was then stirred (800 rpm) open to air at RT for 24 h. After this time, the reaction mixture was diluted with ethyl acetate (10 mL), quenched with 5% aqueous lithium chloride (5 mL), the layers separated and the organic phase washed with 5% aqueous lithium chloride (4 × 5 mL). The combined aqueous phases were back-extracted with ethyl acetate (15 mL), the combined organics dried (phase separator) and the solvent removed *in vacuo*. The crude material was dissolved in MeOH (3.5 mL), placed under an atmosphere of nitrogen and cooled to 0 °C. Sodium borohydride (166 mg, 10.0 eq.) was subsequently added and the resultant solution stirred at 0 °C for 3 h. After this time, ethyl acetate (4 mL) and saturated aqueous brine (4 mL) were added and the layers separated. The aqueous phase was extracted with ethyl acetate (3 × 4 mL), the combined organics dried (phase separator) and the solvent removed *in vacuo*. Purification by automated column chromatography (0-100% ethyl acetate in heptane) afforded **3k** as a colourless oil (29 mg, 25%). ^{1}H NMR (400 MHz, $CDCl_3$, 30 °C, TMS): δ = 7.62 (d, *J* = 8.6 Hz, 2H), 7.52 (d, *J* = 8.6 Hz, 2H), 4.95 (t, *J* = 5.1 Hz, 1H), 3.77 - 3.86 (m, 2H), 3. 35 - 3.61 (m, 1H), 1.91 - 2.02 (m, 3H), 1.60 - 1.79 ppm (m, 3H); ^{13}C NMR (101 MHz, $CDCl_3$, 25 °C, TMS): δ = 145.6, 129.3 (d, $^2J_{C\text{-}F}$ = 30.5 Hz), 126.8 (2 × C), 126.0, 125.4 (d,$^3J_{C\text{-}F}$ = 4.6 Hz, 2 × C), 72.3, 72.2, 63.9, 29.7, 26.2, 18.8 ppm; ^{19}F NMR (376 MHz, $CDCl_3$, 25 °C): δ = -62.48 ppm (s, 3F); $[\alpha]_D^{19}$ = -28 (*c* 0.1 in $CHCl_3$); ν_{max} / cm^{-1} 3358, 2940, 1678, 1618, 1516, 1412, 1322, 1161, 1117, 1065, 1040, 1016, 840, 821; LC-MS or HRMS – no mass ion found.

Enantiomeric ratio by chiral HPLC (Chiralcel OJ-H, 5 µm, 4.6 mm × 250 mm; 98% heptane/2% *iso*-propylalcohol (+0.2% *iso*-propylamine), 1.0 mL min^{-1}, 215 nm; tr (major) = 15.9 min), >99:1 e.r.

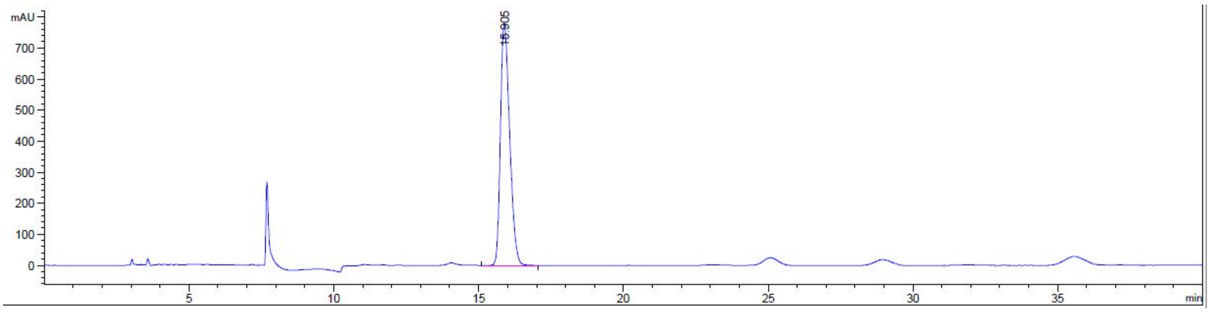

Peak #	RetTime [min]	Type	Width [min]	Area [mAU*s]	Height [mAU]	Area %
1	15.905	VBA	0.3615	1.72651e4	758.63446	100.0000

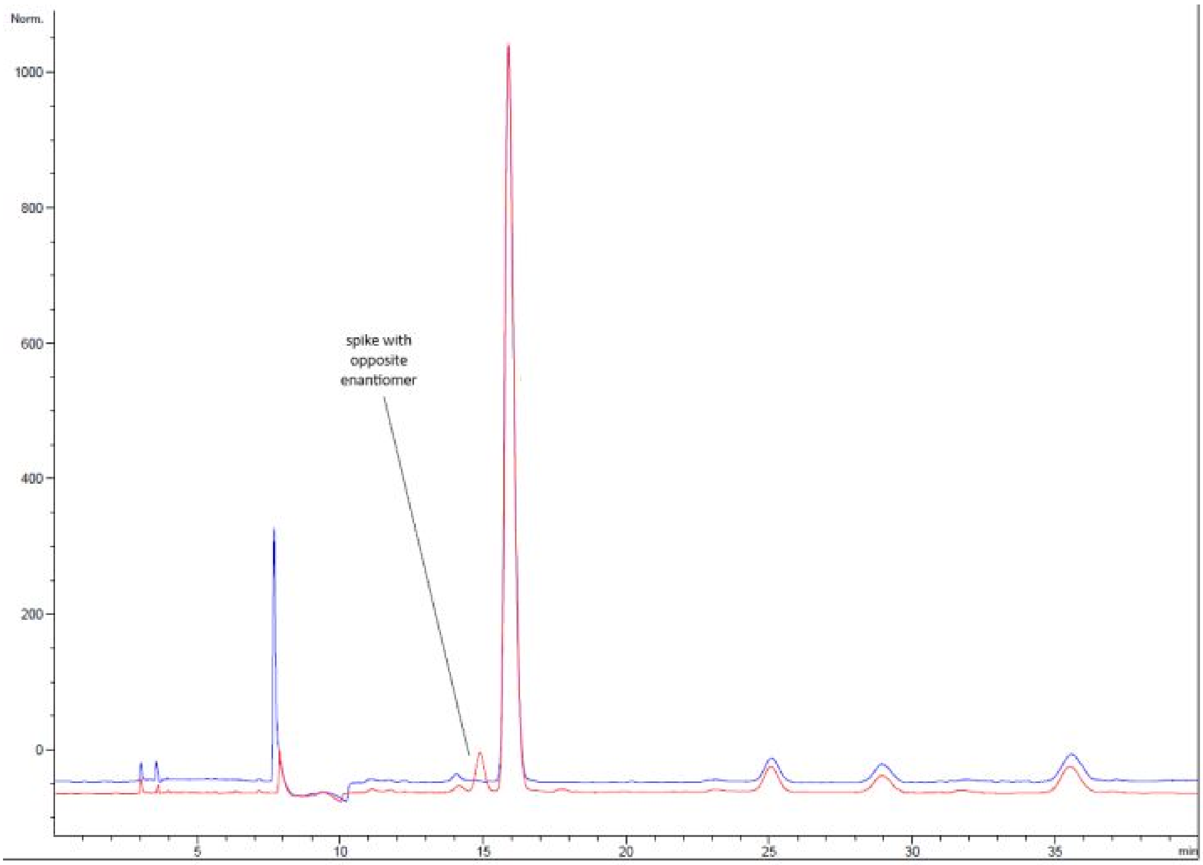

Synthesis of methyl 4-((2*R*,6*R*)-6-(hydroxymethyl)tetrahydro-2*H*-pyran-2-yl)benzoate, **3l**

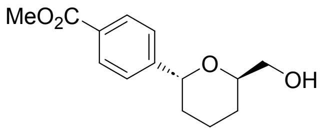

3l
$C_{14}H_{18}O_4$
MW 250.29

General Procedure D was used with (*p*-methoxycarbonylphenyl)boronic acid (158 mg, 0.88 mmol). Purification by automated column chromatography (0-100% ethyl acetate in cyclohexane) afforded **3l** as a colourless oil (17 mg, 16%). ^{1}H NMR (400 MHz, $CDCl_3$, 30 °C, TMS): δ = 8.03 (ddd, *J* = 8.0, ^4J = 2.0, ^4J = 2.0 Hz, 2H), 7.47 (d, *J* = 8.0 Hz, 2H), 4.94 (t, *J* = 5.1 Hz, 1H), 3.91 (s, 3H), 3.77 - 3.86 (m, 2H), 3.52 - 3.58 (m, 1H), 1.95 - 2.00 (m, 2H), 1.59 - 1.76 (m, 4H), 1.50 - 1.57 ppm (m, 1H); ^{13}C NMR (101 MHz, $CDCl_3$, 30 °C, TMS): δ = 167.0, 146.9, 129.8 (2 × C), 129.0, 126.4 (2 × C), 72.5, 72.2, 63.9, 52.1, 29.7, 26.2, 18.8 ppm; $[\alpha]_D^{19}$ = -26 (*c* 0.1 in $CHCl_3$); ν_{max} / cm^{-1} 3423, 2940, 2868, 1718, 1611, 1575, 1435, 1275, 1110, 1039, 1018, 735; LC-MS or HRMS – no mass ion found.

Enantiomeric ratio by chiral HPLC (Chiralpak AD-H, 5 µm, 4.6 mm × 250 mm; 80% heptane/20% EtOH (+0.2% *iso*-propylamine), 1.0 mL min^{-1}, 215 nm; t_r (major) = 19.1 min), >99:1 e.r.

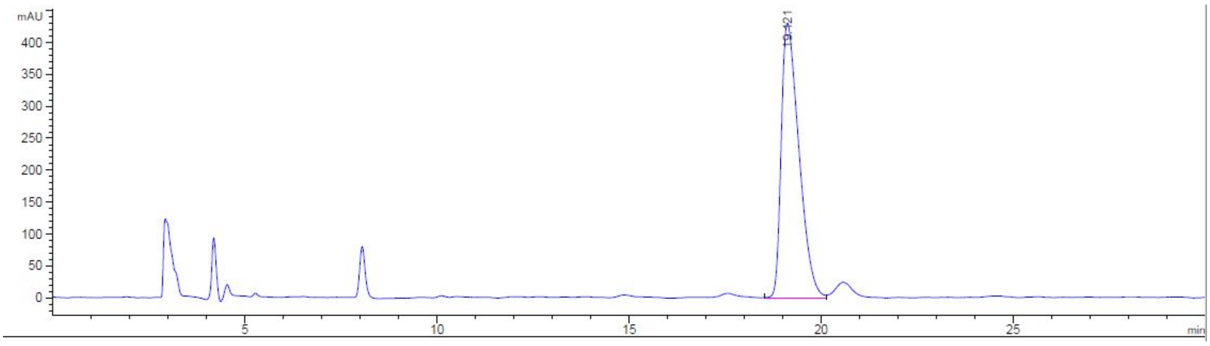

Peak #	RetTime [min]	Type	Width [min]	Area [mAU*s]	Height [mAU]	Area %
1	19.121	BV	0.4806	1.37442e4	430.37436	100.0000

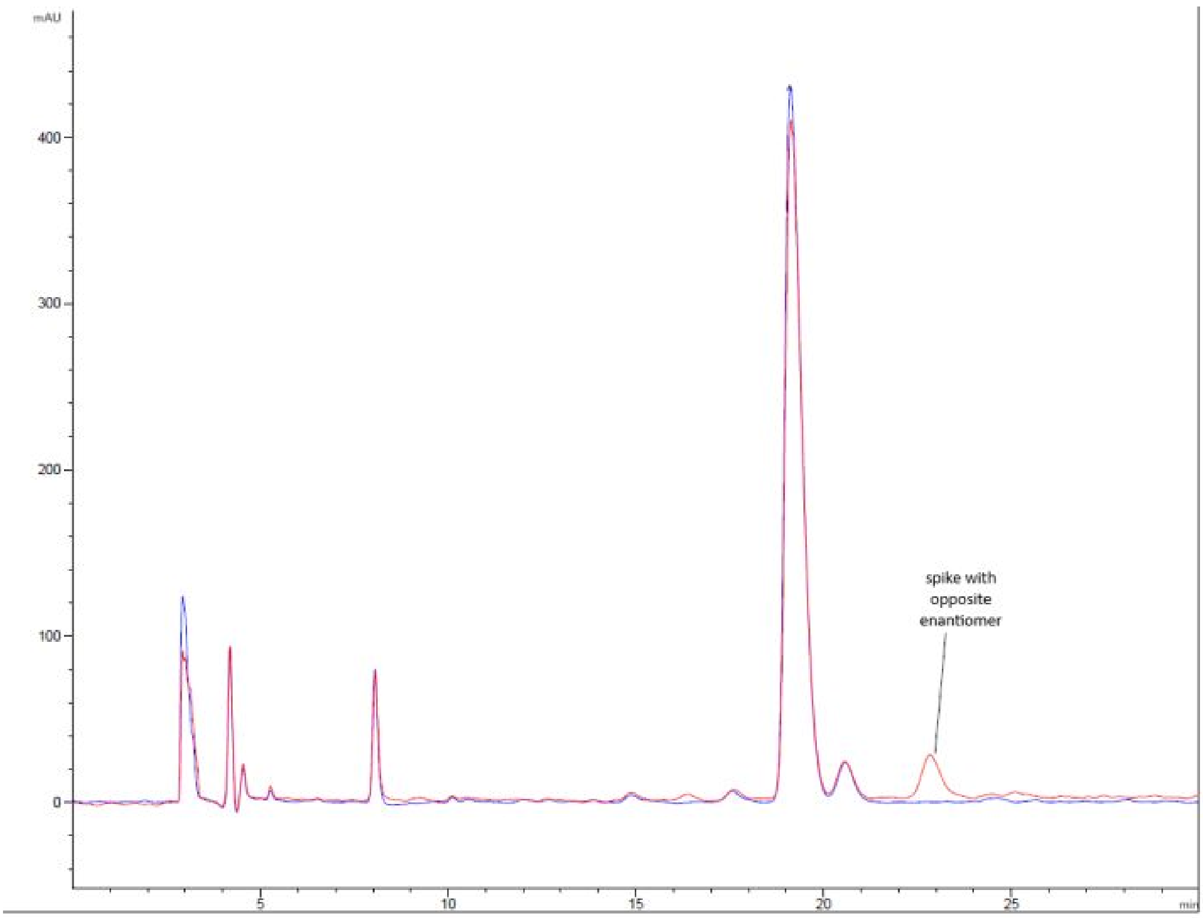

Synthesis of ((2*R*,6*R*)-6-(3-nitrophenyl)tetrahydro-2*H*-pyran-2-yl)methanol, **3m**

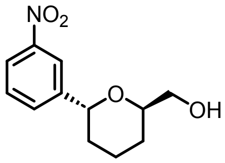

3m

$C_{12}H_{15}NO_4$

MW 237.26

Only trace aldehyde observed under the conditions in **General Procedure D**. Therefore the following procedure was used:

To a glass vial was added palladium(II) acetate (9.8 mg, 10 mol%), PyrOx **L1** (17.9 mg, 15 mol%), copper(II) trifluoromethanesulfonate (6.3 mg, 4 mol%), 6 × 3 Å molecular sieve beads (~250 mg) and anhydrous DMF (2.2 mL, 0.2 M). The resulting mixture was stirred (800 rpm) open to air at RT for 15 min. A solution of (*R*)-(3,4-dihydro-2*H*-pyran-2-yl)methanol (50.0 mg, 0.438 mmol, 1.0 eq.) and (*m*-nitrophenyl)boronic acid (146 mg, 0.88 mmol, 2.0 eq.) in anhydrous DMF (2.2 mL, 0.2 M) was then

added in a single portion. Water (7.89 µL, 1.0 eq.) was then quickly added. The resulting mixture was then stirred (800 rpm) open to air at RT for 24 h. After this time, the reaction mixture was diluted with ethyl acetate (10 mL), quenched with 5% aqueous lithium chloride (5 mL), the layers separated and the organic phase washed with 5% aqueous lithium chloride (4 × 5 mL). The combined aqueous phases were back-extracted with ethyl acetate (15 mL), the combined organics dried (phase separator) and the solvent removed *in vacuo*. The crude material was dissolved in MeOH (3.5 mL), placed under an atmosphere of nitrogen and cooled to 0 °C. Sodium borohydride (166 mg, 10.0 eq.) was subsequently added and the resultant solution stirred at 0 °C for 3 h. After this time, ethyl acetate (4 mL) and saturated aqueous brine (4 mL) were added and the layers separated. The aqueous phase was extracted with ethyl acetate (3 × 4 mL), the combined organics dried (phase separator) and the solvent removed *in vacuo*. Purification by automated reverse phase column chromatography (formic method, 5-95% 0.1% formic acid in MeCN in 0.1% formic acid in water) afforded **3m** as a colourless oil (18 mg, 17%). ^{1}H NMR (400 MHz, $CDCl_3$, 30 °C, TMS): δ = 7.14 (t, J = 7.8 Hz, 1H), 6.86 - 6.68 (m, 2H), 6.68 - 6.52 (m, 1H), 4.81 (t, J = 5.1 Hz, 1H), 3.91 - 3.84 (m, 1H), 3.84 - 3.78 (m, 1H), 3.55 - 3.50 (m, 1H), 2.07 - 1.80 (m, 2H), 1.77 - 1.57 (m, 3H), 1.49 ppm (m, 1H); ^{13}C NMR (101 MHz, $CDCl_3$, 30 °C, TMS): δ = 146.7, 142.8, 129.4, 116.7, 113.9, 113.2, 72.7, 72.0, 63.9, 29.7, 26.3, 19.0 ppm; $[\alpha]_D^{21}$ = -21 (c 0.1 in $CHCl_3$); ν_{max} / cm^{-1} 3350, 2936, 2866, 2242, 1604, 1458, 1035, 728, 696.

Enantiomeric ratio by chiral HPLC (Chiralcel OJ-H, 5 µm, 4.6 mm × 250 mm; 40% heptane/60% EtOH (+0.2% *iso*-propylamine), 1.0 mL min^{-1}, 215 nm; t_r (major) = 8.0 min), >99:1 e.r.

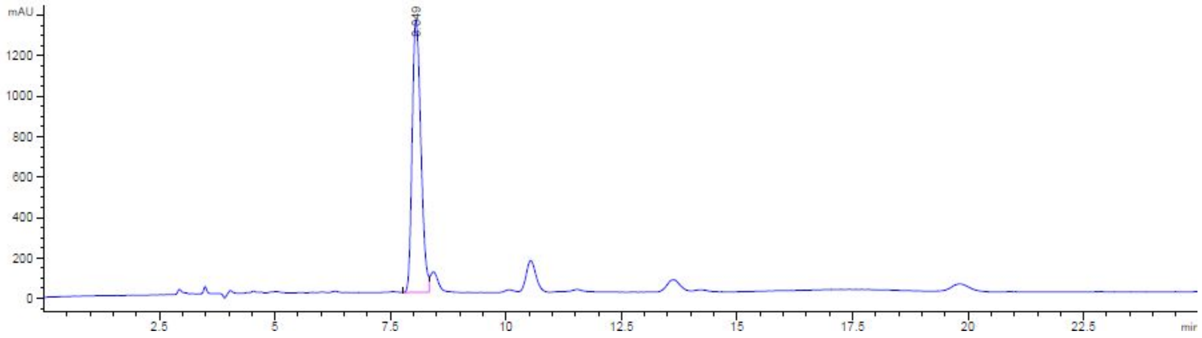

Peak #	RetTime [min]	Type	Width [min]	Area [mAU*s]	Height [mAU]	Area %
1	8.049	BV	0.2091	1.77276e4	1350.46448	100.0000

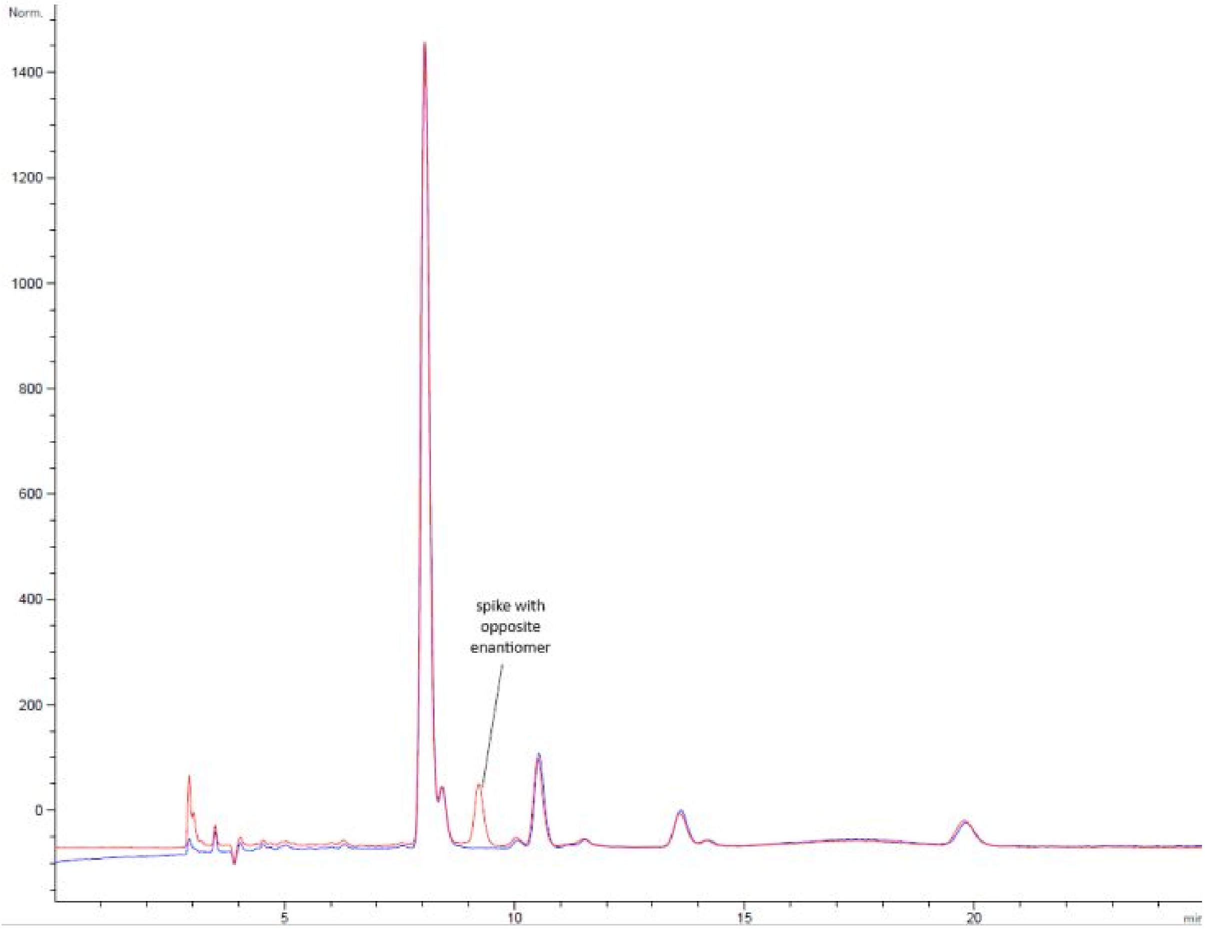

Synthesis of ((2*R*,6*R*)-6-(4-bromophenyl)tetrahydro-2*H*-pyran-2-yl)methanol, **3n**

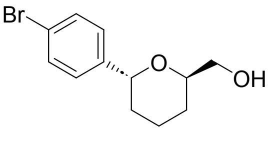

3n
$C_{12}H_{15}BrO_2$
MW 271.15

General Procedure D was used with (*p*-bromophenyl)boronic acid (176 mg, 0.88 mmol). Purification by automated reverse phase column chromatography (formic method, 5-95% MeCN in water (1% formic acid)) afforded **3n** as a pale yellow oil (10 mg, 9%). ^{1}H NMR (400 MHz, $CDCl_3$, 30 °C, TMS): δ = 7.46 - 7.50 (m, 2H), 7.24 - 7.29 (m, 2H), 4.86 (t, *J* = 5.1 Hz, 1H), 3.76 - 3.85 (m, 2H), 3.50 - 3.58 (m, 1H), 1.89 - 2.00 (m, 3H), 1.58 - 1.75 (m, 3H), 1.45 - 1.56 ppm (m, 1H); ^{13}C NMR (101 MHz, $CDCl_3$, 30 °C, TMS): δ = 140.5, 131.6 (2 × C), 128.3 (2 × C), 121.0, 72.2, 72.0, 64.0, 29.5, 26.2, 18.8 ppm; $[\alpha]_D^{20}$ = +3 (*c* 0.1 in $CHCl_3$); ν_{max} / cm^{-1} 3370, 2936, 1584, 1558, 1487, 1410, 1317, 1270, 1100, 1068, 1039, 1009, 814, 723; LC-MS or HRMS – no mass ion found.

Enantiomeric ratio by chiral HPLC (Chiralpak AD-H, 5 µm, 4.6 mm × 250 mm; 90% heptane/10% EtOH (+0.2% *iso*-propylamine), 1.0 mL min^{-1}, 215 nm; t_r (major) = 10.5 min), >99:1 e.r.

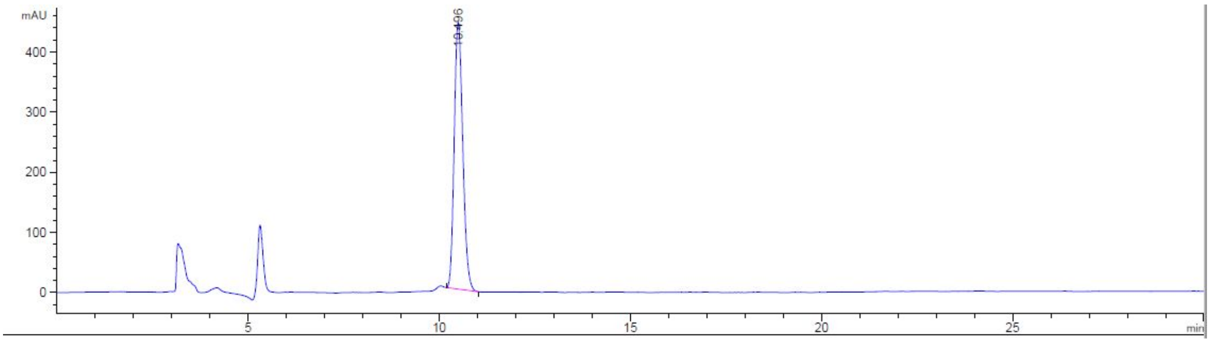

Peak #	RetTime [min]	Type	Width [min]	Area [mAU*s]	Height [mAU]	Area %
1	10.496	BBA	0.2413	6898.77979	443.83951	100.0000

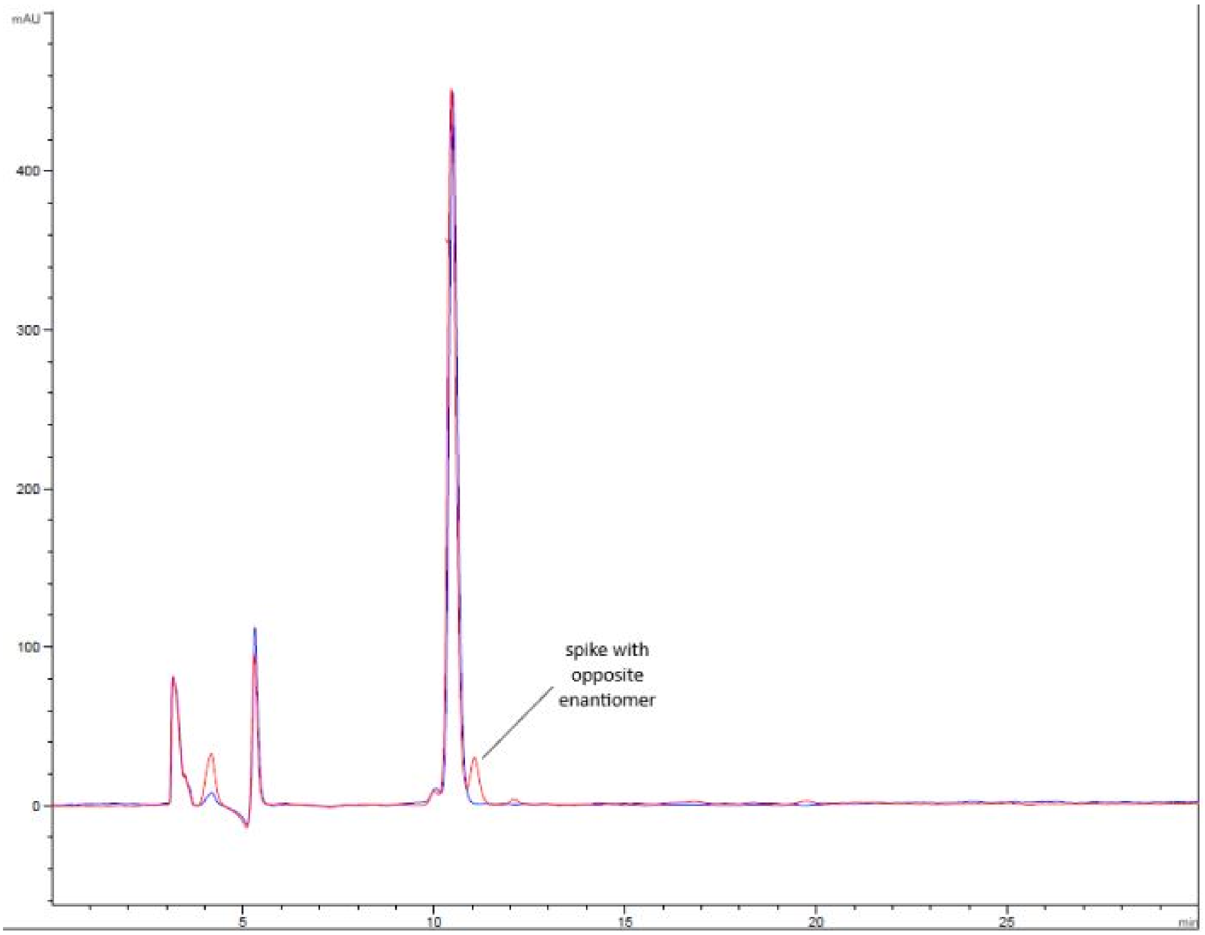

Synthesis of ((2*R*,6*R*)-6-(furan-2-yl)tetrahydro-2*H*-pyran-2-yl)methanol, **3o**

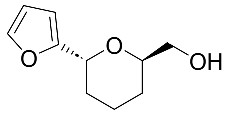

3o
$C_{10}H_{14}O_3$
MW 182.22

Only trace aldehyde observed under the conditions in **General Procedure D**. Therefore the following procedure was used:

To a glass vial was added palladium(II) acetate (9.8 mg, 10 mol%), PyrOx **L1** (17.9 mg, 15 mol%), copper(II) trifluoromethanesulfonate (6.3 mg, 4 mol%), 6 × 3 Å molecular sieve beads (~250 mg) and anhydrous DMF (2.2 mL, 0.2 M). The resulting mixture was stirred (800 rpm) open to air at RT for 15 min. A solution of (*R*)-(3,4-dihydro-2*H*-pyran-2-yl)methanol (50.0 mg, 0.438 mmol, 1.0 eq.) and furan-2-ylboronic acid (98.0 mg, 0.88 mmol, 2.0 eq.) in anhydrous DMF (2.2 mL, 0.2 M) was then added in a single portion. Water (7.89 µL, 1.0 eq.) was then quickly added. The resulting mixture was then stirred (800 rpm) open to air at RT for 24 h. After this time, the reaction mixture was diluted with ethyl acetate (10 mL), quenched with 5% aqueous lithium chloride (5 mL), the layers separated and the organic phase washed with 5% aqueous lithium chloride (4 × 5 mL). The combined aqueous phases were back-extracted with ethyl acetate (15 mL), the combined organics dried (phase separator) and the solvent removed *in vacuo*. The crude material was dissolved in MeOH (3.5 mL), placed under an atmosphere of nitrogen and cooled to 0 °C. Sodium borohydride (166 mg, 10.0 eq.) was subsequently added and the resultant solution stirred at 0 °C for 3 h. After this time, ethyl acetate (4 mL) and saturated aqueous brine (4 mL) were added and the layers separated. The aqueous phase was extracted with ethyl acetate (3 × 4 mL), the combined organics dried (phase separator) and the solvent removed *in vacuo*. Purification by automated column chromatography (0-100% ethyl acetate in heptane) afforded **3o** as a colourless oil (17 mg, 21%). ^{1}H NMR (400 MHz, $CDCl_3$, 30 °C, TMS): δ = 7.40 (d, *J* = 2.0 Hz, 1H), 6.35 (dd, *J* = 3.4, 2.0 Hz, 1H), 6.26 - 6.31 (m, 1H), 5.01 (dd, *J* = 5.6, 2.7 Hz, 1H), 3.52 - 3.74 (m, 3H), 1.89 - 2.15 (m, 3H), 1.69 - 1.86 (m, 2H), 1.39 - 1.66 ppm (m, 2H); ^{13}C NMR (101 MHz, $CDCl_3$, 30 °C, TMS): δ = 154.2, 142.0, 110.1, 107.8, 71.9, 68.7, 65.6, 26.9, 26.7, 19.1 ppm; $[\alpha]_D^{21}$ = -31 (*c* 0.1 in $CHCl_3$); ν_{max} / cm^{-1} 1414, 2938, 2870, 1604, 1501, 1037, 1013, 737, 599.

Enantiomeric ratio by chiral HPLC (Chiralpak AD-H, 5 µm, 4.6 mm × 250 mm; 90% heptane/10% EtOH (+0.2% *iso*-propylamine), 1.0 mL min^{-1}, 215 nm; t_r (major) = 8.9 min, t_r (minor) = 11.3 min), 93:7 e.r.

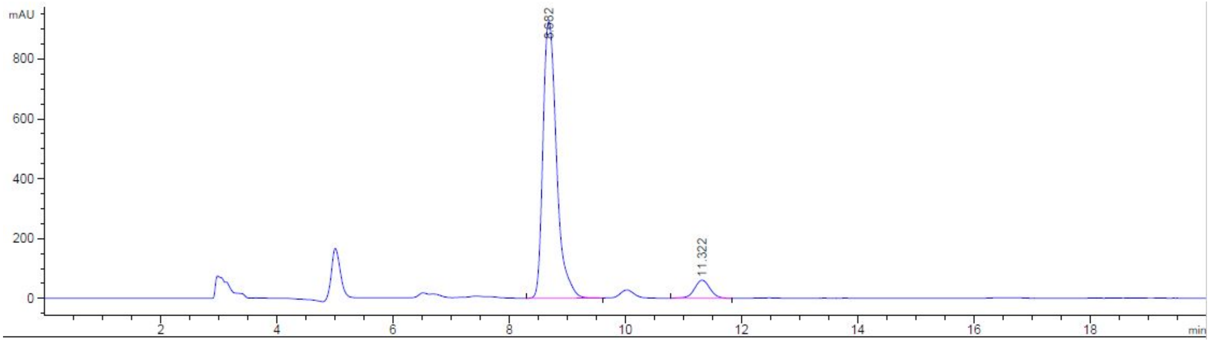

Peak #	RetTime [min]	Type	Width [min]	Area [mAU*s]	Height [mAU]	Area %
1	8.682	BB	0.2542	1.50624e4	923.91779	93.0374
2	11.322	BB	0.2916	1127.21472	60.42573	6.9626

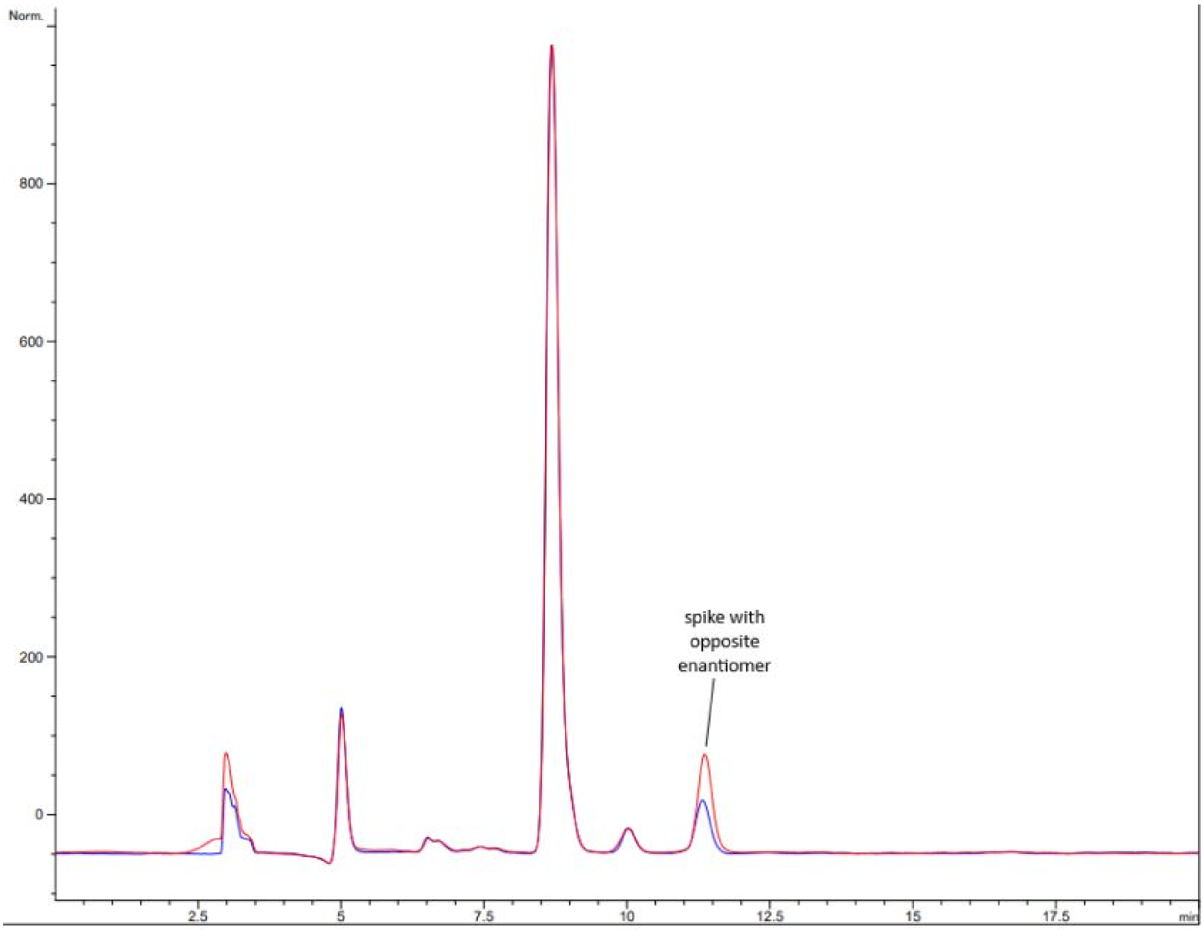

Synthesis of ((2*R*,6*R*)-6-(furan-3-yl)tetrahydro-2*H*-pyran-2-yl)methanol, **3p**

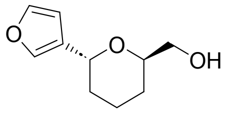

3p
$C_{10}H_{14}O_3$
MW 182.22

General Procedure D was used with (3-furanyl)boronic acid (98.0 mg, 0.88 mmol). Purification by automated reverse phase column chromatography (high pH method, 0-100% MeCN in 10 mM ammonium bicarbonate at pH 10) afforded **3p** as a colourless oil (31 mg, 39%). ^{1}H NMR (400 MHz, $CDCl_3$, 30 °C, TMS): δ = 7.41 (t, *J* = 1.5 Hz, 1H), 7.33 (dd, *J* = 2.9, 1.5 Hz, 1H), 6.37 - 6.40 (m, 1H), 4.98 (t, *J* = 3.9 Hz, 1H), 3.68 - 3.76 (m, 1H), 3.58 - 3.63 (m, 1H), 3.50 - 3.55 (m, 1H), 1.84 - 2.00 (m, 3H), 1.67 - 1.76 (m, 2H), 1.49 - 1.55 (m, 1H), 1.37 - 1.47 ppm (m, 1H); ^{13}C NMR (101 MHz, $CDCl_3$, 30 °C, TMS): δ = 143.3, 139.7, 125.7, 109.8, 71.2, 67.9, 65.4, 28.6, 26.8, 18.7 ppm; $[\alpha]_D^{21}$ = -5 (*c* 0.1 in $CHCl_3$); ν_{max} / cm^{-1} 3410, 2937, 2868, 1598, 1502, 1035, 1021, 874, 796, 599.

HPLC separation (Chiralpak AD-H, 5 µm, 4.6 mm × 250 mm; 90% heptane/10% EtOH (+0.2% *iso*-propylamine), 1.0 mL min^{-1}, 215 nm; t_r (major) = 10.0 min, t_r (minor) = 11.5 min), 99:1 e.r.

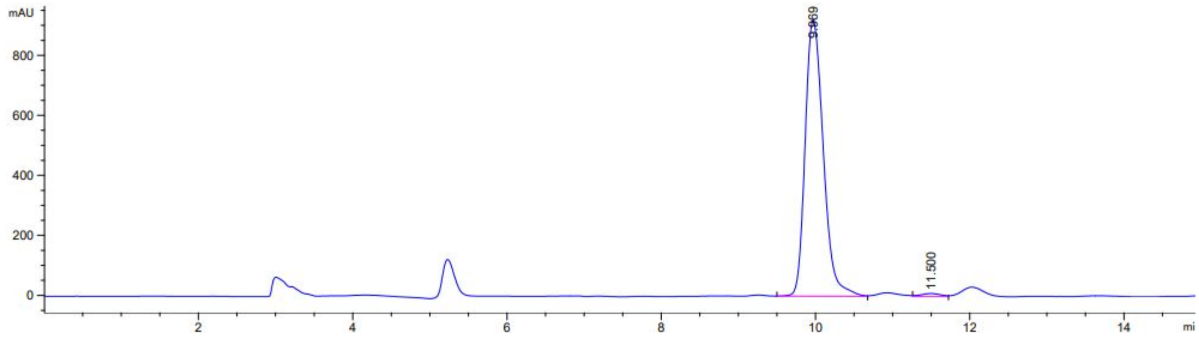

Peak #	RetTime [min]	Type	Width [min]	Area [mAU*s]	Height [mAU]	Area %
1	9.969	BV	0.2573	1.50875e4	920.32849	98.8214
2	11.500	VV	0.2721	179.94308	9.99412	1.1786

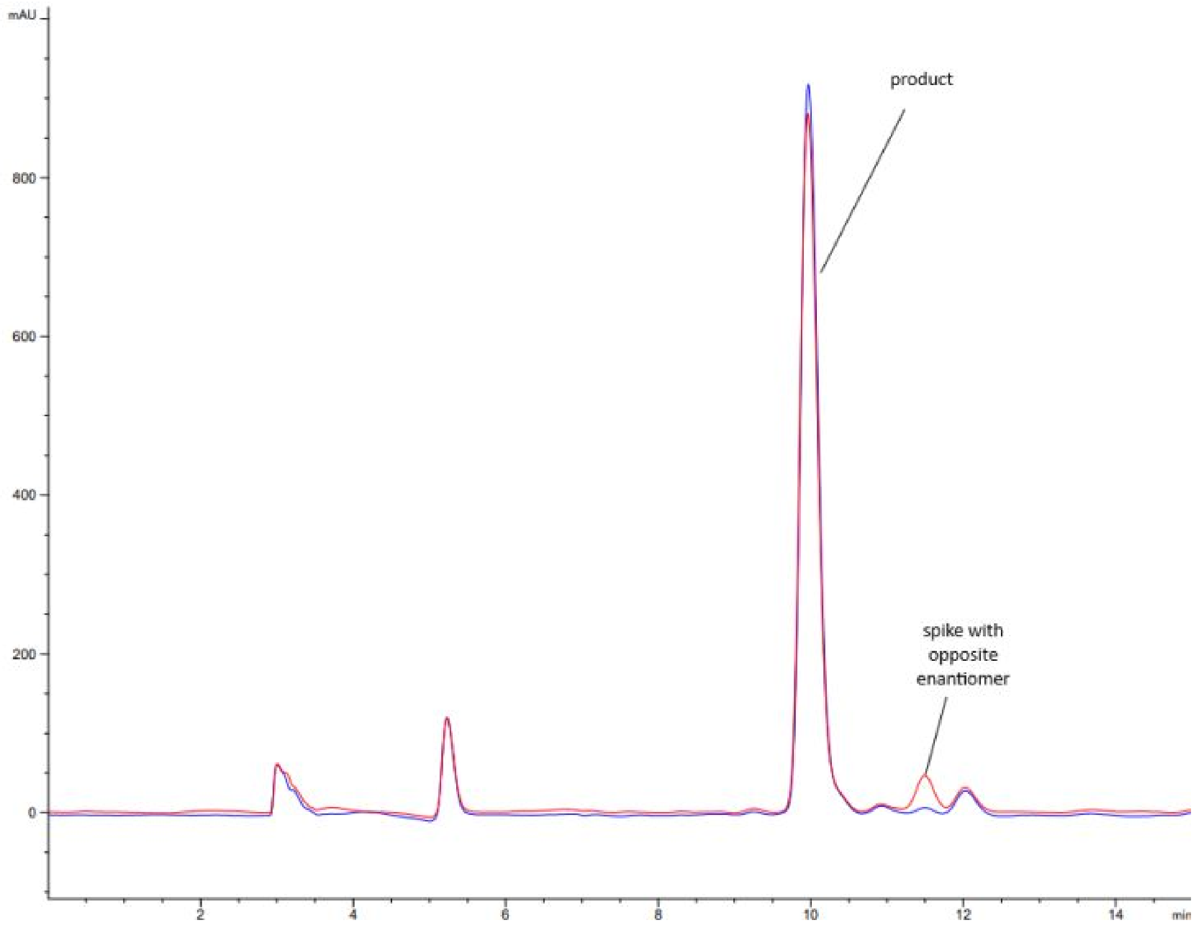

mAU
800
600
400
200
0
2
4
6
8
10
12
14
min
product
spike with
opposite
enantiomer

3.9 Synthesis of a *trans*- Stereoisomer of Centrolobine (Manuscript Scheme 3)

Synthesis of (2*S*,6*R*)-6-(4-methoxyphenyl)tetrahydro-2*H*-pyran-2-carbaldehyde, *ent*-**2c**

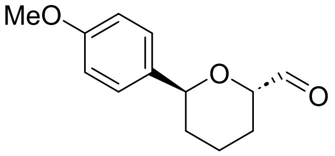

ent-**2d**
$C_{13}H_{16}O_3$
MW: 220.27

To a glass vial was added bis(acetonitrile)palladium(II) *p*-toluenesulfonate (9.3 mg, 18.0 µmol, 4 mol%), PyrOx (*R*)-**L1** (7.2 mg, 26.0 µmol, 6 mol%), copper(II) triflate(3.2 mg, 8.8 µmol, 2 mol%), 6 × 3 Å molecular sieve beads (~250 mg) and anhydrous DMF (2.2 mL, 0.2 M). The resulting mixture was stirred (800 rpm) open to the air at RT for 20 min. A solution of (*S*)-(3,4- dihydro-2*H*-pyran-2-yl)methanol (*S*)-**1** (50.0 mg, 0.44 mmol, 1.0 eq.) and (*p*-methoxyphenyl)boronic acid (133 mg, 0.88 mmol, 2.0 eq.) in anhydrous DMF (2.2 mL, 0.2 M) was then added in a single portion. Water (7.89 µL, 0.44 mmol, 1.0 eq.) was then quickly added. The resulting mixture was then stirred (800 rpm) open to the air at RT for 23 h. After this time, the reaction mixture was diluted with EtOAc (10 mL), quenched with 5%*w/w* aqueous lithium chloride (5 mL), the layers separated and the organic phase washed with 5%*w/w* aqueous lithium chloride (4 × 5 mL). The combined aqueous phases were back-extracted with EtOAc (10 mL), the combined organic phases dried (phase separator) and the solvent removed *in vacuo*. Purification by automated column chromatography (0-100% ethyl acetate in heptane) afforded *ent*-**2c** as a pale yellow oil (58 mg, 60%). 1H NMR (400 MHz, $CDCl_3$, 30 °C, TMS) δ = 9.96 (s, 1H), 7.35 (td, *J* = 8.6, ^4J = 2.4 Hz, 2H), 6.93 (td, *J* = 8.6, ^4J = 2.4 Hz, 2H), 4.67 (dd, *J* = 9.5, 2.7 Hz, 1H), 4.34 (dd, *J* = 6.4, 2.9 Hz, 1H), 3.83 (s, 3H), 1.89 - 1.74 (m, 4H), 1.65 - 1.52 (m, 2H) ppm; ^{13}C NMR (101 MHz, $CDCl_3$, 27 °C, TMS) δ = 204.9, 159.2, 134.1, 127.4, 113.9, 79.4, 76.2, 55.8, 31.9, 23.7, 20.3 ppm.

A sample of racemic aldehyde *rac*-**2d** was prepared using the same procedure except that (3,4-dihydro-2*H*-pyran-2-yl)methanol *rac*-**1** (50.0 mg, 0.44 mmol, 1.0 eq.) and PyrOx **L0** (5.7 mg, 26.5 µmol, 6 mol%) were used. Under these conditions, *rac*-**2d** was obtained in 50% yield. All characterisation was in agreement with the enantioenriched material.

Synthesis of (4-(benzyloxy)benzyl)triphenylphosphoniumbromide **4**[8]

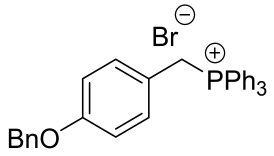

4
$C_{32}H_{28}OPBr$
MW: 539.49

To a solution of (4-(benzyloxy)phenyl)methanol (1.00 g, 4.67 mmol, 1.0 eq.) in Et_2O (30 mL) at 0 °C under nitrogen was slowly added bromotrimethylsilane (1.30 mL, 9.52 mmol, 2.04 eq.). The resultant solution was allowed to warm slowly to RT and stirred for 16 h. After this time, water (60 mL) was added with stirring. The layers were separated and the aqueous phase was back-extracted with Et_2O (4 × 30 mL). The combined organic phases were washed with saturated aqueous brine (75 mL), dried (phase separator) and the solvent removed *in vacuo*. Toluene (45 mL) and triphenylphosphine (3.67 g, 14.0 mmol, 3.0 eq.) were subsequently added at RT. The resultant solution was heated at reflux under nitrogen for 16 h. After this time, the reaction mixture was concentrated under vacuum. The resultant white solid was triturated with Et_2O (20 mL) then isolated by filtration, washing with Et_2O (20 mL). The solid was then dried under vacuum to give phosphonium salt **4** as a white solid (2.35 g, 93%). ^{1}H NMR (400 MHz, $CDCl_3$, 30 °C, TMS) δ = 7.79 - 7.71 (m, 9H), 7.66 - 7.59 (m, 6H), 7.37 (d, J = 4.4 Hz, 2H), 7.35 - 7.29 (m, 3H), 7.03 (dd, J = 8.8, 2.4 Hz, 2H), 6.73 (d, J = 8.8 Hz, 2H), 5.36 (d, $^2J_{P-H}$ = 13.7 Hz, 2H), 4.98 (s, 2H) ppm; ^{13}C NMR (101 MHz, $CDCl_3$, 27 °C, TMS) δ = 158.7 (d, $^5J_{C-P}$ = 4.6 Hz), 136.6, 134.9 (d, $^4J_{C-P}$ = 3.1 Hz), 134.4 (d, $^3J_{C-P}$ = 7.6 Hz), 132.7 (d, $^3J_{C-P}$ = 6.1 Hz), 130.1 (d, $^2J_{C-P}$ = 13.7 Hz), 128.6, 128.0, 127.5, 118.9 (d, $^2J_{C-P}$ = 7.6 Hz), 117.9 (d, $^1J_{C-P}$ = 85.5 Hz), 115.2 (d, $^4J_{C-P}$ = 4.6 Hz), 69.9, 30.2 (d, $^1J_{C-P}$ = 45.8 Hz) ppm; ^{31}P{^{1}H} NMR (162 MHz, $CDCl_3$) δ = 22.45 (s, 1P) ppm; ν_{max} / cm^{-1} 3046, 2846, 2770, 1604, 1579, 1508, 1435, 1236, 1111, 747, 739, 714, 695, 682, 519, 487, 499; HRMS (ES) *m/z* calcd for $[M]^+$ $C_{32}H_{28}OP$: 459.1881; found: 459.1878; mp 221-226 °C. All spectroscopic data was in agreement with the literature.[8]

Synthesis of (2*S*,6*S*)-2-(4-(benzyloxy)styryl)-6-(4-methoxyphenyl)tetrahydro-2*H*-pyran **5**

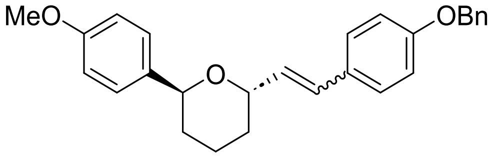

5
$C_{27}H_{28}O_3$
MW: 400.52

To a round-bottom flask was added (4-(benzyloxy)benzyl)triphenylphosphonium bromide **4** (75 mg, 0.14 mmol, 1.2 eq.) and a stir bar. The flask was purged with nitrogen for 30 min. Anhydrous THF (3 mL) was added and the resultant white solution was cooled to 0 °C. Potassium *tert*-butoxide (17.8 mg, 0.16 mmol, 1.4 eq.) was added and the resultant orange solution was stirred for 30 min at 0 °C. After this time, a solution of (2*S*,6*S*)-6-(4-methoxyphenyl)tetrahydro-2*H*-pyran-2-carbaldehyde *ent*-**2c** (25 mg, 0.11 mmol, 1.0 eq.) in anhydrous THF (3 mL) was added dropwise. The orange mixture was allowed to warm to RT. Water (5 mL) and EtOAc (10 mL) were added, the layers separated and the aqueous phase back-extracted with EtOAc (2 × 10 mL). The combined organic phases were dried (phase separator) and concentrated *in vacuo*. Purification by automated column chromatography (0-20% ethyl acetate in heptane) afforded **5** (3:2 *E*:*Z*) as a colourless oil (33 mg, 72%).

Note: the following data is for the *E*/*Z* mixture. ^{1}H NMR (400 MHz, $CDCl_3$, 30 °C, TMS) δ = 7.44 - 7.24 (m, 9H), 6.98 - 6.82 (m, 4H), 6.61 - 6.52 (m, 1H), 6.26 (dd, *J* = 16.1, 4.9 Hz, 0.4H), 6.00 (dd, *J* = 11.7, 8.3 Hz, 0.6H), 5.07 (s, 0.8H), 5.04 (s, 1.2H), 4.84 - 4.76 (m, 1.4H), 4.59 - 4.54 (m, 0.4H), 3.80 (s, 1.1H), 3.78 (s, 1.8H), 1.98 - 1.61 (m, 6H) ppm; ^{13}C NMR (101 MHz, $CDCl_3$, 27 °C, TMS) δ = 158.7, 158.7, 158.4, 158.0, 137.0, 135.1, 134.8, 132.3, 130.7, 130.5, 130.1, 129.7, 128.9, 128.6, 128.1, 127.9, 127.7, 127.6, 127.6, 127.4, 115.0, 114.9, 114.6, 113.8, 113.7, 113.6, 72.7, 72.5, 72.1, 70.1, 67.0, 68.9, 55.29, 55.27, 31.5, 31.4, 30.0, 29.5, 19.4, 19.2 ppm; $[\alpha]_D^{21}$ = -364 (*c* 0.1 in $CHCl_3$); ν_{max} / cm^{-1} 3030, 2934, 2881, 1606, 1509, 1244, 1174, 1026, 829.

A racemic sample, *rac*-**5**, was prepared as follows:

To a round-bottom flask was added (4-(benzyloxy)benzyl)triphenylphosphonium bromide **4** (225 mg, 0.41 mmol, 1.2 eq.) and a stir bar. The flask was purged with nitrogen for 30 min. Anhydrous THF (4.5 mL) was added and the resultant white solution was cooled to 0 °C. Potassium *tert*-butoxide (53.2 mg, 0.47 mmol, 1.4 eq.) was added and the resultant orange solution was stirred for 30 min at 0 °C. After

this time, a solution of 6-(4-methoxyphenyl)tetrahydro-2*H*-pyran-2-carbaldehyde *rac*-**2d** (74.6 mg, 0.34 mmol, 1.0 eq.) in anhydrous THF (4.5 mL) was added dropwise. The orange mixture was allowed to warm to RT. Water (10 mL) and EtOAc (20 mL) were added, the layers separated and the aqueous phase back-extracted with EtOAc (2 × 20 mL). The combined organic phases were dried (phase separator) and concentrated *in vacuo*. Purification by automated column chromatography (0-20% ethyl acetate in heptane) afforded *rac*-**5** (3:2 *E*:*Z*) as a colourless oil (108 mg, 80%).

Synthesis of (3*S*,7*S*)-Centrolobine **6**

A vial containing 5%w/w Pd/alumina (6 mg, 3.5 mol%) and (2*S*,6*S*)-2-(4-(benzyloxy)styryl)-6-(4-methoxyphenyl)tetrahydro-2*H*-pyran **5** (32.1 mg, 80.0 µmol, 1.0 eq.) in anhydrous THF (1 mL) was heated to 50 °C and placed under a hydrogen atmosphere (5 bar / 75 PSI) in an Endeavour reactor, and stirred for 17 h. The reaction mixture was filtered to remove the palladium and the solvent removed *in vacuo.* Purification by automated reverse phase column chromatography (formic method, 5-95% 0.1% formic acid in MeCN in 0.1% formic acid in water) afforded centrolobine **6** as a colourless oil (17 mg, 68%). ^{1}H NMR (400 MHz, $CDCl_3$, 30 °C, TMS) δ = 7.35 (d, *J* = 8.8 Hz, 2H), 7.08 (d, *J* = 8.3 Hz, 2H), 6.92 (d, *J* = 8.8 Hz, 2H), 6.75 (d, *J* = 8.3 Hz, 2H), 4.83 (t, *J* = 5.4 Hz, 1H), 3.84 (s, 3H), 3.82 - 3.75 (m, 1H), 2.77 (ddd, ^{2}J = 14.2, *J* = 10.5, 5.4 Hz, 1H), 2.58 (ddd, ^{2}J = 14.2, *J* = 10.5, 6.6 Hz, 1H), 2.16 - 2.06 (m, 1H), 1.97 - 1.89 (m, 2H), 1.81 - 1.66 (m, 4H), 1.53 - 1.43 (m, 1H) ppm; ^{13}C NMR (101 MHz, $CDCl_3$, 27 °C, TMS) δ = 158.6, 153.6, 134.6, 134.4, 129.5 (2 × C), 127.8 (2 × C), 115.2 (2 × C), 113.8 (2 × C), 71.9, 71.3, 55.3, 35.3, 31.3, 30.1, 30.0, 19.1 ppm; $[\alpha]_D^{21}$ = -2.8 (*c* 0.1 in DCM) [lit.[9a] $[\alpha]_D^{25}$= -3.8, *c* 0.8 in $CHCl_3$, lit.[9b] $[\alpha]_D^{20}$= −3.6. *c* 0.6 in DCM)]; ν_{max} / cm^{-1} 3371, 2957, 2929, 2856, 1699, 1409, 1090, 834, 770. All spectroscopic data was in agreement with the literature.[9]

A racemic sample of centrolobine, *rac*-**6**, could be generated by the same method, except using 2-(4-(benzyloxy)styryl)-6-(4-methoxyphenyl)tetrahydro-2*H*-pyran *rac*-**5** (32.1 mg, 80.0 µmol, 1.0 eq.) in place of (2*S*,6*S*)-2-(4-(benzyloxy)styryl)-6-(4-methoxyphenyl)tetrahydro-2*H*-pyran **5**. Following this procedure, 21 mg of *rac*-centrolobine *rac*-**6** (84% yield) was obtained.

4. NMR Spectra

(*S*)-*N*-(1-hydroxy-3,3-dimethylbutan-2-yl)-5-(trifluoromethyl)picolinamide, *int*-PyrOx **L1**

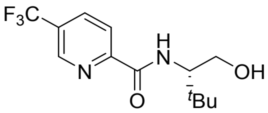

int-PyrOx **L1** ^{1}H NMR (400 MHz, $CDCl_3$):

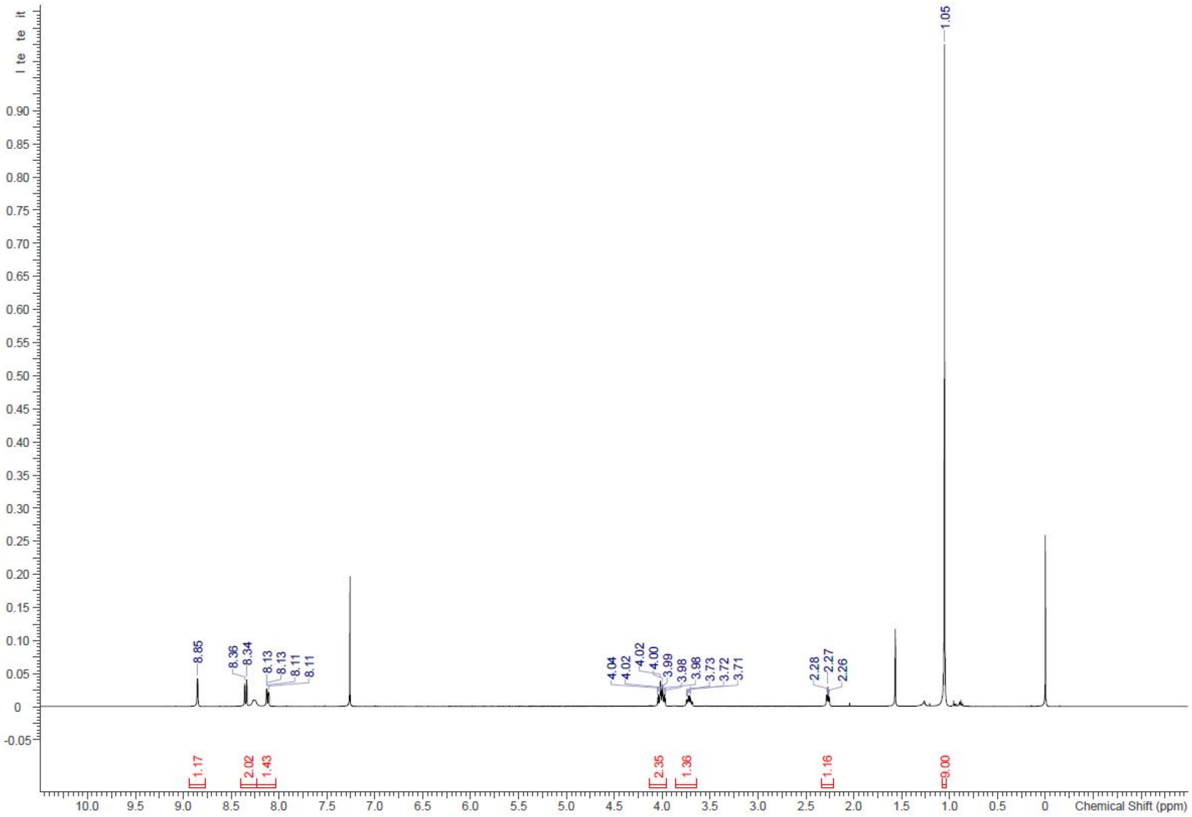

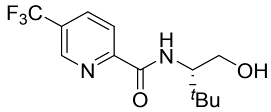

int-PyrOx **L1** ^{13}C NMR (101 MHz, $CDCl_3$):

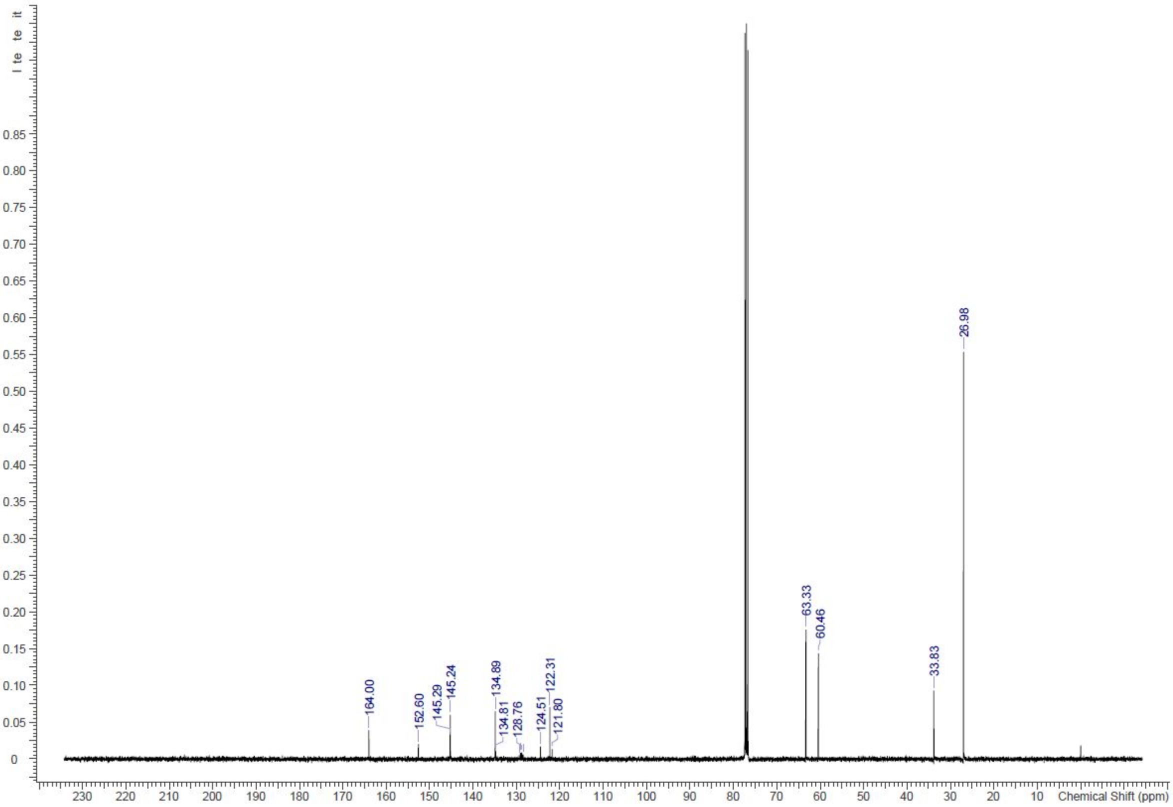

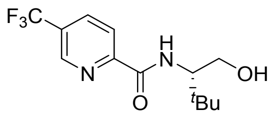

int-PyrOx **L1** $^{19}F\{^{1}H\}$ NMR (376 MHz, $CDCl_3$):

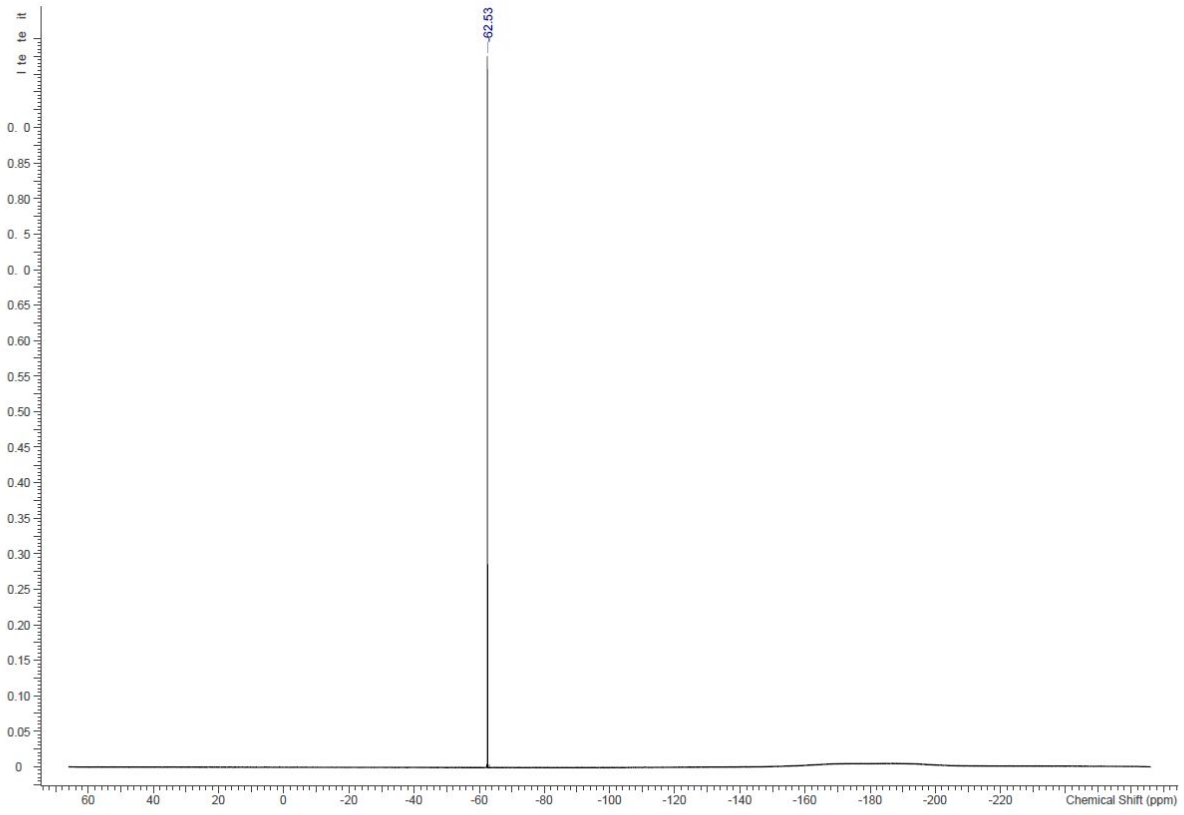

(*S*)-4-(*tert*-butyl)-2-(5-(trifluoromethyl)pyridin-2-yl)-4,5-dihydrooxazole, PyrOx **L1**

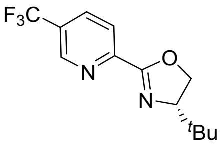

PyrOx **L1** ^{1}H NMR (400 MHz, $CDCl_3$):

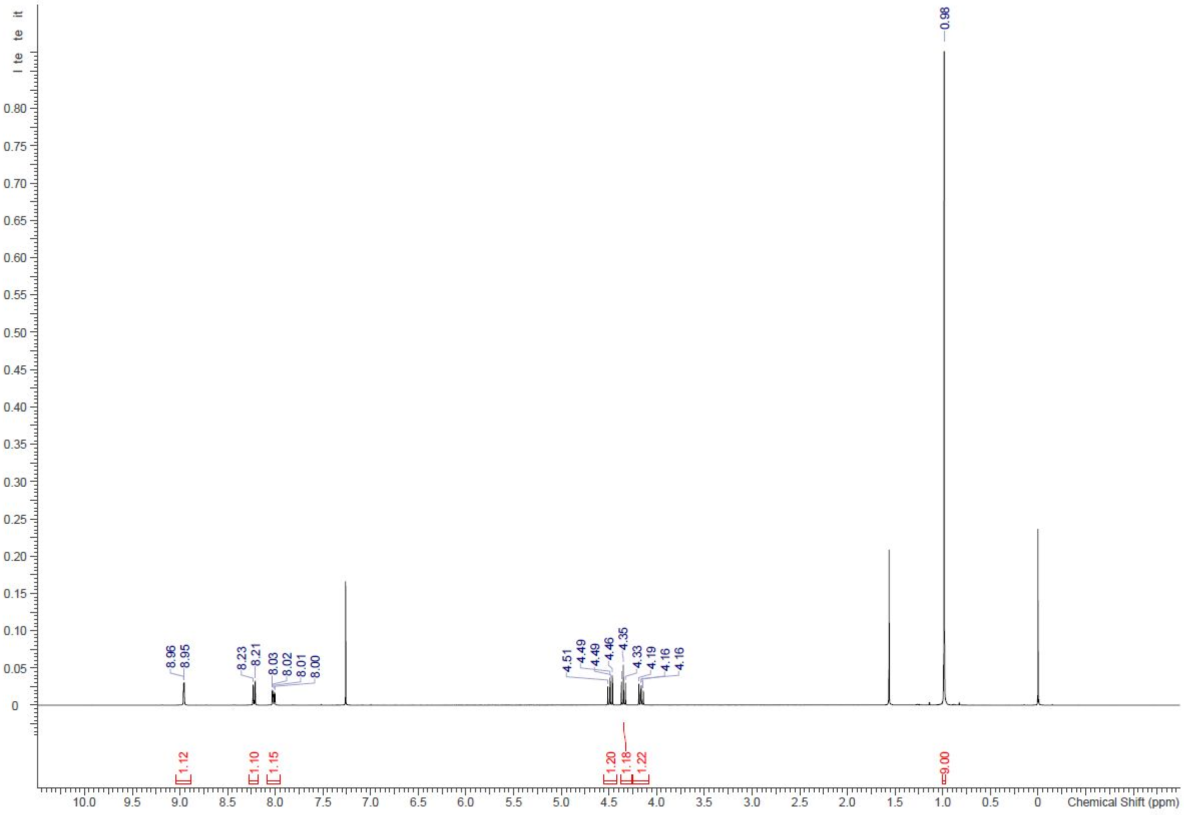

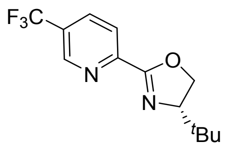

PyrOx **L1** ^{13}C NMR (101 MHz, $CDCl_3$):

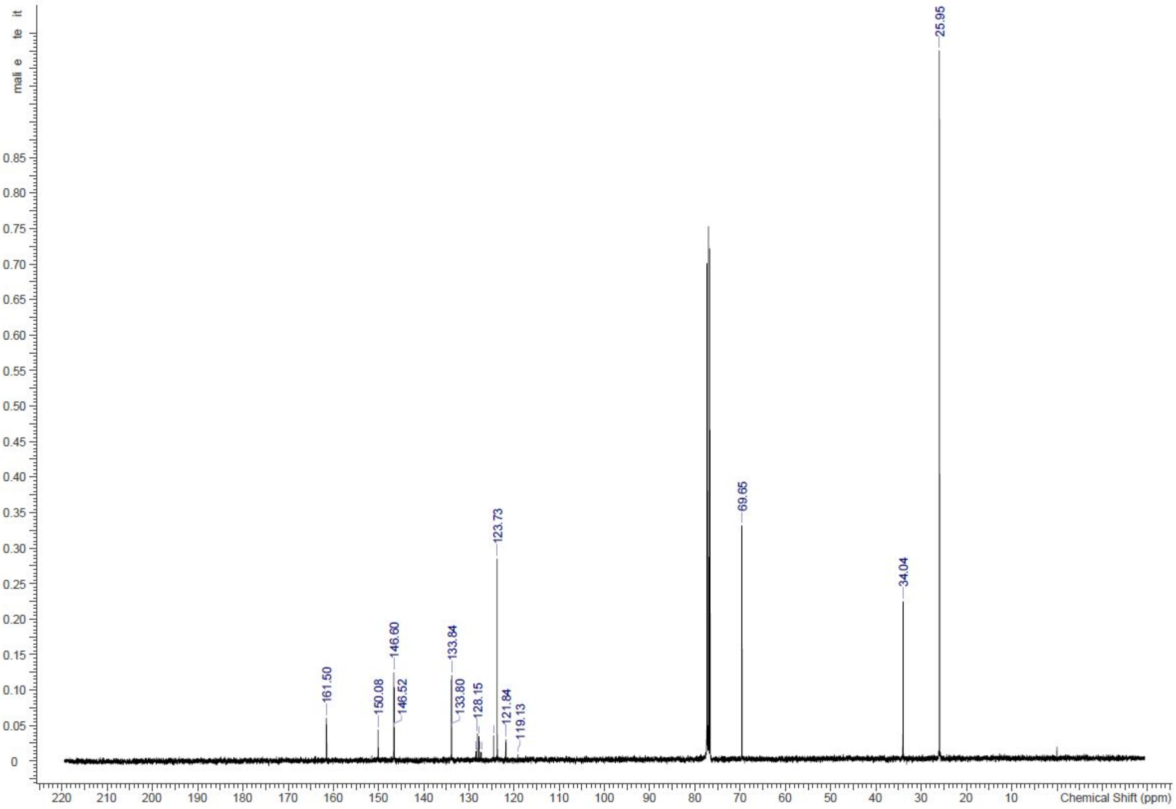

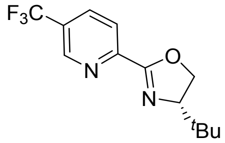

int-PyrOx **L1** $^{19}F\{^1H\}$ NMR (376 MHz, $CDCl_3$):

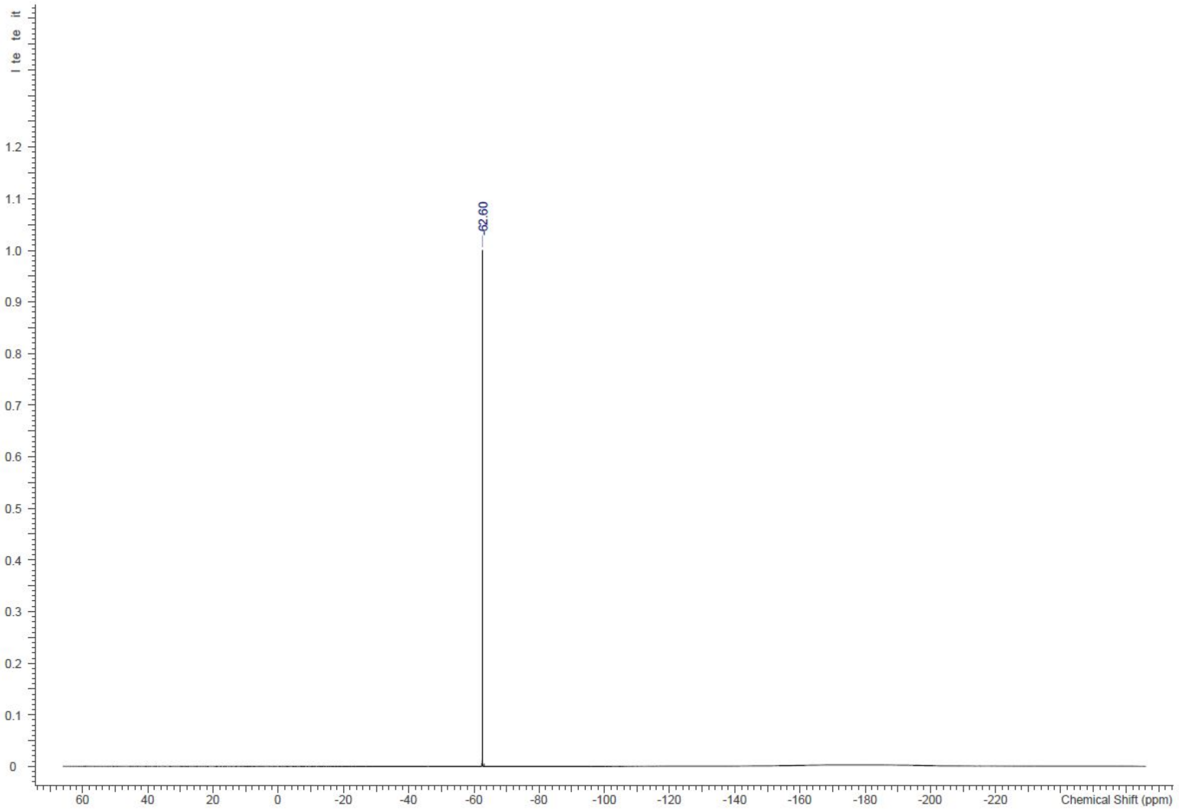

(*S*)-4-(*tert*-butyl)-2-(5-nitropyridin-2-yl)-4,5-dihydrooxazole, PyrOx **L2**

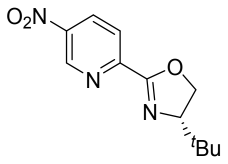

PyrOx **L2** ^{1}H NMR (400 MHz, $CDCl_3$):

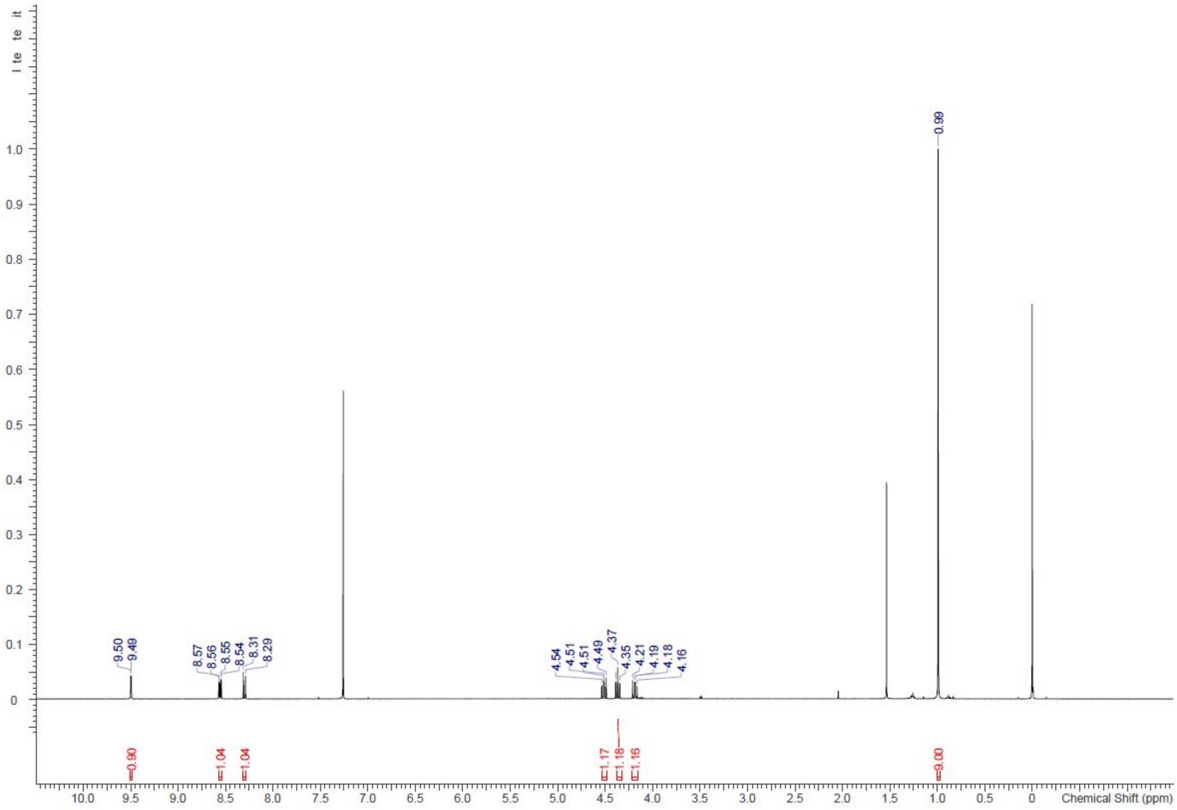

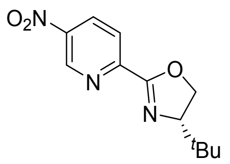

PyrOx **L2** ^{13}C NMR (101 MHz, $CDCl_3$):

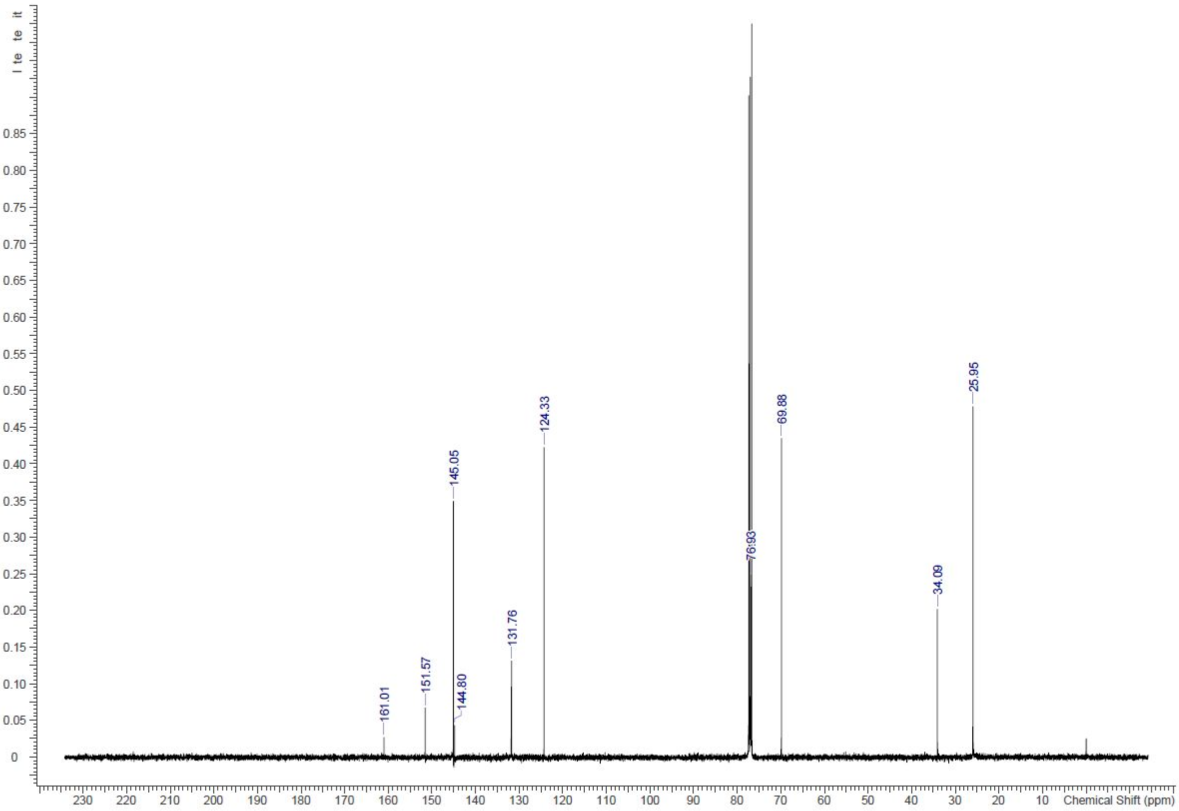

(S)-N-(1-hydroxy-3,3-dimethylbutan-2-yl)-5-nitropicolinamide, *int*-PyrOx **L3**

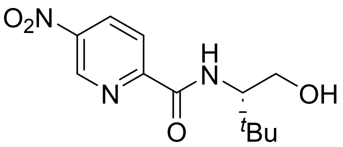

int-PyrOx **L3** ^{1}H NMR (400 MHz, $CDCl_3$):

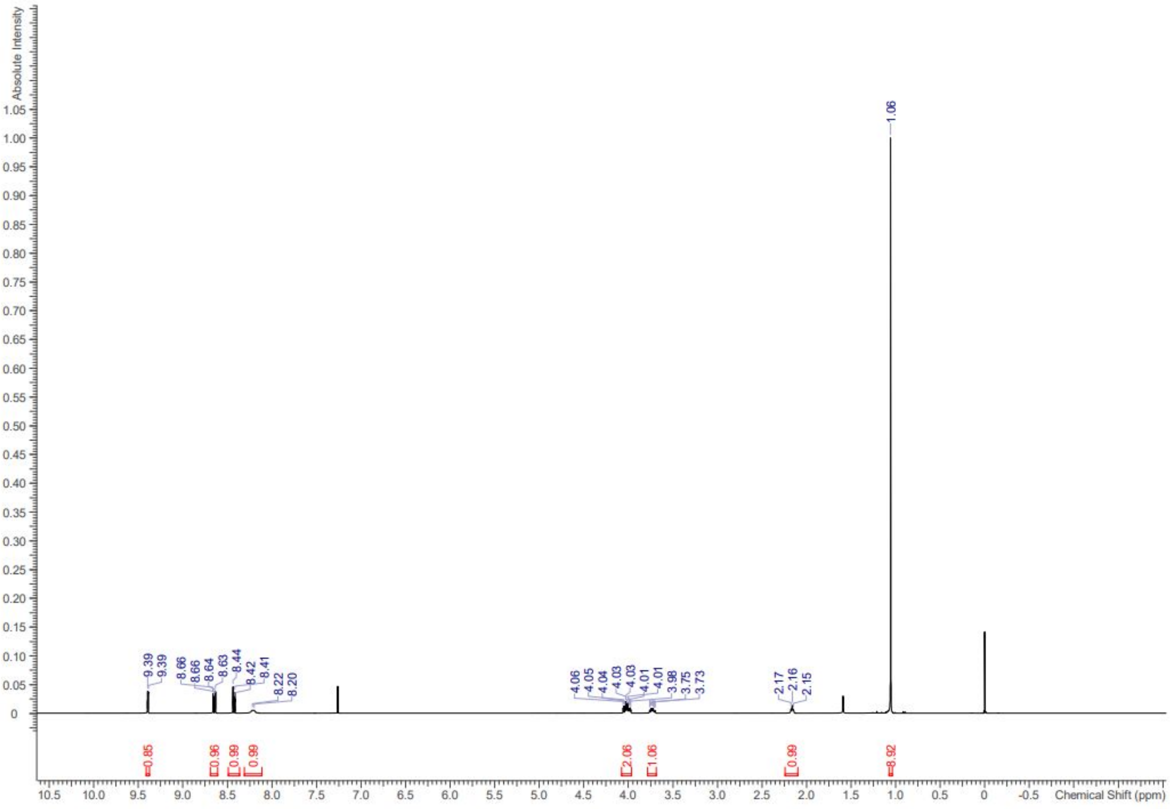

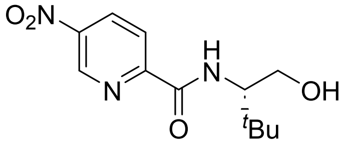

int-PyrOx **L3** ^{13}C NMR (101 MHz, $CDCl_3$):

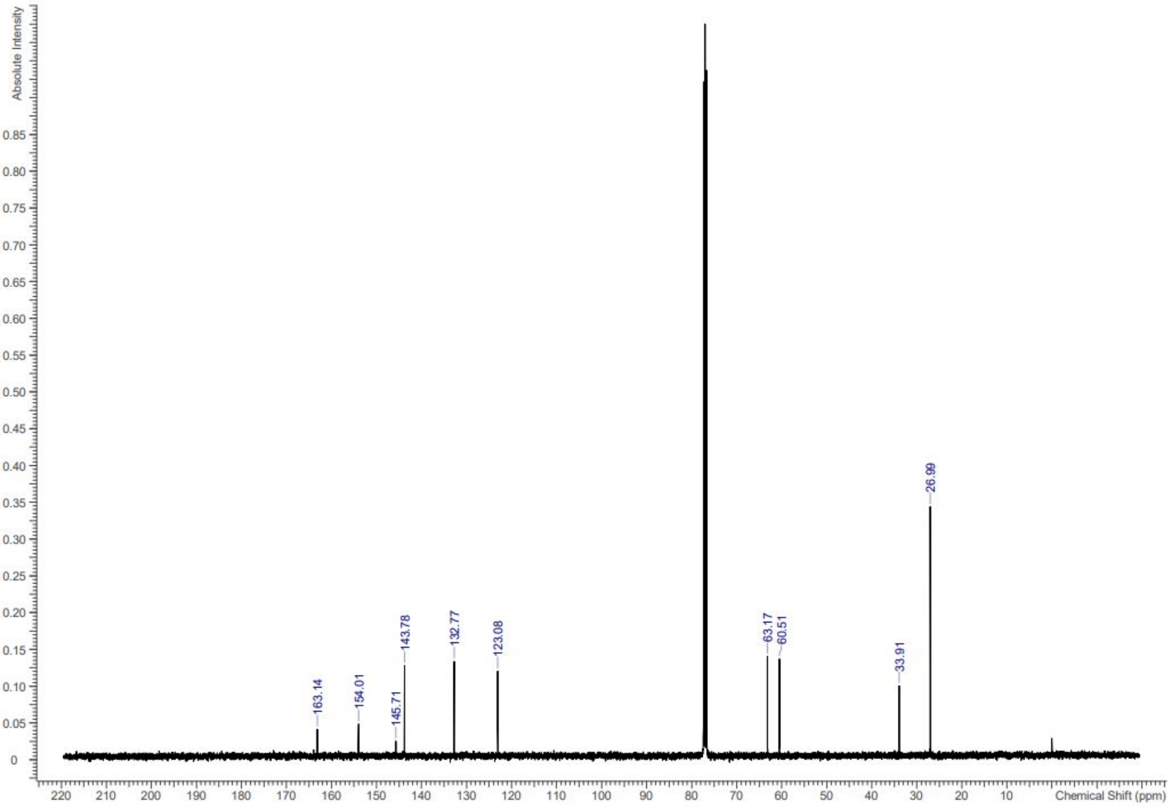

(*S*)-4-(*tert*-butyl)-2-(5-methoxypyridin-2-yl)-4,5-dihydrooxazole, PyrOx **L3**

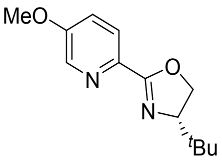

PyrOx **L3** ^{1}H NMR (400 MHz, $CDCl_3$):

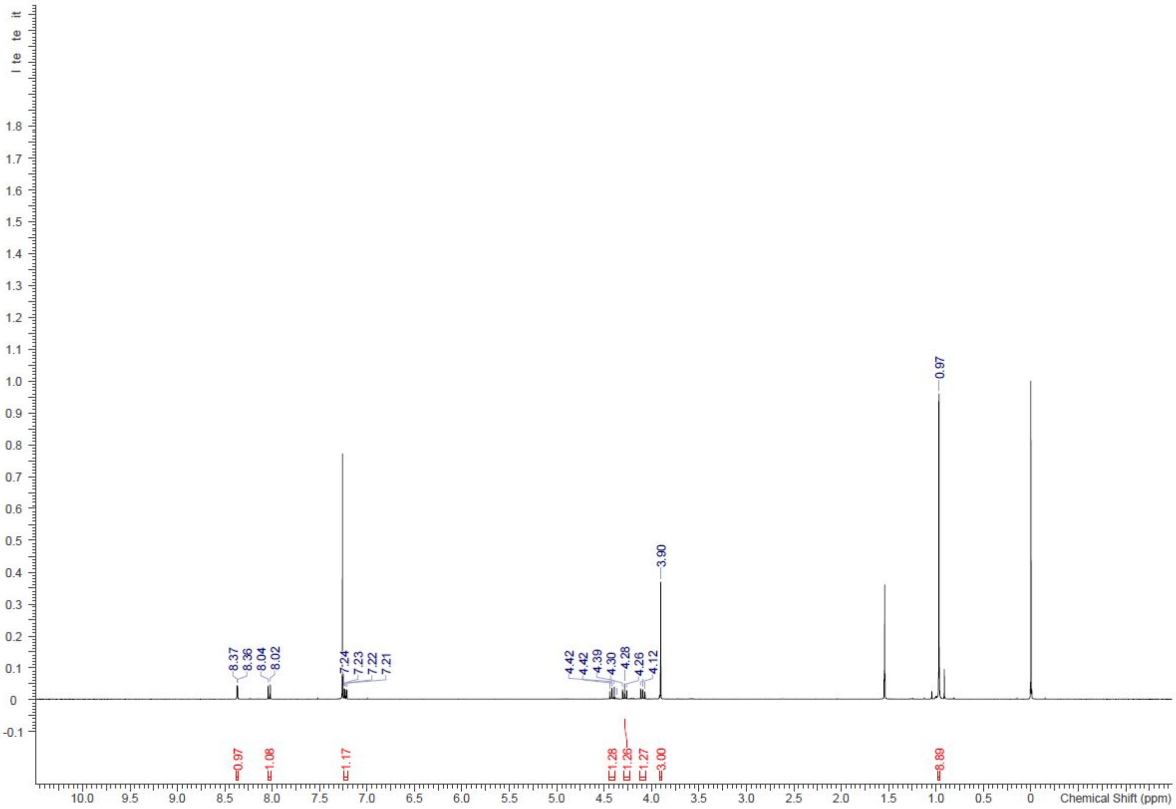

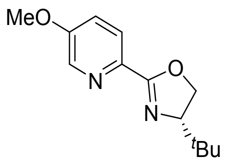

PyrOx **L3** ^{13}C NMR (101 MHz, $CDCl_3$):

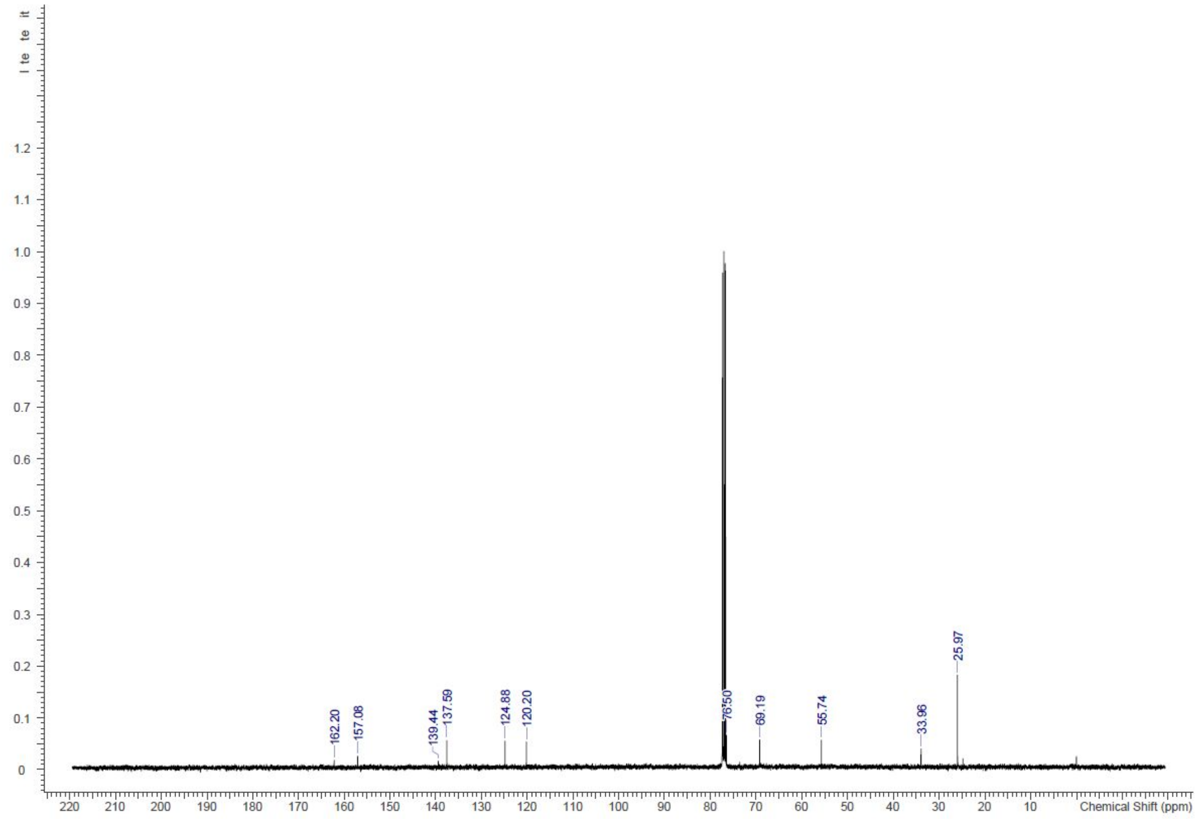

(*S*)-4-phenyl-2-(5-(trifluoromethyl)pyridin-2-yl)-4,5-dihydrooxazole, PyrOx **L4**

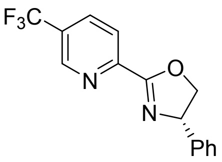

PyrOx **L4** ^{1}H NMR (400 MHz, $CDCl_3$):

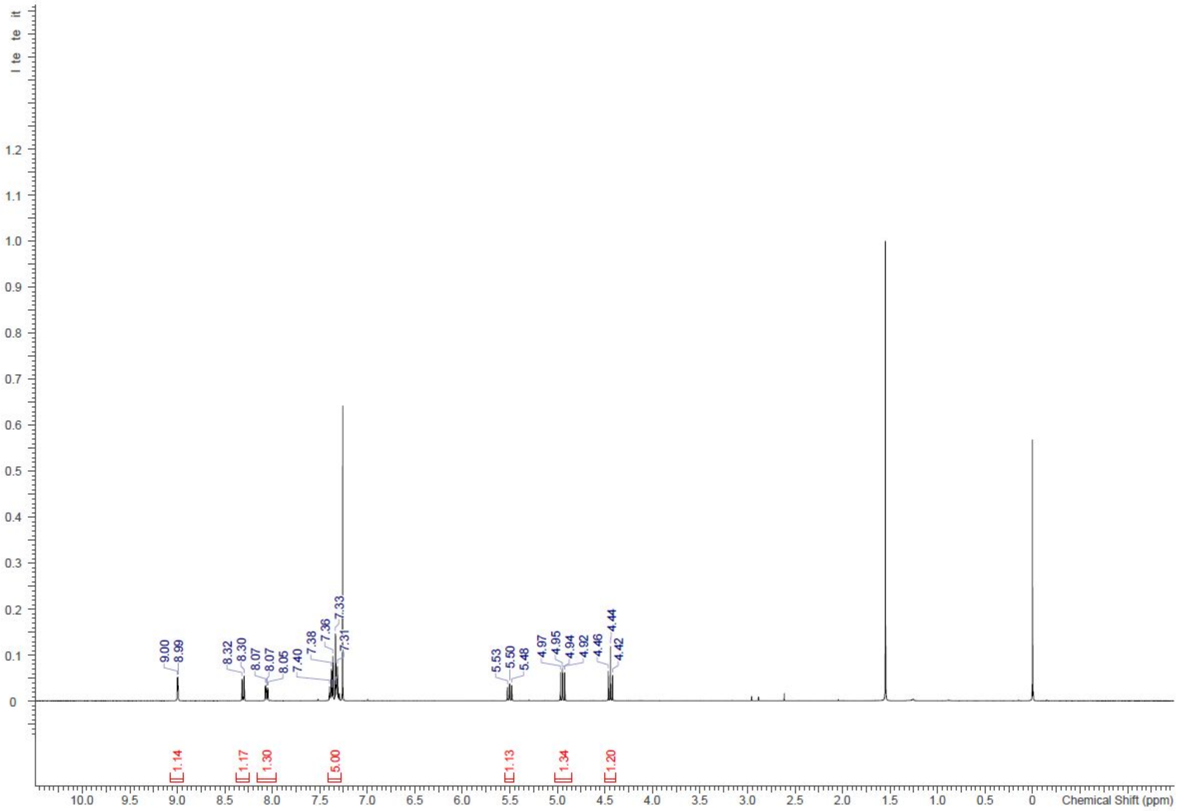

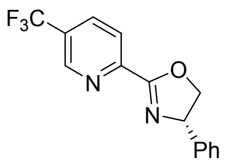

PyrOx **L4** ^{13}C NMR (101 MHz, $CDCl_3$):

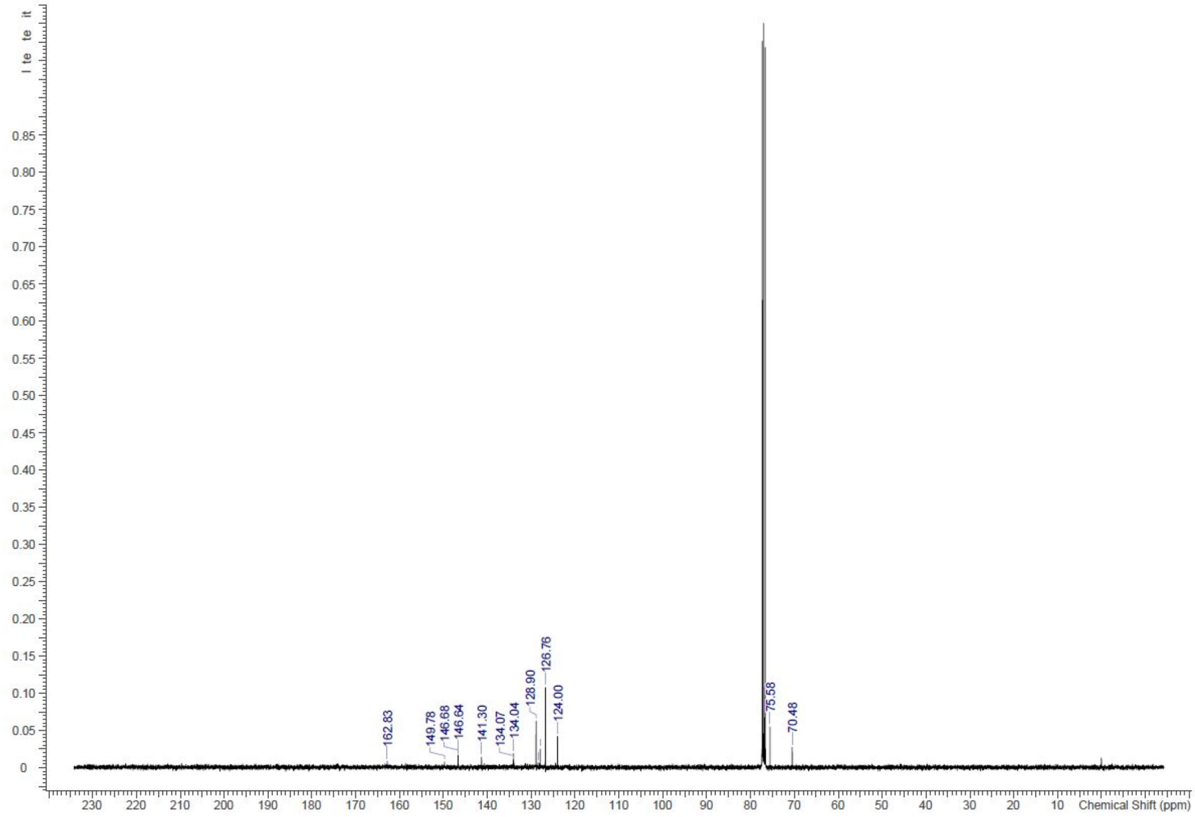

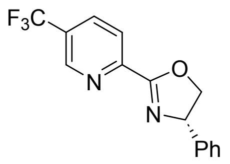

PyrOx **L4** ^{19}F NMR (376 MHz, $CDCl_3$):

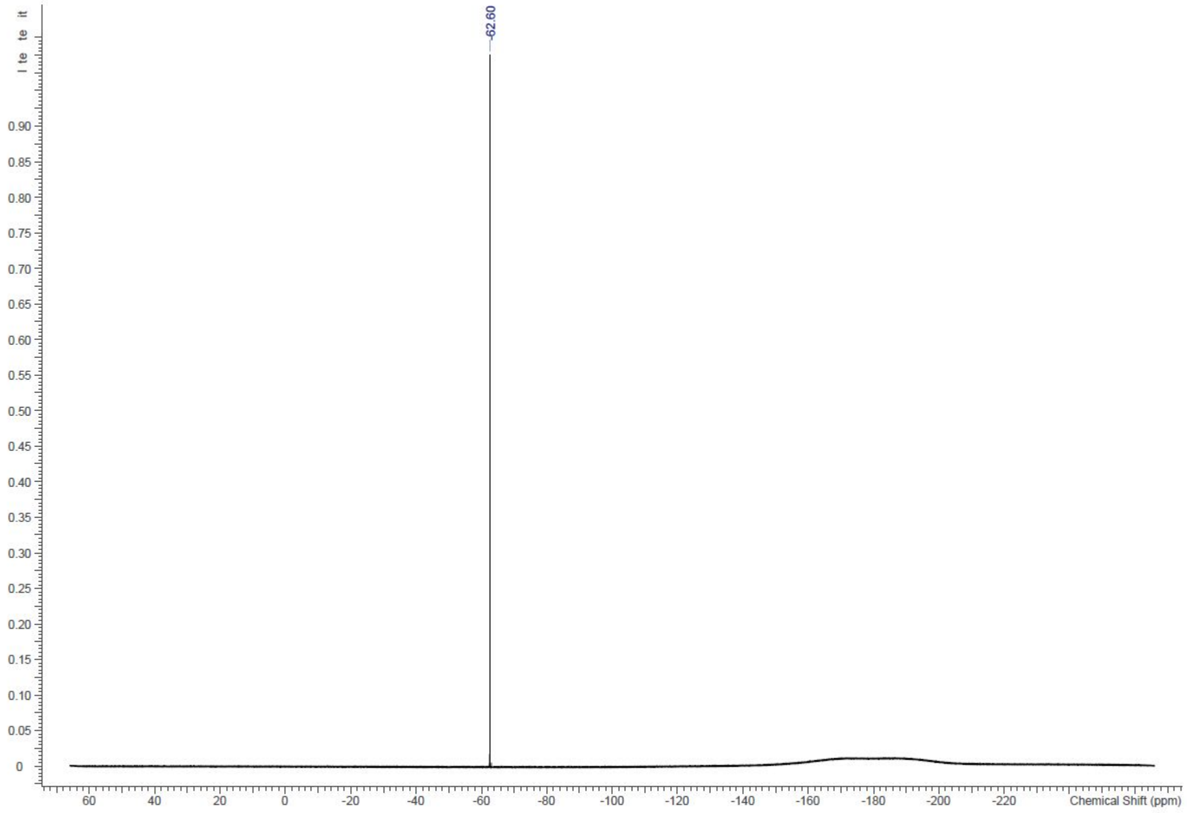

(S)-2-(5-nitropyridin-2-yl)-4-phenyl-4,5-dihydrooxazole, PyrOx **L5**

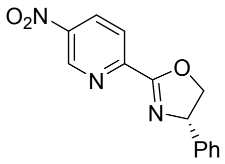

PyrOx **L5** ^{1}H NMR (400 MHz, $CDCl_3$):

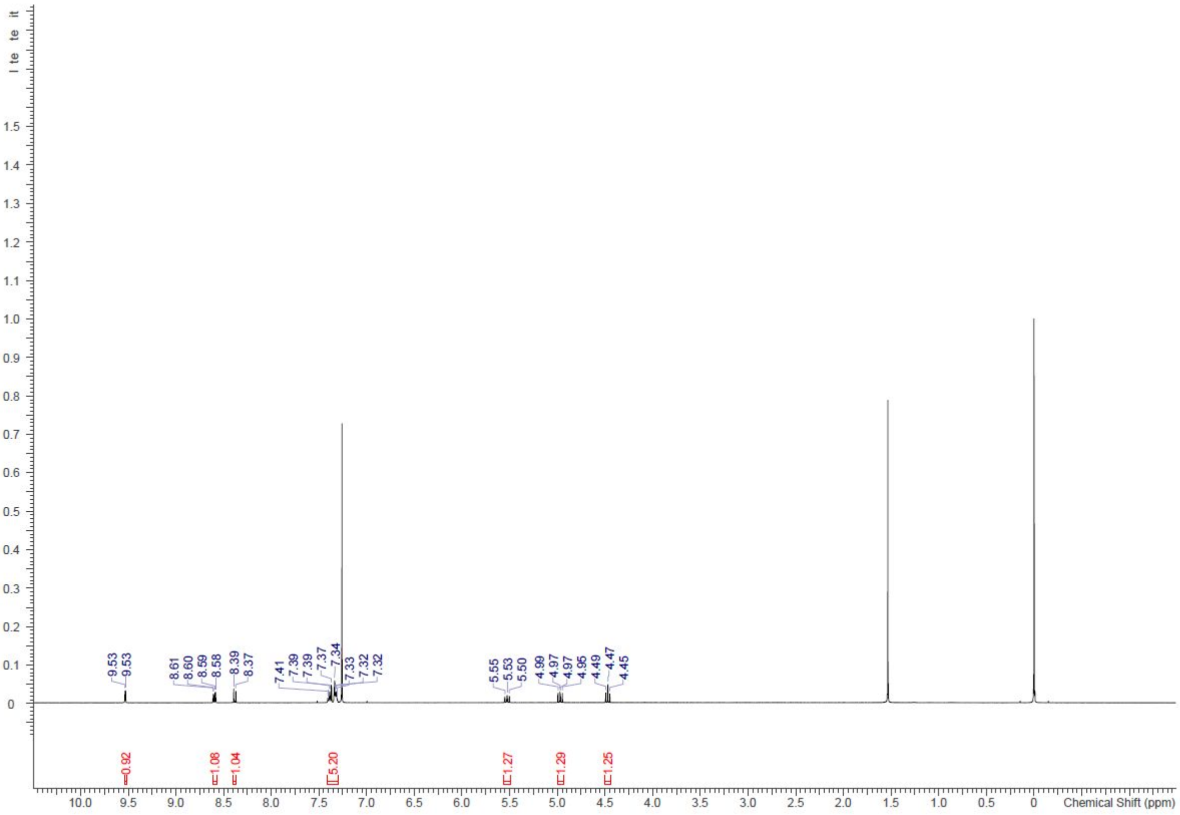

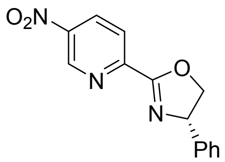

PyrOx **L5** ^{13}C NMR (101 MHz, $CDCl_3$):

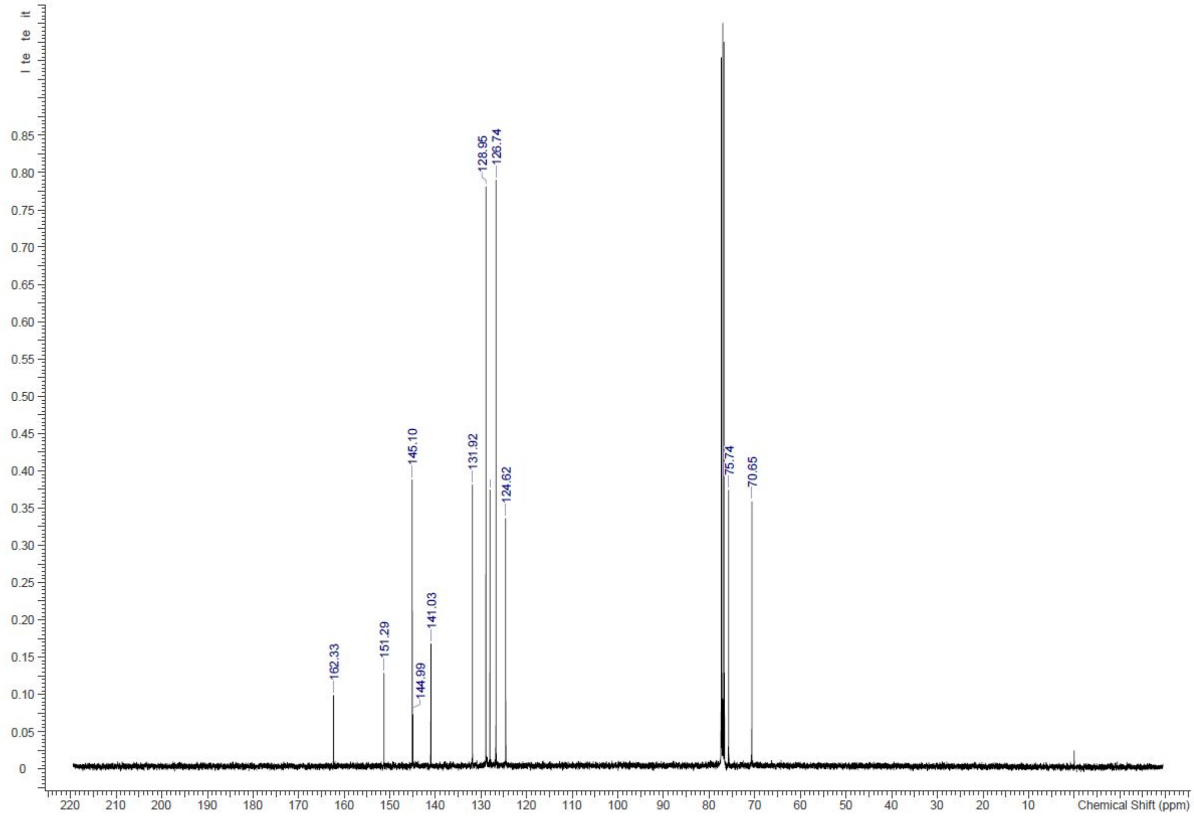

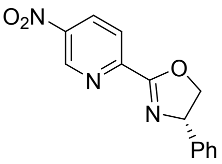

PyrOx **L5** [^{1}H,^{1}H]-COSY (400 MHz, $CDCl_3$):

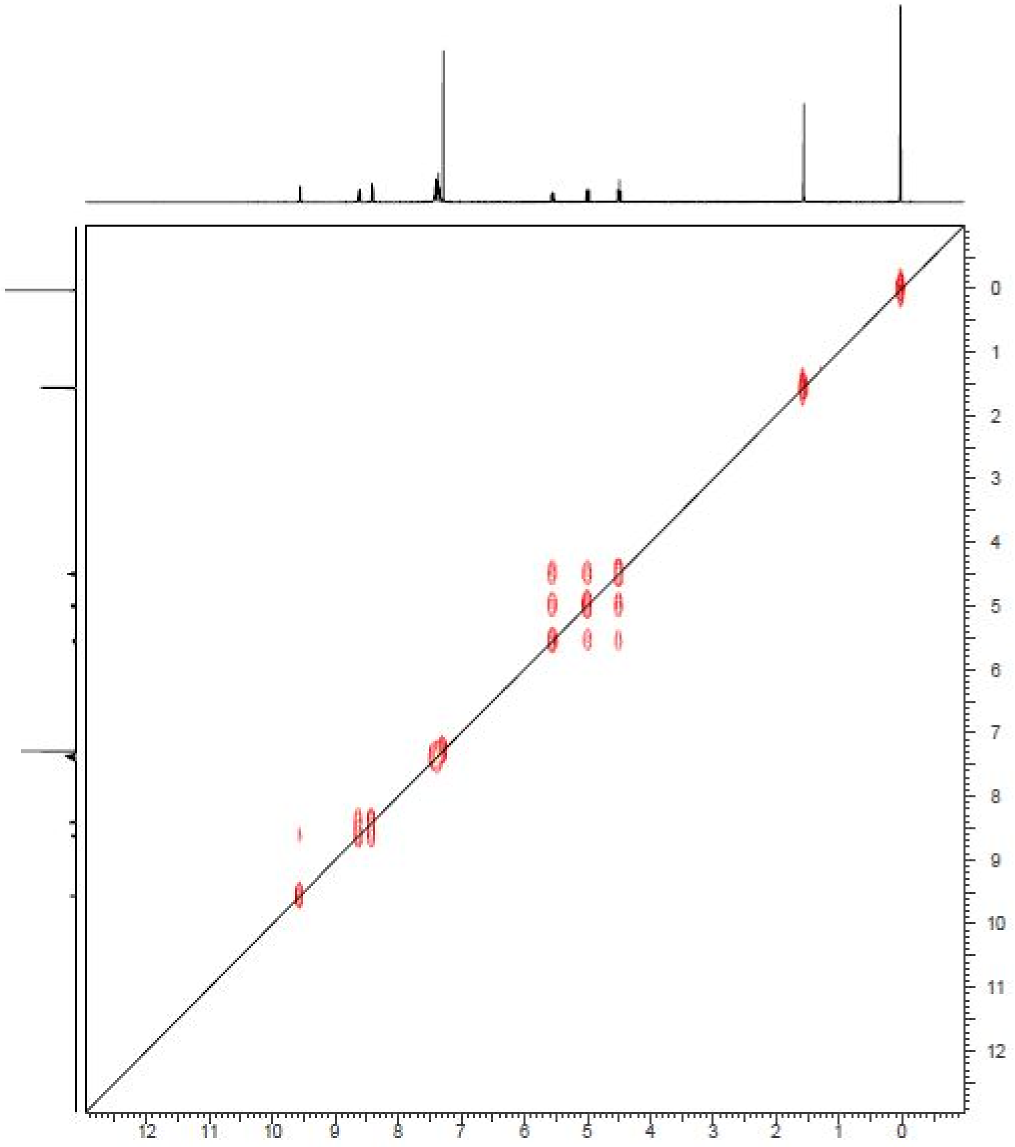

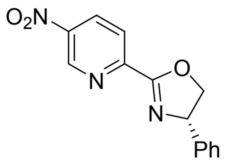

L5 [^{1}H,^{13}C]-HSQC (400 MHz, 101 MHz, $CDCl_3$):

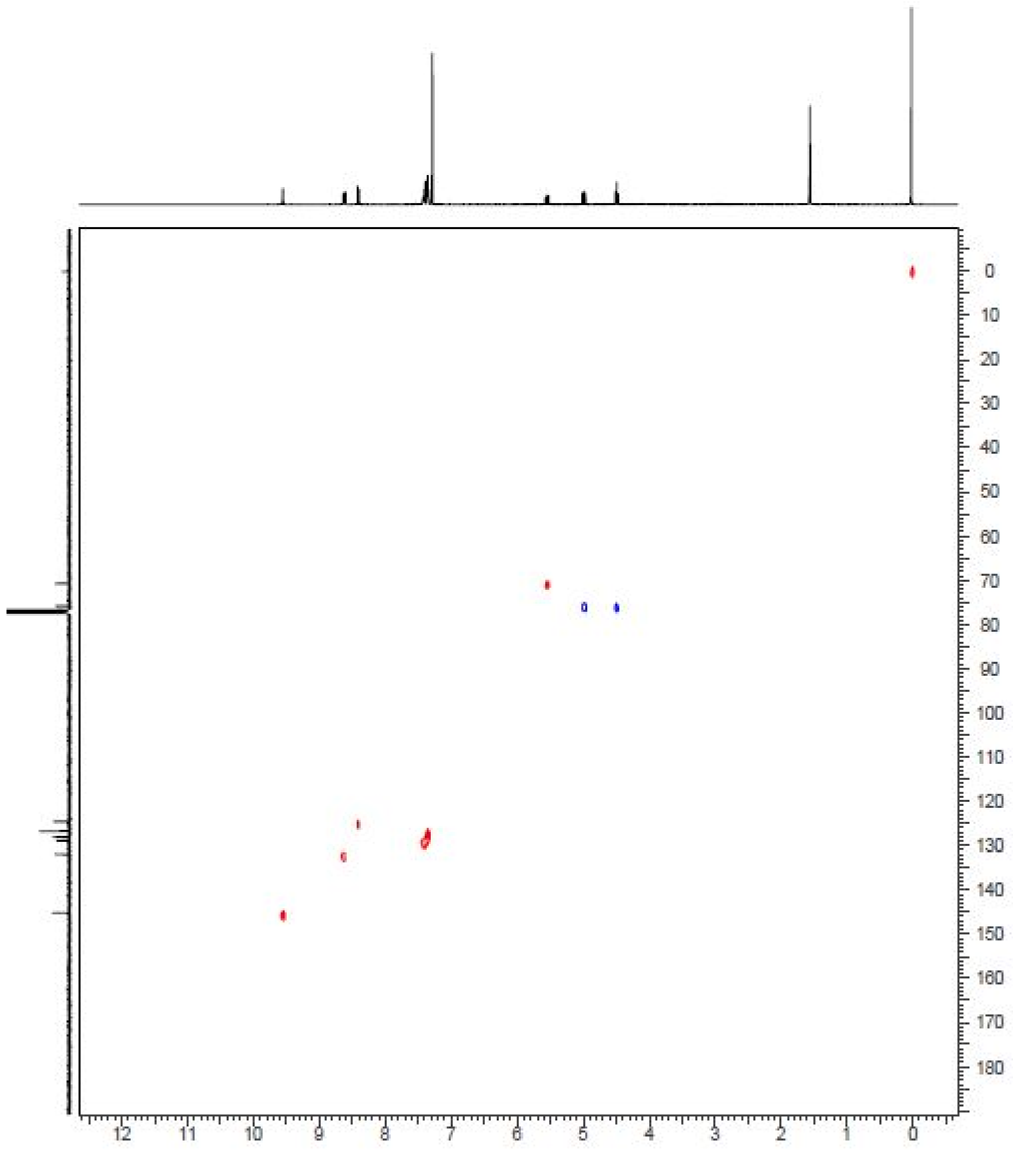

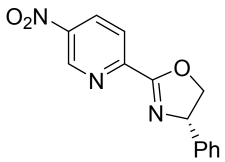

L5 [^{1}H,^{13}C]-HMBC (400 MHz, 101 MHz, $CDCl_3$):

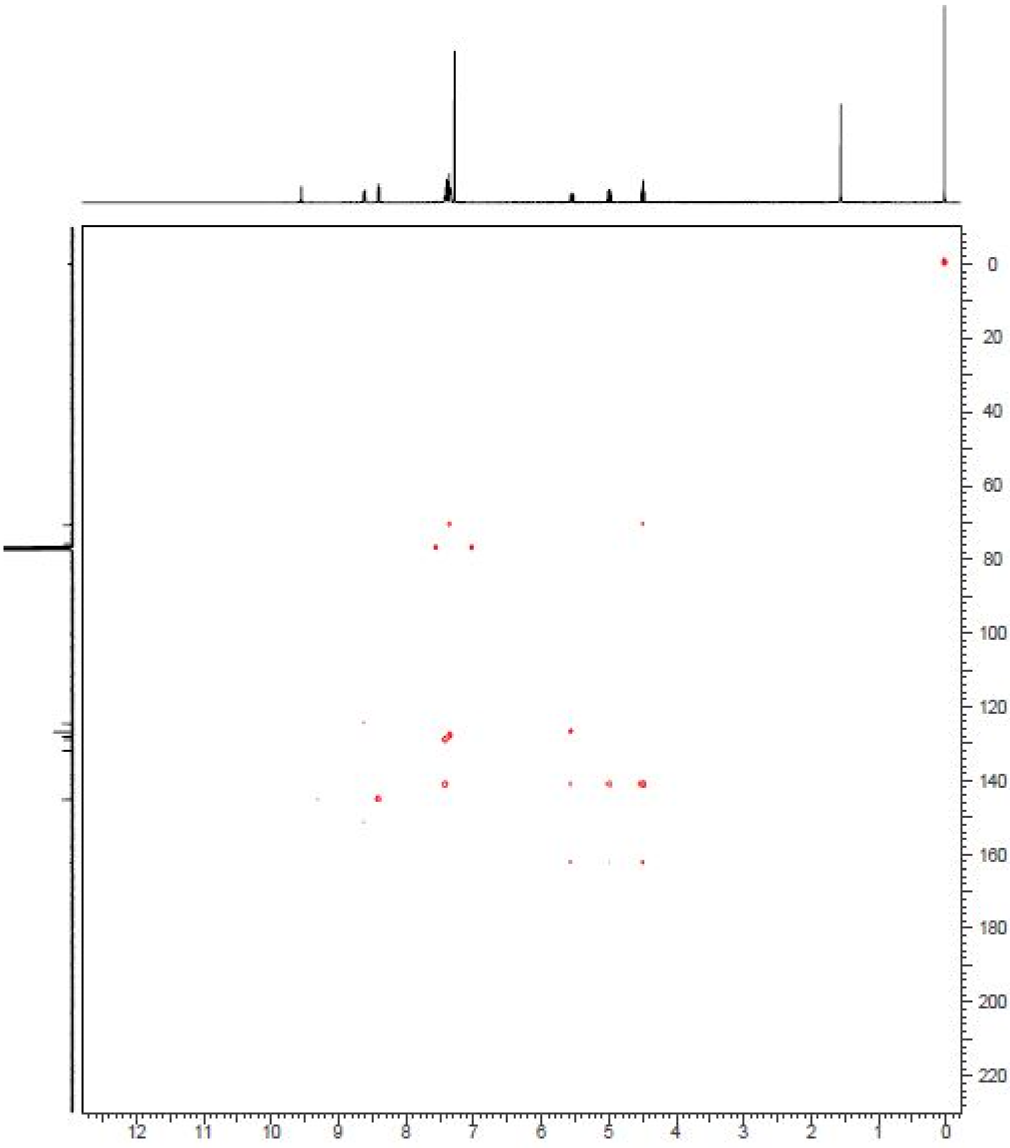

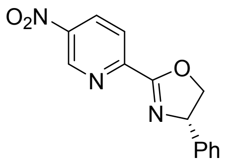

PyrOx **L5** [^{1}H,^{1}H]-ROESY (400 MHz, $CDCl_3$):

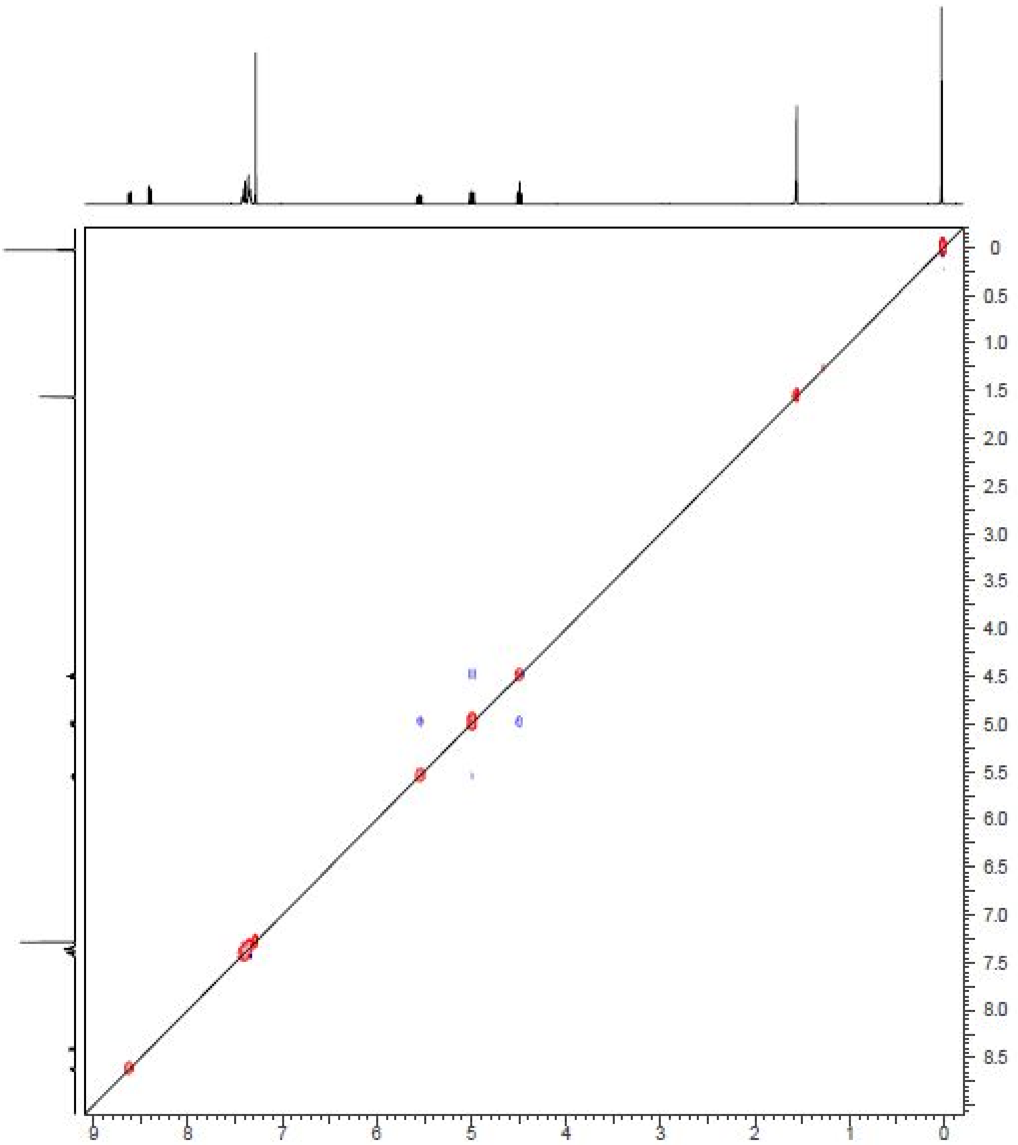

(3,4-dihydro-2*H*-pyran-2-yl)methyl benzoate

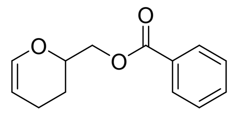

^{1}H NMR (400 MHz, $CDCl_3$):

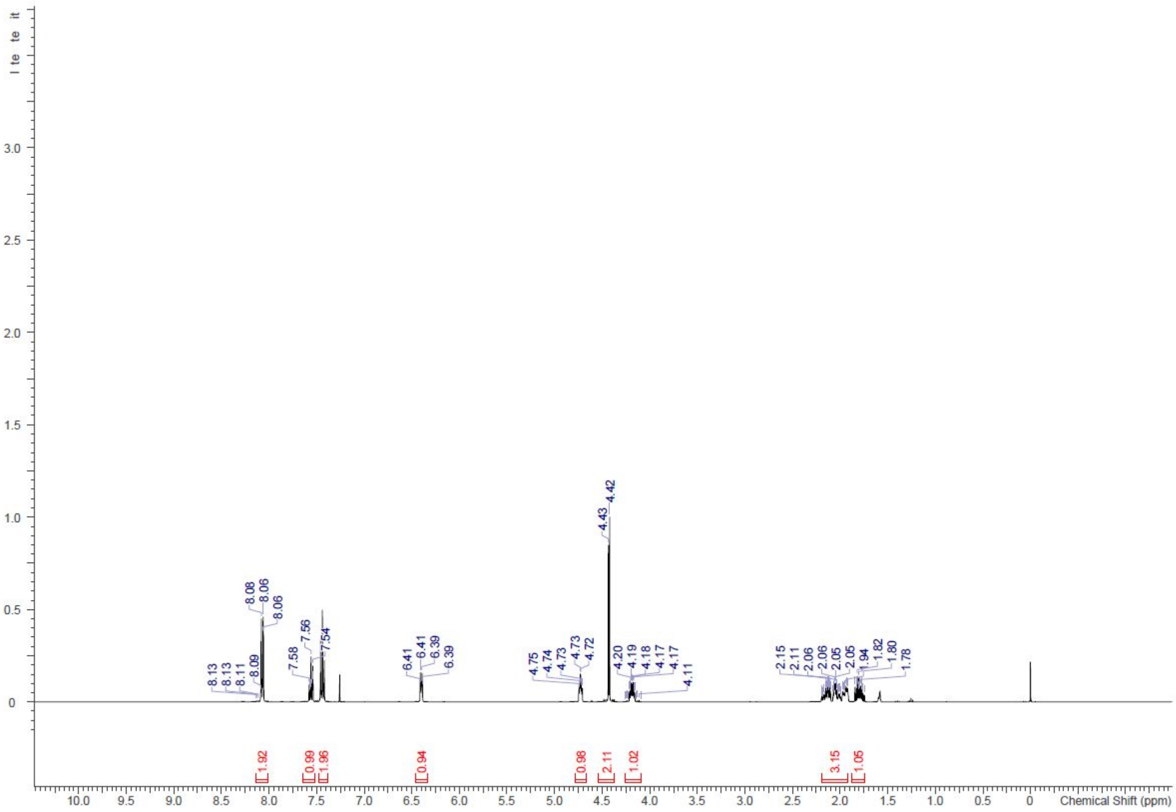

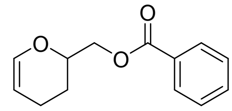

^{13}C NMR (101 MHz, $CDCl_3$):

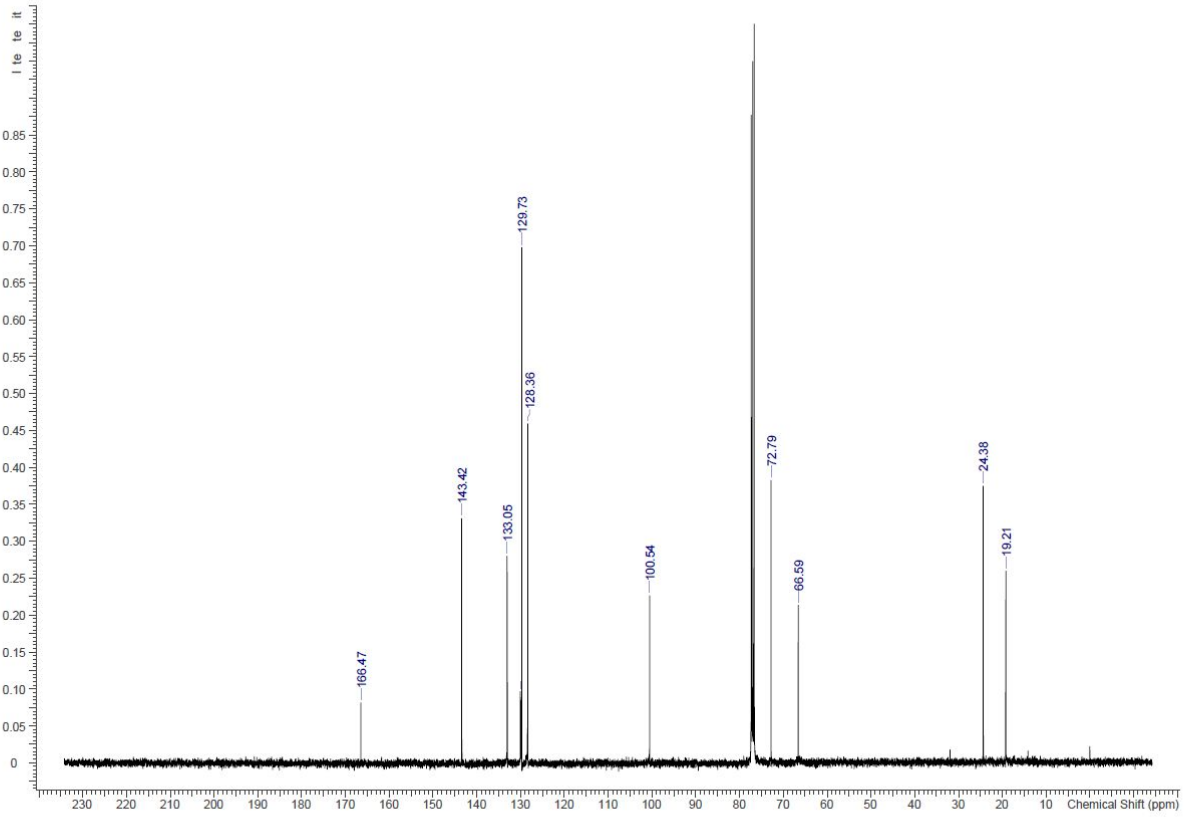

(3,4-dihydro-2*H*-pyran-2-yl)methanol, (*R*)-**1**

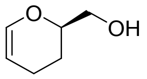

(R)-**1** ^{1}H NMR (400 MHz, $CDCl_3$):

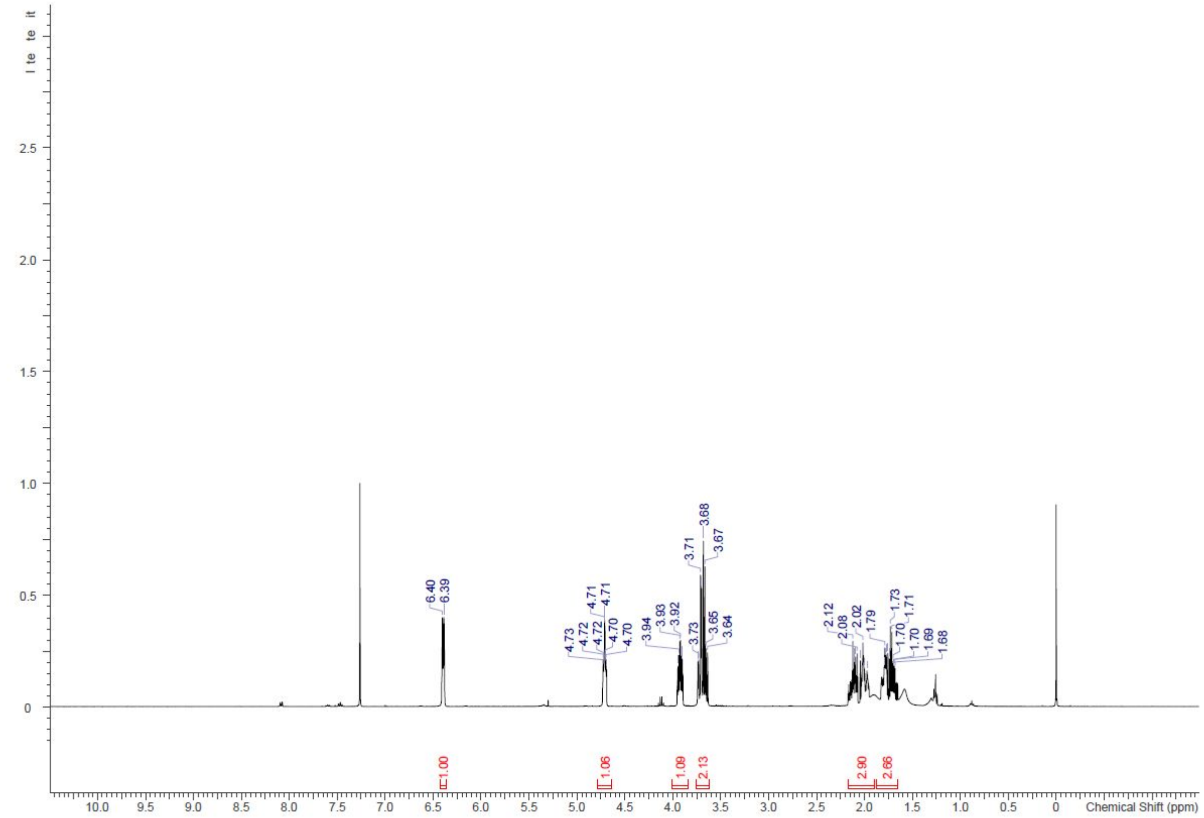

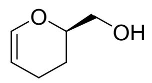

(R)-**1** ^{13}C NMR (101 MHz, $CDCl_3$):

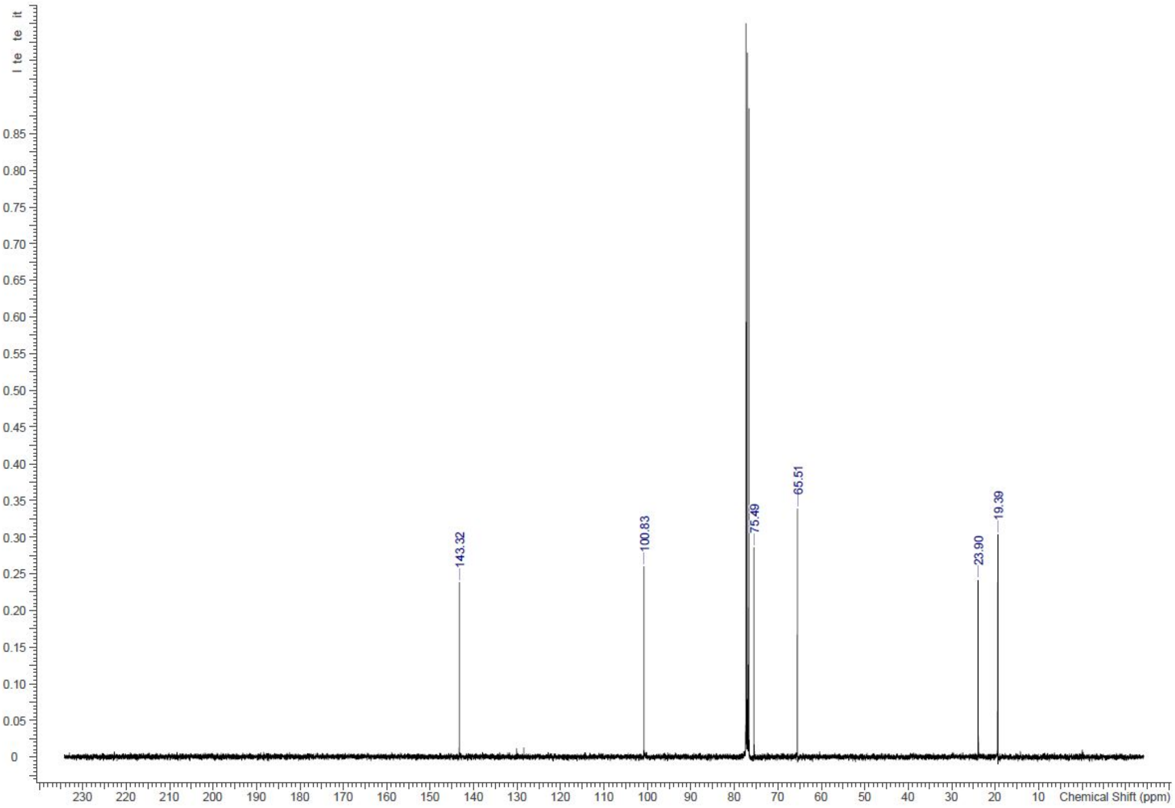

(3,4-dihydro-2*H*-pyran-2-yl)methyl acetate

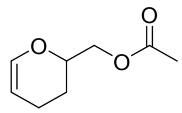

^{1}H NMR (400 MHz, $CDCl_3$):

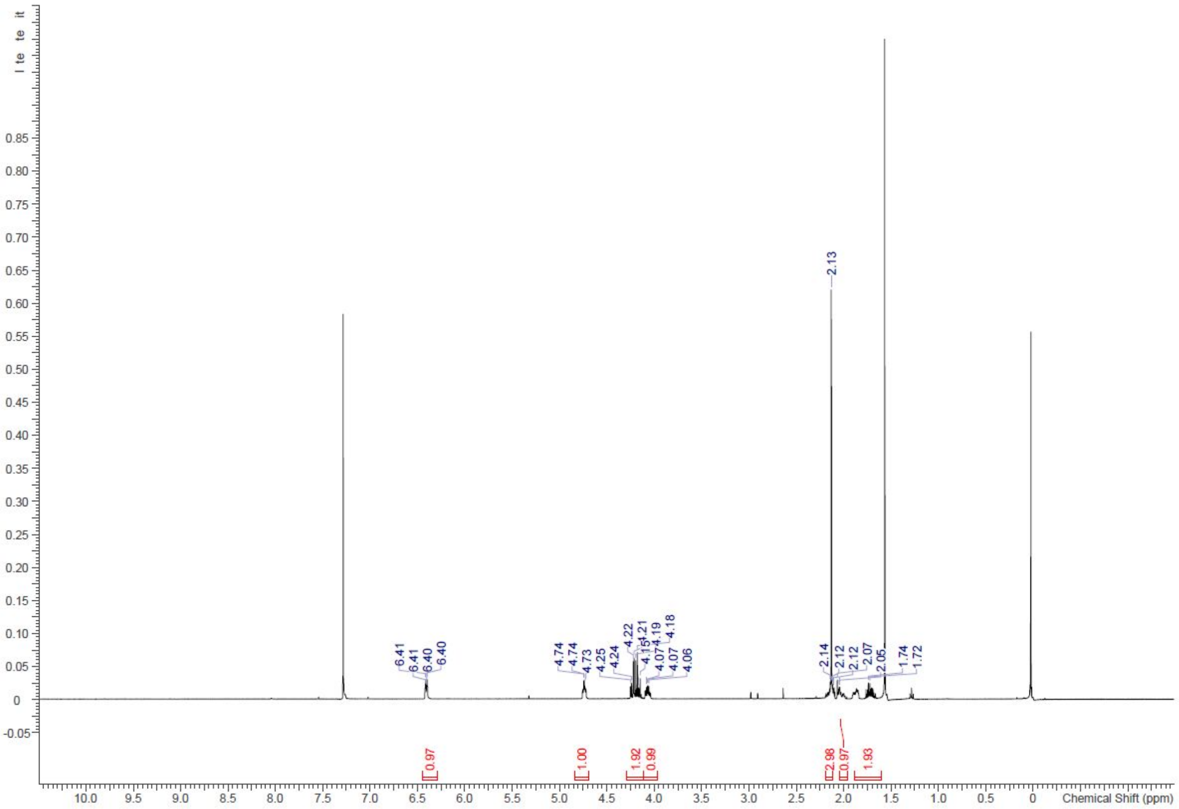

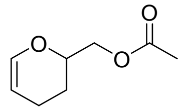

^{13}C NMR (101 MHz, $CDCl_3$):

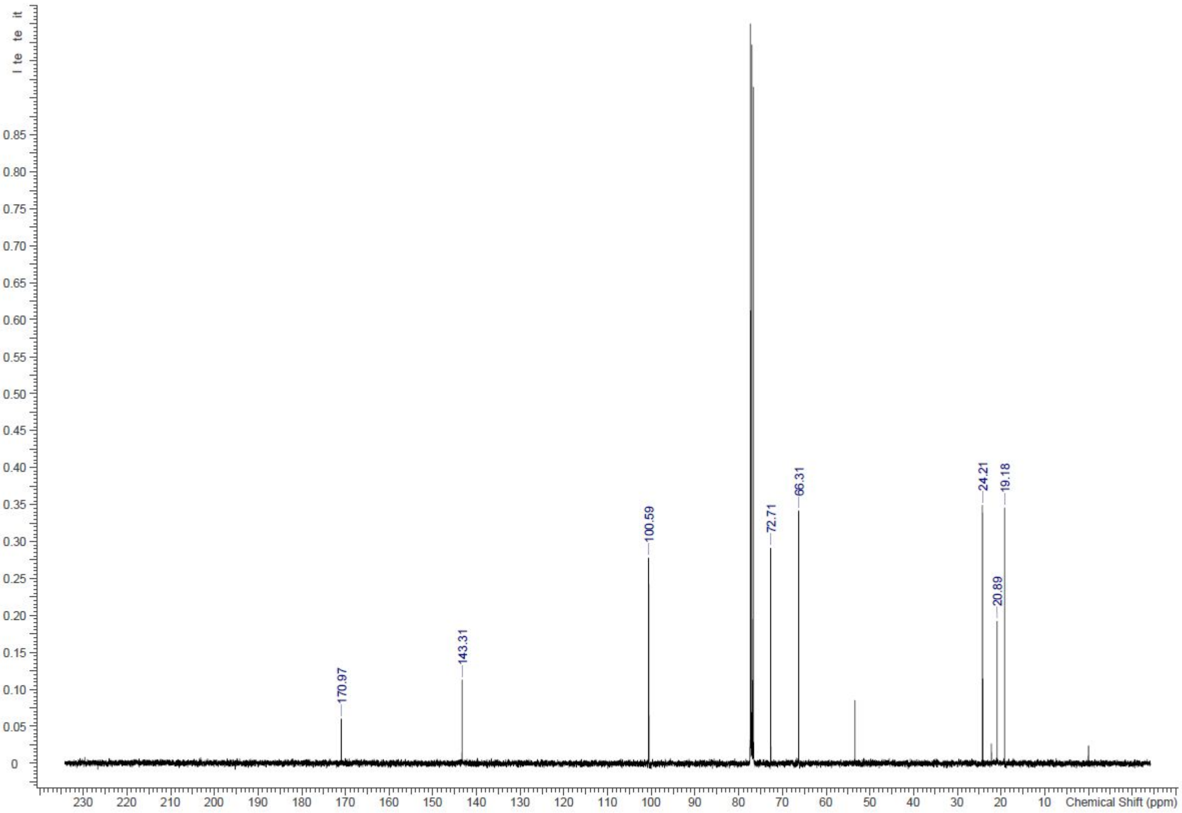

((2*R*,6*R*)-6-(4-fluorophenyl)tetrahydro-2*H*-pyran-2-yl)methanol, **3a**

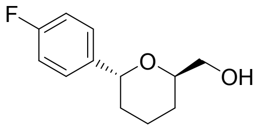

3a ^{1}H NMR (400 MHz, $CDCl_3$):

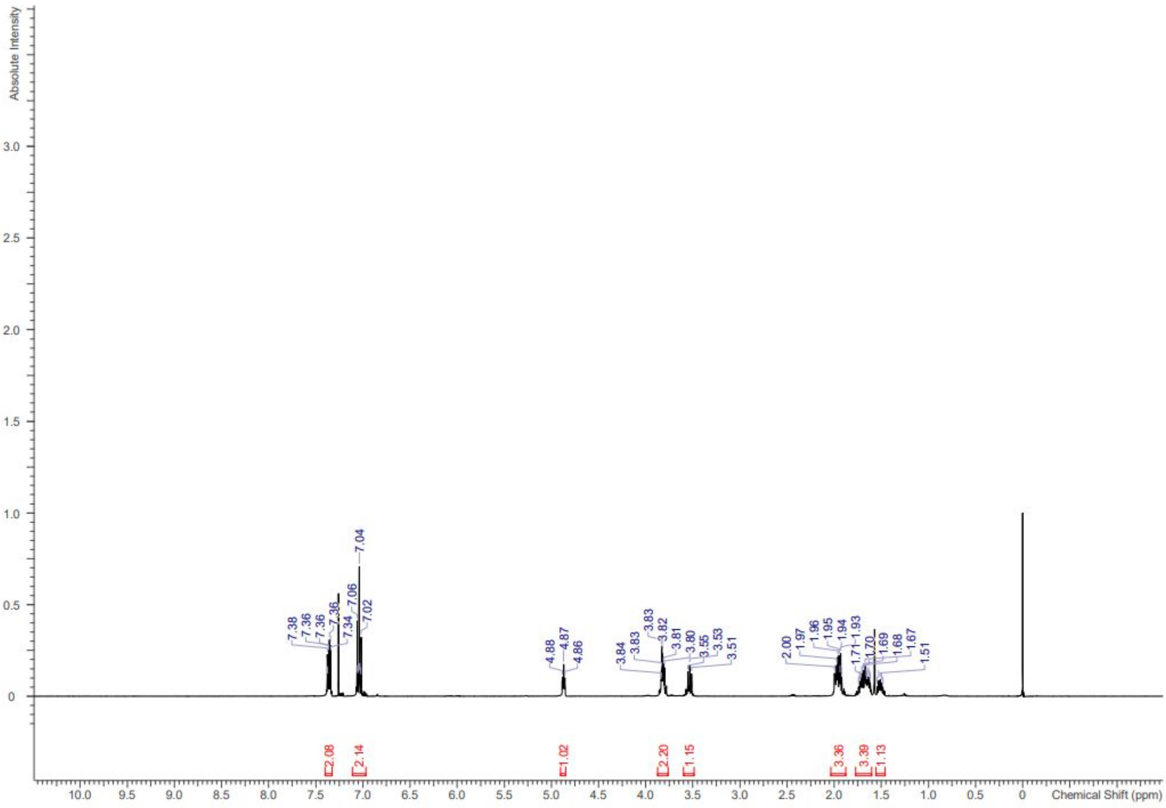

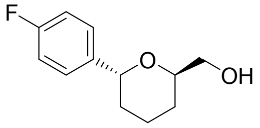

3a ^{13}C NMR (101 MHz, $CDCl_3$):

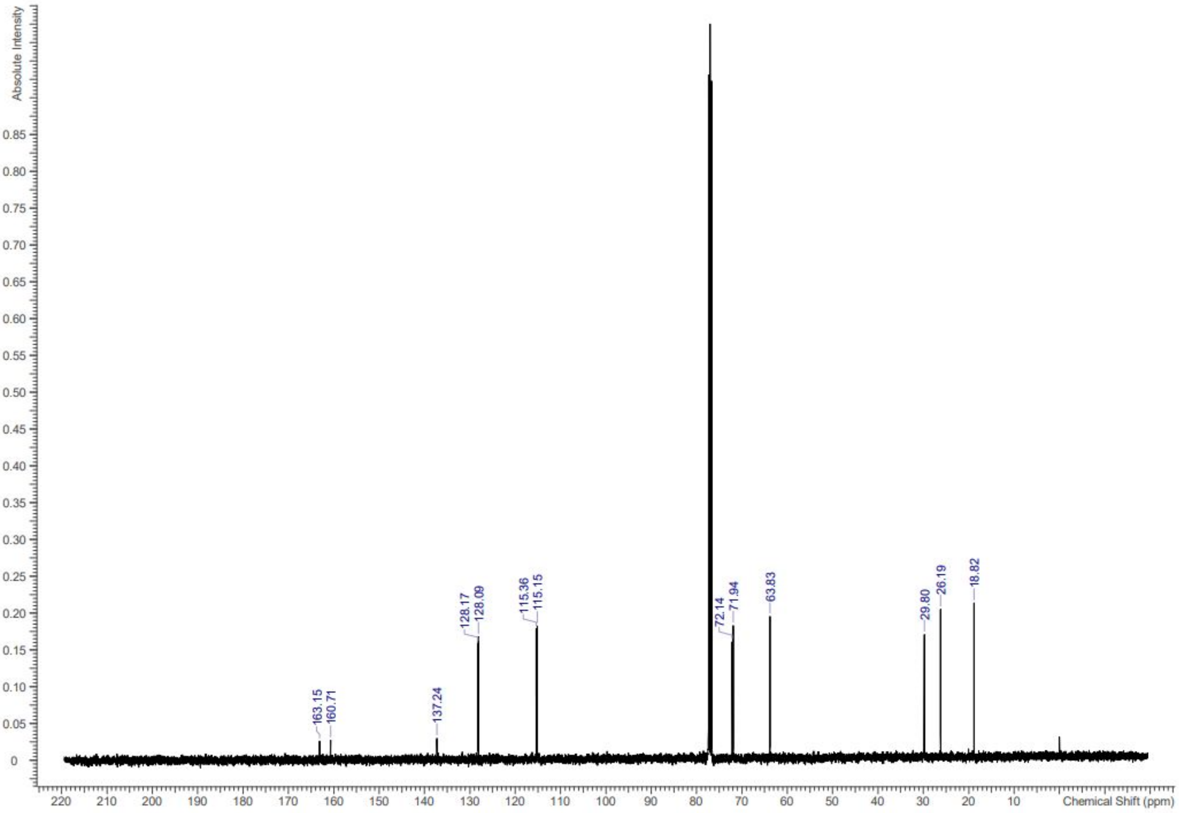

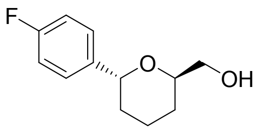

3a $^{19}F\{^{1}H\}$ NMR (376 MHz, $CDCl_3$):

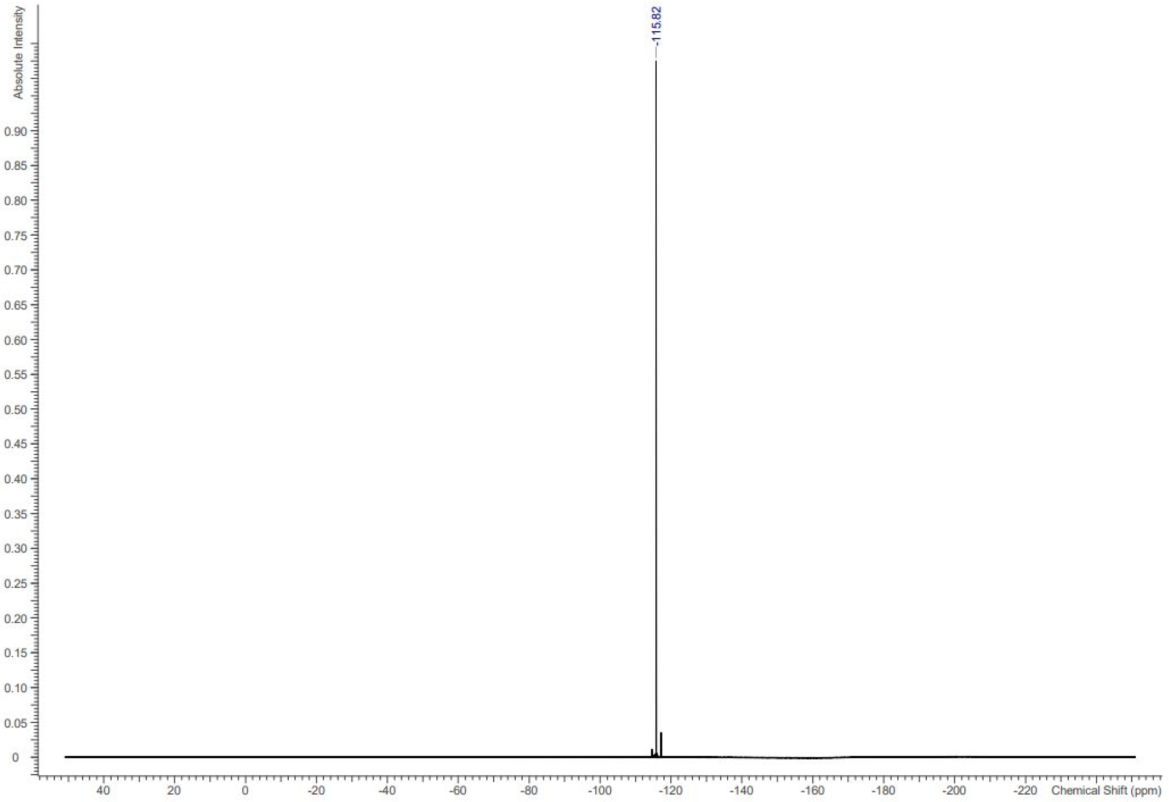

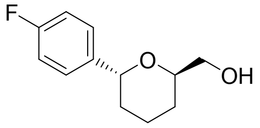

3a [^{1}H,^{1}H]-COSY (400 MHz, $CDCl_3$):

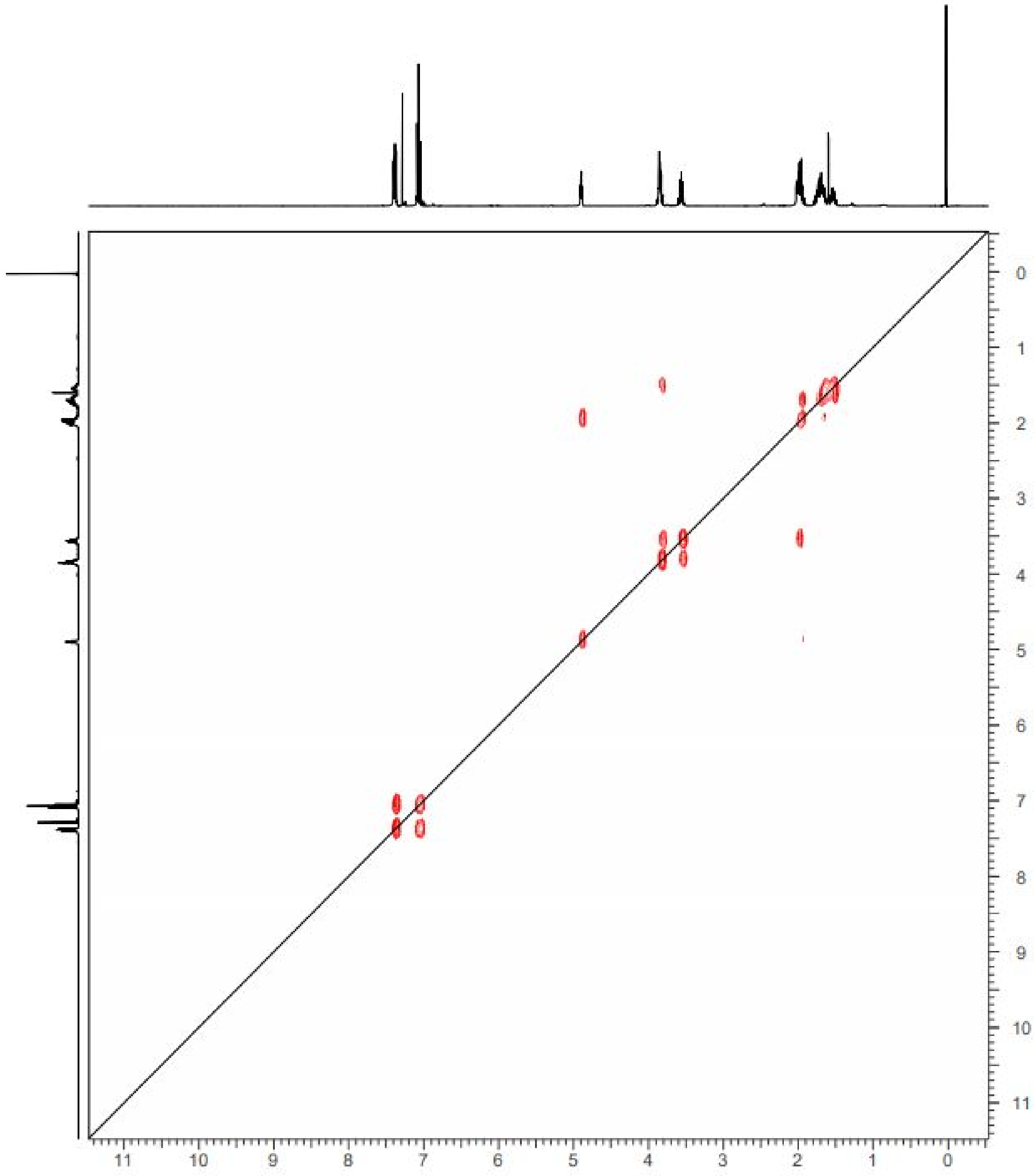

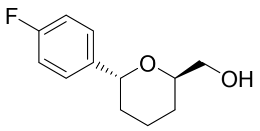

3a [^{1}H,^{13}C]-HSQC (400 MHz, 101 MHz, $CDCl_3$):

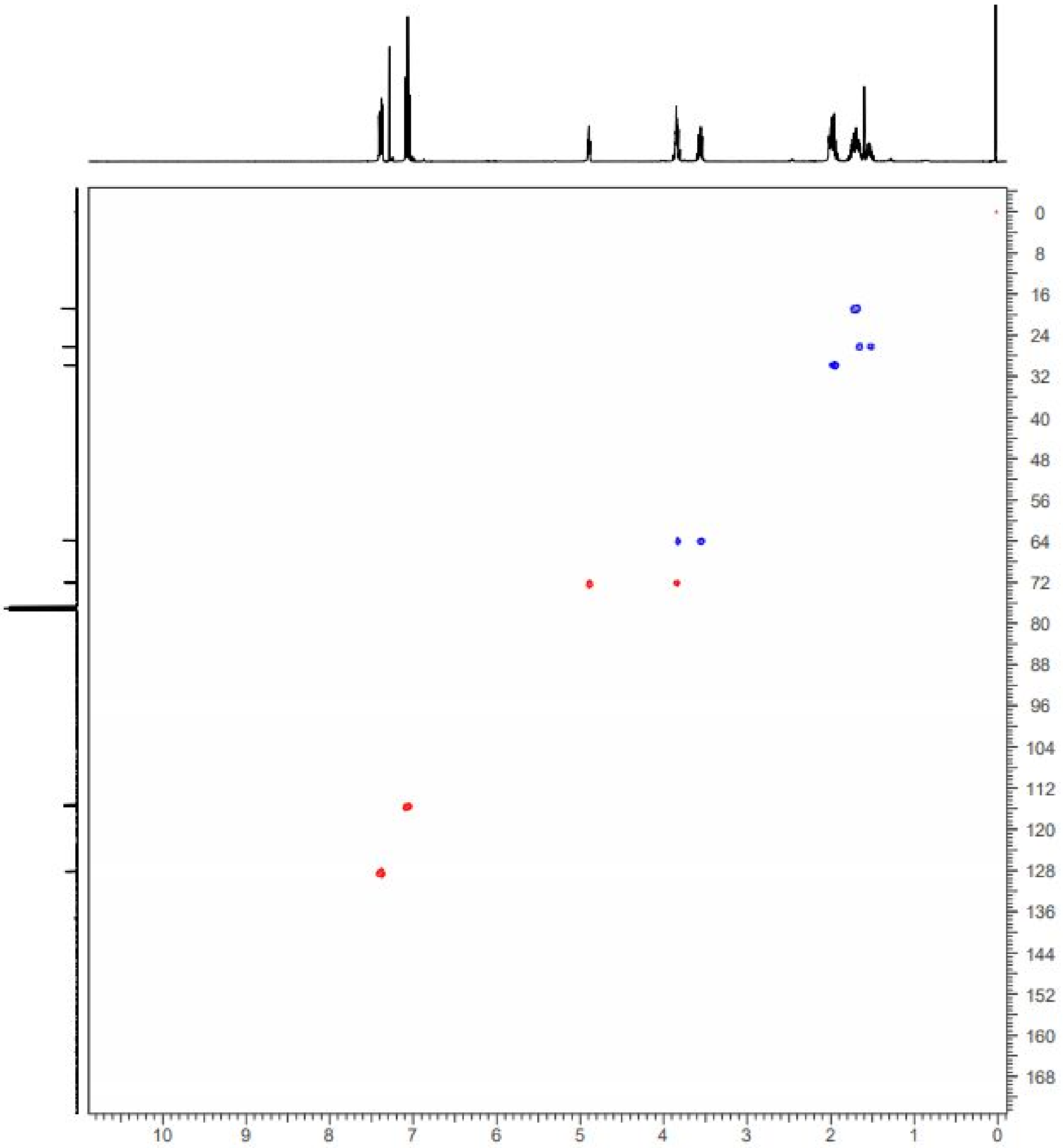

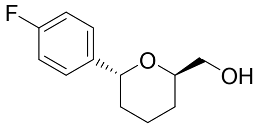

3a [^{1}H,^{13}C]-HMBC (400 MHz, 101 MHz, $CDCl_3$):

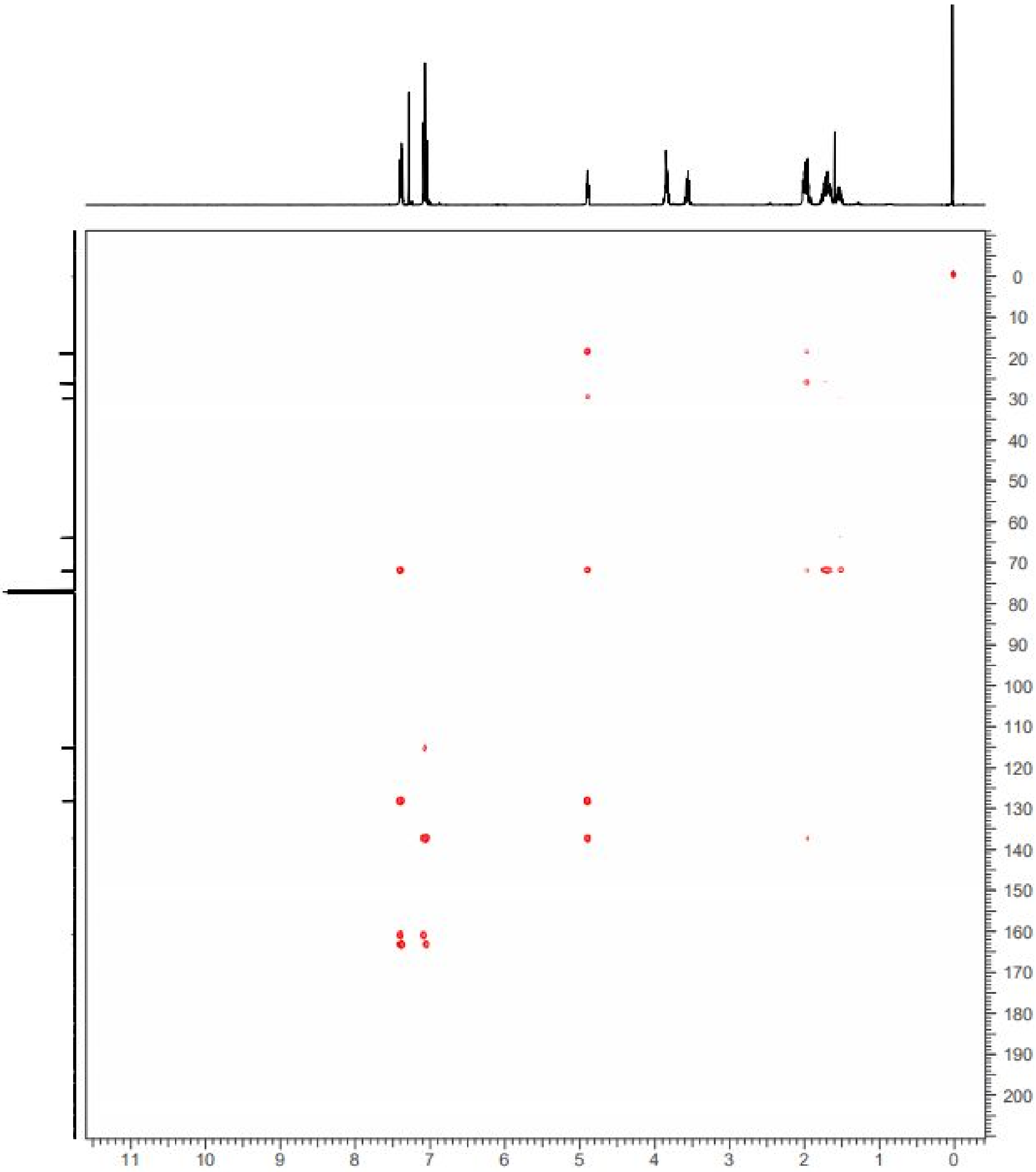

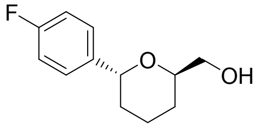

3a [1H,1H]-ROESY (400 MHz, $CDCl_3$):

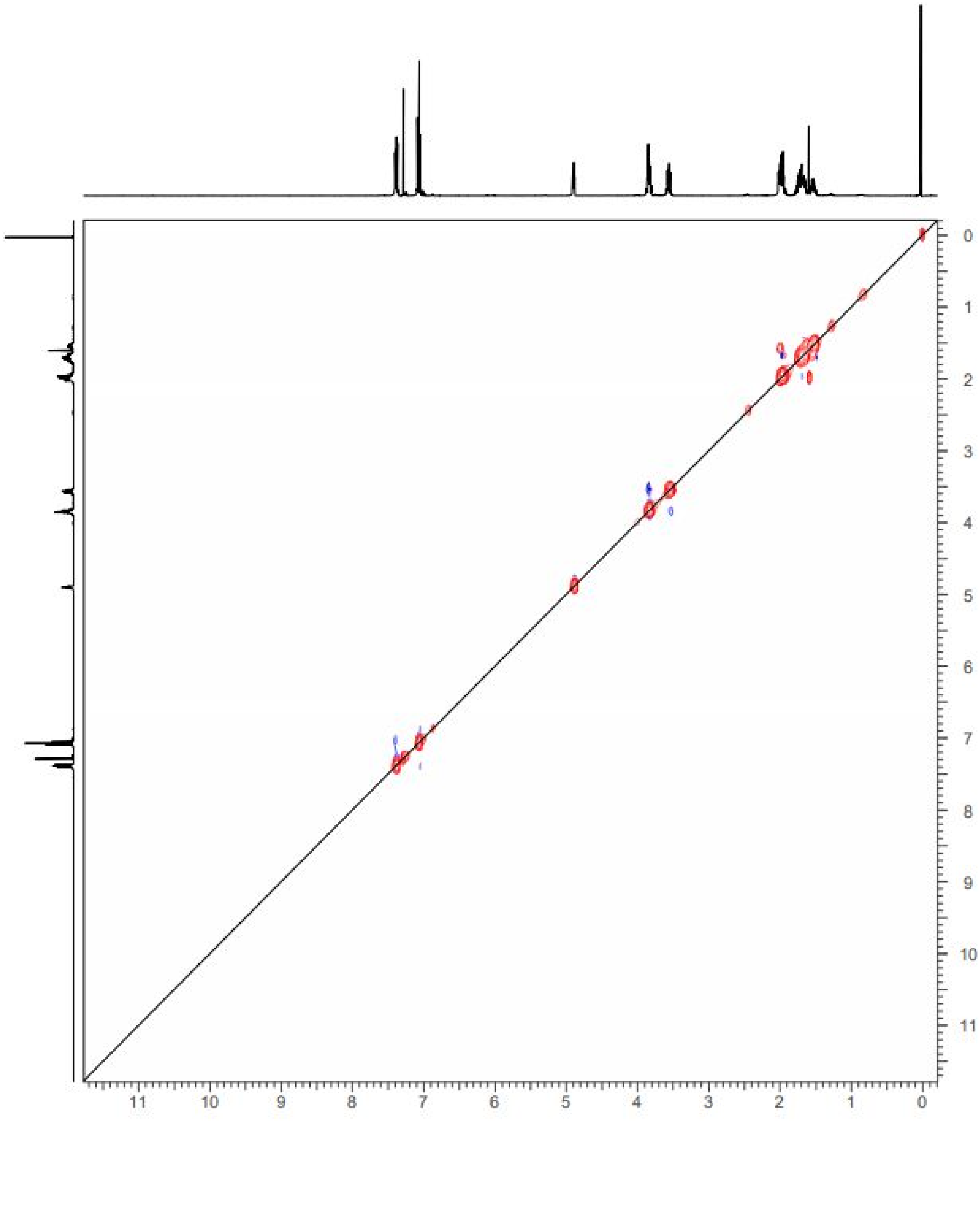

Ferrocenoyl derivative **Fc-3a**:

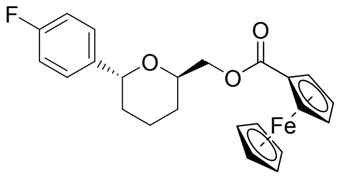

Fc-3a ^{1}H NMR (400 MHz, $CDCl_3$):

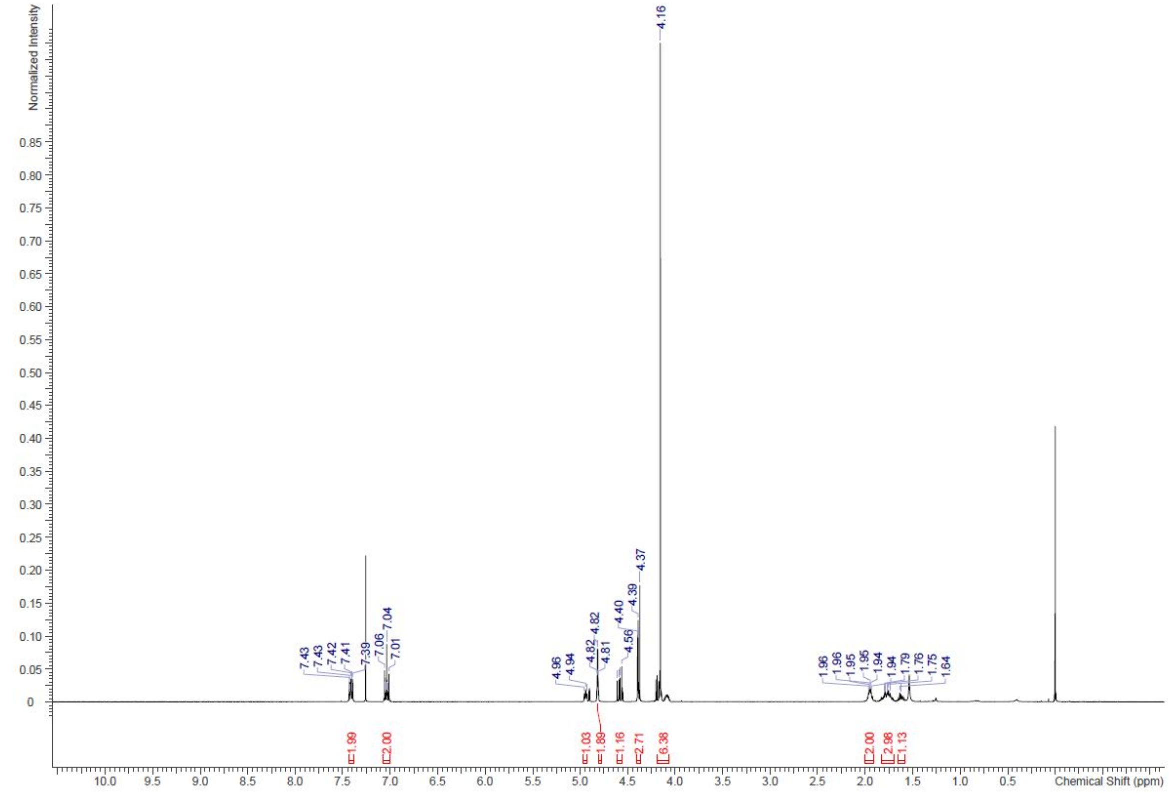

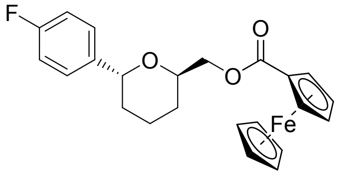

Fc-3a ^{13}C NMR (101 MHz, $CDCl_3$):

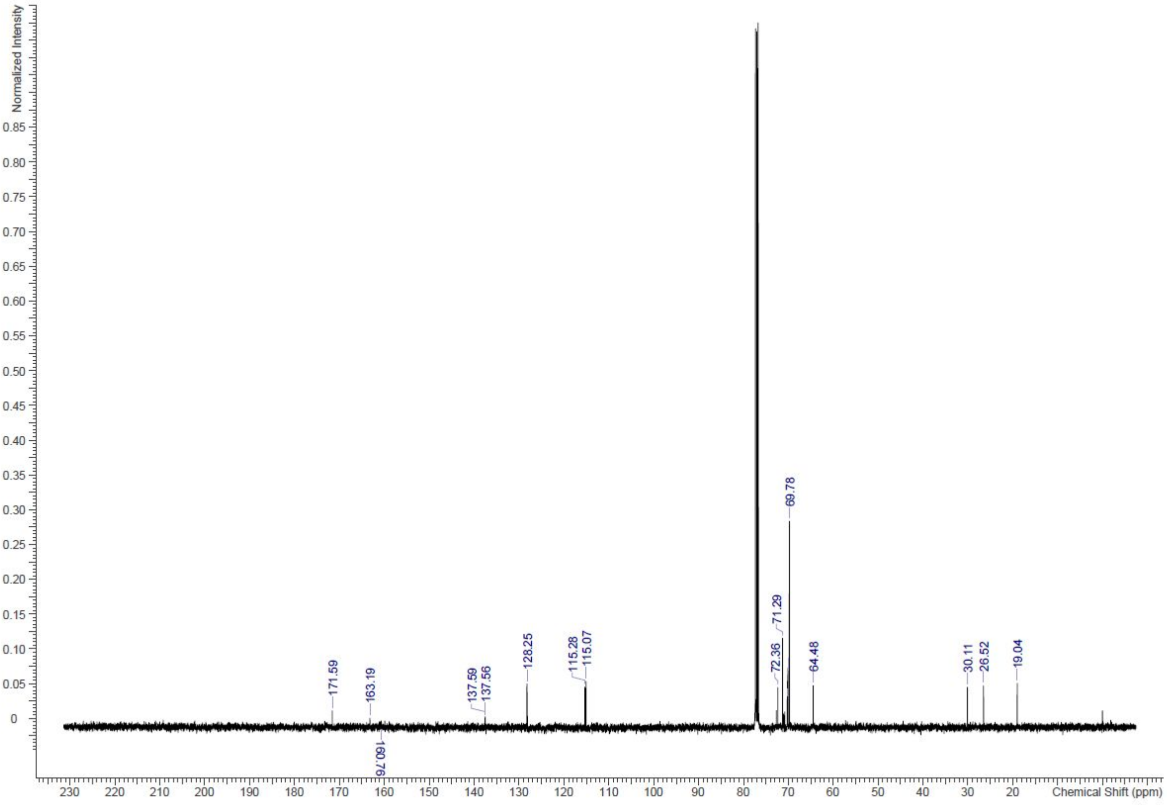

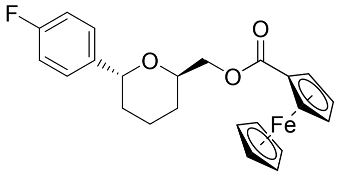

Fc-3a $^{19}F\{^{1}H\}$ NMR (376 MHz, $CDCl_3$):

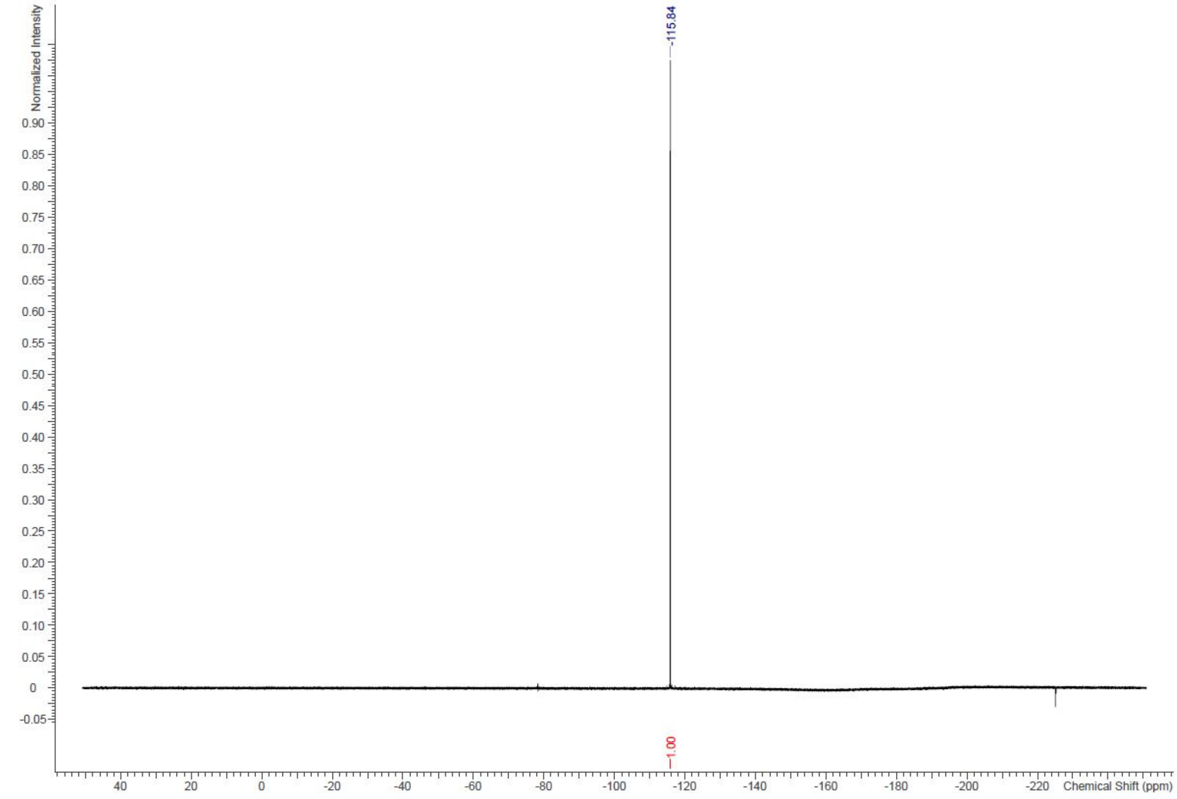

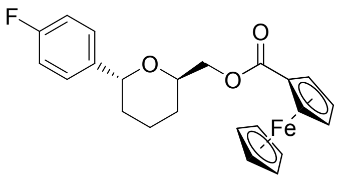

Fc-3a [^{1}H,^{1}H]-COSY (400 MHz, $CDCl_3$):

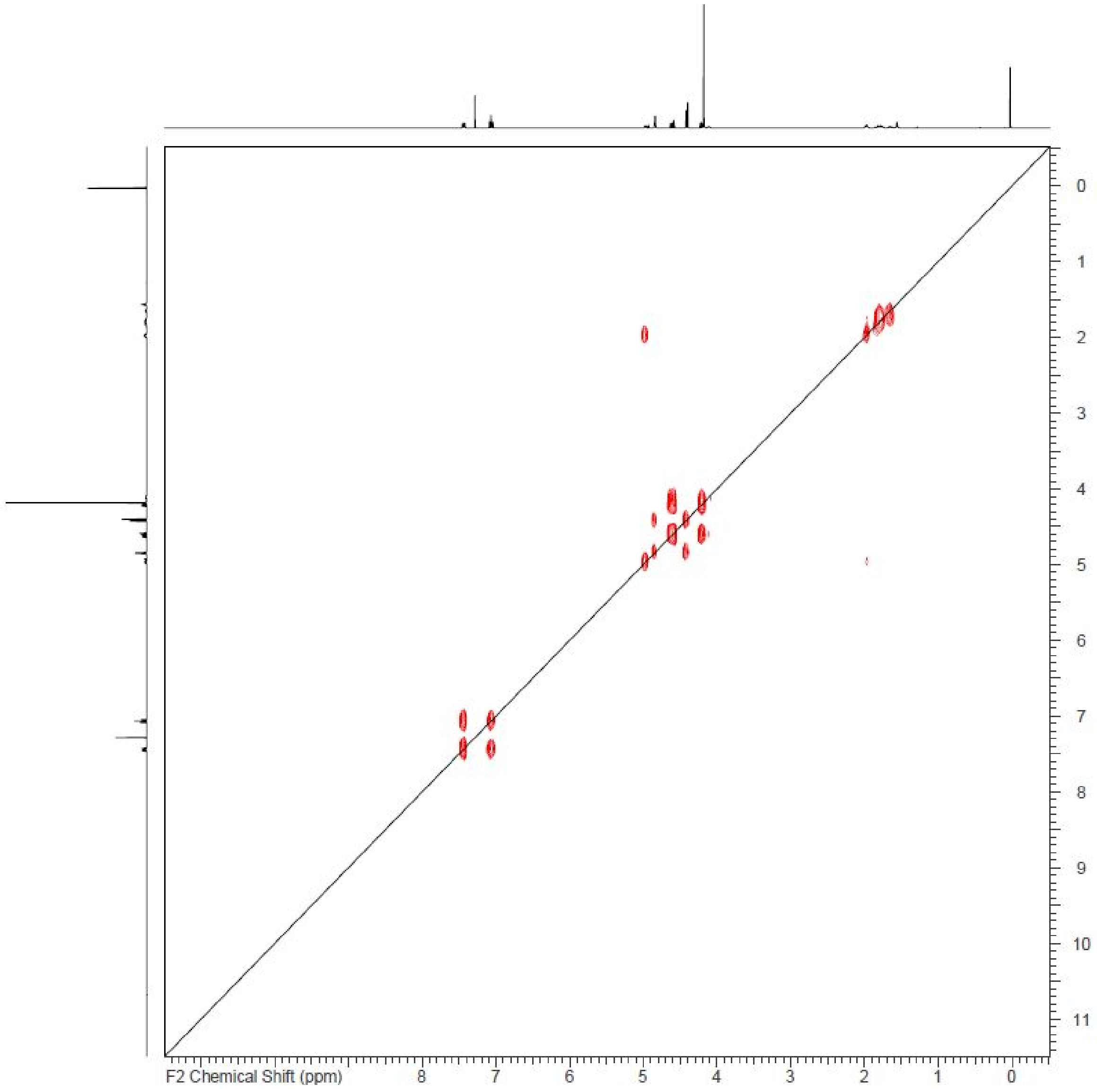

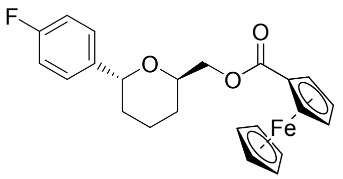

Fc-3a [^{1}H,^{13}C]-HSQC (400 MHz, 101 MHz, $CDCl_3$):

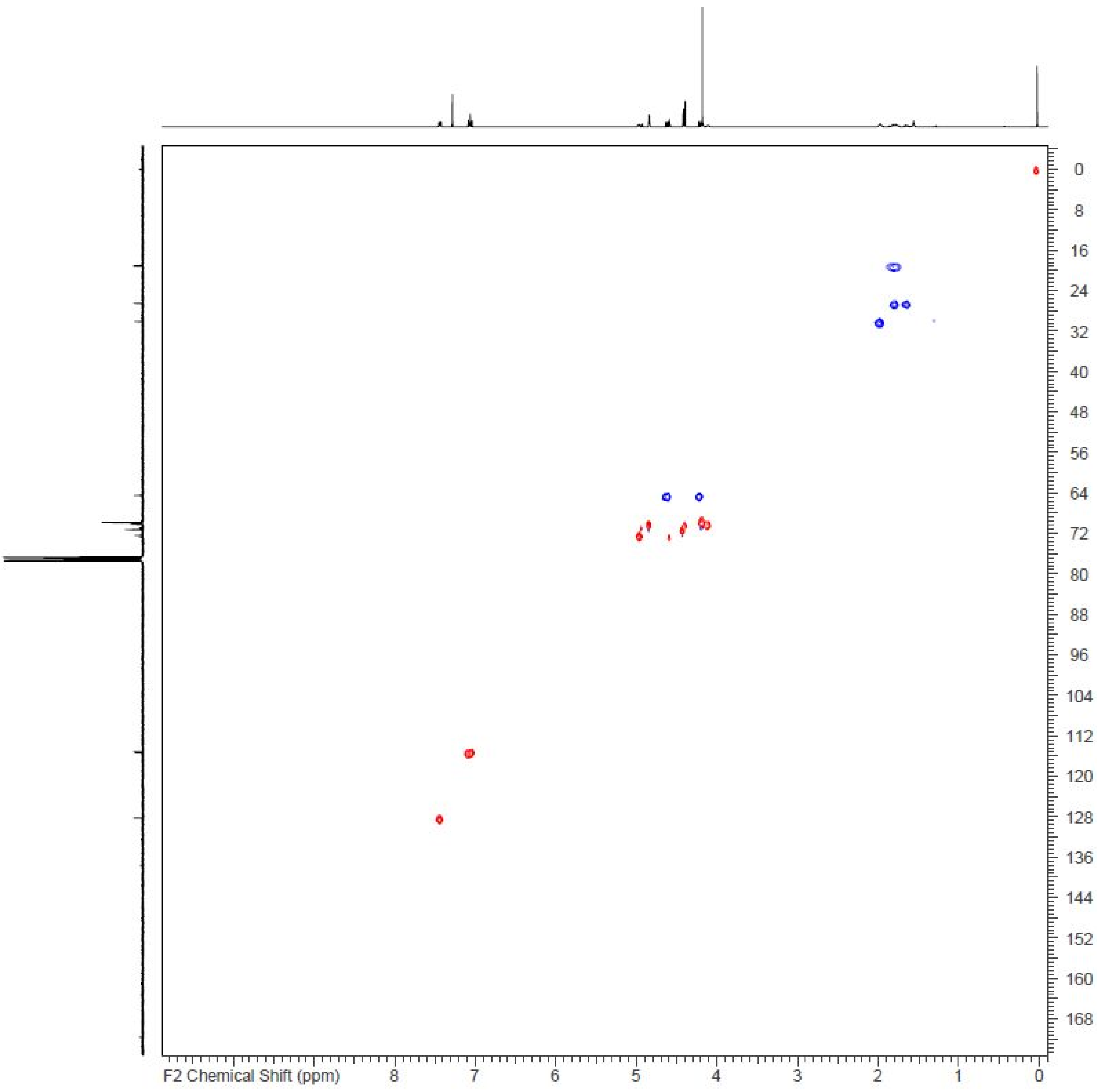

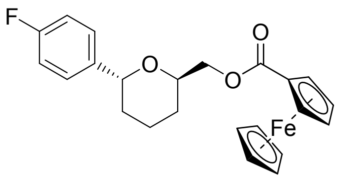

Fc-3a [^{1}H,^{13}C]-HMBC (400 MHz, 101 MHz, $CDCl_3$):

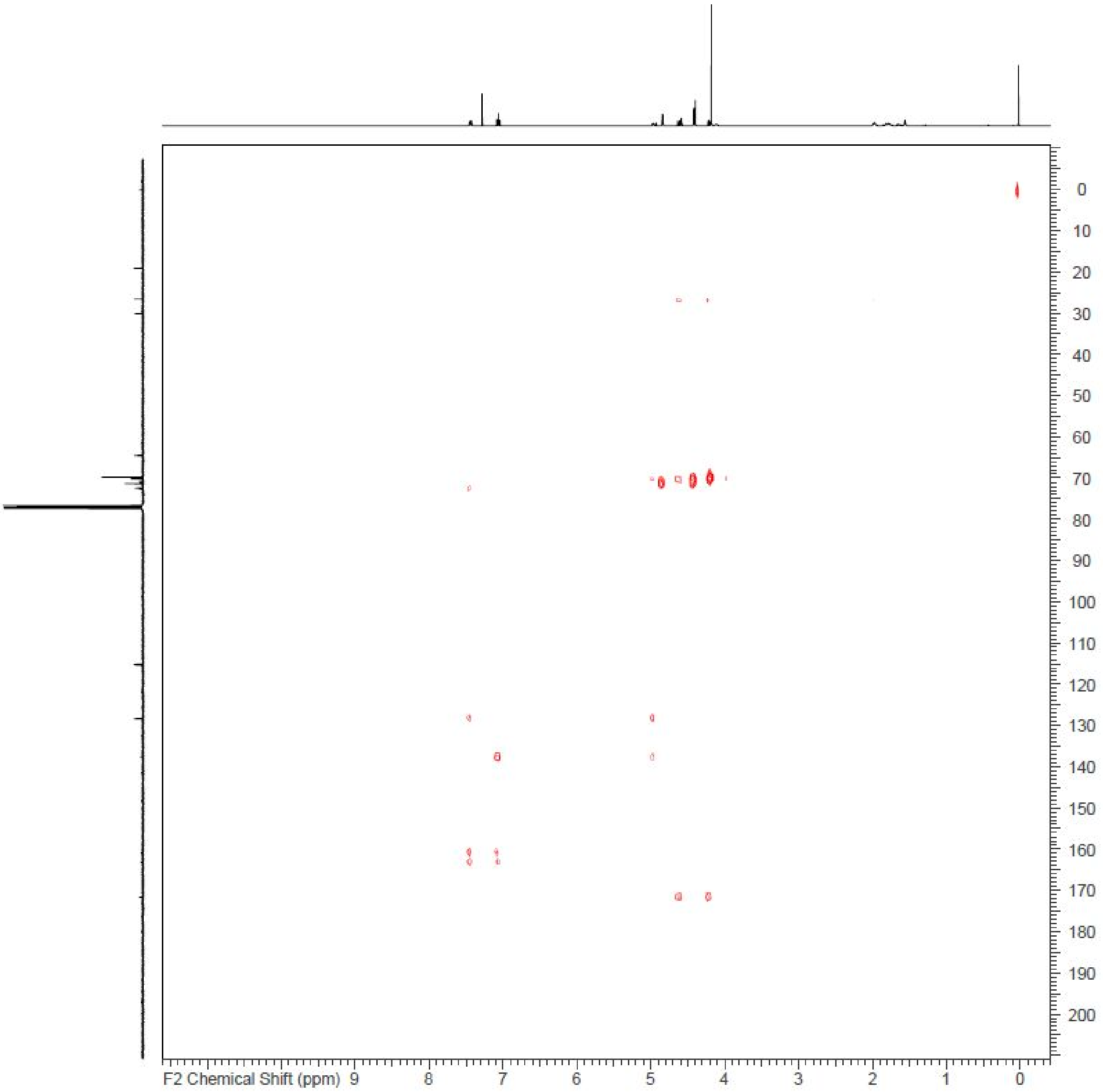

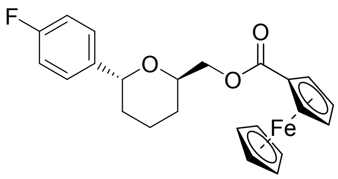

Fc-3a [^{1}H,^{1}H]-ROESY (400 MHz, $CDCl_3$):

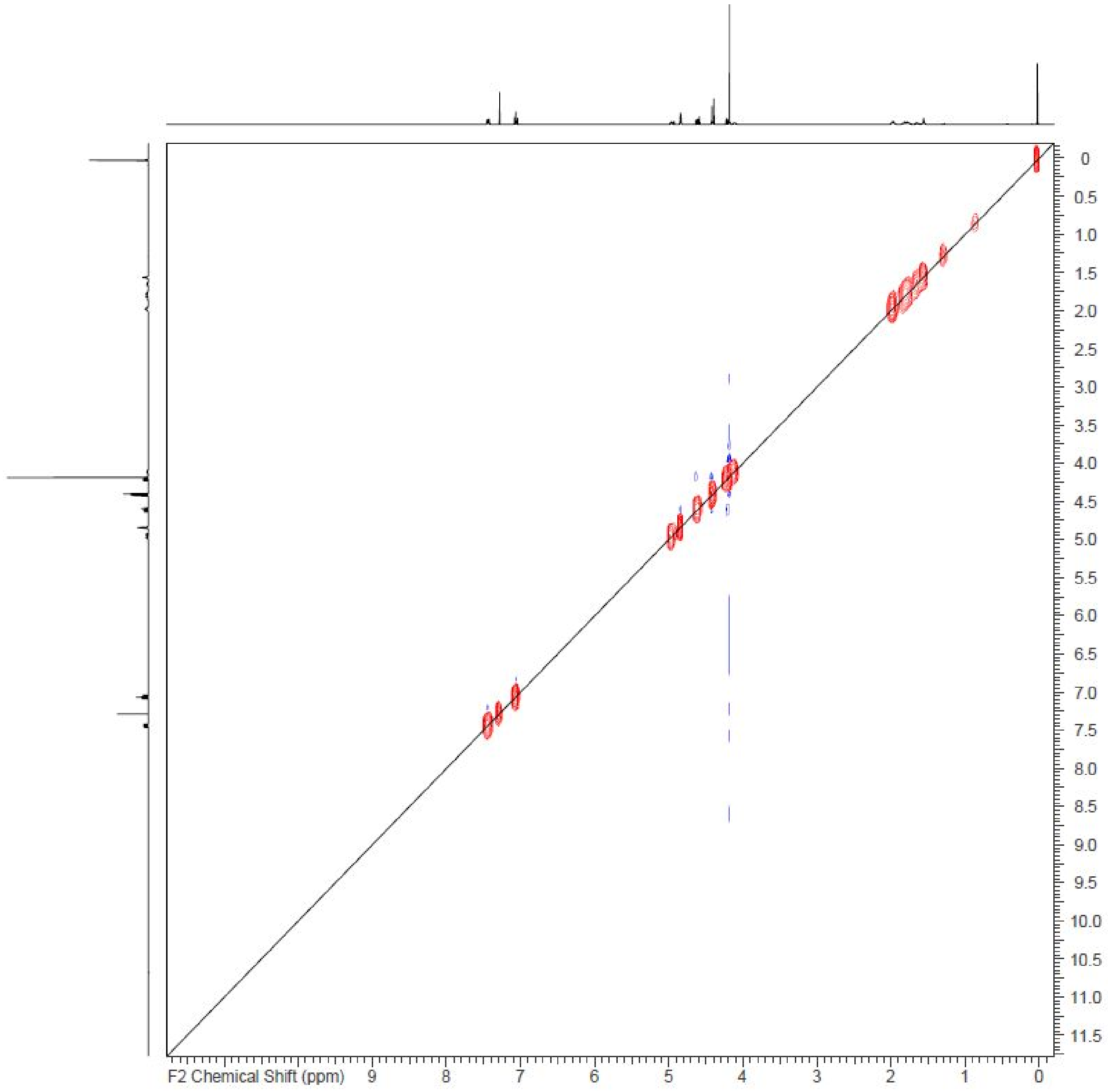

((2*R*,6*R*)-6-(3-fluorophenyl)tetrahydro-2*H*-pyran-2-yl)methanol, **3b**

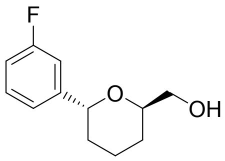

3b ^{1}H NMR (400 MHz, $CDCl_3$):

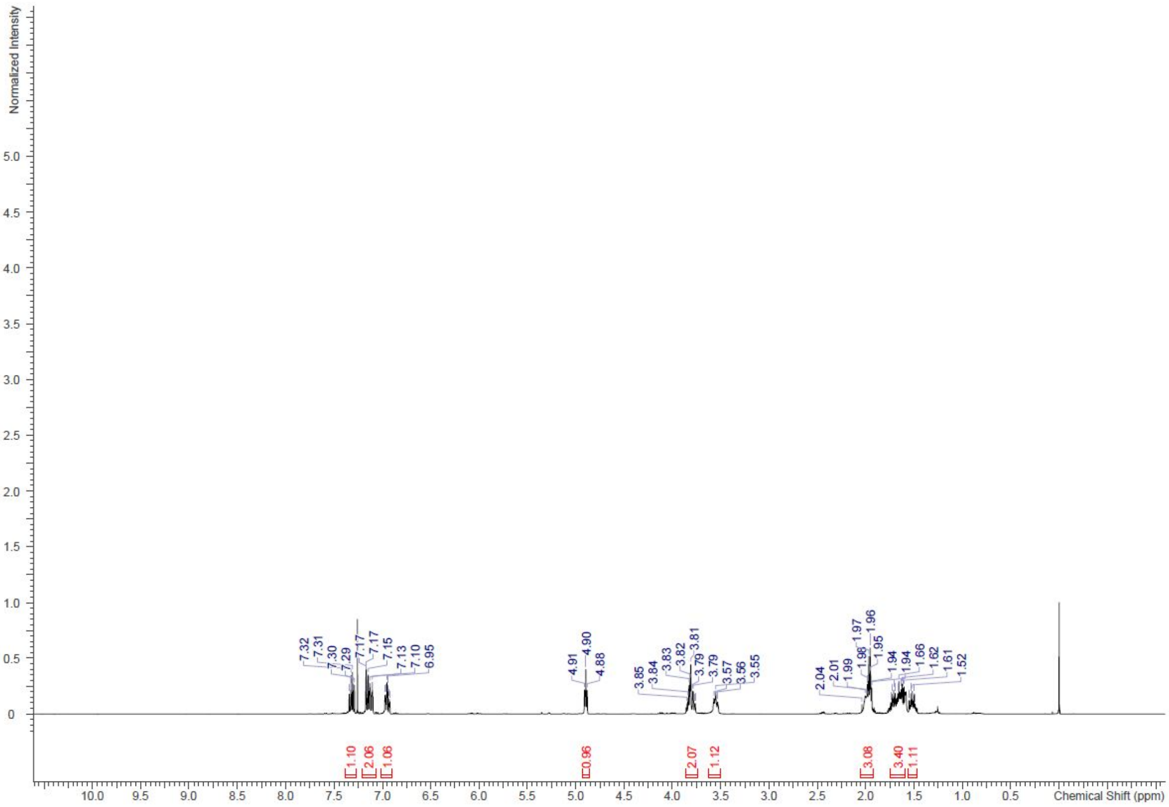

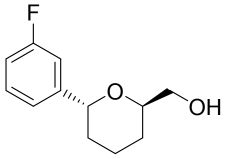

3b ^{13}C NMR (101 MHz, $CDCl_3$):

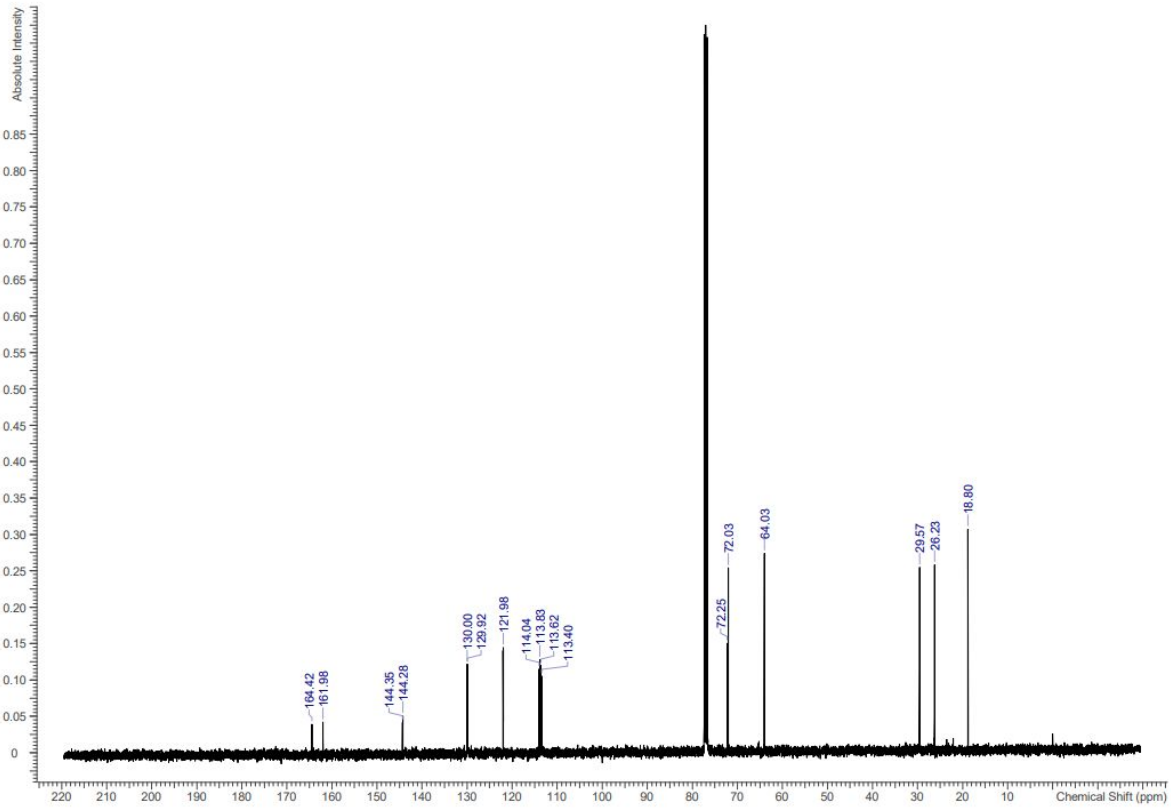

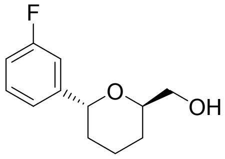

3b $^{19}F\{^{1}H\}$ NMR (376 MHz, $CDCl_3$):

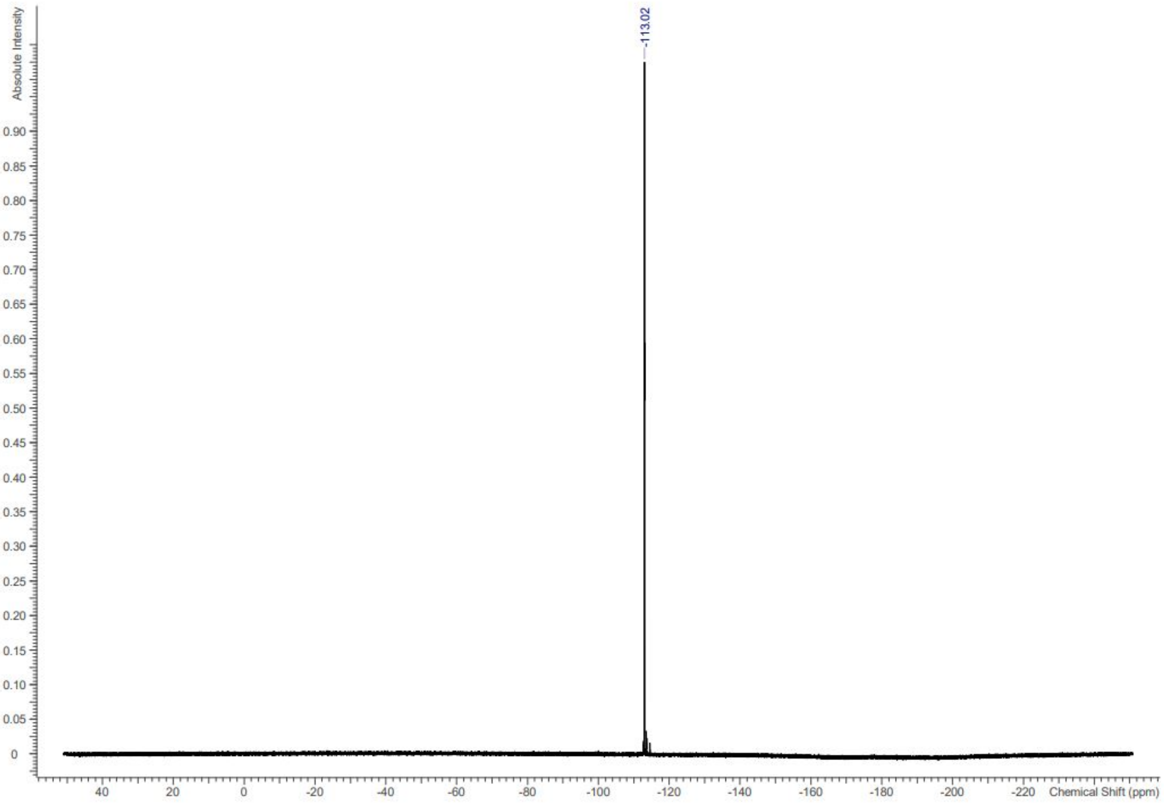

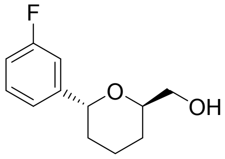

3b [^{1}H,^{1}H]-COSY (400 MHz, $CDCl_3$):

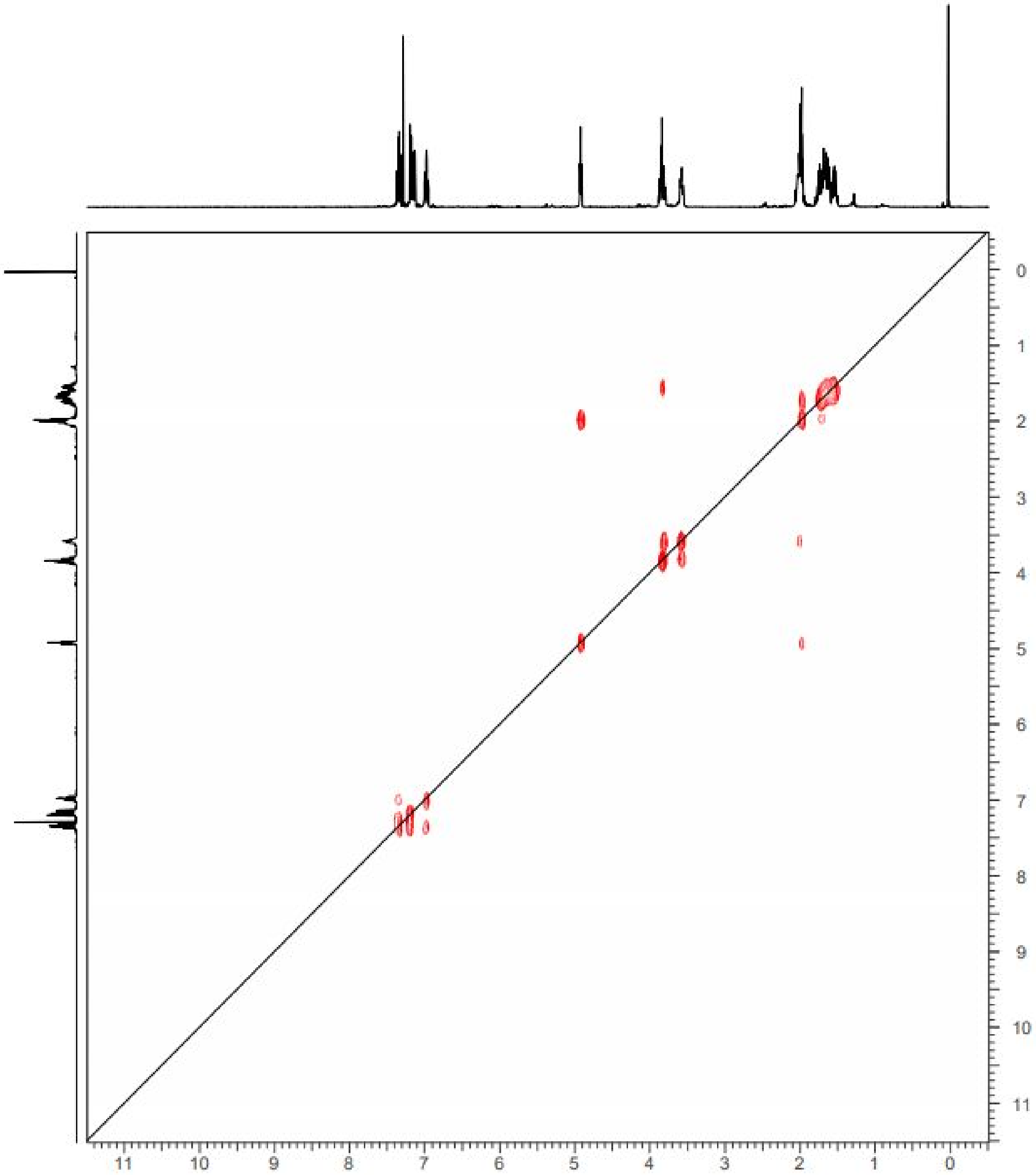

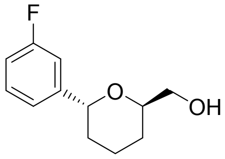

3b [^{1}H,^{13}C]-HSQC (400 MHz, 101 MHz, $CDCl_3$):

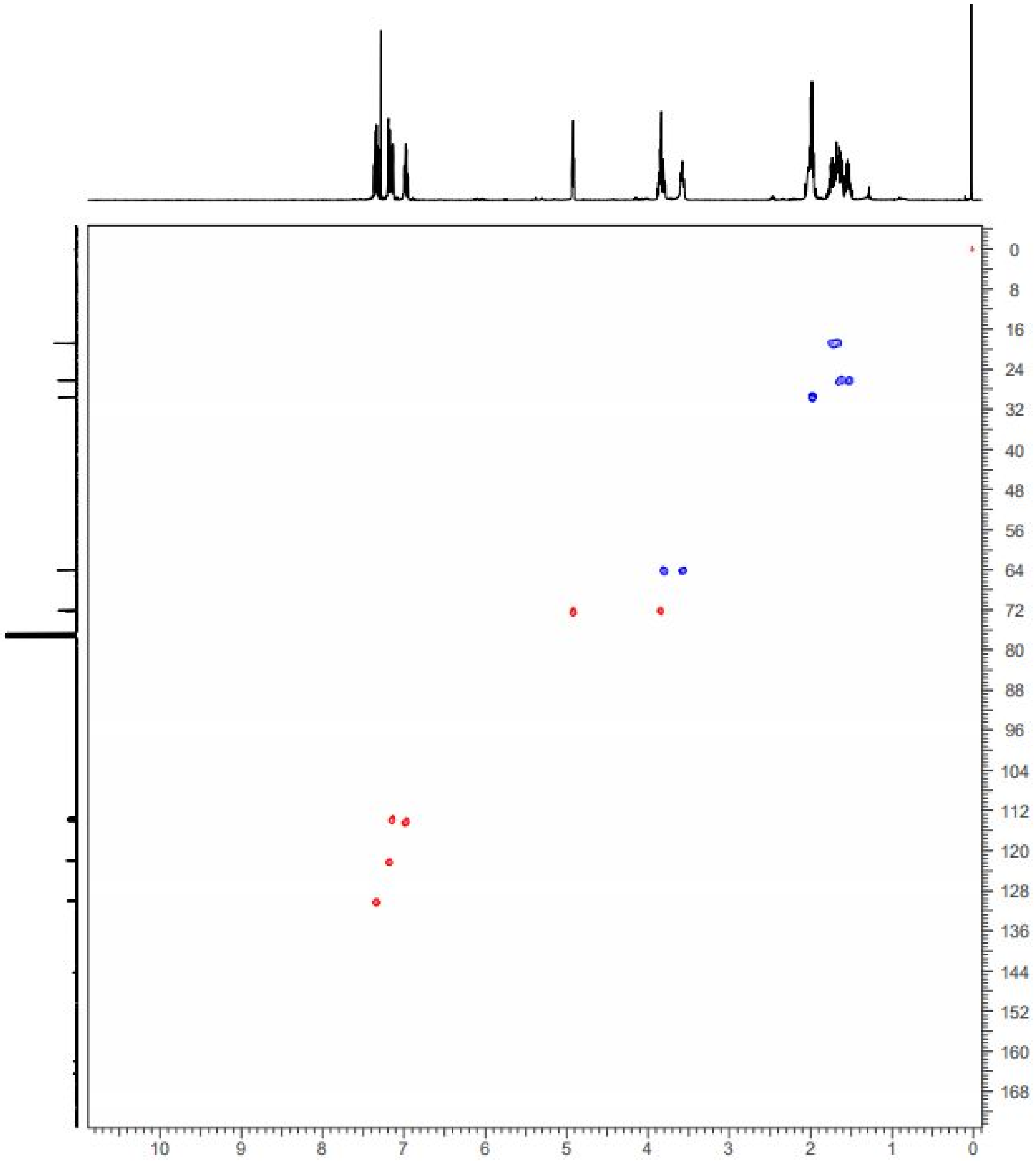

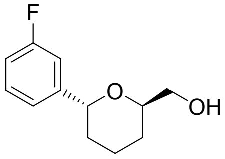

3b [^{1}H,^{13}C]-HMBC (400 MHz, 101 MHz, $CDCl_3$):

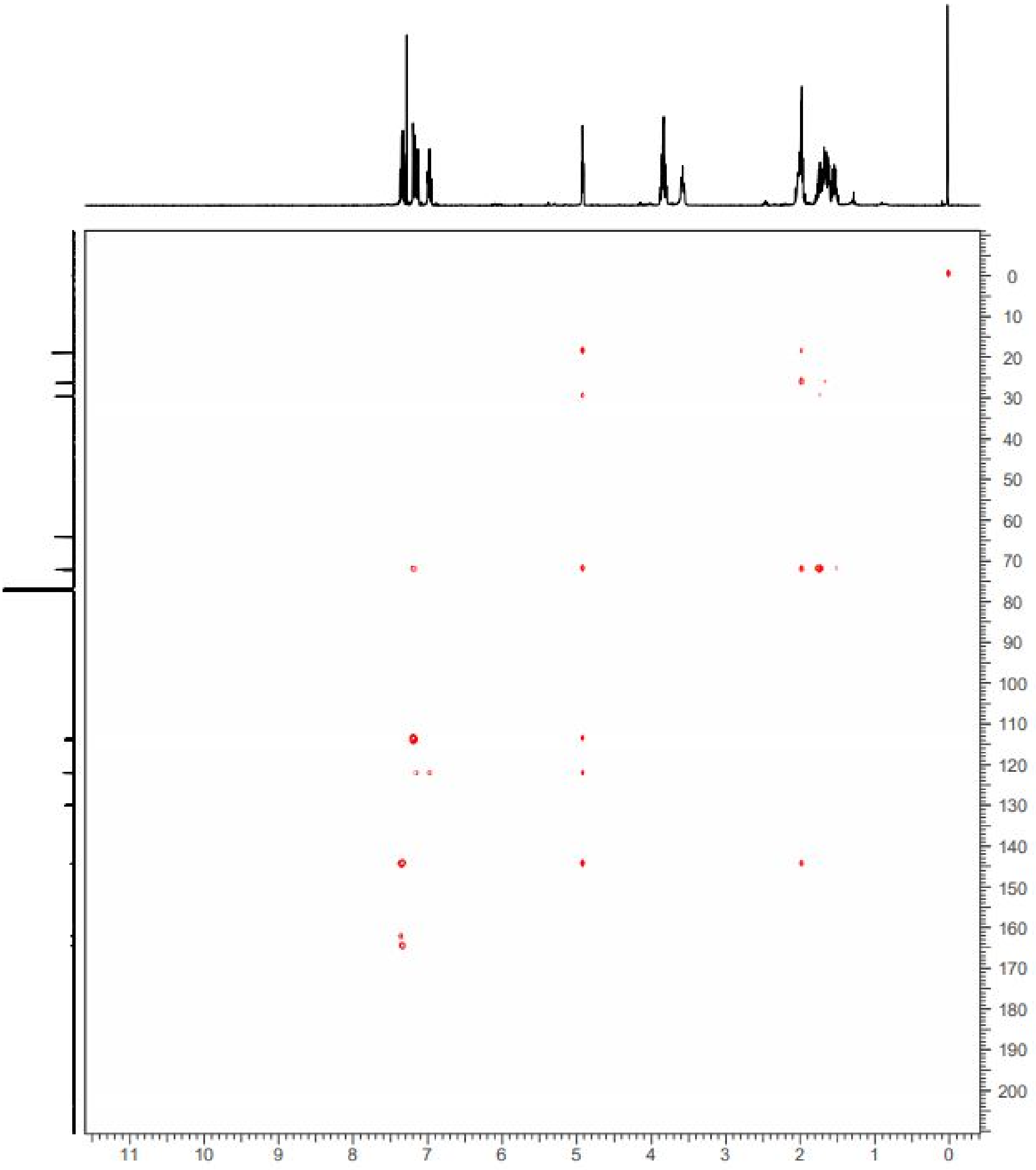

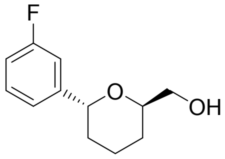

3b [^{1}H,^{1}H]-ROESY (400 MHz, $CDCl_3$):

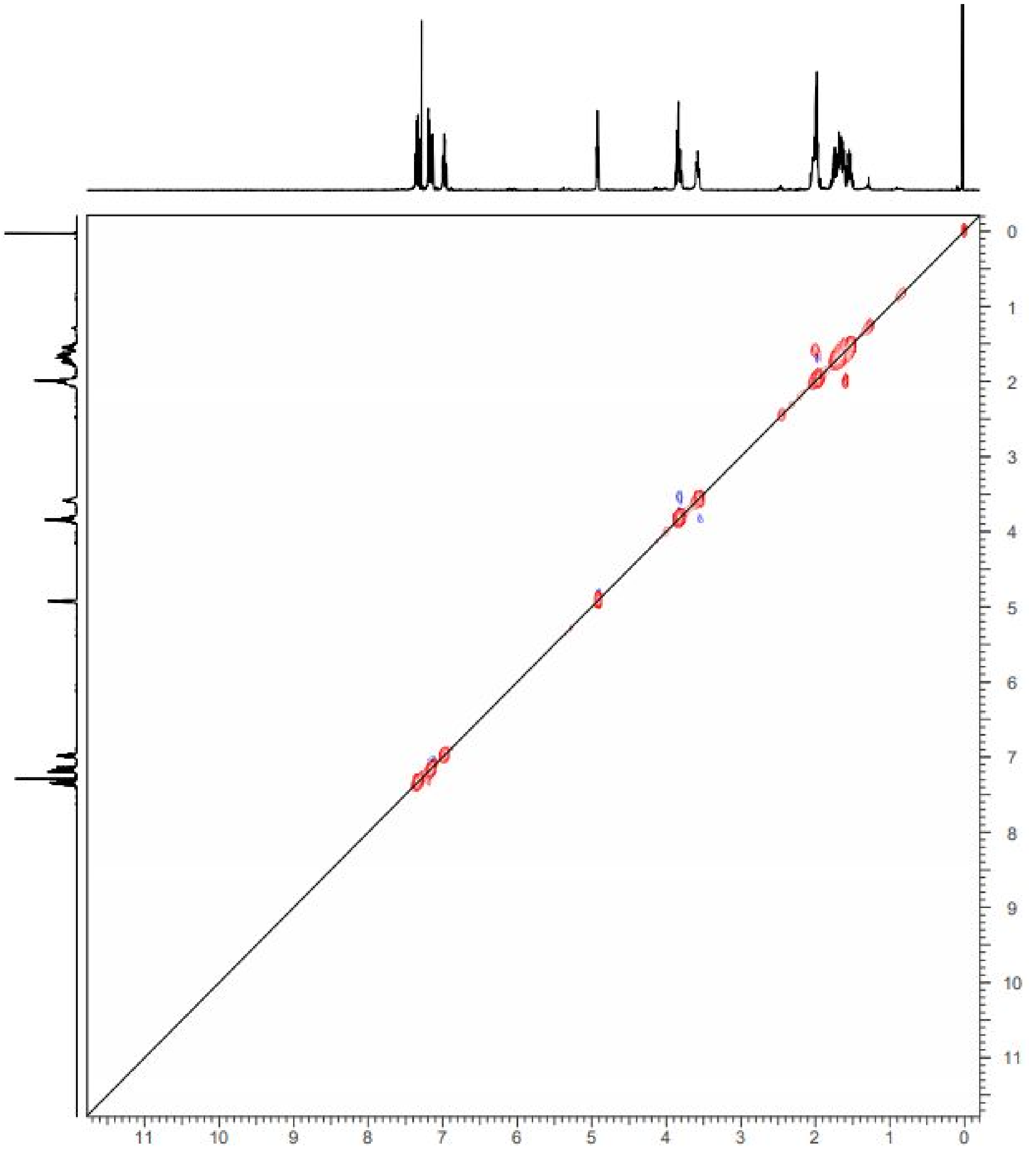

((2*R*,6*R*)-6-(4-methoxyphenyl)tetrahydro-2*H*-pyran-2-yl)methanol, **3c**

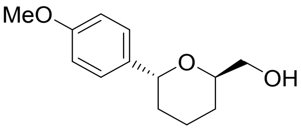

3c ^{1}H NMR (400 MHz, $CDCl_3$):

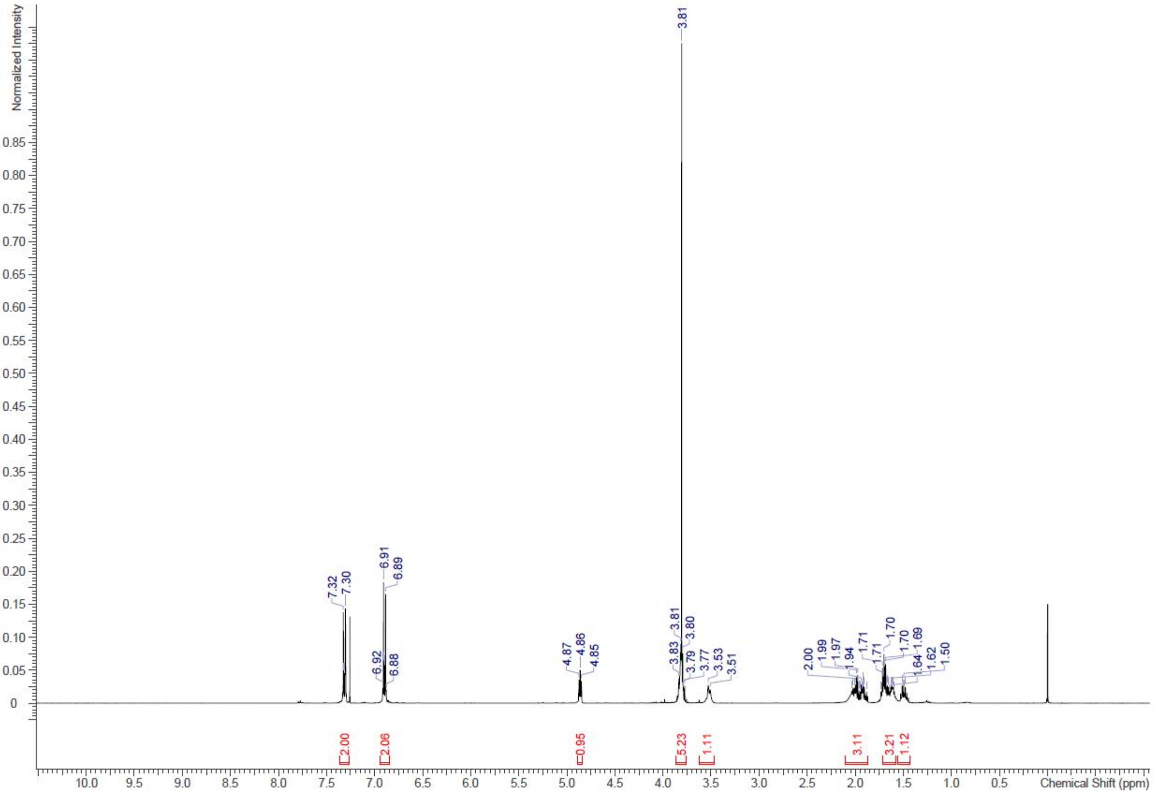

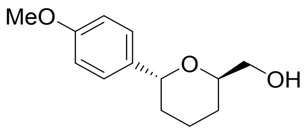

3c ^{13}C NMR (101 MHz, $CDCl_3$):

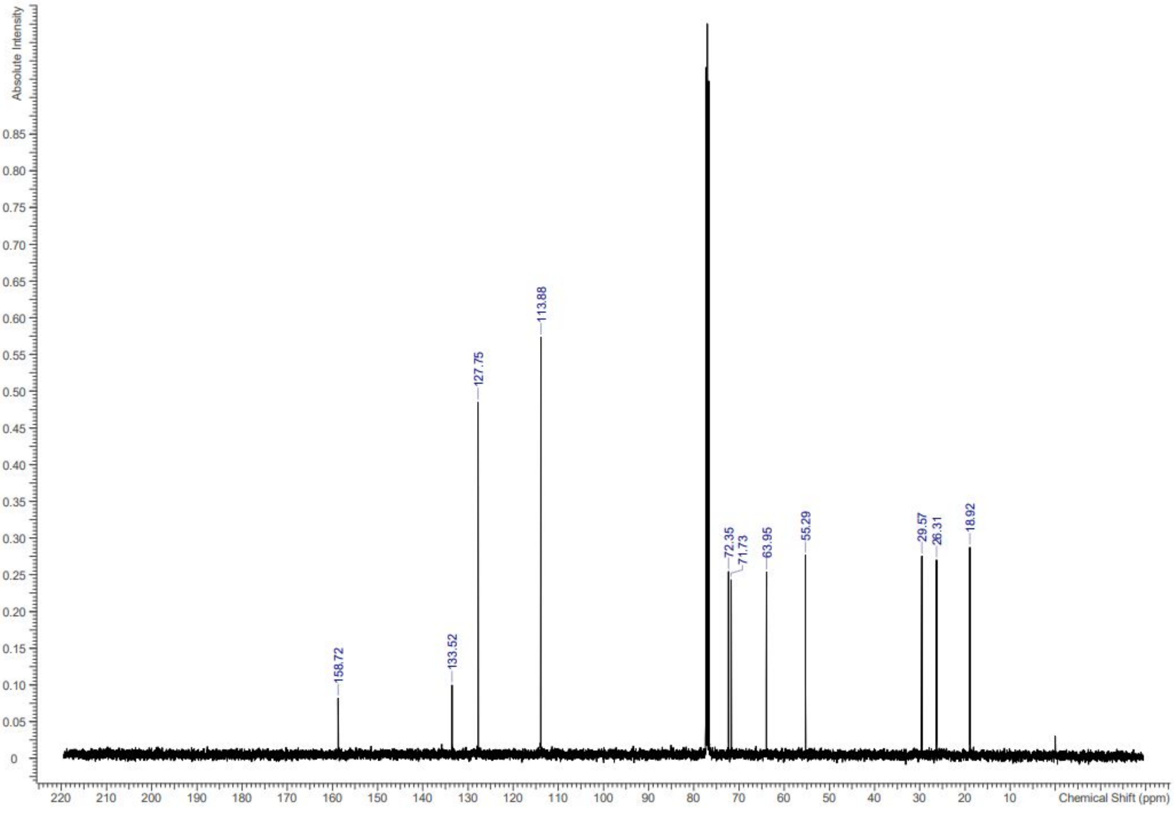

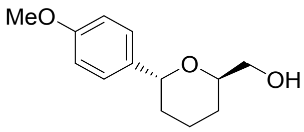

3c [^{1}H,^{1}H]-COSY (400 MHz, $CDCl_3$):

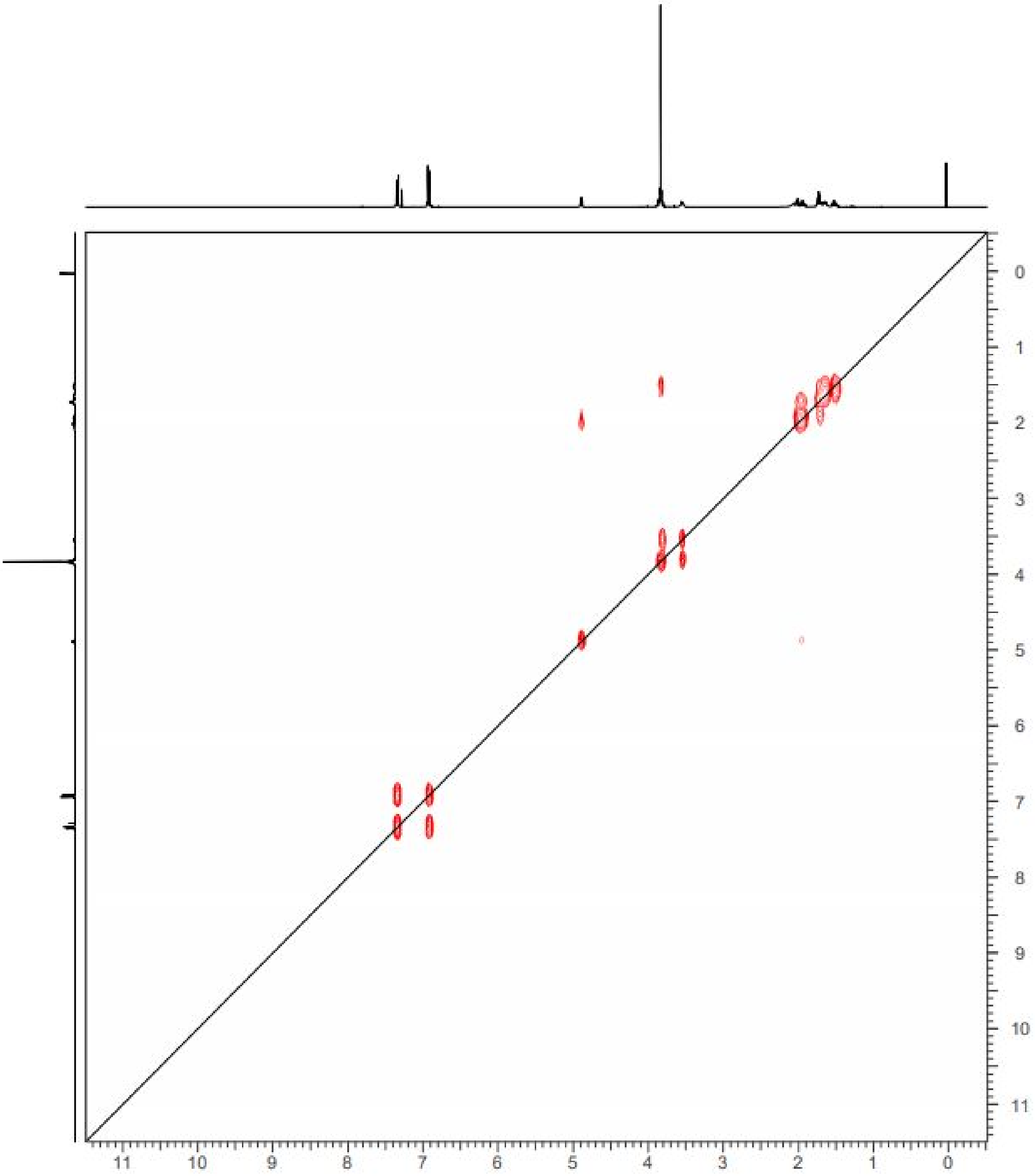

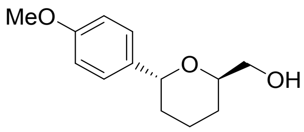

3c [^{1}H,^{13}C]-HSQC (400 MHz, 101 MHz, $CDCl_3$):

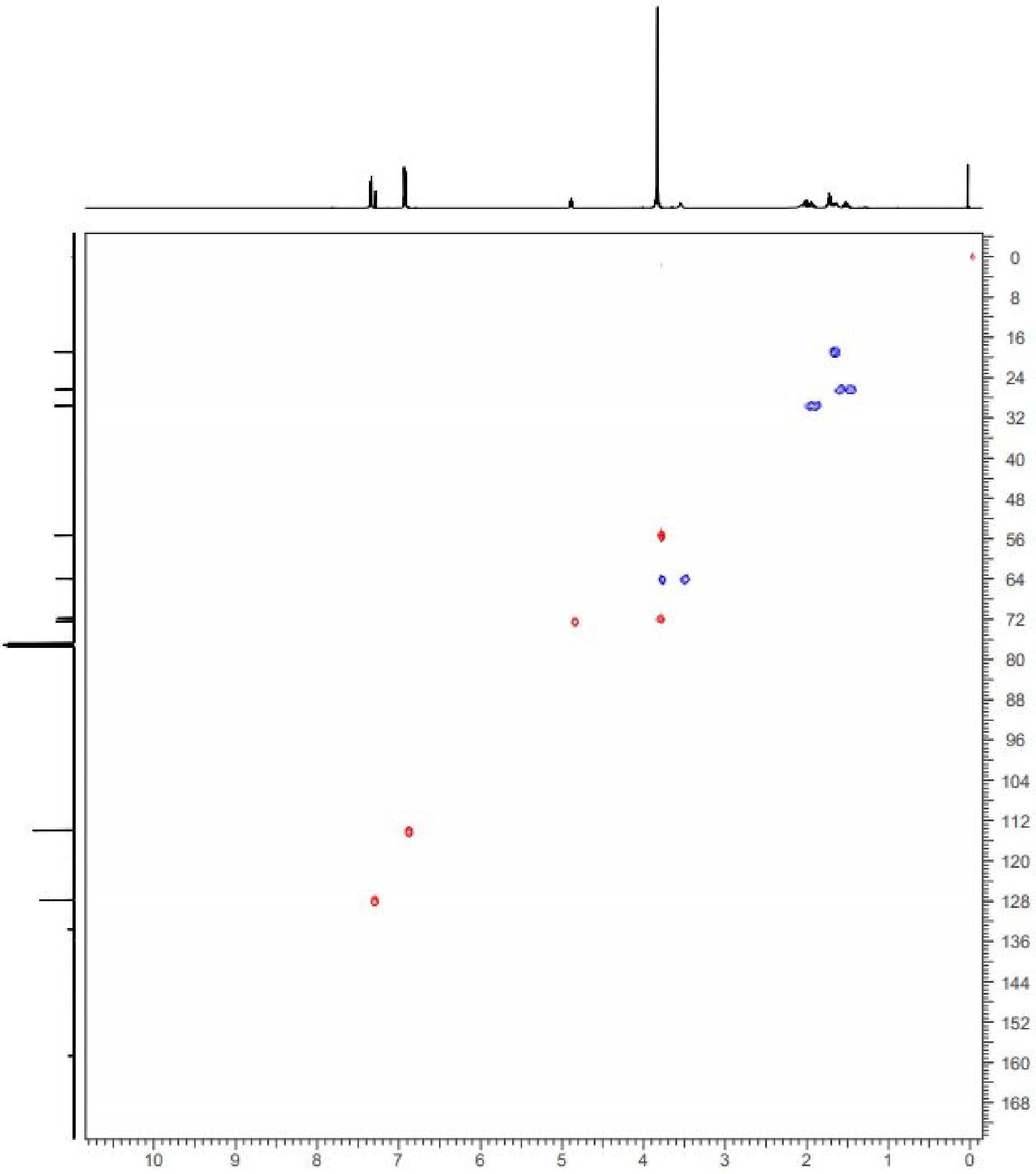

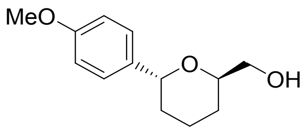

3c [^{1}H,^{13}C]-HMBC (400 MHz, 101 MHz, $CDCl_3$):

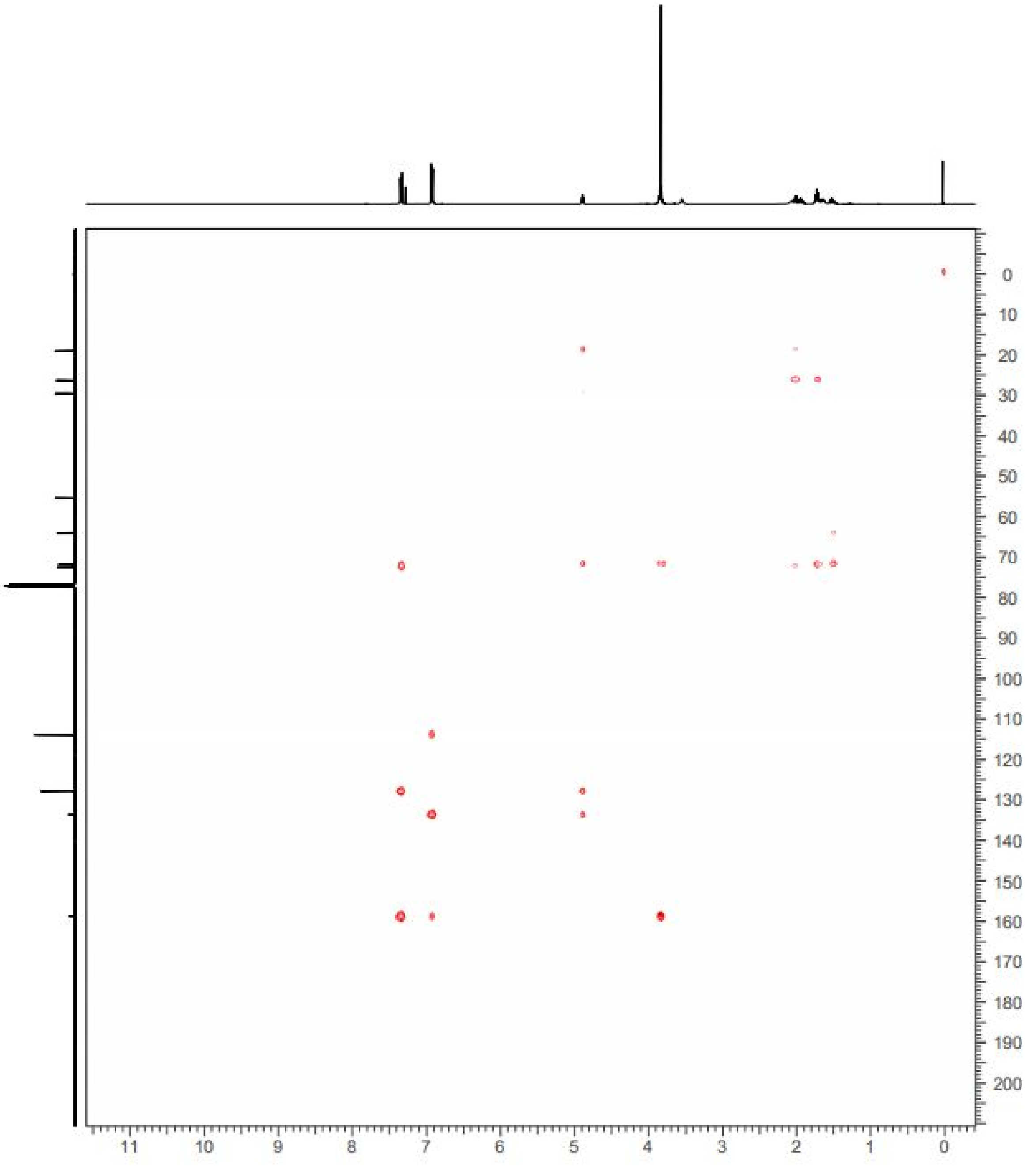

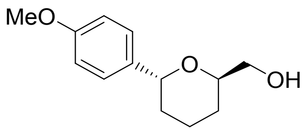

3c [^{1}H,^{1}H]-ROESY (400 MHz, $CDCl_3$):

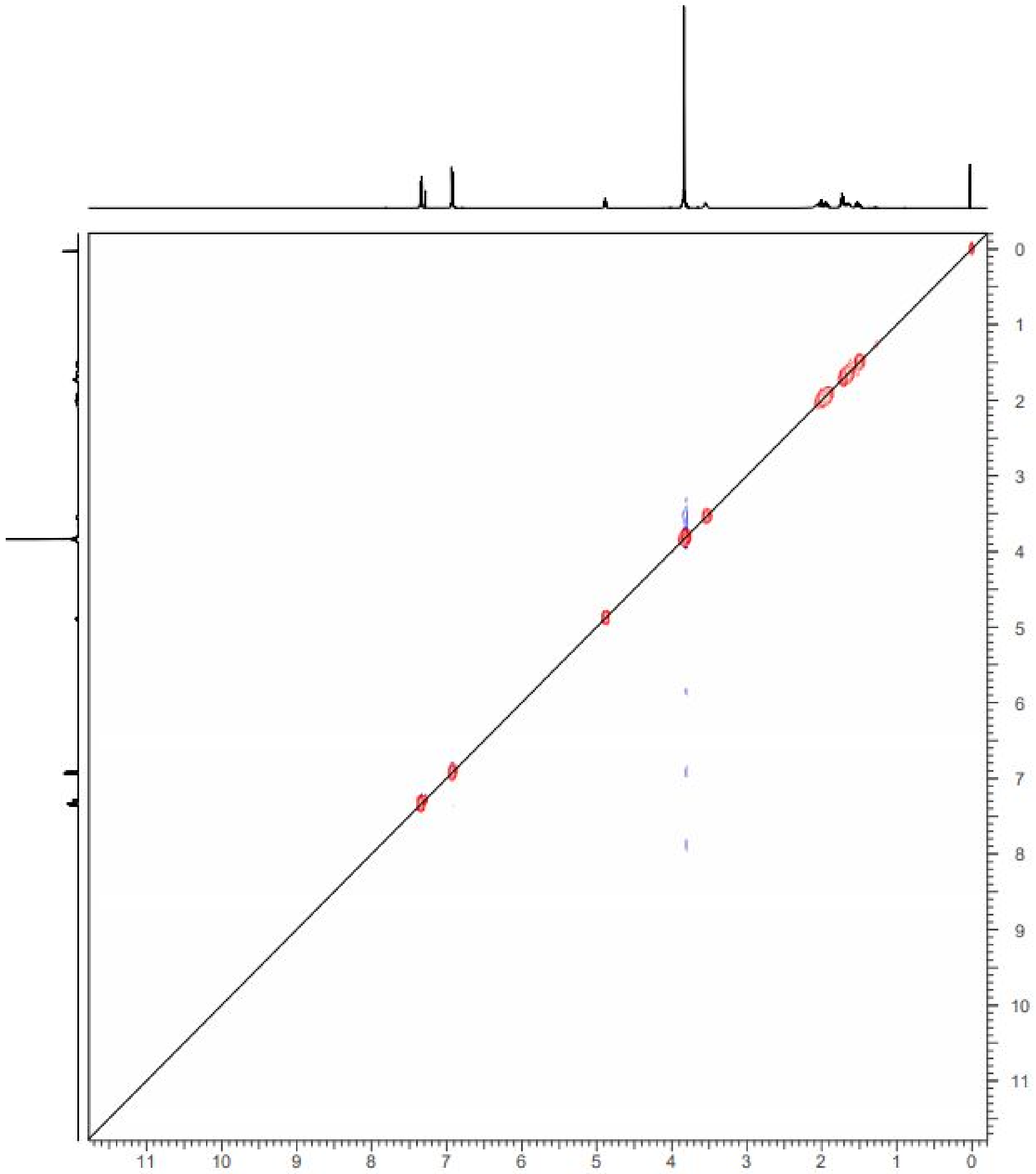

((2*R*,6*R*)-6-(3-methoxyphenyl)tetrahydro-2*H*-pyran-2-yl)methanol, **3d**

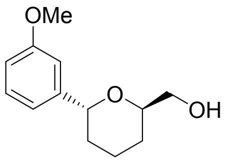

3d ^{1}H NMR (400 MHz, $CDCl_3$):

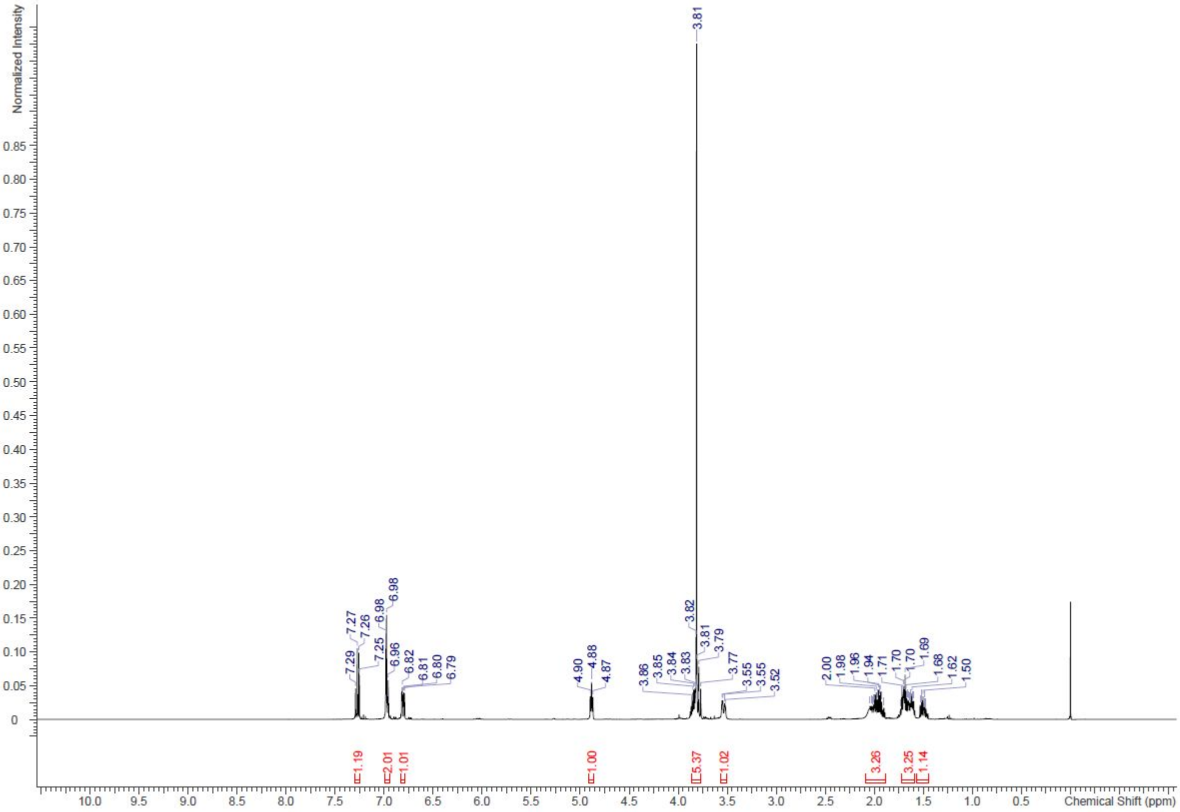

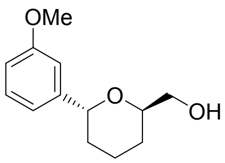

3d ^{13}C NMR (101 MHz, $CDCl_3$):

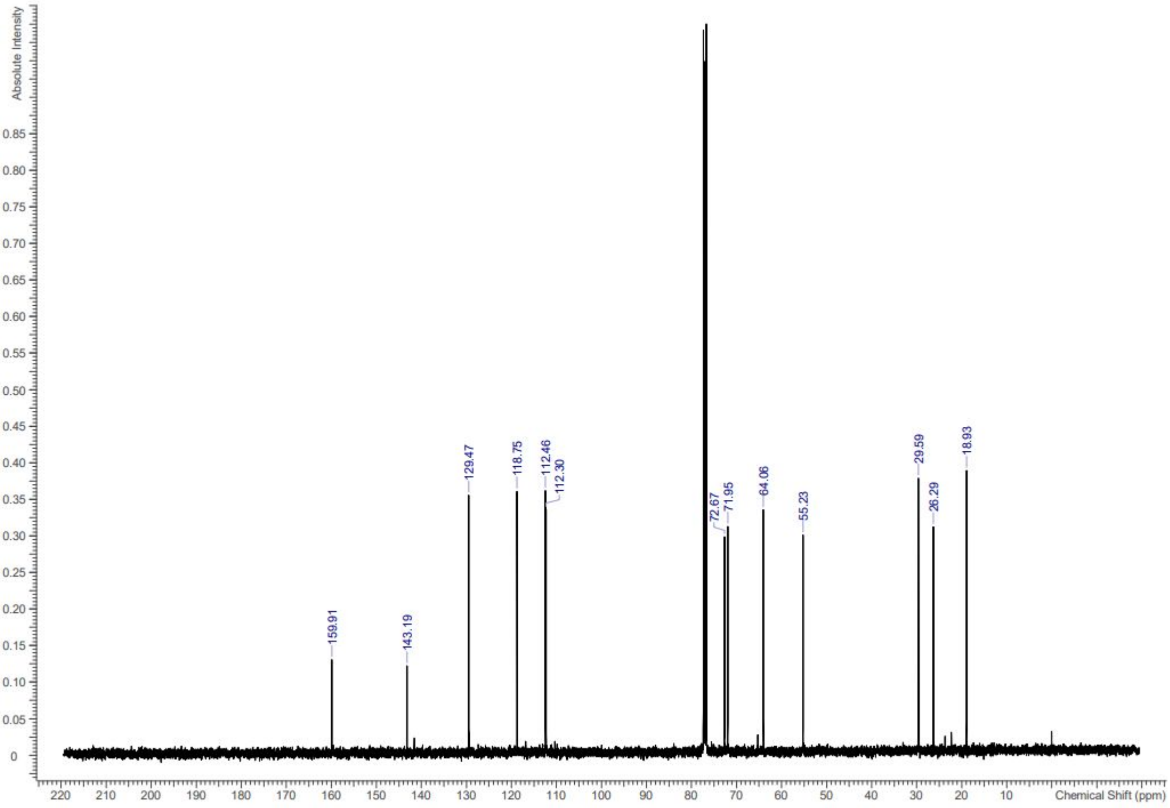

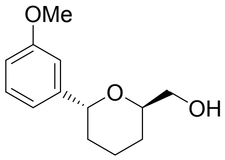

3d [1H,1H]-COSY (400 MHz, $CDCl_3$):

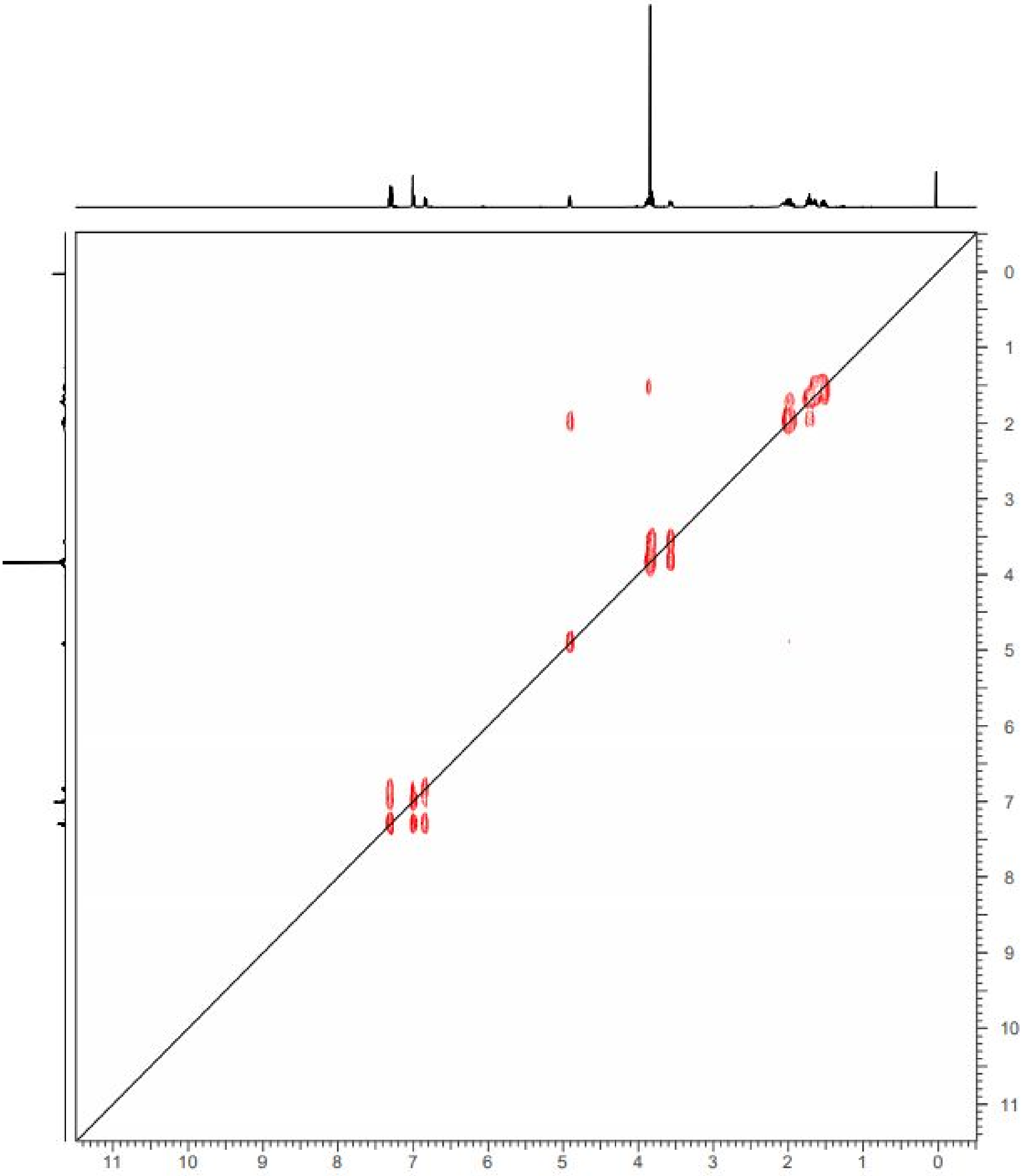

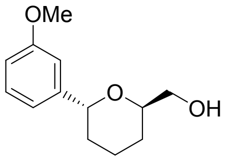

3d [^{1}H,^{13}C]-HSQC (400 MHz, 101 MHz, $CDCl_3$):

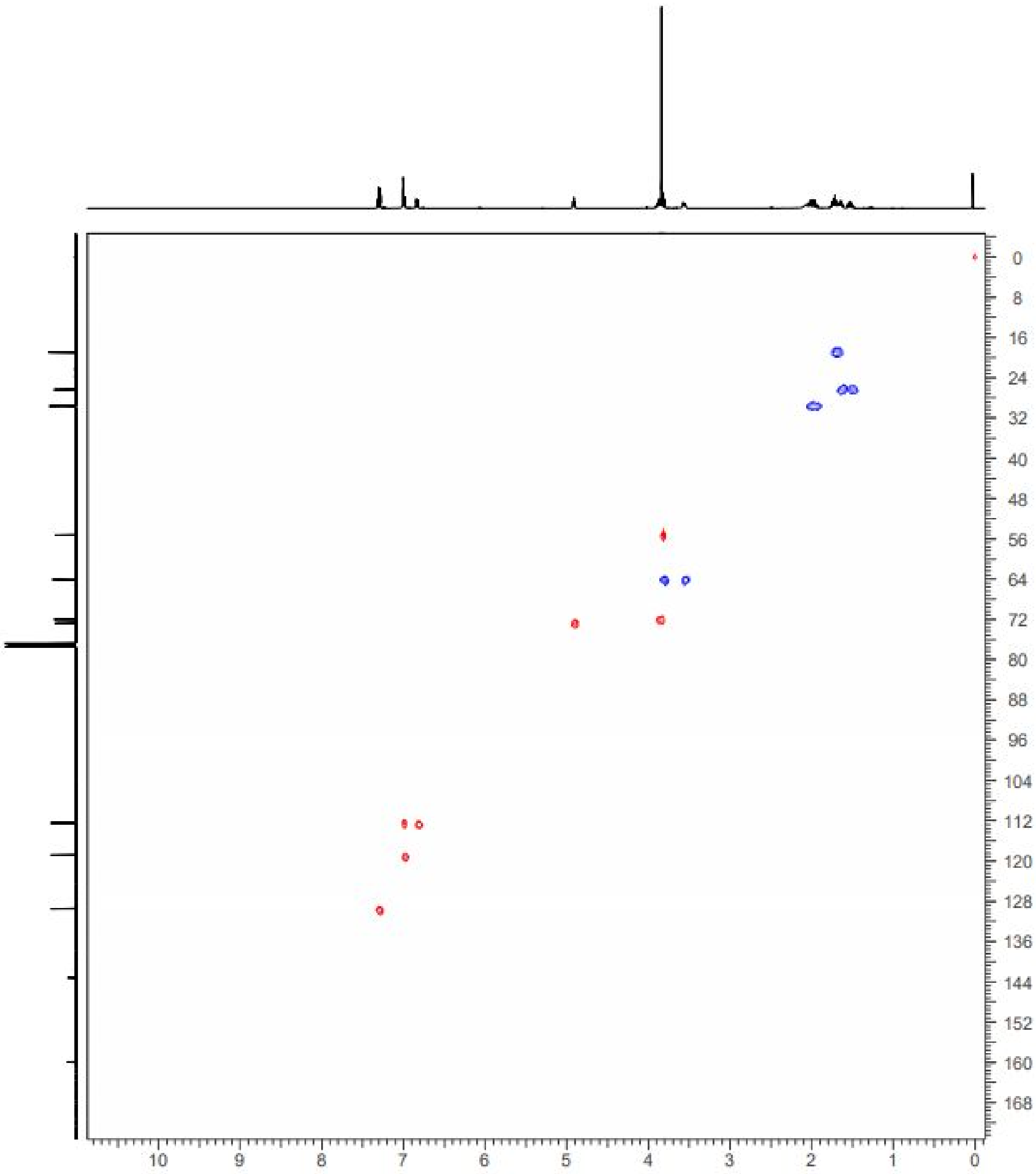

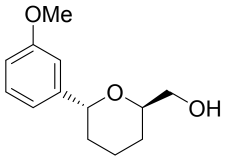

3d [^{1}H,^{13}C]-HMBC (400 MHz, 101 MHz, $CDCl_3$):

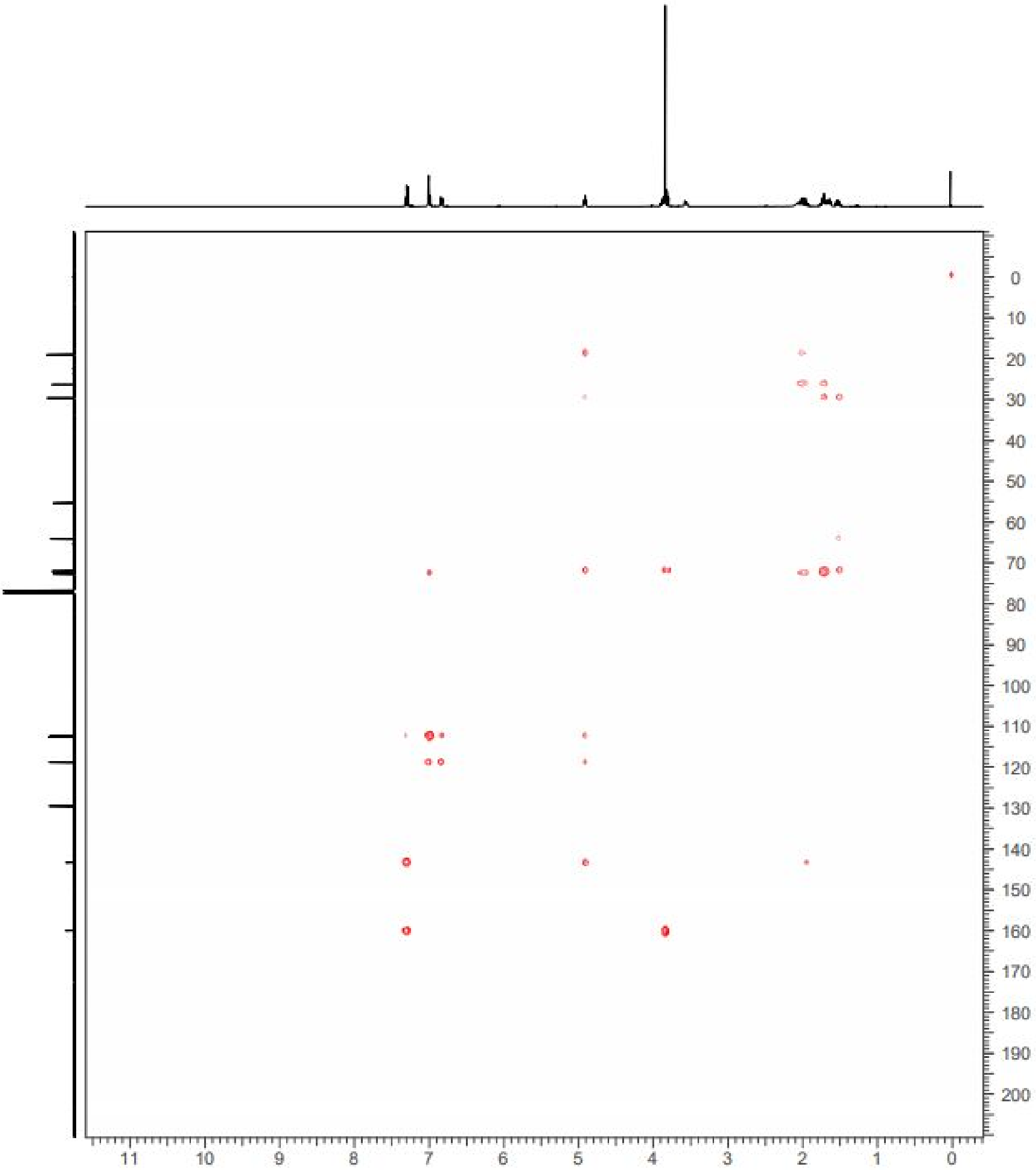

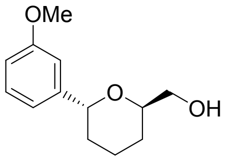

3d [^{1}H,^{1}H]-ROESY (400 MHz, $CDCl_3$):

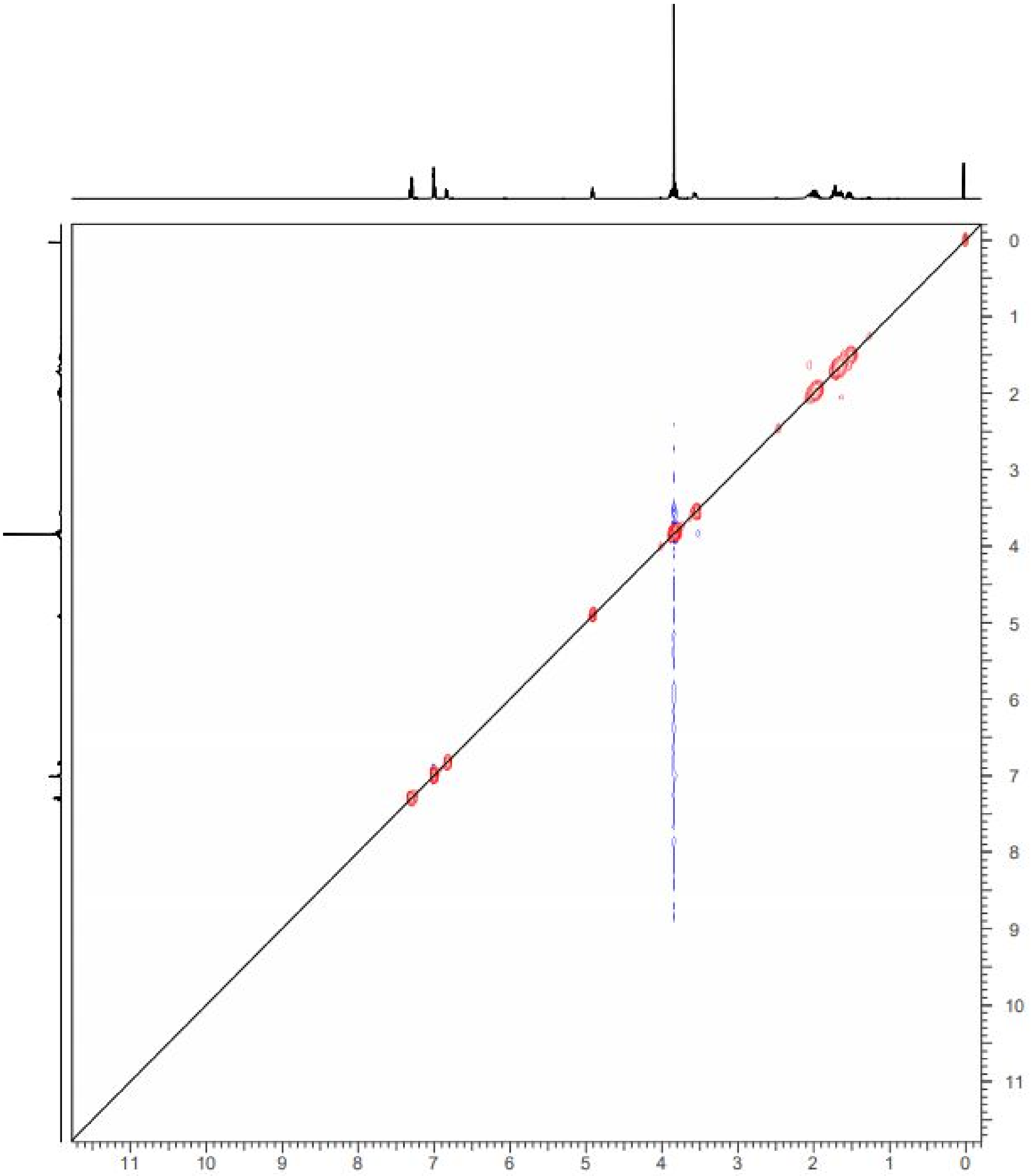

((2*R*,6*R*)-6-(2-methoxyphenyl)tetrahydro-2*H*-pyran-2-yl)methanol, **3e**

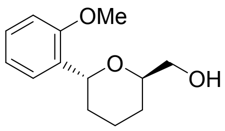

3e ^{1}H NMR (400 MHz, $CDCl_3$):

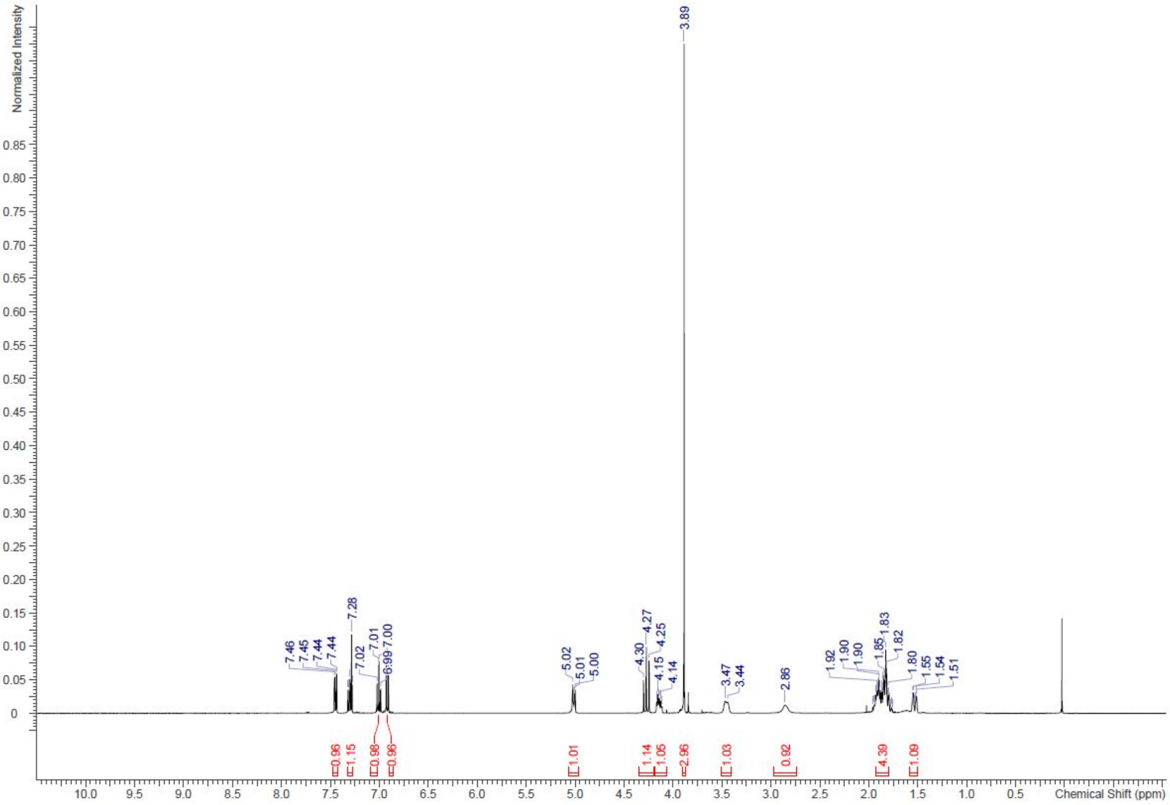

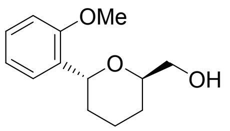

3e ^{13}C NMR (101 MHz, $CDCl_3$):

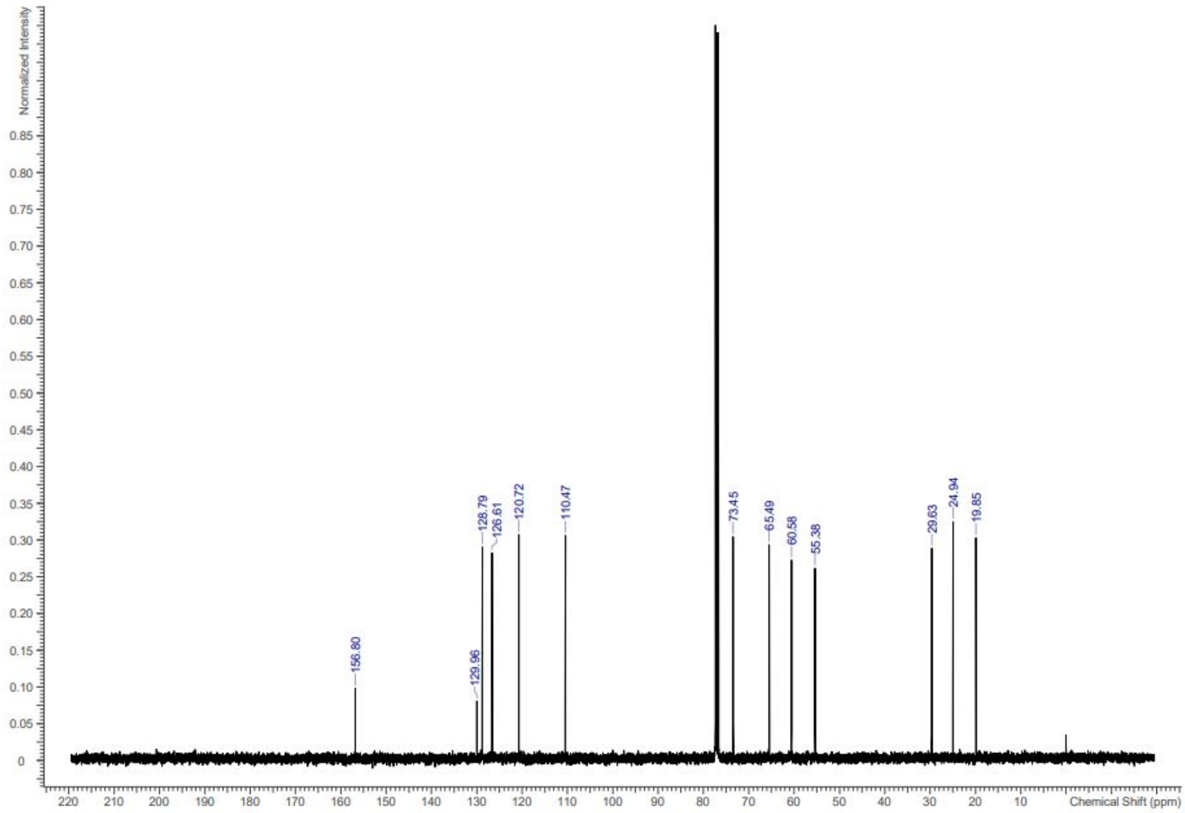

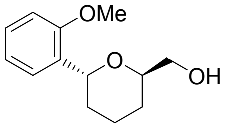

3e [^{1}H,^{1}H]-COSY (400 MHz, $CDCl_3$):

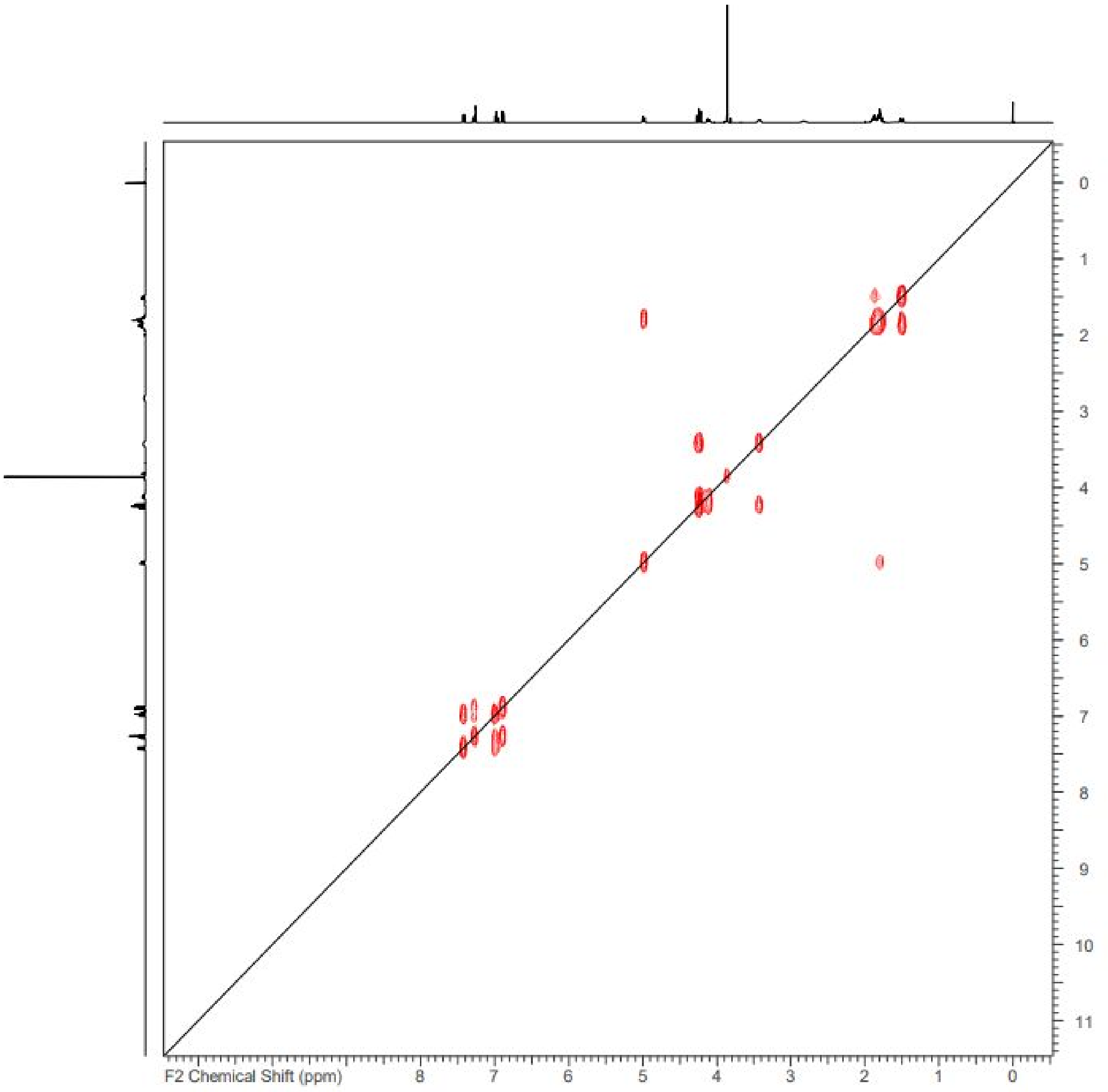

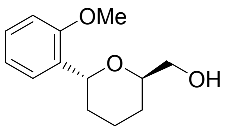

3e [^{1}H,^{13}C]-HSQC (400 MHz, 101 MHz, $CDCl_3$):

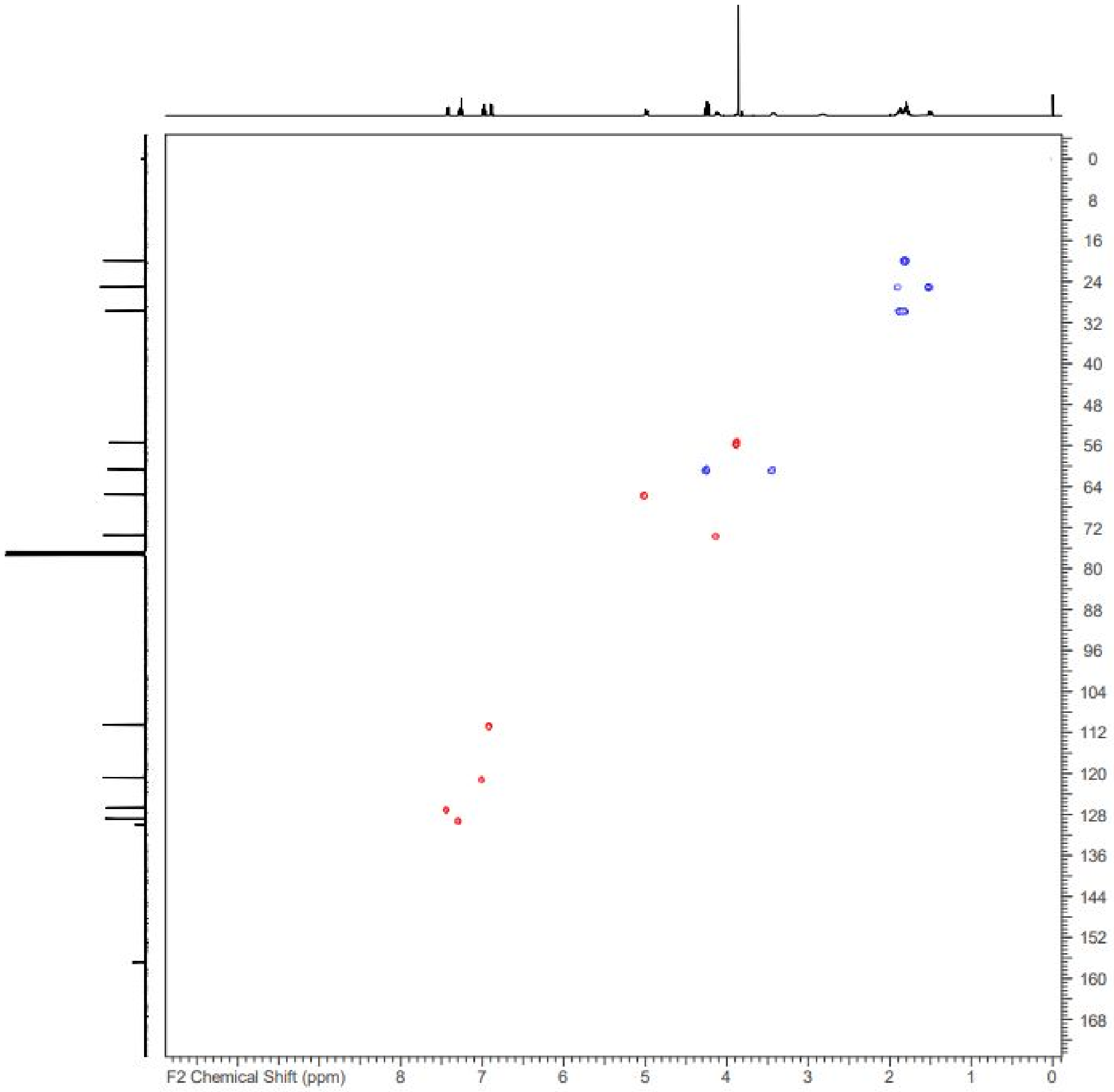

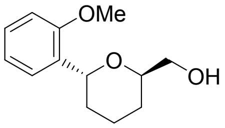

3e [^{1}H,^{13}C]-HMBC (400 MHz, 101 MHz, $CDCl_3$):

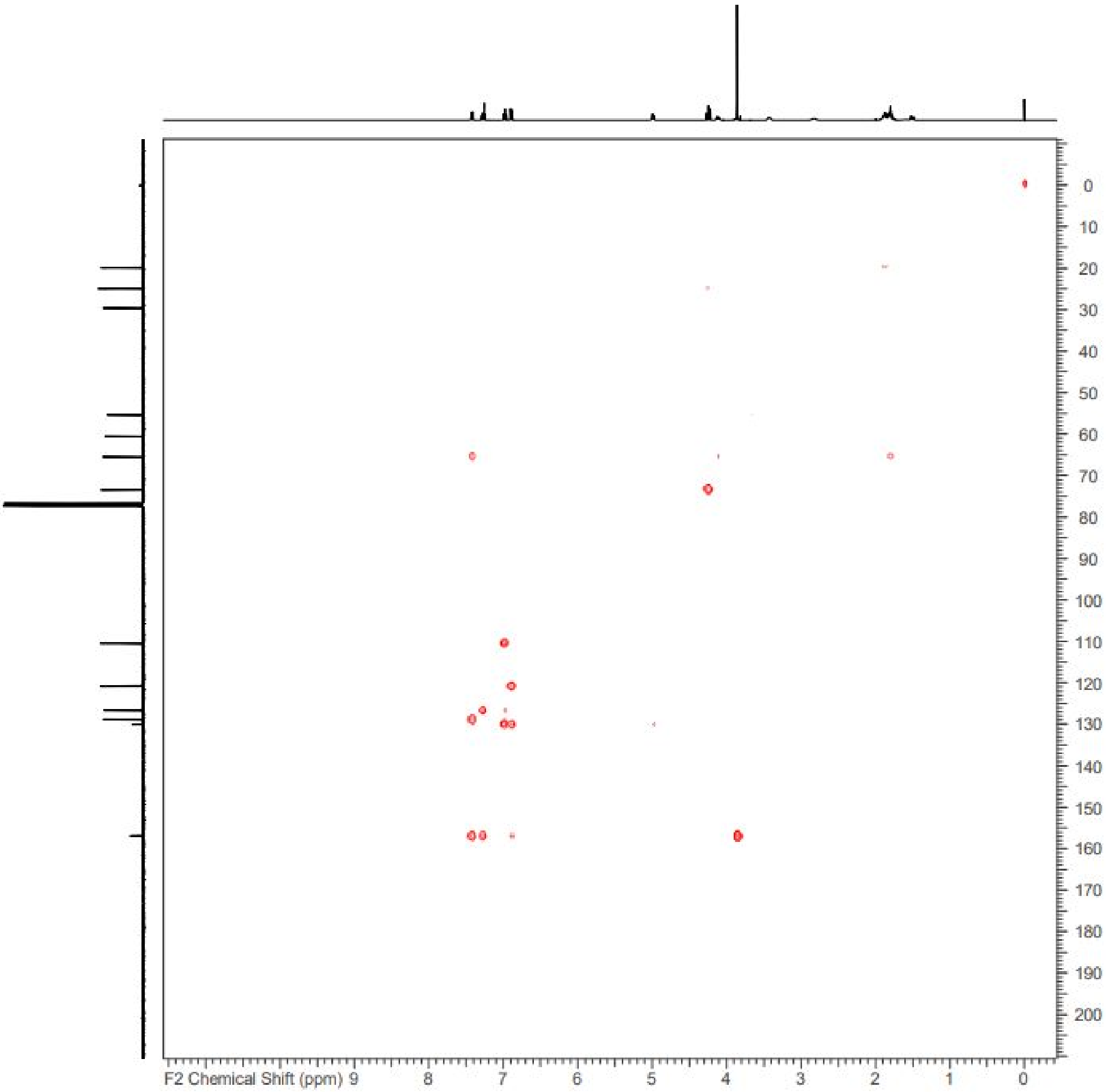

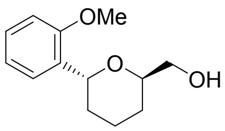

3e[1H,1H]-ROESY (400 MHz, $CDCl_3$):

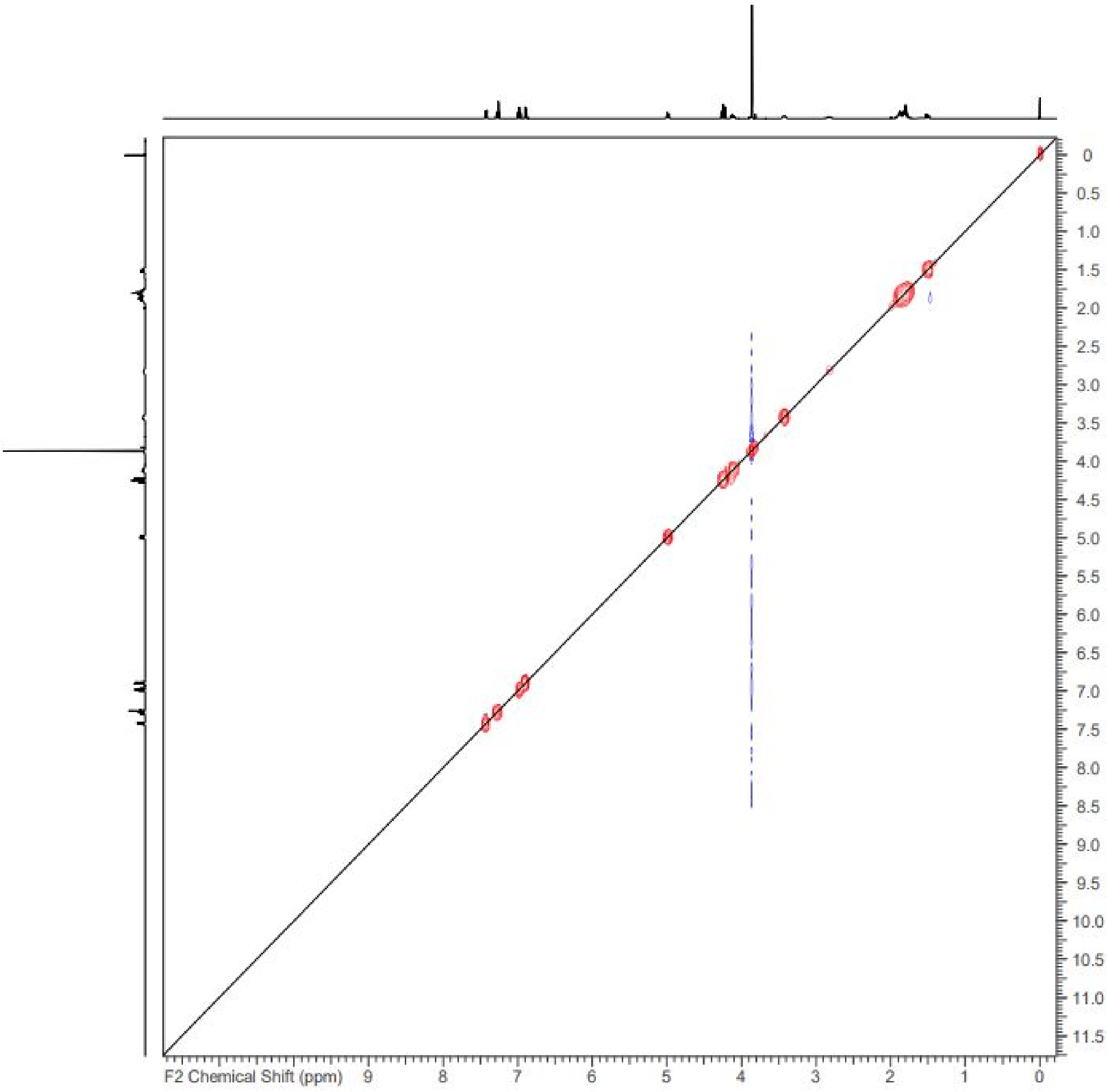

4-((2*R*,6*R*)-6-(hydroxymethyl)tetrahydro-2*H*-pyran-2-yl)phenol, **3f**

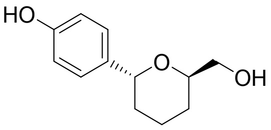

3f ^{1}H NMR (400 MHz, $CDCl_3$):

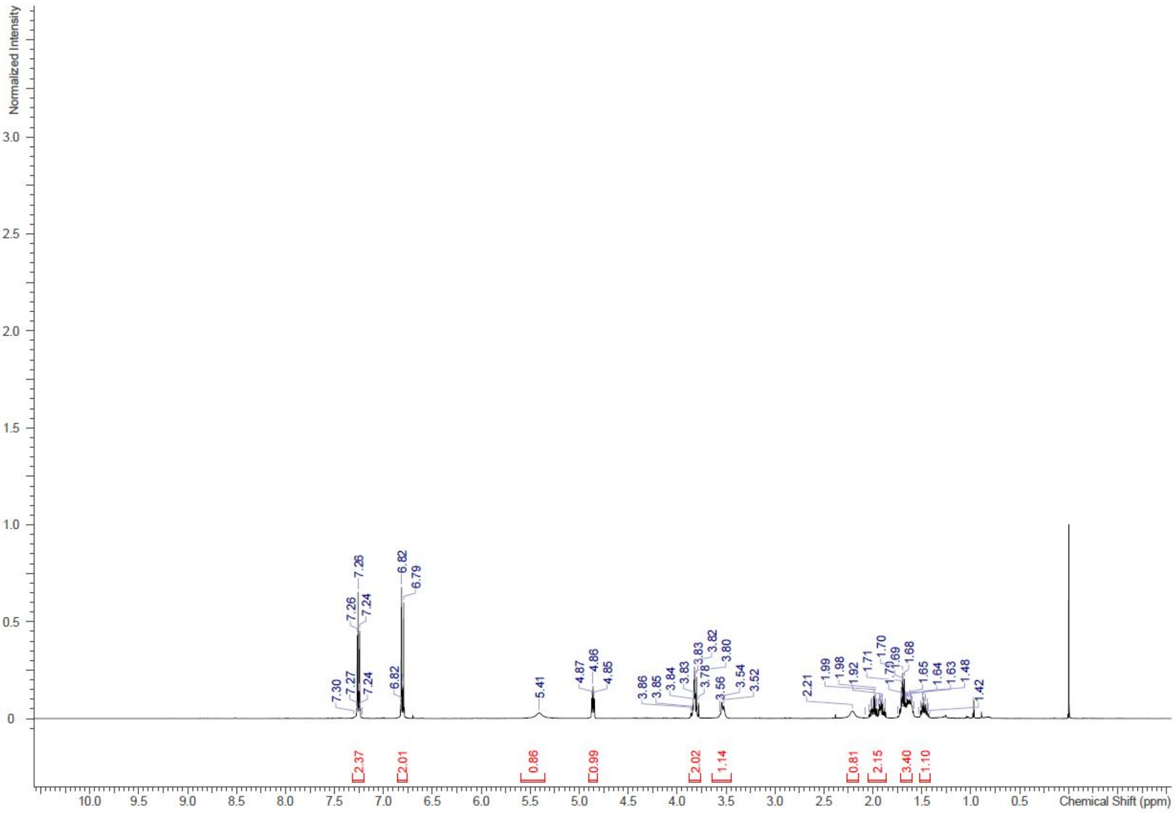

3f ^{1}H NMR (400 MHz, $CDCl_{3,}$) D_2O shake:

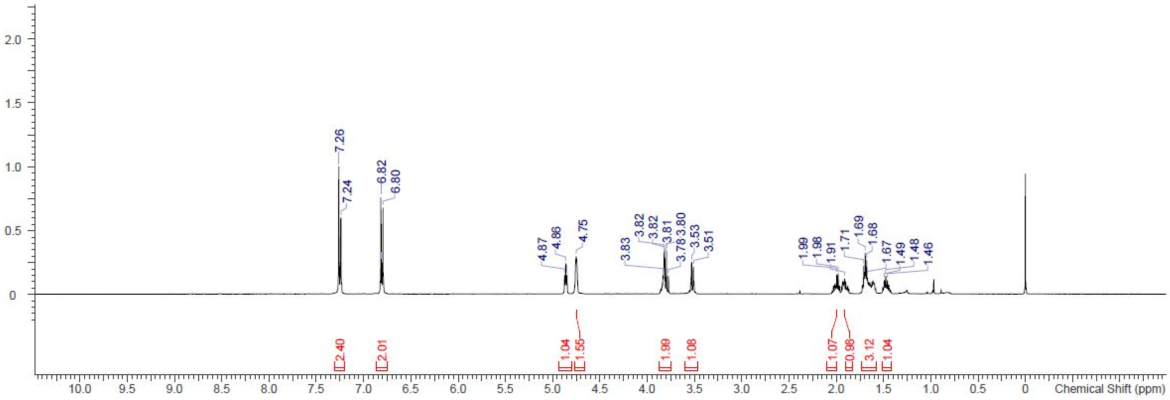

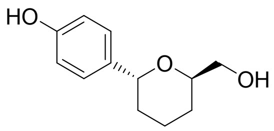

3f ^{13}C NMR (101 MHz, $CDCl_3$):

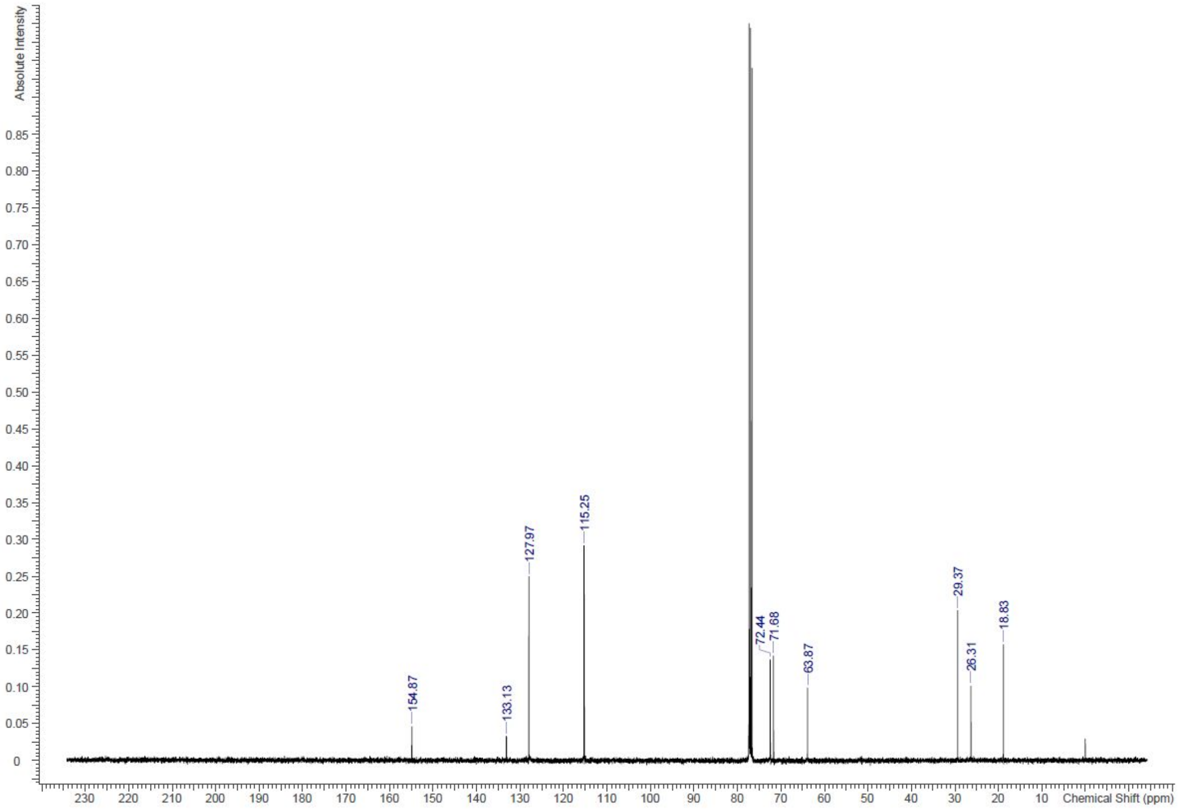

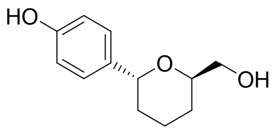

3f [^{1}H,^{1}H]-COSY (400 MHz, $CDCl_3$):

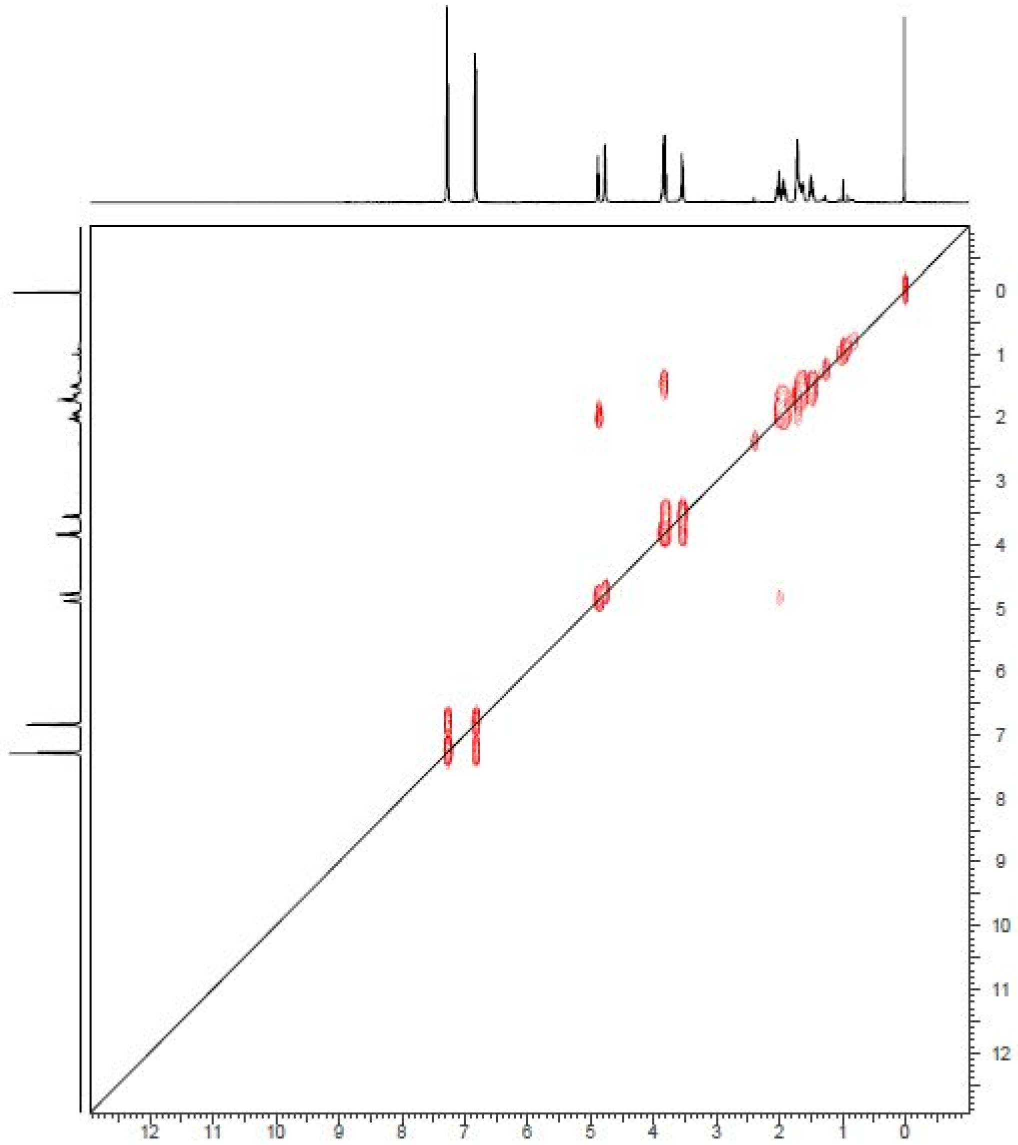

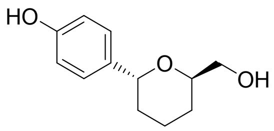

3f [^{1}H,^{13}C]-HSQC (400 MHz, 101 MHz, $CDCl_3$):

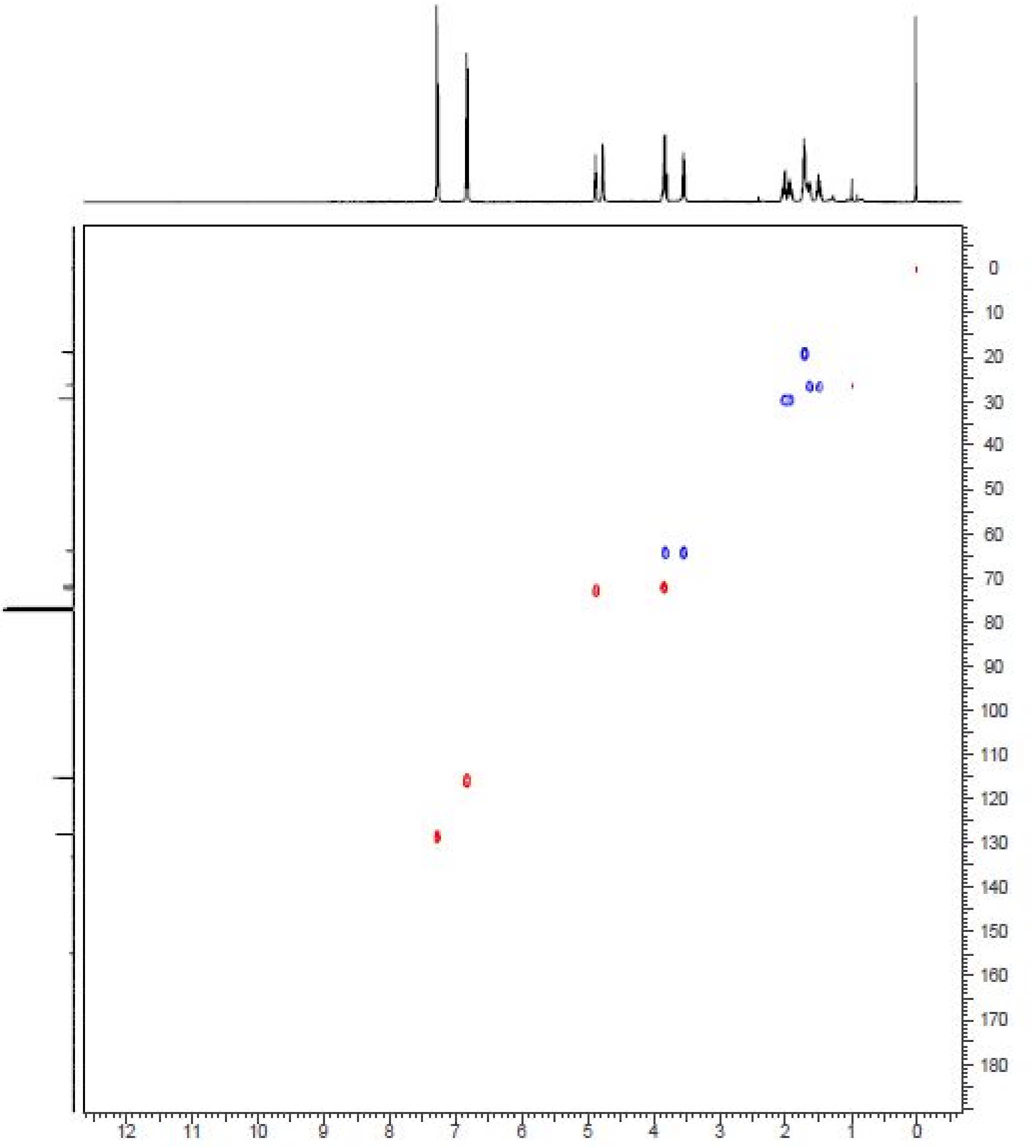

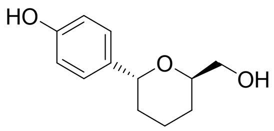

3f[1H,13C]-HMBC (400 MHz, 101 MHz, $CDCl_3$):

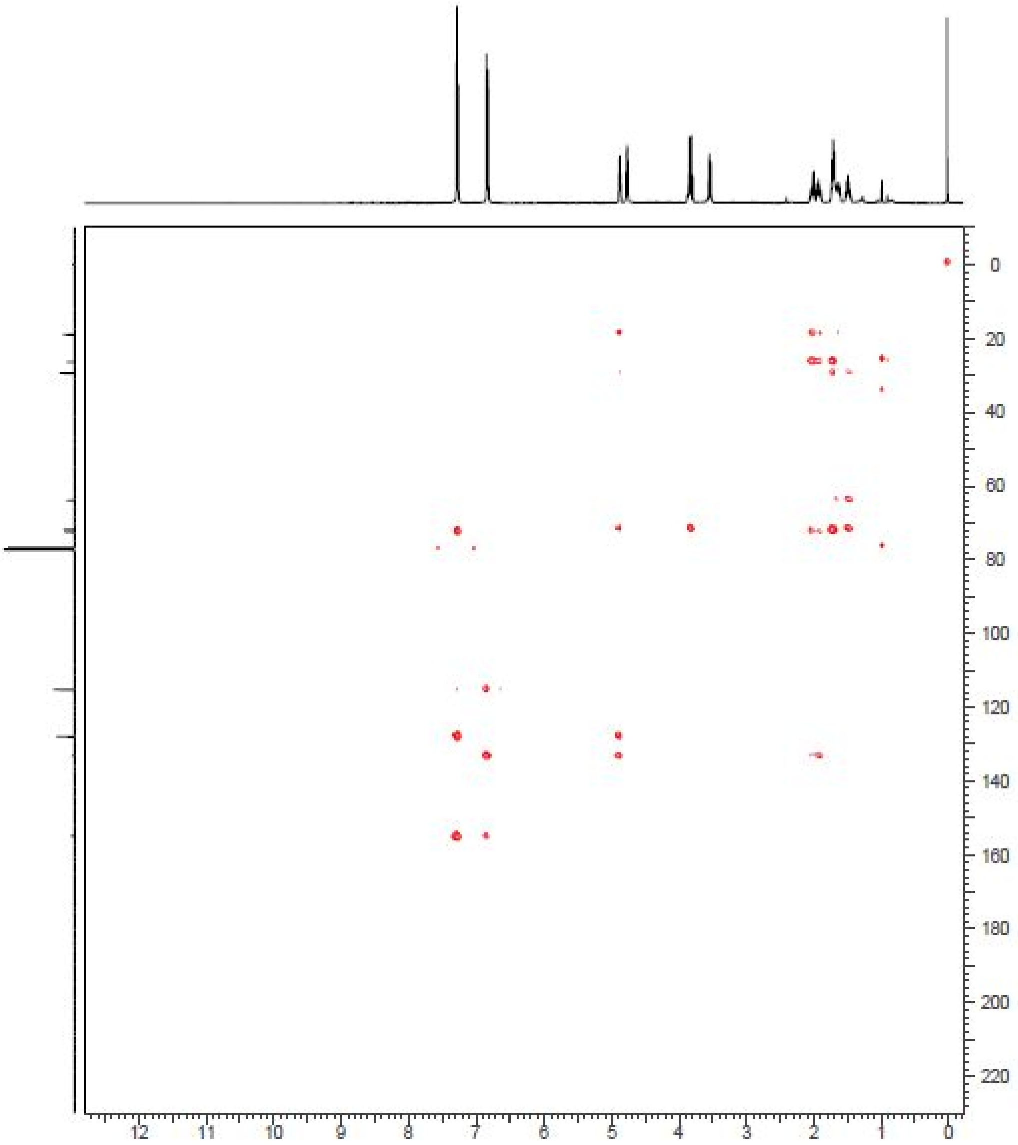

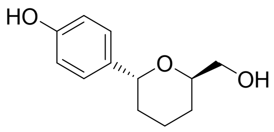

3f [^{1}H,^{1}H]-ROESY (400 MHz, $CDCl_3$):

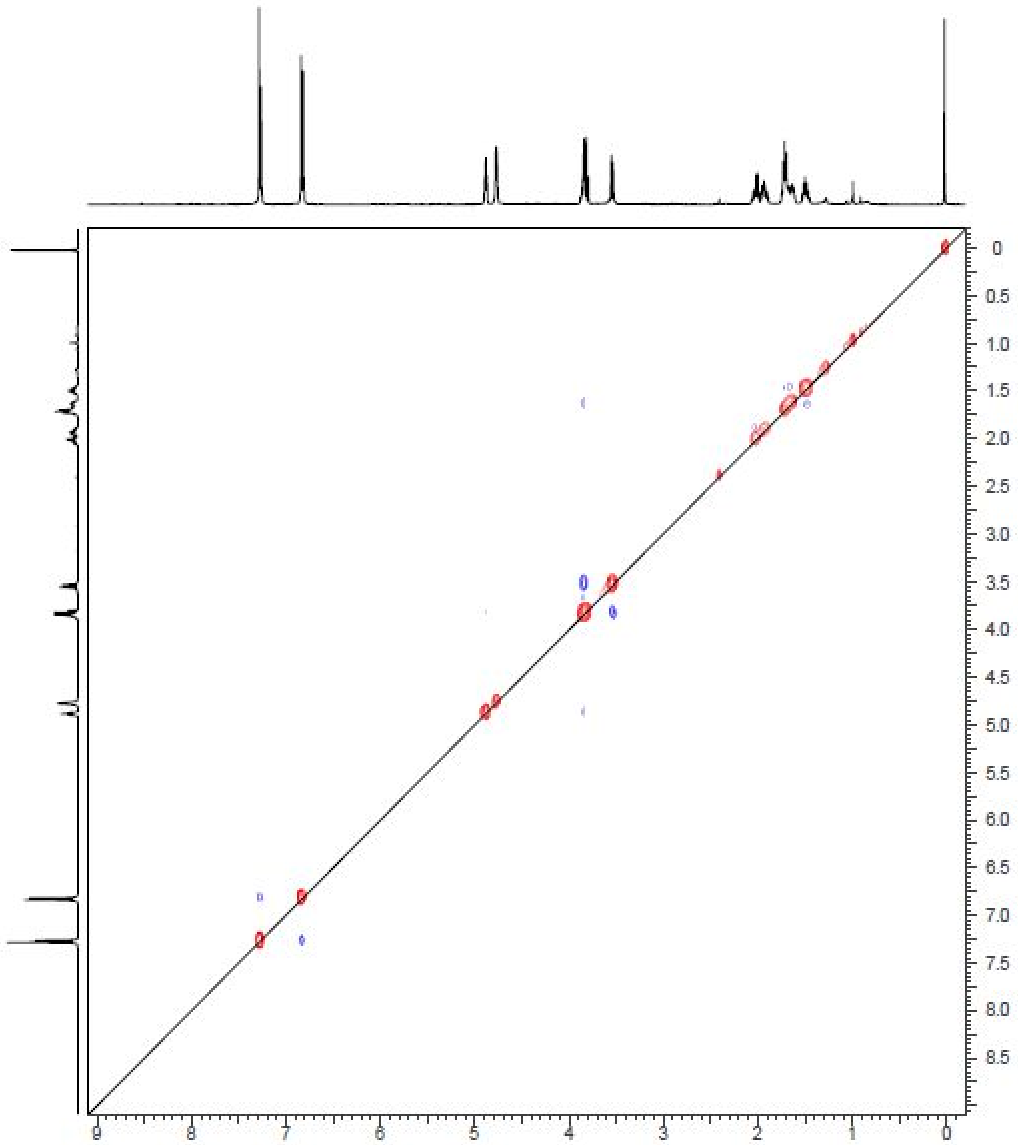

((2*R*,6*R*)-6-(*p*-benzyloxy)tetrahydro-2*H*-pyran-2-yl)methanol, **3g**

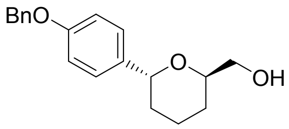

3g ^{1}H NMR (400 MHz, $CDCl_3$):

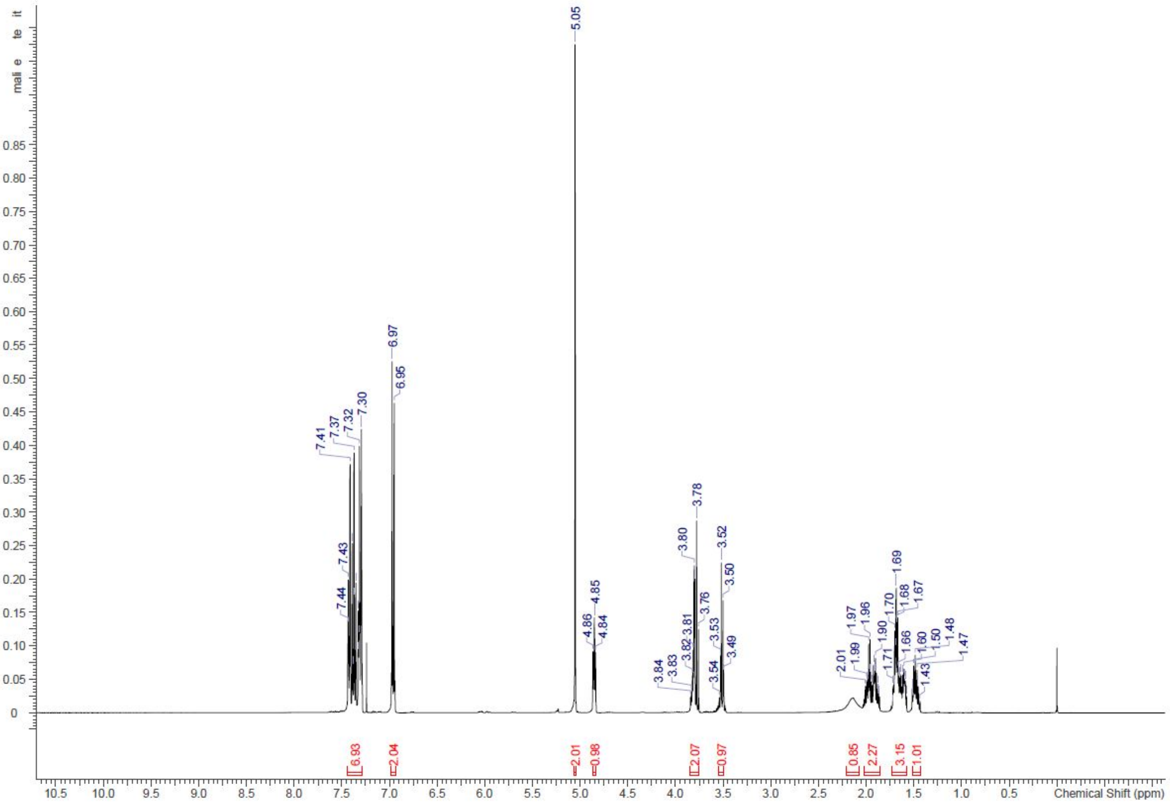

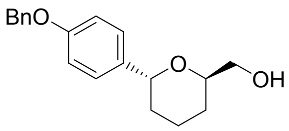

3g ^{13}C NMR (101 MHz, $CDCl_3$):

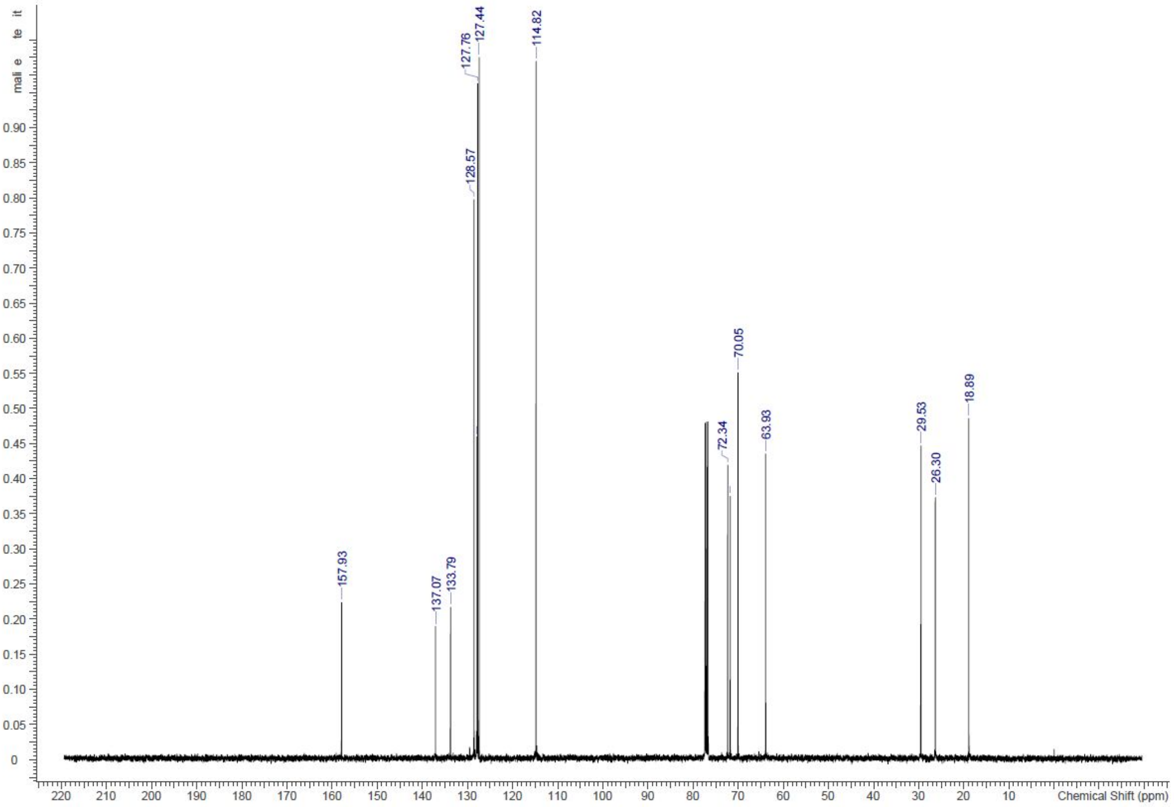

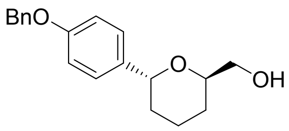

3g [^{1}H,^{1}H]-COSY (400 MHz, $CDCl_3$):

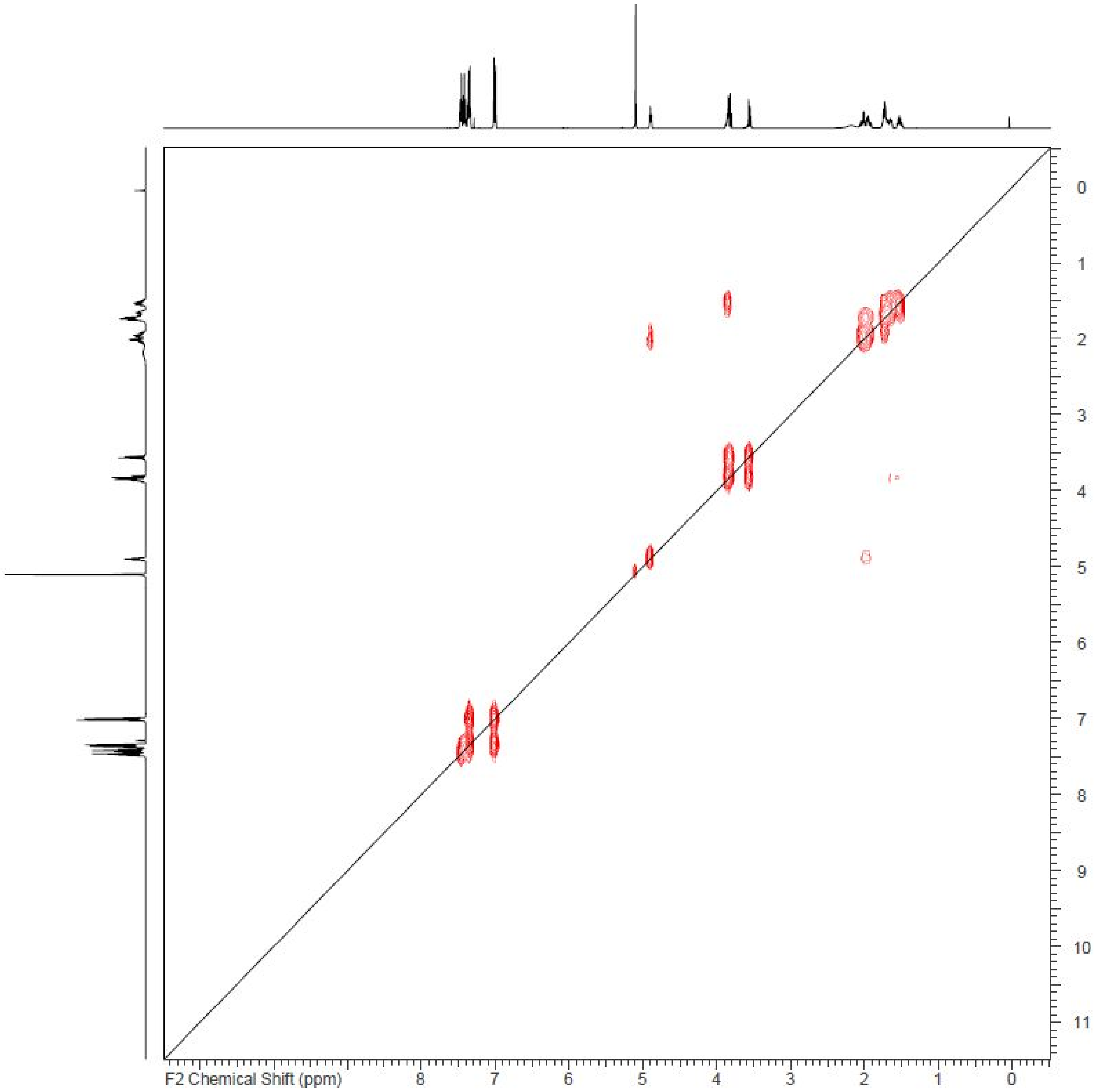

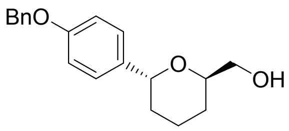

3g [^{1}H,^{13}C]-HSQC (400 MHz, 101 MHz, $CDCl_3$):

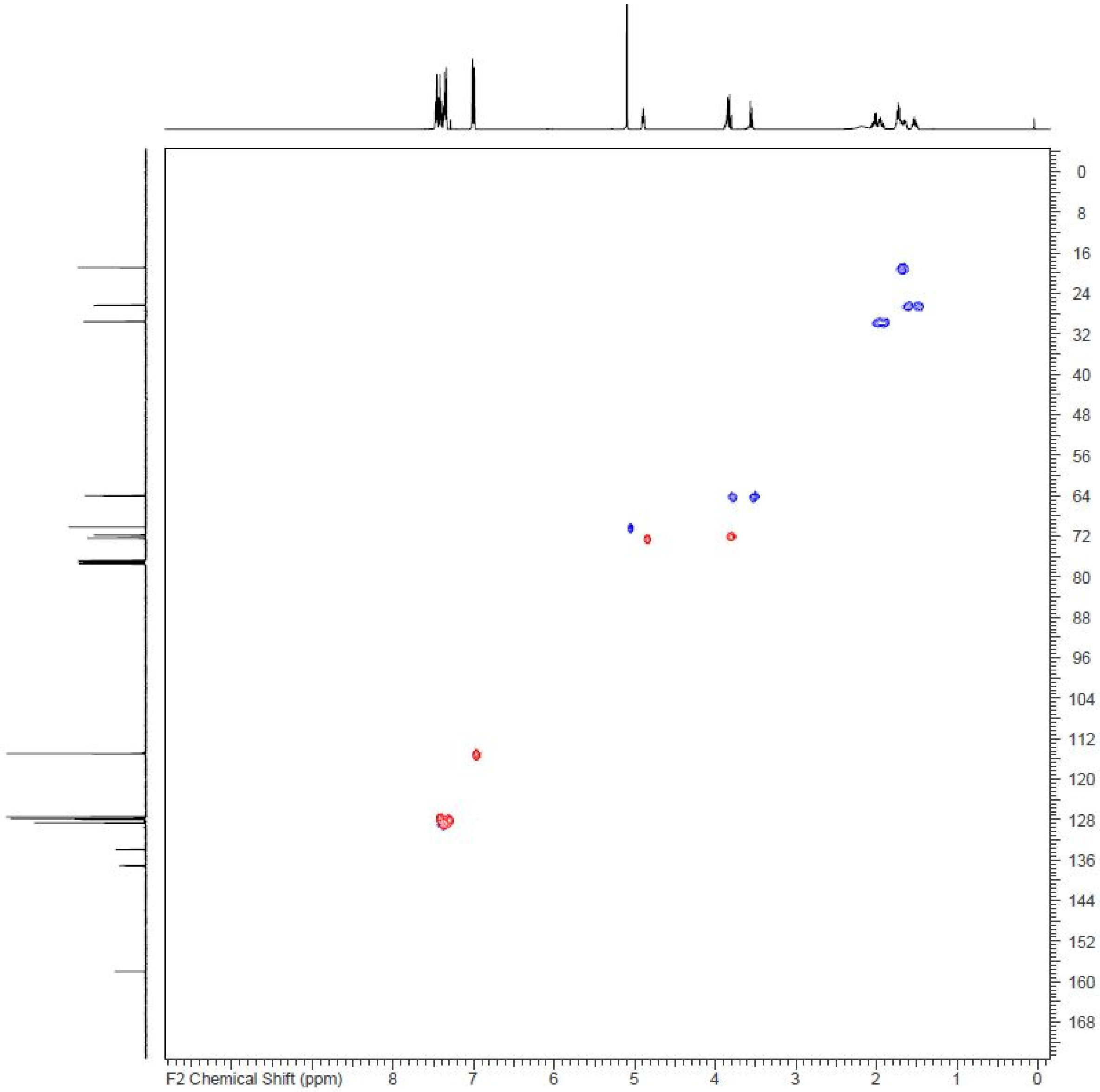

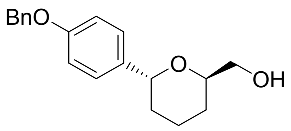

3g [^{1}H,^{13}C]-HMBC (400 MHz, 101 MHz, $CDCl_3$):

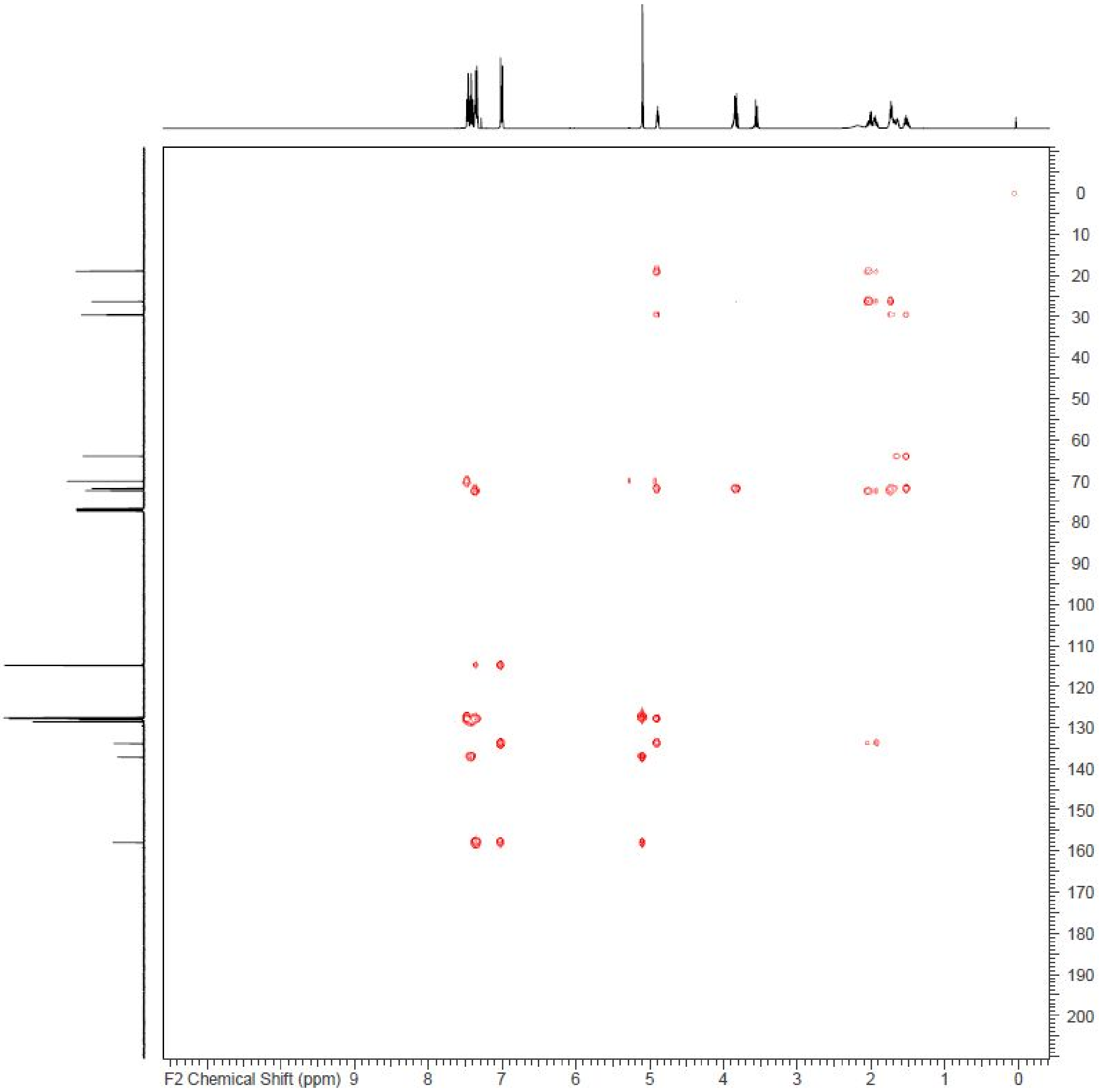

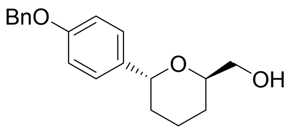

3g [^{1}H,^{1}H]-ROESY (400 MHz, $CDCl_3$):

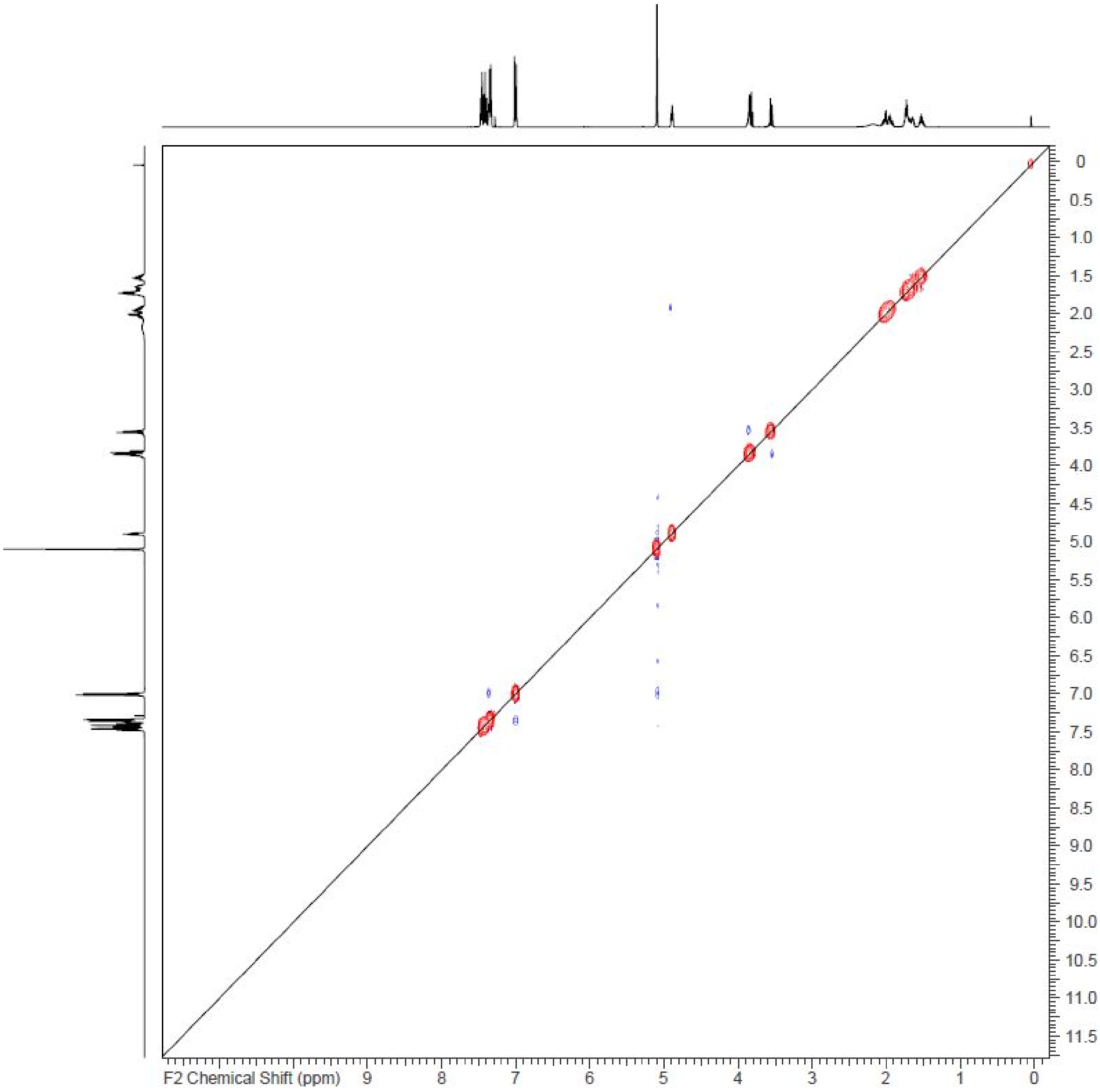

((2R,6R)-6-(naphthalen-2-yl)tetrahydro-2*H*-pyran-2-yl)methanol, **3h**

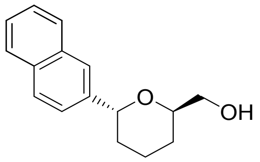

3h ^{1}H NMR (400 MHz, $CDCl_3$):

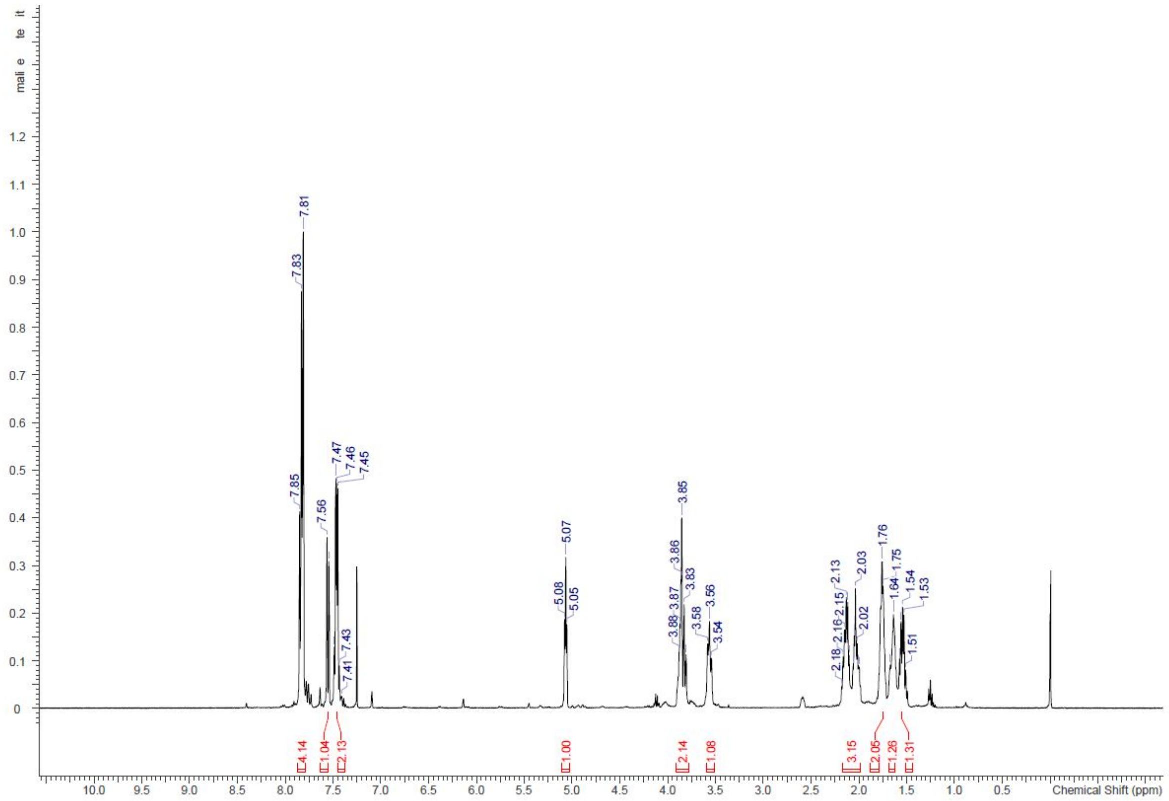

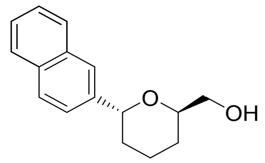

3h ^{13}C NMR (101 MHz, $CDCl_3$):

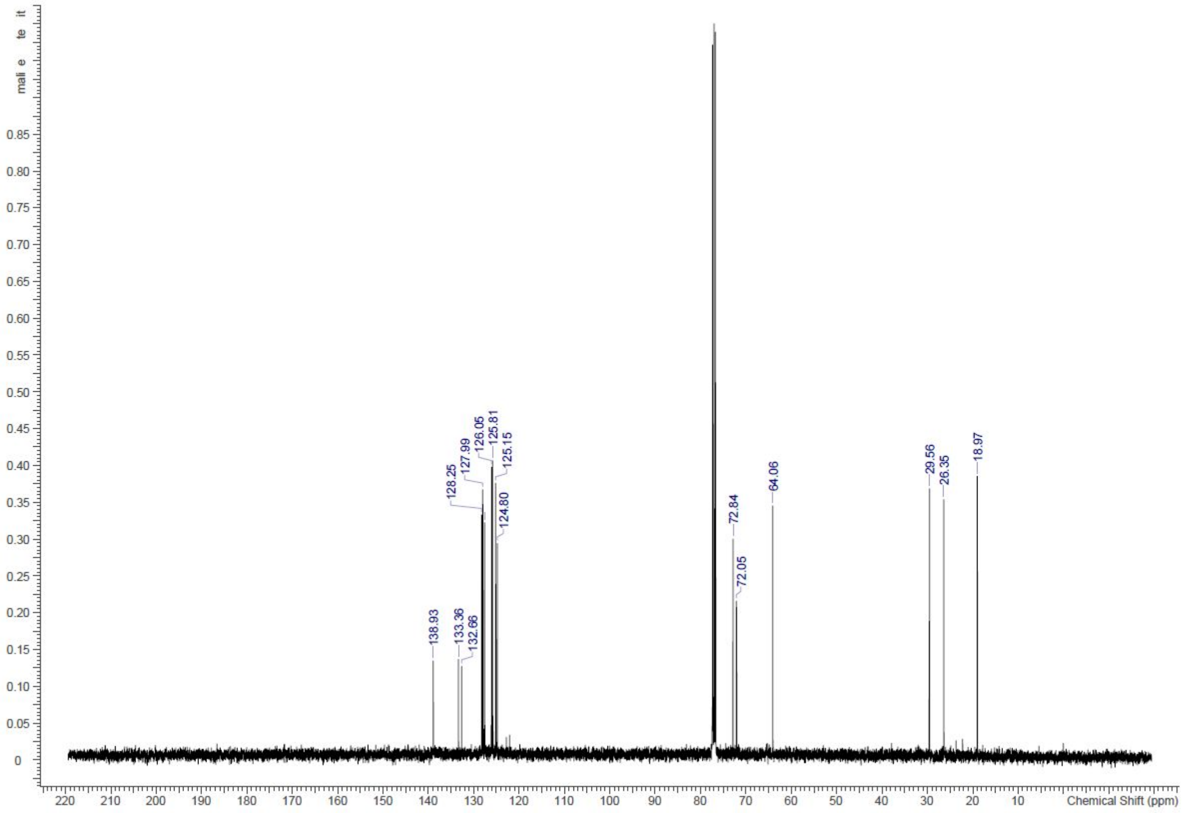

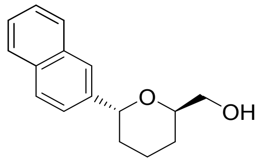

3h [^{1}H,^{1}H]-COSY (400 MHz, $CDCl_3$):

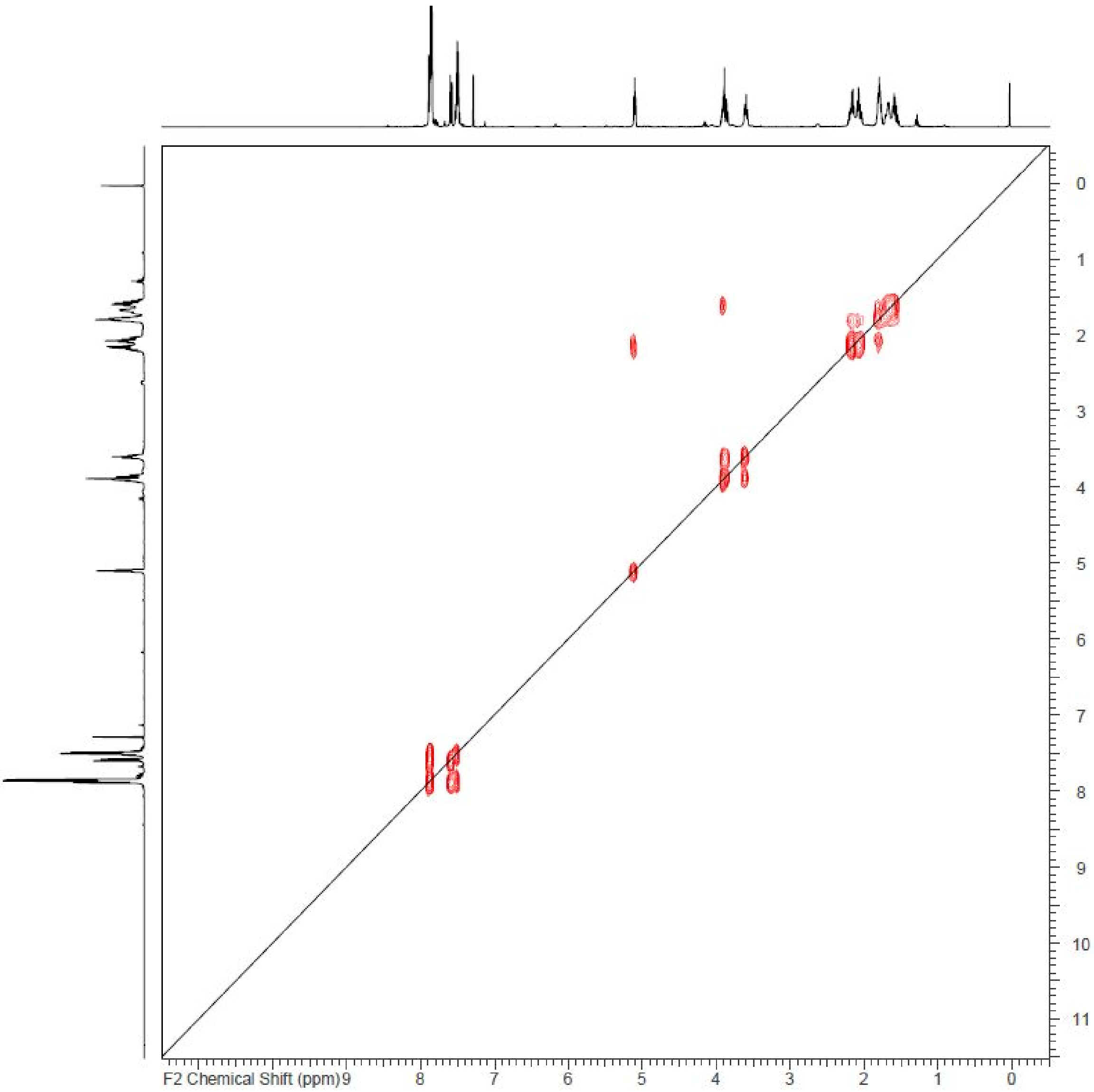

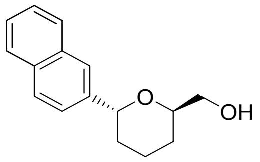

3h [^{1}H,^{13}C]-HSQC (400 MHz, 101 MHz, $CDCl_3$):

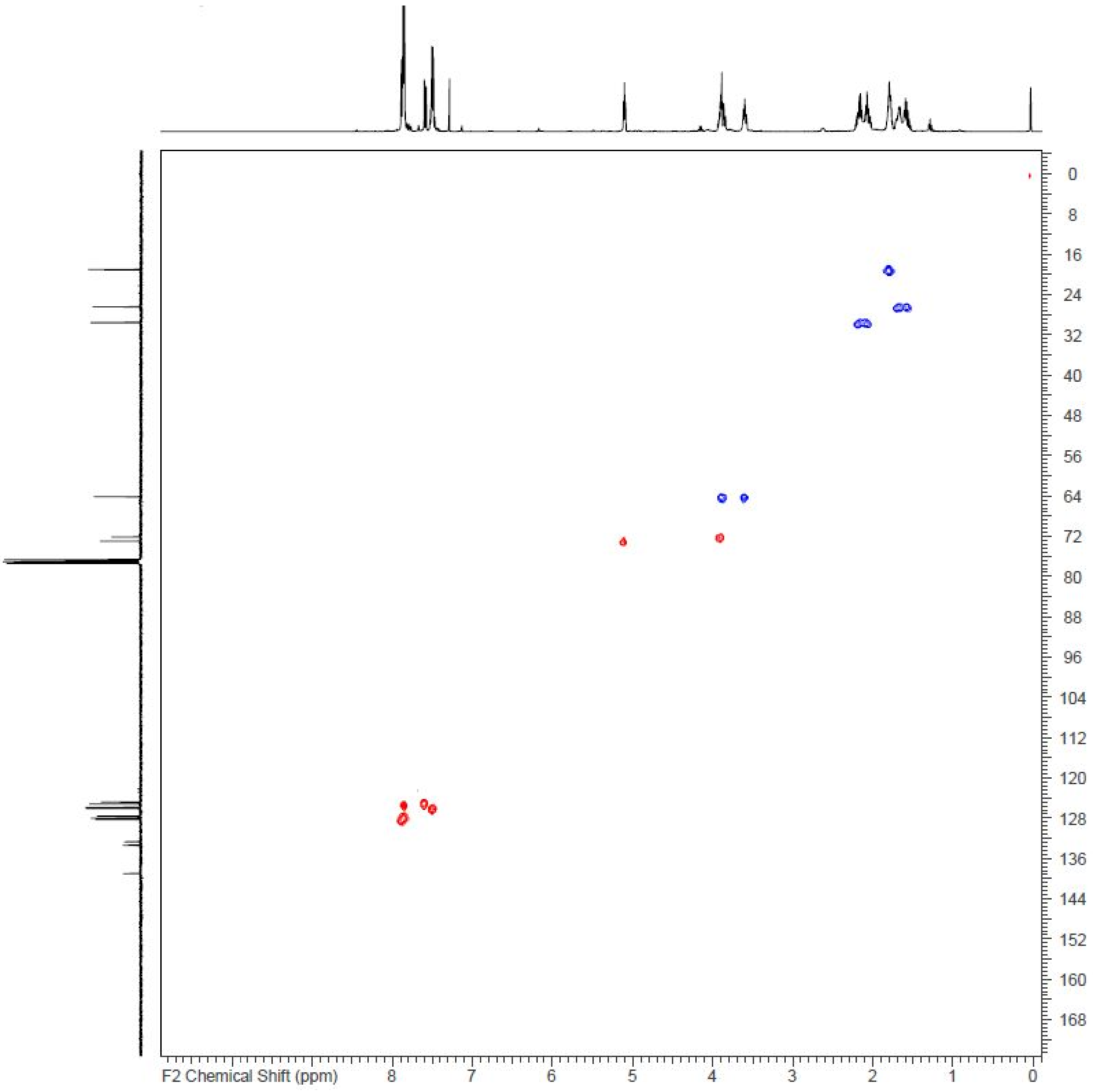

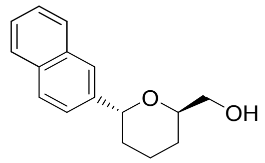

3h [^{1}H,^{13}C]-HMBC (400 MHz, 101 MHz, $CDCl_3$):

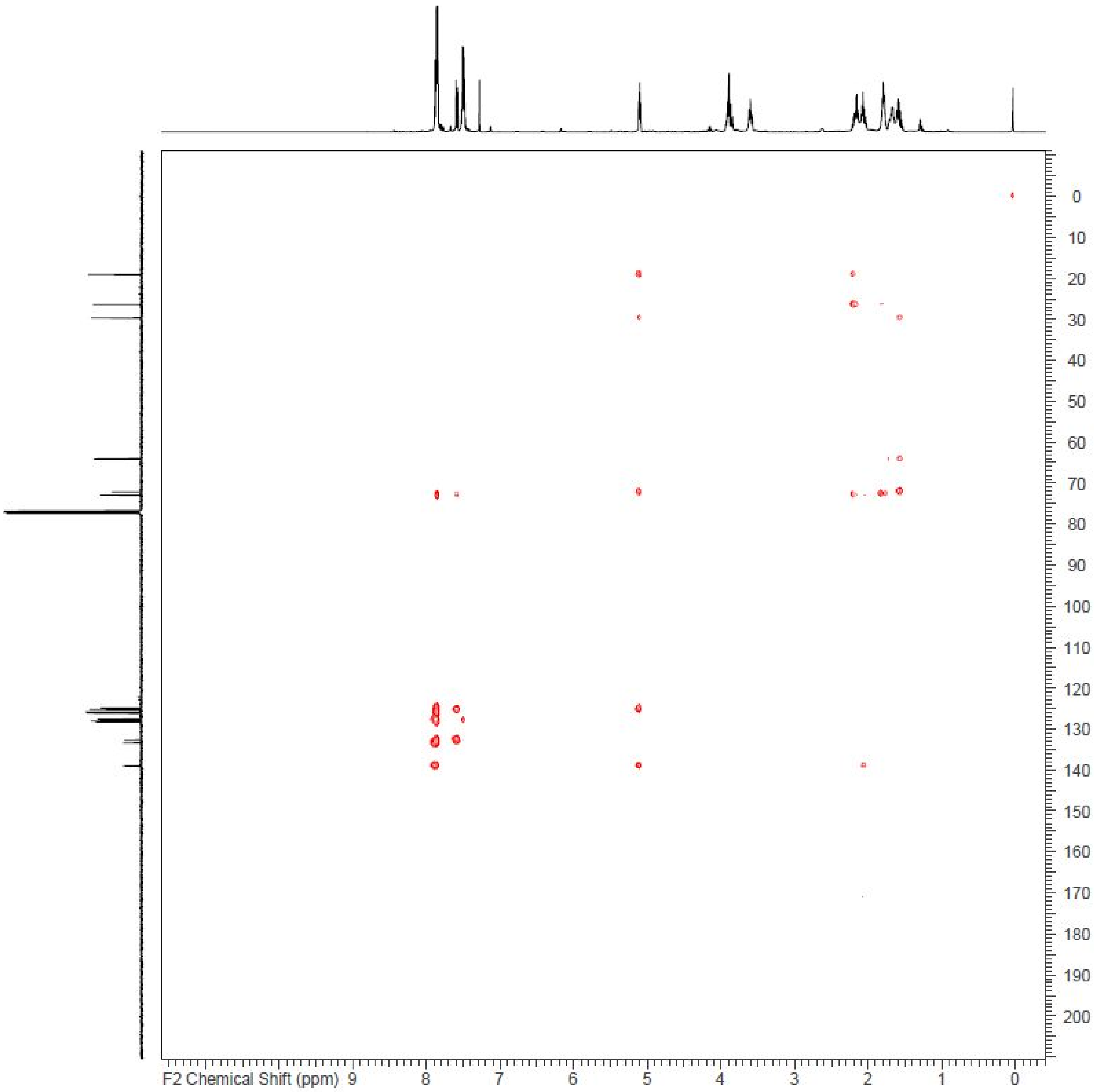

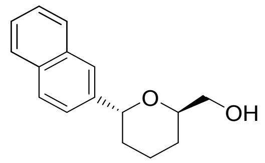

3h [^{1}H,^{1}H]-ROESY (400 MHz, $CDCl_3$):

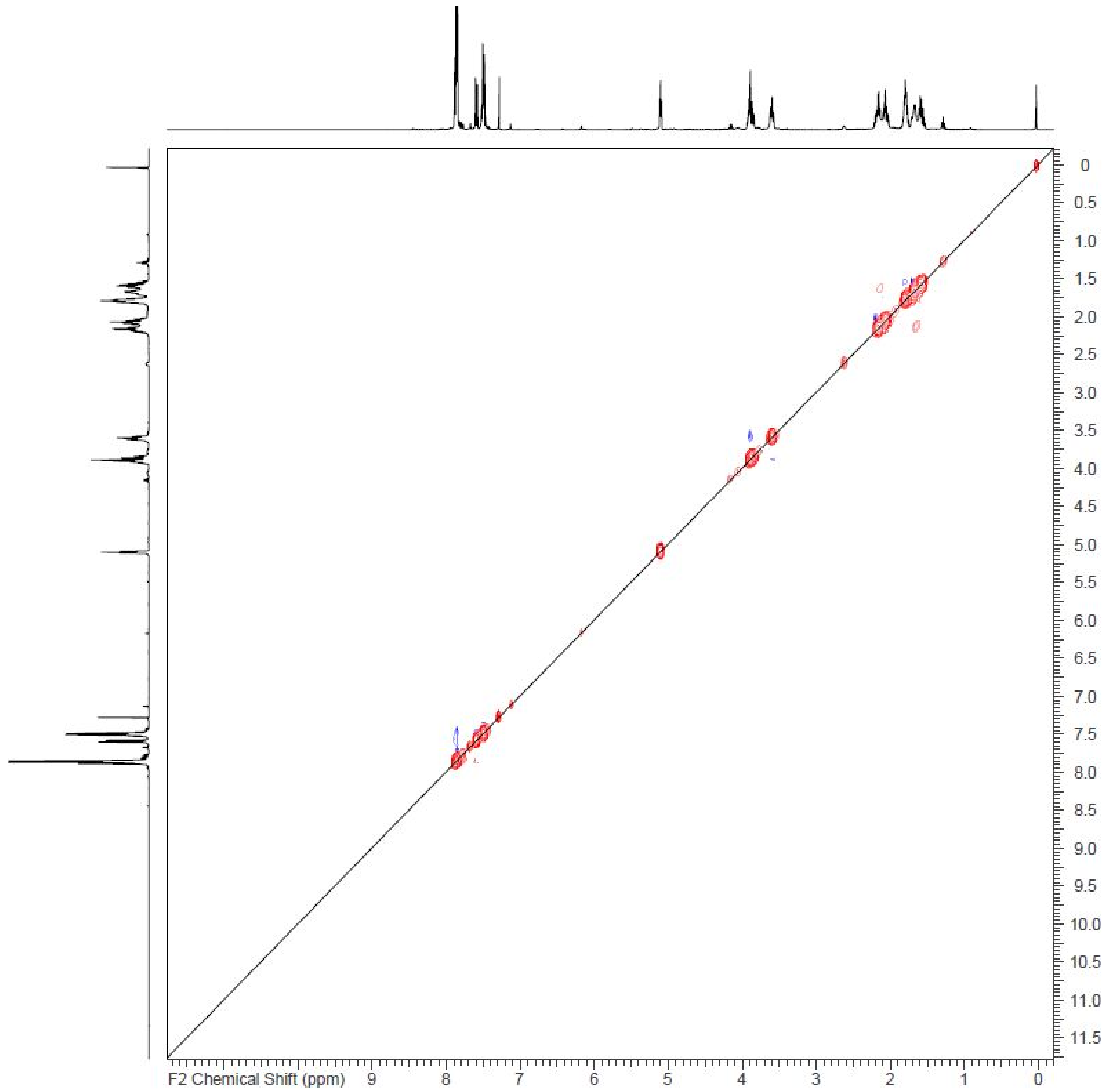

((2*R*,6*R*)-6-(*p*-tolyl)tetrahydro-2*H*-pyran-2-yl)methanol, **3i**

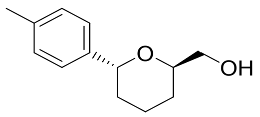

3i ^{1}H NMR (400 MHz, $CDCl_3$):

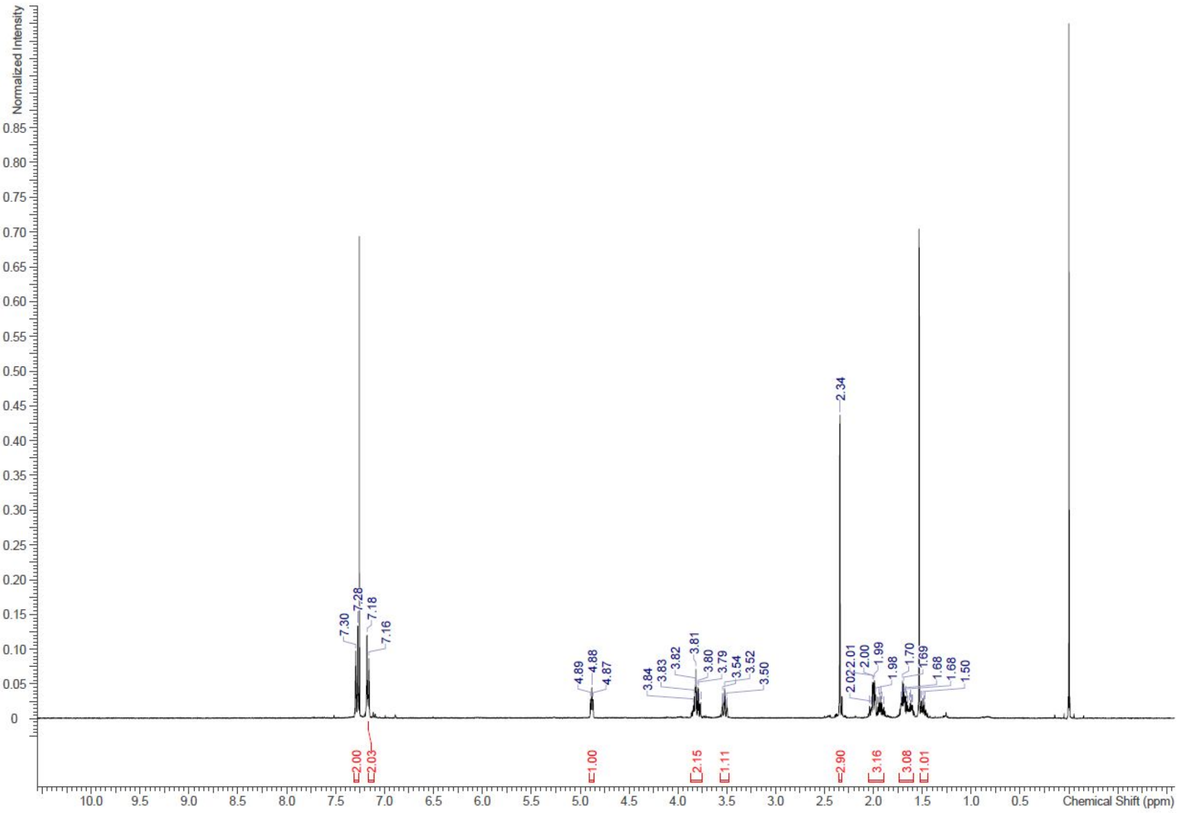

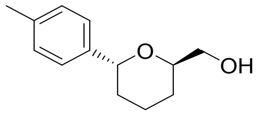

3i ^{13}C NMR (101 MHz, $CDCl_3$):

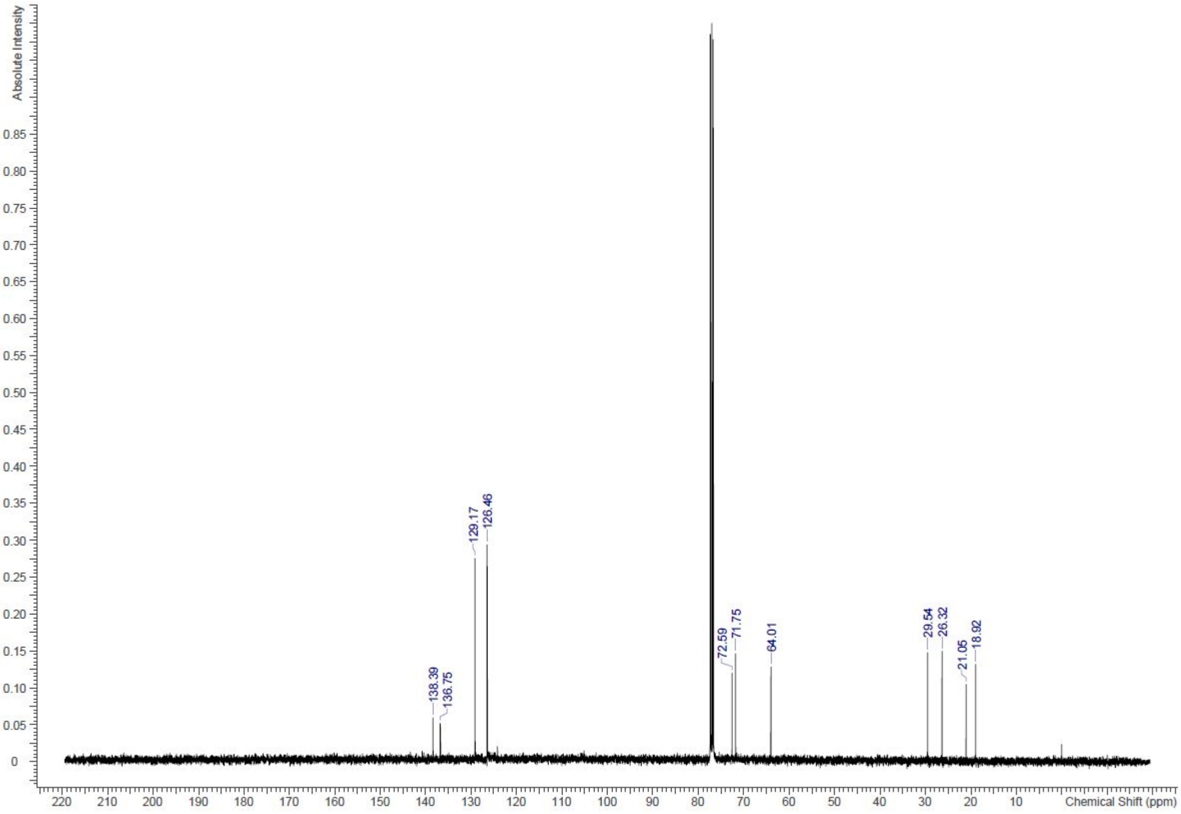

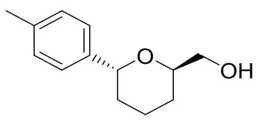

3i [^{1}H,^{1}H]-COSY (400 MHz, $CDCl_3$):

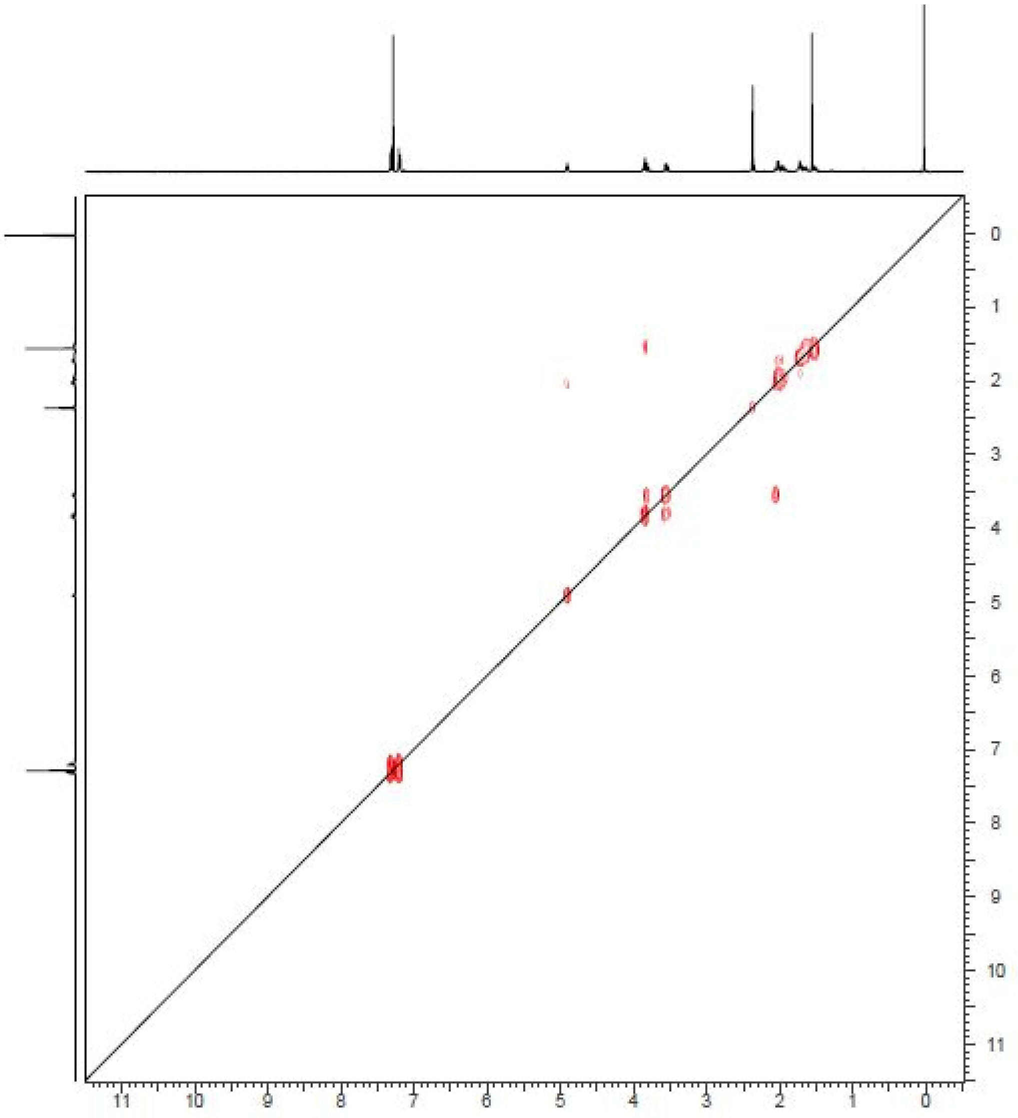

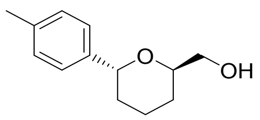

3i [^{1}H,^{13}C]-HSQC (400 MHz, 101 MHz, $CDCl_3$):

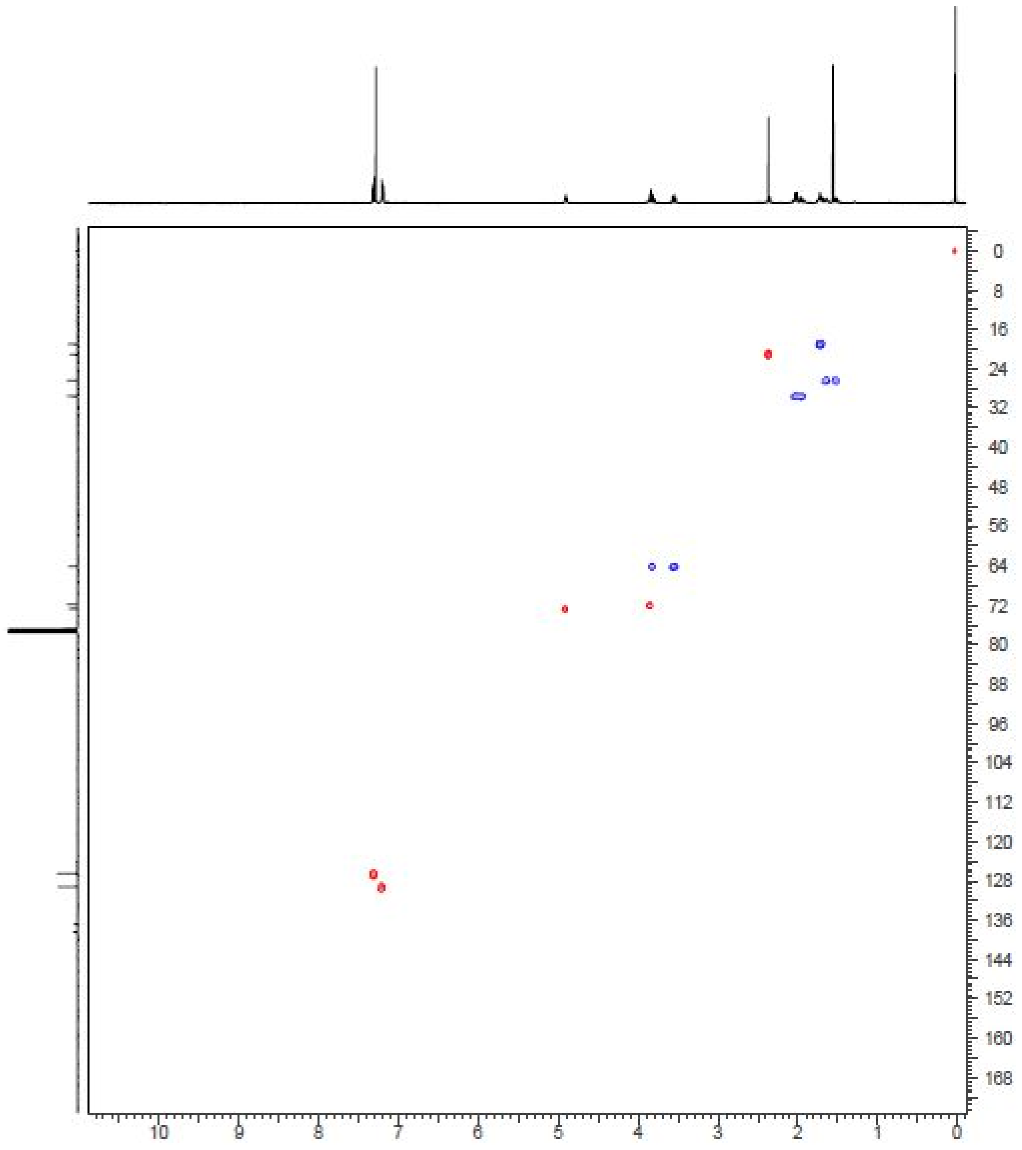

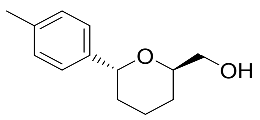

3i [^{1}H,^{13}C]-HMBC (400 MHz, 101 MHz, $CDCl_3$):

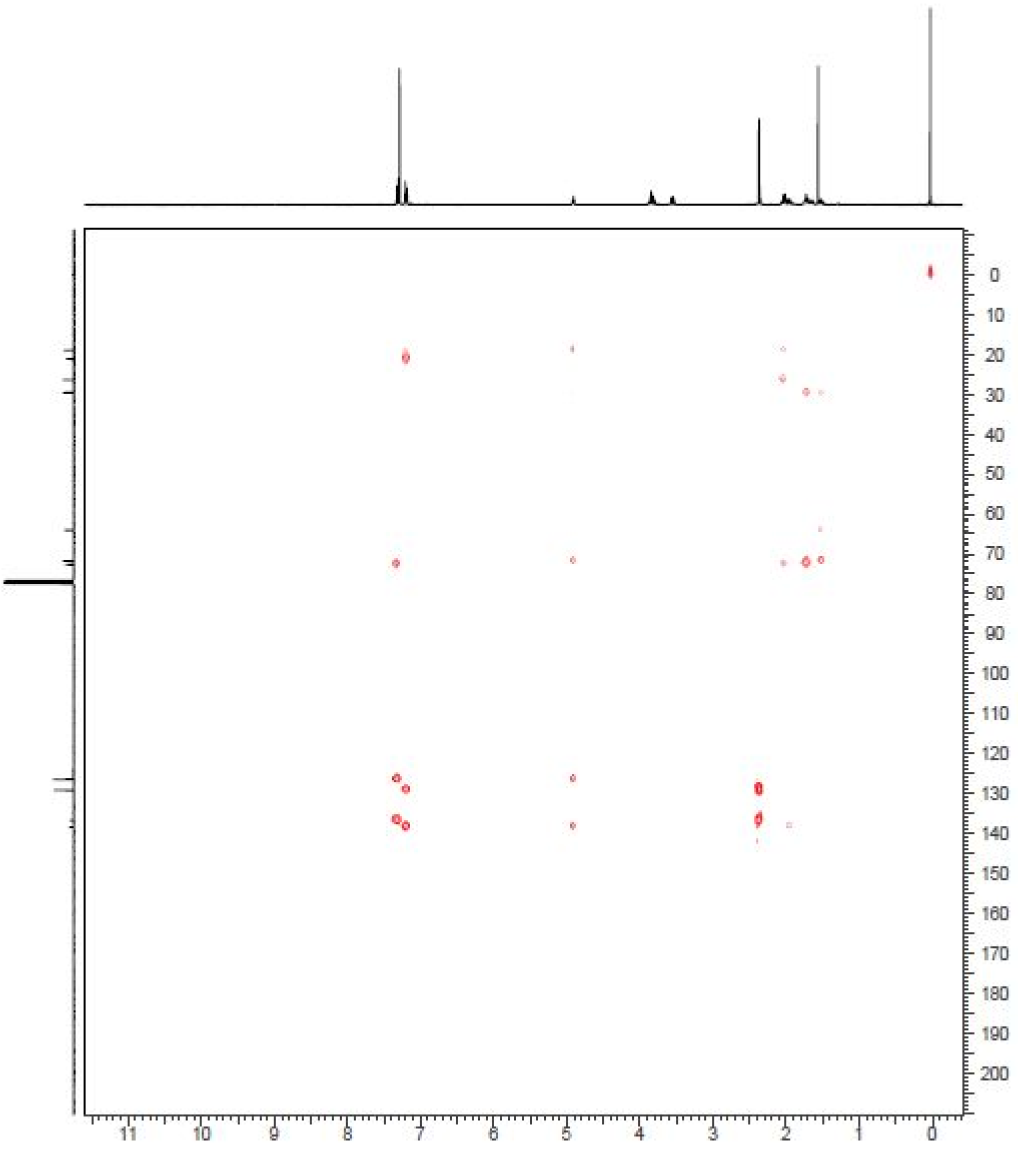

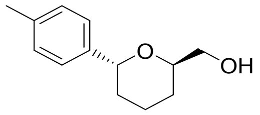

3i [^{1}H,^{1}H]-ROESY (400 MHz, $CDCl_3$):

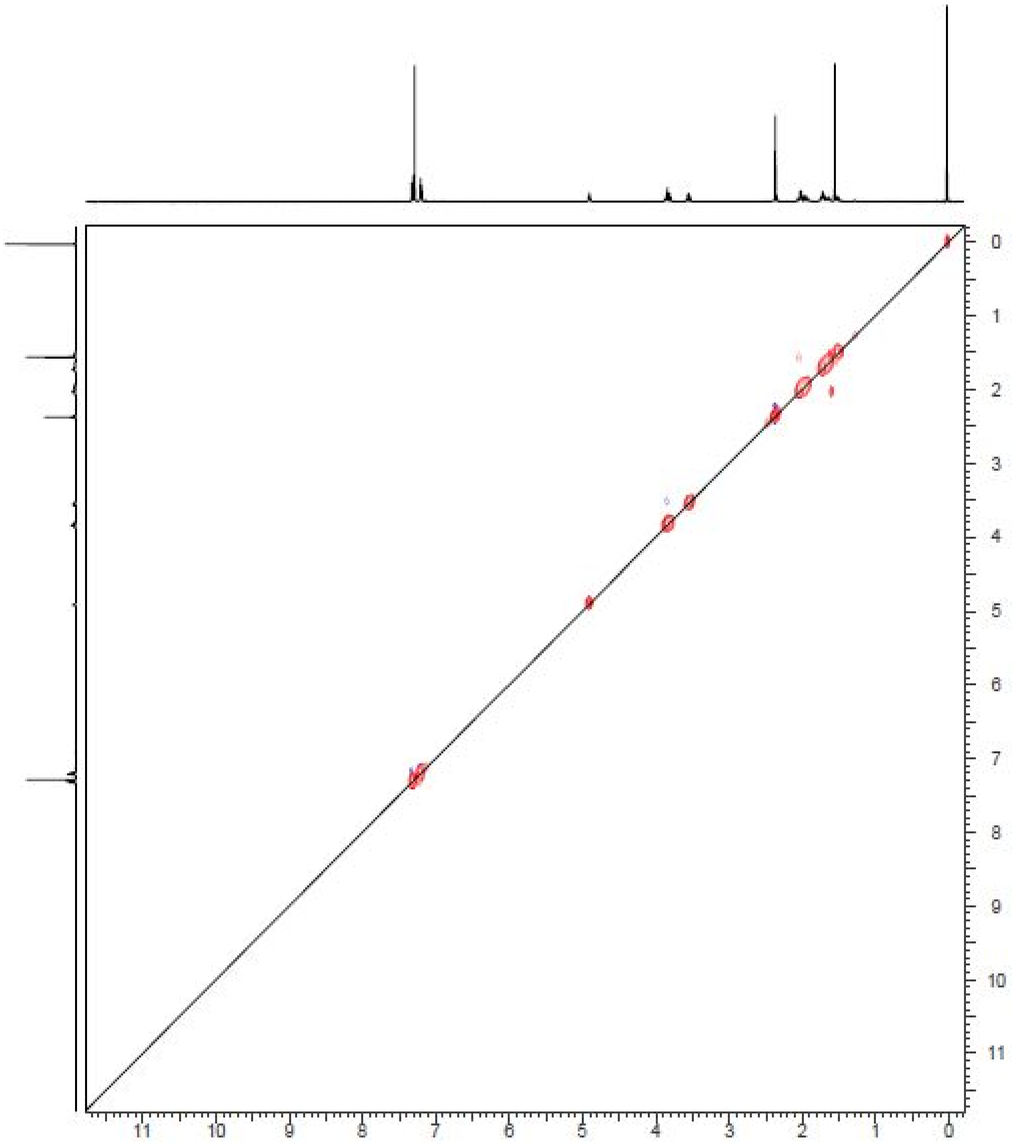

((2*R*,6*R*)-6-(4-chlorophenyl)tetrahydro-2*H*-pyran-2-yl)methanol, **3j**:

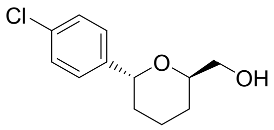

3j ^{1}H NMR (400 MHz, $CDCl_3$):

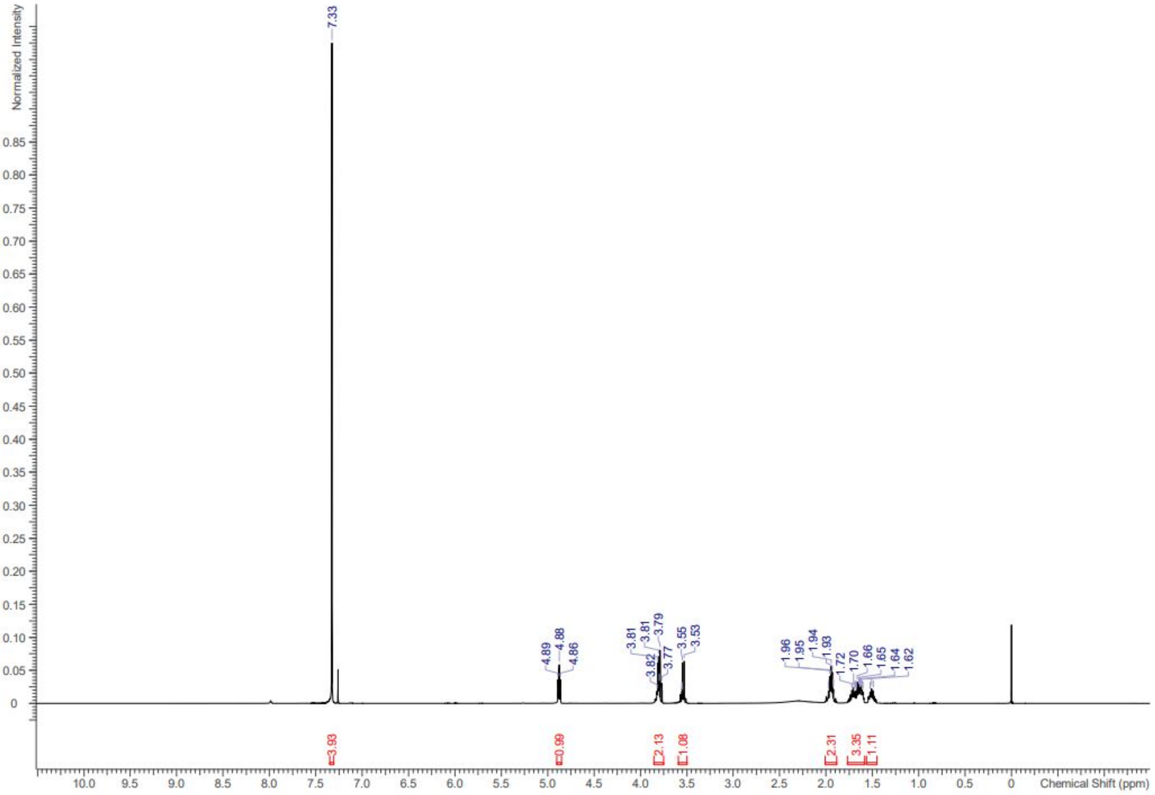

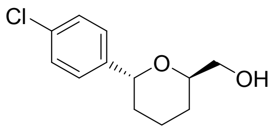

3j ^{13}C NMR (101 MHz, $CDCl_3$):

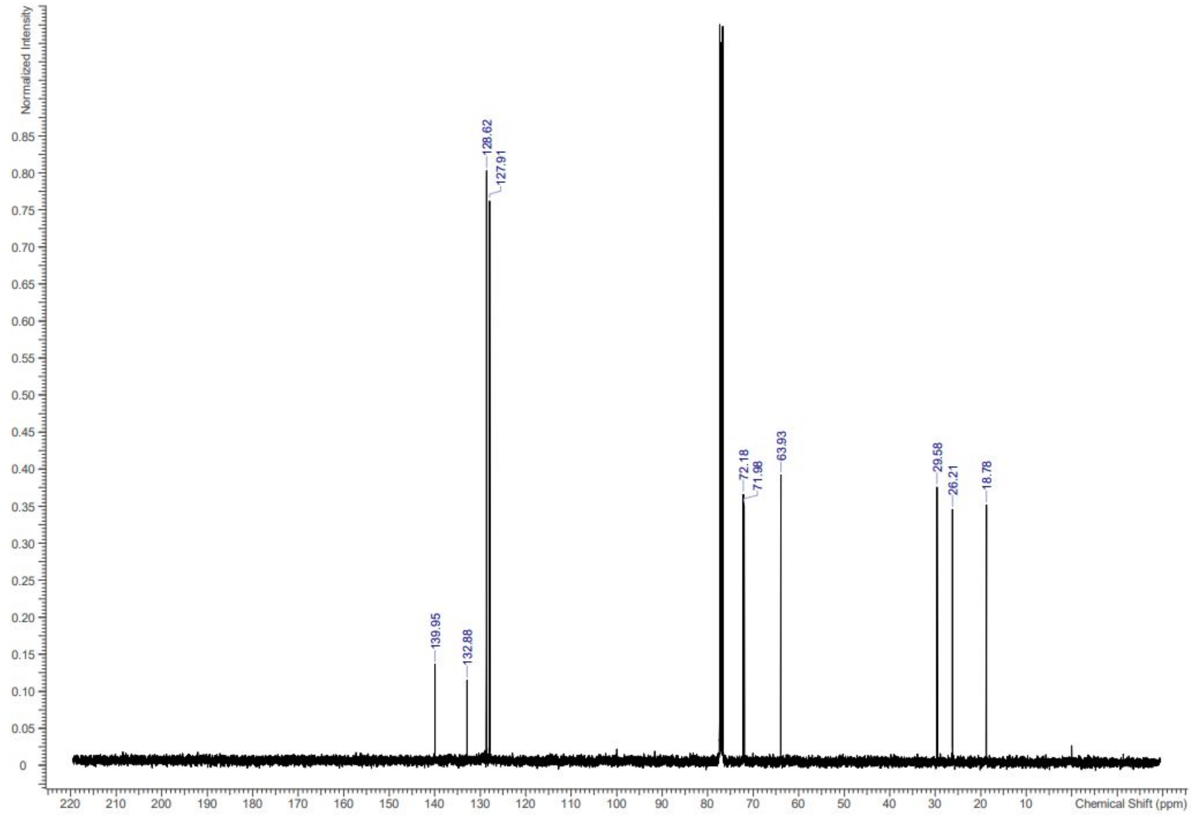

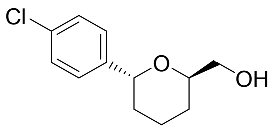

3j [^{1}H,^{1}H]-COSY (400 MHz, $CDCl_3$):

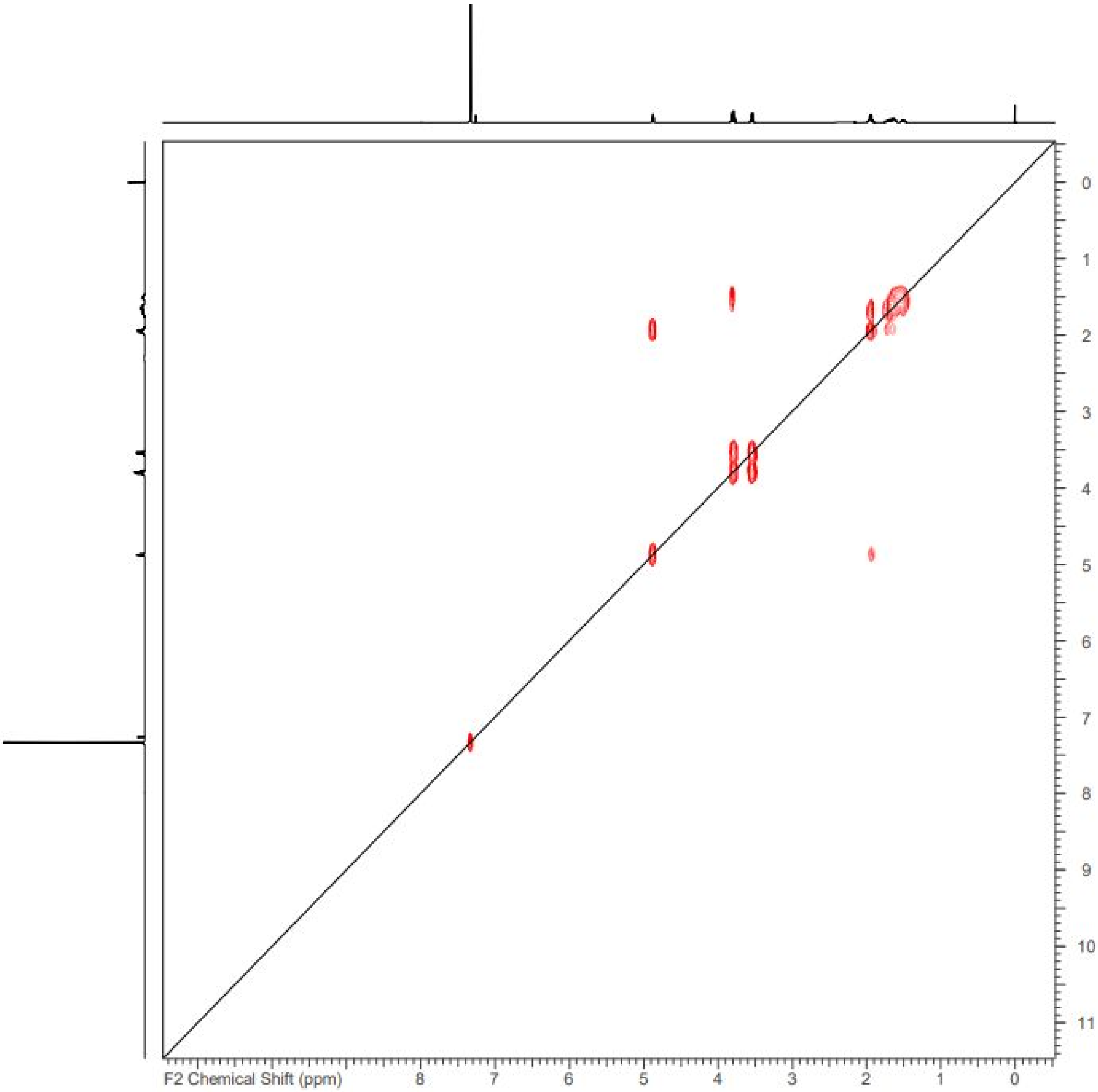

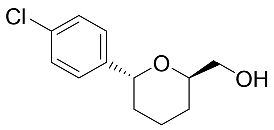

3j [^{1}H,^{13}C]-HSQC (400 MHz, 101 MHz, $CDCl_3$):

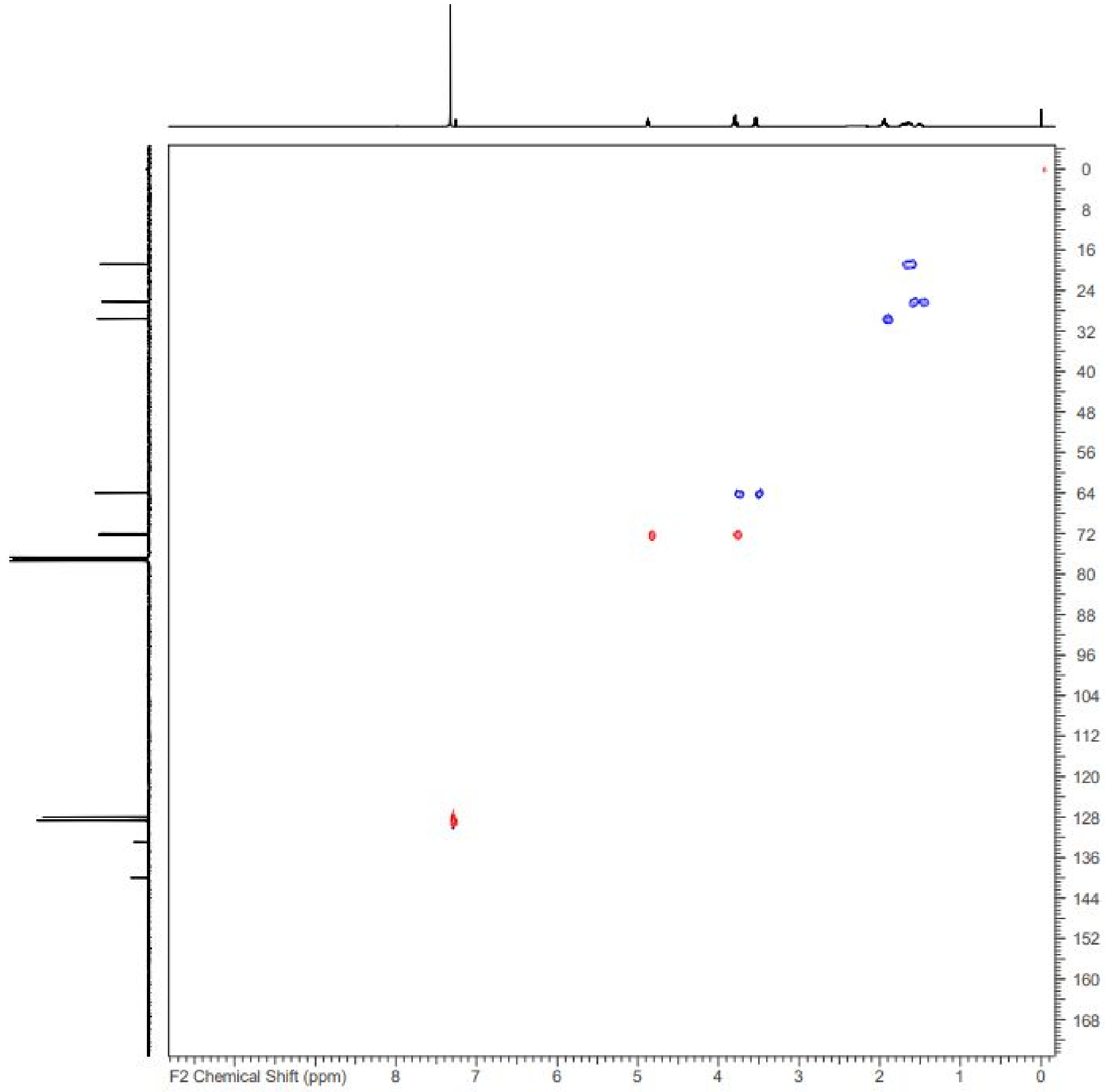

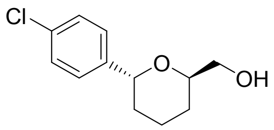

3j [^{1}H,^{13}C]-HMBC (400 MHz, 101 MHz, $CDCl_3$):

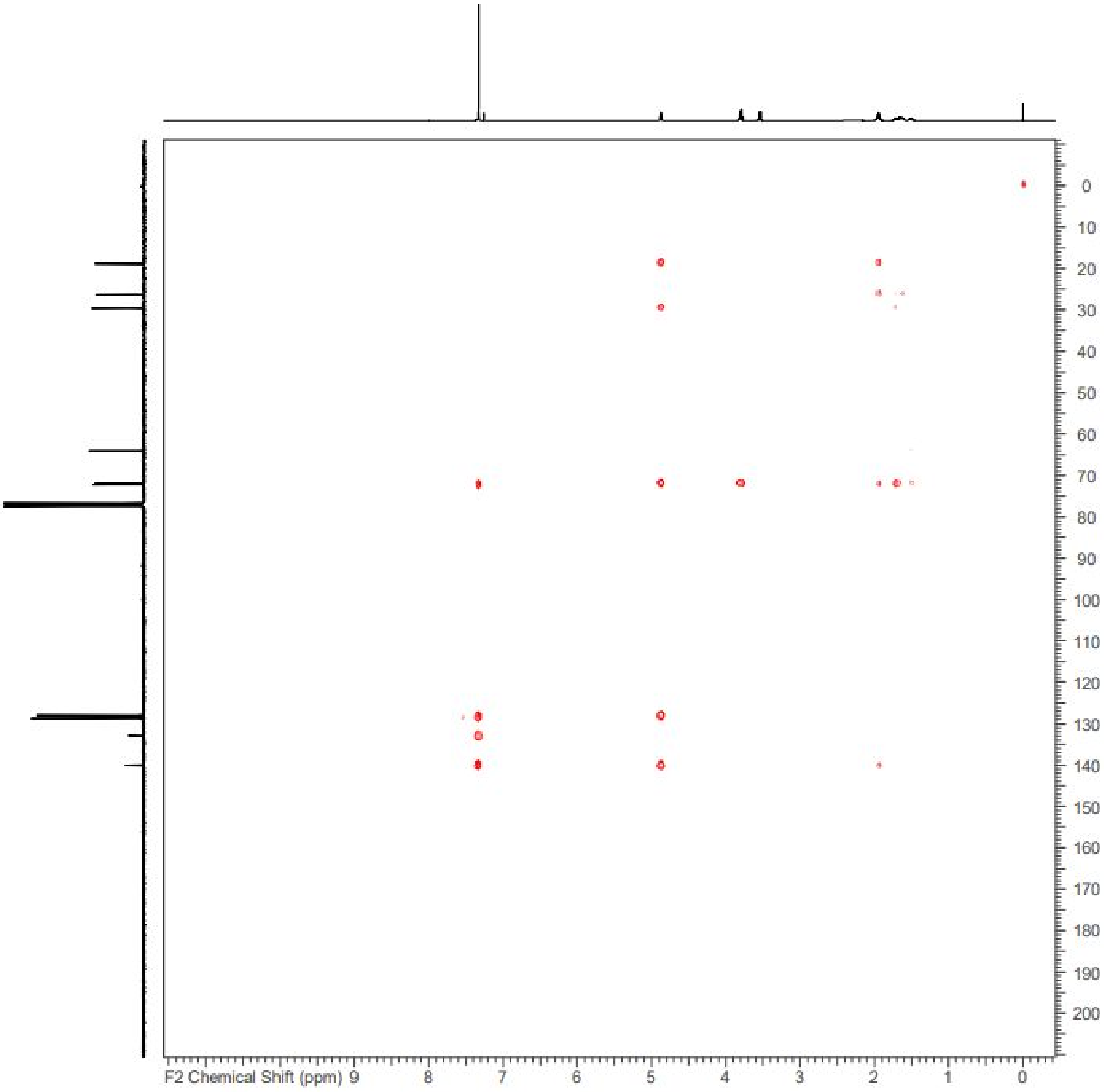

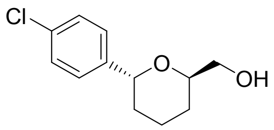

3j [^{1}H,^{1}H]-ROESY (400 MHz, $CDCl_3$):

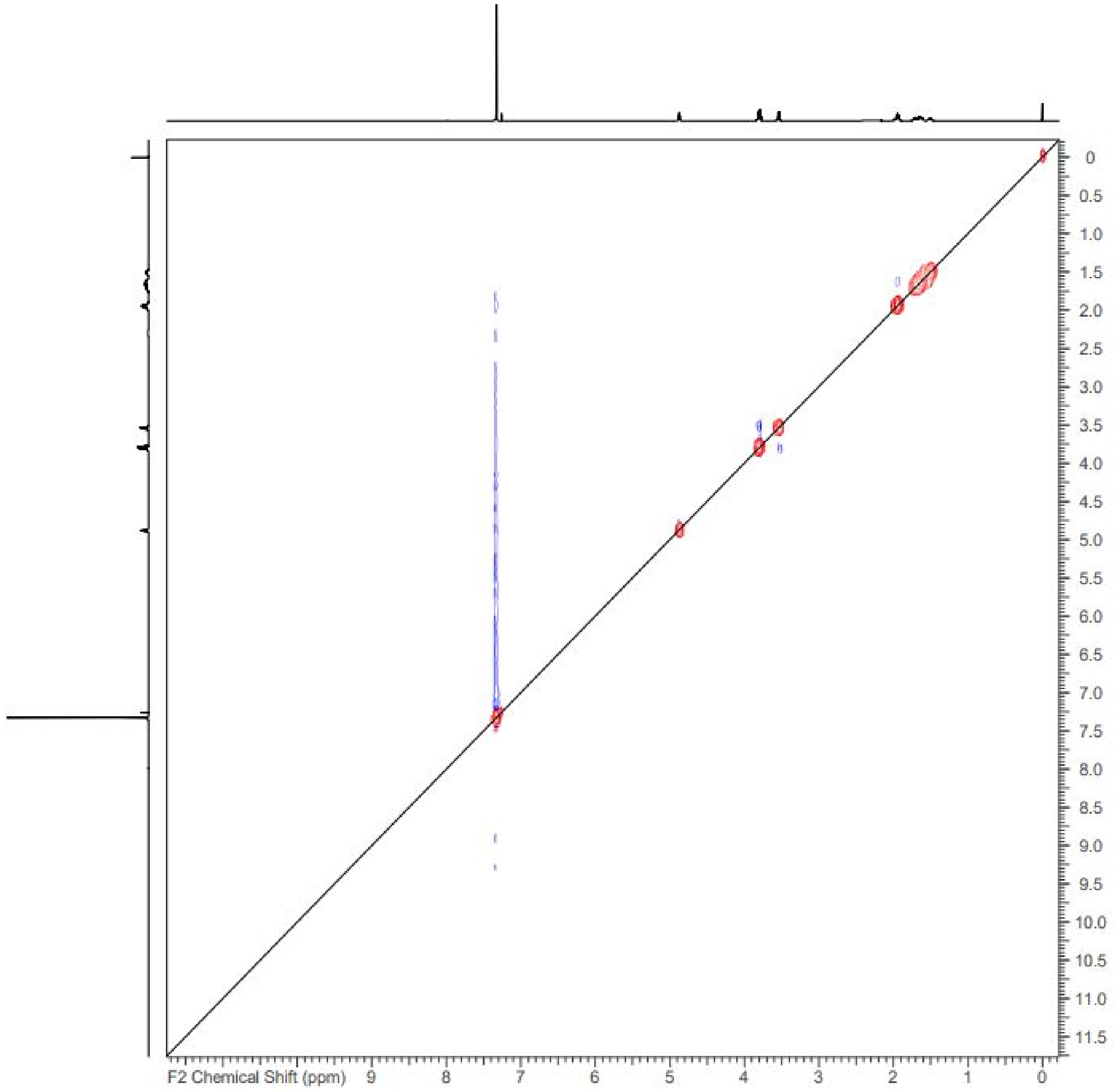

((2*R*,6*R*)-6-(4-(trifluoromethyl)phenyl)tetrahydro-2*H*-pyran-2-yl)methanol, **3k**:

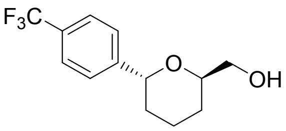

3k ^{1}H NMR (400 MHz, $CDCl_3$):

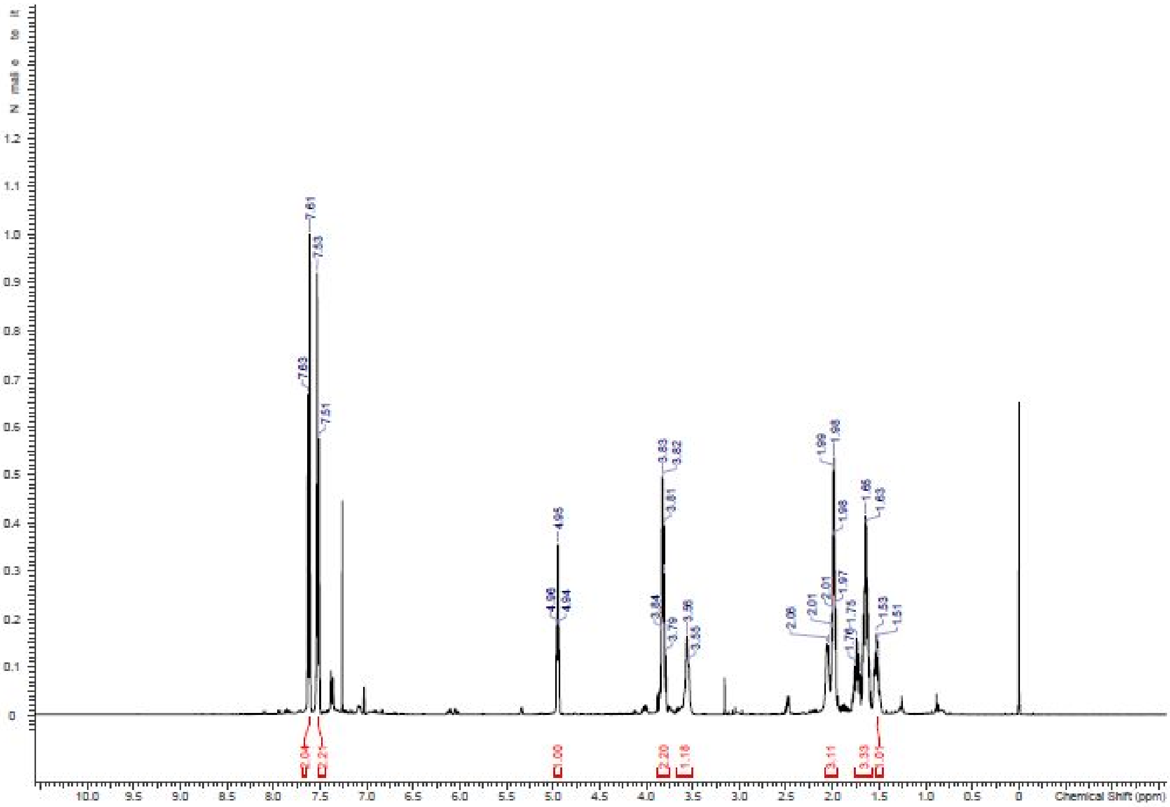

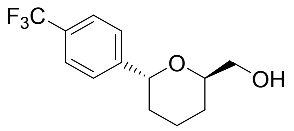

3k ^{13}C NMR (101 MHz, $CDCl_3$):

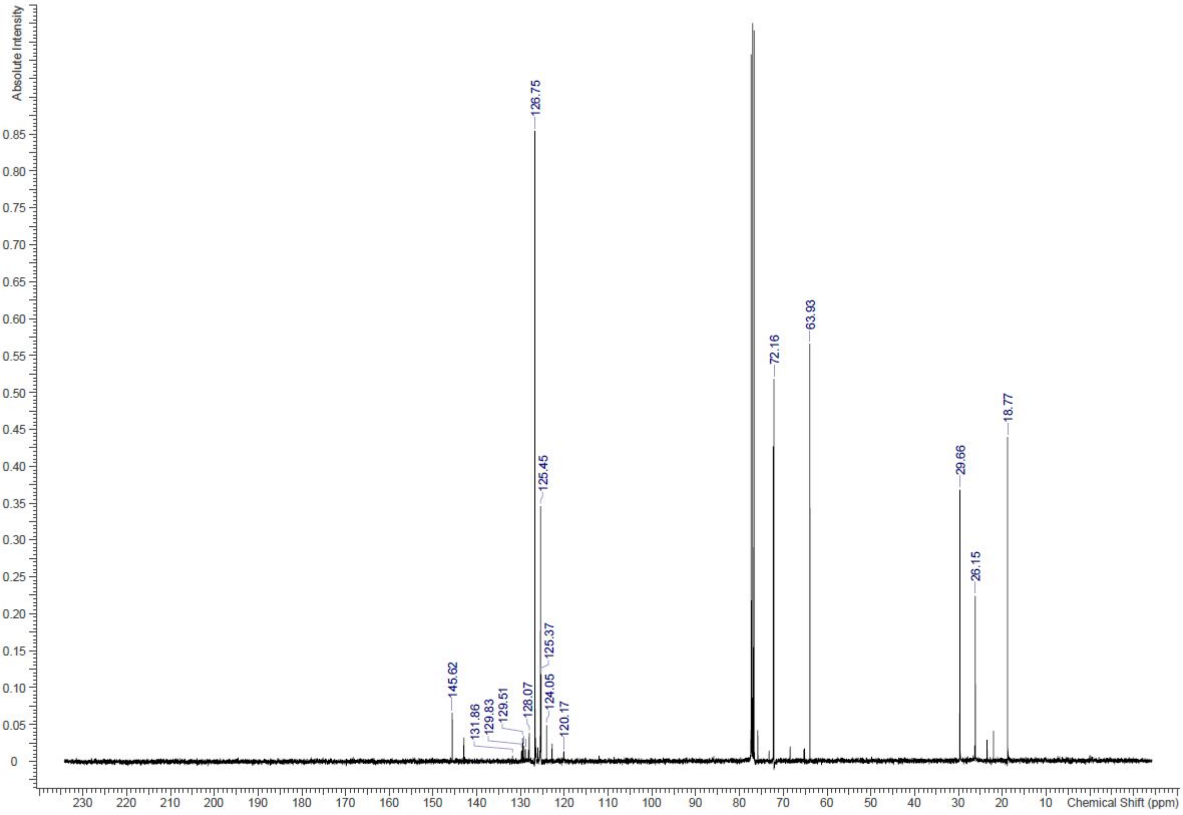

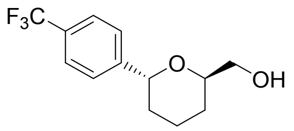

3k $^{19}F\{^{1}H\}$ NMR (376 MHz, $CDCl_3$):

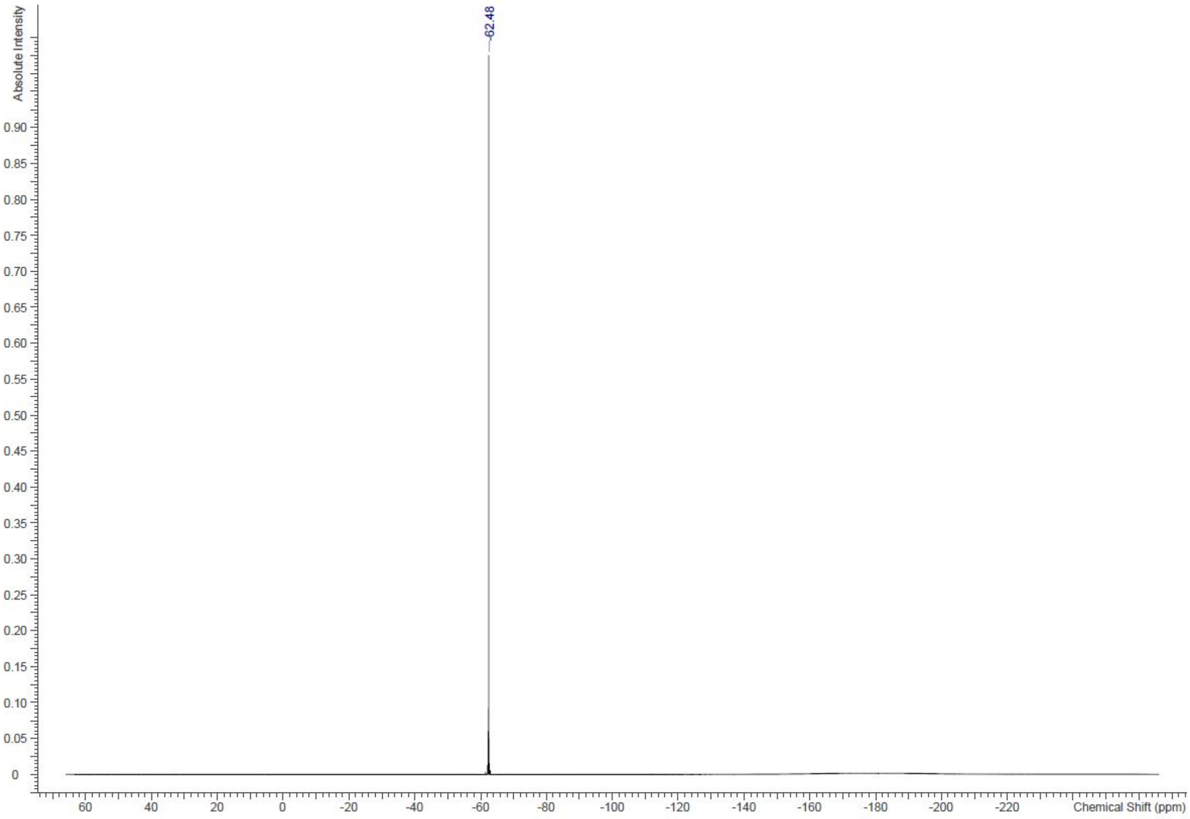

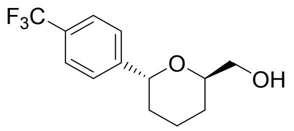

3k [^{1}H,^{1}H]-COSY (400 MHz, $CDCl_3$):

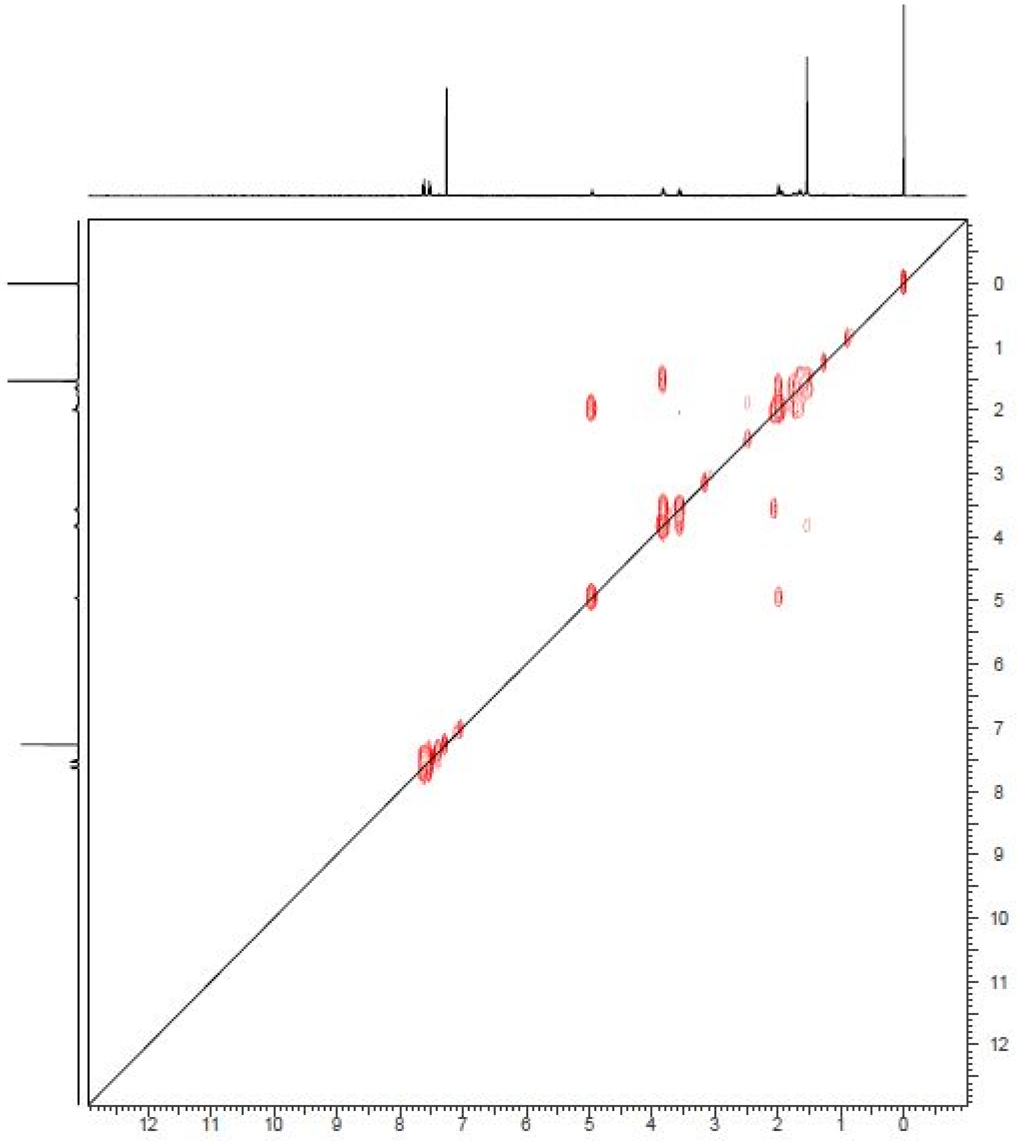

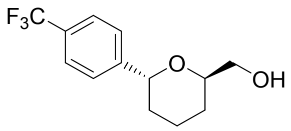

3k [^{1}H,^{13}C]-HSQC (400 MHz, 101 MHz, $CDCl_3$):

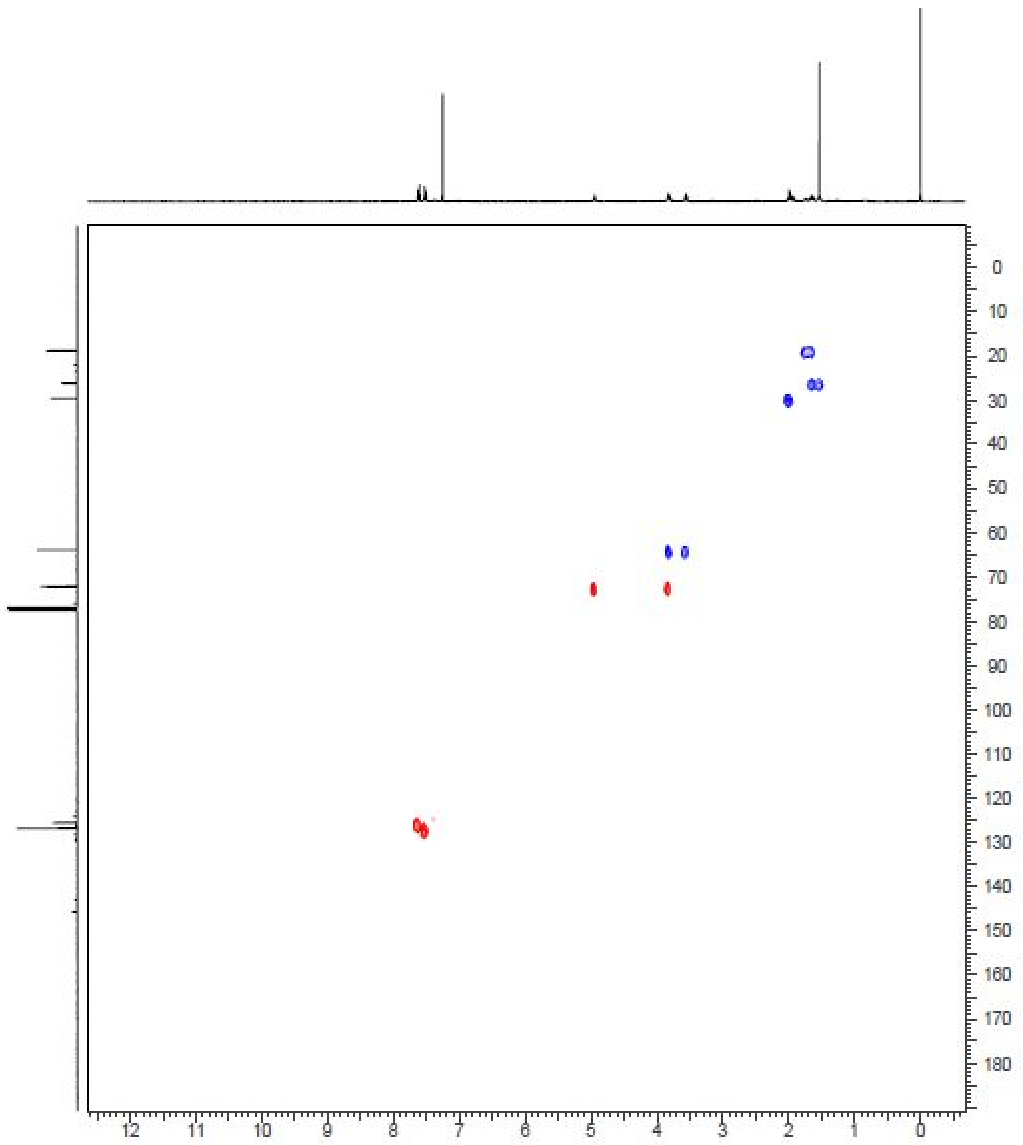

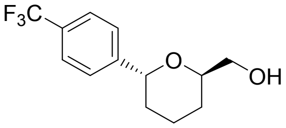

3k [1H,13C]-HMBC (400 MHz, 101 MHz, $CDCl_3$):

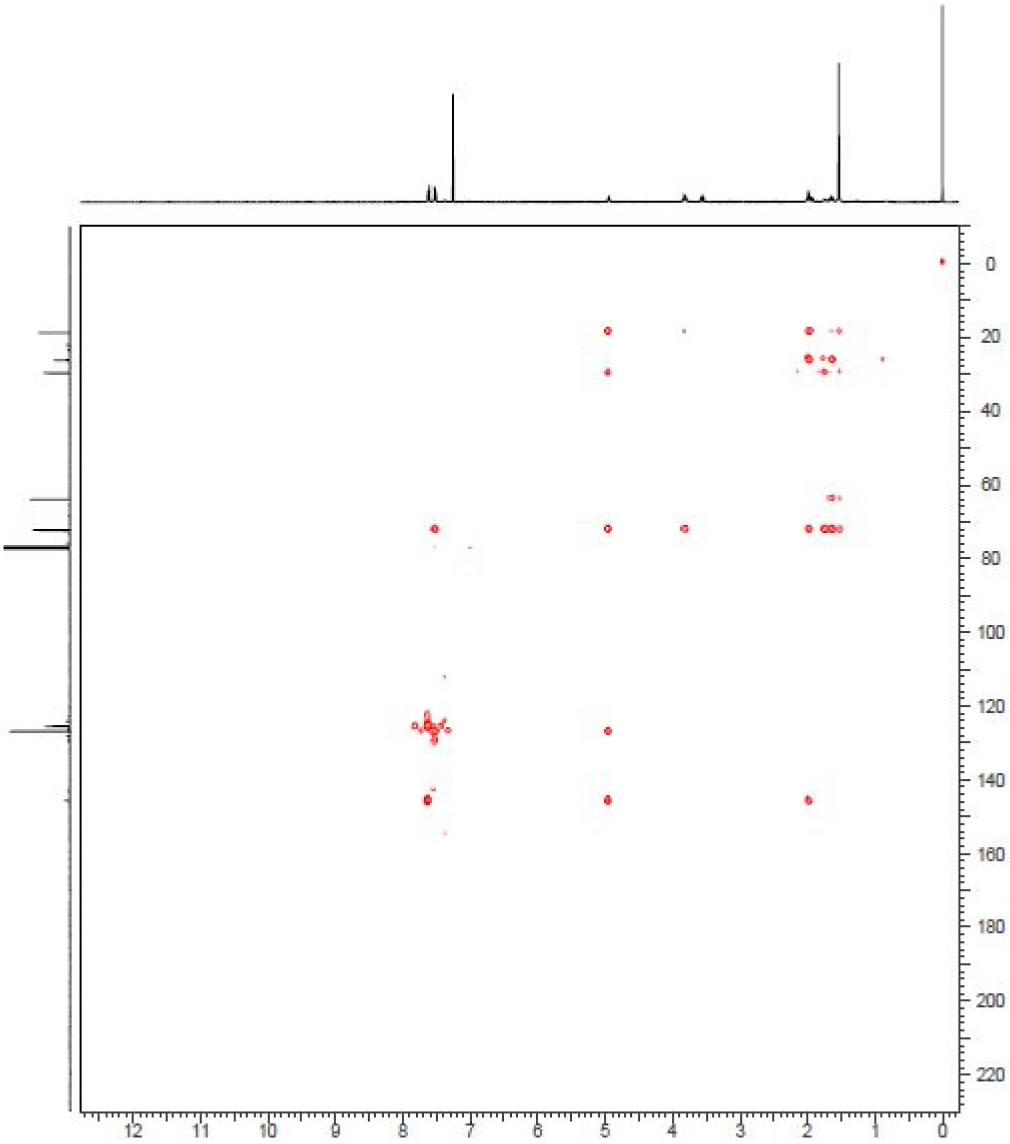

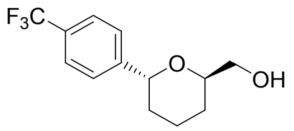

3k [^{1}H,^{1}H]-ROESY (400 MHz, $CDCl_3$):

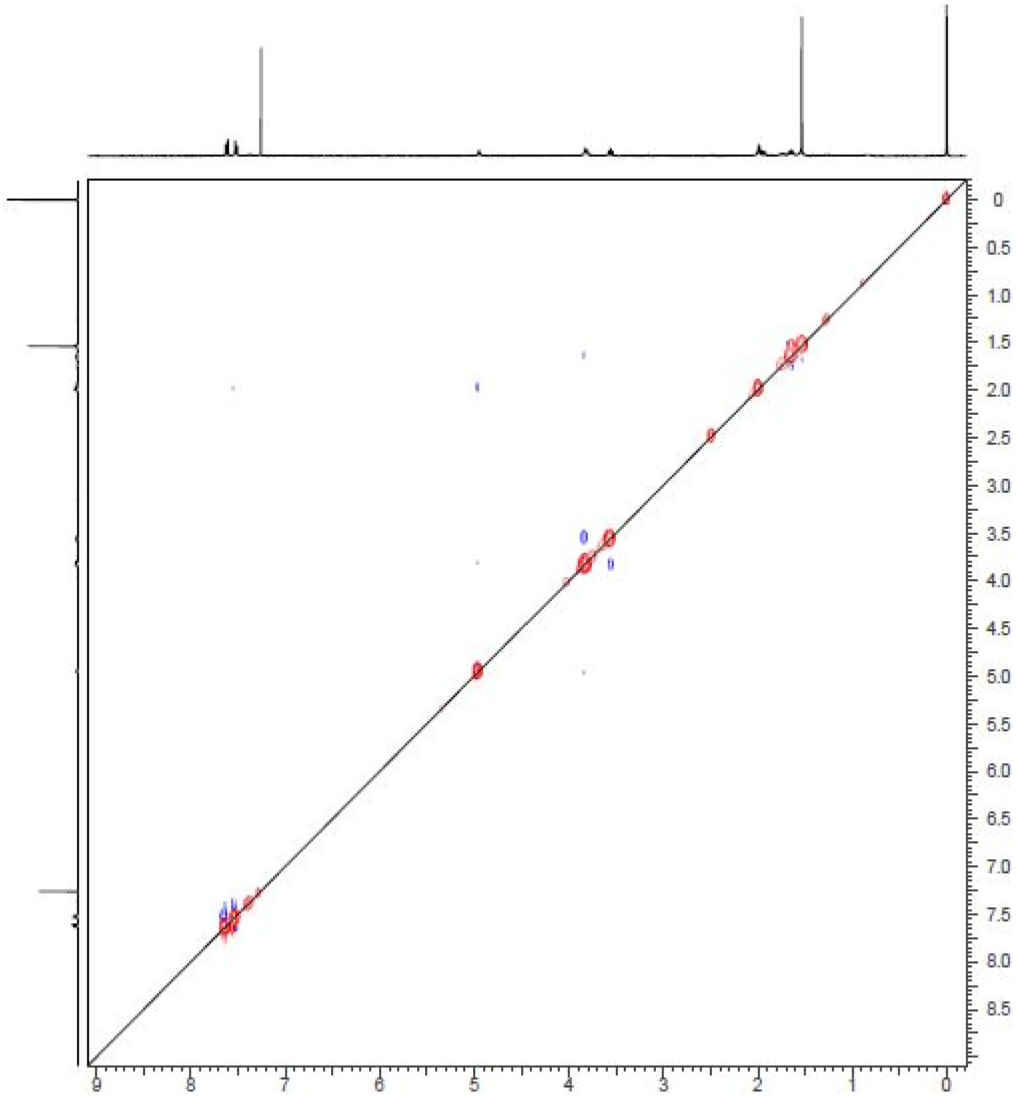

methyl 4-((2*R*,6*R*)-6-(hydroxymethyl)tetrahydro-2*H*-pyran-2-yl)benzoate, **3l**:

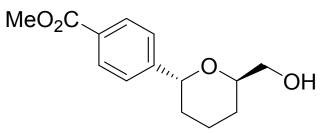

3l ^{1}H NMR (400 MHz, $CDCl_3$):

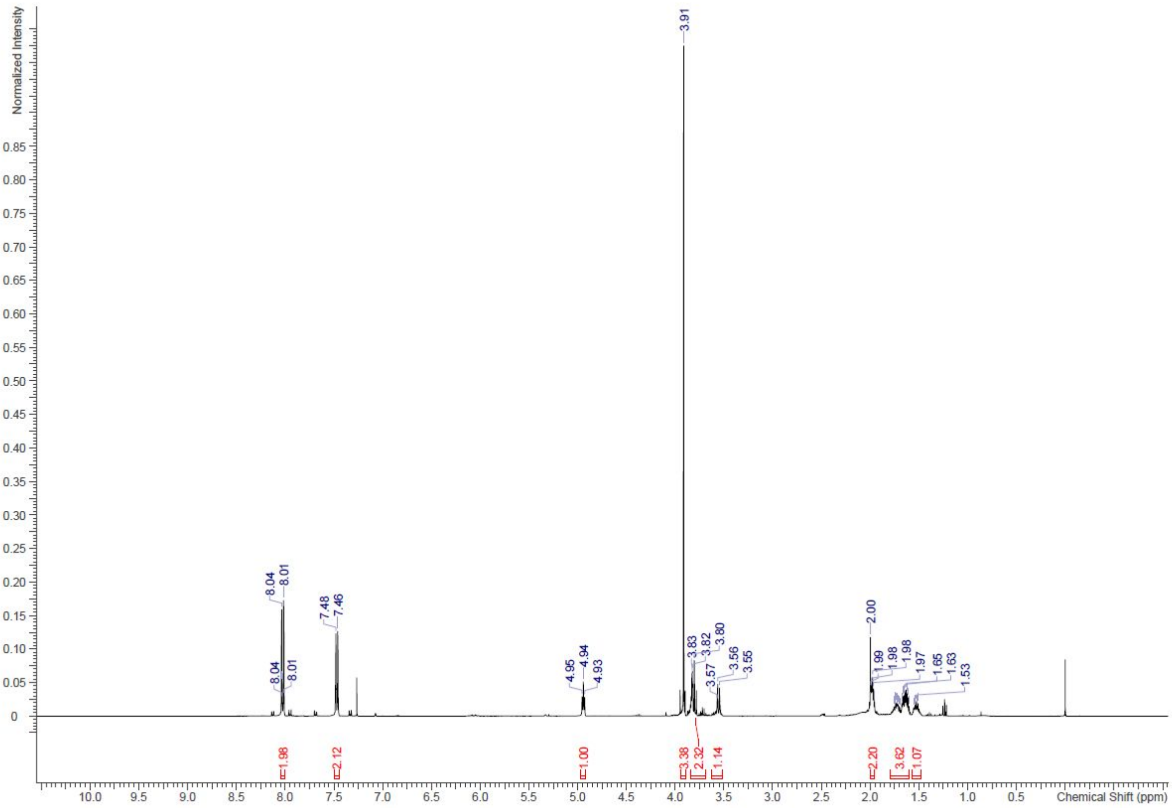

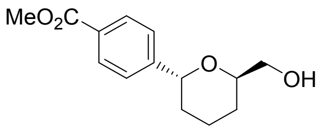

3l ^{13}C NMR (101 MHz, $CDCl_3$):

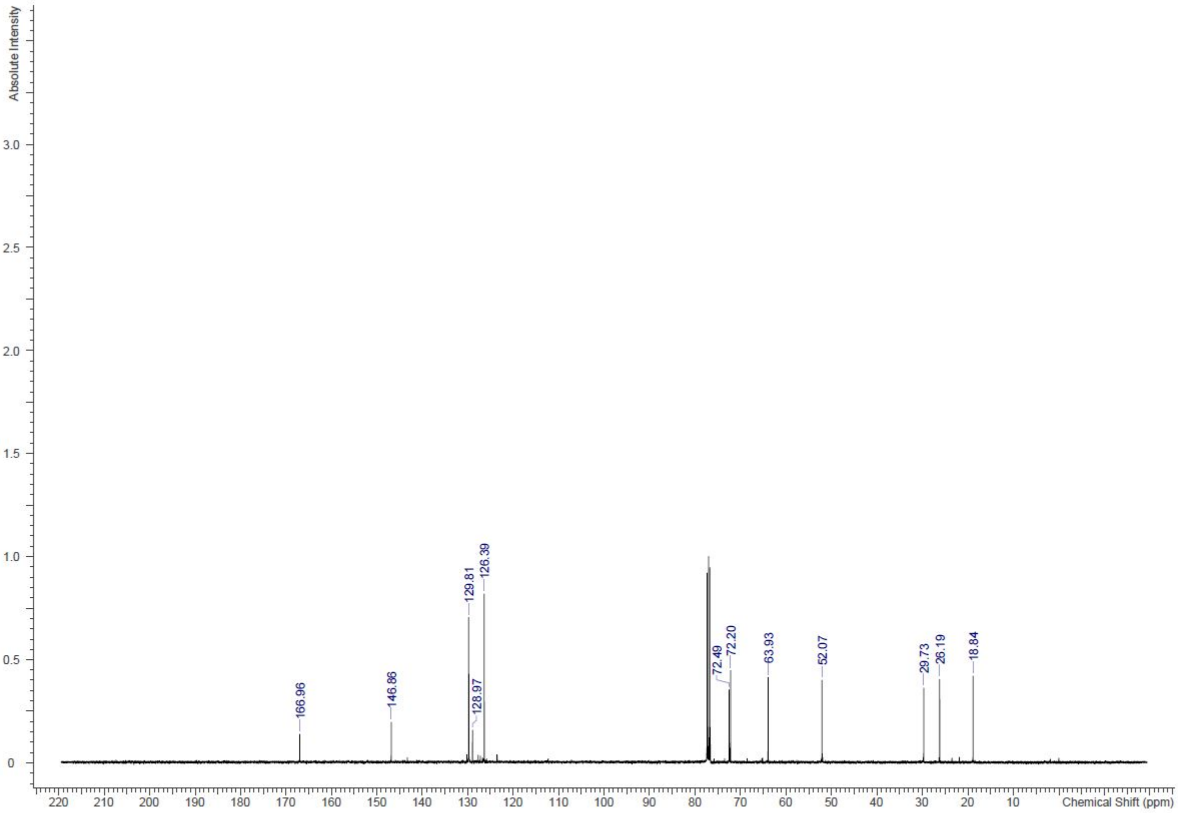

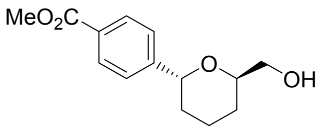

3l [1H,1H]-COSY (400 MHz, $CDCl_3$):

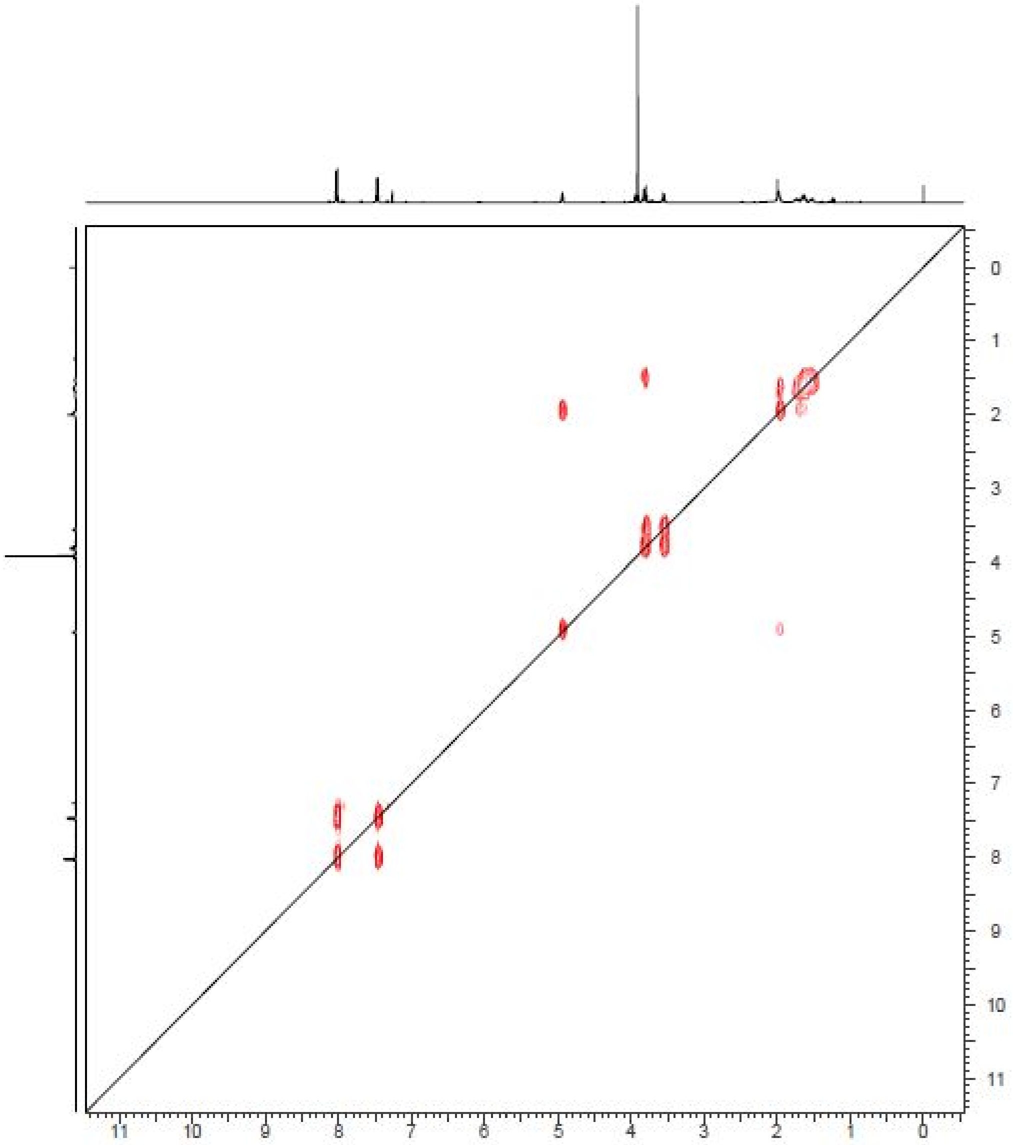

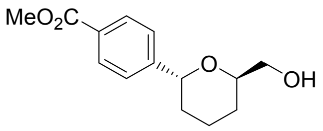

3l [^{1}H,^{13}C]-HSQC (400 MHz, 101 MHz, $CDCl_3$):

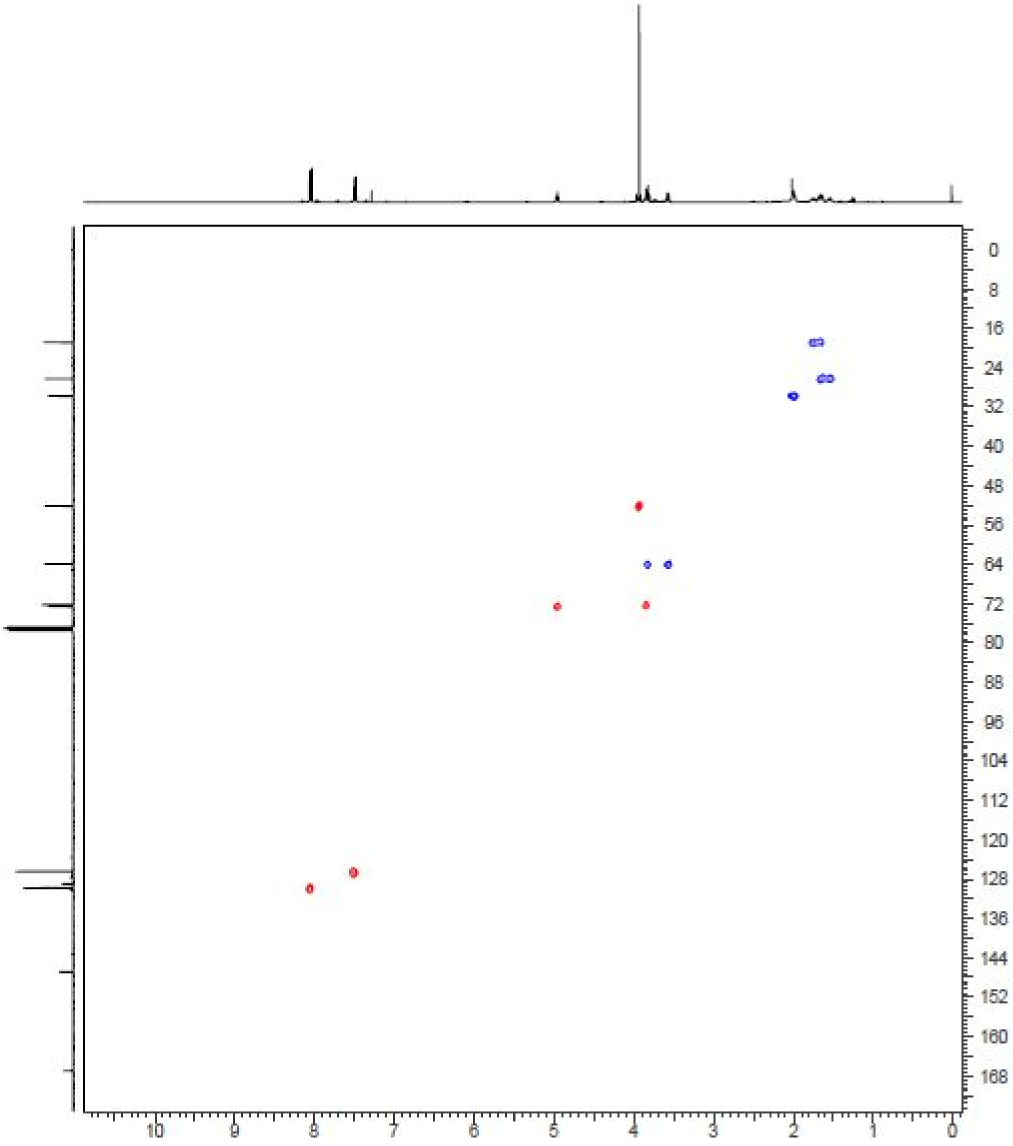

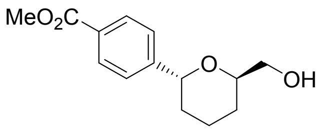

3l [^{1}H,^{13}C]-HMBC (400 MHz, 101 MHz, $CDCl_3$):

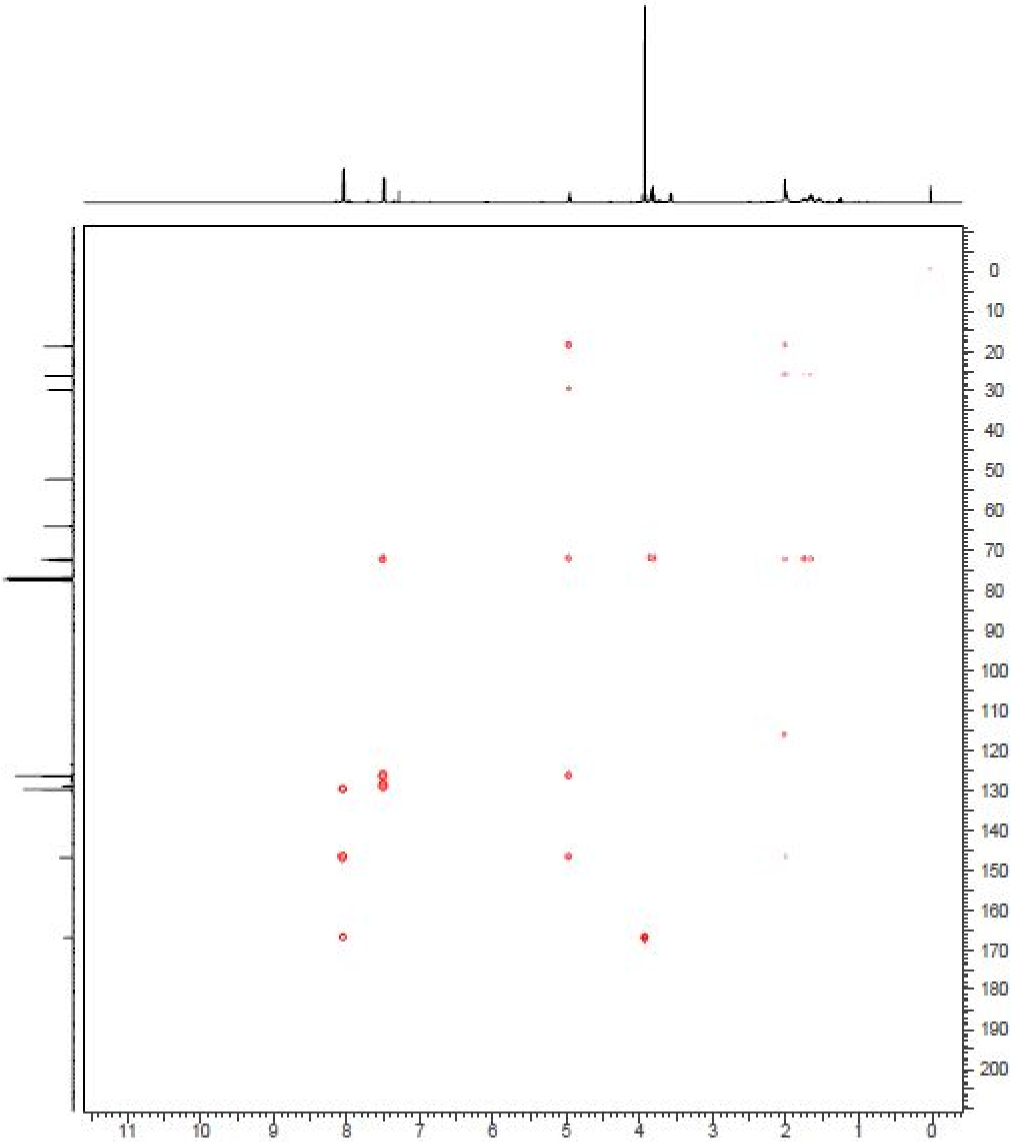

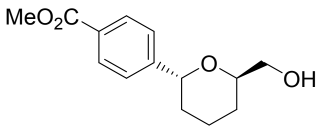

3l [^{1}H,^{1}H]-ROESY (400 MHz, $CDCl_3$):

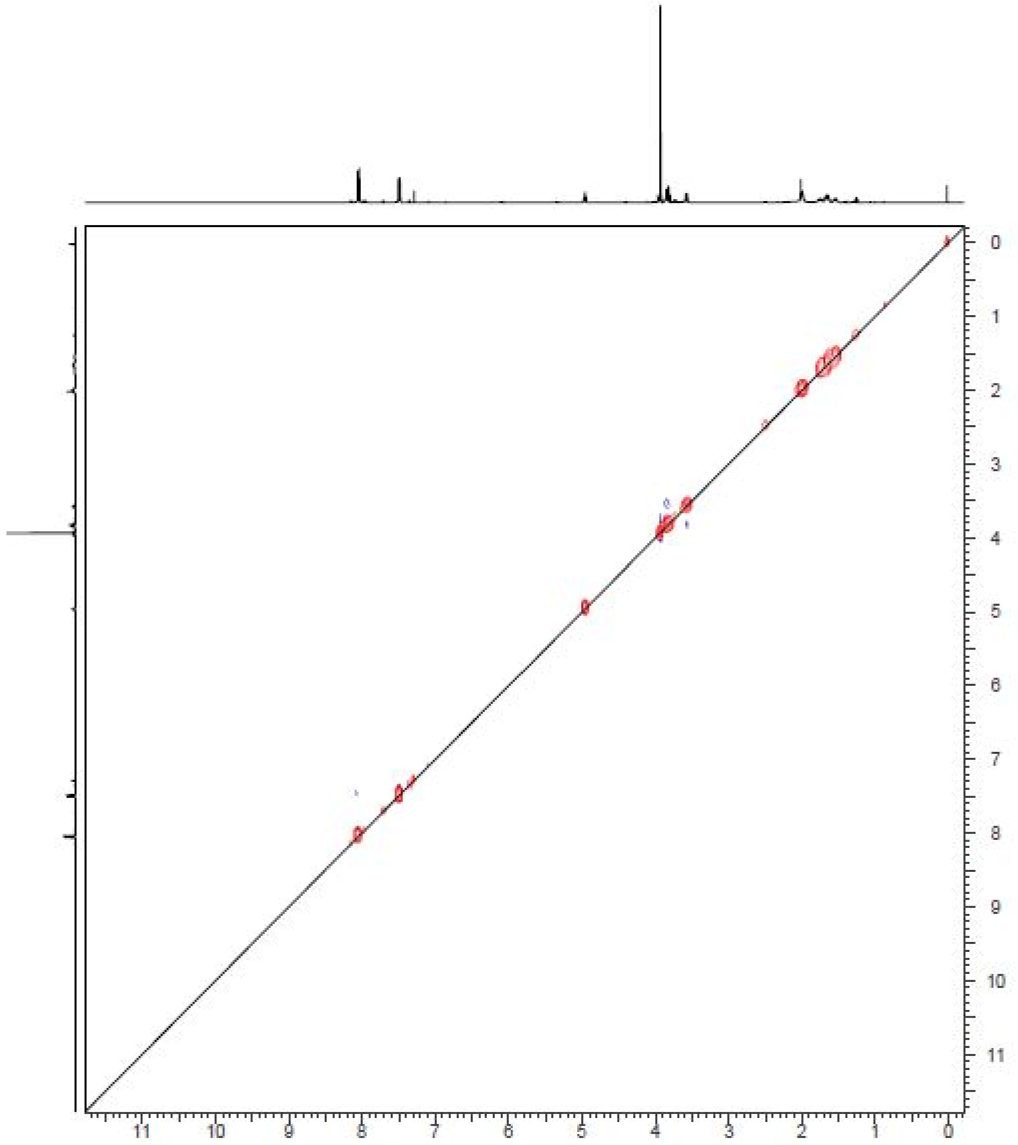

((2*R*,6*R*)-6-(3-nitrophenyl)tetrahydro-2*H*-pyran-2-yl)methanol, **3m**:

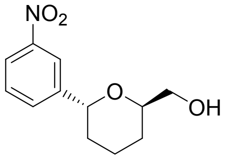

3m ^{1}H NMR (400 MHz, $CDCl_3$):

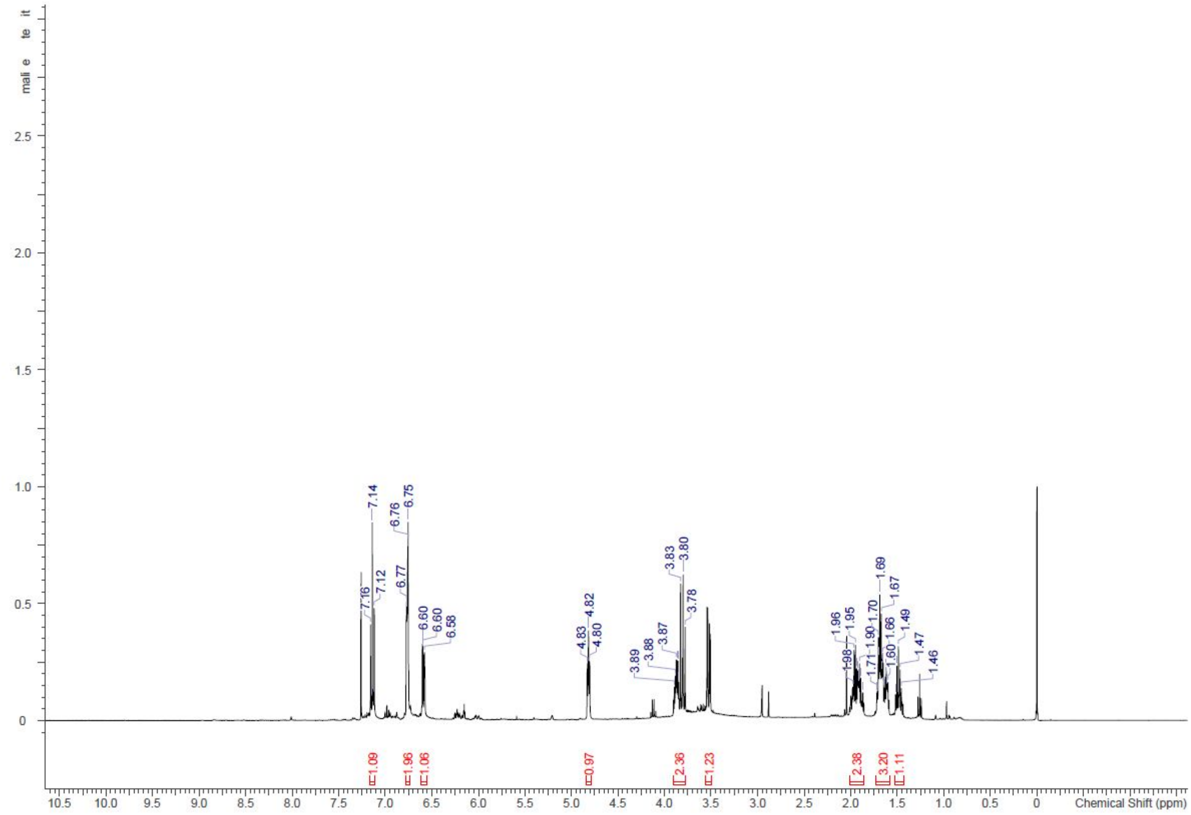

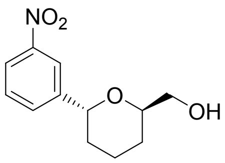

3m ^{13}C NMR (101 MHz, $CDCl_3$):

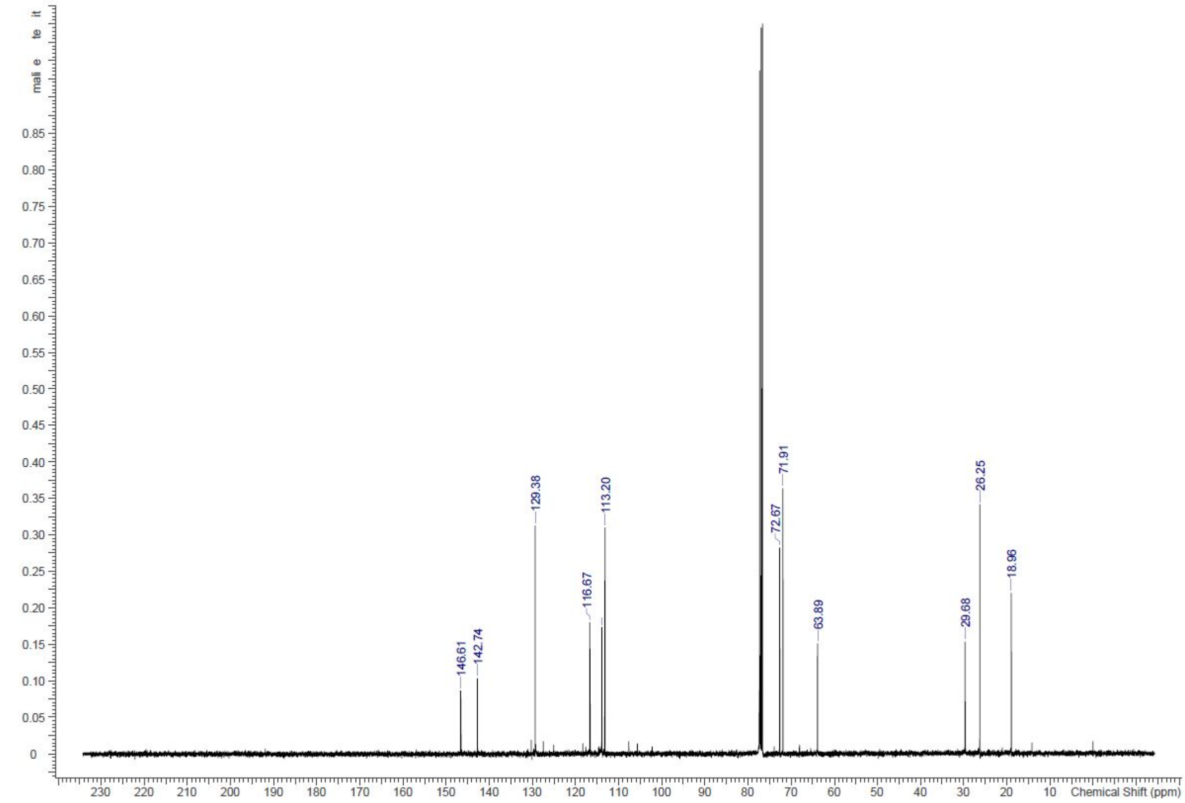

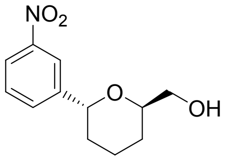

3m [1H,1H]-COSY (400 MHz, $CDCl_3$):

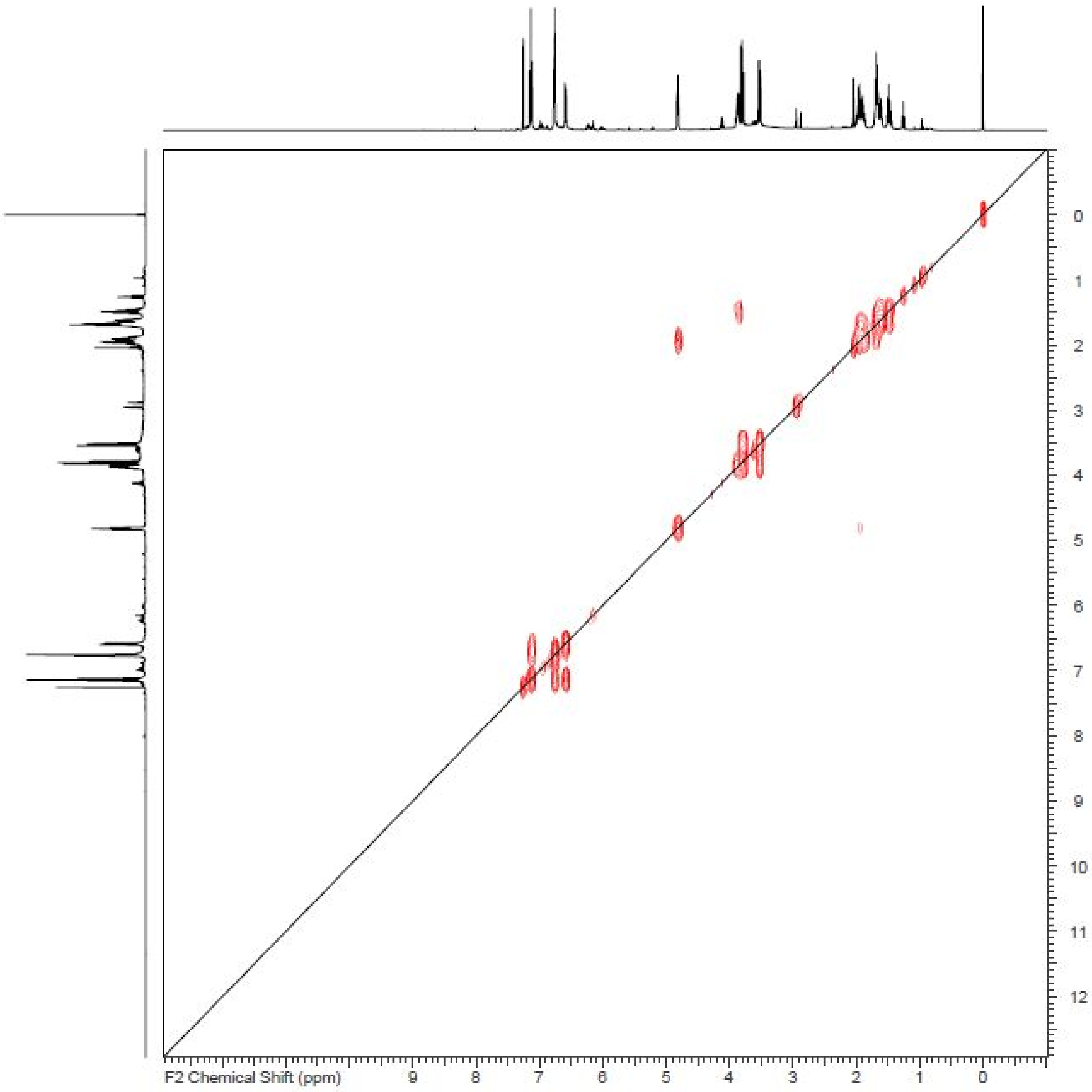

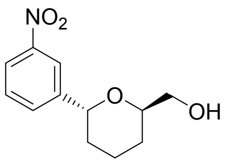

3m [^{1}H,^{13}C]-HSQC (400 MHz, 101 MHz, $CDCl_3$):

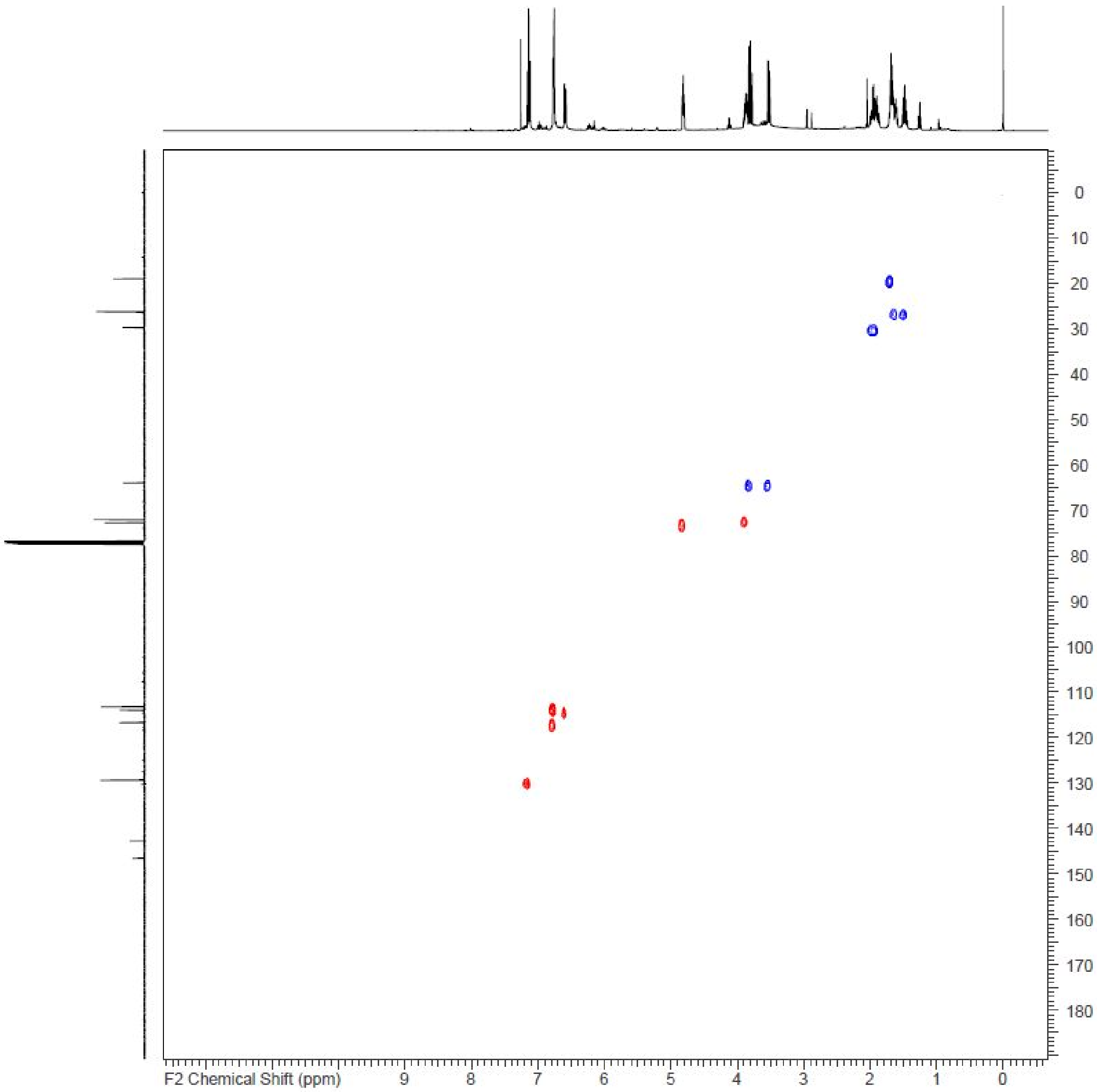

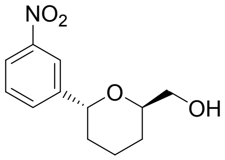

3m [1H,13C]-HMBC (400 MHz, 101 MHz, $CDCl_3$):

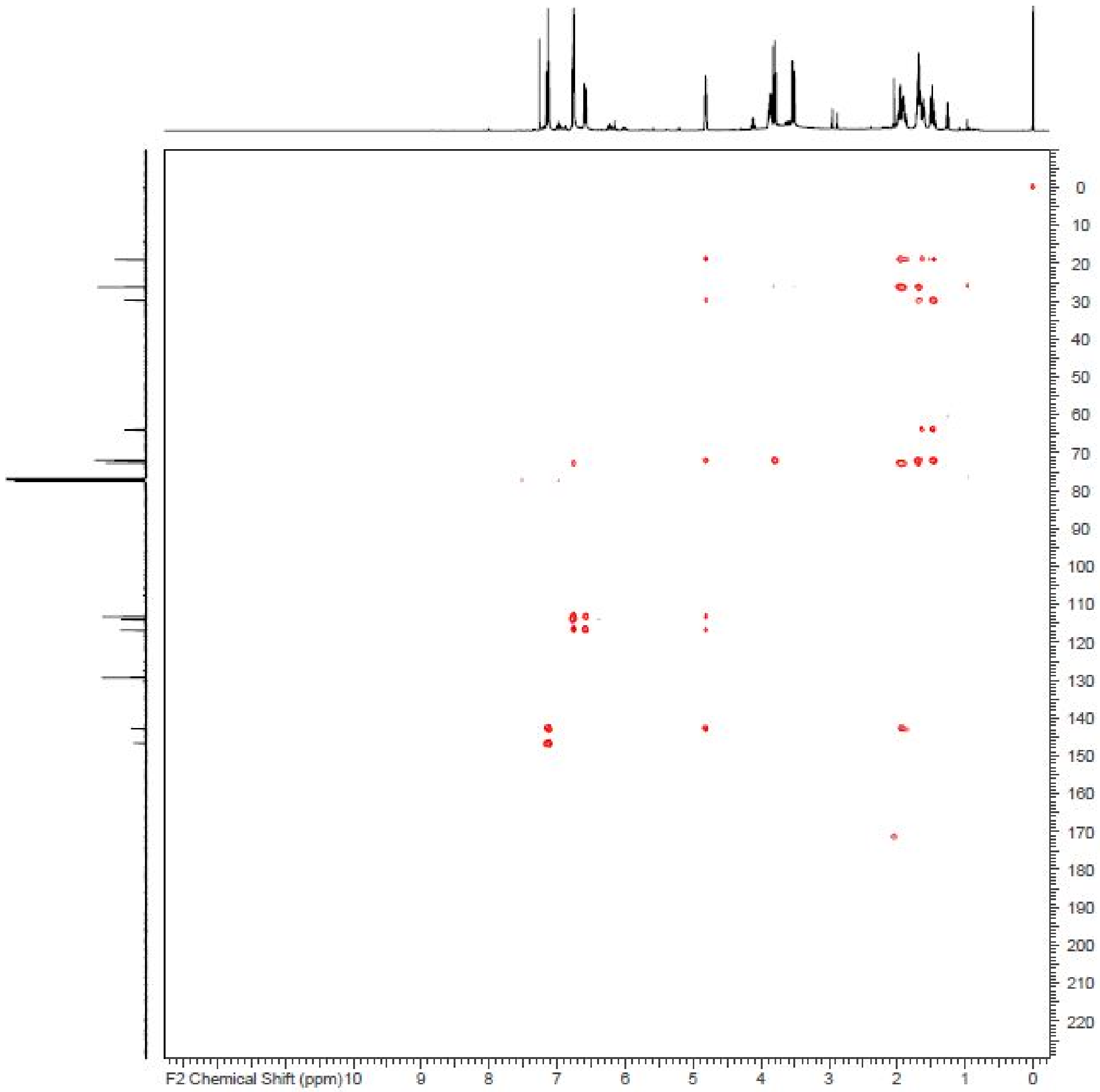

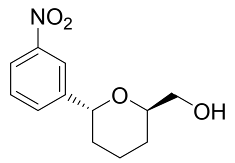

3m [1H,1H]-ROESY (400 MHz, $CDCl_3$):

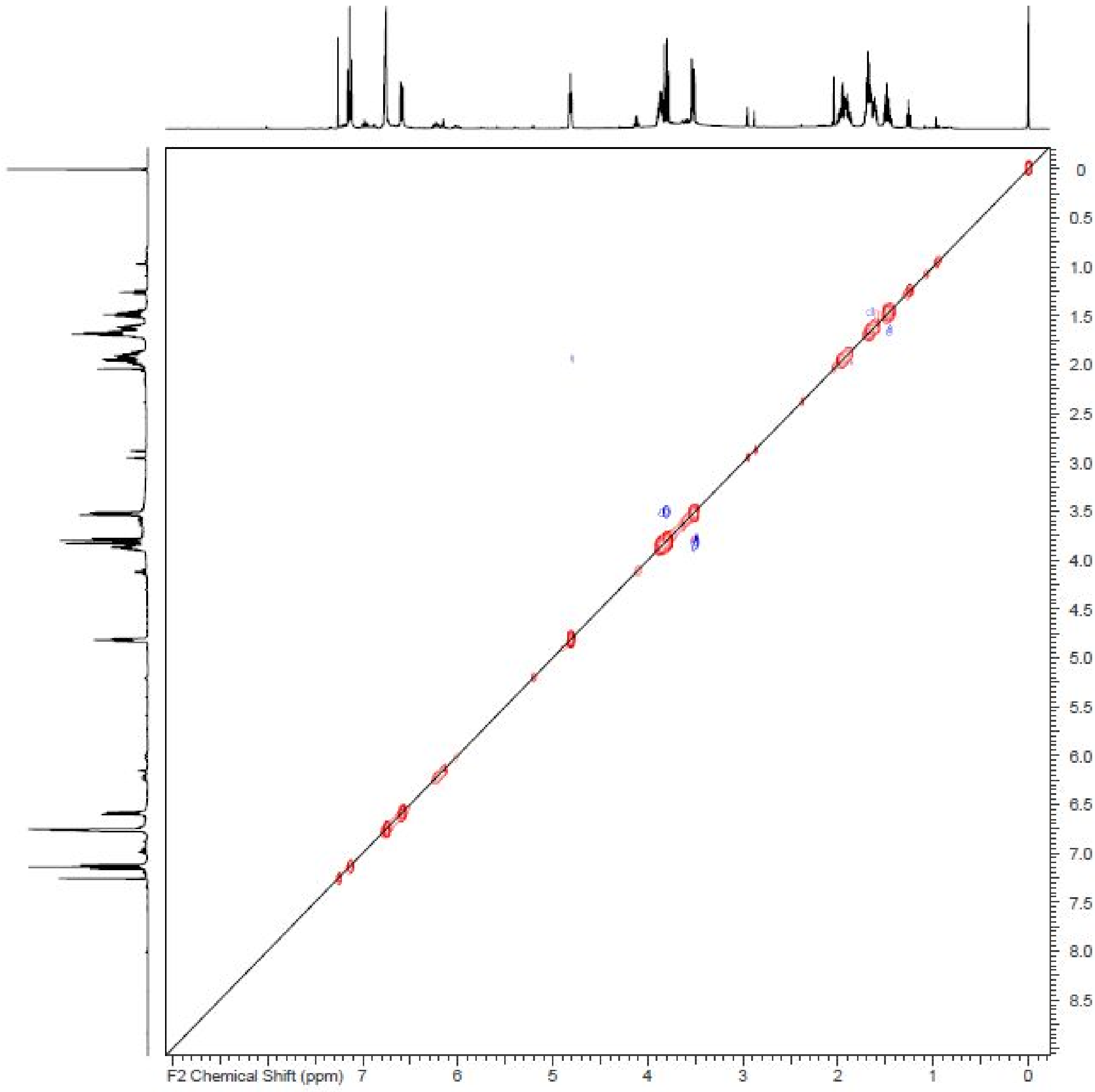

((2*R*,6*R*)-6-(4-bromophenyl)tetrahydro-2*H*-pyran-2-yl)methanol, **3n**

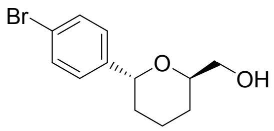

3n ^{1}H NMR (400 MHz, $CDCl_3$):

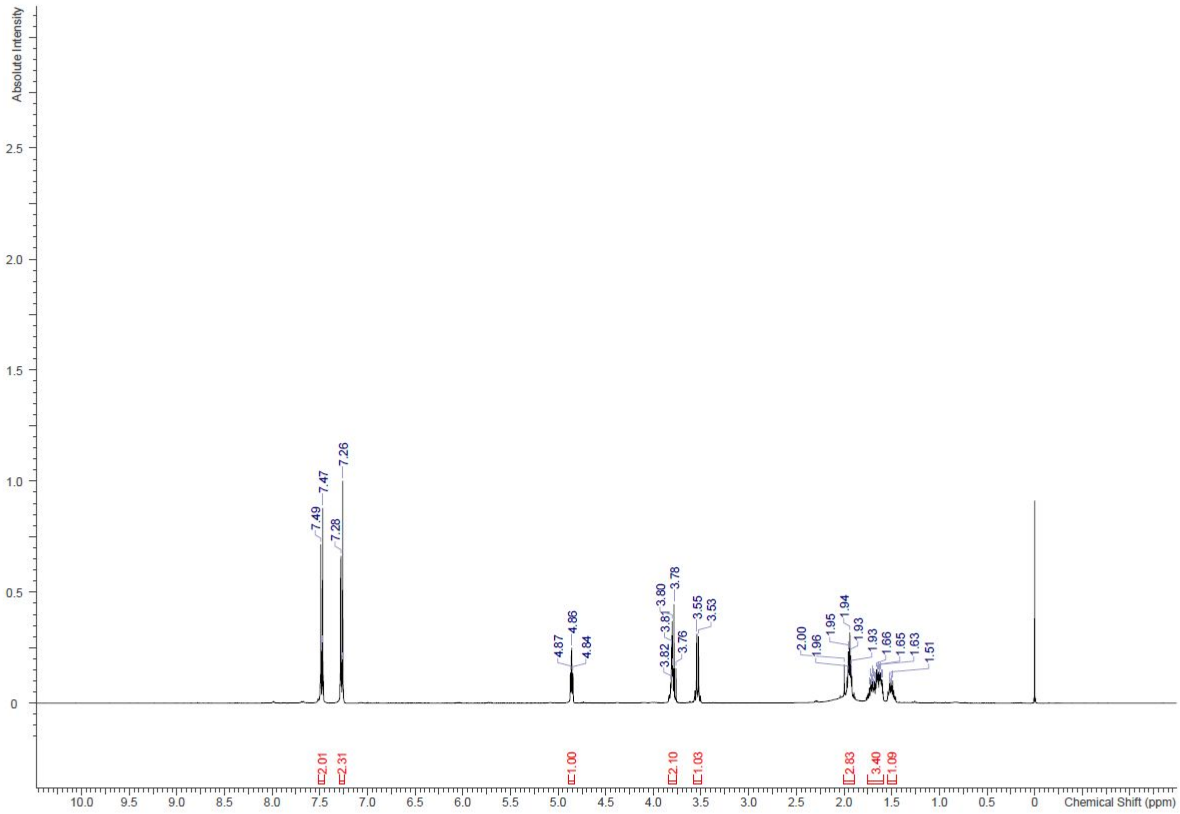

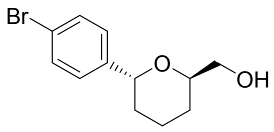

3n ^{13}C NMR (101 MHz, $CDCl_3$):

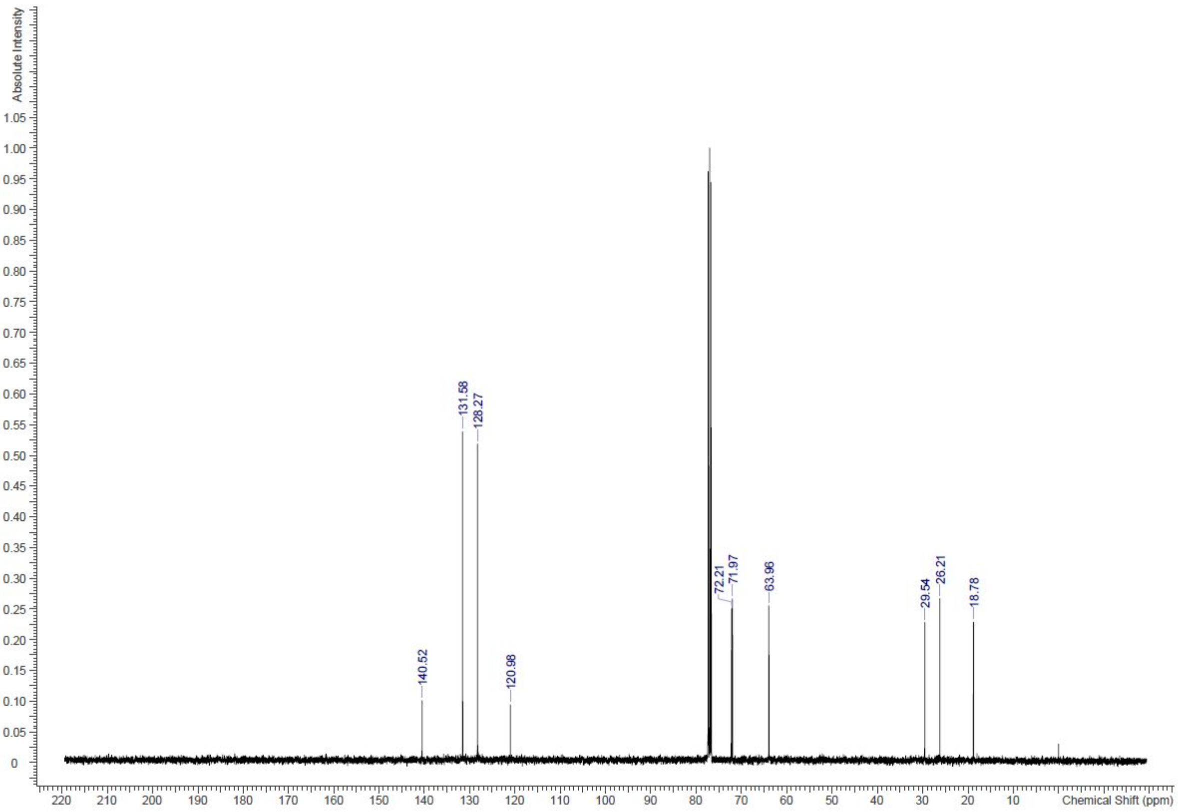

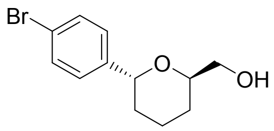

3n [^{1}H,^{1}H]-COSY (400 MHz, $CDCl_3$):

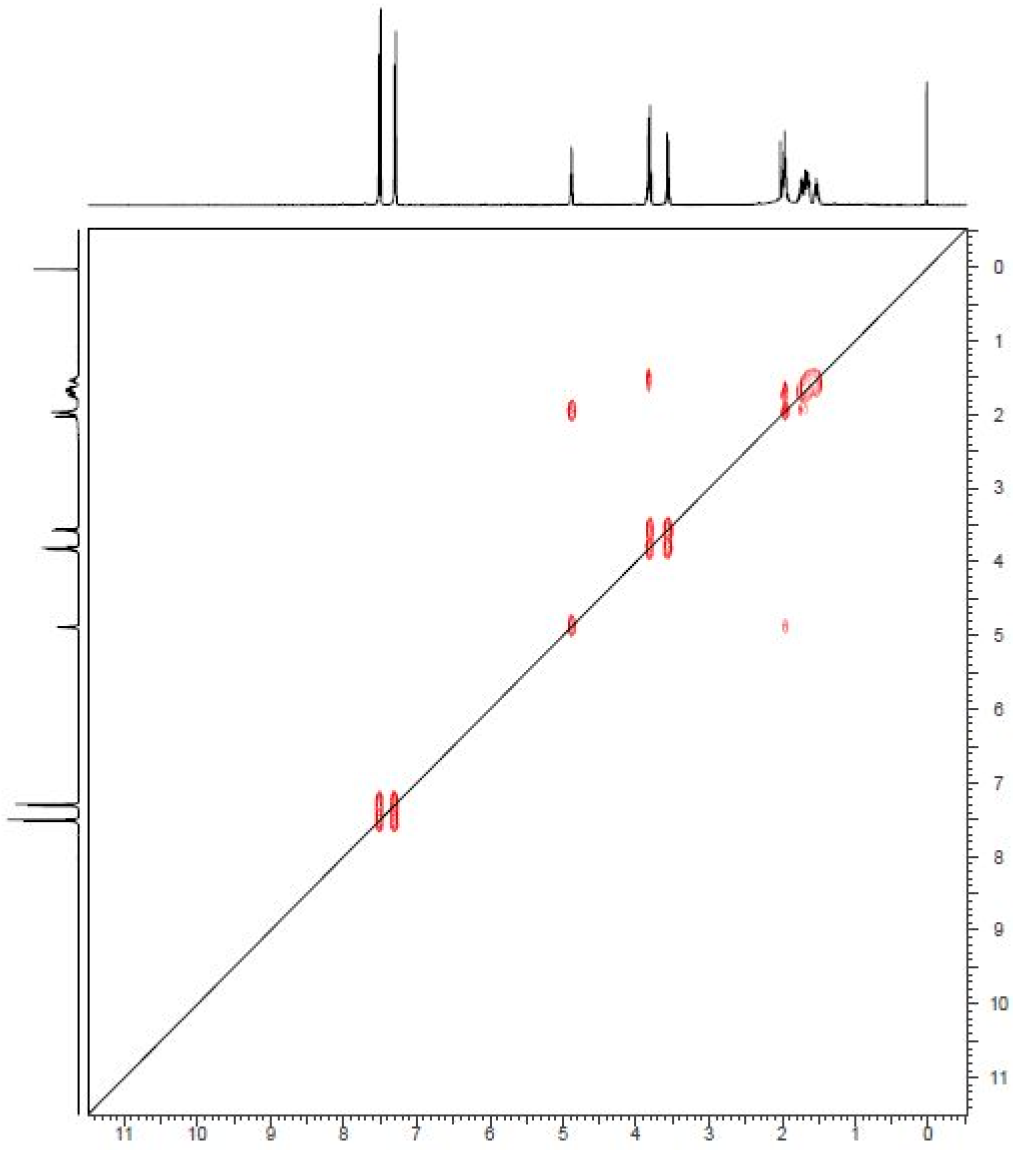

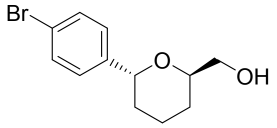

3n [^{1}H,^{13}C]-HSQC (400 MHz, 101 MHz, $CDCl_3$):

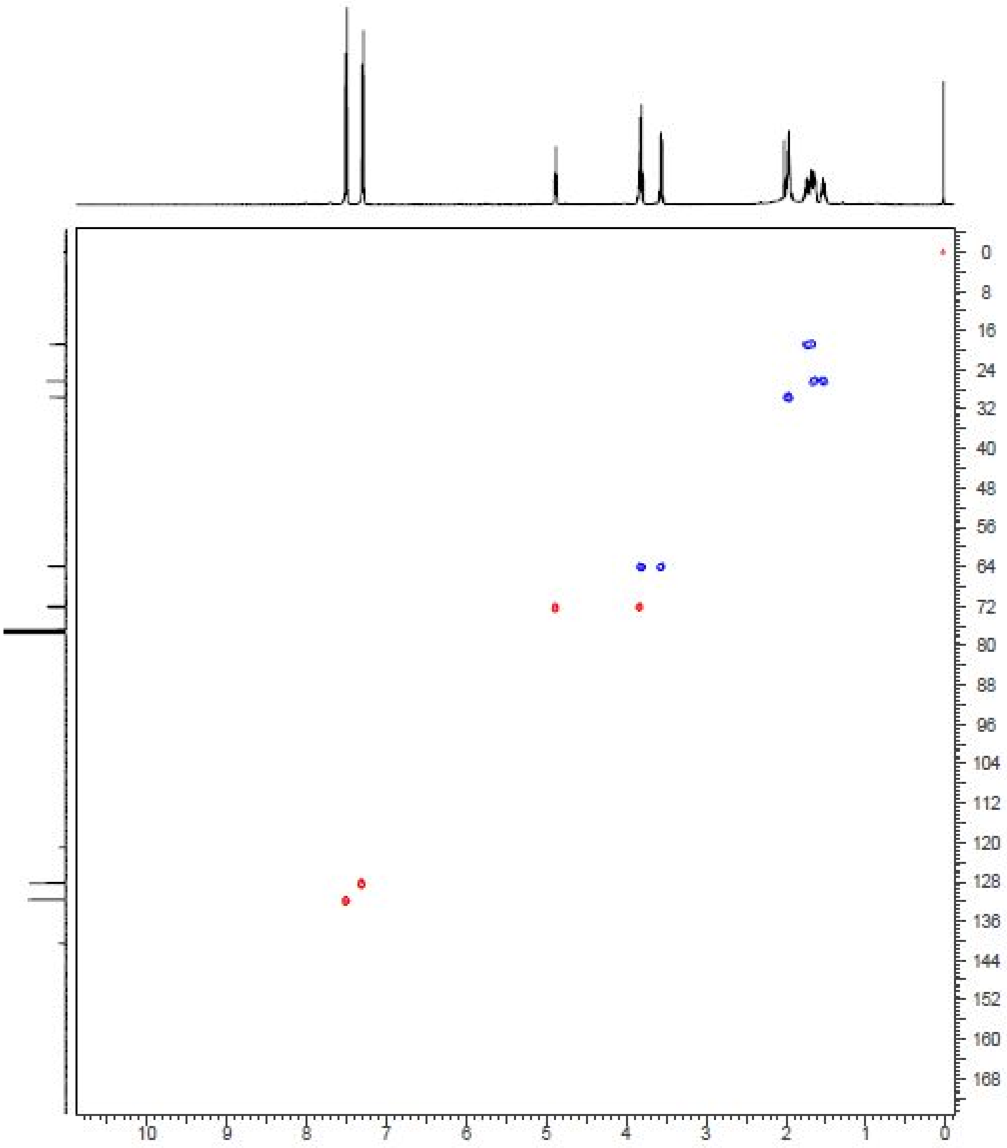

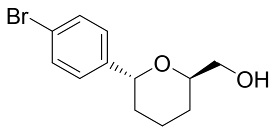

3n [^{1}H,^{13}C]-HMBC (400 MHz, 101 MHz, $CDCl_3$):

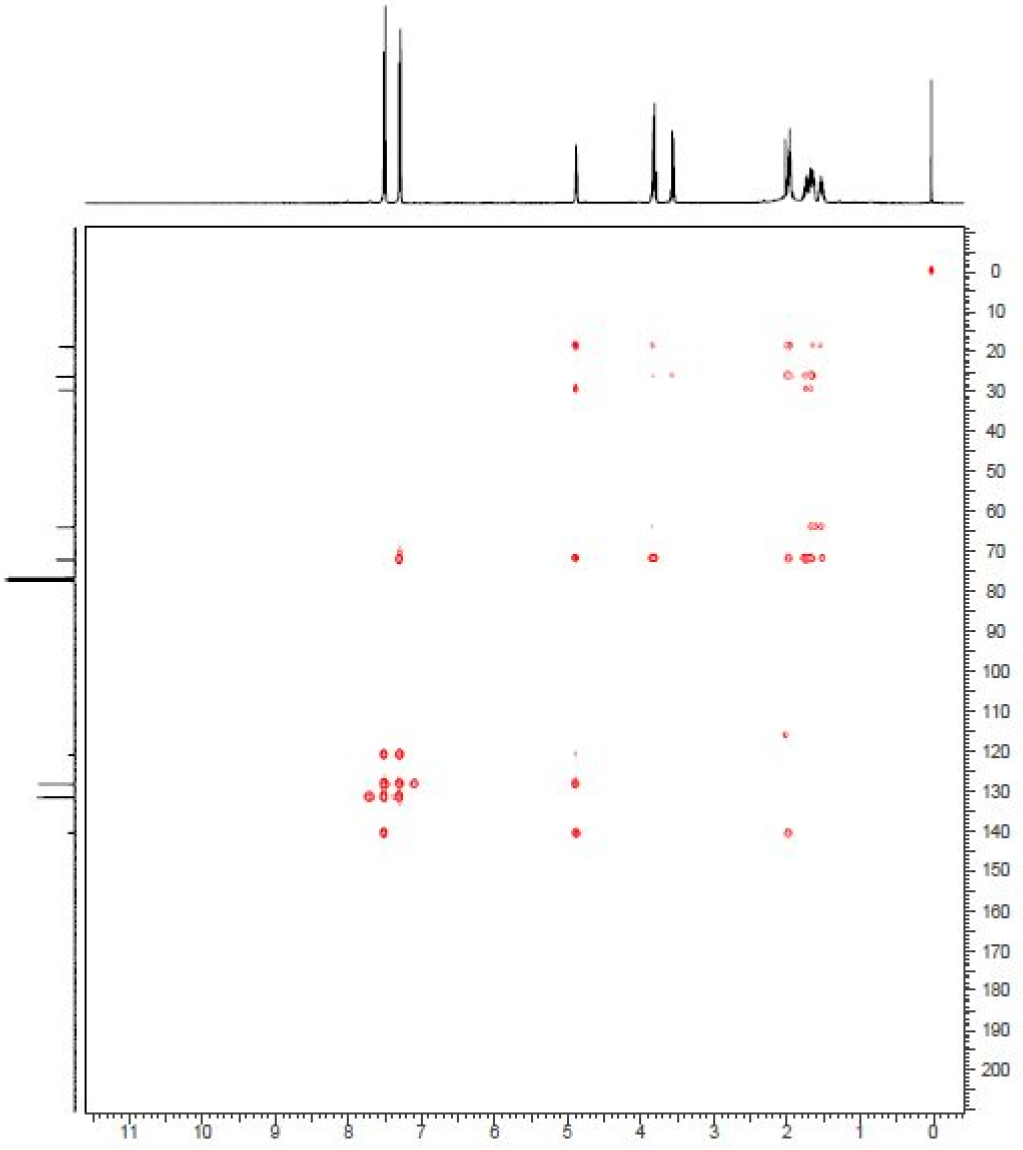

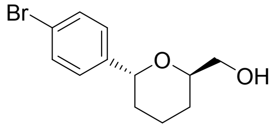

3n [^{1}H,^{1}H]-ROESY (400 MHz, $CDCl_3$):

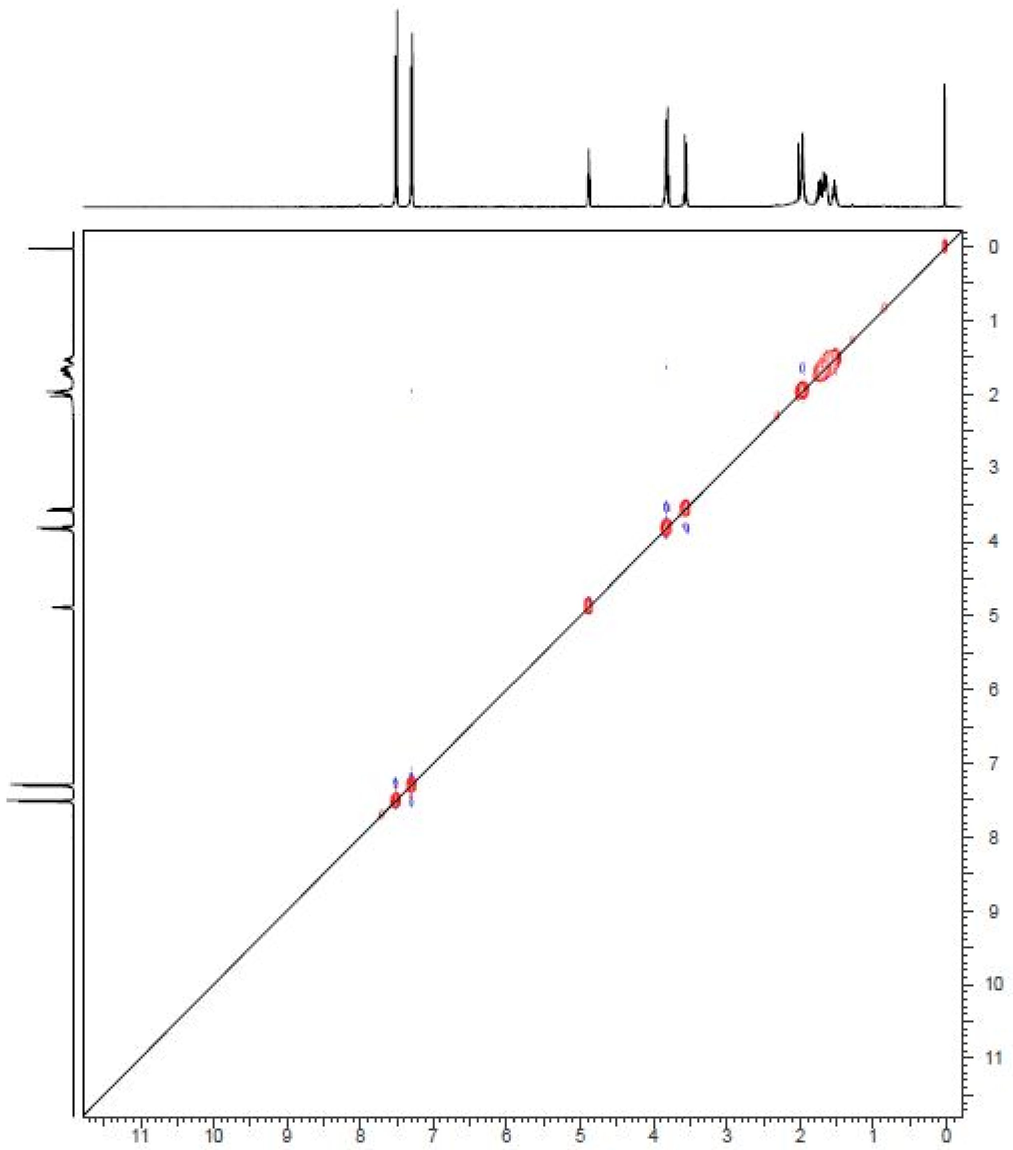

((2*R*,6*R*)-6-(furan-2-yl)tetrahydro-2*H*-pyran-2-yl)methanol, **3o**

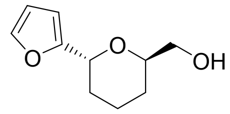

3o ^{1}H NMR (400 MHz, $CDCl_3$):

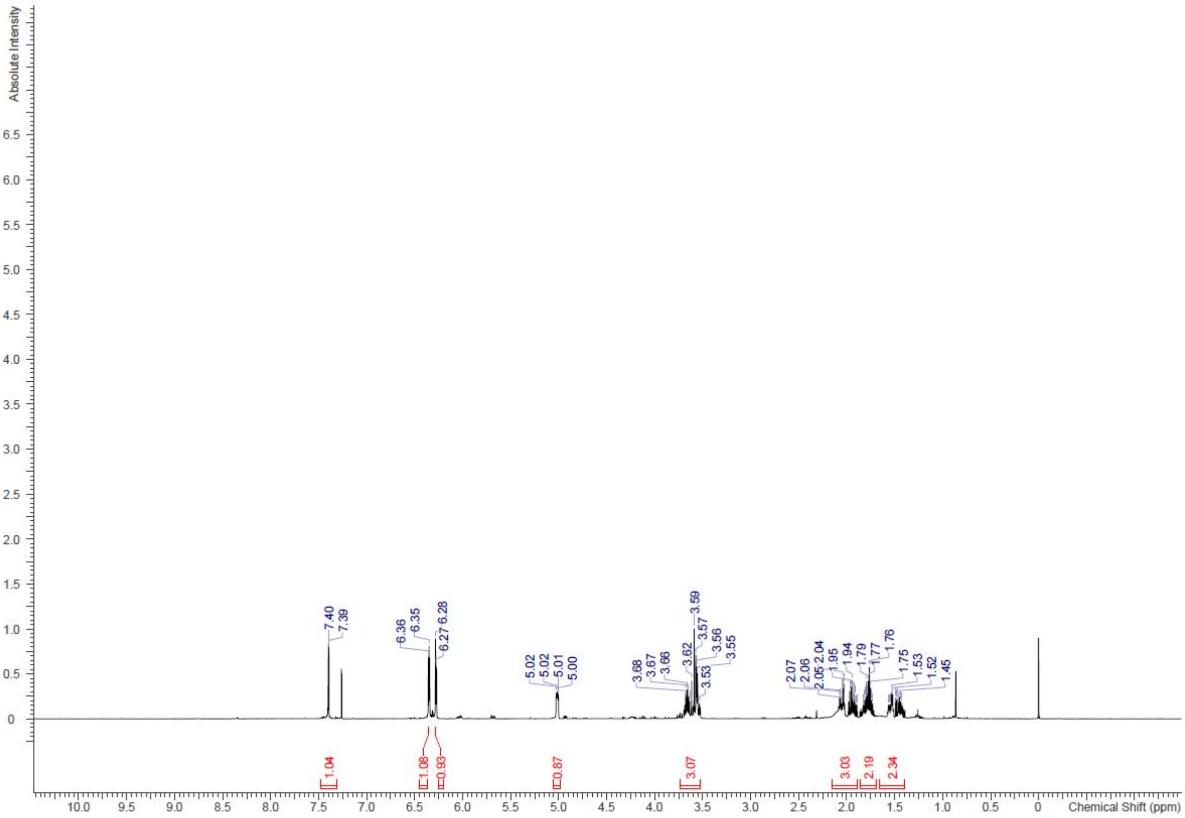

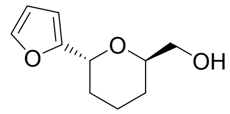

3o ^{13}C NMR (101 MHz, $CDCl_3$):

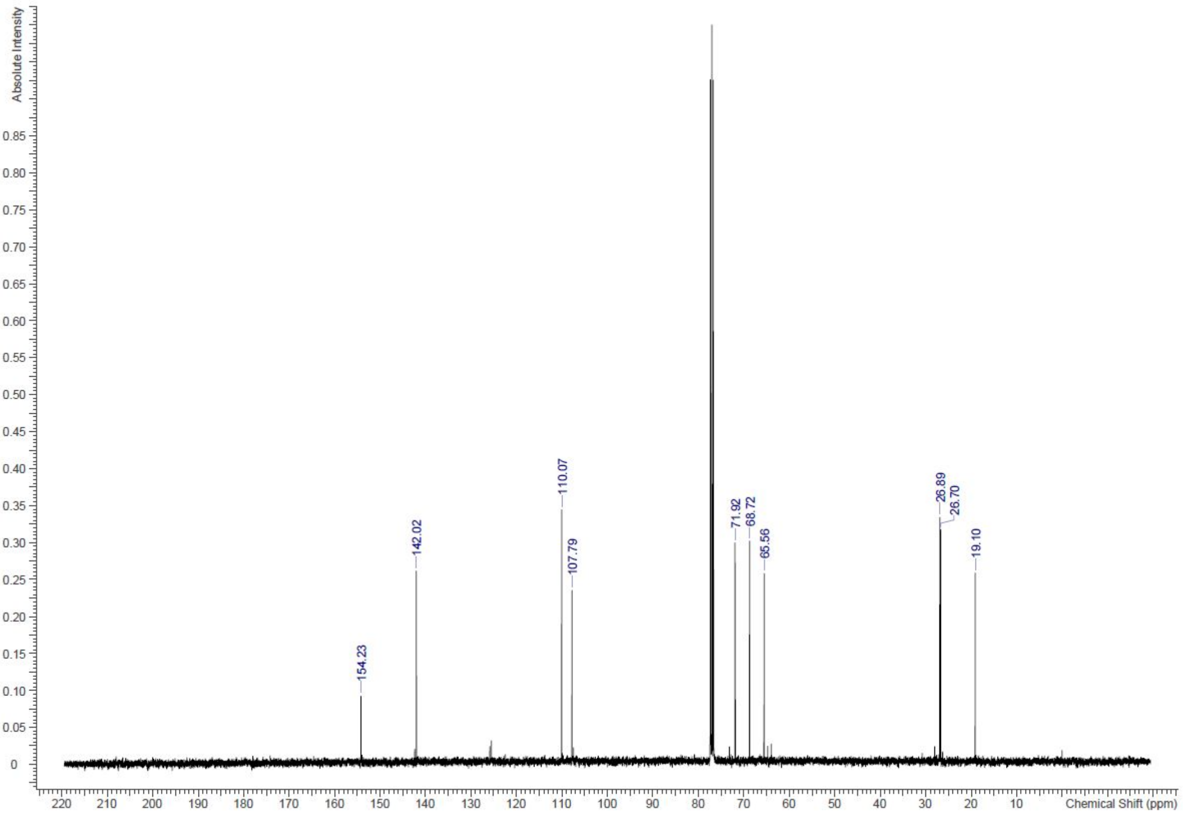

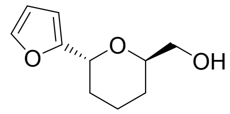

3o [1H,1H]-COSY (400 MHz, $CDCl_3$):

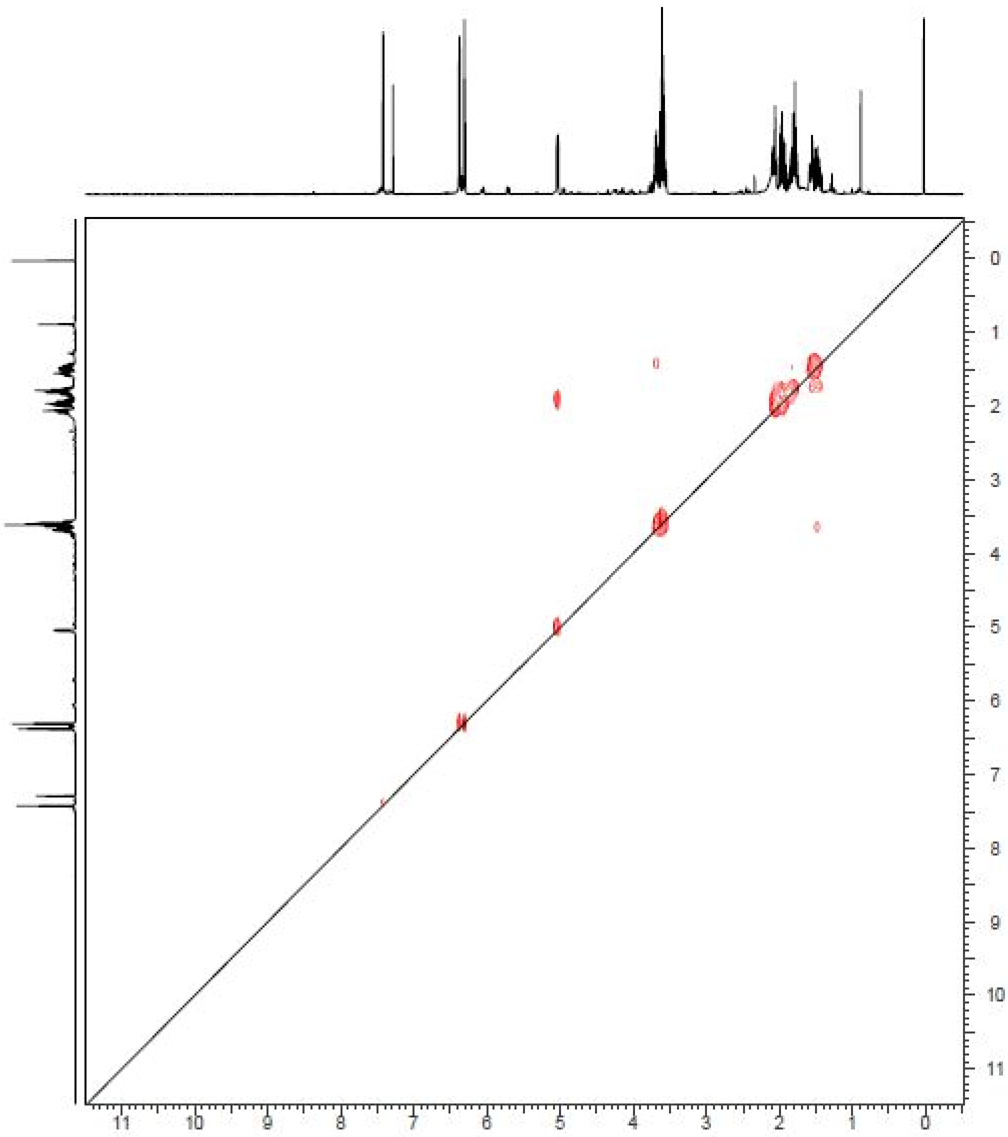

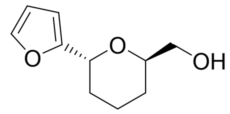

3o [^{1}H,^{13}C]-HSQC (400 MHz, 101 MHz, $CDCl_3$):

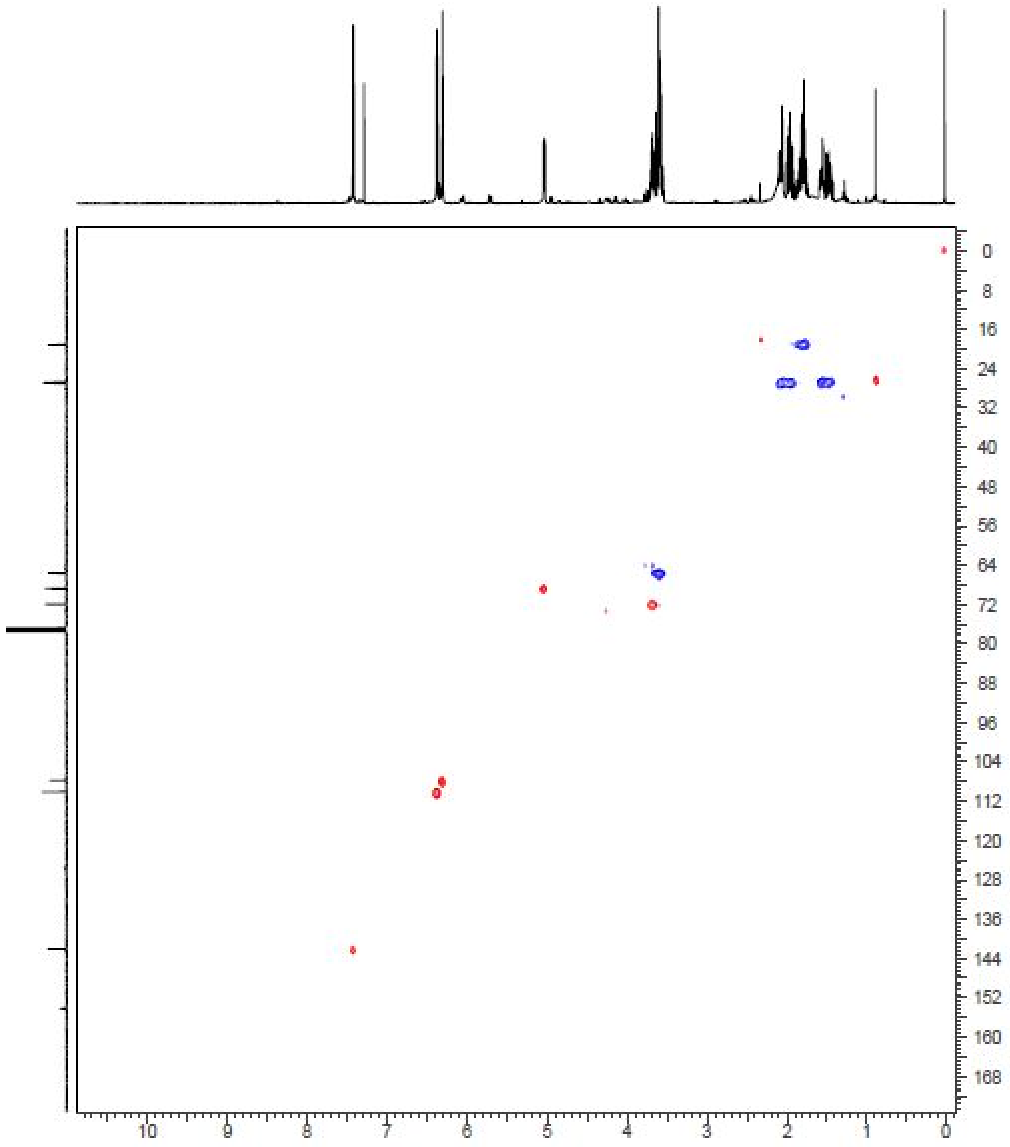

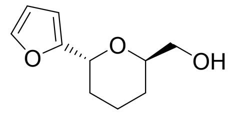

3o [^{1}H,^{13}C]-HMBC (400 MHz, 101 MHz, CDCl$_3$):

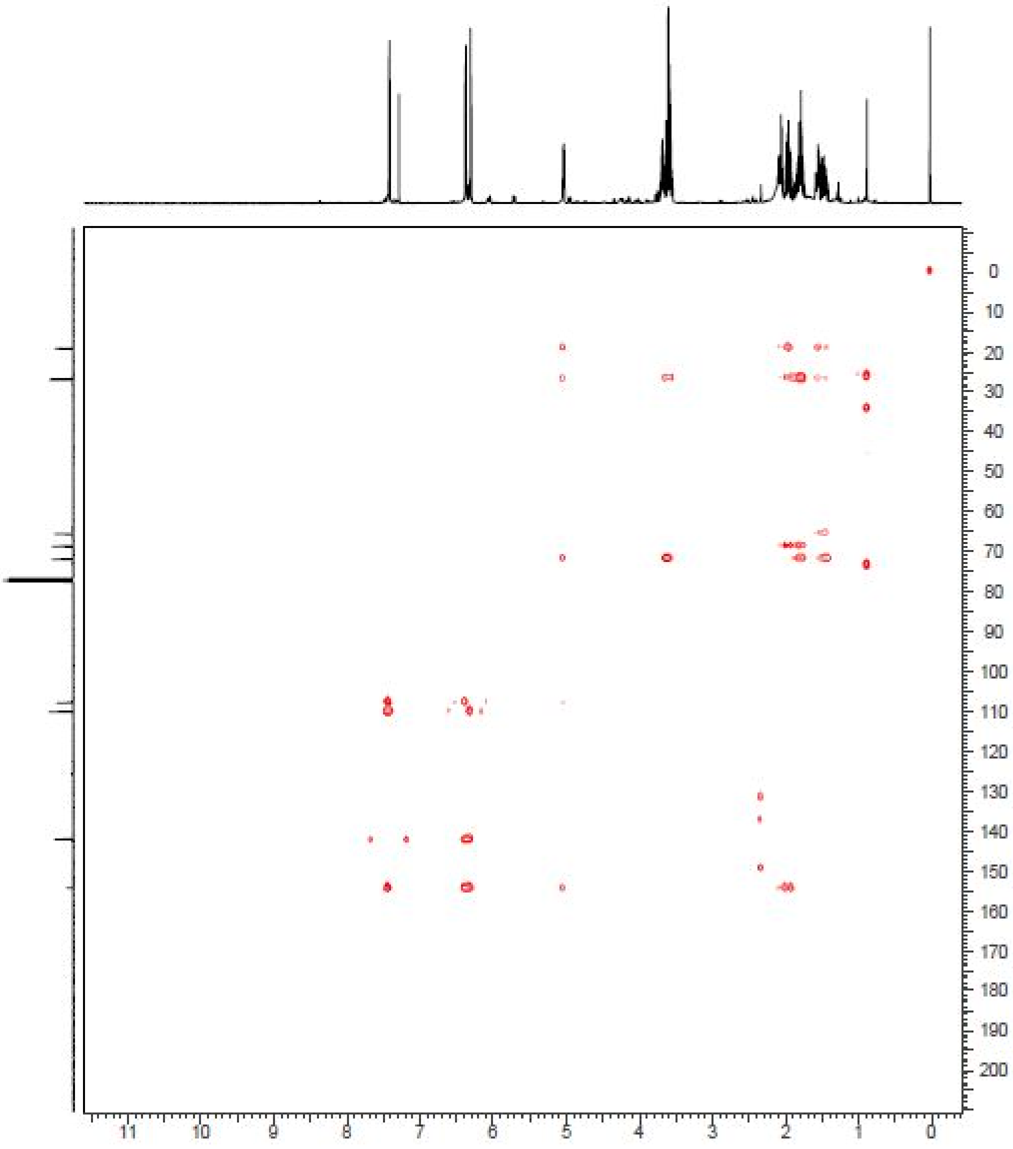

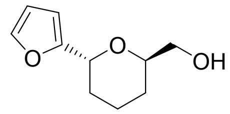

3o [^{1}H,^{1}H]-ROESY (400 MHz, $CDCl_3$):

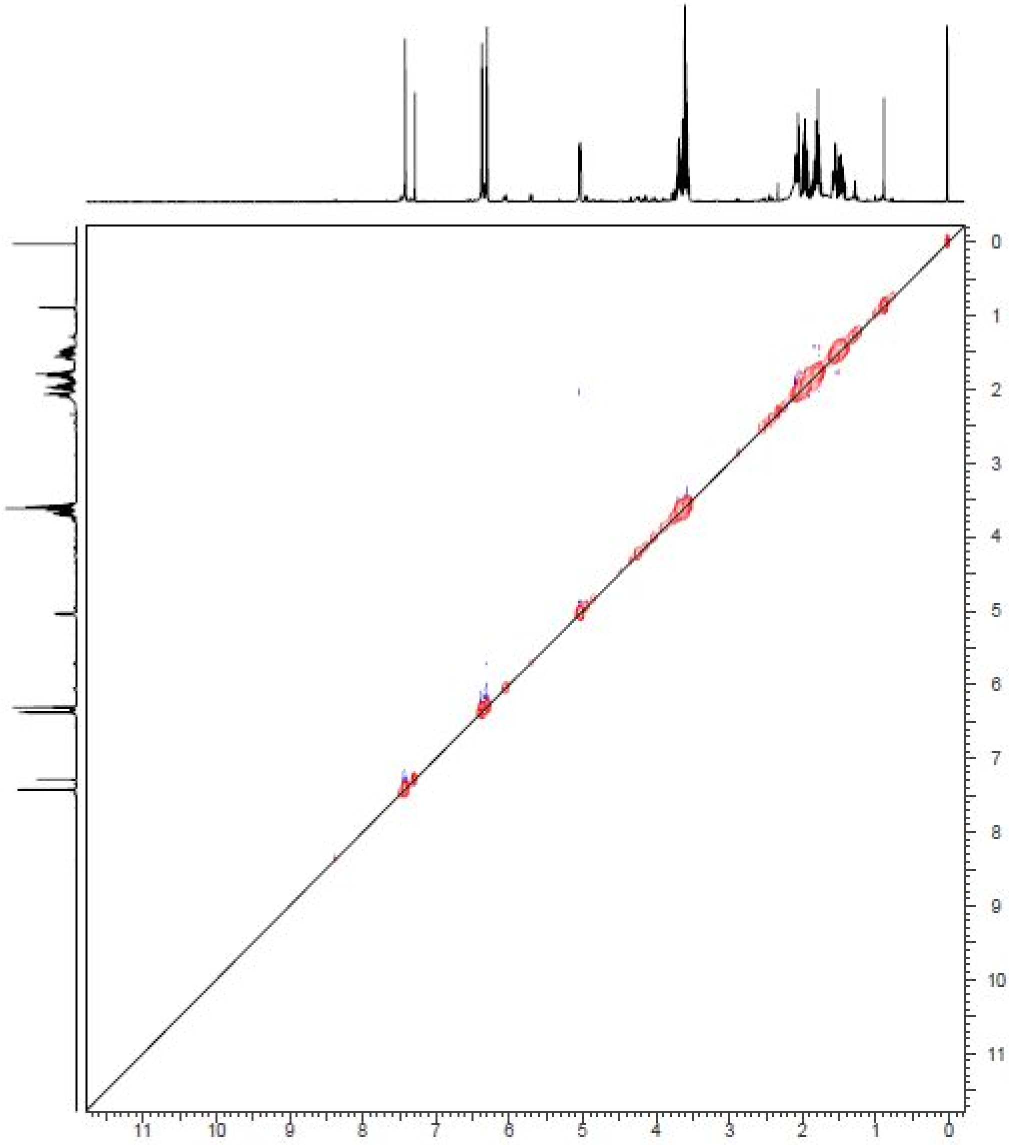

((2*R*,6*R*)-6-(furan-3-yl)tetrahydro-2*H*-pyran-2-yl)methanol, **3p**

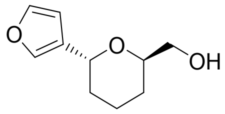

3p ^{1}H NMR (400 MHz, $CDCl_3$):

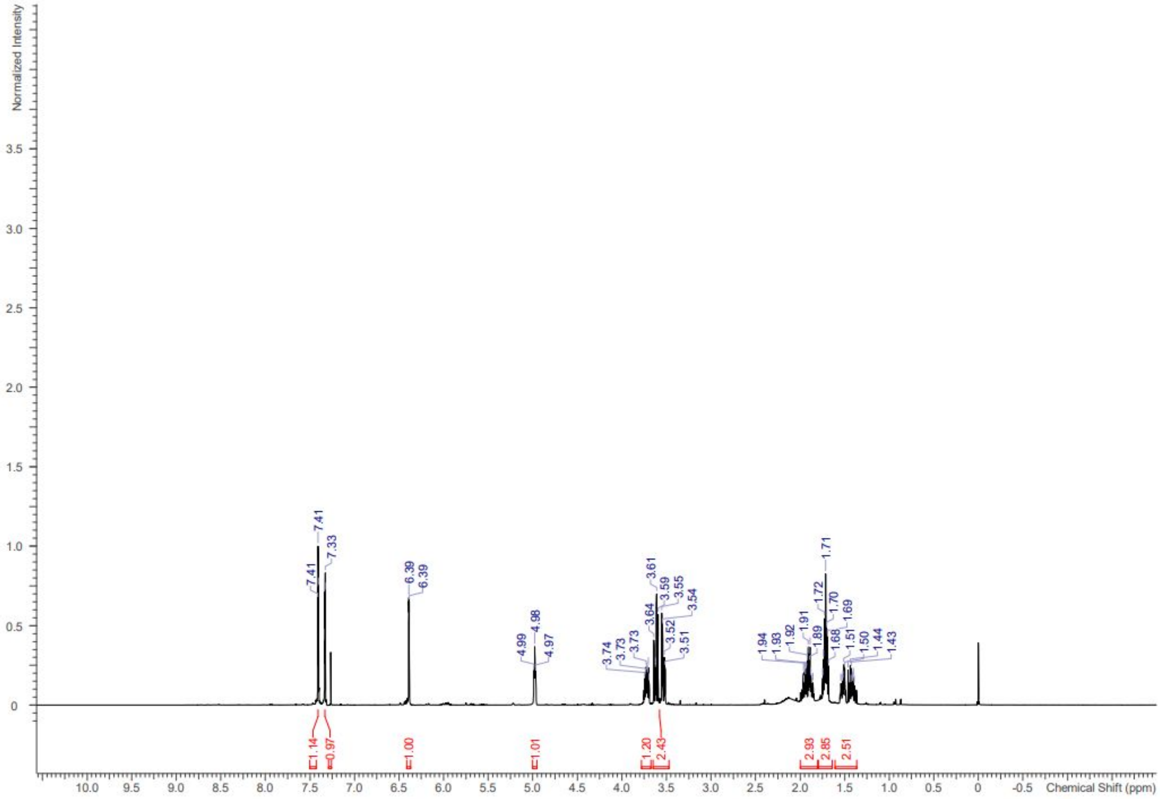

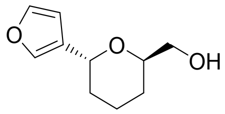

3p ^{13}C NMR (101 MHz, $CDCl_3$):

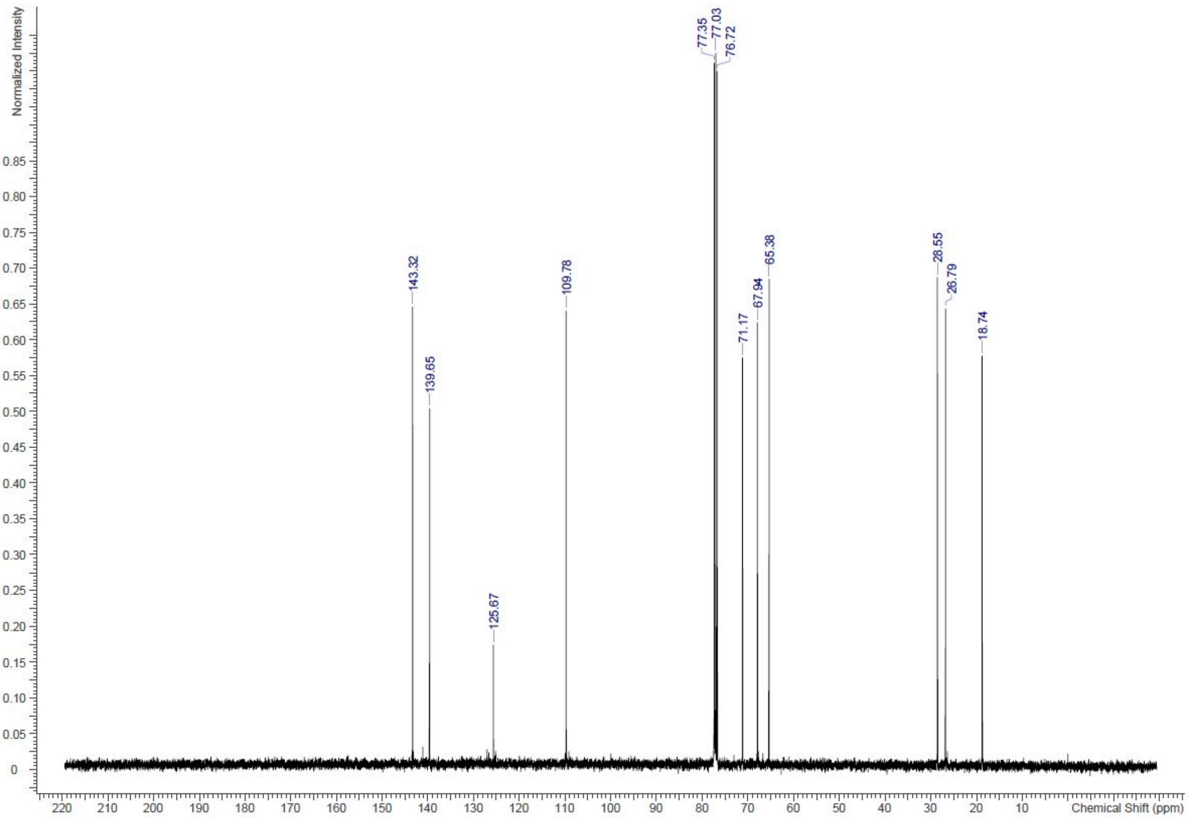

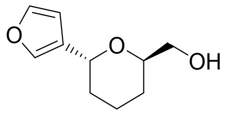

3p [1H,1H]-COSY (400 MHz, $CDCl_3$):

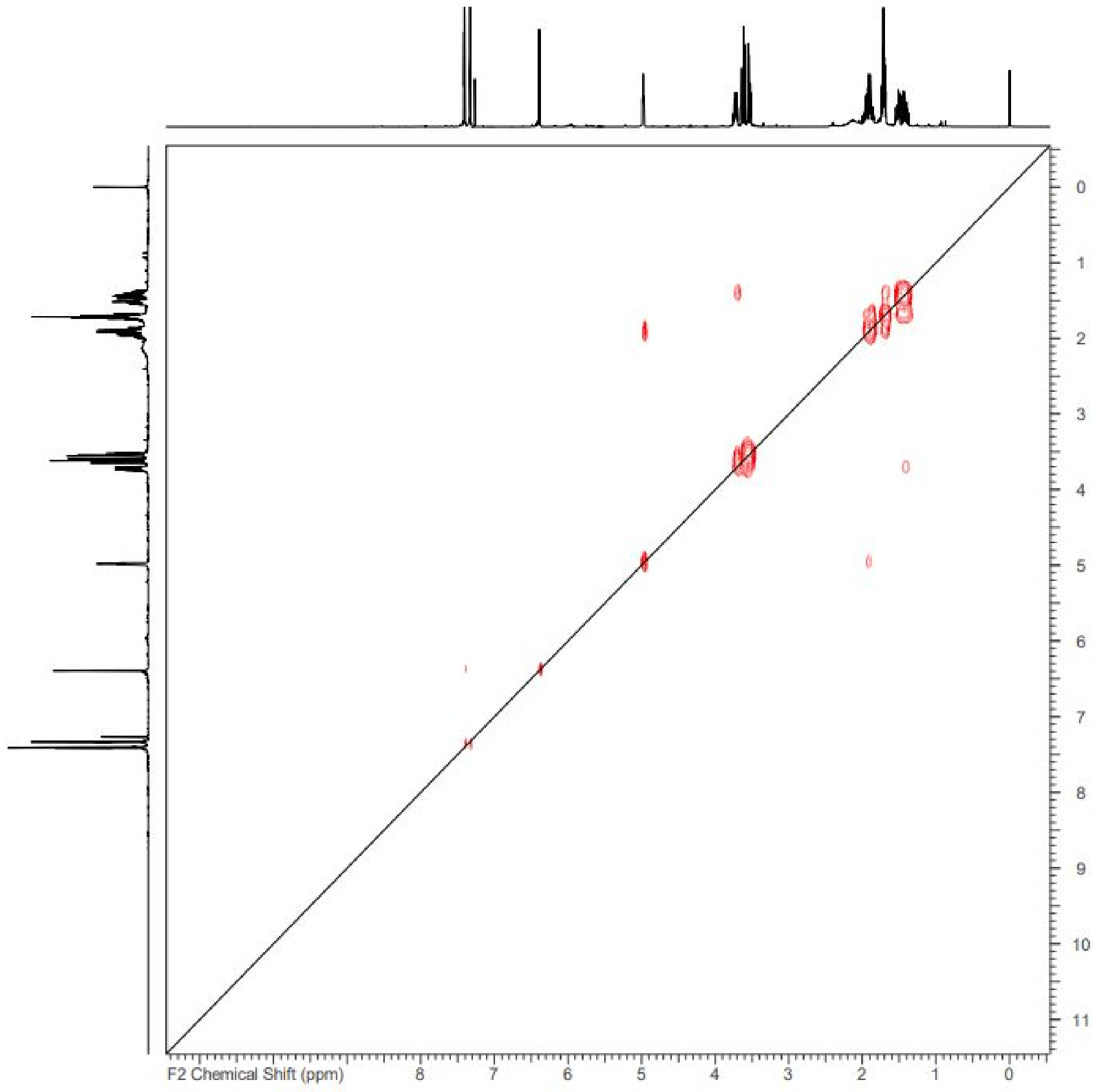

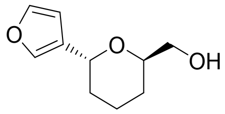

3p [^{1}H,^{13}C]-HSQC (400 MHz, 101 MHz, $CDCl_3$):

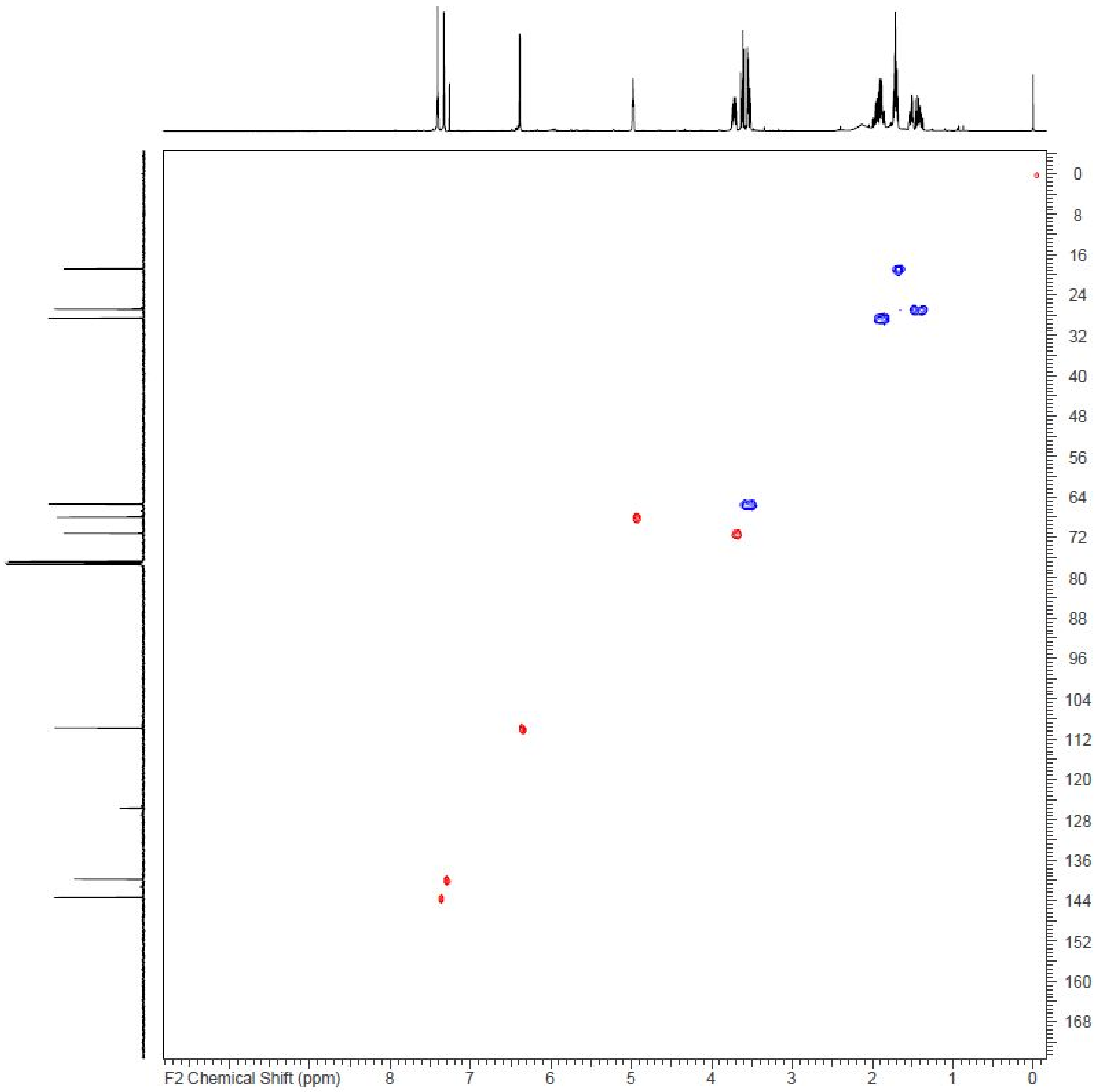

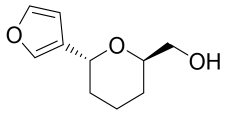

3p [^{1}H,^{13}C]-HMBC (400 MHz, 101 MHz, $CDCl_3$):

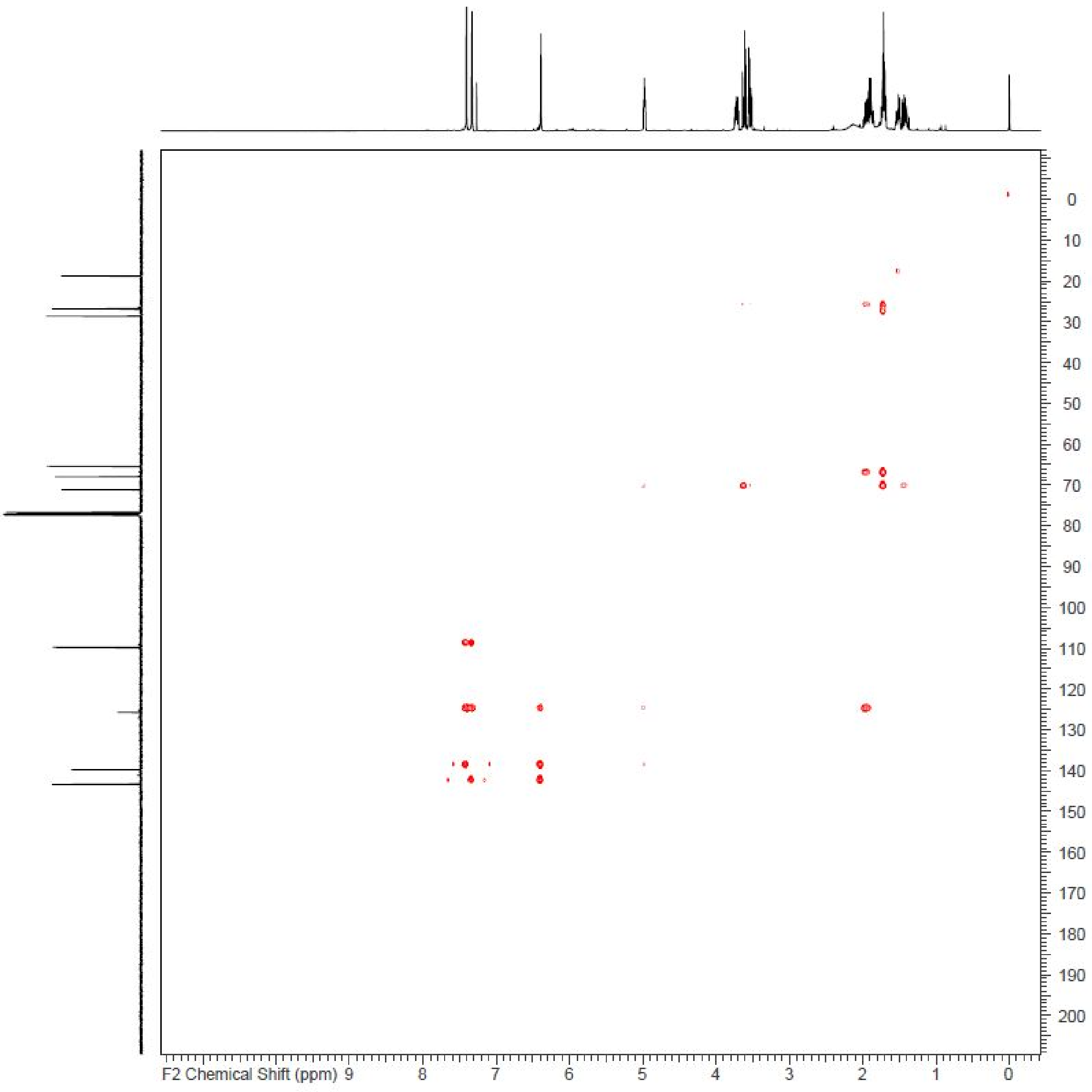

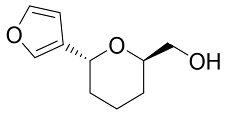

3p [^{1}H,^{1}H]-ROESY (400 MHz, $CDCl_3$):

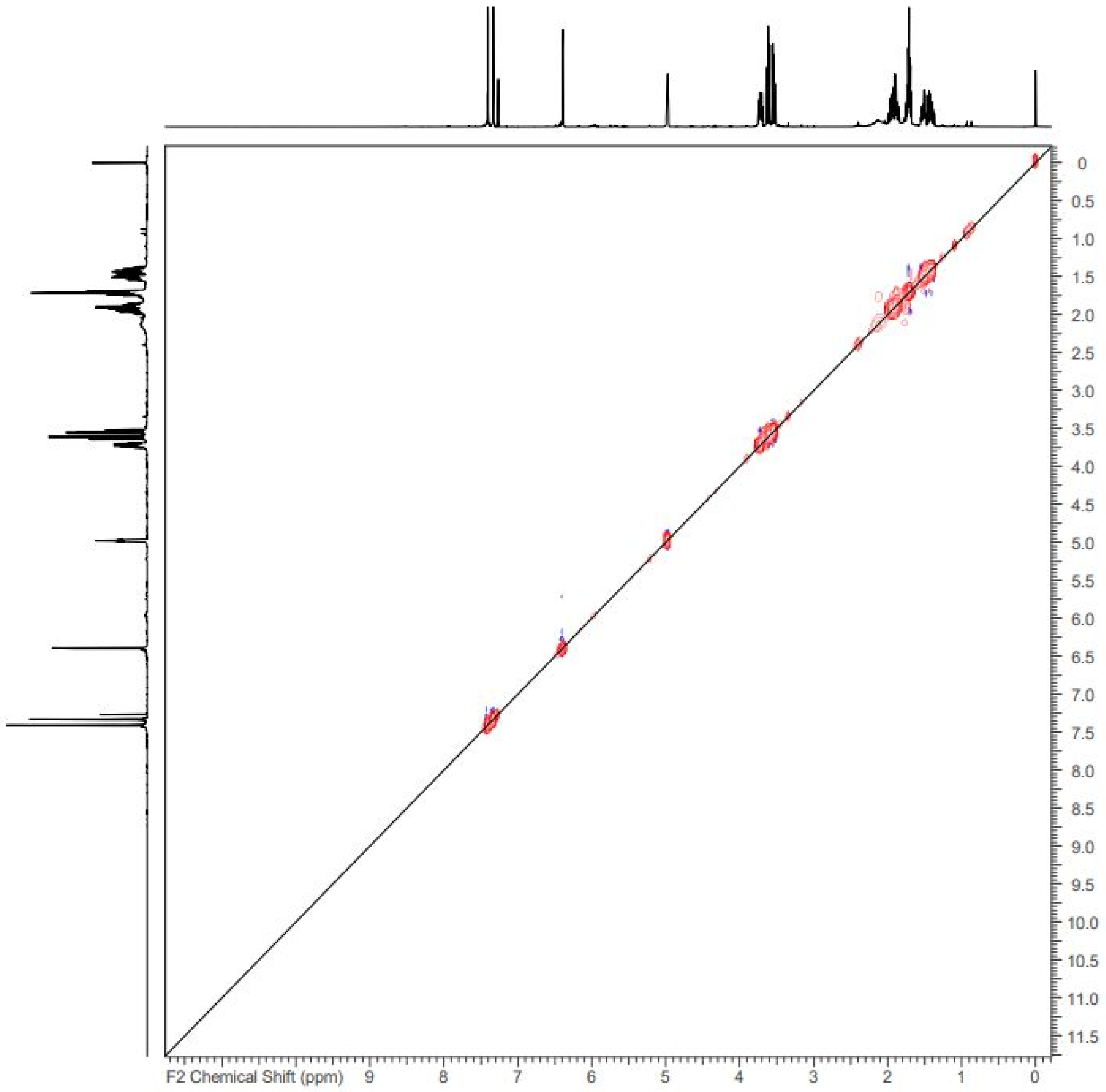

(2*S*,6*S*)-6-(4-methoxyphenyl)tetrahydro-2*H*-pyran-2-carbaldehyde, *ent*-**2c**

ent-**2c** ^{1}H NMR (400 MHz, $CDCl_3$):

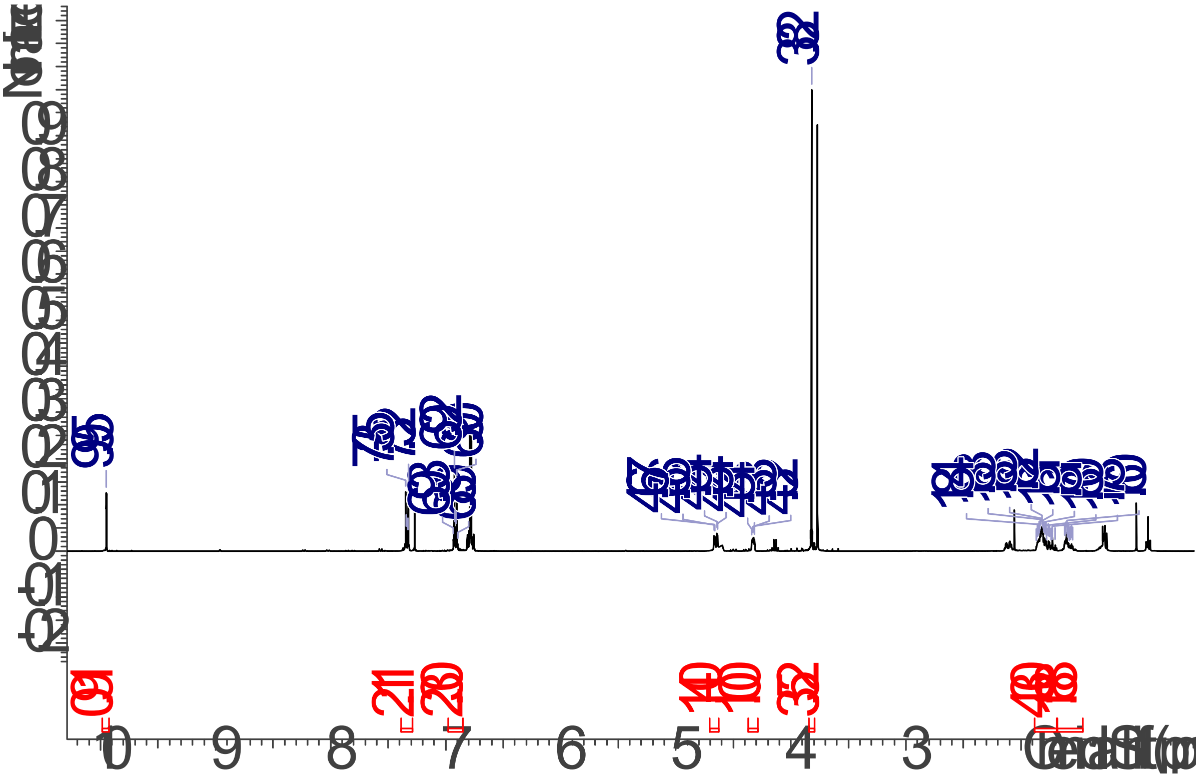

ent-**2c** ^{13}C NMR (101 MHz, $CDCl_3$):

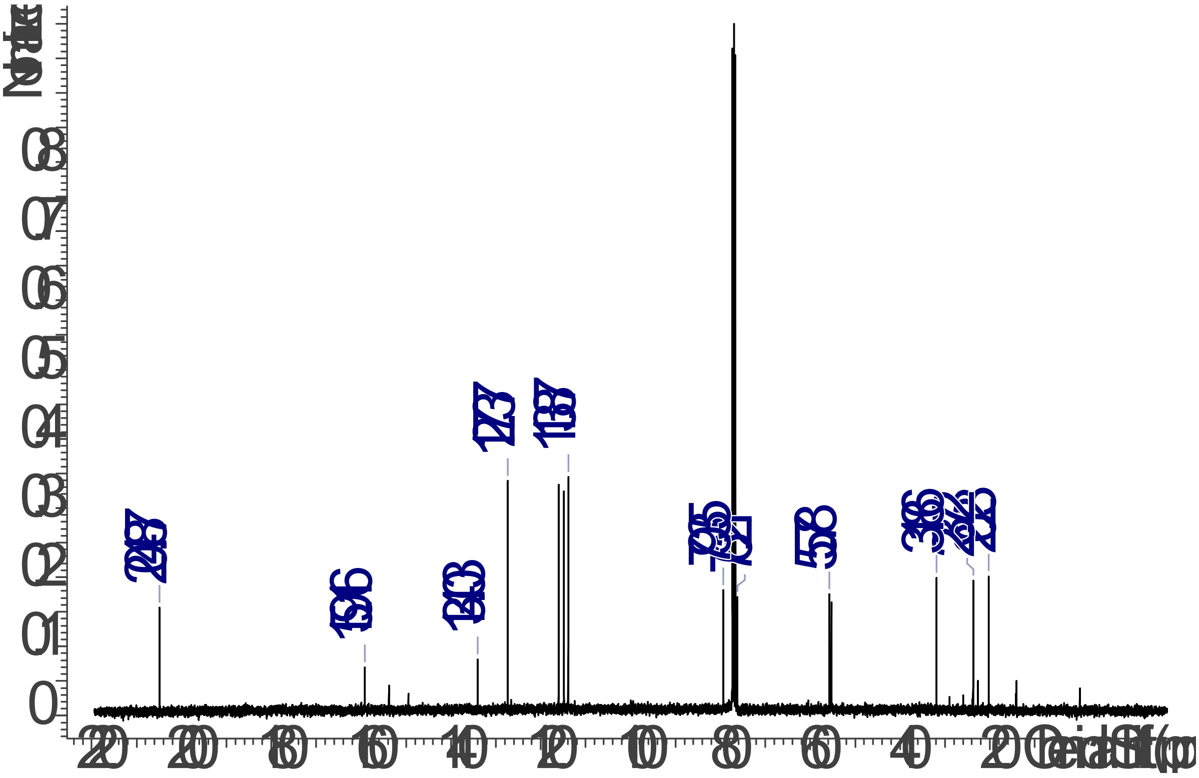

(4-(benzyloxy)benzyl)triphenylphosphonium bromide **4**

4 ^{1}H NMR (400 MHz, $CDCl_3$):

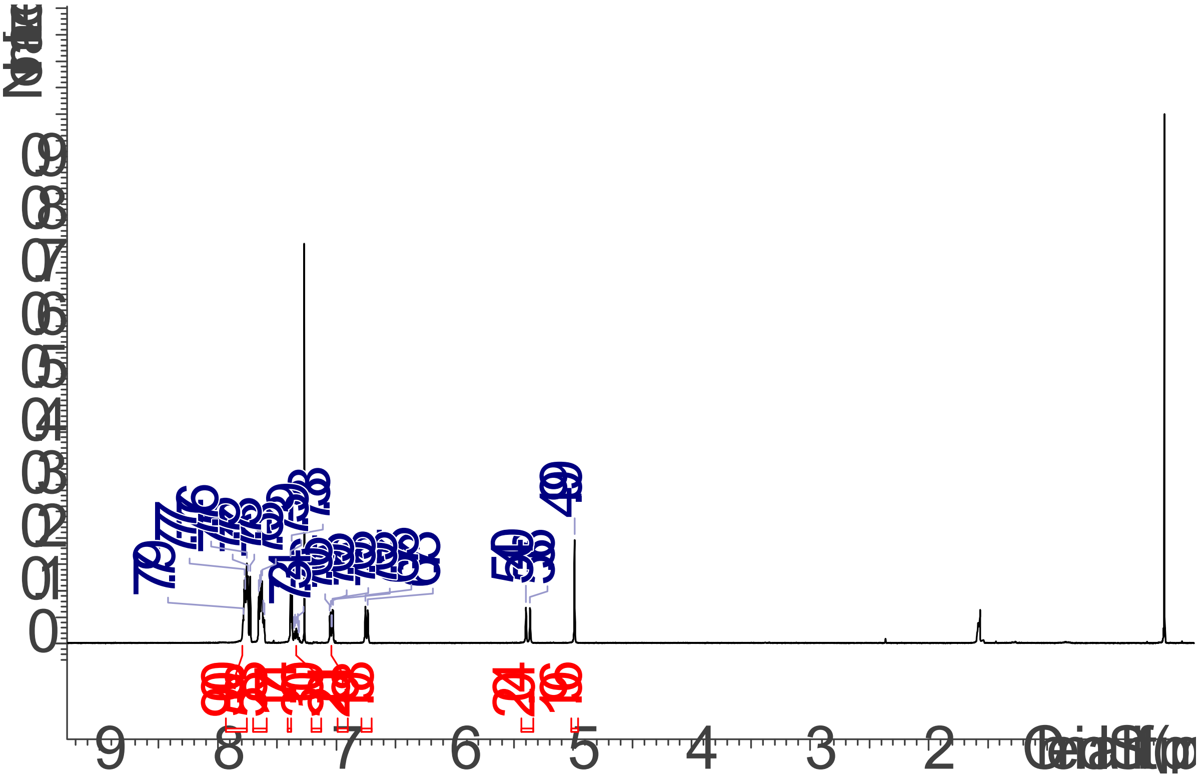

4 ^{13}C NMR (101 MHz, $CDCl_3$):

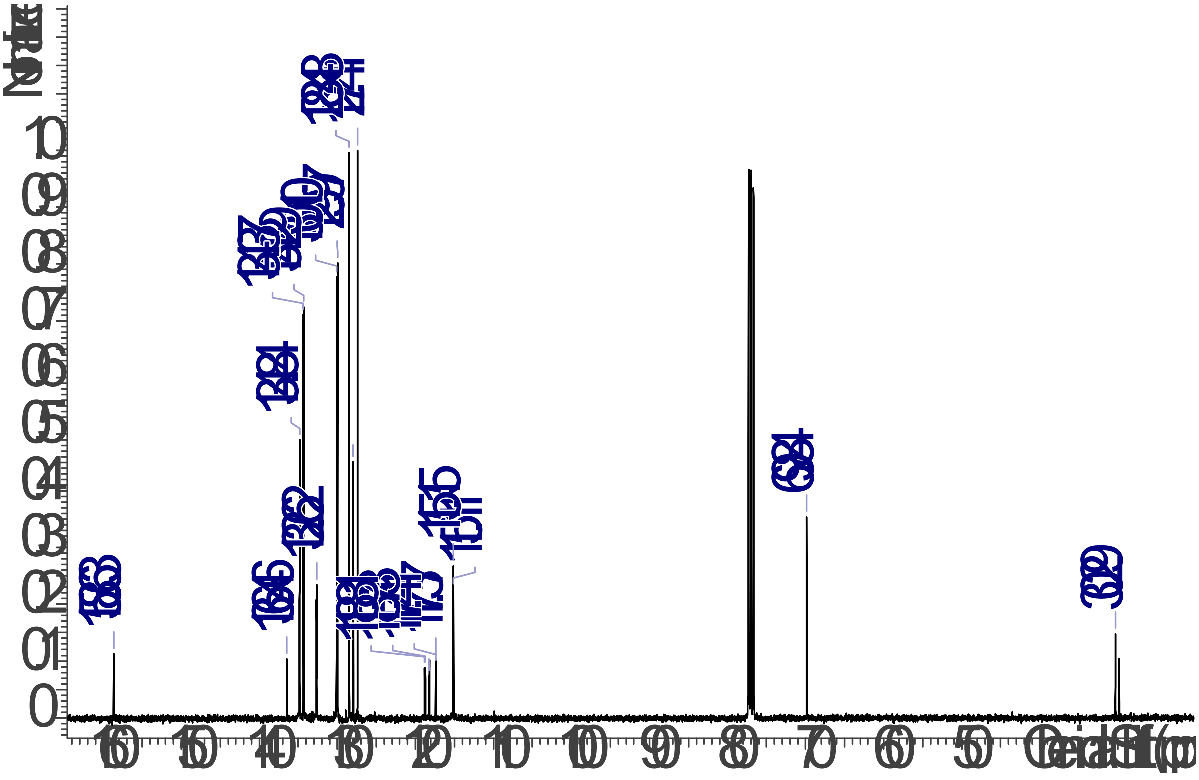

(2*S*,6*S*)-2-(4-(benzyloxy)styryl)-6-(4-methoxyphenyl)tetrahydro-2*H*-pyran **5**

5 ^{1}H NMR (400 MHz, $CDCl_3$):

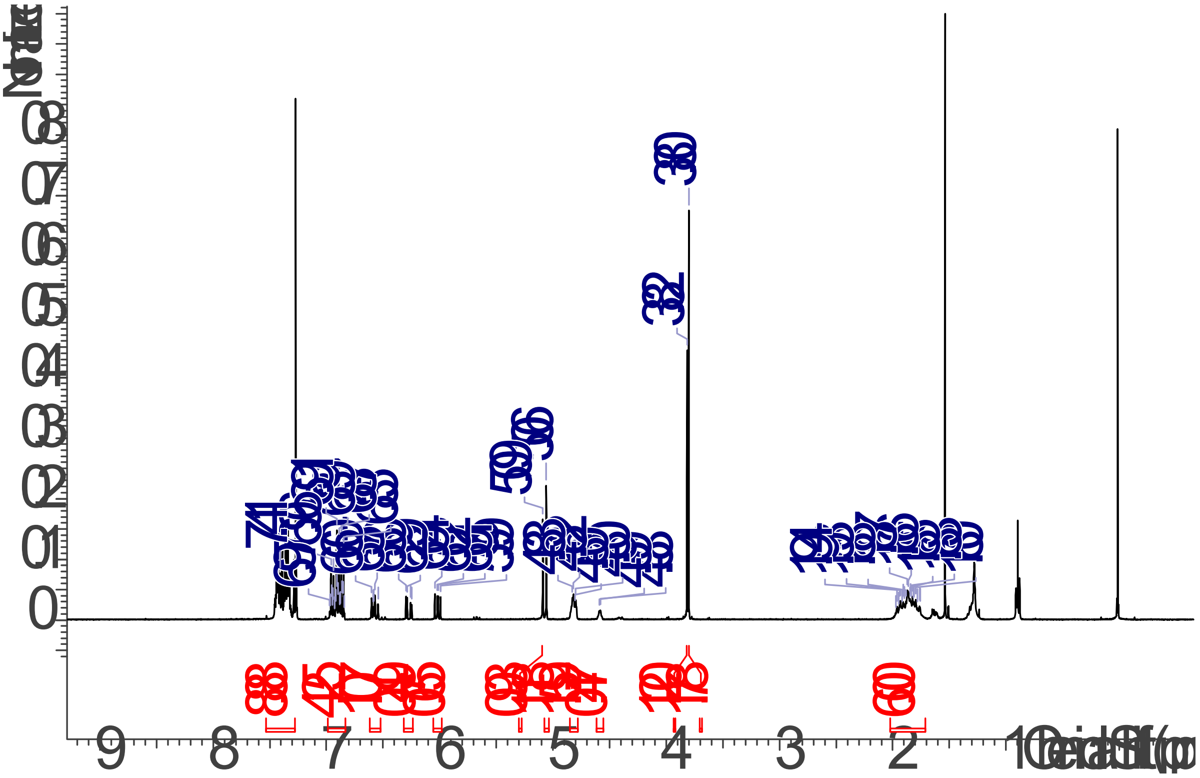

5 ^{13}C NMR (101 MHz, $CDCl_3$):

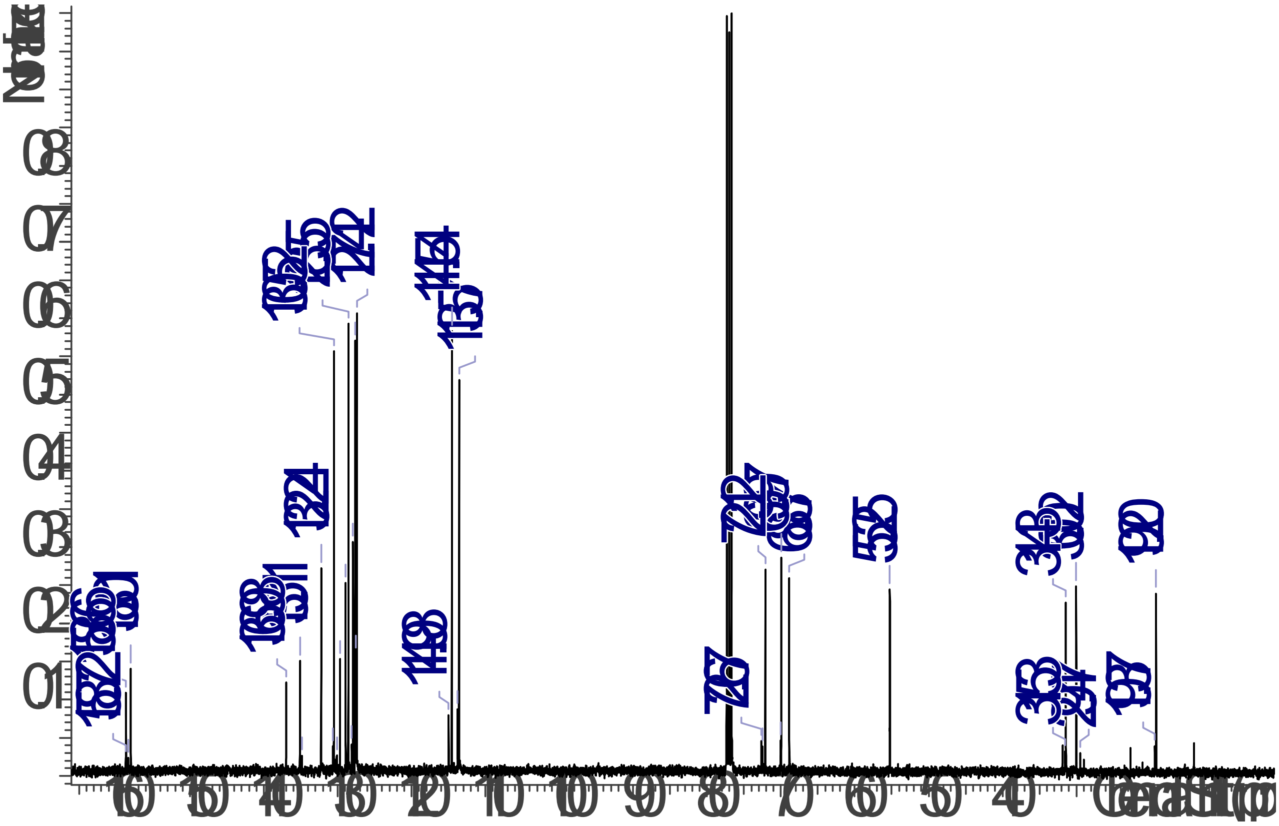

(3*S*,7*S*)-Centrolobine **6**

6 ^{1}H NMR (400 MHz, $CDCl_3$):

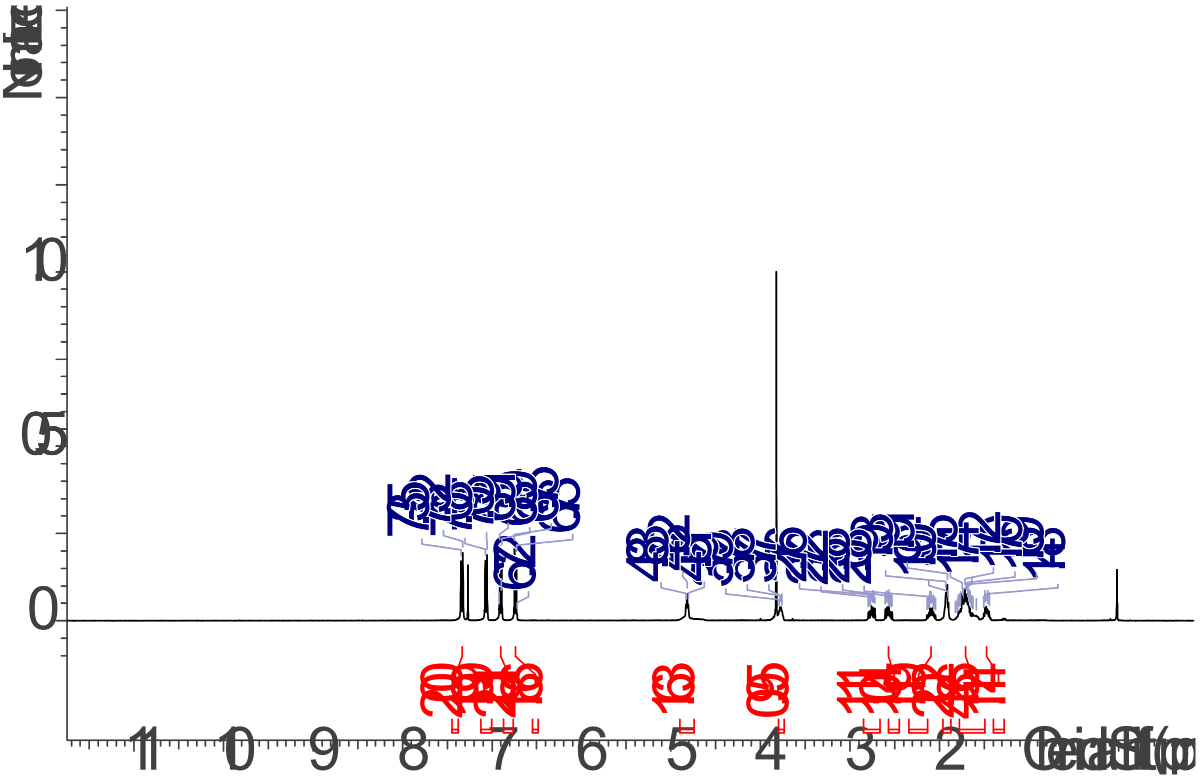

6 ^{13}C NMR (101 MHz, $CDCl_3$):

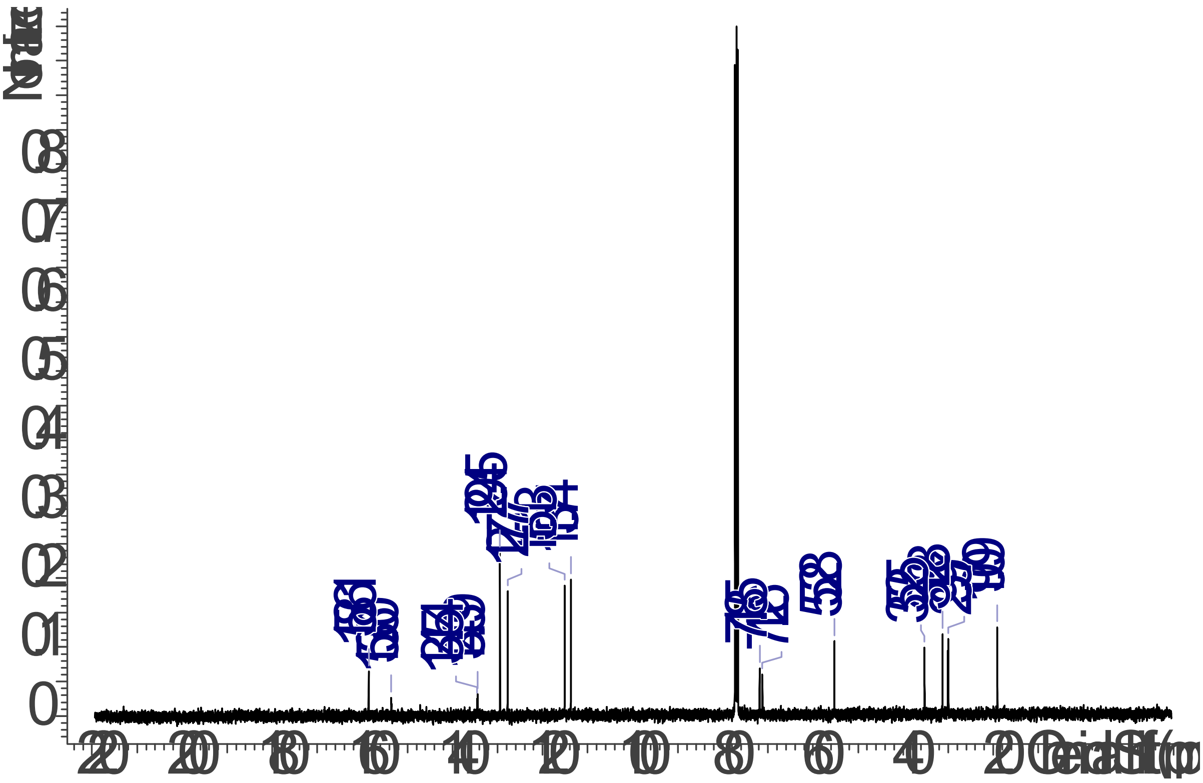

Appendix 1. Computational Studies

Protocol for calculations

Approximate model systems were constructed in MOE[10] and then geometry optimised at the quantum mechanical level of theory. Calculations were performed in Gaussian 16.[11] The chemical model chosen was Truhlar's hybrid functional M06L[12] with a basis set of 6-31G(d,p) on non-metal atoms[13] and LANL2DZ on the palladium.[14,15] Transition states were sought using the standard Berny algorithm with force constants calculated at the first iteration.[16] The integration grid set to UltraFine in all cases. Extra-step quadratically convergent self-consistent field was used. Occasionally SCF calculations would not converge, so later calculations also included a maximum iteration limit of 1000. The solvent was represented by the SMD model[17] with solvent set to DMF. A typical route is shown below.

```
# opt=(TS,calcfc,noeigen)
# int=(ultrafine) geom=(nocrowd)
# m06L/gen nosymm pseudo=read
# scrf=(smd,solvent=DMF) scf=(xqc,maxcycles=1000)
```

Computational study of the effect of the PyrOx ligand

In agreement with computational studies conducted by Correia and co-workers[18] on acyclic alkenol aryl ethers, the lowest energy transition state (TS1) with PyrOx L1 exhibited a key pyridinyl C–H aryl π-interaction, which, in conjunction with the steric repulsion between the tert-butyl group on the PyrOx ligand and DHP-alcohol **1**, are likely accountable for the excellent enantioselectivities observed (**Figure S4**).

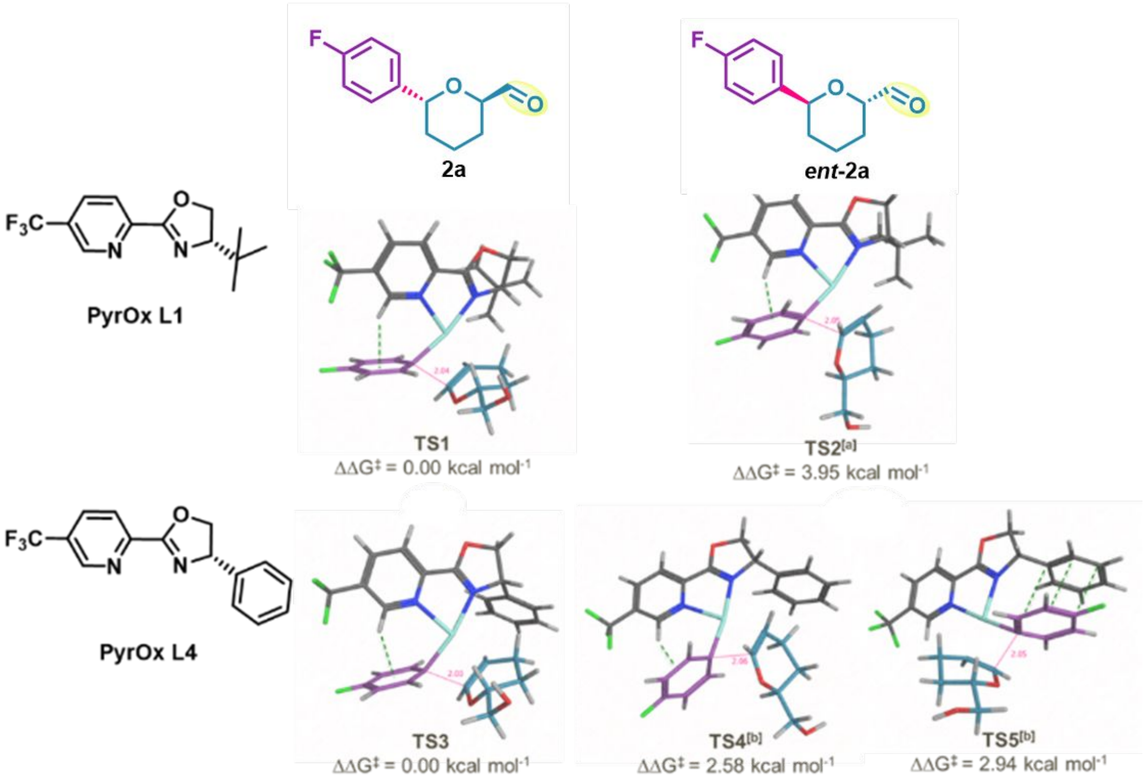

Figure S4. DFT calculations using Truhlar's hybrid functional M06L with a basis set of 6-31G(d,p) on non-metal atoms and LANL2DZ on the palladium. [a]Relative to TS1; [b]Relative to TS3.

For the phenyl-substituted analogue, PyrOx **L4**, although the transition state leading to the major product (**2a**) remained unchanged (**TS3**), it was found that there are two possible transition states of similar energies (**TS4** and **TS5**) leading to the antipodal product (***ent*-2a**). Whilst the geometry of **TS4** is comparable to **TS2**, a different geometry is adopted in **TS5**. Here, the reduction in transition state energy can be attributed to the off-set π-π stacking between the phenyl substituent of the PyrOx ligand and the aryl group to be inserted. Both of these transition states are more accessible compared to the corresponding *tert*-butyl ligand, providing a rationale for the observed erosion of enantioselectivity with the phenyl derivatives.

Representative geometries

E(M06L)= -1831.16548096 A.U.

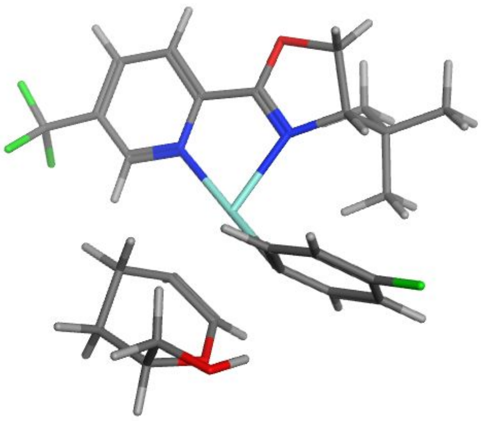

Center Number	Atomic Number	Atomic Type	Coordinates (Angstroms) X	Y	Z
1	6	0	3.310819	-0.091490	-0.180358
2	6	0	2.694678	1.198501	-0.485849
3	7	0	1.479516	1.484540	-0.165406
4	8	0	3.431904	2.132160	-1.088555
5	6	0	2.498373	3.229691	-1.350161
6	6	0	1.293931	2.918182	-0.454603
7	7	0	2.480260	-0.982561	0.412408
8	6	0	4.644477	-0.371262	-0.446148
9	6	0	5.151787	-1.614598	-0.084091
10	1	0	6.188789	-1.866291	-0.276140
11	6	0	4.300774	-2.523756	0.529472
12	6	0	2.970493	-2.170196	0.759625
13	46	0	0.365861	-0.372694	0.440595
14	6	0	-1.622629	0.142394	0.092564
15	6	0	-2.330554	1.111178	0.819164
16	6	0	-1.834054	0.041962	-1.294987
17	6	0	-2.698376	0.910885	-1.952340
18	6	0	-3.209030	1.979330	0.179815
19	6	0	-3.371058	1.863375	-1.194965
20	6	0	1.239673	3.752352	0.853244
21	1	0	2.262766	3.202508	-2.417294
22	1	0	3.020640	4.159185	-1.118251
23	1	0	0.348587	3.057997	-0.993167
24	1	0	5.270097	0.372581	-0.925820
25	1	0	2.282496	-2.862989	1.237699
26	1	0	-2.202196	1.185203	1.896582
27	1	0	-1.309015	-0.719109	-1.868489
28	1	0	-2.862704	0.854181	-3.023728

29	1	0	-3.765882	2.733019	0.727434
30	9	0	-4.224413	2.697925	-1.817880
31	6	0	0.075382	3.273408	1.714313
32	6	0	1.007797	5.213739	0.472585
33	6	0	2.541361	3.618417	1.642022
34	1	0	0.083069	5.331378	-0.103672
35	1	0	1.829826	5.625539	-0.122967
36	1	0	0.920245	5.829170	1.373957
37	1	0	-0.879590	3.397912	1.192962
38	1	0	0.027376	3.852653	2.642791
39	1	0	0.177449	2.214695	1.980913
40	1	0	2.726936	2.581153	1.944014
41	1	0	2.486367	4.217985	2.556294
42	1	0	3.411112	3.971860	1.077088
43	6	0	4.754548	-3.889490	0.945292
44	9	0	6.061786	-4.085587	0.711736
45	9	0	4.538941	-4.098346	2.260434
46	9	0	4.075127	-4.851543	0.285869
47	6	0	-0.491022	-2.187769	0.910330
48	6	0	-1.791387	-1.621754	1.131326
49	8	0	-2.932225	-2.146552	0.652313
50	6	0	-0.403790	-3.243012	-0.159811
51	1	0	0.055380	-2.368959	1.842536
52	1	0	-1.969294	-1.120698	2.079770
53	1	0	-0.380614	-2.773864	-1.152934
54	1	0	0.520643	-3.818188	-0.062975
55	6	0	-1.630953	-4.147505	-0.047149
56	6	0	-2.911990	-3.353147	-0.188605
57	1	0	-3.762819	-3.915336	0.209556
58	1	0	-1.618767	-4.658349	0.921666
59	1	0	-1.623240	-4.922830	-0.820444
60	6	0	-3.230787	-2.951873	-1.617792
61	1	0	-2.338906	-2.528773	-2.106751
62	1	0	-3.488800	-3.861854	-2.170005
63	8	0	-4.337946	-2.086570	-1.702668
64	1	0	-4.119626	-1.316913	-1.159862

E(M06L)= -1831.16618602 A.U.

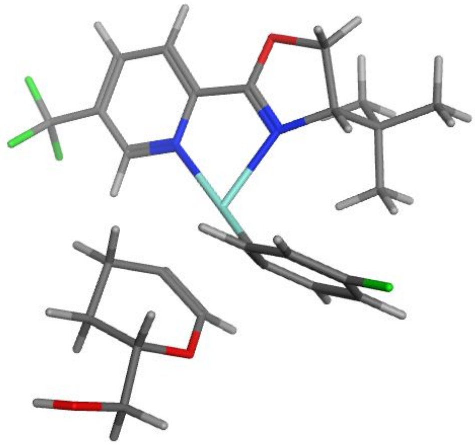

Center Number	Atomic Number	Atomic Type	Coordinates (Angstroms) X	Y	Z
1	6	0	3.326981	-0.097444	-0.184188
2	6	0	2.712234	1.194550	-0.484619
3	7	0	1.498397	1.482261	-0.160509
4	8	0	3.448139	2.126730	-1.091487
5	6	0	2.514259	3.223695	-1.353764
6	6	0	1.311330	2.914607	-0.455158
7	7	0	2.495143	-0.989986	0.404288
8	6	0	4.659389	-0.378845	-0.454872
9	6	0	5.163839	-1.626216	-0.102962
10	1	0	6.199783	-1.879443	-0.298687
11	6	0	4.311119	-2.537909	0.504520
12	6	0	2.982331	-2.182164	0.740176
13	46	0	0.378096	-0.373602	0.433140
14	6	0	-1.601946	0.141228	0.077930
15	6	0	-2.302111	1.113719	0.809064
16	6	0	-1.838928	0.026438	-1.306621
17	6	0	-2.719562	0.887827	-1.953055
18	6	0	-3.193466	1.973882	0.178490
19	6	0	-3.379257	1.844746	-1.192556
20	6	0	1.257963	3.755118	0.848537
21	1	0	2.276742	3.194184	-2.420416
22	1	0	3.036986	4.153689	-1.124953
23	1	0	0.364896	3.050233	-0.993090
24	1	0	5.286049	0.366486	-0.930884
25	1	0	2.292777	-2.877066	1.213182
26	1	0	-2.154175	1.196930	1.883122
27	1	0	-1.316441	-0.730722	-1.887116
28	1	0	-2.901189	0.825792	-3.021204
29	1	0	-3.740626	2.732901	0.728639
30	9	0	-4.242990	2.675669	-1.807759
31	6	0	0.095577	3.279382	1.713845
32	6	0	1.023854	5.214307	0.460770
33	6	0	2.561015	3.626556	1.635943

34	1	0	0.098523	5.327797	-0.115365
35	1	0	1.844858	5.624231	-0.137488
36	1	0	0.936122	5.834180	1.359083
37	1	0	-0.860373	3.400187	1.193470
38	1	0	0.048499	3.863235	2.639490
39	1	0	0.199278	2.222186	1.985565
40	1	0	2.748288	2.590982	1.942557
41	1	0	2.506796	4.230524	2.547368
42	1	0	3.429452	3.978253	1.067909
43	6	0	4.761760	-3.908199	0.908235
44	9	0	6.068512	-4.105585	0.672649
45	9	0	4.545932	-4.127943	2.221589
46	9	0	4.079903	-4.863020	0.240775
47	6	0	-0.488202	-2.195675	0.868923
48	6	0	-1.787732	-1.635069	1.110332
49	8	0	-2.929887	-2.134565	0.617575
50	6	0	-0.375111	-3.223184	-0.232663
51	1	0	0.057057	-2.388641	1.798937
52	1	0	-1.965397	-1.168492	2.075746
53	1	0	-0.183138	-2.726892	-1.193857
54	1	0	0.476934	-3.883401	-0.048764
55	6	0	-1.679005	-4.006884	-0.342927
56	6	0	-2.845226	-3.056938	-0.503262
57	1	0	-2.715291	-2.450799	-1.412310
58	1	0	-1.836878	-4.622543	0.552611
59	1	0	-1.651976	-4.679201	-1.205897
60	6	0	-4.204321	-3.735748	-0.542729
61	1	0	-4.364946	-4.281389	0.399633
62	1	0	-4.985188	-2.971701	-0.614145
63	8	0	-4.332575	-4.569299	-1.674017
64	1	0	-3.859271	-5.387639	-1.483567

E(M06L)= -1831.16666088 A.U.

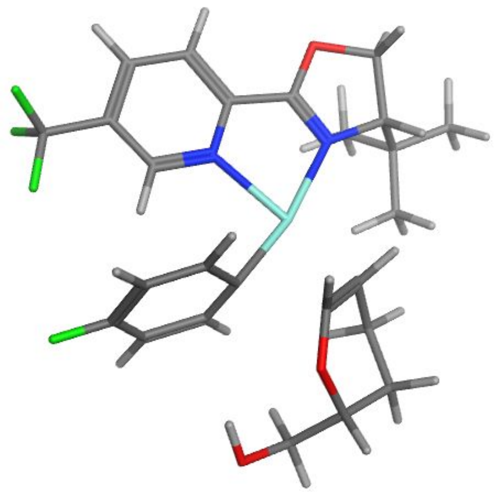

Center Number	Atomic Number	Atomic Type	Coordinates (Angstroms) X	Y	Z

1	6	0	3.327471	-0.073453	-0.038099
2	6	0	2.718740	1.211110	-0.379356
3	7	0	1.477131	1.481193	-0.150769
4	8	0	3.486584	2.154037	-0.922138
5	6	0	2.574458	3.252788	-1.237840
6	6	0	1.283855	2.905077	-0.484931
7	6	0	1.030933	3.754934	0.785413
8	6	0	-0.231014	3.245034	1.471089
9	6	0	2.211214	3.663059	1.750169
10	6	0	0.818032	5.204357	0.353038
11	7	0	2.456483	-0.971648	0.464091
12	6	0	4.683513	-0.344260	-0.189370
13	6	0	5.156111	-1.588924	0.205885
14	1	0	6.207378	-1.839508	0.101844
15	6	0	4.256761	-2.507969	0.739715
16	6	0	2.914174	-2.160138	0.853579
17	46	0	0.318601	-0.335255	0.419546
18	6	0	-0.421991	-2.236913	0.738419
19	6	0	-0.442335	-2.504636	2.118050
20	6	0	-0.085571	-3.269029	-0.154683
21	6	0	0.239779	-4.536705	0.314219
22	6	0	-0.102867	-3.765240	2.603519
23	6	0	0.232107	-4.755881	1.687511
24	1	0	2.453613	3.267381	-2.323710
25	1	0	3.058430	4.176590	-0.915991
26	1	0	0.409498	3.000772	-1.139224
27	1	0	-0.113625	2.206789	1.802415
28	1	0	-1.089790	3.288733	0.790939
29	1	0	-0.468093	3.855522	2.349010
30	1	0	2.377694	2.633725	2.087891
31	1	0	2.016136	4.270983	2.639432
32	1	0	3.142468	4.032278	1.305858
33	1	0	-0.017046	5.290347	-0.351516
34	1	0	1.706917	5.630542	-0.124809
35	1	0	0.586743	5.827529	1.223037
36	1	0	5.348736	0.403632	-0.605336
37	1	0	2.184606	-2.850417	1.266502
38	1	0	-0.703463	-1.717889	2.821853
39	1	0	-0.081098	-3.079973	-1.225656
40	1	0	0.501636	-5.345831	-0.359891
41	1	0	-0.094259	-3.984592	3.666396
42	9	0	0.556959	-5.978058	2.147255
43	6	0	4.735556	-3.849099	1.202134
44	9	0	5.485425	-4.455894	0.261377
45	9	0	5.508157	-3.739410	2.302583
46	9	0	3.717626	-4.676249	1.505874
47	6	0	-1.717968	0.114560	0.288073
48	6	0	-2.013356	-1.244145	-0.076693
49	8	0	-3.052876	-1.953382	0.402938
50	6	0	-2.421087	0.608090	1.524874

51	1	0	-1.668848	0.791044	-0.570586
52	1	0	-1.787915	-1.553805	-1.094718
53	6	0	-3.870180	0.118903	1.493156
54	1	0	-4.378877	0.554487	0.626352
55	1	0	-4.414124	0.448830	2.384558
56	6	0	-3.945616	-1.389891	1.422653
57	1	0	-4.927900	-1.714741	1.064480
58	1	0	-2.389886	1.696237	1.586728
59	1	0	-1.920107	0.226624	2.425473
60	6	0	-3.667067	-2.080253	2.746343
61	1	0	-2.771803	-1.650168	3.222642
62	1	0	-4.508947	-1.871710	3.415176
63	8	0	-3.574712	-3.479376	2.618518
64	1	0	-2.882302	-3.644142	1.964308

E(M06L)= -1831.16765838 A.U.

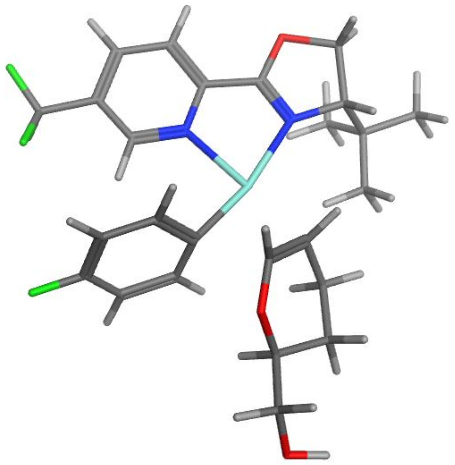

Center Number	Atomic Number	Atomic Type	Coordinates (Angstroms) X	Y	Z
1	6	0	3.356566	-0.062451	-0.036058
2	6	0	2.741825	1.222554	-0.363645
3	7	0	1.501507	1.487803	-0.125015
4	8	0	3.502981	2.169677	-0.909483
5	6	0	2.586367	3.267758	-1.212921
6	6	0	1.298032	2.909556	-0.460041
7	6	0	1.035220	3.758776	0.808404
8	6	0	-0.225186	3.240133	1.490559
9	6	0	2.213413	3.675927	1.776395
10	6	0	0.813249	5.206173	0.373803
11	7	0	2.494635	-0.965232	0.479013
12	6	0	4.707457	-0.332272	-0.219013
13	6	0	5.190656	-1.581941	0.153040
14	1	0	6.237348	-1.832776	0.018369
15	6	0	4.304194	-2.503165	0.698192
16	6	0	2.962727	-2.154170	0.848019

17	46	0	0.351883	-0.335812	0.446600
18	6	0	-0.407459	-2.225614	0.760668
19	6	0	-0.511526	-2.513396	2.134561
20	6	0	-0.050098	-3.255642	-0.129031
21	6	0	0.213513	-4.537920	0.335846
22	6	0	-0.241422	-3.792996	2.614419
23	6	0	0.115199	-4.778084	1.702626
24	1	0	2.463036	3.292313	-2.298325
25	1	0	3.067685	4.190415	-0.883612
26	1	0	0.423464	2.997197	-1.115433
27	1	0	-0.101988	2.202407	1.821071
28	1	0	-1.082616	3.278841	0.808543
29	1	0	-0.468449	3.848212	2.368500
30	1	0	2.386489	2.647813	2.114454
31	1	0	2.011812	4.282785	2.664938
32	1	0	3.143141	4.051629	1.334277
33	1	0	-0.020365	5.285311	-0.333322
34	1	0	1.700484	5.637946	-0.102149
35	1	0	0.574997	5.828941	1.242218
36	1	0	5.362430	0.418642	-0.645688
37	1	0	2.244238	-2.852400	1.269430
38	1	0	-0.780655	-1.730060	2.838632
39	1	0	0.018549	-3.050604	-1.194775
40	1	0	0.494342	-5.343681	-0.334836
41	1	0	-0.298451	-4.030143	3.671860
42	9	0	0.370933	-6.018856	2.158446
43	6	0	4.753288	-3.857171	1.153565
44	9	0	3.818961	-4.796368	0.899023
45	9	0	5.892062	-4.239913	0.551071
46	9	0	4.978092	-3.882178	2.484201
47	6	0	-1.684724	0.118192	0.322435
48	6	0	-1.986098	-1.229140	-0.079732
49	8	0	-3.022281	-1.950726	0.380269
50	6	0	-2.365134	0.606293	1.580021
51	1	0	-1.627666	0.807822	-0.524750
52	1	0	-1.770690	-1.508416	-1.108369
53	6	0	-3.744219	-0.034464	1.711457
54	1	0	-4.409612	0.325802	0.915323
55	1	0	-4.200860	0.234186	2.669089
56	6	0	-3.643102	-1.539725	1.625576
57	1	0	-3.016113	-1.924334	2.444241
58	1	0	-2.444439	1.694718	1.573476
59	1	0	-1.767171	0.344598	2.464026
60	6	0	-4.982904	-2.257006	1.642331
61	1	0	-5.581583	-1.925960	0.779911
62	1	0	-4.816582	-3.333276	1.529969
63	8	0	-5.655907	-2.065674	2.867725
64	1	0	-6.048173	-1.184956	2.845711

E(M06L)= -1831.17395392 A.U.

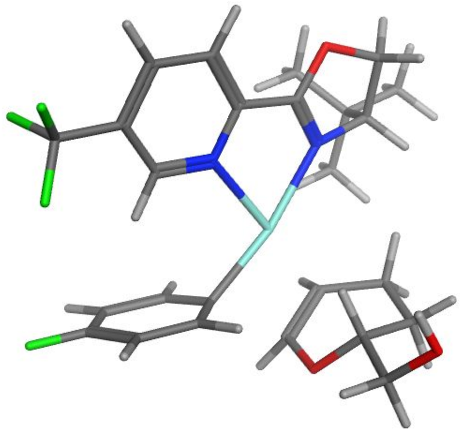

Center Number	Atomic Number	Atomic Type	Coordinates (Angstroms) X	Y	Z
1	6	0	3.333132	-0.059722	-0.045755
2	6	0	2.713480	1.224912	-0.364888
3	7	0	1.462802	1.468396	-0.157464
4	8	0	3.481030	2.203715	-0.840058
5	6	0	2.558870	3.304268	-1.121690
6	6	0	1.266054	2.909912	-0.397832
7	6	0	1.005830	3.675607	0.924959
8	6	0	-0.240277	3.110558	1.600900
9	6	0	2.195882	3.548471	1.874060
10	6	0	0.766405	5.143694	0.577427
11	7	0	2.473586	-0.965938	0.463993
12	6	0	4.690184	-0.316199	-0.207750
13	6	0	5.181380	-1.554693	0.187840
14	1	0	6.233651	-1.794725	0.074273
15	6	0	4.296895	-2.479149	0.733878
16	6	0	2.949998	-2.144886	0.854731
17	46	0	0.308886	-0.332751	0.439389
18	6	0	-0.420790	-2.187401	1.066457
19	6	0	-0.314408	-2.312010	2.460555
20	6	0	-0.160996	-3.298882	0.252449
21	6	0	0.248561	-4.505211	0.811663
22	6	0	0.107809	-3.509796	3.032423
23	6	0	0.386054	-4.581495	2.192871
24	9	0	0.790935	-5.745569	2.738409
25	1	0	2.448176	3.360154	-2.207484
26	1	0	3.027833	4.220029	-0.757923
27	1	0	0.396484	3.053818	-1.049904
28	1	0	-0.091226	2.068901	1.906557
29	1	0	-1.109457	3.147543	0.933536
30	1	0	-0.481768	3.692496	2.496806
31	1	0	2.388730	2.503054	2.142077
32	1	0	1.992570	4.090460	2.803239
33	1	0	3.114741	3.969109	1.450585
34	1	0	-0.084971	5.256483	-0.103501

35	1	0	1.639299	5.606520	0.104058
36	1	0	0.547061	5.715626	1.484852
37	1	0	5.343113	0.438267	-0.631314
38	1	0	2.232831	-2.843105	1.274692
39	1	0	-0.564644	-1.471427	3.103598
40	1	0	-0.280592	-3.226181	-0.825598
41	1	0	0.469294	-5.372944	0.198223
42	1	0	0.211740	-3.623189	4.106716
43	6	0	4.776340	-3.809902	1.225464
44	9	0	5.755852	-4.304683	0.446826
45	9	0	5.274974	-3.723069	2.476651
46	9	0	3.781636	-4.718717	1.259079
47	6	0	-1.694942	0.207118	0.383293
48	6	0	-2.052273	-1.187737	0.373614
49	8	0	-2.372336	-1.806872	-0.777040
50	6	0	-2.025458	1.064521	-0.820872
51	1	0	-1.828975	0.675118	1.360414
52	1	0	-2.614841	-1.596982	1.207837
53	1	0	-2.825348	1.758033	-0.533552
54	6	0	-2.450426	0.240711	-2.035609
55	1	0	-3.541909	0.139998	-2.074979
56	6	0	-1.869365	-1.152442	-1.965249
57	1	0	-0.765457	-1.095279	-1.859835
58	1	0	-2.139075	0.713766	-2.970151
59	1	0	-1.168312	1.700102	-1.068288
60	6	0	-2.242662	-2.053486	-3.126832
61	1	0	-1.875725	-3.072471	-2.933019
62	1	0	-3.334726	-2.109819	-3.203010
63	8	0	-1.775543	-1.537385	-4.354202
64	1	0	-0.816093	-1.640741	-4.360098

E(M06L)= -1831.17070227 A.U.

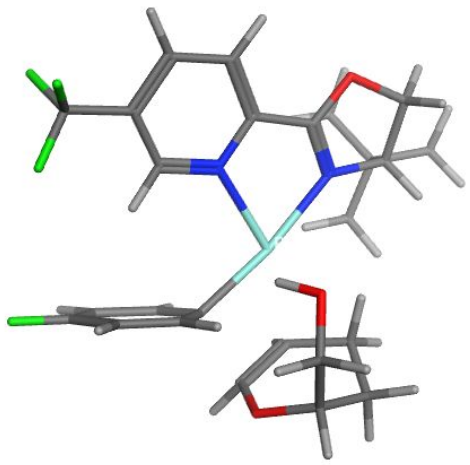

Center Number	Atomic Number	Atomic Type	Coordinates (Angstroms) X	Y	Z

1	6	0	3.498903	-0.031302	0.057599
2	6	0	2.863018	1.244798	-0.266166
3	7	0	1.605545	1.461871	-0.071494
4	8	0	3.610700	2.241212	-0.733986
5	6	0	2.662190	3.318000	-1.029746
6	6	0	1.373641	2.896309	-0.313076
7	6	0	1.091545	3.654953	1.009745
8	6	0	-0.138224	3.057092	1.687692
9	6	0	2.283915	3.561774	1.960040
10	6	0	0.811520	5.115307	0.660292
11	7	0	2.644614	-0.952412	0.548747
12	6	0	4.861902	-0.268468	-0.085556
13	6	0	5.361166	-1.504026	0.306589
14	1	0	6.418822	-1.729772	0.209979
15	6	0	4.479832	-2.446470	0.828778
16	6	0	3.128001	-2.131238	0.932899
17	46	0	0.474715	-0.336450	0.467437
18	6	0	-0.304478	-2.218117	0.969023
19	6	0	-0.261355	-2.388687	2.363329
20	6	0	0.018636	-3.296025	0.134816
21	6	0	0.431976	-4.511363	0.672456
22	6	0	0.161195	-3.593946	2.914960
23	6	0	0.504630	-4.631280	2.054800
24	9	0	0.906740	-5.803655	2.582359
25	1	0	2.558458	3.362089	-2.116685
26	1	0	3.106484	4.247268	-0.669471
27	1	0	0.502742	3.014432	-0.968339
28	1	0	0.036558	2.019190	1.992927
29	1	0	-1.009502	3.072330	1.022245
30	1	0	-0.392717	3.632749	2.584051
31	1	0	2.503809	2.522666	2.231676
32	1	0	2.065080	4.100604	2.887523
33	1	0	3.191966	4.005278	1.536776
34	1	0	-0.043344	5.203259	-0.019946
35	1	0	1.670945	5.600750	0.185030
36	1	0	0.577656	5.682696	1.566942
37	1	0	5.512056	0.498139	-0.491238
38	1	0	2.412522	-2.841281	1.334641
39	1	0	-0.564252	-1.576184	3.019914
40	1	0	-0.063441	-3.195865	-0.944584
41	1	0	0.695061	-5.353984	0.041224
42	1	0	0.215868	-3.741621	3.988632
43	6	0	4.987832	-3.777474	1.289234
44	9	0	5.737400	-4.372781	0.340880
45	9	0	5.771322	-3.650576	2.379922
46	9	0	3.988773	-4.621942	1.607140
47	6	0	-1.508267	0.206634	0.256001
48	6	0	-1.868863	-1.191734	0.249342
49	8	0	-2.236067	-1.844830	-0.871220
50	6	0	-1.755881	1.038956	-0.982978
51	1	0	-1.713905	0.682309	1.217448

52	1	0	-2.427804	-1.553574	1.109554
53	1	0	-2.190511	1.998086	-0.682238
54	6	0	-2.691667	0.314310	-1.943467
55	1	0	-3.713449	0.341957	-1.548198
56	6	0	-2.309883	-1.136215	-2.151945
57	1	0	-3.134810	-1.658557	-2.649722
58	1	0	-2.710703	0.813842	-2.916799
59	1	0	-0.817743	1.270727	-1.495655
60	6	0	-1.088886	-1.401128	-3.013948
61	1	0	-0.887587	-2.482901	-3.014050
62	1	0	-1.366181	-1.132608	-4.039511
63	8	0	0.076709	-0.650501	-2.721465
64	1	0	0.461571	-0.990217	-1.896659

E(M06L)= -1831.16999734 A.U.

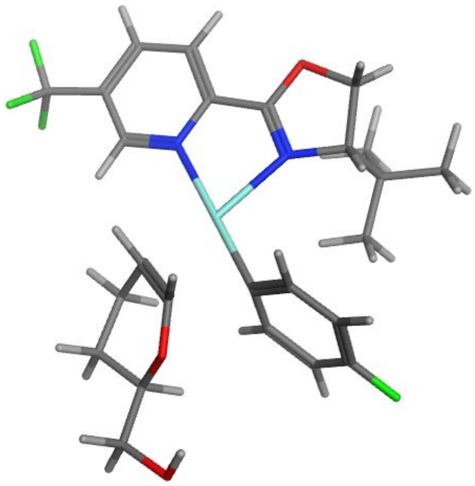

Center Number	Atomic Number	Atomic Type	Coordinates (Angstroms) X	Y	Z
1	6	0	3.350660	-0.062683	-0.075912
2	6	0	2.731822	1.219190	-0.407377
3	7	0	1.498753	1.489610	-0.148661
4	8	0	3.482481	2.152946	-0.993738
5	6	0	2.551031	3.231835	-1.322342
6	6	0	1.288362	2.901603	-0.516127
7	7	0	2.495129	-0.986480	0.422054
8	6	0	4.706401	-0.309107	-0.247574
9	6	0	5.211625	-1.550783	0.121261
10	1	0	6.265594	-1.776957	0.004366
11	6	0	4.335158	-2.494351	0.640101
12	6	0	2.982633	-2.175953	0.769129
13	46	0	0.373303	-0.377360	0.439079
14	6	0	-1.627067	0.180749	0.423687
15	6	0	-2.112895	0.515938	1.703049
16	6	0	-2.165673	0.836910	-0.698923

17	6	0	-3.157229	1.799102	-0.556104
18	6	0	-3.110857	1.471070	1.861907
19	6	0	-3.615675	2.088693	0.724961
20	6	0	1.071572	3.792216	0.732922
21	1	0	2.395591	3.202827	-2.403759
22	1	0	3.036057	4.171678	-1.052976
23	1	0	0.390109	2.972262	-1.141616
24	1	0	5.350741	0.458689	-0.659959
25	1	0	2.272783	-2.901609	1.158392
26	1	0	-1.698367	0.035184	2.585845
27	1	0	-1.818236	0.575072	-1.695896
28	1	0	-3.586215	2.311302	-1.411306
29	1	0	-3.491742	1.744892	2.840563
30	9	0	-4.592497	3.004012	0.866746
31	6	0	-0.131545	3.269789	1.509595
32	6	0	0.782960	5.212583	0.250426
33	6	0	2.303608	3.784521	1.635489
34	1	0	-0.107340	5.240843	-0.388219
35	1	0	1.618105	5.635935	-0.318769
36	1	0	0.603048	5.873755	1.104389
37	1	0	-1.014170	3.189743	0.864301
38	1	0	-0.379442	3.944664	2.336262
39	1	0	0.062621	2.278162	1.931735
40	1	0	2.540135	2.773335	1.986261
41	1	0	2.122259	4.402128	2.521113
42	1	0	3.190701	4.190479	1.136345
43	6	0	4.791640	-3.855830	1.067044
44	9	0	6.102204	-4.045403	0.845233
45	9	0	4.569016	-4.057406	2.382702
46	9	0	4.123396	-4.826507	0.409935
47	6	0	-0.497630	-2.232410	0.707005
48	6	0	-1.731704	-1.826855	0.087905
49	8	0	-2.947544	-2.151690	0.555390
50	6	0	-0.581214	-2.754338	2.121265
51	1	0	0.144659	-2.782468	0.011348
52	1	0	-1.753827	-1.764549	-0.997300
53	1	0	-0.540986	-1.923692	2.838485
54	1	0	0.276375	-3.395726	2.340376
55	6	0	-1.899611	-3.501361	2.315042
56	1	0	-2.023037	-3.813903	3.356337
57	1	0	-1.921116	-4.406873	1.695485
58	6	0	-3.058798	-2.608980	1.938981
59	1	0	-3.070343	-1.704312	2.563316
60	6	0	-4.414542	-3.272957	2.003690
61	1	0	-4.409666	-4.164748	1.355229
62	1	0	-4.587392	-3.614574	3.028939
63	8	0	-5.462422	-2.387309	1.677983
64	1	0	-5.286745	-2.078538	0.780592

E(M06L)= -1831.16255865 A.U.

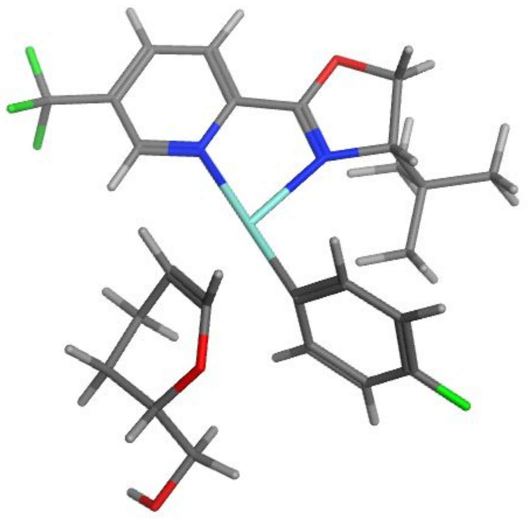

Center Number	Atomic Number	Atomic Type	Coordinates (Angstroms) X	Y	Z
1	6	0	3.345571	-0.066029	-0.076505
2	6	0	2.731643	1.217965	-0.408161
3	7	0	1.499698	1.494275	-0.151210
4	8	0	3.488477	2.147809	-0.993479
5	6	0	2.563071	3.230611	-1.324770
6	6	0	1.299128	2.908247	-0.517926
7	7	0	2.486724	-0.990202	0.415107
8	6	0	4.701972	-0.312988	-0.242764
9	6	0	5.205291	-1.555492	0.125345
10	1	0	6.259801	-1.781631	0.013423
11	6	0	4.325582	-2.499947	0.636782
12	6	0	2.972625	-2.181233	0.759514
13	46	0	0.363667	-0.380308	0.425822
14	6	0	-1.643233	0.175823	0.390694
15	6	0	-2.126191	0.529717	1.662339
16	6	0	-2.153611	0.842000	-0.737973
17	6	0	-3.114010	1.837725	-0.604725
18	6	0	-3.092295	1.515947	1.814974
19	6	0	-3.570140	2.146645	0.672201
20	6	0	1.091086	3.798809	0.732545
21	1	0	2.407510	3.200101	-2.406172
22	1	0	3.052766	4.168606	-1.057368
23	1	0	0.400542	2.986278	-1.142057
24	1	0	5.348279	0.455749	-0.650232
25	1	0	2.261655	-2.909262	1.141572
26	1	0	-1.742469	0.024699	2.545950
27	1	0	-1.810793	0.563048	-1.732130
28	1	0	-3.523515	2.357719	-1.464751
29	1	0	-3.473436	1.800449	2.790745
30	9	0	-4.520460	3.090332	0.805387
31	6	0	-0.117217	3.285692	1.506786

32	6	0	0.815296	5.222706	0.252963
33	6	0	2.323023	3.778371	1.634971
34	1	0	-0.073165	5.259845	-0.387810
35	1	0	1.655310	5.640693	-0.312995
36	1	0	0.638642	5.883158	1.108170
37	1	0	-0.999959	3.221518	0.860322
38	1	0	-0.357451	3.957724	2.338011
39	1	0	0.064512	2.288622	1.921831
40	1	0	2.548931	2.764968	1.986289
41	1	0	2.148361	4.398367	2.520272
42	1	0	3.214314	4.174552	1.135404
43	6	0	4.779203	-3.862789	1.063166
44	9	0	6.090450	-4.052953	0.846007
45	9	0	4.551568	-4.066436	2.377697
46	9	0	4.112654	-4.831849	0.402032
47	6	0	-0.504411	-2.234179	0.662533
48	6	0	-1.754810	-1.830368	0.073500
49	8	0	-2.958249	-2.175375	0.562087
50	6	0	-0.597526	-2.794705	2.055721
51	1	0	0.133951	-2.777508	-0.043198
52	1	0	-1.794803	-1.734908	-1.009261
53	1	0	-0.744896	-1.989472	2.787996
54	1	0	0.325866	-3.307431	2.335183
55	6	0	-1.791689	-3.746903	2.089274
56	1	0	-1.908630	-4.214879	3.072515
57	1	0	-1.630349	-4.559439	1.372344
58	6	0	-3.074669	-3.013445	1.756519
59	1	0	-3.852893	-3.731261	1.474601
60	6	0	-3.639718	-2.187766	2.907302
61	1	0	-2.875168	-1.531737	3.344260
62	1	0	-4.442304	-1.547044	2.526439
63	8	0	-4.207070	-3.039837	3.884567
64	1	0	-3.491638	-3.340894	4.456229

E(M06L)= -1904.95754990 A.U.

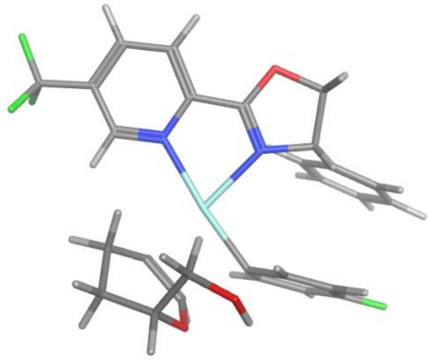

Center Number	Atomic Number	Atomic Type	Coordinates (Angstroms) X	Y	Z

1	6	0	3.210645	-0.374544	-0.444245
2	6	0	2.548311	0.860345	-0.858040
3	7	0	1.303330	1.086592	-0.620484
4	8	0	3.265157	1.803435	-1.467509
5	6	0	2.312323	2.863248	-1.792534
6	6	0	1.014142	2.458604	-1.057790
7	7	0	2.406684	-1.255785	0.198857
8	6	0	4.561012	-0.608722	-0.666156
9	6	0	5.120261	-1.791897	-0.197129
10	1	0	6.171730	-2.006831	-0.351670
11	6	0	4.301377	-2.687432	0.476789
12	6	0	2.950941	-2.383181	0.653674
13	46	0	0.288243	-0.609520	0.407935
14	6	0	-1.597605	0.247779	0.691667
15	6	0	-1.692145	0.998248	1.876449
16	6	0	-2.178719	0.746112	-0.482156
17	6	0	-2.813431	1.983903	-0.488479
18	6	0	-2.328942	2.232935	1.887623
19	6	0	-2.875967	2.701280	0.699347
20	1	0	2.202788	2.877107	-2.878854
21	1	0	2.742295	3.806134	-1.451952
22	1	0	0.169119	2.442729	-1.755009
23	1	0	5.159993	0.125229	-1.192782
24	1	0	2.291146	-3.068696	1.178089
25	1	0	-1.280352	0.600854	2.801881
26	1	0	-2.112811	0.183522	-1.409904
27	1	0	-3.250059	2.396287	-1.392689
28	1	0	-2.405492	2.829614	2.791115
29	9	0	-3.491275	3.899803	0.698391
30	6	0	4.821536	-3.974239	1.040670
31	9	0	4.068219	-5.021702	0.647181
32	9	0	6.086944	-4.218542	0.665124
33	9	0	4.793310	-3.963680	2.389926
34	6	0	0.667121	3.351483	0.110341
35	6	0	1.246407	3.158628	1.368202
36	1	0	1.926801	2.324566	1.529227
37	6	0	0.944161	4.018922	2.421137
38	1	0	1.394397	3.855788	3.396680
39	6	0	0.067232	5.084934	2.225812
40	1	0	-0.169799	5.753719	3.048707
41	6	0	-0.507844	5.286461	0.972466
42	1	0	-1.201291	6.108234	0.815060
43	6	0	-0.211337	4.421462	-0.077922
44	1	0	-0.673511	4.567051	-1.052842
45	6	0	-0.599246	-2.297935	1.196374
46	6	0	-1.912293	-1.708713	1.063035
47	8	0	-2.830326	-2.141436	0.168939
48	6	0	-0.252903	-3.523581	0.371714
49	1	0	-0.242560	-2.284751	2.229312
50	1	0	-2.407102	-1.412052	1.982689
51	1	0	0.415109	-3.262951	-0.460335

52	1	0	0.301683	-4.232288	0.993501
53	6	0	-1.514603	-4.170767	-0.179151
54	6	0	-2.415007	-3.140250	-0.819071
55	1	0	-3.367242	-3.593110	-1.113679
56	1	0	-2.070873	-4.659900	0.627949
57	1	0	-1.270931	-4.939663	-0.917681
58	6	0	-1.836856	-2.425390	-2.020592
59	1	0	-0.995135	-1.779192	-1.702716
60	1	0	-1.430908	-3.161612	-2.722245
61	8	0	-2.821026	-1.684447	-2.720964
62	1	0	-3.364629	-1.243700	-2.055183

E(M06L)= -1904.95621184 A.U.

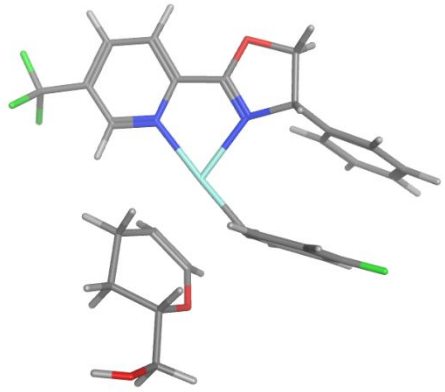

Center Number	Atomic Number	Atomic Type	Coordinates (Angstroms) X	Y	Z
1	6	0	3.637242	-0.298196	-0.269330
2	6	0	3.045514	1.013252	-0.526698
3	7	0	1.803661	1.269489	-0.299203
4	8	0	3.815138	1.975822	-1.034471
5	6	0	2.969489	3.167618	-1.059000
6	6	0	1.549802	2.641818	-0.770636
7	7	0	2.759932	-1.231718	0.171409
8	6	0	4.985434	-0.566105	-0.464143
9	6	0	5.457356	-1.846065	-0.195119
10	1	0	6.504258	-2.091205	-0.335165
11	6	0	4.558638	-2.800655	0.261473
12	6	0	3.217496	-2.454396	0.432425
13	46	0	0.680298	-0.531305	0.381742
14	6	0	-1.258953	0.202177	0.513060
15	6	0	-1.617031	1.103257	1.530856
16	6	0	-1.795543	0.380139	-0.776452
17	6	0	-2.635776	1.454614	-1.053574
18	6	0	-2.473802	2.165263	1.273992
19	6	0	-2.955891	2.324543	-0.019296
20	1	0	3.082949	3.636511	-2.036026
21	1	0	3.338048	3.841117	-0.279040

22	1	0	0.968087	2.566762	-1.699042
23	1	0	5.648988	0.214477	-0.818070
24	1	0	2.493675	-3.182175	0.790557
25	1	0	-1.230128	0.964786	2.538086
26	1	0	-1.534160	-0.307089	-1.577446
27	1	0	-3.037388	1.625072	-2.047340
28	1	0	-2.758773	2.871424	2.047244
29	9	0	-3.764586	3.370461	-0.280247
30	6	0	4.976954	-4.207159	0.564513
31	9	0	6.307690	-4.368405	0.490501
32	9	0	4.588299	-4.578345	1.801805
33	9	0	4.413941	-5.081515	-0.295643
34	6	0	0.784222	3.483612	0.213654
35	6	0	1.077029	3.428843	1.580252
36	1	0	1.842716	2.742399	1.937772
37	6	0	0.390255	4.240629	2.478546
38	1	0	0.619462	4.186842	3.539479
39	6	0	-0.587876	5.122583	2.018592
40	1	0	-1.122045	5.757110	2.720607
41	6	0	-0.883092	5.181846	0.658424
42	1	0	-1.651511	5.859063	0.295050
43	6	0	-0.202693	4.360991	-0.238858
44	1	0	-0.440490	4.394288	-1.300586
45	6	0	-0.240934	-2.311719	0.854430
46	6	0	-1.450486	-1.679155	1.303024
47	8	0	-2.680466	-2.043545	0.906330
48	6	0	-0.339025	-3.254428	-0.323923
49	1	0	0.400171	-2.612453	1.688974
50	1	0	-1.475887	-1.326799	2.330759
51	1	0	-0.183881	-2.707593	-1.263811
52	1	0	0.453738	-4.005425	-0.270753
53	6	0	-1.719681	-3.901220	-0.358617
54	6	0	-2.794594	-2.838380	-0.304558
55	1	0	-2.690690	-2.152366	-1.158567
56	1	0	-1.848277	-4.583450	0.492206
57	1	0	-1.846748	-4.487535	-1.273925
58	6	0	-4.213040	-3.382336	-0.270814
59	1	0	-4.337558	-4.015345	0.621021
60	1	0	-4.914306	-2.546687	-0.177673
61	8	0	-4.535010	-4.065206	-1.462894
62	1	0	-4.132162	-4.939835	-1.411361

E(M06L)= -1904.95636692 A.U.

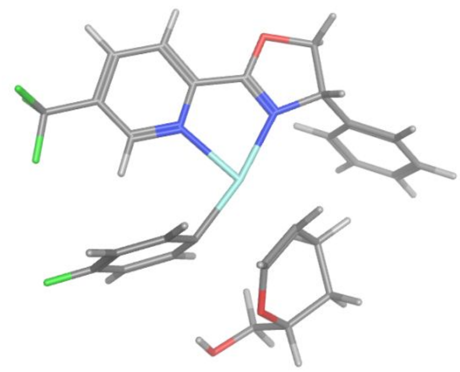

Center Number	Atomic Number	Atomic Type	Coordinates (Angstroms) X	Y	Z
1	6	0	3.556108	-0.230266	0.001595
2	6	0	2.974181	1.092905	-0.217066
3	7	0	1.704541	1.309659	-0.136305
4	8	0	3.774506	2.112793	-0.513654
5	6	0	2.908345	3.290882	-0.552024
6	6	0	1.475306	2.729786	-0.461006
7	7	0	2.642480	-1.187938	0.263990
8	6	0	4.922032	-0.486091	-0.048769
9	6	0	5.359167	-1.783233	0.186999
10	1	0	6.417364	-2.024307	0.158874
11	6	0	4.415273	-2.767479	0.466963
12	6	0	3.065366	-2.428753	0.495544
13	46	0	0.518577	-0.472116	0.283351
14	6	0	-0.322947	-2.344210	0.618325
15	6	0	-0.143117	-2.742460	1.953790
16	6	0	-0.184411	-3.300963	-0.401112
17	6	0	0.157534	-4.616319	-0.104073
18	6	0	0.202316	-4.054530	2.267753
19	6	0	0.348576	-4.965363	1.227937
20	1	0	3.117761	3.824386	-1.478788
21	1	0	3.178120	3.916292	0.303905
22	1	0	0.977558	2.770168	-1.437756
23	1	0	5.621967	0.312879	-0.264463
24	1	0	2.300473	-3.168735	0.710328
25	1	0	-0.247253	-2.018190	2.758282
26	1	0	-0.350686	-3.016161	-1.437558
27	1	0	0.271083	-5.365689	-0.880726
28	1	0	0.359687	-4.372487	3.293539
29	9	0	0.677321	-6.236665	1.522920
30	6	0	4.850773	-4.177717	0.721734
31	9	0	3.837936	-4.952256	1.152109
32	9	0	5.348469	-4.748770	-0.394520
33	9	0	5.825550	-4.228286	1.650732
34	6	0	0.605742	3.414731	0.558939
35	6	0	0.882096	3.285374	1.924069
36	1	0	1.728565	2.680831	2.246376

37	6	0	0.075620	3.915224	2.866888
38	1	0	0.293603	3.803936	3.925604
39	6	0	-1.011604	4.685806	2.454078
40	1	0	-1.641239	5.177176	3.190684
41	6	0	-1.287943	4.823544	1.095433
42	1	0	-2.133508	5.422465	0.768588
43	6	0	-0.483797	4.186112	0.151922
44	1	0	-0.702511	4.281876	-0.909639
45	6	0	-1.438427	0.190100	0.308108
46	6	0	-1.938041	-1.157090	0.228694
47	8	0	-2.876573	-1.656352	1.060292
48	6	0	-1.828165	0.991466	1.522651
49	1	0	-1.459400	0.706402	-0.656464
50	1	0	-2.046615	-1.582687	-0.765948
51	6	0	-3.266290	0.642149	1.900829
52	1	0	-3.940980	0.946193	1.093041
53	1	0	-3.577273	1.181131	2.801724
54	6	0	-3.419126	-0.840569	2.154677
55	1	0	-4.474772	-1.130643	2.147862
56	1	0	-1.725009	2.060206	1.321712
57	1	0	-1.160027	0.768267	2.364925
58	6	0	-2.803541	-1.309457	3.459029
59	1	0	-1.773060	-0.927313	3.553305
60	1	0	-3.375579	-0.869976	4.283050
61	8	0	-2.862680	-2.708483	3.610234
62	1	0	-2.466524	-3.082884	2.811588

E(M06L)= -1904.95678746 A.U.

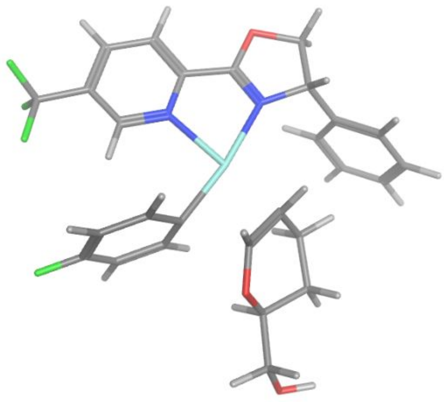

Center Number	Atomic Number	Atomic Type	Coordinates (Angstroms) X	Y	Z
1	6	0	3.560146	-0.193795	-0.122663
2	6	0	2.960305	1.122516	-0.336011
3	7	0	1.688952	1.320336	-0.244286
4	8	0	3.743180	2.156180	-0.633242
5	6	0	2.858057	3.320926	-0.664850
6	6	0	1.434182	2.739119	-0.548989

7	7	0	2.660780	-1.163562	0.148157
8	6	0	4.928800	-0.431588	-0.182609
9	6	0	5.385537	-1.722416	0.051069
10	1	0	6.446538	-1.949186	0.012985
11	6	0	4.456779	-2.718876	0.337530
12	6	0	3.102757	-2.397946	0.378954
13	46	0	0.531386	-0.465773	0.215452
14	6	0	-0.317811	-2.303598	0.639639
15	6	0	-0.183387	-2.601491	2.008650
16	6	0	-0.194968	-3.340442	-0.300951
17	6	0	0.084066	-4.641110	0.104860
18	6	0	0.102721	-3.897820	2.428349
19	6	0	0.233061	-4.890222	1.464606
20	1	0	3.045118	3.851252	-1.598307
21	1	0	3.132227	3.956186	0.182276
22	1	0	0.914252	2.784278	-1.513761
23	1	0	5.615893	0.376387	-0.406030
24	1	0	2.350397	-3.150037	0.597344
25	1	0	-0.274883	-1.812677	2.751305
26	1	0	-0.320991	-3.129150	-1.360254
27	1	0	0.185315	-5.453599	-0.607533
28	1	0	0.229493	-4.143886	3.477762
29	9	0	0.504357	-6.147695	1.864013
30	6	0	4.912937	-4.115406	0.627737
31	9	0	3.887653	-4.986787	0.662628
32	9	0	5.788146	-4.550803	-0.299135
33	9	0	5.541974	-4.186537	1.819447
34	6	0	0.574612	3.395920	0.498707
35	6	0	0.896188	3.274588	1.854832
36	1	0	1.768681	2.694083	2.151030
37	6	0	0.103915	3.884795	2.822356
38	1	0	0.358969	3.781854	3.873588
39	6	0	-1.015995	4.625527	2.443782
40	1	0	-1.633802	5.102581	3.199577
41	6	0	-1.340957	4.750656	1.094703
42	1	0	-2.212954	5.325136	0.794473
43	6	0	-0.549926	4.133914	0.126550
44	1	0	-0.804150	4.223037	-0.927707
45	6	0	-1.428588	0.188743	0.316086
46	6	0	-1.941500	-1.141624	0.130587
47	8	0	-2.911516	-1.687160	0.882921
48	6	0	-1.792014	0.893520	1.601921
49	1	0	-1.450174	0.778369	-0.605153
50	1	0	-1.999343	-1.520416	-0.886820
51	6	0	-3.168613	0.434798	2.071770
52	1	0	-3.946284	0.775460	1.375046
53	1	0	-3.398962	0.856128	3.055312
54	6	0	-3.214050	-1.073463	2.164701
55	1	0	-2.462309	-1.430055	2.884447
56	1	0	-1.758334	1.977168	1.460168
57	1	0	-1.058423	0.661408	2.386241

58	6	0	-4.574885	-1.634263	2.543327
59	1	0	-5.312270	-1.338315	1.781511
60	1	0	-4.526192	-2.728007	2.542542
61	8	0	-4.955632	-1.235593	3.842168
62	1	0	-5.267860	-0.325068	3.783493

E(M06L)= -1904.96082669 A.U.

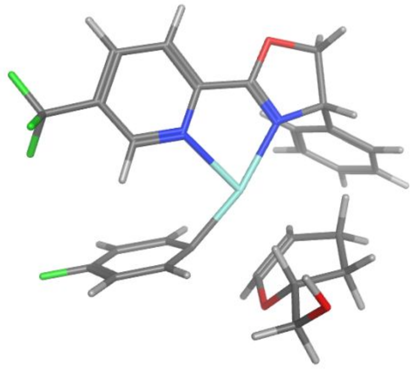

Center Number	Atomic Number	Atomic Type	Coordinates (Angstroms) X	Y	Z
1	6	0	3.712023	-0.123858	-0.150848
2	6	0	3.149031	1.204134	-0.389917
3	7	0	1.876336	1.413226	-0.424380
4	8	0	3.966717	2.243551	-0.525287
5	6	0	3.100947	3.413424	-0.674447
6	6	0	1.662643	2.865805	-0.543521
7	7	0	2.779711	-1.070349	0.095732
8	6	0	5.076014	-0.387009	-0.133478
9	6	0	5.498096	-1.680476	0.157612
10	1	0	6.554769	-1.922901	0.188258
11	6	0	4.537964	-2.650485	0.417023
12	6	0	3.187306	-2.304483	0.371862
13	46	0	0.654993	-0.281090	0.206444
14	6	0	-0.153836	-1.997429	1.082713
15	6	0	0.206701	-2.053115	2.438786
16	6	0	-0.177604	-3.176028	0.326373
17	6	0	0.210535	-4.387534	0.892811
18	6	0	0.611735	-3.254555	3.013115
19	6	0	0.612366	-4.397732	2.223036
20	9	0	1.002563	-5.565373	2.772463
21	1	0	3.309405	3.848806	-1.652627
22	1	0	3.375840	4.120946	0.110279
23	1	0	1.092610	3.065298	-1.457513
24	1	0	5.789326	0.404032	-0.334269
25	1	0	2.409694	-3.038210	0.569349
26	1	0	0.176085	-1.152072	3.047458
27	1	0	-0.501589	-3.153943	-0.710925
28	1	0	0.210654	-5.309474	0.320290

29	1	0	0.912386	-3.316378	4.054060
30	6	0	4.901905	-4.071620	0.718022
31	9	0	4.725523	-4.861923	-0.361875
32	9	0	6.183503	-4.197840	1.100123
33	9	0	4.128044	-4.579631	1.699837
34	6	0	0.881988	3.377028	0.642054
35	6	0	1.257970	3.021454	1.942502
36	1	0	2.126310	2.383103	2.099938
37	6	0	0.522430	3.470616	3.035149
38	1	0	0.820762	3.187541	4.040875
39	6	0	-0.597238	4.279412	2.839091
40	1	0	-1.173759	4.626330	3.692139
41	6	0	-0.973868	4.640789	1.547484
42	1	0	-1.845267	5.270150	1.388910
43	6	0	-0.237124	4.189233	0.453981
44	1	0	-0.535767	4.458705	-0.557162
45	6	0	-1.240062	0.519841	0.424464
46	6	0	-1.746415	-0.816262	0.619801
47	8	0	-2.363880	-1.471366	-0.381199
48	6	0	-1.678826	1.313955	-0.790298
49	1	0	-1.135563	1.083314	1.354988
50	1	0	-2.174739	-1.079048	1.582780
51	1	0	-2.296298	2.151151	-0.442358
52	6	0	-2.451067	0.468159	-1.800180
53	1	0	-3.529489	0.518093	-1.605807
54	6	0	-2.040074	-0.982261	-1.703587
55	1	0	-0.940376	-1.070880	-1.822212
56	1	0	-2.293029	0.820653	-2.822332
57	1	0	-0.807078	1.777730	-1.266184
58	6	0	-2.751329	-1.906163	-2.673938
59	1	0	-2.478220	-2.949204	-2.454663
60	1	0	-3.834181	-1.815647	-2.529164
61	8	0	-2.489379	-1.551709	-4.014376
62	1	0	-1.571730	-1.787729	-4.197356

E(M06L)= -1904.96007837 A.U.

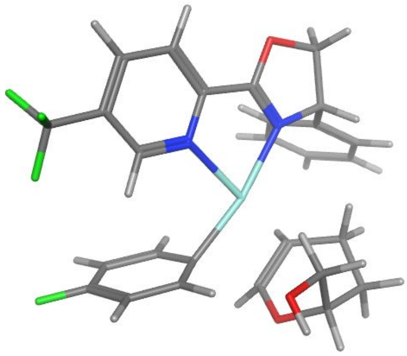

Center Number	Atomic Number	Atomic Type	Coordinates (Angstroms) X	Y	Z

1	6	0	3.788912	-0.093319	-0.005448
2	6	0	3.209659	1.221871	-0.274858
3	7	0	1.934631	1.416540	-0.298202
4	8	0	4.013507	2.265533	-0.453094
5	6	0	3.132255	3.419244	-0.634549
6	6	0	1.701724	2.860482	-0.464548
7	7	0	2.870029	-1.044061	0.265174
8	6	0	5.157739	-0.338770	0.013337
9	6	0	5.594326	-1.616415	0.340459
10	1	0	6.654720	-1.846426	0.376422
11	6	0	4.645729	-2.592527	0.630613
12	6	0	3.292985	-2.267445	0.574065
13	46	0	0.731604	-0.287781	0.313695
14	6	0	-0.115151	-2.037523	1.095295
15	6	0	0.176085	-2.118703	2.467782
16	6	0	-0.085907	-3.195994	0.309635
17	6	0	0.293827	-4.414163	0.870125
18	6	0	0.571385	-3.326784	3.033537
19	6	0	0.629021	-4.450973	2.217581
20	9	0	1.006190	-5.626581	2.760643
21	1	0	3.323099	3.819439	-1.631199
22	1	0	3.408924	4.158706	0.119361
23	1	0	1.116182	3.020357	-1.376555
24	1	0	5.860422	0.454988	-0.212554
25	1	0	2.526322	-3.005959	0.786331
26	1	0	0.097171	-1.234157	3.095933
27	1	0	-0.377603	-3.149533	-0.738106
28	1	0	0.332051	-5.324248	0.279594
29	1	0	0.820649	-3.409427	4.086616
30	6	0	5.073275	-3.981795	0.990403
31	9	0	4.052377	-4.714768	1.472933
32	9	0	5.570884	-4.639018	-0.077592
33	9	0	6.043271	-3.969615	1.925468
34	6	0	0.934149	3.410428	0.711895
35	6	0	1.302007	3.071119	2.018827
36	1	0	2.150158	2.409227	2.188011
37	6	0	0.583470	3.566528	3.102841
38	1	0	0.874879	3.295097	4.113795
39	6	0	-0.510012	4.406475	2.891801
40	1	0	-1.073130	4.789590	3.738278
41	6	0	-0.877417	4.753012	1.593471
42	1	0	-1.727880	5.407403	1.423180
43	6	0	-0.158118	4.255155	0.508548
44	1	0	-0.448696	4.515513	-0.507313
45	6	0	-1.182702	0.479856	0.429865
46	6	0	-1.679103	-0.869057	0.572755
47	8	0	-2.304314	-1.522224	-0.428848
48	6	0	-1.570639	1.287920	-0.793032
49	1	0	-1.149608	1.020019	1.379765
50	1	0	-2.113381	-1.138475	1.531641
51	1	0	-1.908867	2.277085	-0.468688

52	6	0	-2.664268	0.584141	-1.585127
53	1	0	-3.624242	0.676224	-1.065757
54	6	0	-2.351499	-0.889238	-1.741855
55	1	0	-3.191505	-1.405061	-2.220860
56	1	0	-2.785716	1.037269	-2.573060
57	1	0	-0.700139	1.467605	-1.436731
58	6	0	-1.096878	-1.161439	-2.553522
59	1	0	-1.176374	-0.580752	-3.486338
60	1	0	-0.199640	-0.801339	-2.030378
61	8	0	-0.870251	-2.532979	-2.807949
62	1	0	-1.642579	-2.871376	-3.278607

E(M06L)= -1904.98958723 A.U.

Note – only product-like structures were obtained

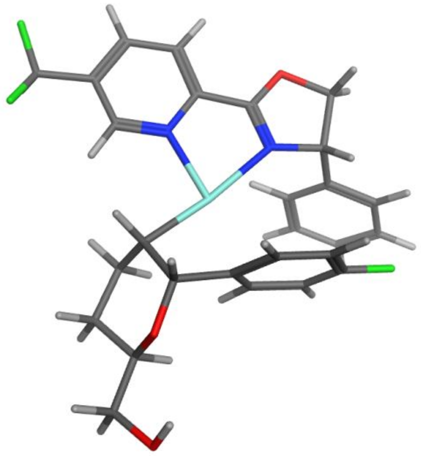

Center Number	Atomic Number	Atomic Type	Coordinates (Angstroms) X	Y	Z
1	6	0	3.187132	0.009834	-0.240566
2	6	0	2.591159	1.322653	-0.482987
3	7	0	1.326983	1.545002	-0.374812
4	8	0	3.396986	2.341334	-0.781846
5	6	0	2.500499	3.464293	-1.051501
6	6	0	1.137770	2.996698	-0.503077
7	7	0	2.318467	-0.944331	0.171207
8	6	0	4.545862	-0.232672	-0.394397
9	6	0	5.034862	-1.500927	-0.109219
10	1	0	6.090531	-1.725524	-0.219130
11	6	0	4.141445	-2.476225	0.317234
12	6	0	2.790021	-2.161165	0.443646
13	46	0	0.231282	-0.327091	0.258945
14	6	0	-2.021842	-0.355957	0.066994
15	6	0	-1.904887	0.544589	1.161581
16	6	0	-2.283067	0.188704	-1.216553
17	6	0	-2.459142	1.545841	-1.400929
18	6	0	-2.104403	1.919207	0.982662

19	6	0	-2.377876	2.385327	-0.288465
20	1	0	2.492331	3.617878	-2.133311
21	1	0	2.912059	4.342617	-0.553825
22	1	0	0.344635	3.201867	-1.230241
23	1	0	5.201649	0.561598	-0.731485
24	1	0	2.070270	-2.905801	0.770398
25	1	0	-1.749539	0.174424	2.169897
26	1	0	-2.365134	-0.488166	-2.063098
27	1	0	-2.664897	1.968010	-2.378740
28	1	0	-2.039862	2.610896	1.815744
29	9	0	-2.561360	3.702885	-0.475464
30	6	0	4.620617	-3.847724	0.684853
31	9	0	5.686672	-4.213632	-0.048416
32	9	0	4.992349	-3.905742	1.980523
33	9	0	3.662475	-4.776072	0.506810
34	6	0	0.762609	3.622807	0.822715
35	6	0	1.110667	3.034720	2.041907
36	1	0	1.638074	2.083050	2.056245
37	6	0	0.770040	3.652679	3.243755
38	1	0	1.040875	3.183083	4.185583
39	6	0	0.084229	4.865338	3.239624
40	1	0	-0.183437	5.344552	4.177255
41	6	0	-0.257098	5.461494	2.026181
42	1	0	-0.792868	6.406795	2.014299
43	6	0	0.078996	4.842370	0.826022
44	1	0	-0.198082	5.300931	-0.120818
45	6	0	-0.562566	-2.127405	0.818253
46	6	0	-1.933099	-1.872411	0.231114
47	8	0	-3.037444	-2.474568	0.901869
48	6	0	-0.484768	-2.320954	2.316076
49	1	0	-0.003181	-2.882045	0.254753
50	1	0	-1.968902	-2.290134	-0.779989
51	1	0	-0.511721	-1.358421	2.843779
52	1	0	0.467737	-2.789103	2.584294
53	6	0	-1.674064	-3.155287	2.797423
54	1	0	-1.667776	-3.234491	3.890075
55	1	0	-1.616818	-4.173846	2.391726
56	6	0	-2.966858	-2.514691	2.336480
57	1	0	-3.039379	-1.487008	2.734028
58	6	0	-4.204445	-3.279553	2.750309
59	1	0	-4.109511	-4.321292	2.396680
60	1	0	-4.269987	-3.308397	3.842987
61	8	0	-5.384251	-2.671223	2.271156
62	1	0	-5.225316	-2.524016	1.329685

E(M06L)= -1904.95284528 A.U.

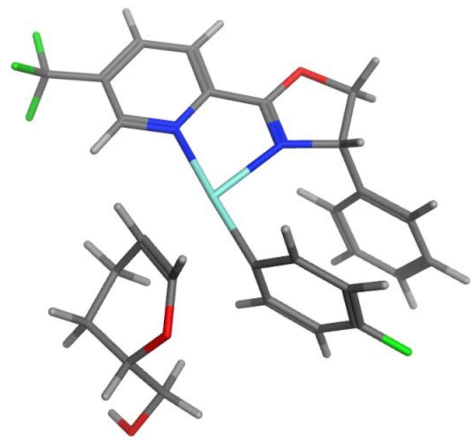

Center Number	Atomic Number	Atomic Type	Coordinates (Angstroms) X	Y	Z
1	6	0	3.419647	-0.196064	-0.239659
2	6	0	2.827104	1.116696	-0.490506
3	7	0	1.560834	1.330791	-0.394951
4	8	0	3.625424	2.130302	-0.819670
5	6	0	2.743245	3.279872	-1.010047
6	6	0	1.327487	2.756276	-0.668936
7	7	0	2.528195	-1.156155	0.106186
8	6	0	4.782561	-0.438441	-0.342127
9	6	0	5.255531	-1.718188	-0.073073
10	1	0	6.314147	-1.942739	-0.139807
11	6	0	4.341523	-2.700282	0.283710
12	6	0	2.985081	-2.380490	0.358917
13	46	0	0.431452	-0.486332	0.209547
14	6	0	-1.500571	0.249598	0.366249
15	6	0	-1.711462	0.801718	1.640549
16	6	0	-2.103212	0.852918	-0.749503
17	6	0	-2.877732	1.998206	-0.604524
18	6	0	-2.486421	1.943497	1.803902
19	6	0	-3.051051	2.519254	0.672339
20	1	0	2.848195	3.602179	-2.047127
21	1	0	3.091558	4.070199	-0.341916
22	1	0	0.666080	2.838464	-1.538872
23	1	0	5.456924	0.361383	-0.625376
24	1	0	2.247020	-3.131459	0.629423
25	1	0	-1.255112	0.338437	2.512778
26	1	0	-1.970495	0.420155	-1.738630
27	1	0	-3.346833	2.484321	-1.454022
28	1	0	-2.647208	2.394656	2.778110
29	9	0	-3.796317	3.631566	0.819457
30	6	0	4.760889	-4.105343	0.591115
31	9	0	4.339645	-4.484214	1.815705
32	9	0	4.228946	-4.980291	-0.287549
33	9	0	6.094404	-4.256306	0.555616
34	6	0	0.680860	3.456590	0.500749

35	6	0	1.093636	3.191209	1.811053
36	1	0	1.862923	2.442470	1.993754
37	6	0	0.522406	3.875404	2.880313
38	1	0	0.845946	3.658707	3.894859
39	6	0	-0.461659	4.837245	2.650865
40	1	0	-0.907386	5.370230	3.486273
41	6	0	-0.874916	5.107857	1.348422
42	1	0	-1.649186	5.847532	1.163305
43	6	0	-0.308400	4.416525	0.279239
44	1	0	-0.636843	4.618307	-0.738637
45	6	0	-0.509189	-2.256435	0.679297
46	6	0	-1.808257	-1.779934	0.288626
47	8	0	-2.921856	-1.959459	1.018170
48	6	0	-0.385390	-2.712874	2.108243
49	1	0	-0.040709	-2.884877	-0.086002
50	1	0	-2.042725	-1.760291	-0.773250
51	1	0	-0.315193	-1.846541	2.781096
52	1	0	0.525175	-3.299202	2.253374
53	6	0	-1.633366	-3.525161	2.447412
54	1	0	-1.602259	-3.906211	3.473757
55	1	0	-1.691229	-4.399181	1.789345
56	6	0	-2.880645	-2.679964	2.290882
57	1	0	-3.765062	-3.324102	2.234645
58	6	0	-3.122144	-1.701874	3.435264
59	1	0	-2.225857	-1.102275	3.645009
60	1	0	-3.920284	-1.008729	3.148427
61	8	0	-3.560325	-2.395402	4.588206
62	1	0	-2.777345	-2.736843	5.035096

Appendix 2. X-ray Data

Single Crystal Diffraction. All measurements were made with a Rigaku Synergy-i instrument using CrysalisPro software for data collection and reduction.[1922] Solution was by direct methods and the structure was refined to convergence against F^2 using all unique reflections and the program SHELXL-2018 as implemented within WINGX.[20,2123,24] All H atom positions were observed by difference synthesis but were placed in idealised positions and refined in riding modes. C-H bond lengths were 0.95, 1.00 and 0.99 Å for sp^2 CH, sp^3 CH and CH_2 respectively and $U_{iso}(H)$ values were set to $1.2U_{eq}(C)$ of the appropriate parent atom. Selected crystallographic details and refinement parameters are given in **Table S6** and full crystallographic details in cif format have been deposited with the CCDC, reference number 2093498.

Table S6: Selected crystallographic and refinement data for **Fc-3a**.

**Structure**	**Fc-3a**
**Formula**	$C_{23}H_{23}FFeO_3$
**FW**	422.26
**Crystal System**	orthorhombic
**Space Group**	$P2_12_12_1$
**Wavelengh (Å)**	1.54184
a **(Å)**	5.7957(1)
b **(Å)**	11.4769(1)
c **(Å)**	28.9252(3)
**Volume (Å³)**	1924.01(4)
**Z**	4
**Temperature (K)**	100(2)
**2θmax (°)**	142.928
**Measured Reflections**	20024
**Unique Reflections**	3712
**Observed rflns [I > 2σ(I)]**	3580
**Rint**	0.0405
**No. parameters**	253
**Goodness of Fit**	1.044
**R [on F, obs rflns only]**	0.0295
**wR (on F^2, all data)**	0.0714
**Largest diff. peak/hole (e $Å^{-3}$)**	0.445/-0.385
**Flack parameter**	-0.0095(17)

From this, the following X-ray crystal structure was generated (**Figure S5**):

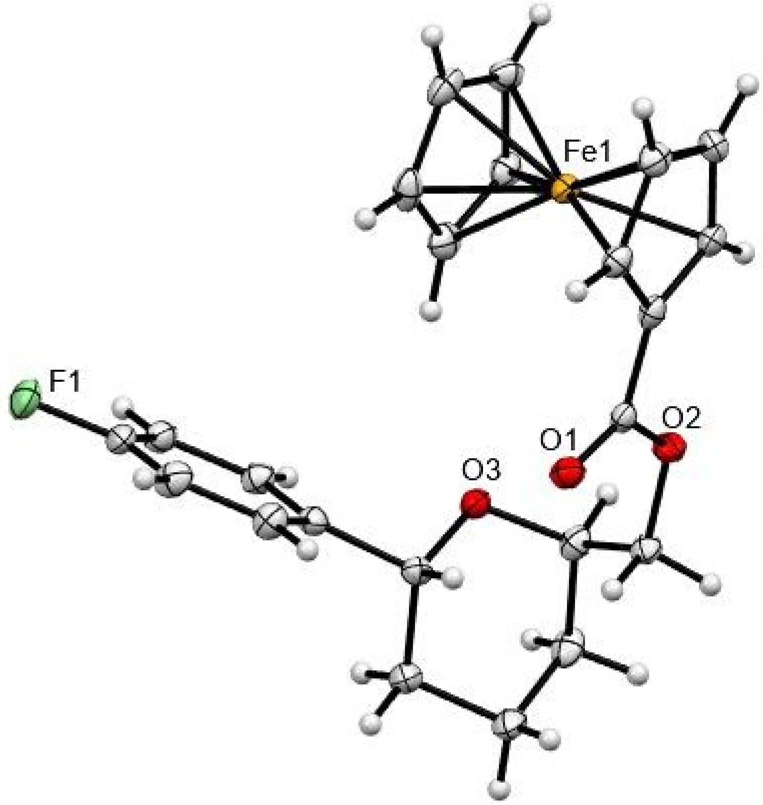

Figure S5: Molecular structure of **Fc-3a** with non-H atoms shown as 50 % probability ellipsoids and H atoms as small spheres of arbitrary size.

Table S7. Crystal data and structure refinement.

Empirical formula	C23 H23 F Fe O3	
Formula weight	422.26	
Temperature	100(2) K	
Wavelength	1.54184 Å	
Crystal system	Orthorhombic	
Space group	$P\ 2_1\ 2_1\ 2_1$	
Unit cell dimensions	a = 5.79570(10) Å	a= 90°.
	b = 11.47690(10) Å	b= 90°.
	c = 28.9252(3) Å	g = 90°.
Volume	1924.01(4) $Å^3$	
Z	4	
Density (calculated)	1.458 Mg/m^3	
Absorption coefficient	6.547 mm^{-1}	
F(000)	880	
Crystal size	0.33 x 0.04 x 0.02 mm^3	
Theta range for data collection	3.056 to 71.464°.	
Index ranges	-7<=h<=6, -14<=k<=14, -35<=l<=35	
Reflections collected	20024	
Independent reflections	3712 [R(int) = 0.0405]	
Completeness to theta = 70.000°	100.0 %	
Absorption correction	Semi-empirical from equivalents	
Max. and min. transmission	1.00000 and 0.30736	
Refinement method	Full-matrix least-squares on F^2	
Data / restraints / parameters	3712 / 0 / 253	
Goodness-of-fit on F^2	1.044	
Final R indices [I>2sigma(I)]	R1 = 0.0295, wR2 = 0.0703	
R indices (All spectroscopic data)	R1 = 0.0313, wR2 = 0.0714	
Absolute structure parameter	-0.0095(17)	
Extinction coefficient	n/a	
Largest diff. peak and hole	0.445 and -0.385 e.$Å^{-3}$	

Table S8. Atomic coordinates (x 10^4) and equivalent isotropic displacement parameters ($Å^2$x 10^3). U(eq) is defined as one third of the trace of the orthogonalized U^{ij} tensor.

	x	y	z	U(eq)
Fe(1)	-321(1)	-7286(1)	-3172(1)	15(1)
F(1)	-5433(3)	-1218(2)	-3124(1)	29(1)
O(1)	-4499(4)	-6967(2)	-4164(1)	20(1)
O(2)	-960(3)	-7312(2)	-4467(1)	19(1)
O(3)	-1006(4)	-4732(2)	-4516(1)	19(1)
C(1)	2109(5)	-6001(3)	-3231(1)	21(1)
C(2)	-126(6)	-5525(2)	-3306(1)	23(1)
C(3)	-1470(6)	-5745(3)	-2899(1)	23(1)
C(4)	-44(6)	-6336(3)	-2576(1)	24(1)
C(5)	2152(6)	-6496(3)	-2780(1)	22(1)
C(6)	-1748(5)	-8054(3)	-3733(1)	17(1)
C(7)	-3115(6)	-8296(3)	-3332(1)	19(1)
C(8)	-1686(6)	-8886(3)	-3011(1)	21(1)
C(9)	558(5)	-9020(2)	-3210(1)	20(1)
C(10)	536(6)	-8499(2)	-3657(1)	18(1)
C(11)	-2570(5)	-7382(3)	-4132(1)	16(1)
C(12)	-1533(5)	-6650(3)	-4877(1)	18(1)
C(13)	-263(5)	-5495(2)	-4882(1)	19(1)
C(14)	-484(6)	-4905(3)	-5353(1)	23(1)
C(15)	-2954(6)	-4500(3)	-5436(1)	25(1)
C(16)	-3718(6)	-3735(3)	-5032(1)	22(1)
C(17)	-3357(5)	-4335(3)	-4566(1)	17(1)
C(18)	-3845(5)	-3524(3)	-4167(1)	18(1)
C(19)	-5946(5)	-3605(3)	-3934(1)	22(1)
C(20)	-6485(6)	-2833(3)	-3579(1)	24(1)
C(21)	-4905(6)	-1988(3)	-3467(1)	22(1)
C(22)	-2809(6)	-1880(3)	-3685(1)	21(1)
C(23)	-2290(5)	-2665(3)	-4038(1)	20(1)

Table S9. Bond lengths [Å] and angles [°].

Fe(1)-C(6)	2.024(3)
Fe(1)-C(10)	2.036(3)
Fe(1)-C(5)	2.040(3)
Fe(1)-C(7)	2.044(3)
Fe(1)-C(4)	2.046(3)
Fe(1)-C(1)	2.047(3)
Fe(1)-C(3)	2.048(3)
Fe(1)-C(8)	2.053(3)
Fe(1)-C(9)	2.056(3)
Fe(1)-C(2)	2.061(3)
F(1)-C(21)	1.364(3)
O(1)-C(11)	1.219(4)
O(2)-C(11)	1.348(3)
O(2)-C(12)	1.447(3)
O(3)-C(13)	1.438(3)
O(3)-C(17)	1.444(4)
C(1)-C(2)	1.422(5)
C(1)-C(5)	1.424(4)
C(1)-H(1)	0.9500
C(2)-C(3)	1.432(4)
C(2)-H(2)	0.9500
C(3)-C(4)	1.420(5)
C(3)-H(3)	0.9500
C(4)-C(5)	1.415(5)
C(4)-H(4)	0.9500
C(5)-H(5)	0.9500
C(6)-C(7)	1.434(4)
C(6)-C(10)	1.436(4)
C(6)-C(11)	1.465(4)
C(7)-C(8)	1.416(4)
C(7)-H(7)	0.9500
C(8)-C(9)	1.431(4)
C(8)-H(8)	0.9500
C(9)-C(10)	1.423(4)
C(9)-H(9)	0.9500
C(10)-H(10)	0.9500

C(12)-C(13)	1.517(4)
C(12)-H(12A)	0.9900
C(12)-H(12B)	0.9900
C(13)-C(14)	1.526(4)
C(13)-H(13)	1.0000
C(14)-C(15)	1.525(5)
C(14)-H(14A)	0.9900
C(14)-H(14B)	0.9900
C(15)-C(16)	1.527(4)
C(15)-H(15A)	0.9900
C(15)-H(15B)	0.9900
C(16)-C(17)	1.529(4)
C(16)-H(16A)	0.9900
C(16)-H(16B)	0.9900
C(17)-C(18)	1.509(4)
C(17)-H(17)	1.0000
C(18)-C(23)	1.387(4)
C(18)-C(19)	1.394(4)
C(19)-C(20)	1.393(4)
C(19)-H(19)	0.9500
C(20)-C(21)	1.373(5)
C(20)-H(20)	0.9500
C(21)-C(22)	1.374(5)
C(22)-C(23)	1.394(4)
C(22)-H(22)	0.9500
C(23)-H(23)	0.9500
C(6)-Fe(1)-C(10)	41.44(13)
C(6)-Fe(1)-C(5)	157.89(13)
C(10)-Fe(1)-C(5)	121.00(13)
C(6)-Fe(1)-C(7)	41.28(12)
C(10)-Fe(1)-C(7)	69.55(13)
C(5)-Fe(1)-C(7)	159.06(13)
C(6)-Fe(1)-C(4)	160.02(14)
C(10)-Fe(1)-C(4)	157.64(13)
C(5)-Fe(1)-C(4)	40.52(13)
C(7)-Fe(1)-C(4)	123.65(13)
C(6)-Fe(1)-C(1)	121.86(13)

C(10)-Fe(1)-C(1)	105.52(13)
C(5)-Fe(1)-C(1)	40.77(12)
C(7)-Fe(1)-C(1)	159.21(12)
C(4)-Fe(1)-C(1)	68.43(13)
C(6)-Fe(1)-C(3)	123.51(13)
C(10)-Fe(1)-C(3)	159.18(13)
C(5)-Fe(1)-C(3)	68.30(13)
C(7)-Fe(1)-C(3)	108.60(13)
C(4)-Fe(1)-C(3)	40.58(13)
C(1)-Fe(1)-C(3)	68.48(13)
C(6)-Fe(1)-C(8)	68.64(12)
C(10)-Fe(1)-C(8)	68.86(12)
C(5)-Fe(1)-C(8)	122.82(13)
C(7)-Fe(1)-C(8)	40.45(12)
C(4)-Fe(1)-C(8)	108.37(13)
C(1)-Fe(1)-C(8)	158.26(13)
C(3)-Fe(1)-C(8)	124.01(13)
C(6)-Fe(1)-C(9)	68.76(12)
C(10)-Fe(1)-C(9)	40.70(11)
C(5)-Fe(1)-C(9)	106.60(13)
C(7)-Fe(1)-C(9)	68.64(12)
C(4)-Fe(1)-C(9)	122.74(13)
C(1)-Fe(1)-C(9)	121.51(13)
C(3)-Fe(1)-C(9)	159.46(13)
C(8)-Fe(1)-C(9)	40.75(13)
C(6)-Fe(1)-C(2)	107.40(12)
C(10)-Fe(1)-C(2)	121.90(12)
C(5)-Fe(1)-C(2)	68.25(13)
C(7)-Fe(1)-C(2)	123.85(13)
C(4)-Fe(1)-C(2)	68.32(12)
C(1)-Fe(1)-C(2)	40.51(13)
C(3)-Fe(1)-C(2)	40.78(13)
C(8)-Fe(1)-C(2)	160.17(14)
C(9)-Fe(1)-C(2)	157.73(13)
C(11)-O(2)-C(12)	117.5(2)
C(13)-O(3)-C(17)	113.7(2)
C(2)-C(1)-C(5)	107.9(3)
C(2)-C(1)-Fe(1)	70.28(17)

C(5)-C(1)-Fe(1)	69.36(18)
C(2)-C(1)-H(1)	126.0
C(5)-C(1)-H(1)	126.0
Fe(1)-C(1)-H(1)	125.9
C(1)-C(2)-C(3)	107.7(3)
C(1)-C(2)-Fe(1)	69.20(17)
C(3)-C(2)-Fe(1)	69.13(17)
C(1)-C(2)-H(2)	126.2
C(3)-C(2)-H(2)	126.2
Fe(1)-C(2)-H(2)	127.1
C(4)-C(3)-C(2)	107.9(3)
C(4)-C(3)-Fe(1)	69.62(18)
C(2)-C(3)-Fe(1)	70.10(17)
C(4)-C(3)-H(3)	126.0
C(2)-C(3)-H(3)	126.0
Fe(1)-C(3)-H(3)	125.8
C(5)-C(4)-C(3)	108.1(3)
C(5)-C(4)-Fe(1)	69.52(17)
C(3)-C(4)-Fe(1)	69.80(17)
C(5)-C(4)-H(4)	125.9
C(3)-C(4)-H(4)	125.9
Fe(1)-C(4)-H(4)	126.3
C(4)-C(5)-C(1)	108.3(3)
C(4)-C(5)-Fe(1)	69.96(17)
C(1)-C(5)-Fe(1)	69.87(18)
C(4)-C(5)-H(5)	125.8
C(1)-C(5)-H(5)	125.8
Fe(1)-C(5)-H(5)	125.9
C(7)-C(6)-C(10)	108.3(3)
C(7)-C(6)-C(11)	124.0(3)
C(10)-C(6)-C(11)	127.5(3)
C(7)-C(6)-Fe(1)	70.13(17)
C(10)-C(6)-Fe(1)	69.74(17)
C(11)-C(6)-Fe(1)	122.3(2)
C(8)-C(7)-C(6)	107.5(3)
C(8)-C(7)-Fe(1)	70.12(18)
C(6)-C(7)-Fe(1)	68.59(17)
C(8)-C(7)-H(7)	126.3

C(6)-C(7)-H(7)	126.3
Fe(1)-C(7)-H(7)	126.6
C(7)-C(8)-C(9)	108.6(3)
C(7)-C(8)-Fe(1)	69.44(18)
C(9)-C(8)-Fe(1)	69.74(17)
C(7)-C(8)-H(8)	125.7
C(9)-C(8)-H(8)	125.7
Fe(1)-C(8)-H(8)	126.7
C(10)-C(9)-C(8)	108.2(3)
C(10)-C(9)-Fe(1)	68.89(16)
C(8)-C(9)-Fe(1)	69.51(17)
C(10)-C(9)-H(9)	125.9
C(8)-C(9)-H(9)	125.9
Fe(1)-C(9)-H(9)	127.3
C(9)-C(10)-C(6)	107.4(3)
C(9)-C(10)-Fe(1)	70.41(16)
C(6)-C(10)-Fe(1)	68.82(17)
C(9)-C(10)-H(10)	126.3
C(6)-C(10)-H(10)	126.3
Fe(1)-C(10)-H(10)	126.0
O(1)-C(11)-O(2)	123.8(3)
O(1)-C(11)-C(6)	124.3(3)
O(2)-C(11)-C(6)	111.8(2)
O(2)-C(12)-C(13)	110.8(2)
O(2)-C(12)-H(12A)	109.5
C(13)-C(12)-H(12A)	109.5
O(2)-C(12)-H(12B)	109.5
C(13)-C(12)-H(12B)	109.5
H(12A)-C(12)-H(12B)	108.1
O(3)-C(13)-C(12)	112.3(2)
O(3)-C(13)-C(14)	111.1(2)
C(12)-C(13)-C(14)	110.8(2)
O(3)-C(13)-H(13)	107.4
C(12)-C(13)-H(13)	107.4
C(14)-C(13)-H(13)	107.4
C(15)-C(14)-C(13)	110.9(3)
C(15)-C(14)-H(14A)	109.5
C(13)-C(14)-H(14A)	109.5

C(15)-C(14)-H(14B)	109.5
C(13)-C(14)-H(14B)	109.5
H(14A)-C(14)-H(14B)	108.1
C(14)-C(15)-C(16)	109.0(3)
C(14)-C(15)-H(15A)	109.9
C(16)-C(15)-H(15A)	109.9
C(14)-C(15)-H(15B)	109.9
C(16)-C(15)-H(15B)	109.9
H(15A)-C(15)-H(15B)	108.3
C(15)-C(16)-C(17)	112.2(2)
C(15)-C(16)-H(16A)	109.2
C(17)-C(16)-H(16A)	109.2
C(15)-C(16)-H(16B)	109.2
C(17)-C(16)-H(16B)	109.2
H(16A)-C(16)-H(16B)	107.9
O(3)-C(17)-C(18)	107.2(2)
O(3)-C(17)-C(16)	111.0(2)
C(18)-C(17)-C(16)	111.8(2)
O(3)-C(17)-H(17)	108.9
C(18)-C(17)-H(17)	108.9
C(16)-C(17)-H(17)	108.9
C(23)-C(18)-C(19)	119.0(3)
C(23)-C(18)-C(17)	121.5(3)
C(19)-C(18)-C(17)	119.4(3)
C(20)-C(19)-C(18)	120.6(3)
C(20)-C(19)-H(19)	119.7
C(18)-C(19)-H(19)	119.7
C(21)-C(20)-C(19)	118.3(3)
C(21)-C(20)-H(20)	120.9
C(19)-C(20)-H(20)	120.9
F(1)-C(21)-C(20)	118.6(3)
F(1)-C(21)-C(22)	118.3(3)
C(20)-C(21)-C(22)	123.1(3)
C(21)-C(22)-C(23)	117.9(3)
C(21)-C(22)-H(22)	121.0
C(23)-C(22)-H(22)	121.0
C(18)-C(23)-C(22)	121.1(3)
C(18)-C(23)-H(23)	119.5

C(22)-C(23)-H(23)	119.5

Table S10. Anisotropic displacement parameters ($Å^2$x 10^3). The anisotropic displacement factor exponent takes the form: $-2p^2[h^2a^{*2}U^{11} + ... + 2 h k a^* b^* U^{12}]$

	U^{11}	U^{22}	U^{33}	U^{23}	U^{13}	U^{12}
Fe(1)	16(1)	15(1)	13(1)	-1(1)	0(1)	-1(1)
F(1)	32(1)	32(1)	22(1)	-11(1)	1(1)	6(1)
O(1)	18(1)	26(1)	18(1)	-1(1)	-1(1)	1(1)
O(2)	21(1)	22(1)	15(1)	2(1)	2(1)	3(1)
O(3)	18(1)	21(1)	16(1)	-4(1)	-1(1)	1(1)
C(1)	23(2)	18(1)	21(2)	-2(1)	2(1)	-4(1)
C(2)	31(2)	15(1)	24(2)	0(1)	-3(1)	-5(1)
C(3)	15(2)	21(2)	33(2)	-10(1)	2(1)	-1(1)
C(4)	32(2)	23(2)	19(1)	-7(1)	2(1)	-8(2)
C(5)	20(2)	24(2)	21(2)	-2(1)	-4(1)	-2(1)
C(6)	17(2)	17(1)	17(1)	-5(1)	0(1)	-2(1)
C(7)	19(2)	19(2)	19(1)	-3(1)	-1(1)	-4(1)
C(8)	25(2)	18(1)	20(2)	1(1)	0(1)	-4(1)
C(9)	22(2)	17(1)	21(1)	-1(1)	-2(1)	5(1)
C(10)	22(2)	17(1)	16(1)	-5(1)	-1(1)	1(1)
C(11)	17(1)	16(1)	15(1)	-4(1)	-1(1)	-1(1)
C(12)	22(2)	18(2)	13(1)	0(1)	2(1)	1(1)
C(13)	17(2)	22(1)	18(1)	-3(1)	3(1)	-1(1)
C(14)	29(2)	22(2)	19(1)	-1(1)	8(1)	-3(1)
C(15)	36(2)	23(2)	15(1)	-1(1)	-2(1)	4(1)
C(16)	27(2)	21(2)	17(1)	-1(1)	-3(1)	4(1)
C(17)	16(2)	18(1)	16(1)	-1(1)	-2(1)	1(1)
C(18)	21(2)	17(1)	16(1)	1(1)	-3(1)	0(1)
C(19)	20(2)	23(2)	21(2)	-1(1)	2(1)	-2(1)
C(20)	19(2)	34(2)	19(1)	-1(1)	2(1)	0(2)
C(21)	29(2)	22(2)	14(1)	-3(1)	-2(1)	8(1)
C(22)	25(2)	20(1)	18(1)	-1(1)	-4(1)	-1(1)
C(23)	19(2)	23(2)	18(1)	-1(1)	1(1)	0(1)

Table S11. Hydrogen coordinates (x 10^4) and isotropic displacement parameters (Å^{2}x 10^3).

	x	y	z	U(eq)
H(1)	3354	-5990	-3445	25
H(2)	-637	-5132	-3576	28
H(3)	-3038	-5533	-2854	28
H(4)	-488	-6582	-2276	29
H(5)	3436	-6870	-2640	26
H(7)	-4692	-8097	-3289	23
H(8)	-2142	-9149	-2713	25
H(9)	1841	-9391	-3069	24
H(10)	1799	-8454	-3865	22
H(12A)	-3217	-6507	-4886	21
H(12B)	-1112	-7105	-5156	21
H(13)	1410	-5664	-4830	23
H(14A)	570	-4227	-5366	28
H(14B)	-26	-5459	-5598	28
H(15A)	-3989	-5182	-5464	29
H(15B)	-3038	-4051	-5728	29
H(16A)	-5371	-3540	-5069	26
H(16B)	-2832	-2998	-5038	26
H(17)	-4416	-5021	-4545	21
H(19)	-7020	-4193	-4019	26
H(20)	-7909	-2890	-3418	29
H(22)	-1747	-1288	-3598	26
H(23)	-848	-2611	-4193	24

Table S12. Torsion angles [°].

C(5)-C(1)-C(2)-C(3)	-0.8(3)
Fe(1)-C(1)-C(2)-C(3)	58.6(2)
C(5)-C(1)-C(2)-Fe(1)	-59.4(2)
C(1)-C(2)-C(3)-C(4)	0.9(3)
Fe(1)-C(2)-C(3)-C(4)	59.6(2)
C(1)-C(2)-C(3)-Fe(1)	-58.6(2)
C(2)-C(3)-C(4)-C(5)	-0.7(3)
Fe(1)-C(3)-C(4)-C(5)	59.2(2)
C(2)-C(3)-C(4)-Fe(1)	-59.9(2)
C(3)-C(4)-C(5)-C(1)	0.2(3)
Fe(1)-C(4)-C(5)-C(1)	59.5(2)
C(3)-C(4)-C(5)-Fe(1)	-59.3(2)
C(2)-C(1)-C(5)-C(4)	0.4(3)
Fe(1)-C(1)-C(5)-C(4)	-59.6(2)
C(2)-C(1)-C(5)-Fe(1)	60.0(2)
C(10)-C(6)-C(7)-C(8)	-0.1(3)
C(11)-C(6)-C(7)-C(8)	-175.8(3)
Fe(1)-C(6)-C(7)-C(8)	-59.6(2)
C(10)-C(6)-C(7)-Fe(1)	59.5(2)
C(11)-C(6)-C(7)-Fe(1)	-116.2(3)
C(6)-C(7)-C(8)-C(9)	-0.3(3)
Fe(1)-C(7)-C(8)-C(9)	-58.9(2)
C(6)-C(7)-C(8)-Fe(1)	58.6(2)
C(7)-C(8)-C(9)-C(10)	0.6(3)
Fe(1)-C(8)-C(9)-C(10)	-58.15(19)
C(7)-C(8)-C(9)-Fe(1)	58.7(2)
C(8)-C(9)-C(10)-C(6)	-0.6(3)
Fe(1)-C(9)-C(10)-C(6)	-59.14(19)
C(8)-C(9)-C(10)-Fe(1)	58.5(2)
C(7)-C(6)-C(10)-C(9)	0.4(3)
C(11)-C(6)-C(10)-C(9)	175.9(3)
Fe(1)-C(6)-C(10)-C(9)	60.15(19)
C(7)-C(6)-C(10)-Fe(1)	-59.7(2)
C(11)-C(6)-C(10)-Fe(1)	115.8(3)
C(12)-O(2)-C(11)-O(1)	3.4(4)
C(12)-O(2)-C(11)-C(6)	-178.8(2)

C(7)-C(6)-C(11)-O(1)	-0.4(5)
C(10)-C(6)-C(11)-O(1)	-175.3(3)
Fe(1)-C(6)-C(11)-O(1)	-87.1(3)
C(7)-C(6)-C(11)-O(2)	-178.3(3)
C(10)-C(6)-C(11)-O(2)	6.9(4)
Fe(1)-C(6)-C(11)-O(2)	95.1(3)
C(11)-O(2)-C(12)-C(13)	106.2(3)
C(17)-O(3)-C(13)-C(12)	-66.8(3)
C(17)-O(3)-C(13)-C(14)	58.0(3)
O(2)-C(12)-C(13)-O(3)	-66.2(3)
O(2)-C(12)-C(13)-C(14)	168.8(2)
O(3)-C(13)-C(14)-C(15)	-56.7(3)
C(12)-C(13)-C(14)-C(15)	68.9(3)
C(13)-C(14)-C(15)-C(16)	54.0(3)
C(14)-C(15)-C(16)-C(17)	-52.9(4)
C(13)-O(3)-C(17)-C(18)	-178.4(2)
C(13)-O(3)-C(17)-C(16)	-56.0(3)
C(15)-C(16)-C(17)-O(3)	53.5(3)
C(15)-C(16)-C(17)-C(18)	173.2(3)
O(3)-C(17)-C(18)-C(23)	45.6(4)
C(16)-C(17)-C(18)-C(23)	-76.3(4)
O(3)-C(17)-C(18)-C(19)	-137.1(3)
C(16)-C(17)-C(18)-C(19)	101.0(3)
C(23)-C(18)-C(19)-C(20)	0.3(5)
C(17)-C(18)-C(19)-C(20)	-177.0(3)
C(18)-C(19)-C(20)-C(21)	0.5(5)
C(19)-C(20)-C(21)-F(1)	179.1(3)
C(19)-C(20)-C(21)-C(22)	-0.9(5)
F(1)-C(21)-C(22)-C(23)	-179.5(3)
C(20)-C(21)-C(22)-C(23)	0.5(5)
C(19)-C(18)-C(23)-C(22)	-0.8(5)
C(17)-C(18)-C(23)-C(22)	176.5(3)
C(21)-C(22)-C(23)-C(18)	0.4(5)

References

1. Hickey, D. P.; Sandford, C.; Rhodes, Z.; Gensch, T.; Fries, L. R.; Sigman, M. S.; Minteer, S. D. Investigating the Role of Ligand Electronics on Stabilizing Electrocatalytically Relevant Low-Valent Co(I) Intermediates. *J. Am. Chem. Soc.* **2019**, *141*, 1382-1392.
2. Werner, E. W.; Mei, T. S.; Burckle, A. J.; Sigman, M. S. Enantioselective Heck Arylations of Acyclic Alkenyl Alcohols Using a Redox-Relay Strategy. *Science* **2012**, *338*, 1455-1458.
3. Patel, H. H.; Sigman, M. S. Enantioselective Palladium-Catalyzed Alkenylation of Trisubstituted Alkenols to form Allylic Quaternary Centers. *J. Am. Chem. Soc.* **2016**, *138*, 14226-14229.
4. Zhang, T.; Shen, H. C.; Xu, J. C.; Fan, T.; Han, Z. Y.; Gong, L. Z. Pd(II)-Catalyzed Asymmetric Oxidative Annulation of *N*-Alkoxyheteroaryl Amides and 1,3-Dienes. *Org. Lett.* **2019**, *21*, 2048-2051.
5. Zhang, C.; Santiago, C. B.; Crawford, J. M.; Sigman, M. S. Enantioselective Dehydrogenative Heck Arylations of Trisubstituted Alkenes with Indoles to Construct Quaternary Stereocenters. *J. Am. Chem. Soc.* **2015**, *137*, 15668-15671.
6. Ikawamoto, I. Synthesis and antifungal activity of novel azoles containing the tetrahydropyran ring. *Sankyo Kenkyusho Nenpo* **1985**, *37*, 40-56.
7. Karageorgis, G.; Dow, M.; Aimon, A.; Warriner, S.; Nelson, A. Activity-Directed Synthesis with Intermolecular Reactions: Development of a Fragment into a Range of Androgen Receptor Agonists. *Angew. Chem. Int. Ed.* **2015**, *54*, 13538-13544.
8. Carreño, M. C.; Des Mazery, R.; Urbano, A.; Colobert, F.; Solladié, G. Reductive Cyclizations of Hydroxysulfinyl Ketones: Enantioselective Access to Tetrahydropyran and Tetrahydrofuran Derivatives. *J. Org. Chem.* **2003**, *68*, 7779-7787
9. (a) Nagarjuna, B.; Thirupathi, B.; Rao, C. V.; Mohapatra, D. K. Chemoenzymatic total synthesis of four stereoisomers of centrolobine. *Tetrahedron Lett.* **2015**, *56*, 4916-4918; (b) Schmidt, B.; Hölter, F. A Stereodivergent Synthesis of All Stereoisomers of Centrolobine: Control of Selectivity by a Protecting-Group Manipulation. *Chem. Eur. J.* **2009**, *15*, 11948-11953.
10. *Molecular Operating Environment (MOE)*, 2019.01; Chemical Computing Group ULC, 1010 Sherbrooke St. West, Suite #910, Montreal, QC, Canada, H3A 2R7, **2019**.
11. *Gaussian 16, Revision B.01*, Frisch, M. J.; Trucks, G. W.; Schlegel, H. B.; Scuseria, G. E.; Robb, M. A.; Cheeseman, J. R.; Scalmani, G.; Barone, V.; Petersson, G. A.; Nakatsuji, H.; Li, X.; Caricato, M.; Marenich, A. V.; Bloino, J.; Janesko, B. G.; Gomperts, R.; Mennucci, B.; Hratchian, H. P.; Ortiz, J. V.; Izmaylov, A. F.; Sonnenberg, J. L.; Williams-Young, D.; Ding, F.; Lipparini, F.; Egidi, F.; Goings, J.; Peng, B.; Petrone, A.; Henderson, T.; Ranasinghe, D.; Zakrzewski, V. G.; Gao, J.; Rega, N.; Zheng, G.; Liang, W.; Hada, M.; Ehara, M.; Toyota, K.; Fukuda, R.; Hasegawa, J.; Ishida, M.; Nakajima, T.; Honda, Y.; Kitao, O.; Nakai, H.; Vreven, T.; Throssell, K.; Montgomery, J. A., Jr.; Peralta, J. E.; Ogliaro, F.; Bearpark, M. J.; Heyd, J. J.; Brothers, E. N.; Kudin, K. N.; Staroverov, V. N.; Keith, T. A.; Kobayashi, R.; Normand, J.; Raghavachari, K.; Rendell, A. P.; Burant, J. C.; Iyengar, S. S.; Tomasi, J.; Cossi, M.; Millam, J. M.; Klene, M.; Adamo, C.; Cammi, R.; Ochterski, J. W.; Martin, R. L.; Morokuma, K.; Farkas, O.; Foresman, J. B.; Fox, D. J. Gaussian, Inc., Wallingford CT, 2016.
12. Zhao, Y.; Truhlar, D. G. The M06 suite of density functionals for main group thermochemistry, thermochemical kinetics, noncovalent interactions, excited states, and transition elements: two new functionals and systematic testing of four M06-class functionals and 12 other functionals. *Theor. Chem. Acc.* **2008**, *120*, 215-241.
13. Frisch, M. J.; Pople, J. A.; Binkley, J. S. Self-Consistent Molecular Orbital Methods. 25. Supplementary Functions for Gaussian Basis Sets. *J. Chem. Phys.* **1984**, *80*, 3265-3269.
14. Dunning Jr, T. H.; Hay, P. J. in *Modern Theoretical Chemistry*, Ed. Schaefer III, H. F. Vol. 3, Plenum, New York, **1977**, 1-28.
15. Hay, P. J.; Wadt, W. R. Ab initio effective core potentials for molecular calculations – potentials for K to Au including the outermost core orbitals. *J. Chem. Phys.* **1985**, *82*, 299-310.
16. Schlegel, H. B. Optimization of Equilibrium Geometries and Transition Structures. *J. Comp. Chem.* **1982**, *3*, 214-218.
17. Marenich, V.; Cramer, C. J.; Truhlar, D. G. Universal solvation model based on solute electron density and a continuum model of the solvent defined by the bulk dielectric constant and atomic surface tensions. *J. Phys. Chem. B* **2009**, *113*, 6378-96.

18. Polo, E. C.; Wang, M. F.; Angnes, R. A.; Braga, A. A. C.; Correia, C. R. D. Enantioselective Heck Arylation of Acyclic Alkenol Aryl Ethers: Synthetic Applications and DFT Investigation of the Stereoselectivity. *Adv. Synth. Catal.* **2020**, *362*, 884-892.
19. *CrysAlisPRO*. Rigaku Ltd, Yarnton, Oxfordshire, England.
20. Sheldrick, G. M. SHELXT – Integrated space-group and crystal-structure determination. *Acta Cryst.* **2015**, *A71*, 3-8.
21. Farrugia, L. J. WinGX and ORTEP for Windows: an update. *.J. Appl. Cryst.* **2012**, *45*, 849-854.
